# Photochemical Deracemization of Chromanes and its Application to the Synthesis of Enantiopure Bioactive Compounds

**DOI:** 10.1002/anie.202521436

**Published:** 2025-12-23

**Authors:** Biki Ghosh, Maximilian Iglhaut, Daria Babushkina, Mike Pauls, Christoph Bannwarth, Thorsten Bach

**Affiliations:** ^1^ School of Natural Sciences Department of Chemistry and Catalysis Research Center Technische Universität München Lichtenbergstr. 4 85747 Garching Germany; ^2^ Institut für Physikalische Chemie RWTH Aachen University Melatener Str. 20 52074 Aachen Germany

**Keywords:** Asymmetric synthesis, C─H activation, Enantioselectivity, Oxygen heterocycles, Photochemistry

## Abstract

Chromanes are frequently encountered as chiral structure elements in active pharmaceutical ingredients (APIs). We have now discovered an access to enantiopure chromanes, which employs a 1:1 mixture of their enantiomers (racemate) in a photochemical deracemization reaction (21 examples, 71%–90% yield, 80%–99% *ee*). A chiral photocatalyst (10 mol%) acts by selective hydrogen abstraction at one chromane enantiomer and establishes a photostationary state in which the other enantiomer prevails. A thiol additive (20 mol%) was found to improve the enantioselectivity of the process. The mechanism of the reaction was investigated by experimental and quantum‐chemical studies. The oxygen atom of the chromane locks the rotation around the exocyclic C─C bond to the amide by forming an intramolecular hydrogen bond. Forward hydrogen atom transfer (HAT) occurs exclusively in one diastereomeric complex via a readily accessible transition state. Reasonable pathways for back HAT were identified which are in line with deuterium labeling experiments. The method was applied to the concise preparation of five chromane‐containing drugs (Doxazosin, Fidarestat, Nebivolol, Repinotan, Sarizotan) as single enantiomers.

## Graphical abstract

Various substituted chromane‐2‐carboxamides were successfully deracemized by employing a chiral benzophenone (**BP**, 10 mol%) as photocatalyst and an achiral thiol as additive (20 mol%). The method was applied to the concise enantioselective synthesis of five active pharmaceutical ingredients.

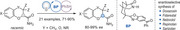

The three‐dimensional structure of a molecule is responsible for its biological activity. Proper binding to a given target requires a perfect positioning of functional groups into a defined environment.^[^ ^1^ ^]^ Against this background, it is not surprising that one enantiomer of a given chiral active pharmaceutical ingredient (API) is often superior in its performance when compared to its mirror image. Thus, the number of chiral drugs which are being administered as single enantiomers continues to grow at a rapid pace.^[^ ^2^ ^]^ Beyond sophisticated methodology for the selective preparation of an enantiomerically pure compound,^[^ ^3^ ^]^ its racemic mixture can potentially serve as an immediate precursor. Among all methods which promise access to a single enantiomer from a racemate, photochemical deracemization is the most straightforward and operationally least complicated technique.^[^ ^4^, ^5^, ^6^, ^7^, ^8^, ^9^, ^10^, ^11^, ^12^ ^]^ In a single step, a racemate is converted to either one of the two possible enantiomers by decoupling the events in which the existing stereogenic element is broken and re‐formed.

In an attempt to make enantiopure APIs accessible by photochemical deracemization, we identified 3,4‐dihydro‐2*H*‐1‐benzopyrans (chromanes) as a frequently encountered motif. The compounds are often substituted in 2‐position, which makes their C2 carbon atom a stereogenic center and renders them chiral.^[^ ^13^, ^14^ ^]^ Several APIs are known which carry either an (*R*)‐ or (*S*)‐configured chromane as pharmacophore and show an improved biological profile if administered in enantiomerically pure form. Examples include Doxazosin,^[^ ^15^ ^]^ Fidarestat,^[^ ^16^ ^]^ Nebivolol,^[^ ^17^, ^18^ ^]^ Repinotan,^[^ ^19^ ^]^ and Sarizotan.^[^ ^20^ ^]^ Synthetic pathways toward enantiomerically pure chromane‐2‐carboxylic acids and its derivatives have so far relied on chemical^[^ ^21^, ^22^ ^]^ or enzymatic^[^ ^23^, ^24^, ^25^ ^]^ resolution, on enantioselective reduction^[^ ^26^ ^]^ or addition,^[^ ^27^ ^]^ and on ex‐chiral‐pool synthesis.^[^ ^28^, ^29^ ^]^ We have now targeted the compound class by photochemical deracemization and identified the respective amides *rac*‐**1** as suitable, readily available starting materials (Scheme 1). The goal was to access either the (*R*)‐enantiomer **1** or its mirror image, the (*S*)‐enantiomer *ent*‐**1**, in high yield and with close to perfect enantiomeric excess (*ee*).

**Scheme 1:**
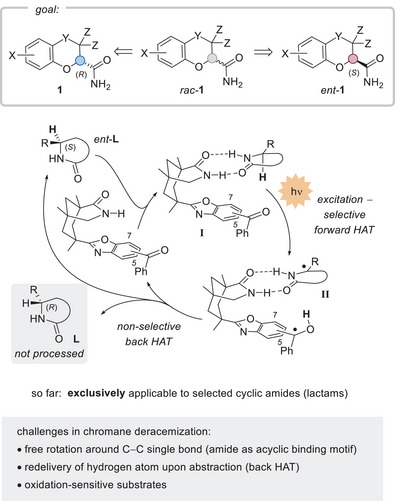
Access to either chromane enantiomer **1** or *ent*‐**1** starting from the racemic mixture *rac*‐**1a** by a deracemization approach (top). Photochemical deracemization strategy via reversible hydrogen atom transfer (middle), and challenges in chromane deracemization (bottom).

In prior studies, our group has achieved the photochemical deracemization of chiral lactams (e.g., *rac*‐**L**) by the reversible removal of a hydrogen atom at the stereogenic carbon atom.^[^ ^30^, ^31^ ^]^ The chiral photocatalyst^[^ ^32^, ^33^, ^34^ ^]^ recognizes one of the two substrate enantiomers as shown in Scheme 1. Here, enantiomer *ent*‐**L** is processed and the photoactive part of the catalyst, a phenyl ketone,^[^ ^35^, ^36^ ^]^ abstracts, upon excitation within complex **I**, a hydrogen atom at the (*S*)‐stereogenic center. An achiral radical intermediate **II** is formed at which the C─H bond is re‐created unselectively. The latter process can occur, for example, via an enol intermediate (vide infra).^[^ ^31^ ^]^ Since enantiomer **L** is not processed by the catalyst, it prevails in the photostationary state as – in an ideal scenario – the only enantiomer (>98% *ee*). Since previous work had exclusively focused on cyclic amides (lactams)^[^ ^30^ ^]^ and primary amides had not yet been used, there were significant challenges associated with chromane deracemization (Scheme 1), particularly related to the rotation around the C─C bond between the carbon atom, from which C─H bond abstraction was to occur, and the amide carbonyl carbon atom.

Preliminary deracemization experiments on parent chromane‐2‐carboxamide (*rac*‐**1a**) started with known chiral benzophenones that display an azabicyclo[3.3.1]nonan‐2‐one backbone as recognition motif.^[^ ^35^, ^36^ ^]^ Catalyst **2** with the benzoyl group in 5‐position of the benzoxazole linker showed promising results, and optimization of the conditions (see the Supplementary Information for details) gave the desired product **1a** in good enantioselectivity (85% *ee*). The absolute configuration was assigned by HPLC comparison with authentic enantiomers of known configuration. To further improve the selectivity, compounds were added that could act as HAT catalysts^[^ ^37^, ^38^ ^]^ facilitating the back HAT (bHAT) and, thus, increasing the number of turnovers to be achieved by the photocatalyst. Their performance was assessed by determining the yield and *ee* of product **1a**. Among all additives, thiophenol (PhSH) performed best and was found to be ideally used in catalytic amounts (20 mol%). Under optimized conditions, product **1a** was obtained in 90% yield and with 94% *ee*. On a 0.5 mmol scale, the yield was 87% (94% *ee*) underpinning the scalability of the method (Scheme 2).

**Scheme 2:**
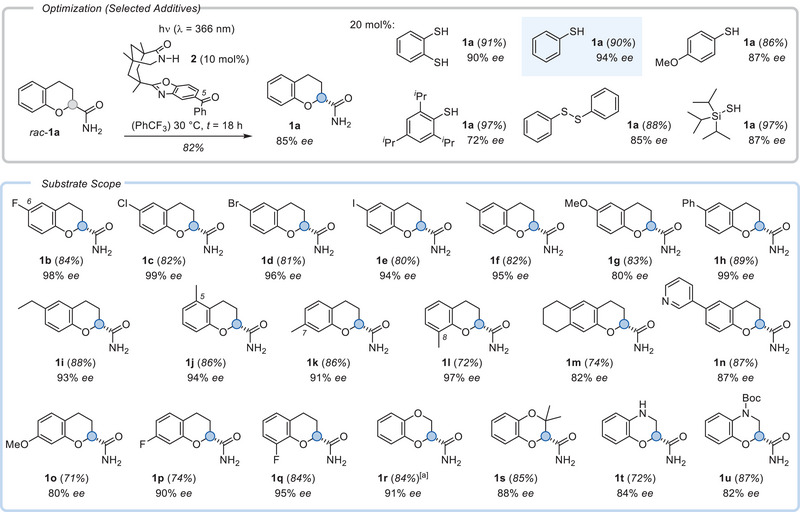
Optimization and scope for the photochemical deracemization of chromane‐2‐carboxamides *rac*‐**1** by chiral photocatalyst **2**. ^[a]^The reaction was run on a 0.5 mmol scale with 40 mol% thiophenol as additive (*t* = 20 h).

The deracemization was applicable to a broad range of substrates with little limitations regarding the substitution pattern and possible functional groups. Substituents placed in position C6 of the chromane backbone included fluoro (**1b**), chloro (**1c**), bromo (**1d**), iodo (**1e**), alkyl (**1f**, **1i**), methoxy (**1** **g**), phenyl (**1** **h**), and pyridyl groups (**1n**). The methyl group was moved around the benzo group of the chromane without any indication that placing the substituent at carbon atoms C5 (**1j**), C7 (**1k**), or C8 (**1l**) would lead to a significant loss in enantioselectivity. The same applies to the fluoro substituent, which was also probed at carbon atoms C7 (**1p**) and C8 (**1q**). Only electron‐rich substrates such as the 5,6‐dialkylsubstituted chromane **1m** and the methoxy‐substituted chromanes **1** **g** and **1o** showed a decline in the *ee*, which we tentatively attribute to their more facile oxidation, possibly compromising the stability of the catalyst. Catalyst decomposition is known to stall the progress of the deracemization at lower *ee* levels.^[^ ^30^ ^]^ Remarkably, replacing the methylene group at C4 with an oxygen atom was tolerated and product **1r,** which is a key component for the Doxazosin synthesis, was isolated in 84% yield and with 91% *ee*. In this instance, a scale‐up of the reaction with an increased loading of the additive (40 mol%) led to a better result than the reaction at small scale (25 µmol), which had given the product in only 87% *ee* (81% yield). Methyl substitution at C3 was compatible with the deracemization (**1s**), and even the electron‐rich 4‐azaderivatives **1t** and **1u** (Boc = *tert*‐butoxycarbonyl) of chromanes gave promising results.

The chromane‐2‐carboxamides represent ideal starting materials for the synthesis of the enantiomerically pure APIs mentioned in the introduction (Scheme 3). By using catalyst **2**, the (*R*)‐configured products became available and were directly employed in follow‐up reactions. The parent compound **1a** was reduced to amine **3**, which was alkylated with bromide **4** to deliver Repinotan in 82% yield. Since the racemate of the compound was not separable by chiral HPLC, the specific rotation [α]_D_ of the compound^[^ ^27^ ^]^ was taken as a measure of its enantiomeric purity. Sarizotan was available in 93% yield from amine **3** by reductive amination with aldehyde **5**. Here, the enantiomeric purity could be determined by chiral HPLC as 99% *ee*. The synthesis of Doxazosin commenced with amide **1r** which was converted via its acid into the amide **7** of secondary amine **6**. After removal of the nitrogen protecting group, the nucleophilic substitution at the 2‐position of 2‐chloroquinazoline **8** was achieved thermally (Δ*T*) and delivered the desired product as its hydrochloride. The enantiomeric purity was high as indicated by its specific rotation.^[^ ^39^ ^]^

**Scheme 3:**
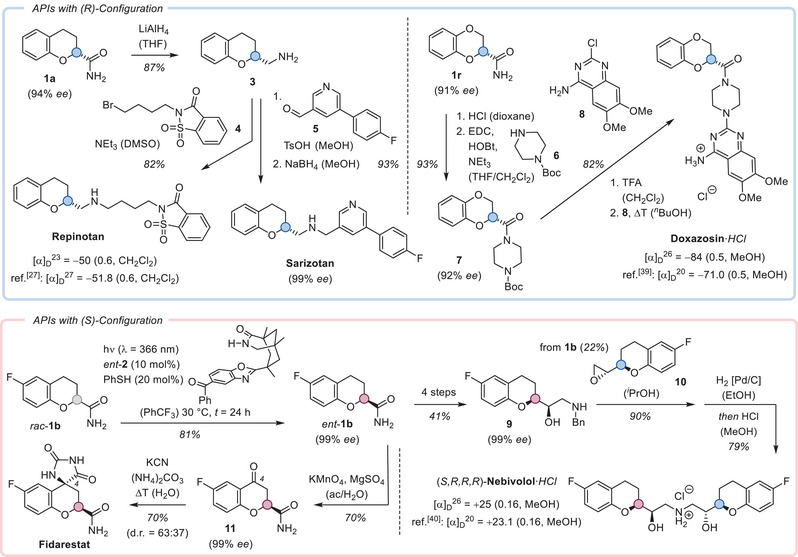
Synthesis of active pharmaceutical ingredients (APIs) in enantiomerically pure form. Abbreviations: ac = acetone; Bn = benzyl; EDC = 1‐ethyl‐3‐(3‐dimethylaminopropyl)carbodiimide; HOBt = hydroxybenzotriazole; TFA = trifluoroacetic acid; Ts = 4‐methylbenzenesulfonyl.

The deracemization approach offers the advantage that either one of the two enantiomers is available from the racemate in high yields. For the synthesis of (*S*)‐configured chromanes, the enantiomeric catalyst *ent*‐**2** was applied and delivered after recrystallization 6‐fluorochromane *ent*‐**1b** in enantiomerically pure form (99% *ee*) and in high yield (81%). The compound was converted in four steps into aminoalcohol **9**, which was competent to attack epoxide **10** in a nucleophilic ring opening reaction. Since the right‐hand part of Nebivolol required an (*R*)‐configuration at the chromane, epoxide **10** was prepared from enantiomer **1b** in three steps. After deprotection of the nitrogen atom, the potent (*S*,*R*,*R*,*R*)‐enantiomer^[^ ^18^ ^]^ of Nebivolol was obtained in enantiomerically pure form.^[^ ^40^ ^]^ The synthesis illustrates the power of photochemical deracemization with a single precursor, i.e., racemate *rac*‐**1b**, serving as substrate for both chromane enantiomers. Fluorochromane *ent*‐**1b** was also used as the precursor of Fidarestat. Here, an oxygenation at carbon atom C4 was performed which did not compromise the enantiopurity of intermediate ketone **11**. The latter compound served as the carbonyl component in the Bucherer‐Bergs reaction^[^ ^41^ ^]^ that created the desired hydantoin (d.r. = diastereomeric ratio).

In a first set of mechanistic experiments (Scheme 4), we interrogated the influence of the chromane oxygen atom on the enantioselectivity. Under otherwise unchanged conditions, the carbocyclic analogue of *rac*‐**1a**, compound *rac*‐**12**, resulted in a very limited enantioselectivity as did the sulfur analogue *rac*‐**13**. The kinetic isotope effect was experimentally probed by subjecting the (*S*)‐enantiomers of carboxamide **1a** as undeuterated version *ent*‐**1a** and as deuterated version *ent*‐**1a**‐*d*_1_ to the standard deracemization conditions employing catalyst **2**. The relative rate was determined by their conversion into the respective (*R*)‐enantiomer, i.e., from the decrease in enantiomeric excess. From separate measurements^[^ ^42^ ^]^ done in triplicate we obtained a primary kinetic isotope effect of k_H_/k_D_ = 2.3 (± 0.1).

**Scheme 4:**
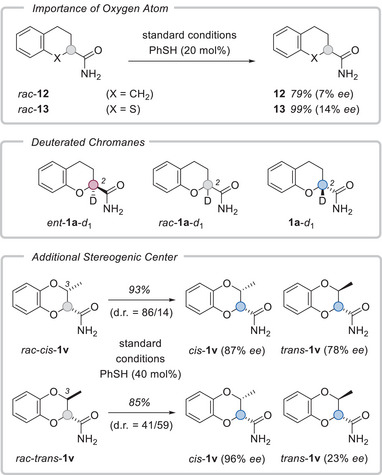
Compounds lacking the chromane oxygen atom fail to deliver meaningful enantioselectivities under the optimized irradiation conditions (top). Substrates and products employed in the determination of the kinetic isotope effect and in deuterium labeling studies (middle). If an additional stereogenic center is present at carbon atom C3 of the substrate, there is a match/mismatch situation preferring the formation of *cis*‐**1v** (bottom, for details see the narrative and the Supporting Information).

The influence of the thiophenol additive was studied by deuterium labeling experiments. It was found that the use of deuterated thiophenol (>90% deuterium) led to deuterium incorporation into the product (**1a**‐*d*_1_). Likewise, if deuterated substrate *rac*‐**1a**‐*d*_1_ was subjected to the standard reaction conditions, a hydrogen incorporation was observed (see the Supporting Information for details). Given that only 50% of the racemate, i.e., only the (*S*)‐enantiomer is processed, the degree of incorporation is in both cases roughly 25%–30%, indicating that the additive is involved in bHAT. From the *ee* values obtained in the labeling studies (43%–76% *ee*), it is evident that the catalyst is less efficient in processing deuterated substrates. Similar observations have recently been made when a chiral thiol was employed in a photochemical deracemization reaction.^[^ ^43^ ^]^ The primary amide moiety at the chromane is critical for binding to the catalyst. If the amide lacks hydrogen atoms that can bind to the lactam carbonyl oxygen atom of the catalyst, no deracemization was observed. Specifically, the racemic tertiary amide *rac‐* **7** was subjected to the optimized reaction conditions showing no indication for a deracemization (<1% *ee*).

If a substrate *rac*‐**1v** with an additional stereogenic center within the chromane ring was subjected to the reaction conditions, the *ee* of product *cis*‐**1v** was high (87% *ee* and 96% *ee*), irrespective whether the racemic starting material was *cis*‐ or *trans*‐configured. However, the results reveal that the enantiomer *ent*‐*cis*‐**1v** is more readily converted to *trans*‐**1v** than *ent*‐*trans*‐**1v** to product *cis*‐**1v**, leading to a lower *ee* for the *trans*‐diastereoisomer. In addition, the prevalence of *cis*‐**1v** suggests that it is not only formed by deracemization at C2 but also from *trans*‐**1v** in an editing process at C3 (for more details, see the Supporting Information).

To further elucidate the deracemization mechanism, quantum‐chemical calculations were conducted. Here, we focused on rationalizing (a) the beneficial effect of the neighboring oxygen atom at C2, (b) the mode of action of the catalyst, and (c) the role of the thiophenol additive (Scheme 5). Association free energies for complexes *ent*‐**1a⋅2** and **1a⋅2** revealed an exergonic association in both cases with −11.6 and −8.4 kJ⋅mol^−1^ (Table S2), indicating that both complexes form in solution. This association involves two‐point hydrogen bonding between substrate and catalyst. Conformer sampling^[^ ^44^ ^]^ further disclosed a significant stabilization via the intramolecular N─H⋯O motif of 18.7 kJ⋅mol^−^^1^ in free substrate **1a** or *ent*‐**1a** (Scheme 5, bottom left).

**Scheme 5:**
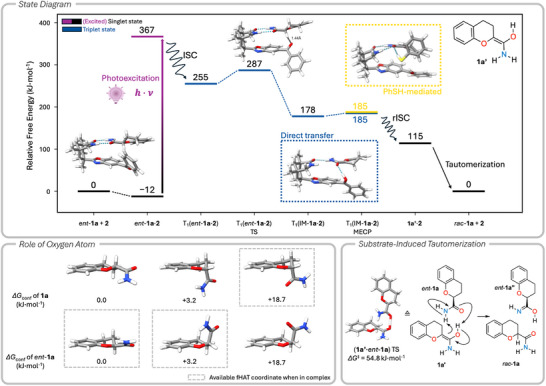
Computed free energy profile for the photocatalytical deracemization of chromane *rac*‐**1a** by processing of enantiomer *ent*‐**1a** (top). Free energy differences of selected **1a** or *ent*‐**1a** conformers (bottom left). Gray frames indicate conformers that – upon complex formation with **2** – expose the hydrogen atom at carbon C2 to the benzophenone moiety of **2**, thus, enabling fHAT. Tautomerization of product **1a’** with a second *ent*‐**1a** as final step of deracemization (bottom right). Geometry optimizations were performed with PBEh‐3c^[^ ^45^ ^]^ + CPCM^[^ ^46^ ^]^ (CH_2_Cl_2_). Free energy single points were computed with PW6B95‐D4/def2‐QZVPP//PBEh‐3c level^[^ ^47^, ^48^, ^49^ ^]^ of theory in the ORCA suite^[^ ^50^, ^51^ ^]^ while solvation contributions were computed with GFN2‐xTB/ALPB (CH_2_Cl_2_).^[^ ^52^, ^53^ ^]^ Relative energies are referenced to the most stable dimeric species.

The stabilization through the N─H⋯O motif in **1a** and *ent*‐**1a** persists even in the complex with catalyst **2** (Scheme 5, top, and Figure S5), preventing a reorientation of the C─H bond at the stereogenic center toward the benzophenone oxygen atom in complex **1a⋅2**, while favoring it in *ent*‐**1a⋅2**. Thus, a suitable reaction coordinate for forward HAT (fHAT) from the stereogenic carbon atom C2 to the benzophenone oxygen atom is only found in the thermodynamically accessible conformations of the latter. The clear difference in conformational free energy due to the intramolecular hydrogen bonds aligns well with the experimentally observed enantioselectivity for the reaction with *rac*‐**1a**. In contrast, the absence of the N─H⋯O motif in the carbocyclic analogue *rac*‐**12** leads to a noticeably reduced conformational free energy difference of < 2.2 kJ⋅mol^−1^ between fHAT active and inactive conformers. Hence, both enantiomers display an accessible fHAT coordinate (Figure S6), which is in line with the reduced enantioselectivity in the photocatalytic deracemization (Scheme 4).

In terms of the photocatalytic mechanism, our computations reveal that photoexcitation to the S_1_ state of catalyst **2** is the only feasible pathway to facilitate population of electronically excited states (Figure S7). In analogy to previous work,^[^ ^35^ ^]^ we expect intersystem crossing (ISC) to produce a long‐lived triplet T_1_ state on the benzophenone (see spin density analysis in Figure S8). From there, we find a feasible fHAT pathway, with a barrier of 31.3 kJ⋅mol^−1^ (Figure S9), in which the hydrogen atom at the stereogenic C2 carbon atom of the substrate is transferred to the benzophenone oxygen atom. The resulting species is a diradical (triplet) species. From here, the ground state is reached via a minimum energy crossing point (MECP) between T_1_ and S_0_. Unbiased MECP sampling^[^ ^54^ ^]^ reveals that the bHAT can proceed via different conformations from the benzophenone oxygen atom to the amide oxygen atom of the substrate (Figure S10). This MECP‐mediated bHAT seems energetically feasible (barrier of 7 kJ⋅mol^−1^). When considering the additive PhSH, an alternative bHAT pathway is found with a comparable free energy barrier (yellow in Scheme 5 top). Despite the entropically disfavored formation of a trimeric complex, this pathway remains feasible, as there is less disruption of the two‐point hydrogen bonding between the substrate and catalyst **2**. In this case, hydrogen atom transfer to the amide oxygen atom occurs from the thiophenol, which in turn receives the hydrogen atom from the benzophenone. The pathway, thus, explains the observations made in the deuterium labeling experiments. In either pathway, we find the formation of the enol **1a’**. To arrive at the chromane‐2‐carboxamide, thermal tautomerization of **1a’** back to either **1a** or *ent*‐**1a** is necessary. According to our calculations, a unimolecular tautomerization is not feasible (barrier of 121.3 kJ⋅mol^−1^, see Figure S11). Instead, this step is expected to proceed through a bimolecular pathway involving a second substrate molecule, i.e., *ent*‐**1a** or **1a** (see Figures S11 and S12). For the tautomerization step, a barrier of 54.8 kJ⋅mol^−1^ is found (Scheme 5 bottom right). In line with previous data on the dissociation rate constants from azabicyclo[3.3.1]nonan‐2‐one scaffolds,^[^ ^55^ ^]^ it is presently assumed that tautomerization occurs after dissociation of enol **1a’** from the catalyst. However, there is a chance that the enol blocks the catalyst and, thus, retards turnover. In this case, its tautomerization could potentially be the turnover‐limiting step.

In summary, our study has been conceptualized to display the potential of photochemical deracemization reactions for the preparation of enantiopure active pharmaceutical ingredients. Reversible HAT has been demonstrated as a useful tool that can be extended to substrates with an acyclic binding site. It is envisioned that further developments in the architecture of chiral photocatalysts will pave the way for a general approach towards editing stereogenic centers at will.

## Supporting Information

Primary research data are openly available in the repository RADAR4Chem at DOI: 10.22000/g3b6rp2qk6m4ndf8. https://radar4chem.radar‐service.eu/radar/en/dataset/g3b6rp2qk6m4ndf8?token=ICgqAKRpnLvMaAKYKyEV.

The authors have cited additional references within the Supporting Information.^[^ ^56^, ^57^, ^58^, ^59^, ^60^, ^61^, ^62^, ^63^, ^64^, ^65^, ^66^, ^67^, ^68^, ^69^, ^70^, ^71^, ^72^, ^73^, ^74^, ^75^, ^76^, ^77^, ^78^, ^79^, ^80^, ^81^, ^82^, ^83^, ^84^, ^85^, ^86^, ^87^, ^88^, ^89^, ^90^, ^91^, ^92^, ^93^, ^94^, ^95^, ^96^, ^97^, ^98^, ^99^, ^100^, ^101^, ^102^, ^103^, ^104^, ^105^, ^106^ ^]^

## Conflict of Interests

The authors declare no conflict of interest.

## Supporting information



Supporting information

## Data Availability

The data that supports the findings of this study are available in the supplementary material of this article. Primary research data are openly available in the repository RADAR4Chem at DOI: 10.22000/g3b6rp2qk6m4ndf8.
