# Supplementary material for: Photochemical Deracemization of Chromanes and its Application to the Synthesis of Enantiopure Bioactive Compounds
Source: Angew Chem Int Ed Engl. 2025 Dec 23;65(5):e21436. doi: 10.1002/anie.202521436 (PMC12851019; doi:10.1002/anie.202521436)
Supplement: Supplementary file 1 — Supporting information [file ANIE-65-e21436-s001.pdf]

Supporting Information

Photochemical Deracemization of Chromanes and its Application to the Synthesis of Enantiopure Bioactive Compounds

Biki Ghosh,[a],‡ Maximilian Iglhaut,[a],‡ Daria Babushkina,[b] Mike Pauls,[b] Christoph Bannwarth,*[b] and Thorsten Bach*[a]

[a]Technische Universität München, School of Natural Sciences, Department of Chemistry and Catalysis Research Center; 85747 Garching, Germany

[b]RWTH Aachen University, Institut für Physikalische Chemie; 52074 Aachen, Germany

‡These authors contributed equally to this work.

Corresponding author: Email: thorsten.bach@ch.tum.de

Table of Contents

S1. General Information 3
S2. Analytical Methods 6
S3. General Synthetic Procedures 8
S4. Computational Studies 14
S5. Optimization of the Reaction Conditions and Profile of the Deracemization Reaction 35
S6. Determination of the Absolute Configuration of Deracemization Product 1a 38
S7. Determination of the Kinetic Isotope Effect (KIE) 40
S8. H/D Exchange Experiments 45
S9. Synthesis and Characterization of Deracemization Substrates 52
S10. Photochemical Deracemization Reactions 100
S11. Stereochemical Editing of *rac-cis*-1v and *rac-trans*-1v 118
S12. Synthesis of APIs 122
S13. NMR Spectra 147
S14. HPLC Traces 245
S15. List of References 281

S1. General Information

All reactions sensitive to air or moisture, were carried out in flame-dried glassware under positive pressure of argon using standard Schlenk techniques.

Commercially available chemicals were used without further purification, if not further mentioned.

For moisture sensitive reactions, dichloromethane (CH_2Cl_2), diethyl ether (Et_2O) and tetrahydrofuran (THF) were purified using a MBSPS 800 *MBraun* solvent purification system. The following columns were used:

CH_2Cl_2: 2 × MB-KOL-A type (aluminum oxide)

Et_2O: 1 × MB-KOL-A type 2 (aluminum oxide), 1 × MB-KOL-M type 2 (3 Å molecular sieve)

THF: 2 × MB-KOL-M type 2 (3 Å molecular sieve)

Anhydrous *α,α,α*-trifluorotoluene ($PhCF_3$) and a 3 M solution of HCl in methanol (MeOH) were purchased from *Sigma Aldrich (Merck).* $PhCF_3$ was additionally stored over 3 Å molecular sieves.

Anhydrous acetonitrile (MeCN), dimethyl sulfoxide (DMSO), MeOH and ethanol (EtOH) were purchased from *Thermo Fisher Scientific* and stored over 3 Å molecular sieves.

Technical solvents for column chromatography [dichloromethane (CH_2Cl_2), ethyl acetate (EtOAc), methanol (MeOH), hexanes, *n*-pentane (pentane)] were used after simple distillation.

Normal-phase flash column chromatography (FCC) was performed on silica 60 (*Merck*, 230-400 mesh) with the indicated eluent mixture.

Commercially available starting materials were purchased either from *Sigma Aldrich (Merck)*, *TCI Chemicals*, *ABCR* or *BLDpharm*.

Unless water was used, round bottom flasks, vials and phototubes were dried with a heat gun at approximately 600 °C under vacuum.

Unless otherwise stated, photochemical reactions at $\lambda = 366$ nm were carried out in Duran phototubes (∅ = 1 cm, 10 mL) under argon atmosphere in a positive geometry setup with a cylindrical array of 16 fluorescent light tubes, UV-A, $\lambda_{max} = 366$ nm (Fig. S1, S2).

Prior to the start of a photoreaction, each reaction mixture was degassed by being sparged with argon under ultrasonication for 15 min.

Lehrstuhl OC 1 - TUM

200 nm | 250 nm | 300 nm | 350 nm | 400 nm | 450 nm | 500 nm | 550 nm | 600 nm | 650 nm

Datasheet FLT024 Philipps-BLB-365

Basic Information

Type	Fluorescent light tube	
Description	Philipps TL 8W BLB	
Manufacturer / Supplier	Philipps / Beleuchtungdirekt.de	
Order number / Date of purch.	n/a / 02/2021	
Internal lot / serial number	2021-02 / FLT024	

Specification Manufacturer

Type / size	T5 tube, G5 socket	
Mechanical specification	16 mm diameter, 288 mm length	
Electrical specification	8 W	
Wavelength (range, typ.)	350 - 400 nm	
Spectral width (FWHM)	~ 16 nm	
Datasheet	n/a	

Characterization

Description of measurement	Measured with Ocean-optics USB4000 spectrometer using a calibrated setup (cosine corrector/fibre). The cosine corrector was placed at 20 mm distance from a single fluorescent tube at half height.	
Measured dominant wavelength / Int.	365 nm	168 µW/mm²nm
Measured spectral width (FWHM)	16 nm	
Integral Reference intensity / range	3059 µW/cm²	300-450 nm

Spectrum

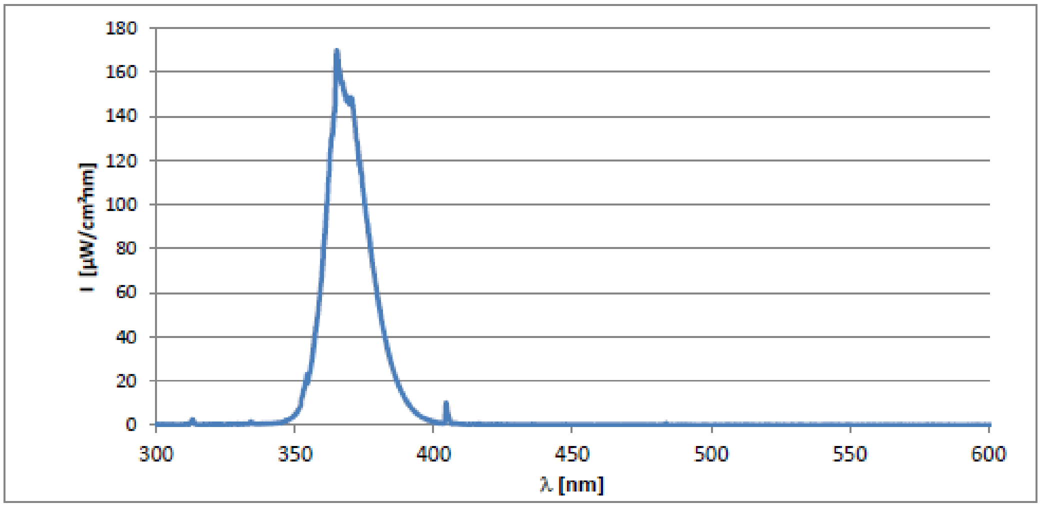

Fig. S1: Emission spectrum of the fluorescent light tube.

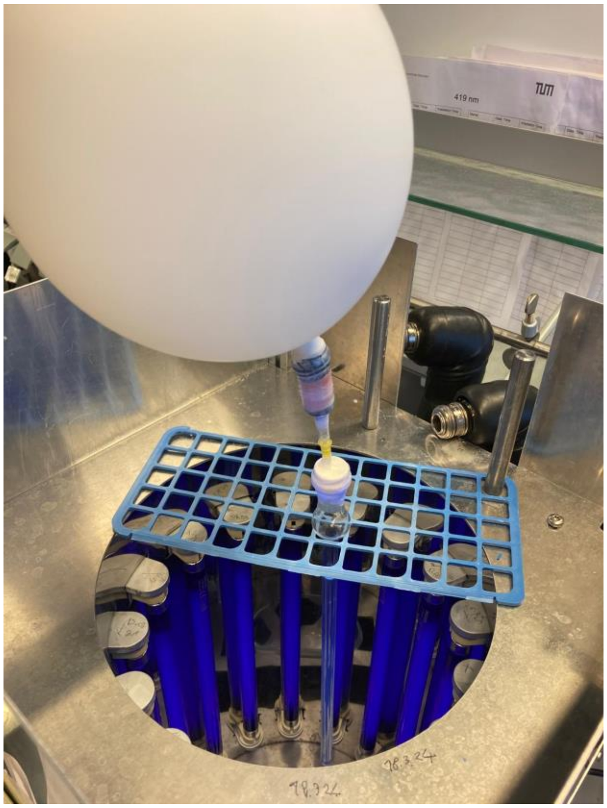

Fig. S2: Typical setup for a photochemical deracemization reaction.

S2. Analytical Methods

Thin layer chromatography (TLC) was performed on silica coated glass plates (silica gel 60 F_{254}) with detection by UV-light (λ = 254 nm) and potassium permanganate stain [$KMnO_4$].

Infrared spectra (IR) were recorded on a JASCO IR-4100 or a *Perkin Elmer* Frontier IR-FTR spectrometer by ATR technique. The signal intensity is assigned using the following abbreviations: s (strong), m (medium), w (weak). The following abbrevations were used: aliph = aliphatic, arom = aromatic.

$KMnO_4$-staining solution: Potassium permanganate (3.00 g), potassium carbonate (20.0 g), aqueous NaOH solution (5%, 5.0 mL), water (300 mL).

2,4-Dinitrophenylhydrazine (DNP)-staining solution: 2,4-Dinitrophenylhydrazine (12.0 g), H_2SO_4 (98%, 80 mL), aqueous NaOH solution (5%, 5.00 mL), EtOH (200 mL).

Melting points (M.p.) were determined using a Kofler ("Thermopan", Fs *Reichert*, Wien) apparatus.

Nuclear magnetic resonance (NMR) (^{1}H, ^{13}C and ^{19}F-NMR) spectra were recorded at room temperature (r.t.) on either a *Bruker* AVHD-400, AVHD-500, or a *Bruker* AV-II-500 equipped with cryo probe head. Chemical shifts of the NMR spectra are reported relative to $CHCl_3$ (^{1}H-NMR: δ = 7.26 ppm, ^{13}C-NMR: δ = 77.16 ppm), MeOH (^{1}H-NMR: δ = 3.31 ppm, ^{13}C-NMR: δ = 49.00 ppm) or DMSO (^{1}H-NMR: δ = 2.50 ppm, ^{13}C-NMR: δ = 128.06 ppm). The data are reported as follows: chemical shift (δ) [multiplicity, coupling constant *J* (Hz), relative integral, number of protons] where multiplicity is defined as: m = multiplet, s = singlet, d = doublet, t = triplet, q = quartet, br broad. Apparent multiplets which occur as a result of coupling constant equality between magnetically non-equivalent protons are marked as virtual (*virt.*).

Mass spectrometry (MS) and **high-resolution mass spectrometry (HRMS)** were measured on a *Thermo Scientific* LTQ-FT Ultra (ESI).

Specific Rotation was determined using an ADP440+ polarimeter (Fa *Bellingham+Stanley*) and is reported as follows: $[\alpha]_D^T$ (c in g per 100 mL solvent). The polarimeter has a variance of ± 0.001 which translates to a variance of ± 2 of the measured rotation for c = 1.0 and a cuvette path length of 0.5 cm.

High Performance Liquid Chromatography (HPLC) was performed using a chiral stationary phase [ChiralPak AD-H (250 x 4.6 mm), Chiralpak IC (250 x 4.6 mm), ChiralPak AS-H (250 x 4.6 mm), ChiralPak IA (250 x 4.6 mm), ChiralPak AS-RH (150 x 4.6 mm), ChiralCel OD-

RH (250 x 4.6 mm), ChiralCel OD-H (250 x 4.6 mm), *Daicel Chemical Industries*] with UVD 340 Photodiode Array Detector, P580 Pump and an ASI-100 Automated Sample Injector at 20 ^{o}C. For normal-phase HPLC a *Daicel* ChiralPak AD-H, ChiralPak IC, ChiralPak AS-H, ChiralPak IA, and ChiralCel OD-H were used as the stationary phase with a mixture of *n*-heptane/*i*-propanol as the mobile phase or a *Daicel* ChiralPak AS-RH and ChiralCel OD-RH were used as the stationary phase with a mixture of MeCN/water as the mobile phase.

S3. General Synthetic Procedures

General Procedure A (GP A): Synthesis of 4-oxo-4*H*-chromene-2-carboxylic acid ethyl esters (S-1)

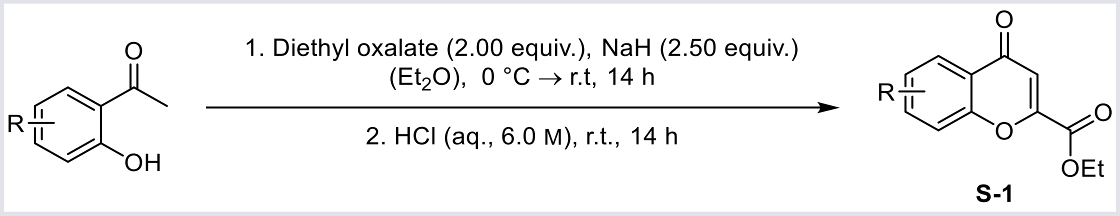

Following a modified procedure by Sabui *et al.,*[56] a mixture of the respective *ortho*-hydroxy acetophenone (1.00 equiv.) and diethyl oxalate (2.00 equiv.) was added dropwise to a suspension of NaH (60 wt%, 2.50 equiv.) in Et_2O (0.5 M) at 0 °C. Then, the resulting suspension was allowed to warm to r.t. and stirred for 14 h, before the reaction mixture was poured on an ice/water mixture. The pH value was adjusted to 1 by addition of aqueous HCl (6.0 M) and the resulting suspension was extracted thrice with Et_2O. The organic layers were combined and the solvents were removed under reduced pressure. The obtained solids were then suspended in aqueous HCl (6.0 M) and the resulting suspension was stirred at r.t. for 14 h. Water was added and the reaction mixture was extracted thrice with Et_2O. The combined organic layers were then washed with brine and dried over Na_2SO_4 before the remaining solvents were removed under reduced pressure. The obtained crude products were then purified by FCC (SiO_2, EtOAc/hexanes) to yield the 4-oxo-4*H*-chromene-2-carboxylic acid ethyl esters (**S-1**) as white solids.

General Procedure B (GP B): Synthesis of chromane-2-carboxylic acid ethyl esters (*rac*-S-2)

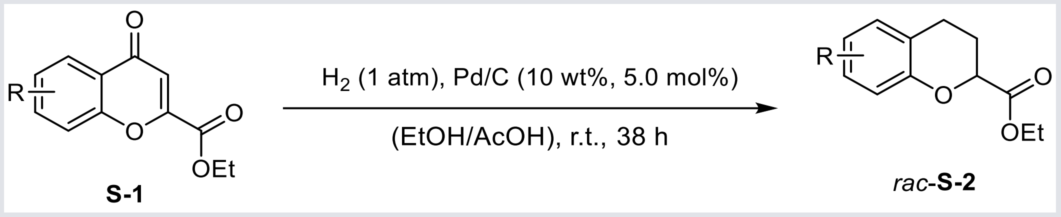

Following a modified procedure by Wakita *et al.*,[57] a suspension of a respective 4-oxo-4*H*-chromene-2-carboxylic acid ethyl ester (**S-1**) and palladium on activated charcoal (Pd/C) (10 wt%, 5.0 mol%) in EtOH (0.18 M) and conc. AcOH (18 M, 11.2 equiv.) was degassed by freeze-pump-thaw cycling (2 ×) and then sparged continuously with H_2 (1 atm) and stirred at r.t. for 38 h. After that, the solid compounds were filtered over celite and washed with copious amounts with EtOAc. The filtrate was then concentrated under reduced pressure and the obtained crude product was subjected to FCC (SiO_2, EtOAc/hexanes) to afford the chromane-2-carboxylic acid ethyl esters (*rac*-**S-2**) as colorless oils.

General Procedure C (GP C): Synthesis of racemic chromane-2-carboxamides (*rac*-1) from chromane-2-carboxylic acid ethyl esters (*rac*-S-2).

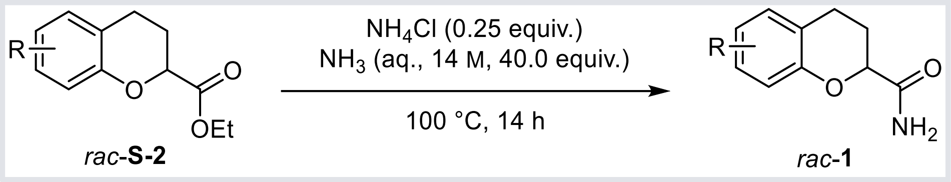

Following a modified procedure by Marco *et al.,*[58] a mixture of the respective chromane-2-carboxylic acid ethyl ester (*rac*-**S-2**), NH_4Cl (0.25 equiv.) and aqueous ammonia (14 M, 40.0 equiv.) was stirred at 100 °C for 5 h. After allowing the mixture to cool to r.t., water and CH_2Cl_2 were added and the aqueous and the organic layers were separated. Then, the aqueous layer was extracted thrice with CH_2Cl_2 and the combined organic layers were washed with brine and dried over Na_2SO_4. The remaining solvents were removed under reduced pressure and the crude products were subjected to FCC (SiO_2, EtAOc/hexanes) to yield the racemic chromane-2-carboxamides (***rac*-1**) as white solids.

General Procedure D (GP D): Synthesis of racemic chromane-2-carboxylic acids (*rac*-S-3) from chromane-2-carboxylic acid ethyl esters (*rac*-S-2).

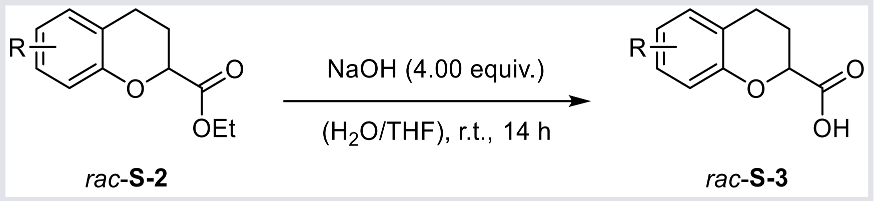

Following a modified procedure by Wakita *et al.*,[57] a solution of the respective chromane-2-carboxylic acid ethyl ester (*rac*-**S-2**) in THF (1.0 M) was added to an aqueous NaOH solution (1.0 M, 4.00 equiv.) at r.t. and the resulting mixture was stirred at r.t. for 14 h. More water was added and the solution was washed thrice with Et_2O before the pH value of the aqueous layer was adjusted to 1 by addition of aqueous HCl (6.0 M). The resulting suspension was extracted thrice with EtOAc and the combined organic layers were washed with brine and dried over Na_2SO_4 before the remaining solvents were removed under reduced pressure to yield the chromane-2-carboxylic acids (*rac*-**S-3**) as white solids.

General Procedure E (GP E): Synthesis of racemic chromane-2-carboxamides (*rac*-1) from chromane-2-carboxylic acids (*rac*-S-3)

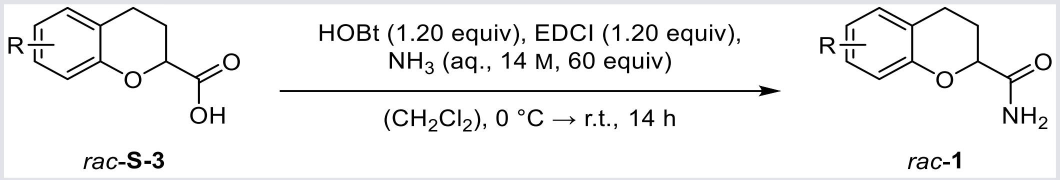

Following a procedure by Plaza *et al.,*[59] 1-Hydroxybenzotriazole hydrate (HOBt · H_2O) (1.20 equiv.) was added to a solution of the respective chromane-2-carboxylic acid (*rac*-**S-3**) in CH_2Cl_2 (0.03 M) at 0 °C. After 10 min, *N*-(3-dimethylaminopropyl)-*N'*-ethylcarbodiimide-hydrochloride (EDCI · HCl) was added and after stirring the resulting mixture at 0 °C for another 30 min, aq. ammonia (14 M, 60.0 equiv.) was added before the reaction mixture was allowed to warm to r.t. and stirred vigorously for 14 h. Then, water was added, the layers were separated and the aqueous layer was extracted thrice with CH_2Cl_2. The combined organic layers were washed with brine and dried over Na_2SO_4. The solvents were removed under reduced pressure and the crude product was subjected to flash column chromatography (FCC) (SiO_2, EtOAc/hexanes) to yield the racemic chromane-2-carboxyamides (*rac*-**1**) as white solids.

General Procedure F (GP F): Photochemical deracemizations of chromane-2-carboxamides (*rac*-1)

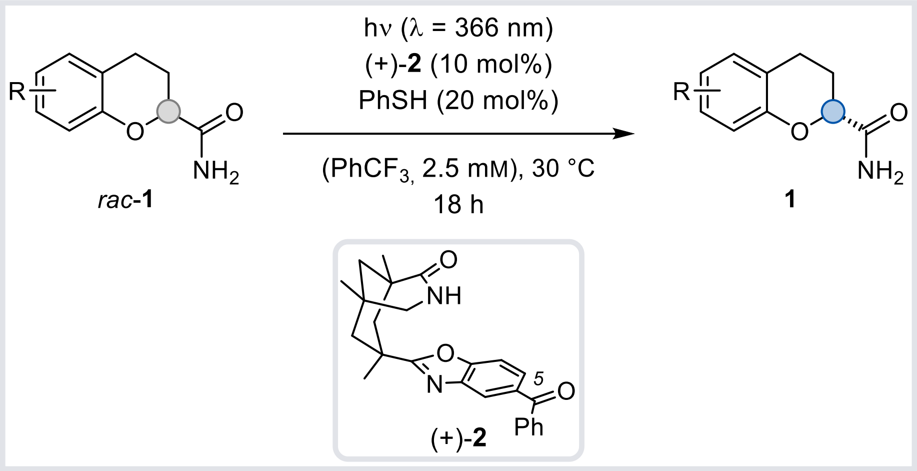

A dried phototube (Ø = 1 cm) was charged with the corresponding racemic chromane-2-carboxamide (*rac*-**1**) (25.0 µmol, c = 2.5 mM, 1.00 equiv.) and enantiomerically pure (+)-benzophenone **2** (10 mol%) under an argon atmosphere. In a separate vial, a stock solution of PhSH was pepared by dissolving PhSH (5.00 µL) in dry $PhCF_3$ (1.00 mL). From this stock solution, 103 µL (5.00 µmol, 20 mol%) was added followed by the addition of dry $PhCF_3$ (10 mL). The resulting solution was degassed by being sparged with argon under ultrasonication for 15 min and irradiated at λ = 366 nm at 30 °C for 18 h. After irradiation, the volatile compounds were removed under reduced pressure and the crude products were subjected to FCC (SiO_2, EtOAc/hexanes) to afford the enantioenriched (*R*)-chromane-2-carboxamides (**1**) as white solids.

S4. Computational Studies

Computational Details

We considered the enantiomeric chromane-2-carboxamide (*ent*-)**1a** and the chiral benzophenone catalyst **2** for our computational study (Fig. S**3**).

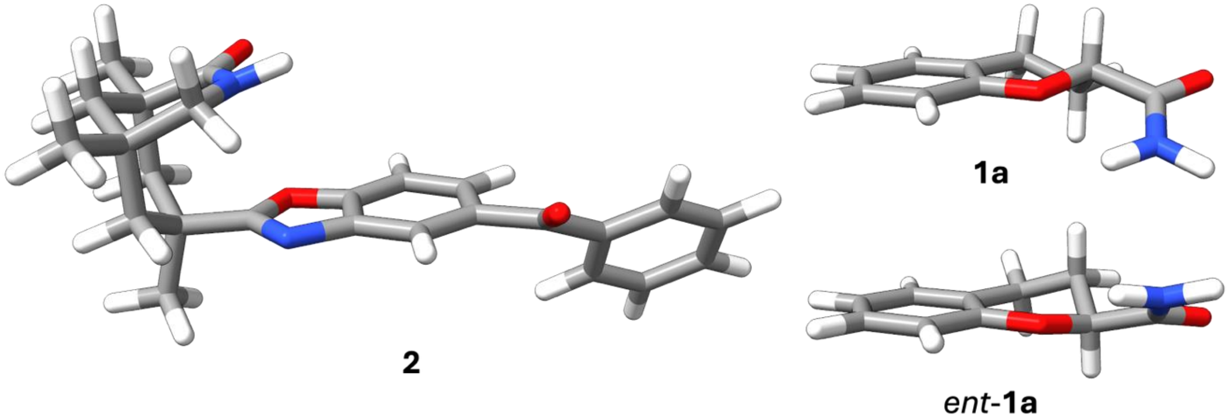

2 **1a** *ent*-**1a**

Fig. S3: Lowest energy conformers of the chromane-2-carboxamide enantiomers **1a** and *ent*-**1a** and the benzophenone catalyst **2** optimized using PBEh-3c + CPCM(DCM) level of theory.

All molecular geometries were first optimized with the semiempirical quantumchemical method (SQM) GFN2-xTB[52] as implemented in the xtb program (version 6.6.1).[60] To account for solvation effects, we used the analytically linearized Poisson–Boltzmann (ALPB) implicit solvation model.[53] Since the experimentally used solvent α,α,α-trifluorotoluene is not parametrized for the ALPB model, dichloromethane (DCM) was chosen as substitute due to its similar dielectric constant.

These optimized structures served as initial input for metadynamics-based conformational sampling, which is performed with the conformer rotamer ensemble sampling tool (CREST) (version 2.11.3),[44,61] applying default settings and GFN2-xTB as the electronic structure theory level. For open-shell species (D_0 or T_1), the number of unpaired electrons was adjusted accordingly (keyword “uhf” in CREST).

The generated conformers were used for the generation of noncovalent complexes according to the intermolecular force field xTB-iFF (version 1.1)[62] approach. The necessary localized molecular orbitals were calculated with GFN1-xTB[63] and ALPB(DCM). The associated structures were further optimized with GFN2-xTB+ALPB(DCM). After conformational sampling and the docking workflow, ensemble sorting and filtering was done with the CREGEN[61,64,65]

routine of the CREST program. Here, default settings and an energy cutoff of 3 kcal·mol^{-1} with respect to the energetically lowest conformer was used.

These geometries were further refined using the ORCA suite (version 6.0.1).[50,51,66] To describe the electronic ground state, restricted Kohn-Sham (RKS) density functional theory (DFT) calculations were performed with the PBEh-3c composite method[45] and the conductor-like polarizable continuum (CPCM)[46] implicit solvation model (ϵ = 9.18) as implemented in ORCA. PBEh-3c is a hybrid DFT method utilizing a modified def2-SVP[49] basis set (def2-mSVP) and a global hybrid functional based on the functional of Perdew, Burke and Ernzerhof (PBE)[67] with 42% of non-local Fock exchange. Dispersion interactions are accounted for using a Grimme dispersion correction (D3[68,69] for PBEh-3c), and a geometric counterpoise correction (gCP)[70] addresses the basis set superposition error.

T_1 and D_0 minima were computed with unrestricted Kohn-Sham (UKS) DFT using PBEh-3c and the CPCM for DCM as implemented in ORCA[71].

Computation of Free Energies

For the computation of Gibbs free energies of species i, G_i, geometries optimized at the PBEh-3c+CPCM(DCM) level of theory were used if not stated otherwise. Following equation 1, the calculated free energies contain three components, namely electronic energies E_{el}, a thermostaticstical correction, which accounts for translational, rotational, and vibrational (TRV) nuclear degrees of freedom and the zero-point vibrational energy, G_{TRV}, and a solvation free energy correction δG_{solv}.

$$\boldsymbol{G_i = E_{\mathrm{el},i} + G_{\mathrm{TRV},i} + \delta G_{\mathrm{solv},i}} \tag{1}$$

As a separate solvation correction is employed, the electronic energy is computed in gas phase. This single-point calculation was performed with PW6B95[47]-D4[48]/def2-QZVPP[49]//PBEh-3c+CPCM(DCM) level of theory. For this single-point energy calculation, the resolution-of-the-identity (RI-J) [72,73] approximation for the evaluation of the Coulomb integrals with the def2/J[74] auxiliary basis set and the chain of spheres for exchange (COSX)[75,76] were used.

TRV contributions to the free energy are described in the modified rigid rotor–harmonic oscillator (mRRHO)[77] formalism. Therein, harmonic frequencies below 50 cm^{-1} were handled by the interpolated free rotor-harmonic oscillator model. The solvation correction $\delta G_{\mathrm{solv},i}$ referred to the GFN2-xTB(+ALPB) level of theory, which accounts for the change in (free) energy between the

calculation in gas phase and was employing the implicit solvation model ALPB for the solvent DCM. Furthermore, a standard state correction for the conversion of 1 mol of an ideal gas at 1 bar to a 1 M solution was accounted for.

To avoid several computationally demanding harmonic frequency calculations at the DFT level, we instead presorted the identified conformers based on a free energy that takes into account a G_{TRV} value, which we computed from GFN2-xTB + ALPB(DCM) frequencies and geometries. After identifying the free energy minimum conformer from these free energies, we re-evaluated G_i with the G_{TRV} contribution obtained from harmonic frequencies at the PBEh-3c+CPCM(DCM) level of theory. Throughout this work, we based the reported free energy differences on this single representative conformer per molecular species.

Table S1 lists the theory levels and details for computing the free energy contributions of the considered species. For the computation of free energies of triplet and doublet species, we used UKS-DFT correspondingly.

Table S1: Overview of the theory levels used to determine Gibbs free energies (see equation 1) for each structure in this computational study. See the text for further details on these calculations.

Geometry optimizations	PBEh-3c + CPCM(DCM)
Electronic energies $E_{\mathrm{el},i}$	PW6B95-D4/def2-QZVPP
Nuclear contributions to the free energy $G_{\mathrm{TRV},i}$	PBEh-3c + CPCM(DCM) harmonic frequencies computed on the respective minima (scaled by 0.95[78]); particle-in-a-box, rigid rotor and modified harmonic oscillator model to account for translational, rotational and vibrational contributions to the nuclear free thermal energy. Harmonic frequencies below 50 cm^{-1} are handled by the interpolated free rotor–harmonic oscillator model.
Solvation free energy correction $\delta G_{\mathrm{solv},i}$	$\delta G_{\mathrm{solv},i} = G_{\mathrm{GFN2-xTB/ALPB(DCM)},i} - E_{\mathrm{GFN2-xTB/gas},i}$ based on PBEh-3c geometries. This term is fitted to include the standard state correction for transferring 1 mol of gas at 1 bar to a 1 M solution (keyword bar1M in xtb).

Free Energy Minima and Association

Conformer sampling, as outlined in in the previous section, was first performed for **1a** and *ent*-**1a**. The resulting conformer ensembles and associated free energies are presented in Scheme 5 in the main article and Fig. S4 below. The lowest-energy conformer of *ent*-**1a** and **1a** shows an orientation of the amide N-H group towards the chromane oxygen. This intramolecular hydrogen bonding interaction explains the calculated energetic difference of +18.7 kJ·mol^{-1} (Fig. S4) relative to conformers that do not exhibit this interaction. Stabilization via similar interaction was also reported in literature.[79,80]

Further confirmation of this trend is found from analyzing conformers of *rac*-**12**, which lacks the chromane oxygen atom. Here, differences in free energy are overall smaller with up to +7.3 kJ·mol^{-1} and no pronounced stabilization of a specific amide orientation is observed.

The reactive conformers of **12** and *ent*-**12** with ΔG_{conf} = 7.3 kJ·mol^{-1} and 1.9 kJ·mol^{-1}, respectively, are both significantly lower in energy, compared to the 18.7 kJ·mol^{-1} necessary for **1a** to adopt a comparable geometry.

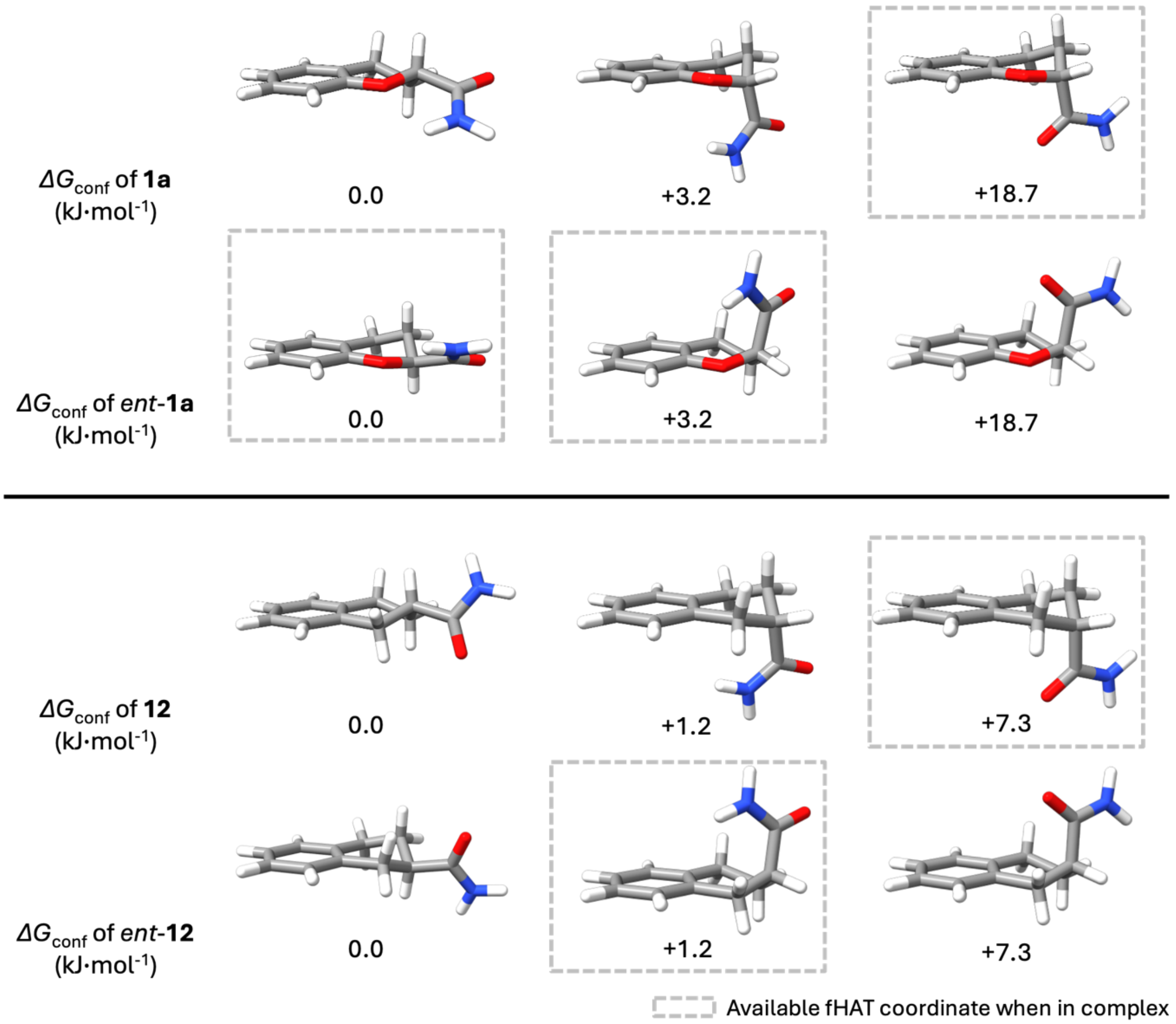

Fig. S4: Free energy differences of selected (*ent*-)**1a** and (*ent*-)**12** conformers. Gray frames mark conformers, where the hydrogen atom at the stereogenic carbon C2 points towards the benzophenone moiety of **2** in their complex, which enables fHAT. Energies were calculated according to Table S1 for structure *ent*-**1a**/**12** and for representation of **1a**/*ent*-**12** mirrored structures and energies of *ent*-**1a**/**12** were used.

The N-H···O binding motif of (*ent*-)**1a** also influences the complex formation with **2**. The resulting free energy minima of complexes *ent*-**1a**·**2** and **1a**·**2** are shown in Fig. S5.

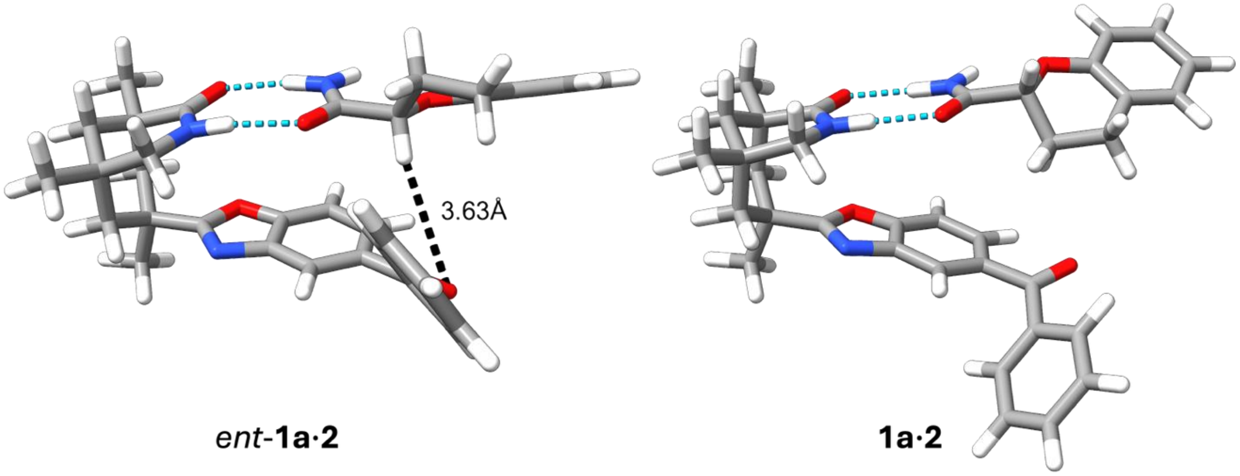

Fig. S5: Energetically lowest complex geometries of the enantiomers **1a** and *ent*-**1a** with catalyst **2** optimized using PBEh-3c+CPCM(DCM). The reaction coordinate for forward hydrogen atom transfer is drawn as a black dashed line and the distance amounts to 3.63 Å.

For both complex geometries, two-point hydrogen bonding between **2** and the substrate's amide unit is observed. The enantiomer *ent*-**1a** exhibits a favorable orientation of the hydrogen at the chiral carbon towards the benzophenone oxygen (3.63 Å) in terms of the photochemically-induced hydrogen abstraction. No such orientation is found among the identified conformers of **1a**·**2** within a 13 kJ·mol^{-1} energy window (equivalent to the cutoff of 3 kcal·mol^{-1} used in the CREGEN sorting algorithm of CREST to filter the conformer ensembles). Both *ent*-**1a** and **1a** retain the internal N-H···O motif while in complex. This stabilizing interaction likely "locks" the amide conformation, hence, preventing a re-orientation of the chiral C-H at 1a towards the benzophenone oxygen. This preferential orientation offers an explanation for the efficient retention of **1a** while *ent*-**1a** is processed.

For reasons of comparability, we proceeded analogously with the substrate **12** to identify relevant complex geometries. In Fig. S6, selected conformers of (*ent*-)**12**·**2** with suitable fHAT coordinate are displayed.

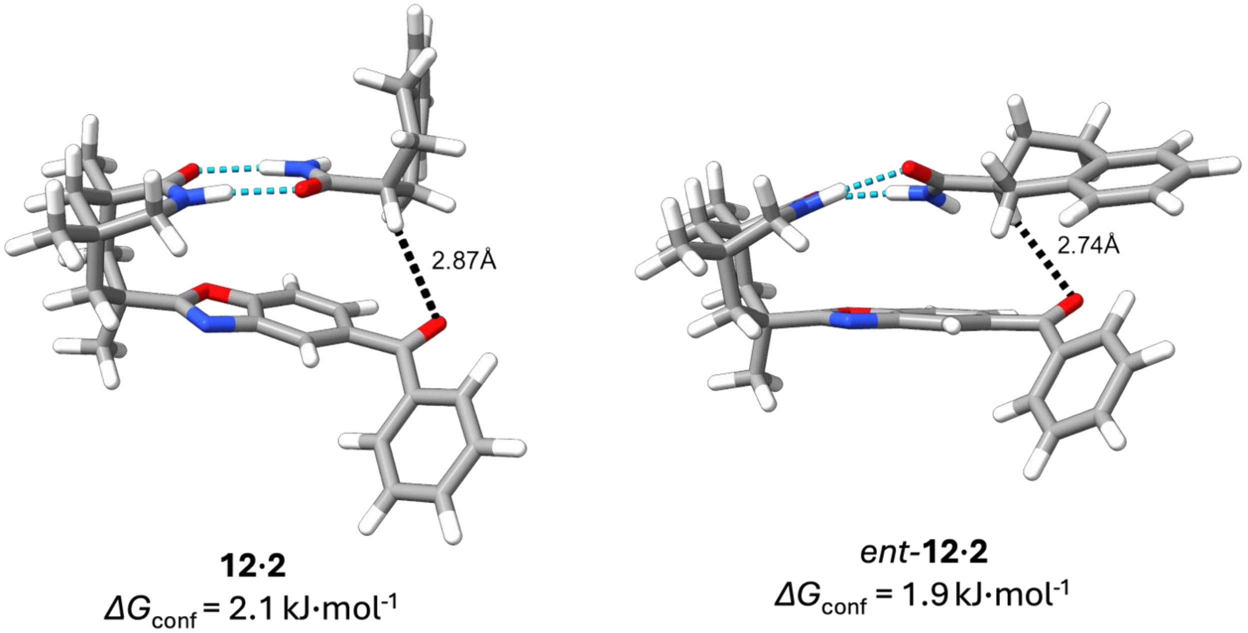

Fig. S6: Low-energy conformers of (*ent*-)**12**·**2** with accessible fHAT coordinates (H···O distance below 3.0 Å). Relative free energies are given relative to the respective minimum free energy conformer.

For both *ent*-**12**·**2** and **12**·**2** available fHAT coordinates exist within an energy range of just +2.1 kJ·mol^{-1} with respect to the identified free energy minimum conformer. This supports the conclusion that the chromane oxygen atom is a key structural element that influences the enantioselectivity in this deracemization. In its absence, as is shown for *rac*-**12**, other conformations that likely can undergo hydrogen abstraction via fHAT from the substrate to catalyst **2** become available for both enantiomers, thereby affecting the enantioselectivity of this reaction.

To further elucidate this, we determined the association free energies for complex formation and dimerization of the individual constituents. The latter is used to calculate effective association free energies, which account for possible dissociation of pre-existing dimers, if dimerization is exergonic. The computed values are listed in Table S2.

Table S2: Computed association free energies ΔG and effective association free energies ΔG_{eff} for complexes between chromanes (*ent*-)**1a** and (*ent*-)**12** to the catalyst **2**, as well as substrate dimerization energies. Calculations follow the methodology in Table S1.

Reaction	$\Delta G_{\mathrm{assoc}}$ (kJ·mol^{-1})	ΔG_{eff} (kJ·mol^{-1})
Catalyst Association		
**1a** + **2**	−12.0	−8.4
ent-**1a** + **2**	−15.1	−11.6
**12** + **2**	−12.5	−6.8
ent-**12** + **2**	−12.4	−6.7
**2**×**2**	−7.2	-
Substrate Association		
2×**1a**	7.7	-
ent-**1a** + **1a**	1.8	-
2×**12**	−2.5	-
ent-**12** + **12**	−4.8	-

One should note that the racemic mixture is used as starting point, hence, heterodimer formation of two enantiomers is possible for the substrates. Given that the formation of **2·2** and *ent*-**12·12** is exergonic, hence, both are used to derive effective association enthalpies.

Consequently, we derive exergonic complexation energies of **1a** and *ent*-**1a** with **2** of ΔG_{eff} = −8.4 kJ·mol^{-1}and −11.6 kJ·mol^{-1}, respectively. Considering the structures in Fig. S5 and Scheme 5 in the main article, the *ent*-**1a·2** complex likely exists in solution with a geometry suitable for the hydrogen atom transfer towards the benzophenone oxygen. In contrast, no such structure of **1a·2** was found within the low-energy conformer ensemble.

Similarly, the association energy amount to −6.7 kJ·mol^{-1} for *ent*-**12·2** and −6.8 kJ·mol^{-1} for **12·2**. Thus, considering also the reported geometries in Fig. S4, for *ent*-**12·2** reactive conformers are identified for both enantiomers when associated to **2** in the noncovalent complexes.

Forward Hydrogen Atom Transfer

In prior publications[31,35] the proposed mechanism for the forward hydrogen atom transfer (fHAT) involves excitation to an excited singlet state (S_1) followed by intersystem crossing (ISC) (and internal conversion) to the triplet state T_1.

Computation of Vertical Excitation Energies

To assess the energetics of the excited singlet and triplet states, we used the density functional theory multireference configuration interaction (DFT/MRCI)[81,82] method. In contrast to conventional Hartree-Fock-based MRCI, DFT/MRCI builds-up on a RKS-DFT reference configuration and corrects for double counting of electron correlation effects employing semiempirical corrections to the Hamiltonian matrix elements.[81] We employed the R2018 Hamiltonian[83] with the "short" parameterization and energy cutoff of 0.8 E_h to handle relevant configuration state functions (CSFs) as implemented in an OpenMP-parallelized[84] version of the DFT/MRCI code provided by the Marian group.[81] Throughout, the lowest five excited states per multiplicity were calculated and we employed a frozen core approximation, that is, molecular orbitals of the anchor configuration with energies outside the interval $-3.0\ E_h < \epsilon < +2.0\ E_h$ were kept frozen in the MRCI expansion.

The RKS-DFT anchor configuration referred to the BHLYP/def2-SVP[49,85] level of theory and was calculated using the ORCA interface. We made use of the RIJ COSX integral approximations as described above. The corresponding auxiliary basis set for RI-J (def2/J) and to construct electronic integrals for the subsequent MRCI calculation (def2-SVP/C)[86] were employed.

In Fig. S7, the calculated vertical excitation energies are presented.

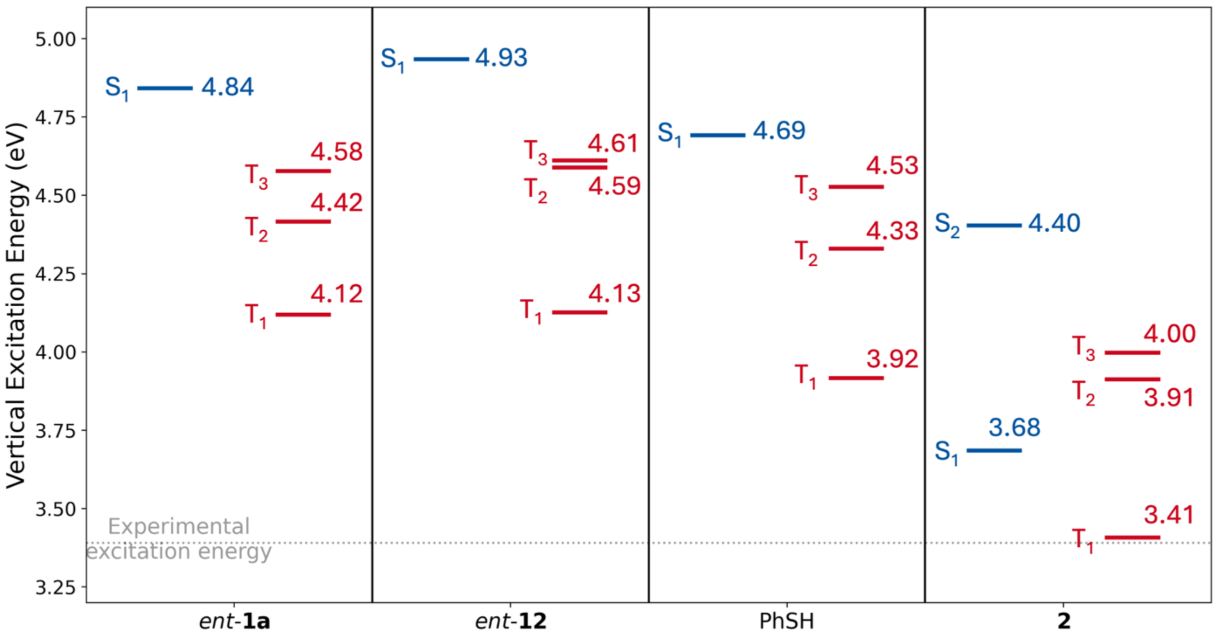

Fig. S7: Vertical excitation energies of *ent*-**1a**, *ent*-**12**, PhSH and **2** calculated using DFT/MRCI with the R2018 Hamiltonian, short parametrization and an energy cutoff of 0.8 Eh. Selected excited singlet (blue) and triplet states (red) are shown and the experimental irradiation wavelength of 366 nm (3.39 eV, dashed line, gray) is drawn for comparison.

Based on the calculated excitation energies, only photoexcitation to the excited S_1 state of **2** (3.68 eV) appears feasible at the experimental irradiation wavelength of 366 nm (3.39 eV). It is worth noting that we only choose one representative experimental wavelength for comparison to the calculated values, while experimental irradiation conditions actually span a broader energy range. Among other reasons, part of the deviation between the experimental and calculated vertical excitation energy can be rationalized this way.

Since the S_1 excitation energies of both substrates *ent*-**1a** (4.84 eV), *ent*-**12** (4.93 eV) and PhSH (4.69 eV) are noticeably higher in energy, direct photoexcitation of the substrates is unlikely. The T_1 state is energetically located below the S_1 state with 3.41 eV. We expect that this triplet state is populated via ISC and that the fHAT proceeds via a triplet mechanism. To further shed light on the nature of the T_1 excited state, which we deem relevant for the observed photochemical reaction, we calculated the T_1 spin density at the UKS-PBEh-3c+CPCM(DCM) level of theory in Fig. S8.

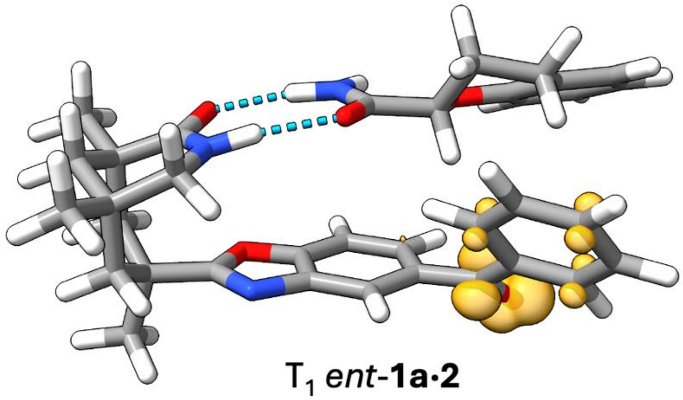

Fig. S8: T_1 spin density of *ent*-**1a**·**2** calculated at the free energy minimum geometry at the PBEh-3c+CPCM(DCM) level of theory. Isosurfaces are shown with a contour value of ± 0.025.

Inspecting the spin density of the T_1 state of complex *ent*-**1a**·**2**, which is almost exclusively located on the benzophenone (carbonyl) moiety of **2**, we find that this is a mostly fragment-localized state of $n\pi^*$ character. This observation aligns well with the above analysis of the excited states and the fact that photoexcitation of the catalyst **2** is preferred over direct excitation of the chiral substrate *ent*-**1a**.

Transition State Calculation

To identify plausible reaction pathways, the transition states (TS) were identified using a combination of SQM and DFT methods. Initially, a distance scan was performed along the fHAT reaction coordinate on the GFN2-xTB potential energy surface (PES). As a starting point for these scans, the conformer of *ent*-**1a**·**2** with the shortest distance between the benzophenone oxygen of **2** and the H atom attached to the stereocenter d_{OH} was used. At each point along the scan, a constrained geometry optimization was performed in ORCA by addressing xtb for the GFN2-xTB gradients, while fixing the d_{OH} distance. Additionally, a GFN2-xTB Hessian was calculated on the resulting geometry. After manually checking that the imaginary mode with largest absolute value features cartesian displacements that match the suspected fHAT process, the corresponding GFN2-xTB geometry and Hessian were used as input for a T_1 TS optimization at the UKS-PBEh-3c+CPCM(DCM) level in ORCA (keyword OptTS)[87] with a frequency calculation on the final geometry.

The resulting TS was verified assuring that only a single imaginary frequency is present at the corresponding geometry. Additionally, an intrinsic reaction coordinate (IRC) calculation,[88] which extrapolates a reaction path from the given TS, was performed to confirm that this TS connects the reactants prior and after fHAT. Both endpoints of the IRC path are then reoptimized (PBEh-3c+CPCM(DCM)) and compared to the expected reactant and product structures. If atomic connectivities match, the TS for the given reaction was considered further.

Transition States for fHAT

To investigate the fHAT, the TS was calculated as described in the previous section, using a triplet spin multiplicity. The relevant points on the reaction path are depicted in Fig. S9.

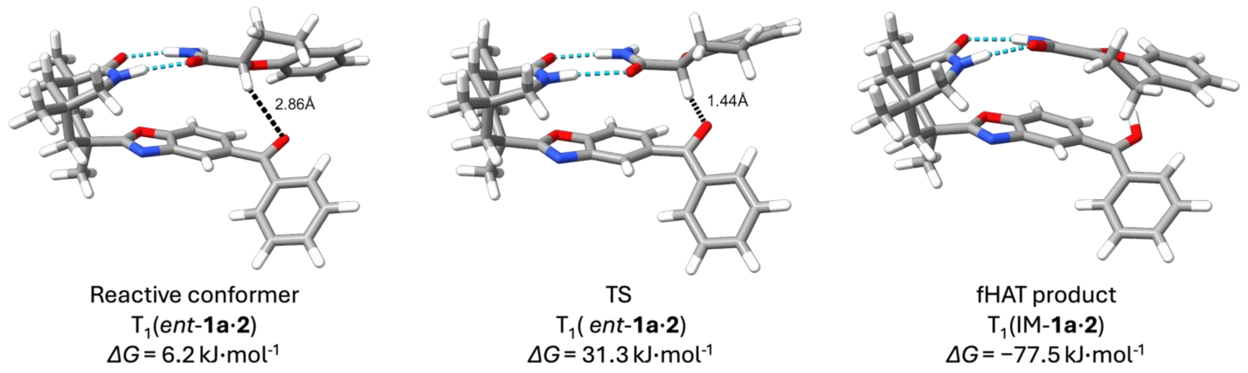

Fig. S9: Reactant (left), transition state (middle) and product (right) of the fHAT of *ent*-**1a**·**2**. Relative free energy is in reference to the global minimum conformer, depicted in Fig. S8 and as T_1(*ent*-**1a**·**2**) in the top Scheme 5 in the main article.

The calculated barrier for the fHAT TS (Fig. S9) is 31.3 kJ·mol^{-1}. To evaluate the kinetic feasibility of this reaction step, the Eyring equation was used to estimate the half life:

$$t_{1/2} = \ln(2) \cdot \left(\frac{k_B T}{h} \cdot \exp\left(-\frac{\Delta G^{\ddagger}}{RT} \right) \right)^{-1} \quad (2)$$

$$= \ln(2) \cdot \left(\frac{1.38 \cdot 10^{-23} \frac{\mathrm{J}}{\mathrm{K}} \cdot 303.15\ \mathrm{K}}{6.63 \cdot 10^{-34}\ \mathrm{J\ s}} \exp\left(-\frac{31300\ \frac{\mathrm{J}}{\mathrm{mol}}}{8.32\ \frac{\mathrm{J}}{\mathrm{mol\ K}} \cdot 303.15\ \mathrm{K}} \right) \right)^{-1}$$

$$= 27\ \mathrm{ns}$$

The half life of 27 ns is sufficiently small for the reaction to happen in the typical lifetime of a triplet excited state. The product of this fHAT step is a biradical (IM-**1a**·**2**), with the hydrogen atom transferred from the chiral C2 carbon of *ent*-**1a** to the benzophenone's carbonyl oxygen (Fig. S9, right).

The same methodology is applied to *ent*-**12** and **12**. The fHAT transition states yield barriers of +43.0 kJ·mol^{-1} for *ent*-**12**·**2** and +47.9 kJ·mol^{-1}for **12**·**2**, respectively. The comparable fHAT barrier heights of both enantiomers (*ent*-**12** and **12**), along with the similar catalyst association free energies, align with the experimental observation that *rac*-**12** does not undergo deracemization.

Back Hydrogen Atom Transfer

To identify possible backward hydrogen transfer (bHAT) pathways, minimum energy crossing points (MECP) between the T_1 and S_0 states were computed. To identify initial MECP structures, we carried out an MECP search at the GFN2-xTB level of theory as implemented in CREST. The used algorithm follows a derivative coupling vector-free formalism, which is described in more detail in the corresponding references.[89,54] Here, we will only briefly outline some relevant features of this methodological choice.

To approximate an MECP's energy at the GFN2-xTB level, an artificial seam PES is constructed averaging the energies of a closed-shell ground state (S_0) and an open-shell excited state (S_1/T_1, which are indistinguishable in GFN2-xTB). An energy gap-dependent bias potential is then applied to guide the system toward a crossing point of these two states.[54] To maintain practicability in the present multilevel computational protocol and to manage the large numbers of generated structures, the molecular identifier MolBar[90,91] was used to filter duplicate structures and implausible geometries based on the molecular connectivity identifiers.

Subsequently, selected MECP guesses were reoptimized at the PBEh-3c+CPCM(DCM) level of theory using UKS-DFT. In this step, CREST addresses TeraChem[92] for the DFT gradient computations (generic runmode).[44] For each reoptimized MECP geometry, a UKS-DFT T_1 and an RKS-DFT S_0 geometry optimization was performed. If a hydrogen atom transfer is observed during these optimizations, the MECP and the corresponding local T_1 minimum structure were considered to derivate an approximate barrier for the bHAT process.

A second approach was used for generating MECP structure candidates that are involved in the bHAT. Here, conformer sampling of the fHAT product T1(IM-**1a**·**2**) was performed, followed by

a MECP reoptimization at the PBEh-3c+CPCM(DCM) theory level. Similar to the first approach, each MECP was used for a UKS-DFT T_1 and an RKS-DFT S_0 geometry optimization.

Both approaches yielded two different pathways, resulting in the same outcome: the formation of the achiral enol **1a’**. These pathways are depicted in Fig. S10. The MECP-based search generated a structure with a relative free energy of 5.0 kJ·mol^{-1} relative to the fHAT product. The conformer sampling-based approach yielded a structure with a relative free energy of 6.6 kJ·mol^{-1} (equivalent to 185 kJ·mol^{-1} in Scheme 5 of the main article).

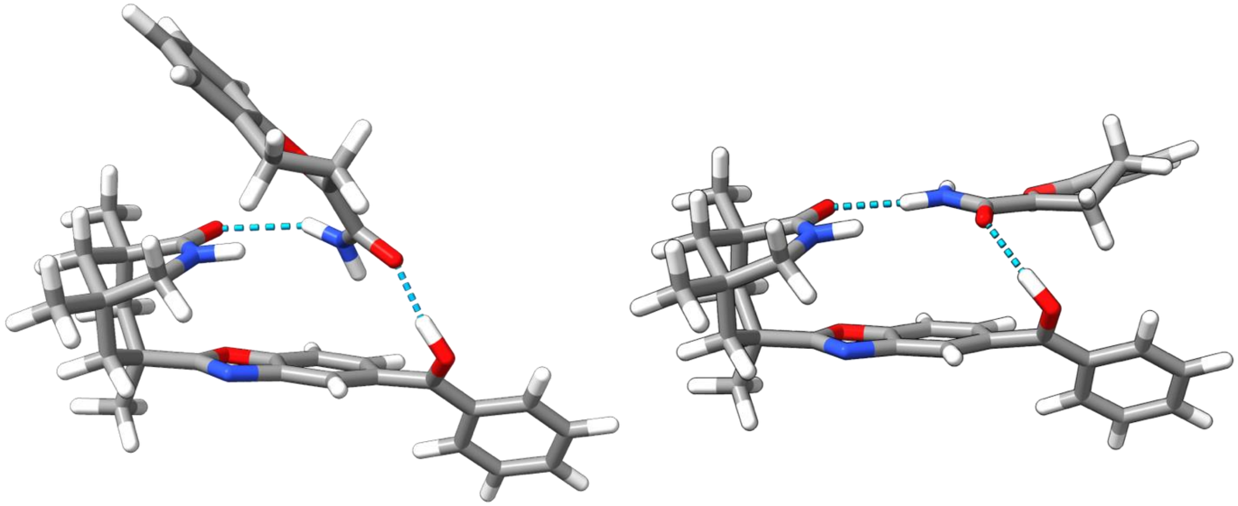

Fig. S10: Two identified T_1/S_0 MECP structures, determined via the MECP screening algorithm (left) and conformer sampling followed by MECP reoptimization (right). Reference for the relative free energy is the fHAT product T_1(IM-**1a**·**2**).

Both MECP pathways lie energetically close with an energy difference of 1.6 kJ·mol^{-1}. In the main article, we decided to show and discuss mainly the energetically slightly higher structure, since it is structurally closer to the minimum free energy conformer and requires less motion and no additional barrier to be overcome. Furthermore, the free energy difference is negligible, if we consider typical error margins and thermal energy at room temperature.

For both cases, reverse ISC towards the ground state complex **1a’**·**2** with a HAT to the amide oxygen atom is proposed. The effective association energy of the enol **1a’** with the catalyst amounts to −6.2 kJ·mol^{-1} (see also Table S3), which is higher than that of complex *ent*-**1a**·**2** (−11.6 kJ·mol^{-1}, Table S2). Hence, we expect **1a’** at the binding site of catalyst **2** to soon be

replaced by a substrate molecule (*ent*-**1a** or **1a** at later stages) after some equilibration time. The subsequent reaction of **1a'** by means of tautomerization is discussed in the following section.

Tautomerization

As described above, the product of the conversion of *ent*-**1a** is the achiral enol **1a'**. Given that experimental yields exceeded 50% and the *ee* reaches 94% (Scheme 2 in the main article), full conversion of *ent*-**1a** to **1a** must occur. Literature suggests that related systems undergo tautomerization via bimolecular mechanisms.[93] To account for the full reaction path, the tautomerization of enol **1a'** towards 1a is therefore investigated.

First, intramolecular tautomerization is investigated. The TS was located via a scan of the hydrogen from the enol group back to the formerly chiral center, as described in Chapter *Transition State Calculation*.

The resulting TS (Fig. S11) exhibits a reaction barrier of 121.3 kJ·mol^{-1}. Similar to what was discussed previously, equation 2 is used to evaluate the kinetic feasibility of the intramolecular tautomerization:

$$t_{1/2} = \ln(2) \cdot \left(\frac{1.38 \cdot 10^{-23} \frac{\mathrm{J}}{\mathrm{K}} \cdot 303.15\ \mathrm{K}}{6.63 \cdot 10^{-34}\ \mathrm{J\ s}} \exp\left(- \frac{121300\ \frac{\mathrm{J}}{\mathrm{mol}}}{8.32\ \frac{\mathrm{J}}{\mathrm{mol\ K}} \cdot 303.15\ \mathrm{K}} \right) \right)^{-1}$$

$$= 2.68\ \mathrm{a}$$

A half life on the order of years renders this pathway kinetically inaccessible under given reaction conditions. Thus, we additionally consider bimolecular mechanisms, where potential reaction partners include the substrates **1a** and *ent*-**1a**, PhSH and traces of water. Docking and TS localization are performed following the methods outlined in Sections *Computational Details* and *Transition State Calculation*, using **1a'** as the tautomerizing species.

All bimolecular mechanisms are computed via a concerted hydrogen shuffling, where a proton from the reaction partner is transferred to the formerly chiral carbon of **1a'**, while the enol proton is transferred to the partner. An exception to this poses the reaction of **1a'** with thiophenol, where the proton transfers first from PhSH to the chiral carbon, followed by a barrierless proton transfer from the enol to PhS^{-}. The TSs for mono- and bimolecular

tautomerization towards **1a** are shown in Fig. S11. Barriers are computed assuming standard concentration of 1 mol·L^{-1}. The lower concentration present in solution, in fact, would further increase the barriers. All values are referenced against the most stable associated dimeric or separated species, with association energies listed in Table S3.

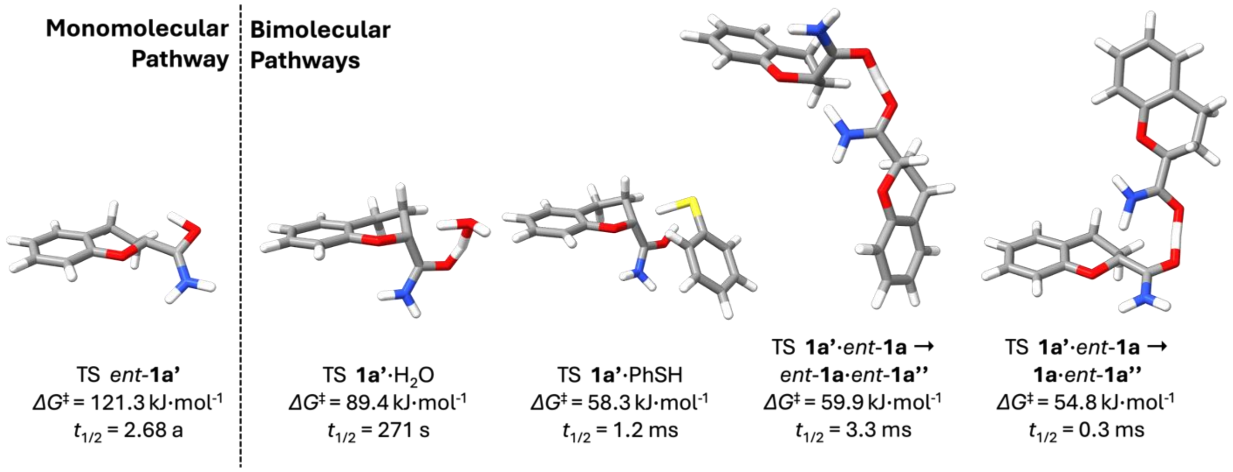

Fig. S11: Transition states for tautomerization of **1a'** to **1a** via different mono- or bimolecular pathways. Reference for the barriers are the most stable (dimeric) species of the pure compound (see Table S3). For water, a free molecule was used for simplicity. Half times are calculated according to equation 2.

Among the computed TSs, tautomerization via thiophenol and substrate molecules exhibit feasible barriers. Given that the thiophenol concentration is lower than the substrate concentration, tautomerization of **1a'** to (*ent*-)**1a** is likely driven by reaction with (*ent*-)**1a**. Since enantiomeric structures are isoenergetic, tautomerization of **1a'** yields **1a** and *ent*-**1a** with equal probability (furthermore, the stereoselectivity of the (*ent*-)**1a** co-substrate in the tautomerization was found to be minor). The latter is returned to the catalytic cycle, thus enabling enrichment of the chromane **1a** in solution. A side product of the tautomerization of **1a'** with the substrate molecules is the formation of the chiral imidic acid (*ent*-)**1a''**, which reconverts back to the corresponding chromane (*ent*-)**1a** spontaneously via a dimeric proton exchange, as illustrated in Fig. S12:

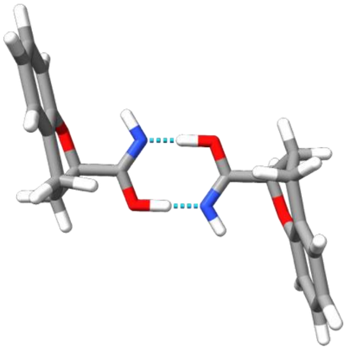

Fig. S12: Transition state for conversion of dimeric imidic acid **1a''·1a''** back to chromane **1a**.

The association free energy for the formation of the dimer **1a"·1a"** is exergonic with $\Delta G_{\text{assoc}} = -38.9$ kJ·mol^{-1}. From this, the proton exchange occurs without a barrier, relative to the dimer species (see Table S3). Thus, the substrate is regenerated and should not impede the catalytic cycle.

Table S3: Association free energies and effective free energies (ΔG_{eff}) for tautomerization complexes.

Reaction	ΔG_{assoc} (kJ·mol^{-1})	ΔG_{eff} (kJ·mol^{-1})
**1a'** + PhSH	11.7	12.2
**1a'** + *ent*-**1a**	−3.5	−3.0
**1a'** + H_2O	12.8	13.3
**1a'** + **2**	−10.3	−6.2
2×**1a'**	−1.1	-
2×**1a"**	−38.9	-

Calculation of the Kinetic Isotope Effect

To compare the experimentally determined kinetic isotope effect (KIE) with theoretical predictions, the reaction barrier of the rate-determining step was recalculated for the deuterated species. This is done by computing G_{TRV} for both the minima and TS structures of the deuterated analogues. The previously calculated geometries were used in these calculations, adjusting the mass of the transferred hydrogen atom to deuterium to M 2.00141 amu in the harmonic frequency calculations in ORCA. Since the electronic energy and solvation corrections are unaffected by the atomic masses, these remain unchanged between the protonated and deuterated structures. The

calculated free energies of the minima and TS structures are used to derive the reaction barriers for the undeuterated ($\Delta G_{\mathrm{H}}^{\ddagger}$) and deuterated ($\Delta G_{\mathrm{D}}^{\ddagger}$) systems. The KIE is subsequently estimated as:

$$\frac{k_{\mathrm{H}}}{k_{\mathrm{D}}} = \exp\left(-\frac{\Delta G_{\mathrm{H}}^{\ddagger} - \Delta G_{\mathrm{D}}^{\ddagger}}{RT}\right)$$

The KIE is calculated from the turnover-limiting step, which is given by the highest barrier in the catalytic cycle. According to our quantumchemical calculations, this corresponds to the tautomerization of **1a’** in the deracemization reaction of *rac*-**1a**, under the provision that the substrate is still bound to the catalyst. For completeness, however, the KIE of the fHAT step is also evaluated. The corresponding results are presented in Table S4.

Table S4: Computed activation barriers and KIEs for selected steps in the deracemization reaction of *rac*-**1a**.

Reaction	$\Delta G^{\ddagger}$ (kJ·mol^{-1})		KIE
	H	D	$k_{\mathrm{H}}/k_{\mathrm{D}}$
**1a’** + *ent*-**1a** → *ent*-**1a** + *ent*-**1a”**	59.9	61.8	2.1
**1a’** + *ent*-**1a** → **1a** + *ent*-**1a”**	54.8	57.5	3.0
**1a’** + PhSH	58.3	-	-
fHAT	31.3	35.2	5.0

The computed KIE of 2.1 for reaction **1a’** + *ent*-**1a** → *ent*-**1a** + *ent*-**1a”** aligns with the experimentally determined KIE of 2.3$\pm$0.1. The reaction leading to the other enantiomer (second line) **1a’** + *ent*-**1a** → **1a** + *ent*-**1a”** exhibits a higher theoretical KIE of 3.0, which is also in the right ballpark.

Nevertheless, it is essential to treat the KIE interpretation with caution: While the fHAT KIE from the quantum chemical computations appears to be quite high, it should be remembered that the overall photochemical process until after the fHAT took place also involves light absorption and vibronically affected ISC. These preceding steps are difficult to quantify but may render the photochemical pathways including the fHAT to be overall turnover-determining.

In that case of multiple subsequent steps, an experimentally determined lower KIE is likely. Hence, we cannot identify the overall turnover-determining step and the origin of the KIE without ambiguity.

Role of Thiophenol

Finally, we wish to examine the role of additives used for the deracemization experiments computationally. Experimental screening of various additives revealed that thiophenol (PhSH) can improve both yield and *ee*% in the deracemization of *rac*-**1a**.

The ground state association of PhSH to *ent*-**1a·2** is calculated to be endergonic with +13.0 kJ·mol^{-1}. This renders a direct incorporation to form trimeric species in solution unlikely. From the above-described vertical excitation energies (Fig. S7), we furthermore conclude that the excited singlet states of PhSH are not directly accessible at the given irradiation conditions. As a consequence of these two findings, we anticipate that PhSH is not mandatory for fHAT to occur and that it does not directly influence this step of the reaction mechanism. Still, experimentally, deuteration experiments indicate that it is involved somehow in the hydrogen transfer processes.

We find that PhSH may facilitate the bHAT reaction step, and to a lesser degree, the subsequent tautomerization. This observation is underlined from the association free energy derived for the fHAT product complex in its excited triplet state T_1(IM-**1a·2**), where the formation of a trimeric complex is only slightly endergonic with 6.9 kJ·mol^{-1}. In this complex, PhSH would be located between the amide oxygen and the benzophenone oxygen (see Scheme 5 in the main article).

Conformer search followed by MECP optimization (Section *Backward HAT*) and successive RKS-DFT S_0 optimization reveals a concerted hydrogen atom transfer mechanism to be operative. Specifically, PhSH here serves as the hydrogen atom source for the amide oxygen, while the hydrogen from the benzophenone moiety is transferred to PhSH. The result of this "double hydrogen transfer" is the formation of the achiral enol **1a'**, i.e., the same product formed via the direct bHAT pathway described previously. Notably, the only energetic barrier in this PhSH-assisted mechanism is the initial association energy, which is thermodynamically and statistically disfavored in solution. However, this mechanism does not require disruption of internal hydrogen bonds, unlike in the unassisted bHAT pathways. Based on these indications, we further argue that PhSH may participate in the bHAT offering an additional, isoenergetic pathway.

Furthermore, it may participate in the tautomerization of **1a'** to **1a**, as discussed previously. However, this contribution is expected to be minor due to the relatively low concentration of thiophenol compared to the substrate. At the same time, the PhSH-mediated tautomerization barrier is slightly higher in free energy compared to the substrate-mediated tautomerization (see Fig. S11). Overall, we conclude that the primary role of PhSH is to facilitate bHAT. This

interpretation can be brought in agreement with observations from thiol additive screening (Scheme 2). Here, bulkier thiophenols are found to be less effective, possibly due to steric hindrance within the confined binding pocket of the *ent*-**1a**·**2** complex.

Free Energy Contributions

Table S5: Free energy contributions for all species investigated in this study (equation 1), divided into isolated (monomeric) and associated (dimeric and trimeric) species. The values were calculated on the theory level outlined in Table S1. MECP electronic energies are calculated with PBEh-3c+CPCM(DCM) as described in Section *Backward HAT*.

System	State	E_{el} (Eh)	G_{TRV} (Eh)	δG_{solv} (Eh)	G (Eh)
		Monomeric			
**2**	S_0	-557,987713	0,178968	-0,017689	-557,826434
	T_1	-557,846989	0,170754	-0,017848	-557,694083
**ent1a**	S_0	-593,940533	0,155688	-0,016817	-593,801662
	S_0 conformer 2	-593,940534	0,156278	-0,016196	-593,800451
	S_0 conformer 3	-593,932209	0,156117	-0,018440	-593,794532
	T_1	-593,803132	0,145019	-0,016944	-593,675057
**ent12**	S_0	-557,987762	0,178789	-0,017676	-557,826649
	S_0 conformer 2	-557,988401	0,179553	-0,017093	-557,825941
	S_0 conformer 3	-557,985552	0,179241	-0,017516	-557,823827
**1a'**	S_0	-593,901785	0,155164	-0,013856	-593,760476
TS **1a'**	S_0	-593,832056	0,150621	-0,015769	-593,697204
**1a''**	S_0	-593,918563	0,156109	-0,014102	-593,776557
PhSH	S_0	-631,205745	0,066584	-0,006824	-631,145986
H_2O	S_0	-76,550186	0,002510	0,000193	-76,547483
		Dimeric			
**2**·**2**	S_0	-2611,705403	0,834727	-0,075901	-2610,946578
**1a**·**1a**	S_0	-1187,903389	0,335562	-0,032561	-1187,600389
ent-**1a**·**1a**	S_0	-1187,903393	0,334268	-0,033530	-1187,602655
**12**·**12**	S_0	-1115,999463	0,380633	-0,034981	-1115,653811
ent-**12**·**12**	S_0	-1115,999066	0,379681	-0,035521	-1115,654906
**1a**·**2**	S_0	-1899,807209	0,583966	-0,054915	-1899,278158
	T_1	-1899,702529	0,581376	-0,055557	-1899,176709
ent-**1a**·**2**	S_0	-1899,809611	0,584156	-0,053895	-1899,279350
	T_1	-1899,704034	0,581577	-0,055274	-1899,177731
	T_1 conformer 2	-1899,700371	0,580936	-0,055952	-1899,175387
	T_1 deuterated	-1899,704034	0,578150	-0,055274	-1899,181158
TS *ent*-**1a**·**2**	T_1	-1899,689771	0,578242	-0,054290	-1899,165819
	T_1 deuterated	-1899,689771	0,576320	-0,054290	-1899,167741
IM-**1a**·**2**	T_1	-1899,734684	0,580646	-0,053248	-1899,207285
	T_1 conformer 2	-1899,732620	0,581284	-0,053307	-1899,204642
	MECP 2	-1892,362100	-	-	-
	T_1 conformer 3	-1899,734479	0,581954	-0,052728	-1899,205253

	MECP 3	-1892,364273	-	-	-
**1a'**·**2**	S_0	-1899,767405	0,584941	-0,053861	-1899,236325
**12**·**2**	S_0	-1863,857325	0,608658	-0,054646	-1863,303312
	S_0 conformer 2	-1863,856785	0,607924	-0,053660	-1863,302521
	T_1	-1863,750542	0,604965	-0,055831	-1863,201407
TS **12·2**	T_1	-1863,730376	0,601072	-0,053860	-1863,183164
ent-**12·2**	S_0	-1863,857612	0,608507	-0,054179	-1863,303284
	S0 conformer 2	-1863,856112	0,608193	-0,054644	-1863,302563
	T_1	-1863,750640	0,605051	-0,055392	-1863,200981
TS *ent*-**12·2**	T_1	-1863,732965	0,601383	-0,053037	-1863,184619
**1a'**·**1a'**	S_0	-1187,828487	0,336268	-0,029132	-1187,521351
**1a''**·**1a''**	S_0	-1187,872607	0,333765	-0,029068	-1187,567911
TS **1a''**·**1a''**	S_0	-1187,871490	0,331173	-0,028573	-1187,568890
**1a'**·PhSH	S_0	-1225,121724	0,242628	-0,022929	-1224,902025
TS **1a'**·PhSH	S_0	-1225,099751	0,242378	-0,026879	-1224,884252
**1a'**·H_2O	S_0	-670,458561	0,174811	-0,019350	-670,303099
TS **1a'**·H_2O	S_0	-670,428994	0,172361	-0,017262	-670,273895
**1a'**·*ent*-**1a**	S_0	-1187,862973	0,333355	-0,033866	-1187,563484
	S_0 deuterated	-1187,862973	0,326602	-0,033866	-1187,570237
TS to **1a**	S_0	-1187,837670	0,326074	-0,031006	-1187,542602
**1a'**·*ent*-**1a**	S_0 deuterated	-1187,837670	0,320340	-0,031006	-1187,548336
TS to *ent*-**1a**	S_0	-1187,836755	0,327510	-0,031432	-1187,540677
**1a'**·*ent*-**1a**	S_0 deuterated	-1187,836755	0,321478	-0,031432	-1187,546709
			Trimeric		
ent-**1a**·**2**·PhSH	S_0	-2531,032873	0,674603	-0,062108	-2530,420378
IM-**1a**·**2**·PhSH	T_1	-2530,961051	0,671124	-0,060701	-2530,350627
	MECP	-2521,855313	-	-	-

S5. Optimization of the Reaction Conditions and Profile of the Deracemization Reaction

The reaction conditions were first optimized for chroman-2-carboxamide *rac*-**1a** using catalysts (+)-**2** and its regioisomer (+)-**2'** without the addition of any additives (Table S6).

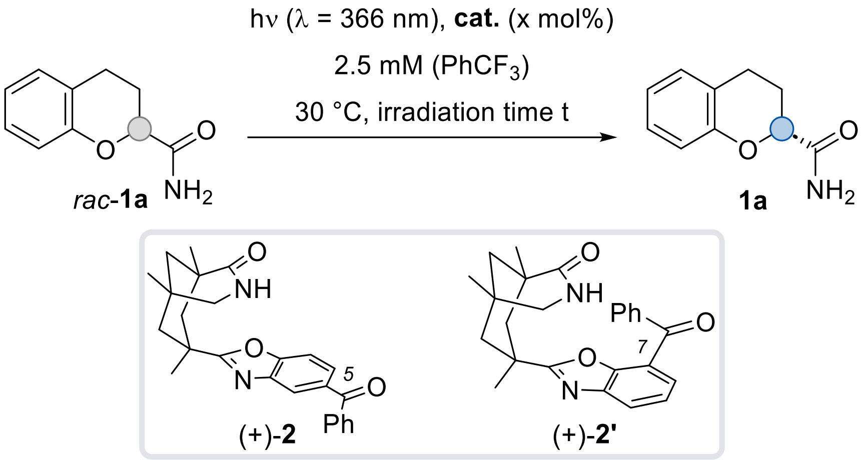

Table S6: Optimization of reaction conditions for the deracemization of *rac*-**1a**. All reactions were performed on a 25.0 μmol scale.

Entry	**cat.**	x [mol%]	t [h]	yield [%]	*ee* [%]
1	(+)-**2**	5	7	93	40
2	(+)-**2**	10	13	89	58
3	(+)-**2'**	10	13	91	17
4	(+)-**2**	15	13	71	83
5	(+)-**2**	10	18	82	85
6	(+)-**2**	10	24	81	80

In order to further increase both the *ee* and the yield of **1a** obtained with conditions 5, several additives were screened (Fig. S13, Table S7).

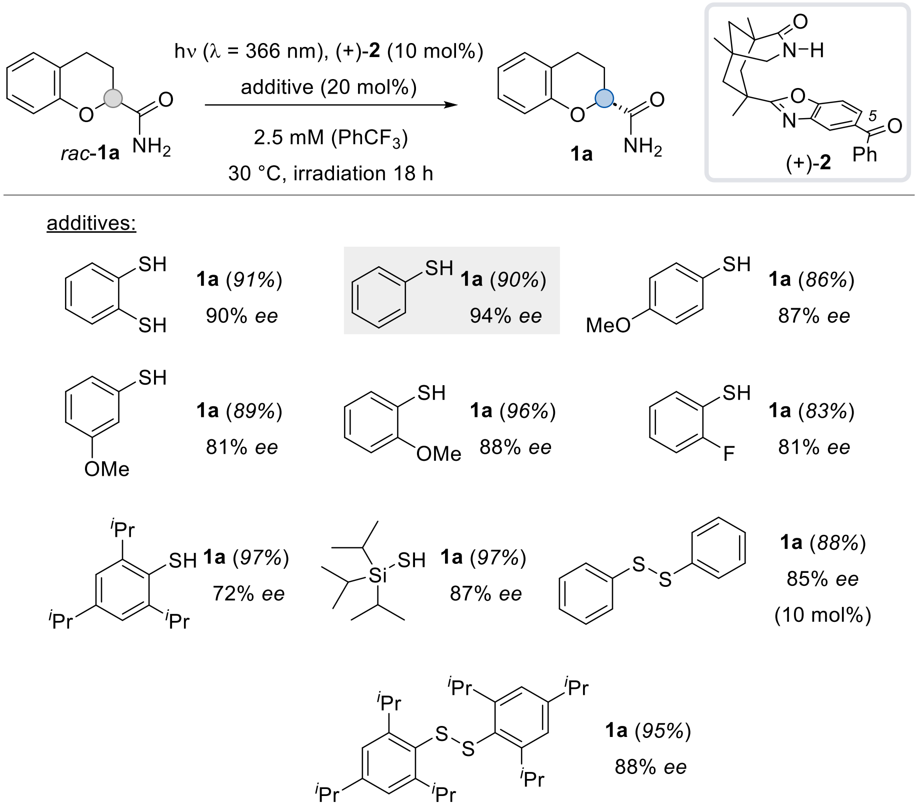

Fig. S13: Optimization of additives for the deracemization of *rac*-**1a**. All reactions were performed on a 25.0 μmol scale.

Table S7: Optimization of reaction conditions for the deracemization of *rac*-**1a** with PhSH as an additive. All reactions were performed on a 25.0 µmol scale.

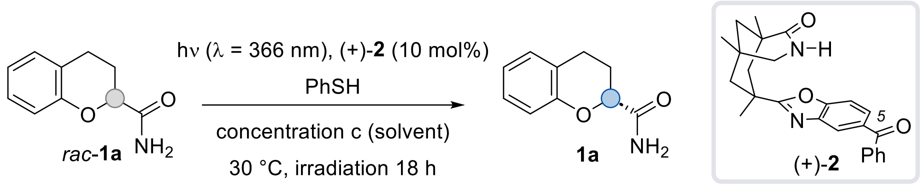

Entry	solvent	PhSH [mol%]	c [mM]	yield [%]	*ee* [%]
1	$PhCF_3$	20	2.5	90	94
2	$PhCF_3$	10	2.5	93	86
3	$PhCF_3$	40	2.5	90	83
4	$PhCF_3$	100	2.5	84	68
5	PhCl	20	2.5	74	79
6	DCM	20	2.5	97	15
7	MeCN	20	2.5	97	9
8	$PhCF_3$	20	5.0	86	75

With optimal reaction conditions in hand, we investigated the rate profile of the deracemization of *rac*-**1a**. For this, aliquots of the reaction mixture were taken at different time points and the *ee* of **1a** was analyzed (Fig. S14).

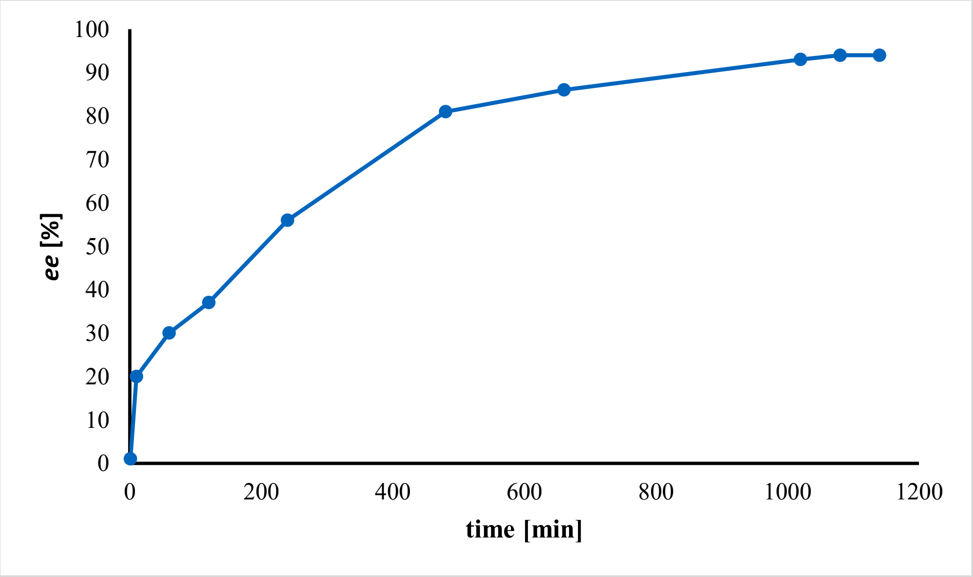

Fig. S14. Development of the *ee* of **1a** relative to the reaction time.

S6. Determination of the Absolute Configuration of Deracemization Product 1a

The absolute configuration of chromane-2-carboxamide **1a** was determined as (*R*) by comparison of the retention times of **1a** with *ent*-**1a**, which was prepared from the commercially available (*S*)-chromane-2-carboxylic acid following GP F, on chiral HPLC.

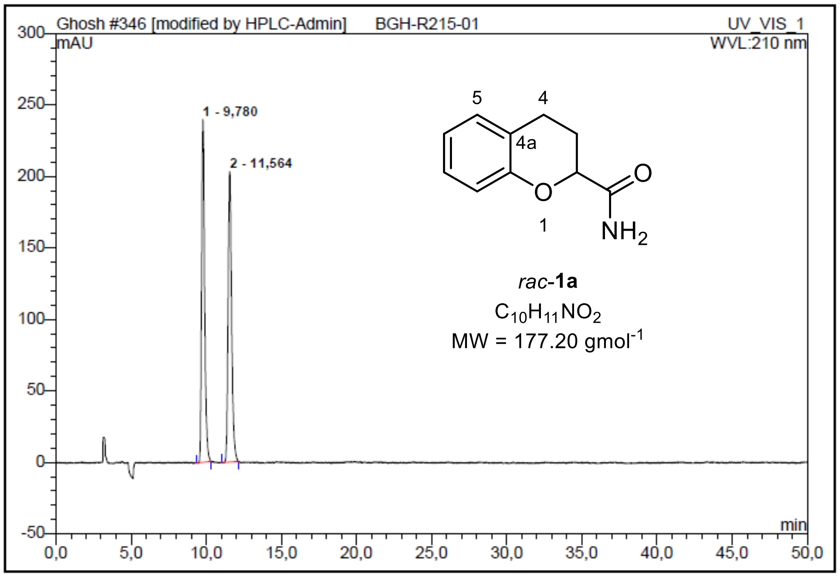

No.	Ret.Time min	Peak Name	Height mAU	Area mAU*min	Rel.Area %	Amount	Type
1	9,78	n.a.	239,871	55,231	50,06	n.a.	BMB*
2	11,56	n.a.	203,014	55,099	49,94	n.a.	BMB*
Total:			442,886	110,330	100,00	0,000	

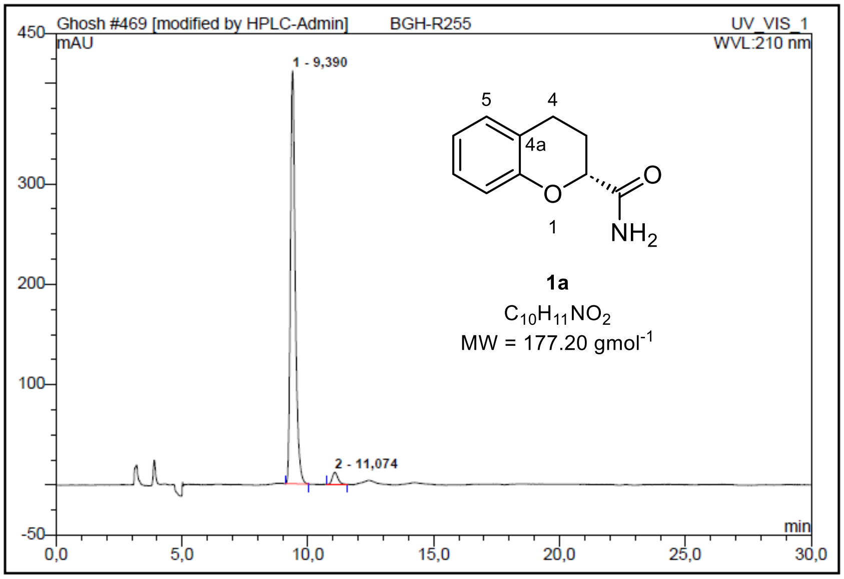

No.	Ret.Time min	Peak Name	Height mAU	Area mAU*min	Rel.Area %	Amount	Type
1	9,39	n.a.	411,787	91,188	96,68	n.a.	BMB
2	11,07	n.a.	12,083	3,128	3,32	n.a.	BMB*
Total:			423,870	94,317	100,00	0,000	

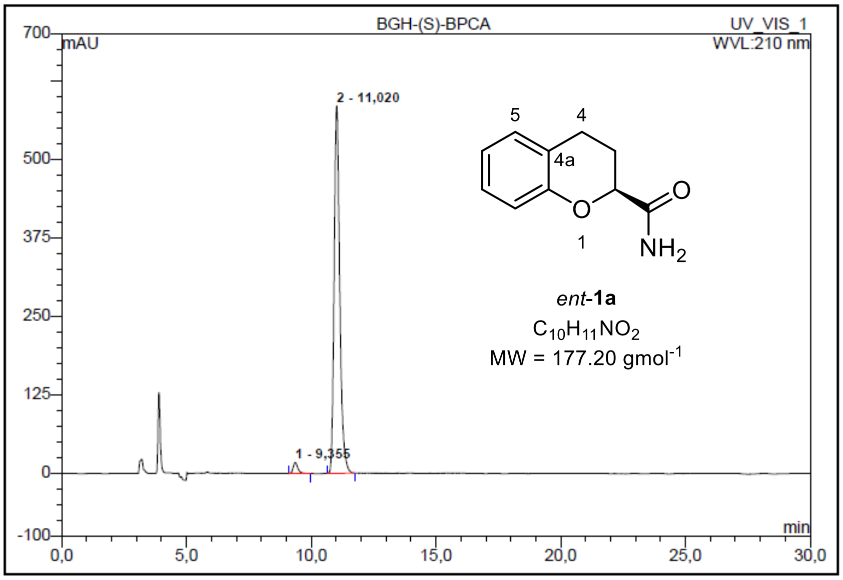

No.	Ret.Time min	Peak Name	Height mAU	Area mAU*min	Rel.Area %	Amount	Type
1	9,35	n.a.	17,799	3,990	2,56	n.a.	BMB*
2	11,02	n.a.	584,733	151,918	97,44	n.a.	BMB
Total:			602,532	155,908	100,00	0,000	

S7. Determination of the Kinetic Isotope Effect (KIE)

The kinetic isotope effect (KIE) is defined as

$$\mathrm{KIE} = \frac{k_H}{k_D}$$

The rate constants k_H and k_D cannot be determined individually without knowledge of the rate law. However, the ratio of k_H to k_D can be calculated from the ratio of the two reaction rates r_H and r_D at time t = 0 under identical reaction conditions (with $r_H = k_H \cdot c$ and $r_D = k_D \cdot c$).

$$\mathrm{KIE} = \frac{k_H}{k_D} = \frac{\frac{r_H}{c}}{\frac{r_D}{c}} = \frac{r_H}{r_D} \quad (\text{at time } t = 0)$$

For this study, the rates r for the inversion of the stereoinformation of substrates (*S*)-**1a** (r_H) and of (*S*)-**1a**-d_1 (r_D) with a deuterium instead of a proton at the stereogenic center were measured. The enantiopure substrates with (*S*)-configuration were used to prevent undesired side reactions or inhibition of the chiral benzophenone catalyst (+)-**2** by the unprocessed (*R*)-configured substrates. To ensure a concentration-independent zero order reaction profile (y = rx + a), only data points up to a conversion of 15% (15% *ee*) were used and a linear regression was applied.

The slopes of the linear regressions give rise to the rates r_H and r_D (Table S8, S9, S10 and Fig. S15, S16, S17). The photochemical inversions of (*S*)-**1a** and of (*S*)-**1a**-d_1 were conducted as parallel experiments in triplicate.

Procedure:

According to the GP F, two separate dried phototubes (Ø = 1 cm) were charged with (*S*)-**1a** (4.43 mg, 25.0 µmol, 1.00 equiv.) and (*S*)-**1a**-d_1 (4.46 mg, 25.0 µmol, 1.00 equiv.), respectively, and enantiomerically pure (+)-benzophenone **2** (1.01 mg, 2.50 µmol, 10 mol%) was added under an argon atmosphere. In a separate vial, a stock solution of PhSH was prepared by dissolving PhSH (5.00 µL) in dry $PhCF_3$ (1.00 mL). From this stock solution, 103 µL (551 µg, 5.00 µmol, 20 mol%) was added followed by the addition of dry $PhCF_3$ (10 mL). The resulting solution was degassed by being sparged with argon under ultrasonication for 15 min and irradiated at λ = 366 nm at 30 °C. During the reactions, aliquots were taken from the solutions simultaneously, the solvents

removed under reduced pressure and subjected to purification by small scale preparative TLC. The enantiopurity of the samples was then assessed by chiral HPLC.

Table S8: Data for first run. *ee* (H) refers to the *ee* of non-deuterated substrate (*S*)-**1a** and *ee* (D) refers to the *ee* of deuterated substrate (*S*)-**1a**-d_1.

Entry	time [min]	% *ee* (H)	% *ee* (D)
1	0	99	99
2	1	97	98
3	2	95	97
4	3	92	96
5	5	88	94

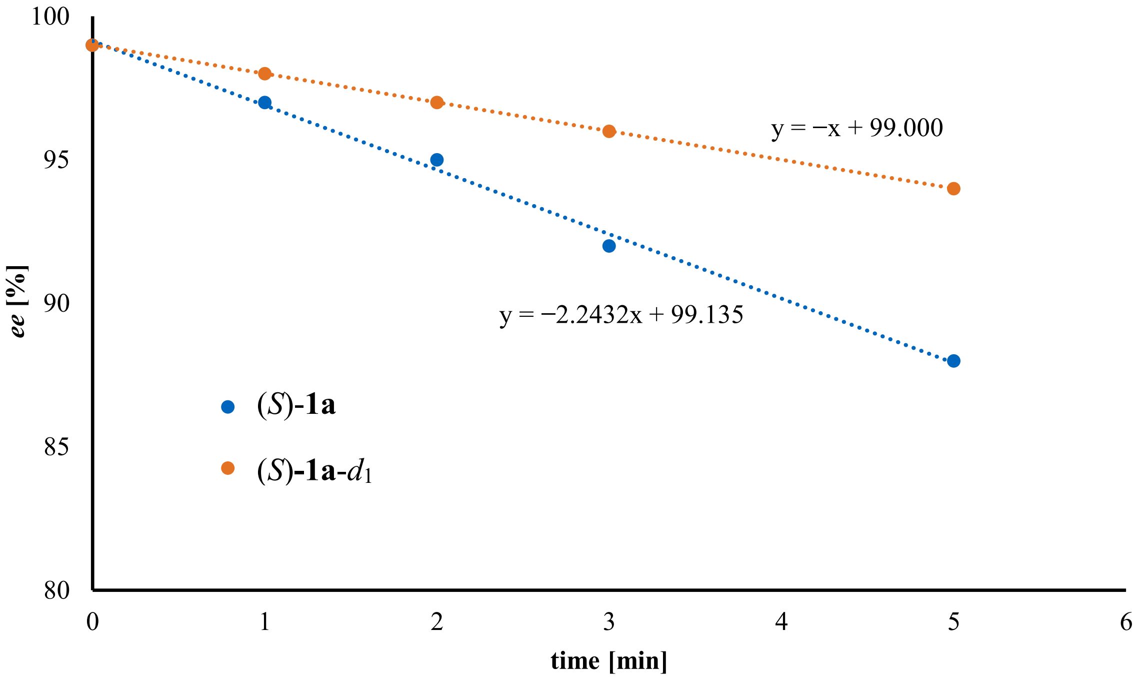

Fig. S15: Determination of r_H and r_D for first run.

Table S9: Data for second run. *ee* (H) refers to the *ee* of non-deuterated substrate (*S*)-**1a** and *ee* (D) refers to the *ee* of deuterated substrate (*S*)-**1a**-d_1.

Entry	time [min]	% *ee* (H)	% *ee* (D)
1	0	99	99
2	1	96	98
3	2	93.5	97
4	3	91.5	96
5	4	89	94.5
6	5	87	94

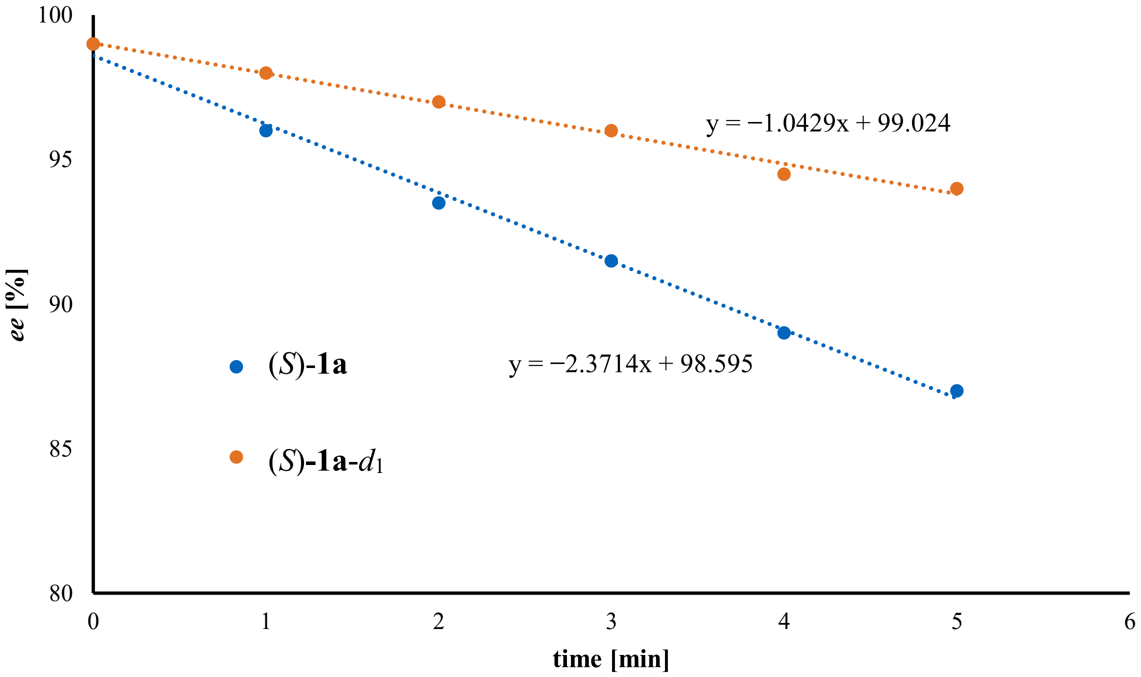

Fig. S16: Determination of r_H and r_D for second run.

Table S10: Data for third run. *ee* (H) refers to the *ee* of non-deuterated substrate (*S*)-**1a** and *ee* (D) refers to the *ee* of deuterated substrate (*S*)-**1a**-d_1.

Entry	time [min]	% *ee* (H)	% *ee* (D)
1	0	99	99
2	1	96	98
3	2	93.5	97
4	3	91	96
5	4	88.75	95
6	5	87.25	94

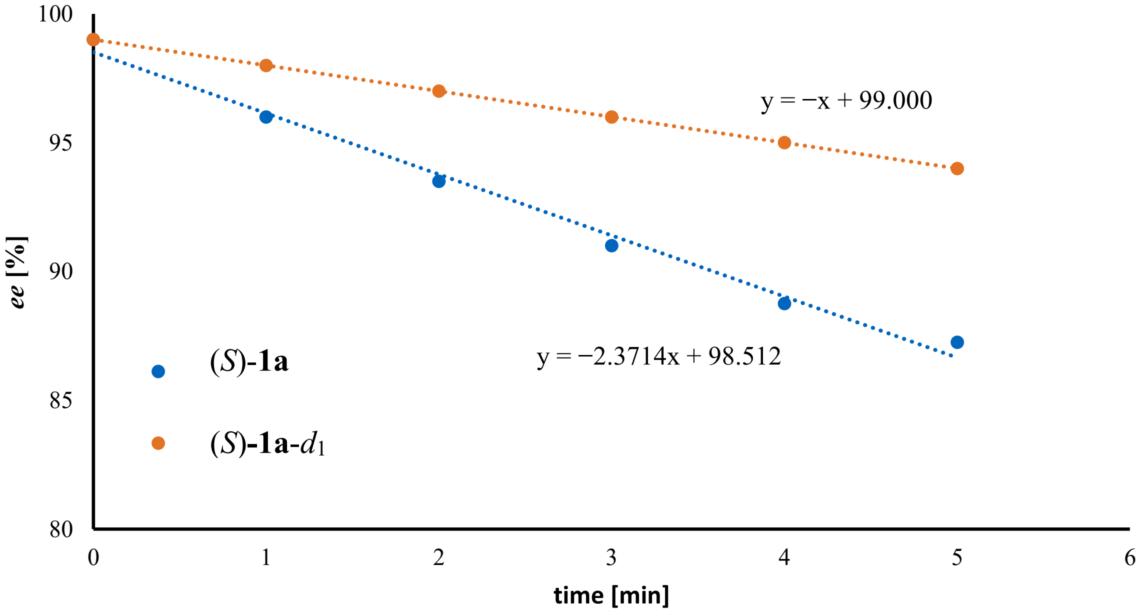

Fig. S16: Determination of r_H and r_D for third run.

Table S11: Overview of the determined reaction rates r_H and r_D.

i	$r_{H,i}$ [% min^{-1}]	$r_{D,i}$ [% min^{-1}]
1	–2.2432	–1.0000
2	–2.3714	–1.0429
3	–2.3714	–1.0000

From the obtained reaction rates r_H and r_D the mean values $\bar{r}_H$ and $\bar{r}_D$ as well as the mean value of the KIE ($\overline{KIE}$) were calculated:

$$\overline{KIE} = \frac{\bar{r}_H}{\bar{r}_D} = \frac{\frac{1}{3}(r_{H,1} + r_{H,2} + r_{H,3})}{\frac{1}{3}(r_{D,1} + r_{D,2} + r_{D,3})} = \frac{\frac{1}{3}[(-2.2432) + (-2.3714) + (-2.3714)]}{\frac{1}{3}[(-1.0000) + (-1.0429) + (-1.0000)]} = \frac{-2.3287}{-1.0143} = 2.2959$$

The standard error of the mean $\sigma_{\bar{r}}$ for a given mean value $\bar{r}$ is calculated as follows (r_i = single measurement of a reaction rate; n = number of experiments):

$$\sigma_{\bar{r}} = \sqrt{\frac{1}{n(n-1)}\sum_{i=1}^{n}(r_i - \bar{r})^2} = \sqrt{\frac{1}{3(3-1)}[(r_1 - \bar{r})^2 + (r_2 - \bar{r})^2 + (r_3 - \bar{r})^2]}$$

Therefore, $\sigma_{\bar{r}_H} = 0.04273$ and $\sigma_{\bar{r}_D} = 0.01430$ were obtained.

The standard error of the mean value of the KIE can be determined from the law of propagation of uncertainty:

$$\sigma_{\overline{KIE}} = \sqrt{\left(\frac{\partial \frac{\bar{r}_H}{\bar{r}_D}}{\partial \bar{r}_H}\right)^2 (\sigma_{\bar{r}_H})^2 + \left(\frac{\partial \frac{\bar{r}_H}{\bar{r}_D}}{\partial \bar{r}_D}\right)^2 (\sigma_{\bar{r}_D})^2} = \sqrt{\left(\frac{1}{\bar{r}_D}\right)^2 (\sigma_{\bar{r}_H})^2 + \left(\frac{-\bar{r}_H}{\bar{r}_D{}^2}\right)^2 (\sigma_{\bar{r}_D})^2} = 0.0531$$

With a confidence interval of 95% (z-value = 1.96), the value of the KIE including the margin of error ($z \cdot \sigma_{\overline{KIE}}$) is determined as follows:

$$KIE = \frac{k_H}{k_D} = \frac{\bar{r}_H}{\bar{r}_D} = 2.2959 \pm 1.96 \cdot 0.0531 \approx 2.3 \pm 0.1$$

S8. H/D Exchange Experiments

Chromane-2-carboxylic acid ethyl ester (*rac*-**S-2a**)

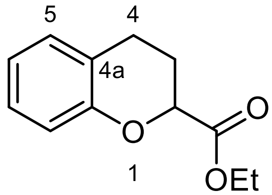

rac-**S-2a**
$C_{12}H_{14}O_3$
MW = 206.24 gmol^{-1}

Conc. H_2SO_4 (110 mg, 60.2 µL, 1.12 mmol, 20 mol%) was added to a solution of chromane-2-carboxylic acid (1.00 g, 5.61 mmol, 1.00 equiv.) in EtOH (22.5 mL) at r.t. and the resulting solution was heated to 60 °C and stirred at this temperature for 15 h. After allowing the solution to cool to r.t., the reaction mixture was concentrated under reduced pressure and a saturated aq. solution of $NaHCO_3$ was added until a pH value of 8 was reached. The mixture was then extracted thrice with CH_2Cl_2 (3 × 20 mL) and the combined organic layers were washed with brine and dried over Na_2SO_4. The solvents were removed under reduced pressure to yield chromane-2-carboxliyc acid ethyl ester (*rac*-**S-2a**) (1.13 g, 5.61 mmol, 98%) as a colorless oil.

^{1}H-NMR (400 MHz, $CDCl_3$, 300 K): δ [ppm] = 7.14 – 7.09 (m, 1H, H7), 7.04 – 7.02 (m, 1H, H5), 6.93 (dd, ^3J = 8.3 Hz, ^4J = 1.3 Hz, 1H, H8), 6.86 (*virt.* dt, ^3J = 7.4 Hz, $^4J \approx {}^4J$ = 1.2 Hz, 1H, H6), 4.71 (dd, ^3J = 7.6 Hz, ^3J = 3.6 Hz, 1H, H2), 4.26 (q, ^3J = 7.1 Hz, 2H, OCH_2), 2.88 – 2.72 (m, 2H, H4), 2.28 (dddd, ^2J = 13.6 Hz, ^3J = 6.7 Hz, ^3J = 5.9 Hz, ^3J = 3.6 Hz, 1H, H3^a), 2.18 (*virt.* dtd, ^2J = 13.6 Hz, $^3J \approx {}^3J$ = 7.8 Hz, ^3J = 5.7 Hz, 1H, H3^b), 1.29 (t, ^3J = 7.1 Hz, 3H, CH_3).

^{13}C-NMR (101 MHz, $CDCl_3$, 300 K): δ [ppm] = 171.1 (COOEt), 153.6 (C8a), 129.5 (C5), 127.7 (C7), 121.4 (C4a), 120.9 (C6), 117.1 (C8), 73.9 (C2), 61.5 (OCH_2), 24.8 (C3), 23.5 (C4), 14.3 (CH_3).

HRMS (ESI) *m/z* $[M+H]^+$: calculated for $[C_{12}H_{15}O_3]^+$: 207.1016; found: 207.1013.

IR (film) $\tilde{\nu}_{max}/cm^{-1}$ = 3042 (w, CH_{arom}), 2991 (w, CH_{arom}), 2937 (m, CH_{aliph}), 2906 (w, CH_{aliph}), 2850 (w, CH_{aliph}), 1752 (m, C=O), 1732 (m, C=O), 1583 (m, C=C), 1489 (m, CH_{arom}), 1457 (m, CH_{aliph}), 1185 (s, C–O), 1165 (s, C–O), 1114 (s, C–O).

Chromane-2-carboxamide (*rac*-**1a-*d*₁**)

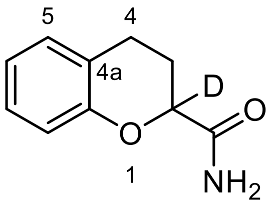

rac-**1a-*d*₁**
$C_{10}H_{10}DNO_2$
MW = 178.21 gmol^{-1}

A solution of ^{n}BuLi in hexanes (2.2 M, 932 mg, 2.05 mL, 14.6 mmol, 5.00 equiv.) was added dropwise to a solution of ^{i}Pr$_2$NH (1.47 g, 2.05 mL, 14.6 mmol, 5.00 equiv.) in THF (36 mL) at −78 °C. The resulting solution was allowed to warm to 0 °C and stirred at this temperature for 10 min before it was cooled to −78 °C again. To this lithium diisopropyl amide solution was added a solution of chromane-2-carboxylic acid ethyl ester (600 mg, 2.91 mmol, 1.00 equiv.) in THF (5.8 mL) and the resulting orange mixture was stirred at −78 °C for 1 h. D_2O (10.8 g, 9.70 mL, 538 mmol, 185 equiv.) was added and the reaction mixture was allowed to warm to r.t.. The mixture was then cooled to 0 °C again and a solution of ^{n}BuLi in hexanes (2.2 M, 932 mg, 2.05 mL, 14.6 mmol, 5.00 equiv.) was added very slowly into the aqueous layer of the unstirred mixture. Once added, the mixture was stirred vigorously at r.t. for 72 h. Acetic acid-d_1 was added until a pH value of 4 was reached in the aqueous layer and the layers were separated. The organic layer was dried over Na_2SO_4 and the solvent was removed under reduced pressure to yield of the crude intermediate chromane-2-carboxylic acid-d_1 (123 mg, 686 μmol, 24%) which was not further purified.

According to GP E, HOBt · H_2O (126 mg, 823 μmol, 1.20 equiv.) was added to a solution of chromane-2-carboxylic acid-d_1 (123 mg, 686 mmol, 1.00 equiv.) in CH_2Cl_2 (23 mL) at 0 °C. After 10 min, EDCI · HCl (158 mg, 823 μmol, 1.20 equiv.) was added and after stirring the resulting mixture at 0 °C for another 30 min, aqueous ammonia (14 M, 701 mg, 2.94 mL, 41.2 mmol, 60.0 equiv.) was added before the reaction mixture was allowed to warm to r.t. and stirred vigorously for 14 h. Then, water (10 mL) was added, the layers were separated and the aqueous layer was extracted thrice with CH_2Cl_2 (3 × 15 mL). The combined organic layers were washed with brine and dried over Na_2SO_4. The solvents were removed under reduced pressure and the crude product was subjected to FCC (SiO_2, 50 → 100% EtOAc/hexanes) to yield chromane-2-carboxamide (*rac*-**1a-*d*₁**) as a white solid (103 mg, 578 mmol, 84%) with a degree of deuteration in 2-position of 99%.

TLC (80% EtOAc/pentane): R_f = 0.55 [UV, $KMnO_4$].

M.p.: 138 °C

^{1}H-NMR (400 MHz, $CDCl_3$, 300 K): δ [ppm] = 7.15 – 7.11 (m, 1H, H7), 7.07 (dd, ^3J = 7.5 Hz, ^4J = 1.6 Hz, 1H, H5), 6.92 – 6.87 (m, 2H, H6, H8), 6.59 (bs, 1H, NH_2^a), 5.87 (bs, 1H, NH_2^b), 2.92 – 2.76 (m, 2H, H4), 2.41 (*virt.* dt, ^2J = 13.7 Hz, $^3J \approx {}^3J$ = 5.4 Hz, 1H, H3[a]), 2.08 (ddd, ^2J = 13.7 Hz, ^3J = 9.7 Hz, ^3J = 5.5 Hz, 1H, H3[b]).

^{13}C-NMR (101 MHz, $CDCl_3$, 300 K): δ [ppm] = 173.8 ($CONH_2$), 153.0 (C8a), 129.9 (C5), 127.7 (C7), 122.2 (C4a), 121.4 (C6), 116.8 (C8), 75.1 (t, $^2J_{CD}$ = 23.1 Hz, C2), 24.7 (C3), 24.1 (C4).

HRMS (ESI) *m/z* $[M+H]^+$: calculated for $[C_{10}H_{11}DNO_2]^+$: 179.0925; found: 179.0923.

IR (film) $\tilde{\nu}_{max}/cm^{-1}$ = 3405 (m, NH), 3161 (m, NH), 3023 (w, CH_{arom}), 2958 (w, CH_{aliph}), 2922 (w, CH_{aliph}), 1655 (s, C=O), 1608 (m, C=C), 1487 (m, CH_{arom}), 1455 (m, CH_{aliph}), 1243 (s, C–O), 1109 (m, C–N).

Thiophenol-*d*$_1$ (PhSD)

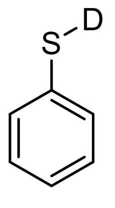

PhSD
$C_6H_5D_S$
MW = 111.18 $gmol^{-1}$

Following a modified procedure by Hussain *et al.,* [88] D_2O (3.83 g, 3.45 mL, 191 mmol, 13.0 equiv.) was added to thiophenol (1.62 g, 1.50 mL, 14.7 mmol, 1.00 equiv.) and the resulting suspension was stirred vigorously for 15 h. The layers were then allowed to separate under an argon atmosphere and the aqueous layer was removed with a syringe. More D_2O (3.83 g, 3.45 mL, 191 mmol, 13.0 equiv.) was added and the mixture was vigorously stirred for another 15 h. The layers were again allowed to separate and the aqueous layer was again removed. No yield was determined and the obtained PhSD was immediately used for deuterium exchange experiments. ^{1}H-NMR indicates a degree of deuteration of at least 90%.

^{1}H-NMR (500 MHz, $CDCl_3$, 300 K): δ [ppm] = 7.32 – 7.24 (m, 4H, H_{aryl}), 7.20 – 7.16 (m, 1H, H_{aryl}).

The spectroscopic data matches the one reported in the literature.[94]

H/D exchange between substrate *rac*-1a and deuterated thiophenol

Following GP F, a dried phototube (Ø = 1 cm) was charged with chromane-2-carboxamide (*rac*-**1a**) (4.43 mg, 25.0 µmol, 1.00 equiv.) and enantiomerically pure (+)-benzophenone **2** (1.01 mg, 2.50 µmol, 10 mol%) under an argon atmosphere. In a separate vial, a stock solution of PhSD was pepared by dissolving PhSD (5.00 µL) in dry $PhCF_3$ (1.00 mL). From this stock solution, 104 µL (556 µg, 5.00 µmol, 20 mol%) was added followed by the addition of dry $PhCF_3$ (10 mL). The resulting solution was degassed by being sparged with argon under ultrasonication for 15 minutes and irradiated at λ = 366 nm at 30 °C for 18 h. After irradiation, the volatile compounds were removed under reduced pressure and the crude reaction mixture was subjected to FCC (SiO_2, 50 → 80% EtOAc/hexanes) to yield **1a**/**1a**-d_1 as a white solid (4.1 mg, 23.1 µmol, 93%, 76% *ee*) (Fig. S17).

a)

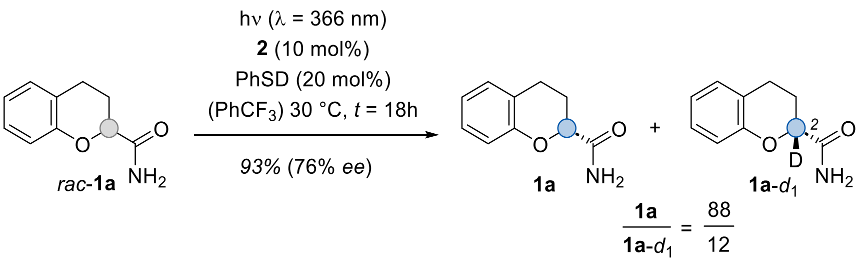

b)

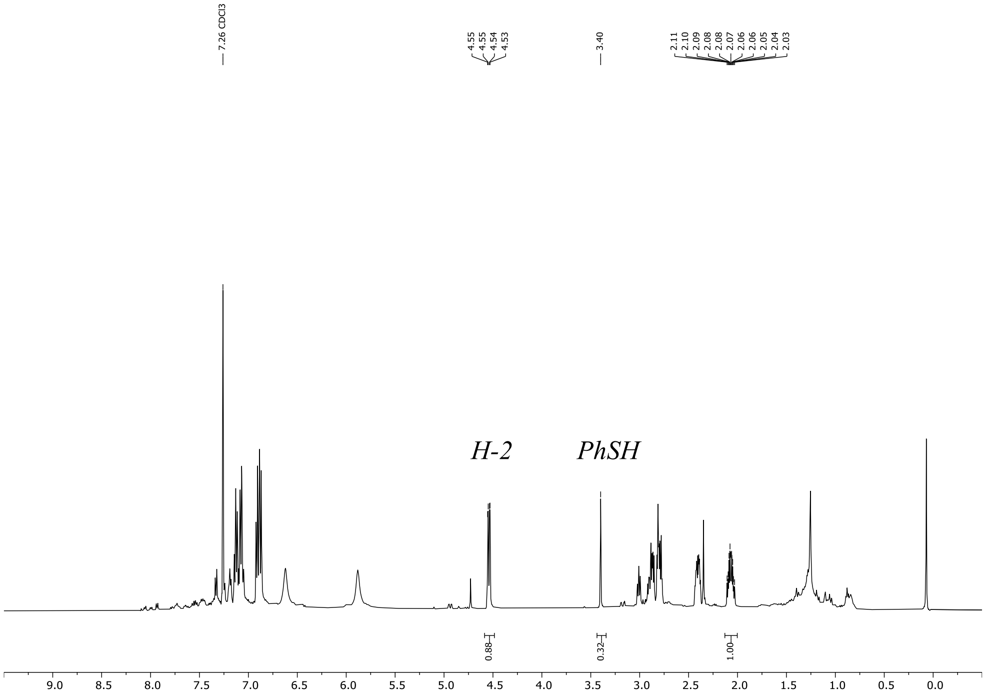

c)

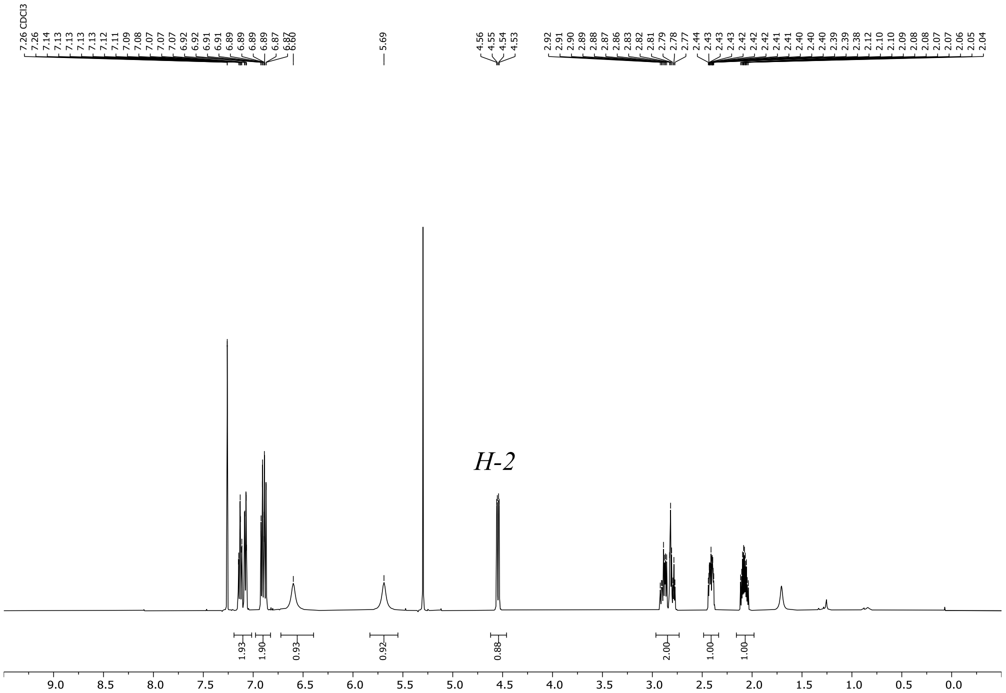

Fig. S17. a) Hydrogen/deuterium exchange experiment between *rac*-**1a** and PhSD under standard conditions. b) Determination of degree of deuteration in 2-position from the crude reaction mixture by ^{1}H-NMR. c) Determination of degree of deuteration in 2-position purification by FCC by ^{1}H-NMR. The NMR spectra were recorded in $CDCl_3$ on an AVHD500 Bruker NMR.

H/D exchange between deuterated substrate *rac*-1a-*d*$_1$ and thiophenol

Following GP F, a dried phototube (Ø = 1 cm) was charged with *rac*-**1a**-*d*$_1$ (4.46 mg, 25.0 μmol, 1.00 equiv.) and enantiomerically pure (+)-benzophenone **2** (1.01 mg, 2.50 μmol, 10 mol%) under an argon atmosphere. In a separate vial, a stock solution of PhSH was pepared by dissolving PhSH (5.00 μL) in dry $PhCF_3$ (1.00 mL). From this stock solution, 103 μL (551 μg, 5.00 μmol, 20 mol%) was added followed by the addition of dry $PhCF_3$ (10 mL). The resulting solution was degassed by being sparged with argon under ultrasonication for 15 minutes and irradiated at λ = 366 nm at 30 °C for 18 h. After irradiation, the volatile compounds were removed under reduced pressure and the crude reaction mixture was subjected to FCC (SiO_2, 50 → 80% EtOAc/hexanes) to yield **1a/1a-*d*$_1$** as a white solid (4.15 mg, 23.3 μmol, 93%, 61% *ee*) (Fig. S18).

a)

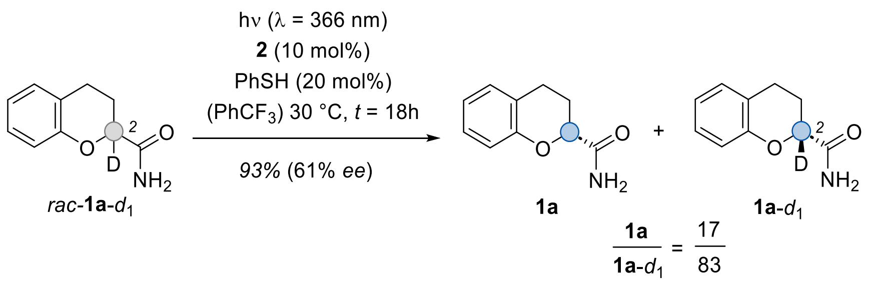

b)

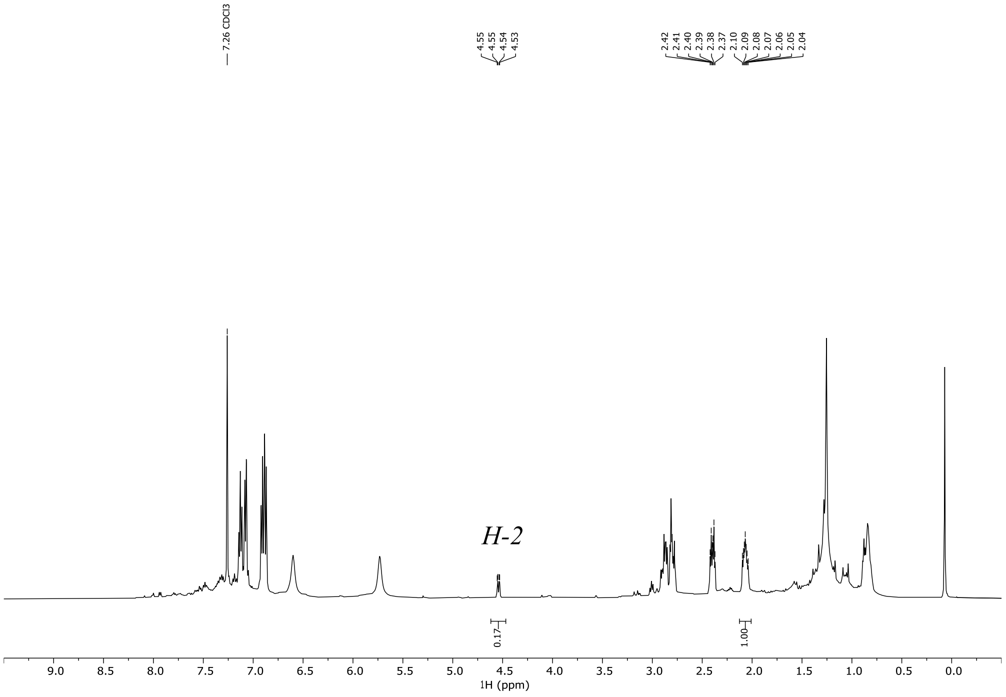

Fig. S18. a) Hydrogen/deuterium exchange experiment between *rac*-**1a**-d_1 and PhSH under standard conditions. b) Determination of degree of deuteration in 2-position from the crude reaction mixture by ^{1}H-NMR. The NMR spectrum was recorded in $CDCl_3$ on an AVHD500 Bruker NMR.

Incorporation of hydrogen in 2-position of deuterated substrate *rac*-1a-d_1 without thiophenol

Following GP F, a dried phototube (Ø = 1 cm) was charged with *rac*-**1a**-d_1 (4.46 mg, 25.0 µmol, 1.00 equiv.) and enantiomerically pure (+)-benzophenone **2** (1.01 mg, 2.50 µmol, 10 mol%) under an argon atmosphere followed by the addition of dry $PhCF_3$ (10 mL). The resulting solution was degassed by being sparged with argon under ultrasonication for 15 minutes and irradiated at λ = 366 nm at 30 °C for 18 h. After irradiation, the volatile compounds were removed under reduced pressure and the crude reaction mixture was subjected to FCC (SiO_2, 50 → 80% EtOAc/hexanes) to yield **1a/1a-**d_1 as a white solid (4.05 mg, 22.7 µmol, 91%, 43% *ee*) (Fig. S19).

a)

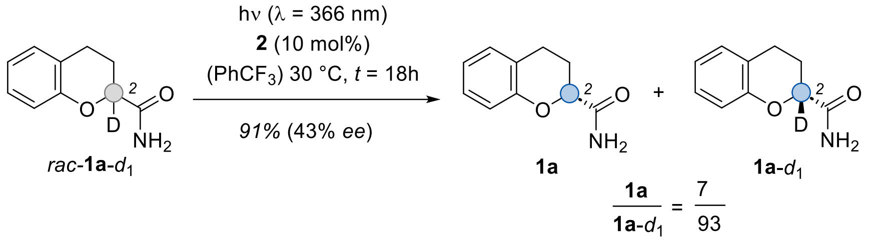

b)

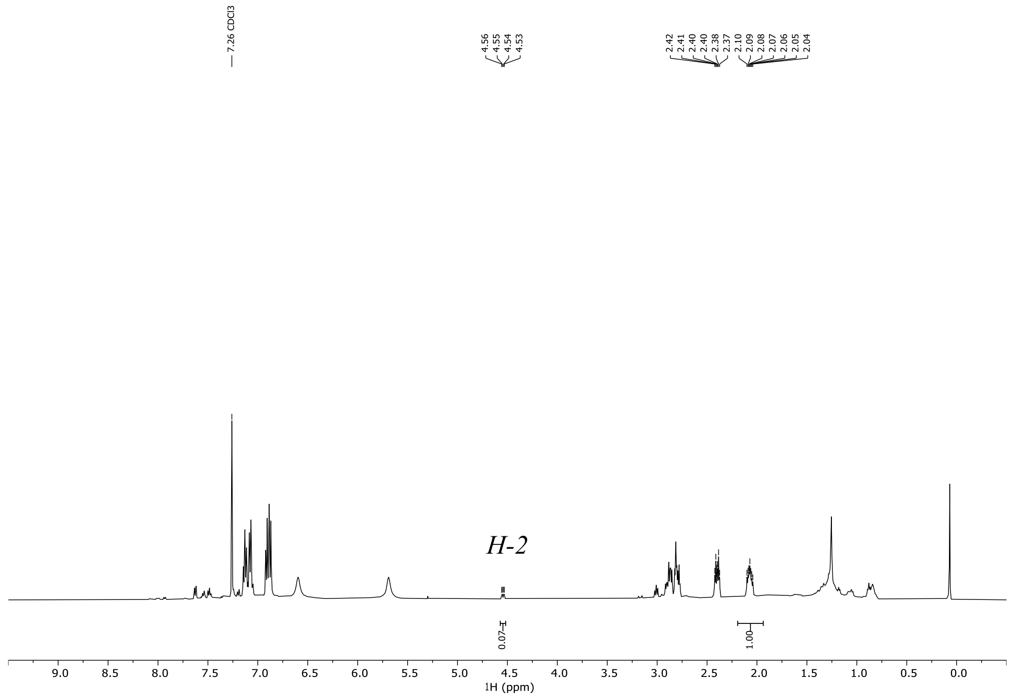

Fig. S19. a) Hydrogen incorporation experiment of *rac*-**1a**-d_1 under standard conditions but in the absence of PhSH. b) Determination of degree of deuteration in 2-position from the crude reaction mixture by ^{1}H-NMR. The NMR spectrum was recorded in $CDCl_3$ on an AVHD500 Bruker NMR.

S9. Synthesis and Characterization of Deracemization Substrates

6-Ethyl-4-oxo-4*H*-chromene-2-carboxylic acid ethyl ester (S-1i)

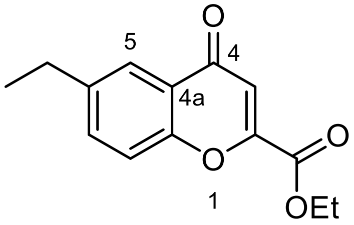

S-1i

$C_{14}H_{14}O_4$

MW = 246.26 gmol^{-1}

According to GP A, a mixture of 5-ethyl-2-hydroxy acetophenone (600 mg, 556 µL, 3.65 mmol, 1.00 equiv.) and diethyl oxalate (1.07 g, 989 µL, 7.31 mmol, 2.00 equiv.) was added dropwise to a suspension of NaH (60 wt%, 365 mg, 9.13 mmol, 2.50 equiv.) in Et_2O (3.7 mL) at 0 °C. Then, the resulting suspension was allowed to warm to r.t. and stirred for 14 h, before the reaction mixture was poured on an ice/water mixture. The pH value was adjusted to 1 by addition of aqueous HCl (6.0 M) and the resulting suspension was extracted thrice with Et_2O (3 × 20 mL). The organic layers were combined and the solvents were removed under reduced pressure. The obtained solids were then suspended in aqueous HCl (6.0 M, 1.07 g, 4.87 mL, 29.2 mmol, 8.00 equiv.) and the resulting suspension was stirred at r.t. for 14 h. Water was added and the reaction mixture was extracted thrice with Et_2O (3 × 20 mL). The combined organic layers were then washed with brine and dried over Na_2SO_4 before the remaining solvents were removed under reduced pressure. The obtained crude product was then subjected to FCC (SiO_2, 10 → 20% EtOAc/hexanes) to yield 6-ethyl-4-oxo-4*H*-chromene-2-carboxylic acid ethyl ester (**S-1i**) (453 mg, 1.84 mmol, 50%) as a white solid.

TLC (10% EtOAc/hexanes): R_f = 0.38 [UV, $KMnO_4$].

M.p.: 52 °C

^{1}H-NMR (400 MHz, $CDCl_3$, 300 K): δ [ppm] = 8.01 (d, ^4J = 2.2 Hz, 1H, H5), 7.58 (dd, ^3J = 8.7 Hz, ^4J = 2.2 Hz 1H, H7), 7.53 (d, ^3J = 8.7 Hz, 1H, H8), 7.10 (s, 1H, H3), 4.46 (q, ^3J = 7.1 Hz, 2H, OCH_2), 2.77 (q, ^3J = 7.6 Hz, 2H, $C6CH_2$), 1.43 (t, ^3J = 7.1 Hz, 3H, $OCH_2C\mathit{H}_3$), 1.29 (t, ^3J = 7.6 Hz, 3H, $C6CH_2C\mathit{H}_3$).

^{13}C-NMR (101 MHz, $CDCl_3$, 300 K): δ [ppm] = 178.7 (C4), 160.8 (COOEt), 154.6 (C8a), 152.3 (C2), 142.5 (C6), 135.1 (C7), 124.4 (C4a), 124.0 (C5), 118.8 (C8), 114.8 (C3), 63.1 (OCH_2), 28.5 ($C6\mathit{C}H_2$), 15.5 ($C6CH_2\mathit{C}H_3$), 14.2 ($OCH_2\mathit{C}H_3$).

HRMS (ESI) *m/z* $[M+H]^+$ calculated for $[C_{14}H_{15}O_4]^+$: 247.0965; found: 247.0962.

IR (film) $\tilde{\nu}_{max}$/cm^{-1} = 2969 (w, CH_{arom}), 2935 (w, CH_{aliph}), 2875 (w, CH_{aliph}), 1745 (m, $C{=}O_{ester}$), 1659 (s, $C{=}O_{ketone}$), 1622 (m, C=C), 1484 (m, CH_{arom}), 1450 (m, CH_{aliph}), 1260 (s, C–O), 1238 (s, C–O).

5-Methyl-4-oxo-4*H*-chromene-2-carboxylic acid ethyl ester (S-1j)

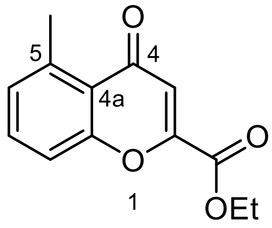

S-1j
$C_{13}H_{12}O_4$
MW = 232.24 $gmol^{-1}$

According to GP A, a mixture of 6-methyl-2-hydroxy acetophenone (610 mg, 4.06 mmol, 1.00 equiv.) and diethyl oxalate (1.19 g, 1.10 mL, 8.12 mmol, 2.00 equiv.) was added dropwise to a suspension of NaH (60 wt%, 406 mg, 10.2 mmol, 2.50 equiv.) in Et_2O (4.1 mL) at 0 °C. Then, the resulting suspension was allowed to warm to r.t. and stirred for 14 h, before the reaction mixture was poured on an ice/water mixture. The pH value was adjusted to 1 by addition of aqueous HCl (6.0 M) and the resulting suspension was extracted thrice with Et_2O (3 × 20 mL). The organic layers were combined and the solvents were removed under reduced pressure. The obtained solids were then suspended in aqueous HCl (6.0 M, 1.18 g, 5.42 mL, 32.5 mmol, 8.00 equiv.) and the resulting suspension was stirred at r.t. for 14 h. Water was added and the reaction mixture was extracted thrice with Et_2O (3 × 20 mL). The combined organic layers were then washed with brine and dried over Na_2SO_4 before the remaining solvents were removed under reduced pressure. The obtained crude product was then subjected to FCC (SiO_2, 5 → 10% EtOAc/hexanes) to yield 5-methyl-4-oxo-4*H*-chromene-2-carboxylic acid ethyl ester (**S-1j**) (622 mg, 2.68 mmol, 66%) as a white solid.

TLC (10% EtOAc/hexanes): $R_f = 0.54$ [UV, $KMnO_4$].

M.p.: 77 °C

^{1}H-NMR (400 MHz, $CDCl_3$, 300 K): δ [ppm] = 7.54 (dd, $^3J = 8.5$ Hz, $^3J = 7.4$ Hz, 1H, H7), 7.43 – 7.40 (m, 1H, H8), 7.17 – 7.14 (m, 1H, H6), 6.99 (s, 1H, H3), 4.45 (q, $^3J = 7.1$ Hz, 2H, CH_2), 2.84 (s, 3H, $C5CH_3$), 1.42 (t, $^3J = 7.1$ Hz, 3H, CH_2CH_3).

^{13}C-NMR (101 MHz, $CDCl_3$, 300 K): δ [ppm] = 180.5 (C4), 160.8 (COOEt), 157.7 (C8a), 150.9 (C2), 141.3 (C5), 133.7 (C7), 128.4 (C6), 123.1 (C4a), 116.8 (C8), 116.2 (C3), 63.0 (OCH_2), 22.7 ($C5CH_3$), 14.2 (CH_2CH_3).

HRMS (ESI) *m/z* $[M+H]^+$ calculated for $[C_{13}H_{13}O_4]^+$: 233.0808; found: 233.0801.

IR (film) $\tilde{\nu}_{max}/cm^{-1}$ = 3070 (w, CH_{arom}), 2981 (w, CH_{arom}), 2925 (w, CH_{aliph}), 2871 (w, CH_{aliph}), 1743 (s, $C{=}O_{ester}$), 1640 (s, $C{=}O_{ketone}$), 1624 (m, C=C), 1475 (m, CH_{arom}), 1455 (m, CH_{aliph}), 1251 (s, C–O), 1214 (s, C–O).

7-Methyl-4-oxo-4*H*-chromene-2-carboxylic acid ethyl ester (S-1k)

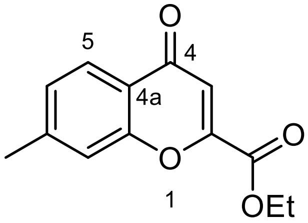

S-1k
$C_{13}H_{12}O_4$
MW = 232.24 gmol^{-1}

According to GP A, a mixture of 4-methyl-2-hydroxy acetophenone (600 mg, 556 µL, 4.00 mmol, 1.00 equiv.) and diethyl oxalate (1.17 g, 1.08 mL, 7.99 mmol, 2.00 equiv.) was added dropwise to a suspension of NaH (60 wt%, 399 mg, 9.99 mmol, 2.50 equiv.) in Et_2O (4.0 mL) at 0 °C. Then, the resulting suspension was allowed to warm to r.t. and stirred for 14 h, before the reaction mixture was poured on an ice/water mixture. The pH value was adjusted to 1 by addition of aqueous HCl (6.0 M) and the resulting suspension was extracted thrice with Et_2O (3 × 20 mL). The organic layers were combined and the solvents were removed under reduced pressure. The obtained solids were then suspended in aqueous HCl (6.0 M, 1.17 g, 5.33 mL, 32.0 mmol, 8.00 equiv.) and the resulting suspension was stirred at r.t. for 14 h. Water was added and the reaction mixture was extracted thrice with Et_2O (3 × 20 mL). The combined organic layers were then washed with brine and dried over Na_2SO_4 before the remaining solvents were removed under reduced pressure. The obtained crude product was then subjected to FCC (SiO_2, 10 → 20% EtOAc/hexanes) to yield 7-methyl-4-oxo-4*H*-chromene-2-carboxylic acid ethyl ester (**S-1k**) (521 mg, 2.24 mmol, 56%) as a white solid.

TLC (10% EtOAc/hexanes): $R_f = 0.35$ [UV, $KMnO_4$].

^{1}H-NMR (400 MHz, $CDCl_3$, 300 K): δ [ppm] = 8.06 (d, $^3J = 8.1$ Hz, 1H, H5), 7.40 – 7.40 (m, 1H, H8), 7.24 (dd, $^3J = 8.1$ Hz, $^4J = 1.6$ Hz, 1H, H6), 7.07 (s, 1H, H3), 4.45 (q, $^3J = 7.1$ Hz, 2H, CH_2CH$_3$), 2.49 (s, 3H, C7CH$_3$), 1.42 (t, $^3J = 7.1$ Hz, 3H, CH$_2$CH_3).

^{13}C-NMR (101 MHz, $CDCl_3$, 300 K): δ [ppm] = 178.4 (C4), 160.8 (COOEt), 156.3 (C8a), 152.2 (C2), 146.4 (C7), 127.6 (C6), 125.6 (C5), 122.4 (C4a), 118.6 (C8), 114.9 (C3), 63.0 (*C*H$_2$CH$_3$), 22.0 (C6*C*H$_3$), 14.2 (CH$_2$*C*H$_3$).

The recorded spectroscopic data match the reported values.[95]

8-Methyl-4-oxo-4*H*-chromene-2-carboxylic acid ethyl ester (S-1l)

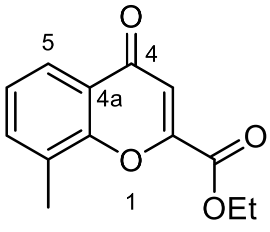

S-1l
$C_{13}H_{12}O_4$
MW = 232.24 gmol^{-1}

Following a procedure by, diethyl oxalate (934 mg, 865 µL, 6.39 mmol, 2.40 equiv.) was added dropwise to a mixture of 3-methyl-2-hydroxy acetophenone (400 mg, 2.66 mmol, 1.00 equiv.) and a suspension of NaH (60 wt%, 266 mg, 6.66 mmol, 2.50 equiv.) in Et_2O (7.5 mL) at 0 °C. Then, the resulting suspension was allowed to warm to r.t. and stirred for 38 h, before the reaction mixture was poured on an ice/water mixture. The pH value was adjusted to 1 by addition of aqueous HCl (5.0 M) and the resulting suspension was extracted thrice with Et_2O (3 × 20 mL). The organic layers were combined and the solvents were removed under reduced pressure. The obtained solids were then suspended in aqueous HCl (5.0 M, 1.85 g, 10.1 mL, 50.6 mmol, 19.0 equiv.) and the resulting suspension was stirred at r.t. for 38 h. Water was added and the reaction mixture was extracted thrice with Et_2O (3 × 20 mL). The combined organic layers were then washed with brine and dried over Na_2SO_4 before the remaining solvents were removed under reduced pressure. The obtained crude product was then washed thrice with cold Et_2O (3 × 5 mL) to yield 8-methyl-4-oxo-4*H*-chromene-2-carboxylic acid ethyl ester **(S-1l)** (560 mg, 2.41 mmol, 91%) as an off-white solid.

M.p.: 86 °C

^{1}H-NMR (400 MHz, $CDCl_3$, 300 K): δ [ppm] = 8.04 (dd, ^3J = 7.9 Hz, ^4J = 1.3 Hz, 1H, H5), 7.59 – 7.57 (m, 1H, H7), 7.34 (*virt.* t, $^3J \approx {}^3J$ = 7.6 Hz, 1H, H6), 7.13 (s, 1H, H3), 4.47 (q, ^3J = 7.1 Hz, 2H, CH_2CH$_3$), 2.56 (s, 3H, C8CH$_3$), 1.44 (t, ^3J = 7.1 Hz, 3H, CH$_2$CH_3).

^{13}C-NMR (101 MHz, $CDCl_3$, 300 K): δ [ppm] = 160.8 (COOEt), 154.7 (C8a), 152.3 (C2), 135.8 (C7), 128.6 (C4), 125.6 (C6), 123.5 (C5), 114.6 (C3), 63.0 (*C*H$_2$CH$_3$), 15.7 (C8*C*H$_3$), 14.2 (CH$_2$*C*H$_3$).

HRMS (ESI) *m/z* $[M+H]^+$ calculated for $[C_{13}H_{13}O_4]^+$: 233.0808; found: 273.0798.

IR (film) $\tilde{\nu}_{max}/cm^{-1}$ = 3092 (w, CH_{arom}), 2988 (w, CH_{arom}), 2959 (w, CH_{aliph}), 2925 (w, CH_{aliph}), 2855 (w, CH_{aliph}), 1739 (s, $C{=}O_{ester}$), 1652 (s, $C{=}O_{ketone}$), 1622 (s, C=C), 1483 (m, CH_{arom}), 1454 (m, CH_{aliph}), 1292 (s, C–O), 1244 (s, C–O).

The signal for C4 cannot be detected in the ^{13}C-NMR spectrum.

6,7,8,9-Tetrahydro-4-oxo-4*H*-benzo[*g*]chromene-2-carboxylic acid ethyl ester (S-1m)

According to GP A, a mixture of 1-(3-hydroxy-5,6,7,8-tetrahydronaphthalen-2-yl)ethan-1-one (600 mg, 3.15 mmol, 1.00 equiv.) and diethyl oxalate (922 mg, 853 µL, 6.31 mmol, 2.00 equiv.) was added dropwise to a suspension of NaH (60 wt%, 315 mg, 7.88 mmol, 2.50 equiv.) in Et_2O (3.2 mL) at 0 °C. Then, the resulting suspension was allowed to warm to r.t. and stirred for 14 h, before the reaction mixture was poured on an ice/water mixture. The pH value was adjusted to 1 by addition of aqueous HCl (6.0 M) and the resulting suspension was extracted thrice with Et_2O (3 × 20 mL). The organic layers were combined and the solvents were removed under reduced pressure. The obtained solids were then suspended in aqueous HCl (6.0 M, 920 mg, 4.21 mL, 25.2 mmol, 8.00 equiv.) and the resulting suspension was stirred at r.t. for 14 h. Water was added and the reaction mixture was extracted thrice with Et_2O (3 × 20 mL). The combined organic layers were then washed with brine and dried over Na_2SO_4 before the remaining solvents were removed under reduced pressure. The obtained crude product was then subjected to FCC (SiO_2, 5 → 20% EtOAc/hexanes) to yield 6,7,8,9-tetrahydro-4-oxo-4*H*-benzo[*g*]chromene-2-carboxylic acid ethyl ester (**S-1m**) (562 mg, 2.07 mmol, 65%) as a white solid.

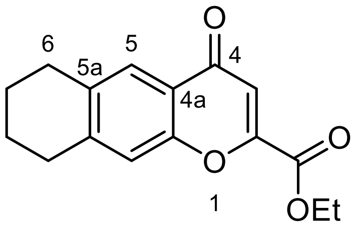

S-1m
$C_{16}H_{16}O_4$
MW = 272.30 gmol^{-1}

TLC (10% EtOAc/hexanes): R_f = 0.28 [UV, $KMnO_4$].

M.p.: 80 °C

^{1}H-NMR (400 MHz, $CDCl_3$, 300 K): δ [ppm] = 7.87 (s, 1H, H5), 7.31 (s, 1H, H8), 7.05 (s, 1H, H3), 4.45 (q, ^3J = 7.1 Hz, 2H, OCH_2), 2.92 – 2.86 (m, 4H, H6, H9), 1.85 – 1.82 (m, 4H, H7, H8), 1.42 (t, ^3J = 7.1 Hz, 3H, CH_3).

^{13}C-NMR (101 MHz, $CDCl_3$, 300 K): δ [ppm] = 178.7 (C4), 160.9 (COOEt), 154.2 (C8a), 152.1 (C2), 146.1 (C9a), 136.1 (C5a), 125.2 (C5), 122.4 (C4a), 118.1 (C8), 114.5 (C3), 63.0 (OCH_2), 30.2 (C9), 29.1 (C6), 22.9 (C7/C8), 22.6 (C7/C8), 14.2 (CH_3).

HRMS (ESI) *m/z* $[M+H]^+$ calculated for $[C_{16}H_{17}O_4]^+$: 273.1121; found: 273.1118.

IR (film) $\tilde{\nu}_{max}$/cm^{-1} = 2982 (w, CH_{arom}), 2936 (m, CH_{aliph}), 2864 (w, CH_{aliph}), 1740 (m, $C{=}O_{ester}$), 1655 (s, $C{=}O_{ketone}$), 1627 (s, C=C), 1448 (m, CH_{aliph}), 1258 (s, C–O), 1237 (s, C–O).

7-Methoxy-4-oxo-4*H*-benzo[*g*]chromene-2-carboxylic acid ethyl ester (S-1o)

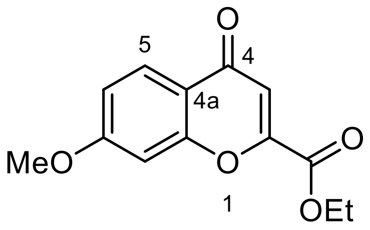

S-1o
$C_{13}H_{12}O_5$
MW = 248.23 gmol^{-1}

Following a modified procedure by Tummanapalli *et al.*,[96] KO^{t}Bu (667 mg, 5.95 mmol, 2.00 equiv.) was added in 3 portions to a solution of 2-fluoro-4-methoxyacetophenone (500 mg, 2.97 mmol, 1.00 equiv.) and diethyl oxalate (652 mg, 603 µL, 4.46 mmol, 1.50 equiv.) in DMF (20 mL) at 0 °C. The resulting dark reaction mixture was then stirred at 80 °C for 3 h. After the mixture was allowed to cool to r.t., water (30 mL) was added and the resulting mixture was extracted three times with EtOAc (3 × 20 mL). The combined organic layers were washed with a sat. aqueous LiCl solution (30 mL) and dried over Na_2SO_4. The solvents were removed under reduced pressure and the obtained crude product was subjected to FCC (SiO_2, 20 → 40% EtOAc/hexanes) to yield 7-methoxy-4-oxo-4*H*-benzo[*g*]chromene-2-carboxylic acid ethyl ester (**S-1o**) (216 mg, 870 µmol, 29%) as an off-white solid.

TLC (40% EtOAc/hexanes): R_f = 0.55 [UV, $KMnO_4$].

^{1}H-NMR (400 MHz, DMSO-d$_6$, 300 K): δ [ppm] = 7.94 (d, ^3J = 8.9 Hz, 1H, H5), 7.22 (d, ^2J = 2.4 Hz, 1H, H8), 7.10 (dd, ^3J = 8.9 Hz, ^2J = 2.4 Hz, 1H, H6), 6.88 (s, 1H, H3), 4.39 (q, ^3J = 7.1 Hz, 2H, OCH_2), 3.92 (s, 3H, OCH_3), 1.35 (t, ^3J = 7.1 Hz, 3H, CH_2CH_3).

^{13}C-NMR (101 MHz, DMSO-d$_6$, 300 K): δ [ppm] = 176.3 (C4), 164.6 (C7), 160.0 (COOEt), 157.3 (C8a), 151.9 (C2), 126.3 (C5), 117.6 (C4a), 115.7 (C6), 114.0 (C3), 101.0 (C8), 62.6 (OCH_2), 56.3 (OCH_3), 13.9 (CH_2CH_3).

The spectroscopic data matches the one reported in the literature.[96]

7-Fluoro-4-oxo-4*H*-benzo[*g*]chromene-2-carboxylic acid ethyl ester (S-1p)

Following a modified procedure by Tummanapalli *et al.*,[96] KO^{t}Bu (666 mg, 5.93 mmol, 2.00 equiv.) was added in 3 portions to a solution of 2,4-difluoroacetophenone (463 mg, 2.97 mmol, 1.00 equiv.) and diethyl oxalate (650 mg, 602 µL, 4.45 mmol, 1.50 equiv.) in DMF (20 mL) at 0 °C. The resulting dark reaction mixture was then stirred at 80 °C for 3 h. After the mixture was allowed to cool to r.t., water (30 mL) was added and the resulting mixture was extracted three times with EtOAc (3 × 20 mL). The combined organic layers were washed with a sat. aqueous LiCl solution (30 mL) and dried over Na_2SO_4. The solvents were removed under reduced pressure and the obtained crude product was subjected to FCC (SiO_2, 5 → 40% EtOAc/hexanes) to yield 7-fluoro-4-oxo-4*H*-benzo[*g*]chromene-2-carboxylic acid ethyl ester (**S-1p**) (610 mg, 2.38 mmol, 80%) as a brown solid.

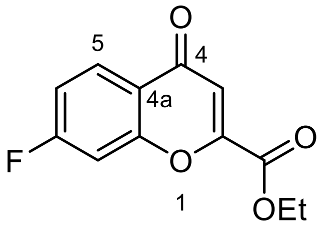

S-1p

$C_{12}H_9FO_4$

MW = 236.20 gmol^{-1}

TLC (40% EtOAc/hexanes): R_f = 0.75 [UV, $KMnO_4$].

^{1}H-NMR (400 MHz, $CDCl_3$, 300 K): δ [ppm] = 8.22 (dd, $^3J_{HH}$ = 8.9 Hz, $^4J_{HF}$ = 6.2 Hz, 1H, H5), 7.30 (dd, $^3J_{HF}$ = 8.9 Hz, $^3J_{HH}$ = 2.4 Hz, 1H, H8), 7.18 (ddd, $^3J_{HH}$ = 8.9 Hz, $^3J_{HF}$ = 8.0 Hz, $^4J_{HH}$ = 2.4 Hz, 1H, H6), 7.10 (s, 1H, H3), 4.46 (q, ^3J = 7.2 Hz, 2H, CH_2), 1.43 (t, ^3J = 7.2 Hz, 3H, CH_3).

^{13}C-NMR (101 MHz, $CDCl_3$, 300 K): δ [ppm] = 177.5 (C4), 166.4 (d, $^1J_{CF}$ = 257 Hz, C7), 160.4 (COOEt), 157.2 (d, $^3J_{CF}$ = 13.6 Hz, C8a), 152.7 (d, $^5J_{CF}$ = 1.2 Hz, C2), 128.5 (d, $^xJ_{CF}$ = 10.7 Hz, C5), 121.5 (d, $^4J_{CF}$ = 2.2 Hz, C4a), 115.1 (C3), 115.0 (d, $^2J_{CF}$ = 23.0 Hz, C6), 105.6 (d, $^2J_{CF}$ = 25.6 Hz, C8), 63.3 (CH_2), 14.2 (CH_3).

^{19}F-NMR (376 MHz, $CDCl_3$, 300 K): δ [ppm] = −100.7 – −100.8 (m).

The spectroscopic data matches the one reported in the literature.[96]

8-Fluoro-4-oxo-4*H*-chromene-2-carboxylic acid ethyl ester (S-1q)

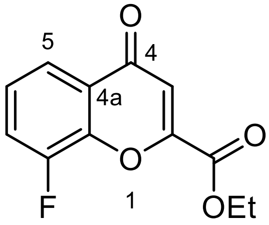

S-1q
$C_{12}H_9FO_4$
MW = 236.20 gmol^{-1}

According to GP A, a mixture of 3-fluoro-2-hydroxy acetophenone (600 mg, 3.89 mmol, 1.00 equiv.) and diethyl oxalate (1.14 g, 1.05 mL, 7.79 mmol, 2.00 equiv.) was added dropwise to a suspension of NaH (60 wt%, 389 mg, 9.73 mmol, 2.50 equiv.) in Et_2O (3.9 mL) at 0 °C. Then, the resulting suspension was allowed to warm to r.t. and stirred for 14 h, before the reaction mixture was poured on an ice/water mixture. The pH value was adjusted to 1 by addition of aqueous HCl (6.0 M) and the resulting suspension was extracted thrice with Et_2O (3 × 20 mL). The organic layers were combined and the solvents were removed under reduced pressure. The obtained solids were then suspended in aqueous HCl (6.0 M, 1.14 g, 5.19 mL, 31.1 mmol, 8.00 equiv.) and the resulting suspension was stirred at r.t. for 14 h. Water was added and the reaction mixture was extracted thrice with Et_2O (3 × 20 mL). The combined organic layers were then washed with brine and dried over Na_2SO_4 before the remaining solvents were removed under reduced pressure. The obtained crude product was then subjected to FCC (SiO_2, 10 → 20% EtOAc/hexanes) to yield 8-fluoro-4-oxo-4*H*-chromene-2-carboxylic acid ethyl ester (**S-1q**) (630 mg, 2.67 mmol, 69%) as a white solid.

TLC (20% EtOAc/hexanes): R_f = 0.58 [UV, $KMnO_4$].

M.p.: 116 °C (decomposition)

^{1}H-NMR (400 MHz, $CDCl_3$, 300 K): δ [ppm] = 7.96 (*virt.* dt, ^3J = 8.1 Hz, $^4J_{HH} \approx {}^5J_{HF}$ = 1.5 Hz, 1H, H5), 7.51 (ddd, $^3J_{HF}$ = 10.0 Hz, ^3J = 8.1 Hz, ^4J = 1.5 Hz, 1H, H7), 7.39 (td, $^3J \approx {}^3J$ = 8.1 Hz, $^4J_{HF}$ = 4.4 Hz, 1H, H6), 7.14 (s, 1H, H3), 4.48 (q, ^3J = 7.1 Hz, 2H, CH_2), 1.44 (t, ^3J = 7.1 Hz, 3H, CH_3).

^{13}C-NMR (101 MHz, $CDCl_3$, 300 K): δ [ppm] = 177.6 (d, $^4J_{CF}$ = 2.7 Hz, C4), 160.2 (COOEt), 152.3 (C2), 151.9 (d, $^1J_{CF}$ = 255 Hz, C8), 145.0 (d, $^2J_{CF}$ = 11.5 Hz, C8a), 126.4 (C4a), 125.7 (d, $^3J_{CF}$ = 6.5 Hz, C6), 121.0 (d, $^4J_{CF}$ = 4.2 Hz, C5), 120.6 (d, $^2J_{CF}$ = 16.7 Hz, C7), 115.1 (C3), 63.3 (CH_2), 14.2 (CH_3).

^{19}F-NMR (376 MHz, $CDCl_3$, 300 K): δ [ppm] = −131.4 – −131.4 (m).

HRMS (ESI) *m/z* $[M+H]^+$ calculated for $[C_{12}H_{10}FO_4]^+$: 237.0558; found: 237.0554.

IR (film) $\tilde{\nu}_{max}/cm^{-1}$ = 3101 (w, CH_{arom}), 3029 (w, CH_{arom}), 2996 (w, CH_{arom}), 2922 (m, CH_{aliph}), 2852 (w, CH_{aliph}), 1741 (m, $C{=}O_{ester}$), 1657 (s, $C{=}O_{ketone}$), 1627 (m, C=C), 1493 (m, CH_{arom}), 1459 (m, CH_{aliph}), 1277 (s, C–O), 1256 (s, C–O), 1144 (m, C–F).

6-Ethylchromane-2-carboxylic acid ethyl ester (*rac*-**S-2i**)

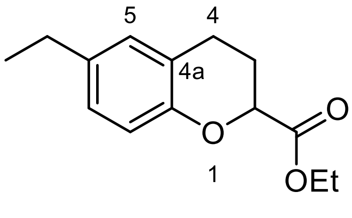

rac-**S-2i**
$C_{14}H_{18}O_3$
MW = 234.30 gmol^{-1}

According to GP B, a suspension of 6-ethyl-4-oxo-4*H*-chromene-2-carboxylic acid ethyl ester (**S-1i**) (403 mg, 1.63 mmol, 1.00 equiv.) and Pd/C (10 wt%, 87.0 mg, 81.7 µmol, 5.0 mol%) in EtOH (9.1 mL) and conc. AcOH (18 M, 1.10 g, 1.02 mL, 18.3 mmol, 11.2 equiv.) was degassed by freeze-pump-thaw cycling (2×) and then sparged continuously sparged with H_2 (1 atm) and stirred at r.t. for 38 h. After that, the solid compounds were filtered over celite and washed with copious amounts with EtOAc. The filtrate was then concentrated under reduced pressure and the obtained crude product was subjected to FCC (SiO_2, 2 → 5% EtOAc/hexanes) to afford 6-ethylchromane-2-carboxylic acid ethyl ester (*rac*-**S-2i**) as a colorless oil (359 mg, 1.53 mmol, 94%).

TLC (2% EtOAc/hexanes): R_f = 0.23 [UV, $KMnO_4$].

^{1}H-NMR (400 MHz, $CDCl_3$, 300 K): δ [ppm] = 6.95 (dd, ^3J = 8.4 Hz, ^4J = 2.2 Hz, 1H, H7), 6.87 – 6.85 (m, 2H, H6, H8), 4.68 (dd, ^3J = 7.7 Hz, ^3J = 3.5 Hz, 1H, H2), 4.26 (q, ^3J = 7.1 Hz, 2H, OCH_2), 2.86 – 2.70 (m, 2H, H4), 2.55 (q, ^3J = 7.6 Hz, 2H, C6CH_2CH$_3$), 2.27 (*virt.* dtd, ^2J = 13.6 Hz, $^3J \approx {}^3J$ = 6.2 Hz, ^3J = 3.5 Hz, 1H, H3^a), 2.17 (*virt.* dtd, ^2J = 13.6 Hz, $^3J \approx {}^3J$ = 7.9 Hz, ^3J = 5.7 Hz, 1H, H3^b), 1.30 (q, ^3J = 7.1 Hz, 3H, OCH$_2$CH_3), 1.20 (t, ^3J = 7.6 Hz, 3H, C6CH$_2$CH_3).

^{13}C-NMR (101 MHz, $CDCl_3$, 300 K): δ [ppm] = 171.1 (COOEt), 151.6 (C8a), 136.6 (C6), 128.7 (C5), 127.1 (C7), 121.0 (C4a), 116.9 (C8), 73.9 (C2), 61.4 (OCH_2), 28.1 (C6CH$_2$CH$_3$), 24.9 (C3), 23.6 (C4), 15.9 (C6CH$_2$$CH_3$), 14.3 (OCH$_2$$CH_3$).

HRMS (ESI) *m/z* [M+H]$^+$ calculated for $[C_{14}H_{19}O_3]^+$: 235.1329; found: 235.1322.

IR (film) $\tilde{\nu}_{max}$/cm^{-1} = 2965 (m, CH$_{arom}$), 2933 (m, CH$_{aliph}$), 2873 (w, CH$_{aliph}$), 1754 (s, C=O), 1734 (s, C=O), 1498 (s, CH$_{arom}$), 1200 (s, C–O), 1128 (m, C–O).

5-Methylchromane-2-carboxylic acid ethyl ester (*rac*-**S-2j**)

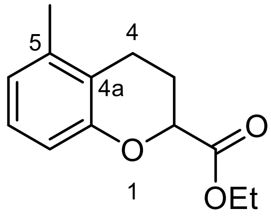

rac-**S-2j**
$C_{13}H_{16}O_3$
MW = 220.27 gmol^{-1}

According to GP B, a suspension of 5-methyl-4-oxo-4*H*-chromene-2-carboxylic acid ethyl ester (**S-1j**) (230 mg, 990 µmol, 1.00 equiv.) and Pd/C (10 wt%, 52.7 mg, 49.5 µmol, 5.0 mol%) in EtOH (5.5 mL) and conc. AcOH (18 M, 666 mg, 616 µL, 11.1 mmol, 11.2 equiv.) was degassed by freeze-pump-thaw cycling (2×) and then sparged continuously sparged with H_2 (1 atm) and stirred at r.t. for 38 h. After that, the solid compounds were filtered over celite and washed with copious amounts with EtOAc. The filtrate was then concentrated under reduced pressure and the obtained crude product was subjected to FCC (SiO_2, 5 → 10% EtOAc/hexanes) to afford 5-methylchromane-2-carboxylic acid ethyl ester (*rac*-**S-2j**) as a colorless oil (184 mg, 835 µmol, 84%).

TLC (5% EtOAc/hexanes): R_f = 0.36 [UV, $KMnO_4$].

^{1}H-NMR (400 MHz, $CDCl_3$, 300 K): δ [ppm] = 7.05 –7.01 (m, 1H, H7), 6.82 –6.80 (m, 1H, H8), 6.77 –6.75 (m, 1H, H6), 4.65 (dd, ^3J = 7.9 Hz, ^3J = 3.4 Hz, 1H, H2), 4.26 (q, ^3J = 7.1 Hz, 2H, OCH_2), 2.75 – 2.60 (m, 2H, H4), 2.32 (*virt.* dtd, ^2J = 13.7 Hz, $^3J \approx {}^3J$ = 6.3 Hz, ^3J = 3.4 Hz, 1H, H3^a), 2.25 – 2.15 (m, 4H, H3^b, C5CH_3), 1.30 (t, ^3J = 7.1 Hz, 3H, OCH_2CH_3).

^{13}C-NMR (101 MHz, $CDCl_3$, 300 K): δ [ppm] = 171.1 (COOEt), 153.7 (C8a), 137.3 (C5), 127.0 (C7), 122.4 (C6), 120.2 (C4a), 115.0 (C8), 73.5 (C2), 61.4 (OCH_2), 24.9 (C3), 21.2 (C4), 19.1 (C5CH_3), 14.3 (OCH_2CH_3).

HRMS (ESI) *m/z* $[M+H]^+$ calculated for $[C_{13}H_{17}O_3]^+$: 221.1172; found: 221.1166.

IR (film) $\tilde{\nu}_{max}/cm^{-1}$ = 2979 (w, CH_{arom}), 2936 (m, CH_{aliph}), 2855 (w, CH_{aliph}), 1753 (s, C=O), 1734 (s, C=O), 1585 (m, C=C), 1468 (s, CH_{arom}), 1446 (m, CH_{aliph}), 1191 (s, C–O), 1174 (s, C–O), 1110 (s, C–O).

7-Methylchromane-2-carboxylic acid ethyl ester (*rac*-**S-2k**)

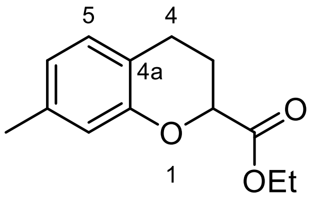

rac-**S-2k**
$C_{13}H_{16}O_3$
MW = 220.27 gmol^{-1}

According to GP B, a suspension of 7-methoxy-4-oxo-4*H*-chromene-2-carboxylic acid ethyl ester (**S**-1k) (403 mg, 1.74 mmol, 1.00 equiv.) and Pd/C (10 wt%, 92.3 mg, 86.8 µmol, 5.0 mol%) in EtOH (9.6 mL) and conc. AcOH (18 M, 1.17 g, 1.08 mL, 19.4 mmol, 11.2 equiv.) was degassed by freeze-pump-thaw cycling (2×) and then sparged continuously sparged with H_2 (1 atm) and stirred at r.t. for 38 h. After that, the solid compounds were filtered over celite and washed with copious amounts with EtOAc. The filtrate was then concentrated under reduced pressure and the obtained crude product was subjected to FCC (SiO_2, 5 → 10% EtOAc/hexanes) to afford 7-methylchromane-2-carboxylic acid ethyl ester (*rac*-**S-2k**) as a colorless oil (302 mg, 1.97 mmol, 79%).

TLC (5% EtOAc/hexanes): R_f = 0.36 [UV, $KMnO_4$].

^{1}H-NMR (400 MHz, $CDCl_3$, 300 K): δ [ppm] = 6.91 (d, ^3J = 7.7 Hz, 1H, H5), 6.76 (d, ^4J = 1.2 Hz, 1H, H8), 6.68 (dd, ^3J = 7.7 Hz, ^4J = 1.2 Hz, 1H, H6), 4.69 (dd, ^3J = 7.4 Hz, ^3J = 3.6 Hz, 1H, H2), 4.25 (q, ^3J = 7.1 Hz, 2H, CH_2CH_3), 2.83 – 2.67 (m, 2H, H4), 2.32 – 2.12 (m, 5H, H3, C7CH_3), 1.21 (t, ^3J = 7.1 Hz, 3H, CH2CH_3).

^{13}C-NMR (101 MHz, $CDCl_3$, 300 K): δ [ppm] = 171.1 (COOEt), 153.4 (C8a), 137.6 (C7), 129.3 (C5), 121.9 (C6), 118.3 (C4a), 117.5 (C8), 73.9 (C2), 61.5 (OCH_2), 24.9 (C3), 23.1 (C4), 21.2 (C7CH_3), 14.3 (OCH_2CH_3).

HRMS (ESI) *m/z* $[M+H]^+$ calculated for $[C_{13}H_{17}O_3]^+$: 221.1172; found: 221.1170.

IR (film) $\tilde{\nu}_{max}$/cm^{-1} = 2981 (m, CH_{arom}), 2933 (m, CH_{aliph}), 1754 (s, C=O), 1735 (m, C=O), 1625 (m, C=C), 1507 (m, CH_{arom}), 1195 (m, C–O), 1150 (s, C–O), 1127 (s, C–O).

8-Methylchromane-2-carboxylic acid ethyl ester (*rac*-**S-2l**)

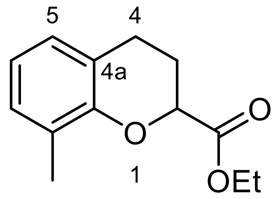

rac-**S-2l**
$C_{13}H_{16}O_3$
MW = 220.27 gmol^{-1}

According to GP B, a suspension of 8-methyl-4-oxo-4*H*-chromene-2-carboxylic acid ethyl ester (**S-1l**) (251 mg, 1.08 mmol, 1.00 equiv.) and Pd/C (10 wt%, 57.4 mg, 54.0 µmol, 5.0 mol%) in EtOH (6.0 mL) and conc. AcOH (18 M, 726 mg, 671 µL, 12.1 mmol, 11.2 equiv.) was degassed by freeze-pump-thaw cycling (2×) and then sparged continuously sparged with H_2 (1 atm) and stirred at r.t. for 38 h. After that, the solid compounds were filtered over celite and washed with copious amounts with EtOAc. The filtrate was then concentrated under reduced pressure and the obtained crude product was subjected to FCC (SiO_2, 5 → 10% EtOAc/hexanes) to afford 8-methylchromane-2-carboxylic acid ethyl ester (*rac*-**S-2l**) as a colorless oil (219 mg, 995 µmol, 92%).

TLC (10% EtOAc/hexanes): R_f = 0.83 [UV, $KMnO_4$].

^{1}H-NMR (400 MHz, $CDCl_3$, 300 K): δ [ppm] = 6.98 (dd, ^3J = 7.5 Hz, ^4J = 1.0 Hz, 1H, H7), 6.87 (dd, ^3J = 7.5 Hz, ^4J = 1.0 Hz, 1H, H5), 6.76 (*virt.* t, $^3J \approx {}^3J$ = 7.4 Hz, 1H, H6), 4.75 (dd, ^3J = 7.3 Hz, ^3J = 3.8 Hz, 1H, H2), 4.24 (q, ^3J = 7.1 Hz, 2H, OCH_2), 2.87 – 2.71 (m, 2H, H4), 2.31 – 2.14 (m, 5H, H3, C8CH_3), 1.29 (t, ^3J = 7.1 Hz, 3H, OCH_2CH_3).

^{13}C-NMR (101 MHz, $CDCl_3$, 300 K): δ [ppm] = 171.2 (COOEt), 151.8 (C8a), 128.9 (C7), 127.0 (C5), 126.3 (C8), 120.9 (C4a), 120.2 (C6), 74.0 (C2), 61.3 (OCH_2), 24.8 (C3), 23.5 (C4), 16.4 (C8CH_3), 14.3 (OCH_2CH_3).

HRMS (ESI) *m/z* $[M+H]^+$ calculated for $[C_{13}H_{17}O_3]^+$: 221.1172; found: 221.1174.

IR (film) $\tilde{\nu}_{max}$/cm^{-1} = 2926 (m, CH_{arom}), 2853 (w, CH_{aliph}), 1755 (m, C=O), 1734 (m, C=O), 1595 (w, C=C), 1469 (m, CH_{arom}), 1445 (w, CH_{aliph}), 1186 (s, C–O), 1106 (s, C–O).

6,7,8,9-Tetrahydro-benzo[*g*]chromane-2-carboxylic acid ethyl ester (*rac*-S-2m)

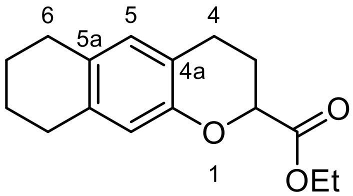

rac-**S-2m**
$C_{16}H_{20}O_3$
MW = 260.33 gmol^{-1}

According to GP B, a suspension of 6,7,8,9-tetrahydro-4-oxo-4*H*-benzo[*g*]chromene-2-carboxylic acid ethyl ester (**S-1m**) (250 mg, 918 µmol, 1.00 equiv.) and Pd/C (10 wt%, 48.9 mg, 45.9 µmol, 5.0 mol%) in EtOH (5.1 mL) and conc. AcOH (18 M, 617 mg, 571 µL, 10.3 mmol, 11.2 equiv.) was degassed by freeze-pump-thaw cycling (2×) and then sparged continuously sparged with H_2 (1 atm) and stirred at r.t. for 38 h. After that, the solid compounds were filtered over celite and washed with copious amounts with EtOAc. The filtrate was then concentrated under reduced pressure and the obtained crude product was subjected to FCC (SiO_2, 2 → 5% EtOAc/hexanes) to afford 8-methylchromane-2-carboxylic acid ethyl ester (*rac*-**S-2m**) as a colorless oil (208 mg, 801 µmol, 87%).

TLC (2% EtOAc/hexanes): R_f = 0.19 [UV, $KMnO_4$].

^{1}H-NMR (400 MHz, $CDCl_3$, 300 K): δ [ppm] = 6.73 (s, 1H, H5), 6.66 (s, 1H, H8), 4.66 (dd, ^3J = 7.5 Hz, ^3J = 3.6 Hz, 1H, H2), 4.25 (q, ^3J = 7.1 Hz, 2H, OCH_2), 2.81 – 2.64 (m, 6H, H4, H6, H9), 2.29 – 2.11 (m, 2H, H3), 1.77 – 1.74 (m, 4H, H7, H8), 1.30 (t, ^3J = 7.1 Hz, 3H, CH_3).

^{13}C-NMR (101 MHz, $CDCl_3$, 300 K): δ [ppm] = 171.2 (COOEt), 151.3 (C8a), 136.7 (C9a), 129.7 (C5), 129.6 (C5a), 118.7 (C4a), 116.7 (C8), 73.9 (C2), 61.4 (OCH_2), 29.3 (C6/C9), 28.7 (C6/C9), 25.1 (C3), 23.6 (C7/C8), 23.4 (C7/C8), 23.2 (C4), 14.4 (CH_3).

HRMS (ESI) *m/z* $[M+H]^+$ calculated for $[C_{16}H_{21}O_3]^+$: 261.1485; found: 261.1482.

IR (film) $\tilde{\nu}_{max}$/cm^{-1} = 2978 (w, CH_{arom}), 2926 (m, CH_{aliph}), 2855 (w, CH_{aliph}), 2840 (w, CH_{aliph}), 1754 (m, C=O), 1732 (m, C=O), 1626 (m, C=C), 1504 (m, CH_{arom}), 1426 (m, CH_{aliph}), 1194 (s, C–O), 1169 (s, C–O), 1113 (s, C–O).

7-Methoxychromane-2-carboxylic acid ethyl ester (*rac*-**S-2o**)

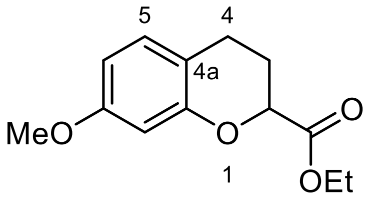

rac-**S-2o**
$C_{13}H_{16}O_4$
MW = 236.27 $gmol^{-1}$

According to GP B, a suspension of 7-methoxy-4-oxo-4*H*-chromene-2-carboxylic acid ethyl ester (**S-1o**) (180 mg, 724 µmol, 1.00 equiv.) and Pd/C (10 wt%, 38.5 mg, 36.2 µmol, 5.0 mol%) in EtOH (4.0 mL) and conc. AcOH (18 M, 487 mg, 451 µL, 8.11 mmol, 11.2 equiv.) was degassed by freeze-pump-thaw cycling (2×) and then sparged continuously sparged with H_2 (1 atm) and stirred at r.t. for 38 h. After that, the solid compounds were filtered over celite and washed with copious amounts with EtOAc. The filtrate was then concentrated under reduced pressure and the obtained crude product was subjected to FCC (SiO_2, 5 → 10% EtOAc/hexanes) to afford 7-methoxychromane-2-carboxylic acid ethyl ester (rac-**S-2o**) as a colorless oil (142 mg, 601 µmol, 83%).

TLC (5% EtOAc/hexanes): R_f = 0.38 [UV, $KMnO_4$].

^{1}H-NMR (400 MHz, $CDCl_3$, 300 K): δ [ppm] = 6.91 (d, ^3J = 8.4 Hz, 1H, H5), 6.51 (d, ^4J = 2.6 Hz, 1H, H8), 6.47 (dd, ^3J = 8.5 Hz, ^4J = 2.6 Hz, 1H, H6), 4.69 (dd, ^3J = 7.6 Hz, ^3J = 3.6 Hz, 1H, H2), 4.26 (q, ^3J = 7.1 Hz, 3H, C*H*2CH3), 3.75 (s, 3H, OCH_3), 2.81 – 2.64 (m, 2H, H4), 2.26 (dddd, ^2J = 13.7 Hz, ^3J = 6.7 Hz, ^3J = 5.9 Hz, ^3J = 3.6 Hz, 1H, $H3^a$), 2.15 (*virt.* dtd, ^2J = 13.7 Hz, $^3J \approx {}^3J$ = 7.8 Hz, ^3J = 5.6 Hz, 1H, $H3^b$), 1.29 (t, ^3J = 7.1 Hz, 3H, CH_2CH_3).

^{13}C-NMR (101 MHz, $CDCl_3$, 300 K): δ [ppm] = 171.0 (COOEt), 159.4 (C7), 154.3 (C8a), 130.0 (C5), 113.4 (C4a), 108.1 (C6), 101.8 (C8), 74.0 (C3), 61.5 (OCH_2), 55.4 (OCH_3), 25.0 (C3), 22.8 (C4), 14.3 (CH_2CH_3).

The spectroscopic data matches the one reported in the literature.[97]

7-Fluorochromane-2-carboxylic acid ethyl ester (*rac*-**S-2p**)

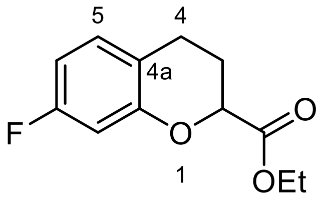

rac-**S-2p**
$C_{12}H_{13}FO_3$
MW = 224.23 gmol^{-1}

According to GP B, a suspension of 7-fluoro-4-oxo-4*H*-chromene-2-carboxylic acid ethyl ester (**S-1p**) (190 mg, 803 µmol, 1.00 equiv.) and Pd/C (10 wt%, 42.7 mg, 40.2 µmol, 5.0 mol%) in EtOH (4.5 mL) and conc. AcOH (18 M, 540 mg, 500 µL, 9.00 mmol, 11.2 equiv.) was degassed by freeze-pump-thaw cycling (2×) and then sparged continuously sparged with H_2 (1 atm) and stirred at r.t. for 38 h. After that, the solid compounds were filtered over celite and washed with copious amounts with EtOAc. The filtrate was then concentrated under reduced pressure and the obtained crude product was subjected to FCC (SiO_2, 5 → 10% EtOAc/hexanes) to afford 7-fluorochromane-2-carboxylic acid ethyl ester (*rac*-**S-2p**) as a colorless oil (148 mg, 661 µmol, 82%).

TLC (5% EtOAc/hexanes): R_f = 0.48 [UV, $KMnO_4$].

^{1}H-NMR (400 MHz, $CDCl_3$, 300 K): δ [ppm] = 6.98 – 6.94 (m, 1H, H5), 6.65 (dd, $^3J_{HF}$ = 10.2 Hz, ^4J = 2.6 Hz, 1H, H8), 6.58 (*virt.* td, $^3J_{HH} \approx {}^3J_{HF}$ = 8.4 Hz, ^4J = 2.6 Hz, 1H, H6), 4.71 (dd, ^3J = 7.0 Hz, ^3J = 3.8 Hz, 1H, H2), 4.25 (q, ^3J = 7.1 Hz, 2H, CH_2CH$_3$), 2.82 – 2.66 (m, 2H, H4), 2.29 – 2.14 (m, 2H, H3), 1.29 (t, ^3J = 7.1 Hz, 3H, CH$_2$CH_3).

^{13}C-NMR (101 MHz, $CDCl_3$, 300 K): δ [ppm] = 170.7 (COOEt), 162.1 ($^1J_{CF}$ = 243 Hz, C7), 154.4 (d, $^3J_{CF}$ = 11.9 Hz, C8a), 130.2 (d, $^3J_{CF}$ = 9.5 Hz, C5), 117.1 (d, $^4J_{CF}$ = 3.2 Hz, C4a), 108.1 (d, $^2J_{CF}$ = 21.7 Hz, C6), 104.2 (d, $^2J_{CF}$ = 24.7 Hz, C8), 73.9 (C2), 61.6 (OCH_2), 24.5 (C3), 22.8 (C4), 14.3 (CH$_2$*C*H$_3$).

^{19}F-NMR (376 MHz, $CDCl_3$, 300 K): δ [ppm] = −114.8 – −114.9 (m).

HRMS (ESI) *m/z* $[M+H]^+$ calculated for $[C_{12}H_{14}FO_3]^+$: 225.0921; found: 225.0922.

IR (film) $\tilde{\nu}_{max}$/cm^{-1} = 2983 (m, CH_{arom}), 2937 (m, CH_{aliph}), 2854 (w, CH_{aliph}), 1752 (m, C=O), 1619 (m, C=C), 1503 (s, CH_{arom}), 1432 (m, CH_{aliph}), 1193 (m, C–O), 1143 (s, C–F), 1110 (m, C–O).

8-Fluorochromane-2-carboxylic acid ethyl ester (*rac*-**S-2q**)

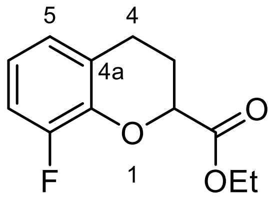

rac-**S-2q**
$C_{12}H_{13}FO_3$
MW = 224.23 gmol^{-1}

According to GP B, a suspension of 8-fluoro-4-oxo-4*H*-chromene-2-carboxylic acid ethyl ester (**S-1q**) (190 mg, 804 µmol, 1.00 equiv.) and Pd/C (10 wt%, 42.8 mg, 40.2 µmol, 5.0 mol%) in EtOH (4.5 mL) and conc. AcOH (18 M, 541 mg, 501 µL, 9.01 mmol, 11.2 equiv.) was degassed by freeze-pump-thaw cycling (2×) and then sparged continuously sparged with H_2 (1 atm) and stirred at r.t. for 38 h. After that, the solid compounds were filtered over celite and washed with copious amounts with EtOAc. The filtrate was then concentrated under reduced pressure and the obtained crude product was subjected to FCC (SiO_2, 2 → 3% EtOAc/hexanes) to afford 8-fluorochromane-2-carboxylic acid ethyl ester (*rac*-**S-2q**) as a colorless oil (157 mg, 702 µmol, 87%).

TLC (5% EtOAc/hexanes): R_f = 0.46 [UV, $KMnO_4$].

^{1}H-NMR (400 MHz, $CDCl_3$, 300 K): δ [ppm] = 6.92 (ddd, $^3J_{HF}$ = 10.9 Hz, ^3J = 6.9 Hz, ^4J = 2.9 Hz, 1H, H7), 6.80 – 6.74 (m, 2H, H5, H6), 4.82 (dd, ^3J = 5.9 Hz, ^3J = 4.4 Hz, 1H, H2), 4.29 – 4.21 (m, 2H, OCH_2), 2.86 – 2.71 (m, 2H, H4), 2.31 – 2.21 (m, 2H, H3), 1.29 (t, ^3J = 7.1 Hz, 3H, CH_3).

^{13}C-NMR (101 MHz, $CDCl_3$, 300 K): δ [ppm] = 170.5 (COOEt), 151.7 ($^1J_{CF}$ = 246 Hz, C8), 141.9 (d, $^2J_{CF}$ = 11.0 Hz, C8a), 124.4 (d, $^4J_{CF}$ = 3.5 Hz, C5), 123.9 (d, $^3J_{CF}$ = 1.6 Hz, C4a), 120.2 (d, $^3J_{CF}$ = 7. 3Hz, C6), 114.2 (d, $^2J_{CF}$ = 18.1 Hz, C7), 73.8 (C2), 61.6 (OCH_2), 24.2 (C3), 22.7 (d, $^4J_{CF}$ = 2.7 Hz, C4), 14.3 (CH_3).

^{19}F-NMR (376 MHz, $CDCl_3$, 300 K): δ [ppm] = −136.8 – −136.8 (m).

HRMS (ESI) *m/z* $[M+H]^+$ calculated for $[C_{12}H_{14}FO_3]^+$: 225.0921; found: 225.0919.

IR (film) $\tilde{\nu}_{max}$/cm^{-1} = 2981 (w, CH_{arom}), 2938 (w, CH_{aliph}), 2852 (w, CH_{aliph}), 1750 (m, C=O), 1734 (m, C=O), 1591 (m, C=C), 1480 (s, CH_{arom}), 1458 (m, CH_{aliph}), 1262 (m, C–O), 1167 (s, C–F), 1094 (s, C–O).

2,3-Dibromo-3-methylbutanoic acid methyl ester (*rac*-S-4)

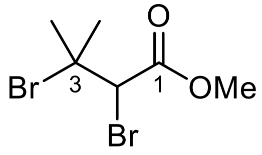

rac-**S-4**
$C_6H_{10}Br_2O_2$
MW = 273.95 gmol^{-1}

Bromine (9.24 g, 2.96 mL, 57.8 mmol, 1.10 equiv.) was added dropwise to a solution of 3-methyl-2-butenoic acid methyl ester (6.00 g, 6.95 mL, 52.6 mmol, 1.00 equiv.) in CH_2Cl_2 (350 mL) at 0 °C. The resulting brown solution was stirred at 0 °C for 15 min before the reaction was quenched by the addition of saturated aqueous Na_2SO_3 solution (100 mL). The layers were separated and the aqueous layer was extracted thrice with CH_2Cl_2 (3 × 30 mL). The combined organic layers were washed with brine (30 mL) and dried over Na_2SO_4 before the remaining solvents was removed under reduced pressure to yield 2,3-Dibromo-3-methylbutanoic acid methyl ester (*rac*-**S-4**) as a slightly orange oil without further purification (14.2 g, 51.8 mmol, 99%).

^{1}H-NMR (400 MHz, $CDCl_3$, 300 K): δ [ppm] = 4.65 (s, 1H, H2), 3.81 (s, 3H, OCH_3), 2.05 (s, 3H, $C3CH_3^a$), 1.95 (3H, $C3CH_3^b$).

^{13}C-NMR (101 MHz, $CDCl_3$, 300 K): δ [ppm] = 168.2 (C1), 61.7 (C3), 54.4 (C2), 53.0 (OCH_3), 33.9 (C3*C*H_3^b), 28.7 (C3*C*H_3^a).

HRMS (ESI) *m/z* $[M+H]^+$ calculated for $[C_6H_{11}{}^{81}Br_2O_2]^+$: 276.9077; found: 276.9073.

IR (film) $\tilde{\nu}_{max}/cm^{-1}$ = 2977 (w, CH_{aliph}), 2954 (w, CH_{aliph}), 1750 (s, C=O), 1389 (m, CH_{aliph}), 1245 (m, C–O), 1144 (s, C–O).

3,3-Dimethyl-1,4-benzodioxane-2-carboxylic acid methyl ester (*rac*-S-2s)

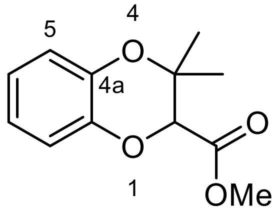

rac-**S-2s**
$C_{12}H_{14}O_4$
MW = 222.24 gmol^{-1}

Cs_2CO_3 (7.61 g, 23.4 mmol, 3.20 equiv.) was added to a solution of catechol (1.21 g, 11.0 mmol, 1.50 equiv.) in DMF (37 mL) at r.t. and the resulting blue suspension was stirred at r.t. for 15 min. Then, 2,3-dibromo-3-methylbutanoic acid methyl ester (*rac*-**S-4**) (2.00 g, 7.30 mmol, 1.00 equiv.) was added and the resulting brown mixture was stirred at 80 °C for 14 h. Water (20 mL) was added and the mixture was extracted four times with CH_2Cl_2 (4 × 60 mL). The combined organic layers were washed thrice with a saturated, aqueous LiCl solution (3 × 20 mL), dried over Na_2SO_4 and the remaining solvents were removed under reduced pressure. The obtained crude product was then purified by FCC (SiO_2, 0.5 $\rightarrow$ 2% EtOAc/hexanes) to yield 3,3-dimethyl-1,4-benzodioxane-2-carboxylic acid methyl ester (*rac*-**S-2s**) (245 mg, 1.10 mmol, 18%) as a colorless oil.

TLC (2% EtOAc/hexanes): R_f = 0.61 [UV, $KMnO_4$].

^{1}H-NMR (400 MHz, $CDCl_3$, 300 K): δ [ppm] = 6.99 – 6.97 (m, 1H, H5/H8), 6.91 –6.83 (m, 3H, H5/H8, H6, H7), 4.42 (s, 1H, H2), 3.82 (s, 3H, OCH_3), 1.47 (s, 3H, $C3CH_3^a$), 1.33 (s, 3H, $C3CH_3^b$).

^{13}C-NMR (101 MHz, $CDCl_3$, 300 K): δ [ppm] = 168.5 (*C*OOCH$_3$), 141.9 (C4a), 141.4 (C8a), 122.4 (C6/C7), 121.5 (C6/C7), 117.6 (C5/C8), 117.0 (C5/C8), 78.8 (C2), 73.4 (C3), 52.6 (OCH_3), 25.1 (CH_3^a), 21.3 (CH_3^b).

HRMS (ESI) *m/z* $[M+H]^+$ calculated for $[C_{12}H_{15}O_4]^+$: 223.0965; found: 223.0959.

IR (film) $\tilde{\nu}_{max}/cm^{-1}$ = 3045 (w, CH_{arom}), 2985 (m, CH_{arom}), 2954 (w, CH_{aliph}), 2924 (w, CH_{aliph}), 2854 (w, CH_{aliph}), 1760 (m, C=O), 1740 (m, C=O), 1598 (w, C=C), 1494 (s, CH_{arom}), 1194 (s, C-O), 1267 (s, C-O), 1208 (m, C-O).

6-Ethylchromane-2-carboxylic acid (*rac*-**S-3i**)

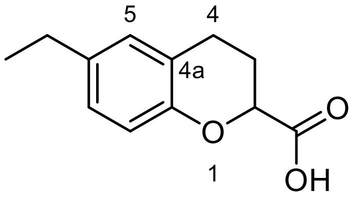

rac-**S-3i**
$C_{12}H_{14}O_3$
MW = 206.24 gmol^{-1}

According to GP D, a solution of 6-ethylchromane-2-carboxylic acid ethyl ester (*rac*-**S-2i**) (200 mg, 854 µmol, 1.00 equiv.) in THF (850 µL) was added to a solution of NaOH (137 mg, 3.41 mmol, 4.00 equiv.) in water (3.4 mL) at r.t. and the resulting mixture was stirred at r.t. for 14 h. More water was added and the solution was washed thrice with Et_2O (3 × 5 mL) before the pH value of the aqueous layer was adjusted to 1 by addition of aqueous HCl (6.0 M). The resulting suspension was extracted thrice with EtOAc (3 × 10 mL) and the combined organic layers were washed with brine and dried over Na_2SO_4 before the remaining solvents were removed under reduced pressure to yield 6-ethylchromane-2-carboxylic acid (*rac*-**S-3i**) as a white solid (125 mg, 606 mmol, 71%).

M.p.: 120°C

^{1}H-NMR (400 MHz, $CDCl_3$, 300 K): δ [ppm] = 6.97 (dd, ^3J = 8.3 Hz, ^4J = 2.2 Hz, 1H, H7), 6.89 – 6.84 (m, 2H, H6, H8), 4.72 (dd, ^3J = 8.4 Hz, ^3J = 3.4 Hz, 1H, H2), 2.86 – 2.70 (m, 2H, H4), 2.56 (q, ^3J = 7.6 Hz, 2H, C*H*$_2$CH$_3$), 2.36 (*virt.* dtd, ^2J = 13.9 Hz, $^3J \approx {}^3J$ = 5.9 Hz, ^3J = 3.4 Hz, 1H, H3^a), 2.17 (*virt.* dtd, ^2J = 13.9 Hz, $^3J \approx {}^3J$ = 8.6 Hz, ^3J = 5.7 Hz, 1H, H3^b), 1.20 (t, ^3J = 7.6 Hz, 3H, CH$_2$C*H*$_3$).

^{13}C-NMR (101 MHz, $CDCl_3$, 300 K): δ [ppm] = 174.7 (COOH), 150.9 (C8a), 137.4 (C6), 128.8 (C5), 127.4 (C7), 121.0 (C4a), 116.8 (C8), 73.6 (C2), 28.1 (*C*H$_2$CH$_3$), 24.8 (C3), 23.7 (C4), 15.9 (CH$_2$*C*H$_3$).

HRMS (ESI) *m/z* [M−H]⁻ calculated for $[C_{12}H_{13}O_3]^-$: 205.0870; found: 205.0868.

IR (film) $\tilde{\nu}_{max}/cm^{-1}$ = 3300 – 2500 (m, O–H), 3060 (m, CH_{arom}), 3010 (m, CH_{arom}), 2962 (w, CH_{aliph}), 2928 (w, CH_{aliph}), 2870 (m, CH_{aliph}), 1710 (s, C=O), 1590 (m, C=C), 1498 (m, CH_{arom}), 1448 (m, CH_{aliph}), 1236 (s, C–O), 1211 (s, C–O), 1128 (m, C–O).

5-Methylchromane-2-carboxylic acid (*rac*-**S-3j**)

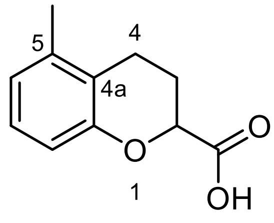

rac-**S-3j**
$C_{11}H_{12}O_3$
MW = 192.21 gmol^{-1}

According to GP D, a solution of 5-methylchromane-2-carboxylic acid ethyl ester (*rac*-**S-2j**) (120 mg, 545 µmol, 1.00 equiv.) in THF (2.2 mL) was added to a solution of NaOH (87.2 mg, 2.18 mmol, 4.00 equiv.) in water (540 µL) at r.t. and the resulting mixture was stirred at r.t. for 14 h. More water was added and the solution was washed thrice with Et_2O (3 × 20 mL) before the pH value of the aqueous layer was adjusted to 1 by addition of aqueous HCl (6.0 M). The resulting suspension was extracted thrice with EtOAc (3 × 20 mL) and the combined organic layers were washed with brine and dried over Na_2SO_4 before the remaining solvents were removed under reduced pressure to yield 5-methylchromane-2-carboxylic acid (*rac*-**S-3j**) as a white solid (97.3 mg, 506 µmol, 93%).

M.p.: 142 °C

^{1}H-NMR (400 MHz, $CDCl_3$, 300 K): δ [ppm] = 7.05 (*virt.* t, $^3J \approx {}^3J$ = 7.8 Hz, 1H, H7), 6.82 –6.79 (m, 2H, H6, H8), 4.71 (dd, ^3J = 8.4 Hz, ^3J = 3.4 Hz, 1H, H2), 2.78 – 2.66 (m, 2H, H4), 2.41 (*virt.* dtd, ^2J = 13.8 Hz, $^3J \approx {}^3J$ = 6.1 Hz, ^3J = 3.4 Hz, 1H, H3^a), 2.27 – 2.17 (m, 4H, H3^b, CH_3).

^{13}C-NMR (101 MHz, $CDCl_3$, 300 K): δ [ppm] = 175.4 (COOH), 153.1 (C8a), 137.6 (C5), 127.2 (C7), 122.9 (C6), 120.2 (C4a), 114.8 (C8), 73.0 (C2), 24.8 (C3), 21.3 (C4), 19.2 (CH_3).

HRMS (ESI) *m/z* [M−H]⁻ calculated for $[C_{11}H_{11}O_3]^-$: 191.0714; found: 191.0705.

IR (film) $\tilde{\nu}_{max}/cm^{-1}$ = 3300 – 2500 (w, O–H), 3069 (w, CH_{arom}), 3016 (w, CH_{arom}), 2971 (w, CH_{aliph}), 2940 (w, CH_{aliph}), 2900 w, CH_{aliph}), 2861 (w, CH_{aliph}), 1728 (s, C=O), 1703 (s, C=O), 1584 (m, C=C), 1466 (m, CH_{aliph}), 1246 (s, C–O), 1209 (m, C–O), 1114 (s, C – O).

7-Methylchromane-2-carboxylic acid (*rac*-**S-3k**)

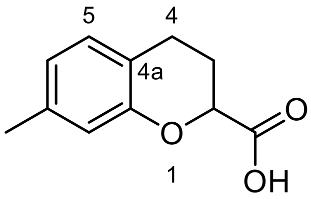

rac-**S-3k**
$C_{11}H_{12}O_3$
MW = 192.21 gmol^{-1}

According to GP D, a solution of 7-methylchromane-2-carboxylic acid ethyl ester (*rac*-**S-2k**) (340 mg, 1.54 mmol, 1.00 equiv.) in THF (1.5 mL) was added to a solution of NaOH (147 mg, 6.17 mmol, 4.00 equiv.) in water (6.2 mL) at r.t. and the resulting mixture was stirred at r.t. for 14 h. More water was added and the solution was washed thrice with Et_2O (3 × 20 mL) before the pH value of the aqueous layer was adjusted to 1 by addition of aqueous HCl (6.0 M). The resulting suspension was extracted thrice with EtOAc (3 × 20 mL) and the combined organic layers were washed with brine and dried over Na_2SO_4 before the remaining solvents were removed under reduced pressure to yield 7-methylchromane-2-carboxylic acid (*rac*-**S-3k**) as a white solid (238 mg, 1.24 mmol, 80%).

M.p.: 151 °C

^{1}H-NMR (400 MHz, $CDCl_3$, 300 K): δ [ppm] = 6.94 (d, ^3J = 7.7 Hz, 1H, H5), 6.76 (d, ^3J = 1.7 Hz, 1H, H8), 6.72 (dd, ^3J = 7.7 Hz, ^4J = 1.7 Hz, 1H, H6), 4.74 (dd, ^3J = 7.9 Hz, ^3J = 3.6 Hz, 1H, H2), 2.87 – 2.72 (m, 2H, H4), 2.38 – 2.29 (m, 4H, H3^a, C7CH_3), 2.18 (*virt.* dtd, ^2J = 13.7 Hz, $^3J \approx {}^3J$ = 8.1 Hz, ^3J = 5.7 Hz, 1H, H3^b).

^{13}C-NMR (101 MHz, $CDCl_3$, 300 K): δ [ppm] = 175.7 (COOH), 152.8 (C8a), 137.9 (C7), 129.4 (C5), 122.4 (C6), 118.2 (C4a), 117.4 (C8), 73.4 (C2), 24.8 (C3), 23.2 (C4), 21.2 (C7*C*H_3).

HRMS (ESI) *m/z* [M−H]$^-$ calculated for $[C_{11}H_{11}O_3]^-$: 191.0714; found: 191.0709.

IR (film) $\tilde{\nu}_{max}$/cm^{-1} = 3300 – 2500 (m, O–H), 3052 (w, CH_{arom}), 3025 (w, CH_{arom}), 2974 (w, CH_{arom}), 2947 w, CH_{aliph}), 2916 (w, CH_{aliph}), 1708 (s, C=O), 1579 (m, C=C), 1450 (w, CH_{aliph}), 1249 (w, C–O), 1155 (w, C–O).

6,7,8,9-Tetrahydro-benzo[*g*]chromane-2-carboxylic acid (*rac*-**S-3m**)

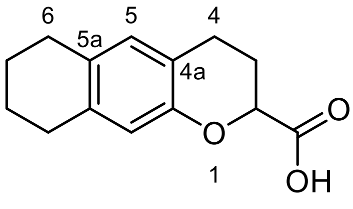

rac-**S-3m**
$C_{14}H_{16}O_3$
MW = 232.28 $gmol^{-1}$

According to GP D, a solution of 6,7,8,9-tetrahydro-4-oxo-4*H*-benzo[*g*]chromene-2-carboxylic acid ethyl ester (*rac*-**S-2m**) (120 mg, 461 µmol, 1.00 equiv.) in THF (1.8 mL) was added to a solution of NaOH (73.8 mg, 1.84 mmol, 4.00 equiv.) in water (460 µL) at r.t. and the resulting mixture was stirred at r.t. for 14 h. More water was added and the solution was washed thrice with Et_2O (3 × 20 mL) before the pH value of the aqueous layer was adjusted to 1 by addition of aqueous HCl (6.0 M). The resulting suspension was extracted thrice with EtOAc (3 × 20 mL) and the combined organic layers were washed with brine and dried over Na_2SO_4 before the remaining solvents were removed under reduced pressure to yield 6,7,8,9-Tetrahydro-4-oxo-4*H*-benzo[*g*]chromene-2-carboxylic acid (*rac*-**S-3m**) as a white solid (101 mg, 435 µmol, 94%).

M.p.: 165 °C (decomposition)

^{1}H-NMR (400 MHz, $CDCl_3$, 300 K): δ [ppm] = 6.76 (s, 1H, H5), 6.65 (s, 1H, H8), 4.71 (dd, ^3J = 8.0 Hz, ^3J = 3.5 Hz, 1H, H2), 2.85 – 2.65 (m, 6H, H4, H6, H9), 2.33 (*virt.* dtd, ^2J = 13.7 Hz, $^3J \approx {}^3J$ = 6.1 Hz, ^3J = 3.5 Hz, 1H, H3[a]), 2.16 (*virt.* dtd, ^2J = 13.7 Hz, $^3J \approx {}^3J$ = 8.2 Hz, ^3J = 5.7 Hz, 1H, H3[b]), 1.78 – 1.74 (m, 4H, H7, H8).

^{13}C-NMR (101 MHz, $CDCl_3$, 300 K): δ [ppm] = 175.2 (COOH), 150.6 (C8a), 137.0 (C9a), 130.2 (C5a), 129.8 (C5), 118.7 (C4a), 116.6 (C8), 73.5 (C2), 29.3 (C6/C9), 28.7 (C6/C9), 24.9 (C3), 23.5 (C7/C8), 23.3 (C7/C8), 23.2 (C4).

HRMS (ESI) *m/z* $[M-H]^-$ calculated for $[C_{14}H_{15}O_3]^-$: 231.1027; found: 231.1026.

IR (film) $\tilde{\nu}_{max}/cm^{-1}$ = 3300 – 2500 (m, O–H), 3043 (w, CH_{arom}), 3008 (w, CH_{arom}), 2926 (m, CH_{aliph}), 2851 (m, CH_{aliph}), 1703 (s, C=O), 1623 (w, C=C), 1498 (m, CH_{arom}), 1438 (m, CH_{aliph}), 1222 (s, C–O), 1208 (m, C–O), 1119 (s, C–O).

8-Fluorochromane-2-carboxylic acid (*rac*-**S-3q**)

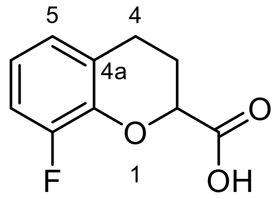

rac-**S-3q**
$C_{10}H_9FO_3$
MW = 196.18 gmol[-1]

According to GP D, a solution of 8-fluorochromane-2-carboxylic acid ethyl ester (*rac*-**S-2q**) (50.0 mg, 223 µmol, 1.00 equiv.) in THF (890 µL) was added to a solution of NaOH (35.7 mg, 892 µmol, 4.00 equiv.) in water (220 µL) at r.t. and the resulting mixture was stirred at r.t. for 14 h. More water was added and the solution was washed thrice with Et_2O (3 × 10 mL) before the pH value of the aqueous layer was adjusted to 1 by addition of aqueous HCl (6.0 M). The resulting suspension was extracted thrice with EtOAc (3 × 10 mL) and the combined organic layers were washed with brine and dried over Na_2SO_4 before the remaining solvents were removed under reduced pressure to yield 8-fluorochromane-2-carboxylic acid (*rac*-**S-3q**) as a white solid (38.0 mg, 194 µmol, 87%).

M.p.: 97 °C

^{1}H-NMR (400 MHz, $CDCl_3$, 300 K): δ [ppm] = 6.97 – 6.90 (m, 1H, Hx), 6.84 – 6.78 (m, 2H, Hx, Hx), 4.85 (dd, ^3J = 7.2 Hz, ^3J = 3.9 Hz, 1H, H2), 2.92 – 2.78 (m, 2H, H4), 2.37 (dddd, ^2J = 14.0 Hz, ^3J = 7.2 Hz, ^3J = 5.9 Hz, ^3J = 3.9 Hz, 1H, H3[a]), 2.25 (*virt.* dtd, ^2J = 14.0 Hz, $^3J \approx {}^3J$ = 7.3 Hz, ^3J = 5.7 Hz, 1H, H3[b]).

^{13}C-NMR (101 MHz, $CDCl_3$, 300 K): δ [ppm] = 175.0 (COOH), 151.6 (d, $^1J_{CF}$ = 246 Hz, C8), 141.4 (d, $^2J_{CF}$ = 10.9 Hz, C8a), 124.5 (d, $^4J_{CF}$ = 3.6 Hz, C5), 123.9 (d, $^3J_{CF}$ = 0.9 Hz, C4a), 120.7 (d, $^3J_{CF}$ = 7. 3Hz, C6), 114.4 (d, $^2J_{CF}$ = 18.0 Hz, C7), 73.4 (C2), 24.2 (C3), 22.9 (d, $^4J_{CF}$ = 2.7 Hz, C4).

^{19}F-NMR (376 MHz, $CDCl_3$, 300 K): δ [ppm] = −136.6 – −136.7 (m).

HRMS (ESI) *m/z* $[M-H]^-$ calculated for $[C_{10}H_8FO_3]^-$: 195.0463; found: 195.0454.

IR (film) $\tilde{\nu}_{max}/cm^{-1}$ = 3300 – 2500 (m, O–H), 3043 (w, CH_{arom}), 2988 (w, CH_{arom}), 2960 (w, CH_{aliph}), 2924 (w, CH_{aliph}), 2896 (w, CH_{aliph}), 1718 (s, C=O), 1591 (m, C=C), 1480 (s, CH_{arom}), 1442 (m, CH_{aliph}), 1262 (s, C–O), 1208 (s, C–O), 1101 (s, C–F).

3,3-Dimethyl-1,4-benzodioxane-2-carboxylic acid (*rac*-S-3s)

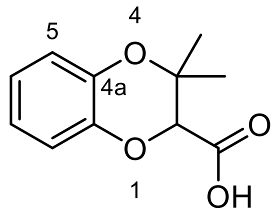

rac-**S-3s**
$C_{11}H_{12}O_4$
MW = 208.21 gmol^{-1}

According to GP D, a solution of 3,3-dimethyl-1,4-benzodioxane-2-carboxylic acid methyl ester (*rac*-**S-2s**) (103 mg, 462 µmol, 1.00 equiv.) in THF (1.9 mL) was added to a solution of NaOH (73.9 mg, 1.85 mmol, 4.00 equiv.) in water (460 µL) at r.t. and the resulting mixture was stirred at r.t. for 14 h. More water was added and the solution was washed thrice with Et_2O (3 × 20 mL) before the pH value of the aqueous layer was adjusted to 1 by addition of aqueous HCl (6.0 M). The resulting suspension was extracted thrice with EtOAc (3 × 20 mL) and the combined organic layers were washed with brine and dried over Na_2SO_4 before the remaining solvents were removed under reduced pressure. The crude product was then purified by FCC (SiO_2, 20% EtOAc/hexanes + 1% AcOH → 80% EtOAc/hexanes + 1% AcOH) to yield 3,3-dimethyl-1,4-benzodioxane-2-carboxylic acid (*rac*-**S-3s**) (53.0 mg, 255 mmol, 55%) as an off-white solid.

M.p.: 134°C

TLC (80% EtOAc/hexanes + 1% AcOH): R_f = 0.21 [UV, $KMnO_4$].

^{1}H-NMR (400 MHz, $CDCl_3$, 300 K): δ [ppm] = 7.01 – 6.98 (m, 1H, H5/H8), 6.93 –6.85 (m, 3H, H5/H8, H6, H7), 4.45 (s, 1H, H2), 1.55 (s, 3H, $CH_3{}^a$), 1.36 (s, 3H, $CH_3{}^b$).

^{13}C-NMR (101 MHz, $CDCl_3$, 300 K): δ [ppm] = 171.8 (COOH), 141.8 (C4a), 140.9 (C8a), 122.7 (C6/C7), 121.7 (C6/C7), 117.7 (C5/C8), 116.9 (C5/C8), 78.4 (C2), 73.5 (C3), 21.2 ($CH_3{}^a$), 21.2 ($CH_3{}^b$).

HRMS (ESI) *m/z* $[M-H]^-$ calculated for $[C_{11}H_{11}O_4]^-$: 207.0663; found: 207.0657.

IR (film) $\tilde{\nu}_{max}/cm^{-1}$ = 3300 – 2700 (m, O–H), 3047 (w, CH_{arom}), 2986 (w, CH_{arom}), 2926 (w, CH_{aliph}), 2855 (w, CH_{aliph}), 1727 (s, C=O), 1599 (w, C=C), 1494 (s, CH_{arom}), 1268 (s, C–O).

4-Oxothiochromane-2-carboxylic acid (*rac*-**S-5**)

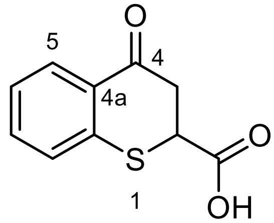

rac-**S-5**
$C_{10}H_8O_3S$
MW = 208.23 gmol^{-1}

Following a modified procedure by Ortiz *et al.*[98] thiophenol (3.37 g, 3.12 mL, 30.6 mmol, 1.00 equiv.) was added to powderized maleic anhydride (3.00 g, 30.6 mmol, 1.00 equiv.) at r.t. and the formed yellow suspension was stirred at r.t. for 45 min. Then, the mixture was heated to 50 °C for 40 min before it was allowed to cool to 0 °C. NEt_3 (40.0 mg, 42.6 µL, 306 µmol, 10 mol%) was added and the resulting dark mixture was again heated to 50 °C for 24 h. $AlCl_3$ at 0 °C was added portionwise and the resulting dark solids were allowed to sit at r.t. for 2 h. Ice (100 mL) was added as well as a 2 M aqueous HCl solution until a pH of 1 was reached. The precipitate was then separated by filtration and then subjected to FCC (SiO_2, 20 → 30% EtOAc/hexanes + 5% AcOH) to yield 4-oxothiochromane-2-carboxylic acid (*rac*-**S-5**) as an off-white solid (3.69 mg, 17.7 mmol, 58%).

TLC (20% EtOAc/pentane + 5% AcOH): R_f = 0.15 [UV, $KMnO_4$].

^{1}H-NMR (400 MHz, DMSO-d$_6$, 300 K): δ [ppm] = 13.1 (bs, 1H, COOH), 7.95 (dd, ^3J = 8.1 Hz, ^4J = 1.6 Hz, 1H, H5), 7.49 (ddd, ^3J = 7.9 Hz, ^3J = 7.2 Hz, ^4J = 1.6 Hz, 1H, H7), 7.35 (dd, ^3J = 7.9 Hz, ^4J = 1.2 Hz, 1H, H8), 7.25 (ddd, ^3J = 8.1 Hz, ^3J = 7.2 Hz, ^4J = 1.2 Hz, 1H, H6), 4.39 (dd, ^3J = 6.2 Hz, ^3J = 4.3 Hz, 1H, H2), 3.15 – 3.02 (m, 2H, H3).

^{13}C-NMR (101 MHz, DMSO-d$_6$, 300 K): δ [ppm] = 192.1 (C4), 171.6 (COOH), 138.6 (C8a), 133.7 (C7), 130.1 (C4a), 127.9 (C5), 127.3 (C8), 125.4 (C6), 41.3 (C2), 40.9 (C3).

The spectroscopic data matches the one reported in the literature.[98]

Chromane-2-carboxamide (*rac*-**1a**)

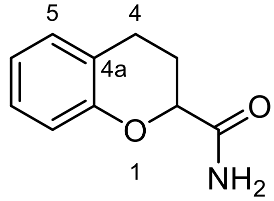

rac-**1a**
$C_{10}H_{11}NO_2$
MW = 177.20 gmol^{-1}

According to GP E, HOBt · H_2O (600 mg, 3.92 mmol, 1.20 equiv.) was added to a solution of chromane-2-carboxylic acid (582 mg, 3.27 mmol, 1.00 equiv.) in CH_2Cl_2 (109 mL) at 0 °C. After 10 min, EDCI · HCl (751 mg, 3.92 mmol, 1.20 equiv.) was added and after stirring the resulting mixture at 0 °C for another 30 min, aqueous ammonia (14 M, 3.34 g, 14.0 mL, 196 mmol, 60.0 equiv.) was added before the reaction mixture was allowed to warm to r.t. and stirred vigorously for 14 h. Then, water (20 mL) was added, the layers were separated and the aqueous layer was extracted thrice with CH_2Cl_2 (3 × 30 mL). The combined organic layers were washed with brine and dried over Na_2SO_4. The solvents were

removed under reduced pressure and the crude product was subjected to FCC (SiO_2, 50 → 100% EtOAc/hexanes) to yield chromane-2-carboxamide (*rac*-**1a**) as a white solid (514 mg, 2.90 mmol, 89%).

TLC (80% EtOAc/pentane): R_f = 0.55 [UV, $KMnO_4$].

M.p.: 141 °C

^{1}H-NMR (400 MHz, $CDCl_3$, 300 K): δ [ppm] = 7.15 – 7.11 (m, 1H, H7), 7.09 – 7.07 (m, 1H, H5), 6.92 – 6.87 (m, 2H, H6, H8), 6.60 (bs, 1H, NH_2^a), 6.00 (bs, 1H, NH_2^b), 4.54 (dd, ^3J = 9.3 Hz, ^3J = 3.1 Hz, 1H, H2), 2.93 – 2.76 (m, 2H, H4), 2.41 (dddd, ^2J = 13.7 Hz, ^3J = 5.7 Hz, ^3J = 4.9 Hz, ^3J = 3.1 Hz, 1H, $H3^a$), 2.08 (*virt.* dtd, ^2J = 13.7 Hz, $^3J \approx {}^3J$ = 9.6 Hz, ^3J = 5.5 Hz, 1H, $H3^b$).

^{13}C-NMR (101 MHz, $CDCl_3$, 300 K): δ [ppm] = 173.8 ($CONH_2$), 153.0 (C8a), 129.9 (C5), 127.7 (C7), 122.2 (C4a), 121.4 (C6), 116.8 (C8), 75.5 (C2), 24.9 (C3), 24.1 (C4).

HRMS (ESI) *m/z* $[M+H]^+$: calculated for $[C_{10}H_{12}NO_2]^+$: 178.0863; found: 178.0862.

IR (film) $\tilde{\nu}_{max}/cm^{-1}$ = 3402 (m, NH), 3166 (m, NH), 3040 (w, CH_{arom}), 3024 (w, CH_{arom}), 2959 (w, CH_{aliph}), 2922 (w, CH_{aliph}), 1660 (s, C=O), 1608 (m, C=C), 1488 (m, CH_{arom}), 1456 (m, CH_{aliph}), 1231 (s, C–O), 1112 (m, C–N).

6-Fluorochromane-2-carboxamide (*rac*-**1b**)

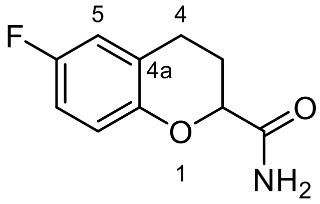

rac-**1b**
$C_{10}H_{10}FNO_2$
MW = 195.19 $gmol^{-1}$

According to GP E, HOBt · H_2O (375 mg, 2.45 mmol, 1.20 equiv.) was added to a solution of 6-fluorochromane-2-carboxylic acid (400 mg, 2.04 mmol, 1.00 equiv.) in CH_2Cl_2 (68 mL) at 0 °C. After 10 min, EDCI · HCl (469 mg, 2.45 mmol, 1.20 equiv.) was added and after stirring the resulting mixture at 0 °C for another 30 min, aqueous ammonia (14 M, 2.08 g, 8.74 mL, 122 mmol, 60.0 equiv.) was added before the reaction mixture was allowed to warm to r.t. and stirred vigorously for 14 h. Then, water (20 mL) was added, the layers were separated and the aqueous layer was extracted thrice with CH_2Cl_2 (3 × 30 mL). The combined organic layers were washed with brine and dried over Na_2SO_4. The solvents were removed under reduced pressure and the crude product was subjected to FCC (SiO_2, 50 → 100% EtOAc/hexanes) to yield 6-fluorochromane-2-carboxamide (*rac*-**1b**) as a white solid (353 mg, 1.81 mmol, 89%).

TLC (60% EtOAc/pentane): R_f = 0.27 [UV, $KMnO_4$].

M.p.: 152 °C

^{1}H-NMR (400 MHz, $CDCl_3$, 300 K): δ [ppm] = 6.83 – 6.76 (m, 3H, H5, H7, H8), 6.55 (bs, 1H, $NH_2{}^a$), 5.06 (bs, 1H, $NH_2{}^b$), 4.50 (dd, ^3J = 9.4 Hz, ^3J = 3.0 Hz, 1H, H2), 2.90 – 2.74 (m, 2H, H4), 2.46 (dddd, ^2J = 13.7 Hz, ^3J = 5.9 Hz, ^3J = 4.7 Hz, ^3J = 3.0 Hz, 1H, H3^a), 2.08 (*virt.* dtd, ^2J = 13.7 Hz, $^3J \approx {}^3J$ = 9.7 Hz, ^3J = 5.6 Hz, 1H, H3^b).

^{13}C-NMR (101 MHz, $CDCl_3$, 300 K): δ [ppm] = 173.6 ($CONH_2$), 157.4 (d, $^1J_{CF}$ = 240 Hz, C6), 149.0 (d, $^4J_{CF}$ = 2.2 Hz, C8a), 123.4 (d, $^3J_{CF}$ = 7.5 Hz, C4a), 117.7 (d, $^3J_{CF}$ = 8.2 Hz, C8), 115.8 (d, $^2J_{CF}$ = 22.8 Hz, C5), 114.4 (d, $^2J_{CF}$ = 23.4 Hz, C7), 75.5 (C2), 24.5 (C3), 24.3 (d, $^4J_{CF}$ = 1.1 Hz, C4).

^{19}F-NMR (376 MHz, $CDCl_3$, 300 K): δ [ppm] = −122.9 – −123.0 (m).

HRMS (ESI) *m/z* $[M+H]^+$ calculated for $[C_{10}H_{11}FNO_2]^+$: 196.0768; found: 196.0769.

IR (film) $\tilde{\nu}_{max}$/cm^{-1} = 3319 (m, NH), 3188 (m, NH), 3076 (w, CH_{arom}), 2960 (m, CH_{aliph}), 2938 (m, CH_{aliph}), 2924 (CH_{aliph}), 2851 (m, CH_{aliph}), 1663 (s, C=O), 1617 (m, C=C), 1490 (s, CH_{arom}), 1432 (m, CH_{aliph}), 1260 (m, C–O), 1205 (s, C–F), 1140 (m, C–N), 1076 (s, C–O).

6-Chlorochromane-2-carboxamide (*rac*-**1c**)

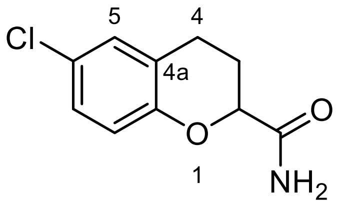

rac-**1c**
$C_{10}H_{10}ClNO_2$
MW = 211.65 gmol^{-1}

According to GP E, HOBt · H_2O (173 mg, 1.13 mmol, 1.20 equiv.) was added to a solution of 6-chlorochromane-2-carboxylic acid (200 mg, 940 µmol, 1.00 equiv.) in CH_2Cl_2 (31 mL) at 0 °C. After 10 min, EDCI · HCl (216 mg, 1.13 mmol, 1.20 equiv.) was added and after stirring the resulting mixture at 0 °C for another 30 min, aqueous ammonia (14 M, 961 mg, 4.03 mL, 56.4 mmol, 60.0 equiv.) was added before the reaction mixture was allowed to warm to r.t. and stirred vigorously for 14 h. Then, water (10 mL) was added, the layers were separated and the aqueous layer was extracted thrice with CH_2Cl_2 (3 × 20 mL). The combined organic layers were washed with brine and dried over Na_2SO_4. The solvents were removed under reduced pressure and the crude product was subjected to FCC (SiO_2, 50 → 100% EtOAc/hexanes) to yield 6-chlorochromane-2-carboxamide (*rac*-**1c**) as a white solid (166 mg, 784 µmol, 83%).

TLC (80% EtOAc/pentane): R_f = 0.46 [UV, $KMnO_4$].

M.p.: 178 °C

^{1}H-NMR (400 MHz, $CDCl_3$, 300 K): δ [ppm] = 7.10 – 7.06 (m, 2H, H5, H7), 6.81 (d, ^3J = 8.5 Hz, 1H, H8), 6.52 (bs, 1H, $NH_2{}^a$), 5.67 (bs, 1H, $NH_2{}^b$), 4.53 (dd, ^3J = 9.3 Hz, ^3J = 3.1 Hz, 1H, H2),

2.90 – 2.74 (m, 2H, H4), 2.40 (dddd, ^2J = 13.8 Hz, ^3J = 5.8 Hz, ^3J = 4.9 Hz, ^3J = 3.1 Hz, 1H, H3[a]), 2.06 (*virt.* dtd, ^2J = 13.8 Hz, $^3J \approx {}^3J$ = 9.6 Hz, ^3J = 5.6 Hz, 1H, H3[b]).

^{13}C-NMR (101 MHz, $CDCl_3$, 300 K): δ [ppm] = 173.1 ($CONH_2$), 151.7 (C8a), 129.5 (C5), 127.7 (C7), 126.3 (C6), 123.8 (C4a), 118.1 (C8), 75.6 (C2), 24.5 (C3), 24.1 (C4).

HRMS (ESI) *m/z* $[M+H]^+$ calculated for $[C_{10}H_{11}{}^{35}ClNO_2]^+$: 212.0473; found: 212.0473.

IR (film) $\tilde{\nu}_{max}/cm^{-1}$ = 3385 (m, NH), 3180 (m, NH), 3076 (w, CH_{arom}), 2969 (w, CH_{aliph}), 2923 (w, CH_{aliph}), 1659 (s, C=O), 1620 (m, C=C), 1481 (m, CH_{arom}), 1444 (w, CH_{aliph}), 1232 (s, C–O), 1112 (w, C–Cl), 1074 (m, C–N).

6-Bromochromane-2-carboxamide (*rac*-**1d**)

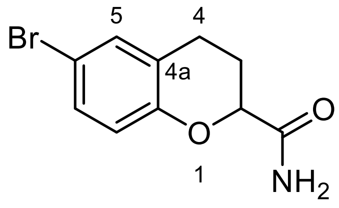

rac-**1d**
$C_{10}H_{10}BrNO_2$
MW = 256.10 gmol^{-1}

According to GP E, HOBt · H_2O (247 mg, 1.62 mmol, 1.20 equiv.) was added to a solution of 6-bromochromane-2-carboxylic acid (346 mg, 1.35 mmol, 1.00 equiv.) in CH_2Cl_2 (11 mL) at 0 °C. After 10 min, EDCI · HCl (310 mg, 1.62 mmol, 1.20 equiv.) was added and after stirring the resulting mixture at 0 °C for another 30 min, aqueous ammonia (14 M, 2.83 g, 5.77 mL, 80.8 mmol, 60.0 equiv.) was added before the reaction mixture was allowed to warm to r.t. and stirred vigorously for 16 h. Then, water (20 mL) was added, the layers were separated and the aqueous layer was extracted thrice with CH_2Cl_2 (3 × 20 mL). The combined organic layers were washed with brine and dried over Na_2SO_4. The solvents were removed under reduced pressure and the crude product was subjected to FCC (SiO_2, 50 → 100% EtOAc/hexanes) to yield 6-bromochromane-2-carboxamide (*rac*-**1d**) as a white solid (305 mg, 1.19 mmol, 88%).

TLC (50% EtOAc/hexanes): R_f = 0.40 [UV, $KMnO_4$].

M.p.: 220 °C

^{1}H-NMR (400 MHz, $CDCl_3$, 300 K): δ [ppm] = 7.24 – 7.21 (m, 2H, H5, H7), 6.76 (d, ^3J = 8.9 Hz, 1H, H8), 6.51 (bs, 1H, NH_2[a]), 5.65 (bs, 1H, NH_2[b]), 4.53 (dd, ^3J = 9.3 Hz, ^3J = 3.1 Hz, 1H, H2), 2.90 – 2.74 (m, 2H, H4), 2.40 (dddd, ^2J = 13.8 Hz, ^3J = 5.8 Hz, ^3J = 4.9 Hz, ^3J = 3.1 Hz, 1H, H3[a]), 2.06 (*virt.* dtd, ^2J = 13.8 Hz, $^3J \approx {}^3J$ = 9.5 Hz, ^3J = 5.6 Hz, 1H, H3[b]).

^{13}C-NMR (101 MHz, $CDCl_3$, 300 K): δ [ppm] = 173.0 ($CONH_2$), 152.2 (C8a), 132.5 (C5), 130.7 (C7), 124.4 (C4a), 118.6 (C8), 113.6 (C6), 75.6 (C2), 24.4 (C3), 24.0 (C4).

HRMS (ESI) *m/z* $[M+H]^+$ calculated for $[C_{10}H_{11}{}^{79}BrNO_2]^+$: 255.9968; found: 255.9968.

IR (film) $\tilde{\nu}_{max}$/cm^{-1} = 3380 (m, NH), 3171 (m, NH), 3072 (w, CH_{arom}), 2970 (w, CH_{aliph}), 2933 (w, CH_{aliph}), 2842 (w, CH_{aliph}), 1656 (s, C=O), 1618 (m, C=C), 1476 (m, CH_{arom}), 1441 (m, CH_{aliph}), 1230 (m, C–O), 1176 (m, C–N), 1110 (m, C–O), 1068 (m, C–Br).

6-Iodochromane-2-carboxamide (*rac*-**1e**)

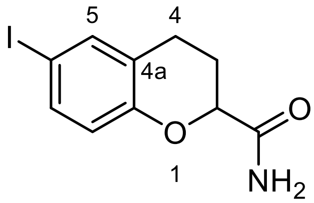

rac-**1e**
$C_{10}H_{10}INO_2$
MW = 303.10 gmol^{-1}

To a solution of chroman-2-carboxylic acid (1.50 g, 8.42 mmol, 1.00 equiv.) in acetic acid (40.0 mL) was added anhydrous $ZnCl_2$ (1.49 g, 10.9 mmol, 1.30 equiv.) and benzyltrimethylammonium dichloroiodate (3.08 g, 8.84 mmol, 1.05 equiv.). The resulting solution was stirred at room temperature for 14 h. Then, water (40 mL) was added, the layers were separated and the aqueous layer was extracted thrice with CH_2Cl_2 (3 × 25 mL). The combined organic layers were washed with brine and dried over Na_2SO_4 and concentrated under reduced pressure. The residue was dissolved in methylene chloride (20 mL) and washed with 10% aqueous sodium thiosulfate (8 mL), brine (10 mL) and dried over Na_2SO_4 and concentrated under reduced pressure to obtained the crude 6-iodochromane-2-carboxylic acid which was used for the next step without any purification.

According to GP E, to GP E, HOBt · H_2O (302 mg, 1.97 mmol, 1.20 equiv.) was added to a solution of crude 6-iodochromane-2-carboxylic acid (500 mg, 1.64 mmol, 1.00 equiv.) in CH_2Cl_2 (20 mL) at 0 °C. After 10 min, EDCI · HCl (378 mg, 1.97 mmol, 1.20 equiv.) was added and after stirring the resulting mixture at 0 °C for another 30 min, aqueous ammonia (14 M, 3.46 g, 7.05 mL, 98.7 mmol, 60.0 equiv.) was added before the reaction mixture was allowed to warm to r.t. and stirred vigorously for 14 h. Then, water was added, the layers were separated and the aqueous layer was extracted thrice with CH_2Cl_2 (3 × 25 mL). The combined organic layers were washed with brine and dried over Na_2SO_4. The solvents were removed under reduced pressure and the crude product was subjected to FCC (SiO_2, 50 → 100% EtOAc/hexanes) to yield 6-iodochromane-2-carboxamide (*rac*-**1e**) as a white solid (412 mg, 1.36 mmol, 83%).

TLC (50% EtOAc/hexanes): R_f = 0.41 [UV, $KMnO_4$].

M.p.: 194 °C

^{1}H-NMR (400 MHz, $CDCl_3$, 300 K): δ [ppm] = 7.42 – 7.39 (m, 2H, H5, H7), 6.65 (d, ^3J = 9.2 Hz, 1H, H8), 6.50 (bs, 1H, NH_2^a), 5.52 (bs, 1H, NH_2^b), 4.53 (dd, ^3J = 9.3 Hz, ^3J = 3.1 Hz, 1H, H2),

2.88 – 2.73 (m, 2H, H4), 2.39 (dddd, ^2J = 13.8 Hz, ^3J = 5.8 Hz, ^3J = 5.0 Hz, ^3J = 3.1 Hz, 1H, H3[a]), 2.06 (*virt.* dtd, ^2J = 13.8 Hz, $^3J \approx {}^3J$ = 9.6 Hz, ^3J = 5.5 Hz, 1H, H3[b]).

^{13}C-NMR (101 MHz, $CDCl_3$, 300 K): δ [ppm] = 173.0 ($CONH_2$), 153.0 (C8a), 138.5 (C5), 136.6 (C7), 125.0 (C4a), 119.6 (C8), 83.7 (C6), 75.6 (C2), 24.4 (C3), 23.8 (C4).

HRMS (ESI) *m/z* $[M+H]^+$ calculated for $[C_{10}H_{11}{}^{127}INO_2]^+$: 303.9829; found: 303.9829.

IR (film) $\tilde{\nu}_{max}/cm^{-1}$ = 3380 (m, NH), 3167 (m, NH), 2972 (w, CH_{arom}), 2935 (w, CH_{aliph}), 2870 (w, CH_{aliph}), 2839 (w, CH_{aliph}), 1657 (s, C=O), 1618 (m, C=C), 1472 (m, CH_{arom}), 1439 (m, CH_{aliph}), 1229 (s, C–O), 1175 (s, C–N), 1108 (s, C–O), 1063 (m, C–I), 1012 (m, C–O).

6-Methylchromane-2-carboxamide (*rac*-**1f**)

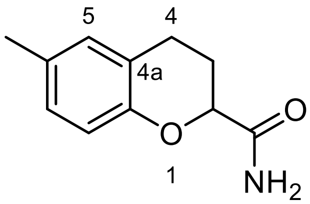

rac-**1f**
$C_{11}H_{13}NO_2$
MW = 191.23 gmol^{-1}

According to GP E, HOBt · H_2O (120 mg, 780 µmol, 1.20 equiv.) was added to a solution of 6-methylchromane-2-carboxylic acid (125 mg, 650 µmol, 1.00 equiv.) in CH_2Cl_2 (7 mL) at 0 °C. After 10 min, EDCI · HCl (150 mg, 780 µmol, 1.20 equiv.) was added and after stirring the resulting mixture at 0 °C for another 30 min, aqueous ammonia (14 M, 1.37 g, 2.79 mL, 39.0 mmol, 60.0 equiv.) was added before the reaction mixture was allowed to warm to r.t. and stirred vigorously for 16 h. Then, water was added, the layers were separated and the aqueous layer was extracted thrice with CH_2Cl_2 (3 × 10 mL). The combined organic layers were washed with brine and dried over Na_2SO_4. The solvents were removed under reduced pressure and the crude product was subjected to FCC (SiO_2, 50 → 100% EtOAc/hexanes) to yield 6-methylchromane-2-carboxamide (*rac*-**1f**) as a white solid (101 mg, 530 µmol, 81%).

TLC (50% EtOAc/hexanes): R_f = 0.40 [UV, $KMnO_4$].

M.p.: 168 °C

^{1}H-NMR (400 MHz, $CDCl_3$, 300 K): δ [ppm] = 6.93 (dd, ^3J = 8.3 Hz, ^4J = 1.3 Hz, 1H, H7), 6.88 (d, ^4J = 1.3 Hz, 1H, H5), 6.77 (d, ^3J = 8.3 Hz, 1H, H8), 6.58 (bs, 1H, NH_2[a]), 5.82 (bs, 1H, NH_2[b]), 4.51 (dd, ^3J = 9.3 Hz, ^3J = 3.1 Hz, 1H, H2), 2.88 – 2.72 (m, 2H, H4), 2.38 (dddd, ^2J = 13.7 Hz, ^3J = 5.7 Hz, ^3J = 4.9 Hz, ^3J = 3.1 Hz, 1H, H3[a]), 2.26 (s, 3H., CH_3), 2.06 (*virt.* dtd, ^2J = 13.7 Hz, $^3J \approx {}^3J$ = 9.5 Hz, ^3J = 5.6 Hz, 1H, H3[b]).

^{13}C-NMR (101 MHz, $CDCl_3$, 300 K): δ [ppm] = 173.9 ($CONH_2$), 150.9 (C8a), 130.7 (C6), 130.2 (C5), 128.3 (C7), 121.9 (C4a), 116.5 (C8), 75.5 (C2), 24.9 (C3), 24.1 (C4), 20.6 (CH_3).

HRMS (ESI) *m/z* $[M+H]^+$ calculated for $[C_{11}H_{14}NO_2]^+$: 192.1019; found: 192.1019.

IR (film) $\tilde{\nu}_{max}/cm^{-1}$ = 3381 (m, NH), 3181 (m, NH), 3005 (w, CH_{arom}), 2965 (w, CH_{aliph}), 2927 (w, CH_{aliph}), 2855 (w, CH_{aliph}), 1657 (s, C=O), 1616 (m, C=C), 1493 (m, CH_{arom}), 1435 (m, CH_{aliph}), 1219 (m, C–O), 1144 (m, C–N), 1109 (m, C–O), 1015 (m, C–O).

6-Methoxychromane-2-carboxamide (*rac*-**1g**)

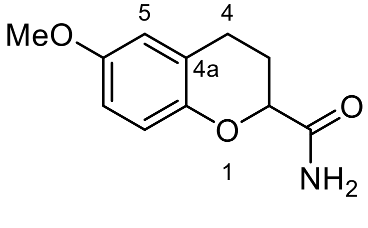

rac-**1g**
$C_{11}H_{13}NO_3$
MW = 207.23 $gmol^{-1}$

According to GP E, HOBt · H_2O (110 mg, 720 μmol, 1.20 equiv.) was added to a solution of 6-methoxychromane-2-carboxylic acid (125 mg, 600 μmol, 1.00 equiv.) in CH_2Cl_2 (6 mL) at 0 °C. After 10 min, EDCI · HCl (138 mg, 720 μmol, 1.20 equiv.) was added and after stirring the resulting mixture at 0 °C for another 30 min, aqueous ammonia (14 M, 1.26 g, 2.57 mL, 36.0 mmol, 60.0 equiv.) was added before the reaction mixture was allowed to warm to r.t. and stirred vigorously for 16 h. Then, water (10 mL) was added, the layers were separated and the aqueous layer was extracted thrice with CH_2Cl_2 (3 × 10 mL). The combined organic layers were washed with brine and dried over Na_2SO_4. The solvents were removed under reduced pressure and the crude product was subjected to FCC (SiO_2, 50 → 100% EtOAc/hexanes) to yield 6-methoxychromane-2-carboxamide (*rac*-**1g**) as a white solid (94.0 mg, 460 μmol, 76%).

TLC (50% EtOAc/hexanes): R_f = 0.26 [UV, $KMnO_4$].

M.p.: 172 °C

^{1}H-NMR (400 MHz, $CDCl_3$, 300 K): δ [ppm] = 6.90 (d, ^3J = 8.9 Hz, 1H, H8), 6.70 (dd, ^3J = 8.9 Hz, ^4J = 3.0 Hz, 1H, H7), 6.61 – 6.58 (m, 2H, H5, NH_2^a), 5.60 (bs, 1H, NH_2^b), 4.50 (dd, ^3J = 9.3 Hz, ^3J = 3.0 Hz, 1H, H2), 3.75 (s, 3H, CH_3), 2.87 (ddd, ^2J = 16.3 Hz, ^3J = 9.8 Hz, ^3J = 5.8 Hz, 1H, $H4^a$), 2.77 (*virt.* dt, ^2J = 16.3 Hz, $^3J \approx {}^3J$ = 5.2 Hz, 1H, $H4^b$), 2.38 (dddd, ^2J = 13.7 Hz, ^3J = 5.9 Hz, ^3J = 4.8 Hz, ^3J = 3.0 Hz, 1H, $H3^a$), 2.06 (*virt.* dtd, ^2J = 13.7 Hz, $^3J \approx {}^3J$ = 9.6 Hz, ^3J = 5.6 Hz, 1H, $H3^b$).

^{13}C-NMR (101 MHz, $CDCl_3$, 300 K): δ [ppm] = 173.8 ($CONH_2$), 154.2 (C8a), 147.1 (C6), 122.8 (C4a), 117.4 (C8), 114.3 (C5), 113.9 (C7), 75.5 (C2), 55.9 (CH_3), 24.9 (C3), 24.5 (C4).

HRMS (ESI) *m/z* $[M+H]^+$ calculated for $[C_{11}H_{14}NO_3]^+$: 208.0968; found: 208.0963.

IR (film) $\tilde{\nu}_{max}/cm^{-1}$ = 3370 (m, NH), 3159 (m, NH), 3001 (w, CH_{arom}), 2970 (w, CH_{aliph}), 2913 (w, CH_{aliph}), 2863 (w, CH_{aliph}), 1658 (m, C=O), 1588 (m, C=C), 1493 (m, CH_{arom}), 1430 (m, CH_{aliph}), 1210 (m, C–O), 1149 (m, C–N), 1110 (m, C–O), 1037 (m, C–O).

6-Phenylchromane-2-carboxamide (*rac*-**1h**)

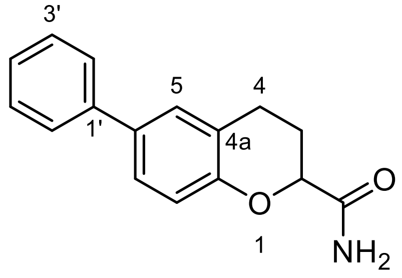

rac-**1h**
$C_{16}H_{15}NO_2$
MW = 253.30 $gmol^{-1}$

A mixture of 6-bromochromane-2-carboxamide (*rac*-**1d**) (100 mg, 390 μmol, 1.00 equiv.), phenylboronic acid (95.2 mg, 781 μmol, 2.00 equiv.), $Pd(PPh_3)_4$ (22.6 mg, 19.5 μmol, 5.0 mol%), K_2CO_3 (270 mg, 1.95 mmol, 5.00 equiv.) in 1,2-dimethoxyethane (1.0 mL) and water (1.0 mL) was degassed by being sparged with argon for 15 min. The suspension was then stirred at 80 °C for 14 h before it was allowed to cool to r.t. again. Water (5 mL) was added, the layers were separated and the aqueous layer was extracted thrice with EtOAc (3 × 5 mL). The combined organic layers were washed with brine and dried over Na_2SO_4 before the remaining solvents were removed under reduced pressure. The obtained crude product was subjected to FCC (SiO_2, 50 → 100% EtOAc/hexanes) to yield 6-phenylchromane-2-carboxamide (*rac*-**1h**) as a white solid (79.0 mg, 312 μmol, 80%).

TLC (50% EtOAc/hexanes): R_f = 0.55 [UV, $KMnO_4$].

M.p.: 183 °C

^{1}H-NMR (400 MHz, $CDCl_3$, 300 K): δ [ppm] = 7.54 – 7.52 (m, 2H, H2'), 7.43 – 7.36 (m, 3H, H5, H3'), 7.33 – 7.29 (m, 2H, H7, H4'), 6.96 (d, ^3J = 8.4 Hz, 1H, H8), 6.60 (bs, 1H, NH_2^a), 5.63 (bs, 1H, NH_2^b), 4.59 (dd, ^3J = 9.3 Hz, ^4J = 3.1 Hz, 1H, H2), 2.99 – 2.83 (m, 2H, H4), 2.45 (dddd, ^2J = 13.8 Hz, ^3J = 5.8 Hz, ^3J = 4.9 Hz, ^3J = 3.1 Hz, 1H, $H3^a$), 2.13 (*virt.* dtd, ^2J = 13.8 Hz, $^3J \approx {}^3J$ = 9.5 Hz, ^3J = 5.5 Hz, 1H, $H3^b$).

^{13}C-NMR (101 MHz, $CDCl_3$, 300 K): δ [ppm] = 173.5 ($CONH_2$), 152.6 (C8a), 140.8 (C1'), 134.8 (C6), 129.0 (C3'), 128.6 (C7), 127.0 (C4'), 126.9 (C2'), 126.5 (C5), 122.5 (C4a), 117.1 (C8), 75.7 (C2), 24.9 (C3), 24.3 (C4).

HRMS (ESI) *m/z* $[M+H]^+$ calculated for $[C_{16}H_{16}NO_2]^+$: 254.1176; found: 254.1167.

IR (film) $\tilde{\nu}_{max}$/cm^{-1} = 3388 (w, NH), 3170 (m, NH), 3029 (w, CH_{arom}), 2964 (m, CH_{aliph}), 2930 (m, CH_{aliph}), 2851 (w, CH_{aliph}), 1659 (s, C=O), 1611 (m C=C), 1481 (m, CH_{arom}), 1435 (m, CH_{aliph}), 1232 (s, C–O), 1173 (m, C–N).

6-Ethylchromane-2-carboxamide (*rac*-**1i**)

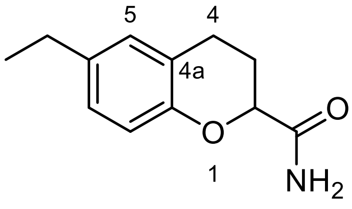

rac-**1i**
$C_{12}H_{15}NO_2$
MW = 205.26 gmol^{-1}

According to GP E, HOBt · H_2O (70.8 mg, 463 µmol, 1.20 equiv.) was added to a solution of 6-ethylchromane-2-carboxylic acid (*rac*-**S-3i**) (79.5 mg, 385 mmol, 1.00 equiv.) in CH_2Cl_2 (13 mL) at 0 °C. After 10 min, EDCI · HCl (88.7 mg, 463 µmol, 1.20 equiv.) was added and after stirring the resulting mixture at 0 °C for another 30 min, aqueous ammonia (14 M, 384 mg, 1.65 mL, 23.1 mmol, 60.0 equiv.) was added before the reaction mixture was allowed to warm to r.t. and stirred vigorously for 14 h. Then, water (5 mL) was added, the layers were separated and the aqueous layer was extracted thrice with CH_2Cl_2 (3 × 10 mL). The combined organic layers were washed with brine and dried over Na_2SO_4. The solvents were removed under reduced pressure and the crude product was subjected to FCC (SiO_2, 50 → 100% EtOAc/hexanes) to yield 6-ethylchromane-2-carboxamide (*rac*-**1i**) as a white solid (67.9 mg, 331 µmol, 86%).

TLC (50% EtOAc/pentane): R_f = 0.23 [UV, $KMnO_4$].

M.p.: 153 °C

^{1}H-NMR (400 MHz, $CDCl_3$, 300 K): δ [ppm] = 6.96 (dd, ^3J = 8.3 Hz, ^4J = 2.2 Hz, 1H, H7), 6.90 (d, ^4J = 2.2 Hz, 1H, H5), 6.80 (d, ^3J = 8.3 Hz, 1H, H8), 6.60 (bs, 1H, NH_2^a), 5.87 (bs, 1H, NH_2^b), 4.52 (dd, ^3J = 9.3 Hz, ^3J = 3.1 Hz, 1H, H2), 2.90 – 2.73 (m, 2H, H4), 2.56 (q, ^3J = 7.6 Hz, 2H, CH_2CH$_3$), 2.39 (dddd, ^2J = 13.7 Hz, ^3J = 5.7 Hz, ^3J = 4.9 Hz, ^3J = 3.1 Hz, 1H, H3^a), 2.06 (*virt.* ddd, ^2J = 13.7 Hz, $^3J \approx {}^3J$ = 9.6 Hz, ^3J = 5.6 Hz, 1H, H3^b), 1.20 (t, ^3J = 7.6 Hz, 3H, CH$_2$CH_3).

^{13}C-NMR (101 MHz, $CDCl_3$, 300 K): δ [ppm] = 173.9 ($CONH_2$), 151.0 (C8a), 137.3 (C6), 129.0 (C5), 127.1 (C7), 121.9 (C4a), 116.5 (C8), 75.5 (C2), 28.1 (CH_2CH_3), 25.0 (C3), 24.2 (C4), 15.9 (CH_3).

HRMS (ESI) *m/z* $[M+H]^+$ calculated for $[C_{12}H_{16}NO_2]^+$: 206.1176; found: 206.1174.

IR (film) $\tilde{\nu}_{max}$/cm^{-1} = 3377 (m, NH), 3176 (m, NH), 3007 (w, CH_{arom}), 2954 (w, CH_{aliph}), 2928 (w, CH_{aliph}), 2873 (m, CH_{aliph}), 2854 (m, CH_{aliph}), 1658 (s, C=O), 1623 (m, C=C), 1496 (m, CH_{arom}), 1435 (m, CH_{aliph}), 1219 (s, C–O), 1126 (m, C–N), 1078 (s, C–O).

5-Methylchromane-2-carboxamide (*rac*-1j)

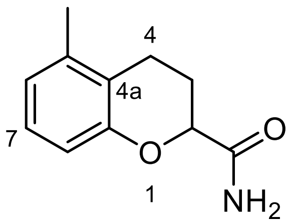

rac-**1j**
$C_{11}H_{13}NO_2$
MW = 191.23 gmol^{-1}

According to GP E, HOBt · H_2O (62.1 mg, 406 µmol, 1.20 equiv.) was added to a solution of 5-methylchromane-2-carboxylic acid (*rac*-**S-3j**) (65.0 mg, 338 mmol, 1.00 equiv.) in CH_2Cl_2 (11 mL) at 0 °C. After 10 min, EDCI · HCl (77.8 mg, 406 µmol, 1.20 equiv.) was added and after stirring the resulting mixture at 0 °C for another 30 min, aqueous ammonia (14 M, 346 mg, 1.45 mL, 20.3 mmol, 60.0 equiv.) was added before the reaction mixture was allowed to warm to r.t. and stirred vigorously for 14 h. Then, water (5 mL) was added, the layers were separated and the aqueous layer was extracted thrice with CH_2Cl_2 (3 × 10 mL). The combined organic layers were washed with brine and dried over Na_2SO_4. The solvents were removed under reduced pressure and the crude product was subjected to FCC (SiO_2, 50 → 100% EtOAc/hexanes) to yield 5-methylchromane-2-carboxamide (*rac*-**1j**) as a white solid (52.0 mg, 272 µmol, 80%).

TLC (60% EtOAc/pentane): R_f = 0.18 [UV, $KMnO_4$].

M.p.: 150 °C

^{1}H-NMR (400 MHz, $CDCl_3$, 300 K): δ [ppm] = 7.04 (virt. t, $^3J \approx {}^3J$ = 7.8 Hz, 1H, H7), 6.80 – 6.74 (m, 2H, H6, H8), 6.59 (bs, 1H, $NH_2{}^a$), 5.89 (bs, 1H, $NH_2{}^b$), 4.48 (dd, ^3J = 9.5 Hz, ^3J = 2.9 Hz, 1H, H2), 2.73 – 2.69 (m, 2H, H4), 2.46 (*virt.* dtd, ^2J = 13.7 Hz, $^3J \approx {}^3J$ = 5.4 Hz, ^3J = 2.9 Hz, 1H, H3^a), 2.22 (s, 3H, CH_3), 2.06 (dddd, ^2J = 13.7 Hz, ^3J = 9.5 Hz, ^3J = 8.4 Hz, ^3J = 7.3 Hz, 1H, H3^b).

^{13}C-NMR (101 MHz, $CDCl_3$, 300 K): δ [ppm] = 173.9 ($CONH_2$), 153.1 (C8a), 137.9 (C5), 127.1 (C7), 122.9 (C6), 121.0 (C4a), 114.6 (C8), 75.0 (C2), 25.0 (C3), 21.9 (C4), 19.2 (CH_3).

HRMS (ESI) *m/z* $[M+H]^+$ calculated for $[C_{11}H_{14}NO_2]^+$: 192.1019; found: 192.1019.

IR (film) $\tilde{\nu}_{max}/cm^{-1}$ = 3386 (m, NH), 3188 (m, NH), 3070 (w, CH_{arom}), 3036 (w, CH_{arom}), 2962 (w, CH_{aliph}), 2929 (w, CH_{aliph}), 2858 (m, CH_{aliph}), 1655 (s, C=O), 1609 (m, C=C), 1467 (m, CH_{arom}), 1250 (m, C–O), 1183 (w, C–N), 1100 (m, C–O).

7-Methylchromane-2-carboxamide (*rac*-**1k**)

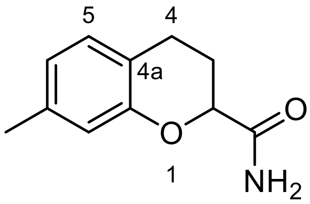

rac-**1k**
$C_{11}H_{13}NO_2$
MW = 191.23 gmol^{-1}

According to GP E, HOBt · H_2O (153 mg, 999 µmol, 1.20 equiv.) was added to a solution of 7-methylchromane-2-carboxylic acid (*rac*-**S-3k**) (160 mg, 832 mmol, 1.00 equiv.) in CH_2Cl_2 (28 mL) at 0 °C. After 10 min, EDCI · HCl (191 mg, 999 µmol, 1.20 equiv.) was added and after stirring the resulting mixture at 0 °C for another 30 min, aqueous ammonia (14 M, 851 mg, 3.57 mL, 49.9 mmol, 60.0 equiv.) was added before the reaction mixture was allowed to warm to r.t. and stirred vigorously for 14 h. Then, water (10 mL) was added, the layers were separated and the aqueous layer was extracted thrice with CH_2Cl_2 (3 × 20 mL). The combined organic layers were washed with brine and dried over Na_2SO_4. The solvents were removed under reduced pressure and the crude product was subjected to FCC (SiO_2, 50 → 100% EtOAc/hexanes) to yield 7-methylchromane-2-carboxamide (*rac*-**1k**) as a white solid (144 mg, 753 µmol, 90%).

TLC (80% EtOAc/pentane): R_f = 0.49 [UV, $KMnO_4$].

M.p.: 178 °C

^{1}H-NMR (400 MHz, $CDCl_3$, 300 K): δ [ppm] = 6.95 (d, ^3J = 7.5 Hz, 1H, H5), 6.74 – 6.71 (m, 2H, H6, H8), 6.57 (bs, 1H, NH_2^a), 5.96 (bs, 1H, NH_2^b), 4.52 (dd, ^3J = 9.3 Hz, ^3J = 3.1 Hz, 1H, H2), 2.87 – 2.71 (m, 2H, H4), 2.38 (dddd, ^2J = 13.7 Hz, ^3J = 5.8 Hz, ^3J = 5.0 Hz, ^3J = 3.1 Hz, 1H, H3^a), 2.29 (s, 3H, CH_3), 2.06 (*virt.* dtd, ^2J = 13.7 Hz, $^3J \approx {}^3J$ = 9.5 Hz, ^3J = 5.6 Hz, 1H, H3^b).

^{13}C-NMR (101 MHz, $CDCl_3$, 300 K): δ [ppm] = 173.9 ($CONH_2$), 152.8 (C8a), 137.7 (C7), 129.6 (C5), 122.4 (C6), 119.1 (C4a), 117.1 (C8), 75.5 (C2), 25.0 (C3), 23.8 (C4), 21.2 (CH_3).

HRMS (ESI) *m/z* $[M+H]^+$ calculated for $[C_{11}H_{14}NO_2]^+$: 192.1019; found: 192.1017.

IR (film) $\tilde{\nu}_{max}$/cm^{-1} = 3360 (m, NH), 3179 (w, NH), 2957 (w, CH_{aliph}), 2921 (w, CH_{aliph}), 2852 (m, CH_{aliph}), 1658 (s, C=O), 1506 (w, CH_{arom}), 1445 (w, CH_{aliph}), 1250 (m, C–O), 1117 (w, C–N), 1076 (w, C–O).

8-Methylchromane-2-carboxamide (*rac*-1l)

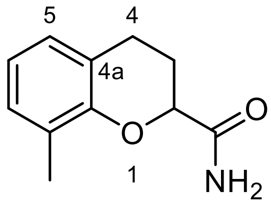

rac-**1l**
$C_{11}H_{13}NO_2$
MW = 191.23 gmol^{-1}

According to GP C, a mixture of 8-methylchromane-2-carboxylic acid ethyl ester (*rac*-**S-2l**) (164 mg, 745 µmol, 1.00 equiv.), NH_4Cl (9.56 mg, 179 µmol, 0.24 equiv.) and aqueous ammonia (14 M, 507 mg, 2.13 mL, 29.8 mmol, 40.0 equiv.) was stirred at 100 °C for 5 h. After allowing the mixture to cool to r.t., water (2 mL) and CH_2Cl_2 (3 mL) were added and the aqueous and the organic layers were separated. Then, the aqueous layer was extracted thrice with CH_2Cl_2 (3 × 3 mL) and the combined organic layers were washed with brine and dried over Na_2SO_4. The remaining solvents were removed under reduced pressure and the crude products were subjected to FCC (SiO_2, 50 → 100% EtOAc/hexanes) to yield 7-methoxychromane-2-carboxamide (*rac*-**1l**) as a white solid (55.8 mg, 292 µmol, 39%).

TLC (60% EtOAc/pentane): R_f = 0.38 [UV, $KMnO_4$].

M.p.: 140 °C

^{1}H-NMR (400 MHz, $CDCl_3$, 300 K): δ [ppm] = 7.00 (d, ^3J = 7.4 Hz, 1H, H7), 6.93 (d, ^3J = 7.4 Hz, 1H, H5), 6.81 (*virt.* t, $^3J \approx {}^3J$ = 7.4 Hz, 1H, H6), 6.57 (bs, 1H, NH_2^a), 6.5.89 (bs, 1H, NH_2^b), 4.56 (dd, ^3J = 9.6 Hz, ^3J = 3.0 Hz, 1H, H2), 2.94 – 2.76 (m, 2H, H4), 2.42 (dddd, ^2J = 13.5 Hz, ^3J = 5.8 Hz, ^3J = 4.5 Hz, ^3J = 3.0 Hz, 1H, $H3^a$), 2.06 (*virt.* dtd, ^2J = 13.5 Hz, $^3J \approx {}^3J$ = 9.9 Hz, ^3J = 5.5 Hz, 1H, $H3^b$),

^{13}C-NMR (101 MHz, $CDCl_3$, 300 K): δ [ppm] = 173.9 ($CONH_2$), 151.2 (C8a), 128.9 (C7), 127.5 (C5), 125.6 (C8), 121.6 (C4a), 120.29 (C6), 75.7 (C2), 25.0 (C3), 24.4 (C4), 16.2 (C8CH$_3$).

HRMS (ESI) *m/z* $[M+H]^+$ calculated for $[C_{11}H_{14}NO_2]^+$: 192.1019; found: 192.1020.

IR (film) $\tilde{\nu}_{max}/cm^{-1}$ = 3401 (m, NH), 3179 (m, NH), 3052 (w, CH_{arom}), 2961 (m, CH_{aliph}), 2948 (m, CH_{aliph}), 2922 (m, CH_{aliph}), 2852 (m, CH_{aliph}), 1656 (s, C=O), 1611 (m, C=C), 1595 (m, CH_{arom}), 1444 (m, CH_{aliph}), 1215 (s, C–O), 1176 (s, C–N), 1101 (s, C–O).

6,7,8,9-Tetrahydro-benzo[*g*]chromane-2-carboxamid (*rac*-**1m**)

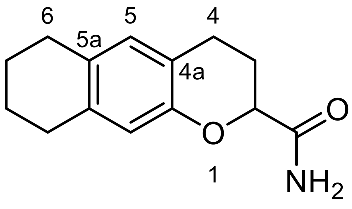

rac-**1m**
$C_{14}H_{17}NO_2$
MW = 231.30 gmol^{-1}

According to GP E, HOBt · H_2O (35.6 mg, 232 µmol, 1.20 equiv.) was added to a solution of 6,7,8,9-tetrahydro-4-oxo-4*H*-benzo[*g*]chromane-2-carboxylic acid (*rac*-**S-3m**) (45.0 mg, 194 µmol, 1.00 equiv.) in CH_2Cl_2 (6.5 mL) at 0 °C. After 10 min, EDCI · HCl (44.6 mg, 232 mmol, 1.20 equiv.) was added and after stirring the resulting mixture at 0 °C for another 30 min, aqueous ammonia (14 M, 198 mg, 830 µL, 11.6 mmol, 60.0 equiv.) was added before the reaction mixture was allowed to warm to r.t. and stirred vigorously for 14 h. Then, water (2 mL) was added, the layers were separated and the aqueous layer was extracted thrice with CH_2Cl_2 (3 × 10 mL). The combined organic layers were washed with brine and dried over Na_2SO_4. The solvents were removed under reduced pressure and the crude product was subjected to FCC (SiO_2, 50 → 100% EtOAc/hexanes) to yield 6,7,8,9-Tetrahydro-4-oxo-4*H*-benzo[*g*]chromene-2-carboxamid (*rac*-**1m**) as a white solid (33.8 mg, 146 µmol, 76%).

TLC (60% EtOAc/pentane): R_f = 0.34 [UV, $KMnO_4$].

M.p.: 196 °C

^{1}H-NMR (400 MHz, $CDCl_3$, 300 K): δ [ppm] = 6.78 (s, 1H, H5), 6.61 – 6.58 (m, 2H, H8, NH_2^a), 5.81 (bs, 1H, NH_2^b), 4.50 (dd, ^3J = 9.1 Hz, ^3J = 3.1 Hz, 1H, H2), 2.85 – 2.66 (m, 6H, H4, H6, H9), 2.36 (*virt.* dtd, ^2J = 14.2 Hz, $^3J \approx {}^3J$ = 5.4 Hz, ^3J = 3.1 Hz, 1H, H3^a), 2.16 (*virt.* dtd, ^2J = 14.2 Hz, $^3J \approx {}^3J$ = 9.3 Hz, ^3J = 5.5 Hz, 1H, H3^b), 1.78 – 1.75 (m, 4H, H7, H8).

^{13}C-NMR (101 MHz, $CDCl_3$, 300 K): δ [ppm] = 174.0 ($CONH_2$), 150.7 (C8a), 136.7 (C9a), 130.2 (C5a), 130.0 (C5), 119.5 (C4a), 116.4 (C8), 75.4 (C2), 29.3 (C6/C9), 28.7 (C6/C9), 25.1 (C3), 23.8 (C4), 23.5 (C7/C8), 23.3 (C7/C8).

HRMS (ESI) *m/z* $[M+H]^+$ calculated for $[C_{14}H_{18}NO_2]^+$: 232.1332; found: 232.1331.

IR (film) $\tilde{\nu}_{max}$/cm^{-1} = 3379 (m, NH), 3164 (m, NH), 3011 (w, CH_{arom}), 2958 (w, CH_{aliph}), 2932 (m, CH_{aliph}), 2849 (w, CH_{aliph}), 1657 (s, C=O), 1619 (m, C=C), 1505 (m, CH_{arom}), 1433 (m, CH_{aliph}), 1262 (m, C–O), 1169 (m, C–N), 1109 (s, C–O).

6-(Pyridine-3-yl)chromane-2-carboxamide (*rac*-1n)

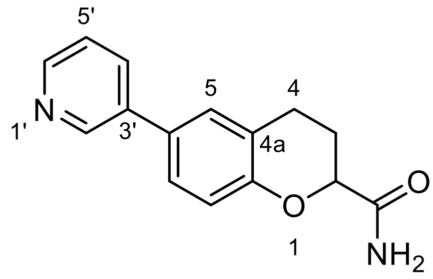

rac-**1n**
$C_{15}H_{14}N_2O_2$
MW = 254.29 gmol^{-1}

A mixture of 6-bromochromane-2-carboxamide (*rac*-**1d**) (100 mg, 390 µmol, 1.00 equiv.), 3-pyridineboronic acid (96.0 mg, 781 µmol, 2.00 equiv.), $Pd(PPh_3)_4$ (22.6 mg, 19.5 µmol, 5.0 mol%), K_2CO_3 (270 mg, 1.95 mmol, 5.00 equiv.) in 1,2-dimethoxyethane (1.0 mL) and water (1.0 mL) was degassed by being sparged with argon for 15 min. The suspension was then stirred at 80 °C for 14 h before it was allowed to cool to r.t. again. Water (5 mL) was added, the layers were separated and the aqueous layer was extracted thrice with EtOAc (3 × 5 mL). The combined organic layers were washed with brine and dried over Na_2SO_4 before the remaining solvents were removed under reduced pressure. The obtained crude product was subjected to FCC (SiO_2, 70 → 100% EtOAc/hexanes) to yield 6-(pyridine-3-yl)chromane-2-carboxamide (*rac*-**1n**) as a white solid (57.0 mg, 224 µmol, 57%).

TLC (90% EtOAc/hexanes): R_f = 0.15 [UV, $KMnO_4$].

M.p.: 160 °C

^{1}H-NMR (500 MHz, $CDCl_3$, 300 K): δ [ppm] = 8.79 (dd, ^3J = 2.4 Hz, ^4J = 0.9 Hz, 1H, H6'), 8.55 (dd, ^3J = 4.8 Hz, ^4J = 1.6 Hz, 1H, H2'), 7.81 (ddd, ^3J = 7.6 Hz, ^3J = 2.4 Hz, ^4J = 1.6 Hz, 1H, H5'), 7.37 – 7.30 (m, 3H, H5, H7, H4'), 6.99 (d, ^3J = 8.4 Hz, 1H, H8), 6.60 (bs, 1H, NH_2^a), 5.65 (bs, 1H, NH_2^b), 4.60 (dd, ^3J = 9.3 Hz, ^4J = 3.1 Hz, 1H, H2), 3.00 – 2.85 (m, 2H, H4), 2.46 (*virt.* dtd, ^2J = 13.7 Hz, $^3J \approx {}^3J$ = 5.3 Hz, ^3J = 3.1 Hz, 1H, $H3^a$), 2.13 (*virt.* dtd, ^2J = 13.7 Hz, $^3J \approx {}^3J$ = 9.5 Hz, ^3J = 5.5 Hz, 1H, $H3^b$).

^{13}C-NMR (126 MHz, $CDCl_3$, 300 K): δ [ppm] = 173.2 ($CONH_2$), 153.3 (C8a), 148.2 (C2'/C6'), 148.2 (C2'/C6'), 136.2 (C3'), 134.0 (C5'), 131.3 (C6), 128.6 (C5), 126.7 (C7), 123.6 (C4'), 122.9 (C4a), 117.5 (C8), 75.7 (C2), 24.8 (C3), 24.3 (C4).

HRMS (ESI) *m/z* $[M+H]^+$ calculated for $[C_{15}H_{15}N_2O_2]^+$: 255.1128; found: 255.1119.

IR (film) $\tilde{\nu}_{max}$/cm^{-1} = 3381 (m, NH), 3172 (m, NH), 3028 (w, CH_{arom}), 2925 (m, CH_{aliph}), 2851 (w, CH_{aliph}), 1658 (s, C=O), 1612 (m C=C), 1475 (m, CH_{arom}), 1432 (m, CH_{aliph}), 1236 (s, C–O), 1174 (m, C–N).

7-Methoxychromane-2-carboxamide (*rac*-**1o**)

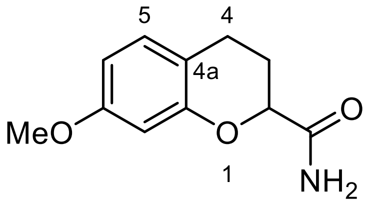

rac-**1o**
$C_{11}H_{13}NO_3$
MW = 207.23 gmol^{-1}

According to GP C, a mixture of 7-methoxychromane-2-carboxylic acid ethyl ester (*rac*-**S-2o**) (117 mg, 495 µmol, 1.00 equiv.), NH_4Cl (6.36 mg, 118 µmol, 0.24 equiv.) and aqueous ammonia (14 M, 337 mg, 1.41 mL, 19.8 mmol, 40.0 equiv.) was stirred at 100 °C for 5 h. After allowing the mixture to cool to r.t., water (2 mL) and CH_2Cl_2 (3 mL) were added and the aqueous and the organic layers were separated. Then, the aqueous layer was extracted thrice with CH_2Cl_2 (3 × 3 mL) and the combined organic layers were washed with brine and dried over Na_2SO_4. The remaining solvents were removed under reduced pressure and the crude products were subjected to FCC (SiO_2, 50 → 100% EtOAc/hexanes) to yield 7-methoxychromane-2-carboxamide (*rac*-**1o**) as a white solid (34.2 mg, 165 µmol, 33%).

TLC (50% EtOAc/pentane): R_f = 0.22 [UV, $KMnO_4$].

M.p.: 165 °C

^{1}H-NMR (400 MHz, $CDCl_3$, 300 K): δ [ppm] = 6.96 (d, ^3J = 8.4 Hz, 1H, H5), 6.56 (bs, 1H, $NH_2{}^a$), 6.50 (dd, ^3J = 8.4 Hz, ^4J = 2.5 Hz, 1H, H6), 6.44 (d, ^4J = 2.5 Hz, 1H, H8), 5.93 (bs, 1H, $NH_2{}^b$), 4.52 (dd, ^3J = 9.3 Hz, ^3J = 3.1 Hz, 1H, H2), 3.77 (s, 3H, CH_3), 2.84 – 2.69 (m, 2H, H4), 2.38 (*virt.* dtd, ^2J = 14.2 Hz, $^3J \approx {}^3J$ = 5.5 Hz, ^3J = 3.1 Hz, 1H, H3^a), 2.05 (*virt.* dtd, ^2J = 14.2 Hz, $^3J \approx {}^3J$ = 9.3 Hz, ^3J = 5.5 Hz, 1H, H3^b).

^{13}C-NMR (101 MHz, $CDCl_3$, 300 K): δ [ppm] = 173.8 ($CONH_2$), 159.4 (C7), 153.7 (C8a), 130.3 (C5), 114.2 (C4a), 108.1 (C6), 101.9 (C8), 75.6 (C2), 55.5 (CH_3), 25.0 (C3), 23.4 (C4).

HRMS (ESI) *m/z* $[M+H]^+$ calculated for $[C_{11}H_{14}NO_3]^+$: 208.0968; found: 208.0970.

IR (film) $\tilde{\nu}_{max}/cm^{-1}$ = 3317 (m, NH), 3155 (m, NH), 3013 (w, CH_{arom}), 2956 (m, CH_{aliph}), 2923 (m, CH_{aliph}), 2851 (m, CH_{aliph}), 1660 (s, C=O), 1619 (m, C=C), 1505 (m, CH_{arom}), 1441 (m, CH_{aliph}), 1253 (m, C–O), 1206 (m, C–O), 1155 (s, C–N), 1109 (s, C–O).

7-Fluorochromane-2-carboxamide (*rac*-**1p**)

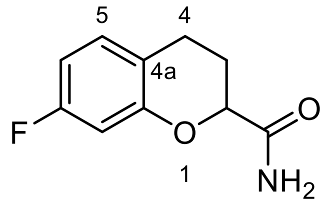

rac-**1p**
$C_{10}H_{10}FNO_2$
MW = 195.19 gmol^{-1}

According to GP C, a mixture of 7-fluorochromane-2-carboxylic acid ethyl ester (*rac*-**S-2p**) (80.2 mg, 358 µmol, 1.00 equiv.), NH_4Cl (4.59 mg, 85.8 µmol, 0.24 equiv.) and aqueous ammonia (14 M, 244 mg, 1.02 mL, 14.3 mmol, 40.0 equiv.) was stirred at 100 °C for 5 h. After allowing the mixture to cool to r.t., water (2 mL) and CH_2Cl_2 (3 mL) were added and the aqueous and the organic layers were separated. Then, the aqueous layer was extracted thrice with CH_2Cl_2 (3 × 3 mL) and the combined organic layers were washed with brine and dried over Na_2SO_4. The remaining solvents were removed under reduced pressure and the crude products were subjected to FCC (SiO_2, 50 → 100% EtOAc/hexanes) to yield 7-fluorochromane-2-carboxamide (*rac*-**1p**) as a white solid (21.3 mg, 109 µmol, 31%).

TLC (60% EtOAc/pentane): R_f = 0.27 [UV, $KMnO_4$].

M.p.: 125 °C

^{1}H-NMR (400 MHz, $CDCl_3$, 300 K): δ [ppm] = 7.03 – 6.99 (m, 1H, H5), 6.66 – 6.59 (m, 2H, H6, H8), 6.52 (s, 1H, NH_2^a), 5.78 (s, 1H, NH_2^b), 4.54 (dd, ^3J = 9.3 Hz, ^3J = 3.1 Hz, 1H, H2), 2.87 – 2.72 (m, 2H, H4), 2.40 (*virt.* dtd, ^2J = 13.7 Hz, $^3J \approx {}^3J$ = 5.3 Hz, ^3J = 3.1 Hz, H3^a), 2.06 (*virt.* dtd, ^2J = 13.7 Hz, $^3J \approx {}^3J$ = 9.6 Hz, ^3J = 5.6 Hz, H3^b).

^{13}C-NMR (101 MHz, $CDCl_3$, 300 K): δ [ppm] = 173.2 ($CONH_2$), 162.1 ($^1J_{CF}$ = 244 Hz, C7), 153.7 (d, $^3J_{CF}$ = 11.7 Hz, C8a), 130.7 (d, $^3J_{CF}$ = 9.5 Hz, C5), 117.9 (d, $^4J_{CF}$ = 3.3 Hz, C4a), 108.7 (d, $^2J_{CF}$ = 21.6 Hz, C6), 104.0 (d, $^2J_{CF}$ = 24.7 Hz, C8), 75.6 (C2), 24.7 (C3), 23.6 (C4).

^{19}F-NMR (376 MHz, $CDCl_3$, 300 K): δ [ppm] = −114.6 – −114.6 (m).

HRMS (ESI) *m/z* [M+H]$^+$ calculated for $[C_{10}H_{11}FNO_2]^+$: 196.0768; found: 196.0770.

IR (film) $\tilde{\nu}_{max}$/cm^{-1} = 3417 (m, NH), 3156 (m, NH), 3076 (w, CH_{arom}), 3037 (w, CH_{arom}), 2960 (m, CH_{aliph}), 2923 (m, CH_{aliph}), 2854 (m, CH_{aliph}), 1661 (s, C=O), 1595 (m, C=C), 1500 (m, CH_{arom}), 1439 (m, CH_{aliph}), 1259 (s, C–O), 1142 (s, C–F), 1110 (s, C–N).

8-Fluorochromane-2-carboxamide (*rac*-**1q**)

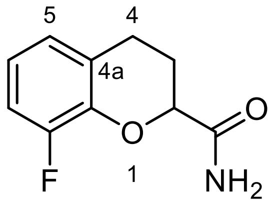

rac-**1q**
$C_{10}H_{10}NO_2$
MW = 195.19 gmol^{-1}

According to GP E, HOBt · H_2O (30.9 mg, 202 μmol, 1.20 equiv.) was added to a solution of 8-fluorochromane-2-carboxylic acid (*rac*-**S-3q**) (33.0 mg, 168 mmol, 1.00 equiv.) in CH_2Cl_2 (5.6 mL) at 0 °C. After 10 min, EDCI · HCl (38.7 mg, 202 μmol, 1.20 equiv.) was added and after stirring the resulting mixture at 0 °C for another 30 min, aqueous ammonia (14 M, 172 mg, 271 μL, 10.1 mmol, 60.0 equiv.) was added before the reaction mixture was allowed to warm to r.t. and stirred vigorously for 14 h. Then, water (2 mL) was added, the layers were separated and the aqueous layer was extracted thrice with CH_2Cl_2 (3 × 10 mL). The combined organic layers were washed with brine and dried over Na_2SO_4. The solvents were removed under reduced pressure and the crude product was subjected to FCC (SiO_2, 50 → 100% EtOAc/hexanes) to yield 8-fluorochromane-2-carboxamide (*rac*-**1q**) as a white solid (29.3 mg, 150 μmol, 89%).

TLC (60% EtOAc/pentane): R_f = 0.29 [UV, $KMnO_4$].

M.p.: 149 °C

^{1}H-NMR (400 MHz, $CDCl_3$, 300 K): δ [ppm] = 6.96 – 6.91 (m, 1H, H7), 6.86 – 6.79 (m, 2H, H5, H6), 6.65 (bs, 1H, NH_2^a), 5.81 (bs, 1H, NH_2^b), 4.58 (dd, ^3J = 9.6 Hz, ^3J = 3.1 Hz, 1H, H2), 2.94 – 2.79 (m, 2H, H4), 2.46 (dddd, ^2J = 13.7 Hz, ^3J = 5.6 Hz, ^3J = 4.6 Hz, ^3J = 3.1 Hz, 1H, H3^a), 2.08 (*virt.* dtd, ^2J = 13.7 Hz, $^3J \approx {}^3J$ = 9.8 Hz, ^3J = 5.6 Hz, 1H, H3^b).

^{13}C-NMR (101 MHz, $CDCl_3$, 300 K): δ [ppm] = 173.0 ($CONH_2$), 151.6 (d, $^1J_{CF}$ = 245 Hz, C8), 141.3 (d, $^2J_{CF}$ = 10.9 Hz, C8a), 124.8 (d, $^3J_{CF}$ = 0.9 Hz, C4a), 124.7 (d, $^4J_{CF}$ = 3.5 Hz, C5), 120.8 (d, $^3J_{CF}$ = 7.2 Hz, C6), 114.2 (d, $^2J_{CF}$ = 17.7 Hz, C7), 75.6 (C2), 24.5 (C3), 24.0 (d, $^4J_{CF}$ = 2.7 Hz, C4).

^{19}F-NMR (376 MHz, $CDCl_3$, 300 K): δ [ppm] = −137.2 – −137.3 (m).

HRMS (ESI) *m/z* $[M+H]^+$ calculated for $[C_{10}H_{11}FNO_2]^+$: 196.0768; found: 196.0769.

IR (film) $\tilde{\nu}_{max}$/cm^{-1} = 3410 (m, NH), 3158 (m, NH), 2959 (w, CH_{aliph}), 2923 (w, CH_{aliph}), 2857 (m, CH_{aliph}), 1658 (s, C=O), 1609 (m, C=C), 1475 (s, CH_{arom}), 1446 (s, CH_{aliph}), 1258 (s, C–O), 1222 (s, C–F), 1176 (m, C–N), 1073 (s, C–O).

1,4-Benzodioxane-2-carboxamide (*rac*-1r)

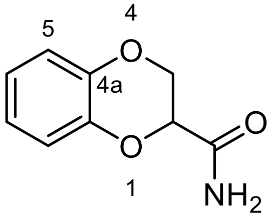

rac-**1r**
$C_9H_9NO_3$
MW = 179.18 gmol^{-1}

According to GP E, HOBt · H_2O (204 mg, 1.33 mmol, 1.20 equiv.) was added to a solution of 1,4-benzodioxane-2-carboxylic acid (200 mg, 1.11 mmol, 1.00 equiv.) in CH_2Cl_2 (37 mL) at 0 °C. After 10 min, EDCI · HCl (255 mg, 1.33 mmol, 1.20 equiv.) was added and after stirring the resulting mixture at 0 °C for another 30 min, aqueous ammonia (14 M, 1.13 g, 4.76 mL, 66.6 mmol, 60.0 equiv.) was added before the reaction mixture was allowed to warm to r.t. and stirred vigorously for 14 h. Then, water was added, the layers were separated and the aqueous layer was extracted thrice with CH_2Cl_2 (3 × 30 mL). The combined organic layers were washed with brine and dried over Na_2SO_4. The solvents were removed under reduced pressure and the crude product was subjected to FCC (SiO_2, 50 → 100% EtOAc/hexanes) to yield 2,3-dihydrobenzo[*b*][1,4]dioxine-2-carboxamide (*rac*-**1r**) as a white solid (192 mg, 1.07 mmol, 97%).

TLC (80% EtOAc/hexanes): R_f = 0.60 [UV, $KMnO_4$].

^{1}H-NMR (400 MHz, $CDCl_3$, 300 K): δ [ppm] = 6.97 – 6.86 (m, 4H, H5/H6/H7/H8), 6.54 (bs, 1H, NH_2^a), 6.17 (bs, 1H, NH_2^b), 4.69 (dd, ^3J = 7.1 Hz, ^3J = 2.8 Hz, 1H, H2), 4.51 (dd, ^2J = 11.4 Hz, ^3J = 2.8 Hz, 1H, H3^a), 4.23 (dd, ^2J = 11.4 Hz, ^3J = 7.1 Hz, 1H, H3^b).

^{13}C-NMR (101 MHz, $CDCl_3$, 300 K): δ [ppm] = 170.2(CONH$_2$), 143.4 (C4a), 141.7 (C8a), 122.6 (C6/C7), 122.1 (C6/C7), 117.9 (C5/C8), 117.2 (C5/C8), 73.3 (C2), 65.2 (C3).

The spectroscopic data matches the one reported in the literature.[39]

3,3-Dimethyl-1,4-benzodioxane-2-carboxamide (*rac*-1s)

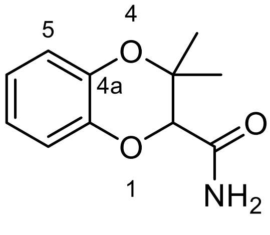

rac-**1s**
$C_{11}H_{13}NO_3$
MW = 207.23 gmol^{-1}

According to GP E, HOBt · H_2O (39.7 mg, 259 μmol, 1.20 equiv.) was added to a solution of 3,3-dimethyl-1,4-benzodioxane-2-carboxylic acid (*rac*-**S-3s**) (45.0 mg, 216 μmol, 1.00 equiv.) in CH_2Cl_2 (7.2 mL) at 0 °C. After 10 min, EDCI · HCl (49.2 mg, 259 μmol, 1.20 equiv.) was added and after stirring the resulting mixture at 0 °C for another 30 min, aqueous ammonia (14 M, 221 mg, 926 μL, 13.0 mmol, 60.0 equiv.) was added before the reaction mixture was allowed to warm to r.t. and stirred vigorously for 14 h. Then, water was added, the layers were separated and the aqueous layer was extracted thrice with CH_2Cl_2 (3 × 10 mL). The combined organic layers were washed with brine and dried over Na_2SO_4. The

solvents were removed under reduced pressure and the crude product was subjected to FCC (SiO_2, 40 → 100% EtOAc/hexanes) to yield 3,3-dimethyl-1,4-benzodioxane-2-carboxamide (*rac*-**1s**) as a white solid (33.0 mg, 159 µmol, 74%).

TLC (40% EtOAc/hexanes): $R_f = 0.31$ [UV, $KMnO_4$].

M.p.: 145°C

^{1}H-NMR (400 MHz, $CDCl_3$, 300 K): δ [ppm] = 6.95 – 6.84 (m, 4H, H5/H6/H7/H8), 6.95 (bs, 1H, NH_2^a), 5.93 (bs, 1H, NH_2^b), 4.27 (s, 1H, H2), 1.63 (s, 3H, CH_3^a), 1.26 (s, 3H, CH_3^b).

^{13}C-NMR (101 MHz, $CDCl_3$, 300 K): δ [ppm] = 170.0 ($CONH_2$), 142.0 (C4a), 140.9 (C8a), 122.9 (C6/C7), 121.3 (C6/C7), 117.8 (C5/C8), 116.7 (C5/C8), 79.7 (C2), 74.5 (C3), 25.5 (CH_3^a), 19.8 (CH_3^b).

HRMS (ESI) *m/z* $[M+H]^+$ calculated for $[C_{11}H_{14}NO_3]^+$: 208.0968; found: 208.0967.

IR (film) $\tilde{\nu}_{max}/cm^{-1}$ = 3476 (w, NH), 3205 (w, NH), 3076 (w, CH_{arom}), 3047 (w, CH_{arom}), 2982 (w, CH_{aliph}), 2937 (w, CH_{aliph}), 1686 (s, C=O), 1598 (m, C=C), 1493 (s, CH_{arom}), 1266 (s, C–O), 1140 (m, C–N), 1108 (m, C–O), 1064 (m, C–O).

2,3-Dihydrobenzo[*b*][1,4]oxazine-2-carboxamide (*rac*-**1t**)

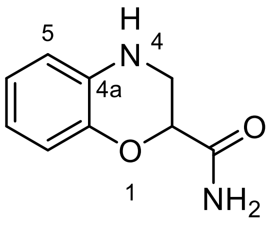

rac-**1t**
$C_9H_{10}N_2O_2$
MW = 178.19 $gmol^{-1}$

According to GP E, HOBt · H_2O (185 mg, 1.21 mmol, 1.20 equiv.) was added to a solution of 2,3-dihydrobenzo[*b*][1,4]oxazine-2-carboxylic acid (180 mg, 1.01 mmol, 1.00 equiv.) in CH_2Cl_2 (10 mL) at 0 °C. After 10 min, EDCI (231 mg, 1.21 mmol, 1.20 equiv.) was added and after stirring the resulting mixture at 0 °C for another 30 min, aqueous ammonia (14 M, 2.11 g, 4.31 mL, 60.3 mmol, 60.0 equiv.) was added before the reaction mixture was allowed to warm to r.t. and stirred vigorously for 16 h. Then, water was added, the layers were separated and the aqueous layer was extracted thrice with CH_2Cl_2 (3 × 15 mL). The combined organic layers were washed with brine and dried over Na_2SO_4. The solvents were removed under reduced pressure and the crude product was subjected to FCC (SiO_2, 50 → 100% EtOAc/hexanes) to yield 2,3-dihydrobenzo[*b*][1,4]oxazine-2-carboxamide (*rac*-**1t**) as a white solid (111 mg, 623 µmol, 62%).

TLC (50% EtOAc/hexanes): $R_f = 0.20$ [UV, $KMnO_4$].

M.p.: 150 °C

^{1}H-NMR (400 MHz, DMSO-d$_6$, 300 K): δ [ppm] = 7.40 – 7.34 (m, 2H, NH$_2$), 6.77 (dd, ^3J = 7.9 Hz, ^4J = 1.5 Hz, 1H, H8), 6.68 (ddd, ^3J = 7.9 Hz, ^3J = 7.2 Hz, ^4J = 1.5 Hz, 1H, H6), 6.58 (dd, ^3J = 7.9 Hz, ^4J = 1.6 Hz, 1H, H5), 6.51 (ddd, ^3J = 7.9 Hz, ^3J = 7.2 Hz, ^4J = 1.6 Hz, 1H, H7), 5.80 (*virt.* t, $^3J \approx {}^3J$ = 2.7 Hz, 1H, NH), 4.43 (dd, ^3J = 7.1 Hz, ^3J = 2.9 Hz, 1H, H2), 3.41 (virt. dt, ^2J = 11.9 Hz, $^3J \approx {}^3J$ = 3.1 Hz, 1H, H3^a), 3.21 (ddd, ^2J = 11.9 Hz, ^3J = 7.1 Hz, ^3J = 2.3 Hz, 1H, H3^b).

^{13}C-NMR (101 MHz, DMSO-d$_6$, 300 K): δ [ppm] = 170.7 (CONH$_2$), 142.0 (C8a), 134.3 (C4a), 121.3 (C6), 117.0 (C7), 116.3 (C8), 114.8 (C5), 73.3 (C2), 41.8 (C3).

The spectroscopic data matches the one reported in the literature.[99]

4-Boc-2,3-Dihydrobenzo[*b*][1,4]oxazine-2-carboxamide (*rac*-**1u**)

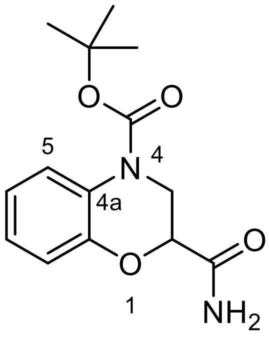

rac-**1u**
$C_{14}H_{18}N_2O_4$
MW = 278.31 gmol^{-1}

According to GP E, HOBt · H_2O (132 mg, 859 µmol, 1.20 equiv.) was added to a solution of 4-Boc-2,3-dihydrobenzo[*b*][1,4]oxazine-2-carboxylic acid (200 mg, 716 µmol, 1.00 equiv.) in CH_2Cl_2 (7 mL) at 0 °C. After 10 min, EDCI (165 mg, 859 µmol, 1.20 equiv.) was added and after stirring the resulting mixture at 0 °C for another 30 min, aqueous ammonia (14 M, 753 mg, 1.53 mL, 21.5 mmol, 30.0 equiv.) was added before the reaction mixture was allowed to warm to r.t. and stirred vigorously for 16 h. Then, water was added, the layers were separated and the aqueous layer was extracted thrice with CH_2Cl_2 (3 × 10 mL). The combined organic layers were washed with brine and dried over Na_2SO_4. The solvents were removed under reduced pressure and the crude product was subjected to FCC (SiO_2, 50 → 100% EtOAc/hexanes) to yield 4-Boc-2,3-dihydrobenzo[*b*][1,4]oxazine-2-carboxamide (*rac*-**1u**) as a white solid (143 mg, 514 µmol, 72%).

TLC (50% EtOAc/hexanes): R_f = 0.55 [UV, $KMnO_4$].

M.p.: 141 °C

^{1}H-NMR (400 MHz, $CDCl_3$, 300 K): δ [ppm] = 7.82 (d, ^3J = 8.1 Hz, 1H, H5), 7.04 – 6.99 (m, 1H, H7), 6.97 – 6.93 (m, 2H, H6, H8), 6.48 (bs, 1H, NH$_2^a$), 5.98 – 5.90 (m, 1H, NH$_2^b$), 4.68 – 4.65 (m, 1H, H2), 4.29 (dd, ^2J = 13.6 Hz, ^3J = 3.2 Hz, 1H, H3^a), 3.78 (dd, ^2J = 13.6 Hz, ^3J = 7.2 Hz, 1H, H3^b), 1.54 (s, 9H, $(CH_3)_3$).

^{13}C-NMR (101 MHz, $CDCl_3$, 300 K): δ [ppm] = 170.7 ($CONH_2$), 152.4 (NCOO), 144.5 (C8a), 126.4 (C4a), 124.7 (C7), 123.8 (C5), 121.6 (C8), 116.9 (C6), 82.2 (*C*(CH_3)$_3$), 74.9 (C2), 43.7 (C3), 28.4 ((CH_3)$_3$).

HRMS (ESI) *m/z* [M+Na]$^+$ calculated for $[C_{14}H_{18}N_2NaO_4]^+$: 301.1159; found: 301.1156.

IR (film) $\tilde{\nu}_{max}/cm^{-1}$ = 3458 (m, NH), 3146 (w, NH), 3048 (w, CH_{arom}), 2978 (w, CH_{arom}), 2933 (w, CH_{aliph}), 1694 (s, C=O), 1587 (m, C=C), 1495 (m, CH_{arom}), 1368 (m, CH_{aliph}), 1357 (m, CH_{aliph}), 1255 (s, C–O), 1160 (s, C–N), 1149 (s, C–N), 1120 (s, C–O), 1071 (s, C–O).

***cis*-3-Methyl-1,4-benzodioxane-2-carboxamide (*rac-cis*-1v) and *trans*-3-methyl-1,4-benzodioxane-2-carboxamide (*rac-trans*-1v)**

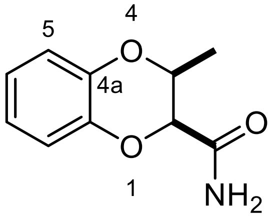

rac-cis-**1v**

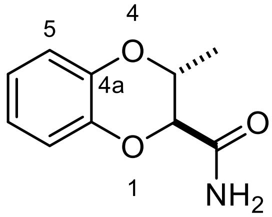

rac-trans-**1v**

$C_{10}H_{11}NO_3$
MW = 193.20 gmol^{-1}
d.r. = 70/30

According to GP E, HOBt · H_2O (114 mg, 742 µmol, 1.20 equiv.) was added to a solution of a mixture of *cis*-3-methyl-1,4-benzodioxane-2-carboxylic acid and *trans*-3-methyl-1,4-benzodioxane-2-carboxylic acid (*rac*-**S-3v**) (d.r. = 66/34) (120 mg, 618 µmol, 1.00 equiv.) in CH_2Cl_2 (6.0 mL) at 0 °C. After 10 min, EDCI · HCl (142 mg, 742 µmol, 1.20 equiv.) was added and after stirring the resulting mixture at 0 °C for another 30 min, aqueous ammonia (14 M, 631 mg, 2.65 mL, 37.1 mmol, 60.0 equiv.) was added before the reaction mixture was allowed to warm to r.t. and stirred vigorously for 14 h. Then, water was added, the layers were separated and the aqueous layer was extracted thrice with CH_2Cl_2 (3 × 10 mL). The combined organic layers were washed with brine and dried over Na_2SO_4. The solvents were removed under reduced pressure and the crude product was subjected to FCC (SiO_2, 50 → 100% EtOAc/hexanes) to yield a diastereomeric mixture of *cis*-3-methyl-1,4-benzodioxane-2-carboxamide (*rac-cis*-**1v**) and *trans*-3-methyl-1,4-benzodioxane-2-carboxamide (*rac-trans*-**1v**) as a white solid (85.5 mg, 443 µmol, 72%. d.r. = 70/30 in favor of *rac-cis*-**1v**). The two diastereoisomers were subsequently separated by chiral preparative HPLC (AD-H 250 × 20 mm, *n*-heptane/^{i}PrOH = 90/10). The diastereoisomers were assigned by comparison of the carboxylic acid starting material with the reported NMR data available in the literature.[100]

HRMS (ESI) *m/z* [M+H]$^+$: calculated for $[C_{10}H_{12}NO_3]^+$: 194.0812; found: 194.0812.

***cis*-3-Methyl-1,4-benzodioxane-2-carboxamide (*rac-cis*-1v)**

TLC (50% EtOAc/hexanes): $R_f = 0.30$ [UV, $KMnO_4$].

M.p.: 132°C

^{1}H-NMR (400 MHz, $CDCl_3$, 300 K): δ [ppm] = 6.95 – 6.85 (m, 4H, H5/H6/H7/H8), 6.63 (bs, 1H, NH_2^a), 5.98 (bs, 1H, NH_2^b), 4.84 (qd, 1H, $^3J = 6.6$ Hz, $^3J = 2.7$ Hz, 1H, H3), 4.61 (d, $^3J = 2.7$ Hz, 1H, H2), 1.28 (d, $^3J = 6.6$ Hz, 3H, CH_3).

^{13}C-NMR (101 MHz, $CDCl_3$, 300 K): δ [ppm] = 170.0 ($CONH_2$), 141.7 (C4a), 141.2 (C8a), 123.0 (C6/C7), 121.5 (C6/C7), 118.2 (C5/C8), 117.1 (C5/C8), 75.2 (C2), 69.9 (C3), 13.8 (CH_3).

IR (film) $\tilde{\nu}_{max}/cm^{-1}$ = 3394 (m, NH), 3176 (m, NH), 2976 (w, CH_{aliph}), 1655 (s, C=O), 1593 (m, C=C), 1465 (m, CH_{arom}), 1251 (s, C–O), 1135 (m, C–N), 1105 (m, C–O), 1079 (m, C–O).

***trans*-3-Methyl-1,4-benzodioxane-2-carboxamide (*rac-trans*-1v)**

TLC (50% EtOAc/hexanes): $R_f = 0.30$ [UV, $KMnO_4$].

M.p.: 145°C

^{1}H-NMR (400 MHz, $CDCl_3$, 300 K): δ [ppm] = 6.96 – 6.86 (m, 4H, H5/H6/H7/H8), 6.46 (bs, 1H, NH_2^a), 6.03 (bs, 1H, NH_2^b), 4.39 – 4.30 (m, 2H, H2, H3), 1.51 (d, $^3J = 6.3$ Hz, 3H, CH_3).

^{13}C-NMR (101 MHz, $CDCl_3$, 300 K): δ [ppm] = 170.5 ($CONH_2$), 142.7 (C4a), 142.3 (C8a), 122.7 (C6/C7), 121.9 (C6/C7), 117.8 (C5/C8), 116.8 (C5/C8), 77.8 (C2), 70.7 (C3), 17.2 (CH_3).

IR (film) $\tilde{\nu}_{max}/cm^{-1}$ = 3412 (m, NH), 3179 (w, NH), 2980 (w, CH_{aliph}), 2930 (w, CH_{aliph}), 1664 (s, C=O), 1608 (m, C=C), 1495 (s, CH_{arom}), 1262 (s, C–O), 1139 (m, C–N), 1057 (m, C–O).

1,2,3,4-Tetrahydronaphthalene-2-carboxamide (*rac*-**12**)

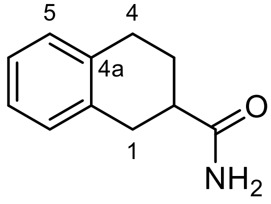

rac-**12**
$C_{11}H_{13}NO$
MW = 175.23 $gmol^{-1}$

According to GP E, HOBt · H_2O (209 mg, 1.36 mmol, 1.20 equiv.) was added to a solution of 1,2,3,4-tetrahydronaphthalene-2-carboxylic acid (200 mg, 1.14 mmol, 1.00 equiv.) in CH_2Cl_2 (10 mL) at 0 °C. After 10 min, EDCI (261 mg, 1.36 mmol, 1.20 equiv.) was added and after stirring the resulting mixture at 0 °C for another 30 min, aqueous ammonia (14 M, 2.39 g, 4.86 mL, 68.1 mmol, 60.0 equiv.) was added before the reaction mixture was allowed to warm to r.t. and stirred vigorously for 16 h. Then, water was added, the layers were separated and the aqueous layer was extracted thrice with CH_2Cl_2 (3 × 15 mL). The combined organic layers were washed with brine and dried over Na_2SO_4. The solvents were removed under reduced pressure and the crude product was subjected to FCC (SiO_2, 50 → 100% EtOAc/hexanes) to yield 1,2,3,4-tetrahydronaphthalene-2-carboxamide (*rac*-**12**) as a white solid (147 mg, 840 µmol, 74%).

TLC (50% EtOAc/pentane): R_f = 0.2 [UV, $KMnO_4$].

M.p.: 139 °C

^{1}H-NMR (400 MHz, $CDCl_3$, 300 K): δ [ppm] = 7.15 – 7.08 (m, 4H, H5, H6, H7, H8), 5.90 (bs, 2H, NH_2), 3.06 – 2.80 (m, 4H, H1, H4), 2.63 (dddd, ^2J = 11.2 Hz, ^3J = 9.7 Hz, ^3J = 6.2 Hz, ^3J = 3.1 Hz, 1H, H2), 2.16 (dddd, ^2J = 12.5 Hz, ^3J = 6.8 Hz, ^3J = 3.5 Hz, ^3J = 1.3 Hz, 1H, $H3^a$), 1.90 (*virt.* dtd, ^2J = 13.0 Hz, $^3J \approx {}^3J$ = 10.9 Hz, ^3J = 6.1 Hz, 1H, $H3^b$).

^{13}C-NMR (101 MHz, $CDCl_3$, 300 K): δ [ppm] = 178.4 ($CONH_2$), 135.7 (C4a), 134.8 (C8a), 129.2 (C5/C8), 129.0 (C5/C8), 126.2 (C6/C7), 126.1 (C6/C7), 41.3 (C2), 32.4 (C1), 28.6 (C4), 26.7 (C3).

HRMS (ESI) *m/z* $[M+H]^+$ calculated for $[C_{11}H_{14}NO]^+$: 176.1070; found: 176.1070.

IR (film) $\tilde{\nu}_{max}/cm^{-1}$ = 3343 (m, NH), 3173 (m, NH), 3014 (w, CH_{arom}), 2962 (w, CH_{aliph}), 2933 (w, CH_{aliph}), 2880 (w, CH_{aliph}), 2835 (w, CH_{aliph}), 1663 (s, C=O), 1623 (s, C=C), 1495 (m, CH_{arom}), 1433 (m, CH_{aliph}), 1147 (w, C–N).

Thiochromane-2-carboxamide (*rac*-**13**)

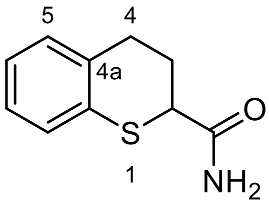

rac-**13**
$C_{10}H_{11}NOS$
MW = 193.26 gmol^{-1}

According to GP E, HOBt · H_2O (88.5 mg, 575 µmol, 1.20 equiv.) was added to a solution of 4-oxothiochromane-2-carboxylic acid (*rac*-**S-5**) (100 mg, 480 µmol, 1.00 equiv.) in CH_2Cl_2 (5 mL) at 0 °C. After 10 min, EDCI (111 mg, 575 µmol, 1.20 equiv.) was added and after stirring the resulting mixture at 0 °C for another 30 min, aqueous ammonia (14 M, 1.01 g, 2.06 mL, 28.8 mmol, 60.0 equiv.) was added before the reaction mixture was allowed to warm to r.t. and stirred vigorously for 16 h. Then, water was added, the layers were separated and the aqueous layer was extracted thrice with CH_2Cl_2 (3 × 10 mL). The combined organic layers were washed with brine and dried over Na_2SO_4. The solvents were removed under reduced pressure to obtain the crude 4-oxothiochromane-2-carboxamide.

Et_3SiH (1.68 g, 2.31 mL, 14.48 mmol, 30.0 equiv.) was added to a solution of the crude 4-oxothiochromane-2-carboxamide in CH_2Cl_2 (4.8 mL) at 0 °C. To this ice-cold solution, boron trifluoride diethyl etherate (1.03 g, 2.28 mL, 7.24 mmol, 15.0 equiv.) was added over 10 min. The resulting solution was then allowed to stir at r.t. for 1 h. The reaction mixture was quenched by the dropwise addition of sat. aq. $NaHCO_3$. Then, CH_2Cl_2 (10 mL) was added, the layers were separated and the aqueous layer was extracted thrice with CH_2Cl_2 (3 × 5 mL). The combined organic layers were washed with brine and dried over Na_2SO_4. The solvents were removed under reduced pressure and the crude product was subjected to FCC (SiO_2, 50 → 100% EtOAc/hexanes) to yield thiochromane-2-carboxamide (*rac*-**13**) as a white solid (55.0 mg, 285 µmol, 59%).

TLC (50% EtOAc/hexanes): $R_f = 0.40$ [UV, $KMnO_4$].

M.p.: 130 °C

^{1}H-NMR (400 MHz, $CDCl_3$, 300 K): δ [ppm] = 7.15 – 7.02 (m, 4H, H5, H6, H7, H8), 6.82 (bs, 1H, NH_2^a), 5.80 (bs, 1H, NH_2^b), 3.90 (dd, $^3J = 6.0$ Hz, $^3J = 4.6$ Hz, 1H, H2), 2.86 – 2.74 (m, 2H, H4), 2.54 (*virt.* dtd, $^2J = 13.5$ Hz, $^3J \approx {}^3J = 5.9$ Hz, $^3J = 4.8$ Hz, 1H, H3^a), 2.20 (dddd, $^2J = 13.5$ Hz, $^3J = 8.8$ Hz, $^3J = 5.8$ Hz, $^3J = 4.6$ Hz, 1H, H3^b).

^{13}C-NMR (101 MHz, $CDCl_3$, 300 K): δ [ppm] = 173.6 ($CONH_2$), 134.2 (C4a), 130.3 (C5/C6/C7/C8), 129.9 (C8a), 127.0 (C5/C6/C7/C8), 126.6 (C5/C6/C7/C8), 125.2 (C5/C6/C7/C8), 44.4 (C2), 27.0 (C4), 25.8 (C3).

HRMS (ESI) *m/z* $[M+H]^+$ calculated for $[C_{11}H_{12}NOS]^+$: 194.0634; found: 194.0631.

IR (film) $\tilde{\nu}_{max}/cm^{-1}$ = 3356 (m, NH), 3161 (m, NH), 3070 (w, CH_{arom}), 3056 (w, CH_{arom}), 3010 (w, CH_{arom}), 2957 (w, CH_{aliph}), 2940 (w, CH_{aliph}), 2918 (w, CH_{aliph}), 1655 (s, C=O), 1567 (m, C=C), 1476 (m, CH_{arom}), 1436 (m, CH_{aliph}), 1408 (m, CH_{aliph}), 1244 (m, C–O), 1121 (m, C–N), 1049 (m, C–O).

S10. Photochemical Deracemization Reactions

(*R*)-Chromane-2-carboxamide (1a)

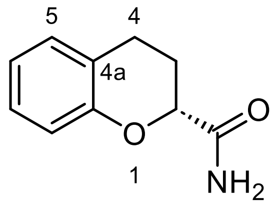

1a

$C_{10}H_{11}NO_2$

MW = 177.20 gmol^{-1}

According to GP F, a dried phototube (Ø = 1 cm) was charged with chromane-2-carboxamide (*rac*-**1a**) (4.43 mg, 25.0 µmol, 1.00 equiv.) and enantiomerically pure (+)-benzophenone **2** (1.01 mg, 2.50 µmol, 10 mol%) under an argon atmosphere. In a separate vial, a stock solution of PhSH was prepared by dissolving PhSH (5.00 µL) in dry $PhCF_3$ (1.00 mL). From this stock solution, 103 µL (551 µg, 5.00 µmol, 20 mol%) was added followed by the addition of dry $PhCF_3$ (10 mL). The resulting solution was degassed by being sparged with argon under ultrasonication for 15 min and irradiated at λ = 366 nm at 30 °C for 18 h. After irradiation, the volatile compounds were removed under reduced pressure and the crude reaction mixture was subjected to FCC (SiO_2, 50 → 80% EtOAc/hexanes) to yield (*R*)-chromane-2-carboxamide (**1a**) as a white solid (4.0 mg, 22.6 µmol, 90%, 94% *ee*).

TLC (80% EtOAc/pentane): R_f = 0.55 [UV, $KMnO_4$].

Specific Rotation: $[\alpha]_D^{27}$: +50 (*c* = 1.0, $CHCl_3$) [94% *ee*].

Chiral HPLC: 94% *ee* (AD-H 250 × 4.6 mm, *n*-heptane/^{i}PrOH = 90/10, 1 ml/min, λ = 210 nm); t_R = 9.39 min (major, **1a**), 11.07 min (minor, *ent*-**1a**).

0.500 mmol Scale:

According to GP F, a dried phototube was charged with chromane-2-carboxamide (*rac*-**1a**) (88.6 mg, 500 µmol, 1.00 equiv.), enantiomerically pure (+)-benzophenone **2** (20.1 mg, 50.0 µmol, 10 mol%) and PhSH (11.0 mg, 10.3 µL 100 µmol, 20 mol%) under an argon atmosphere followed by the addition of dry $PhCF_3$ (200 mL). The resulting solution was degassed by being sparged with argon under ultrasonication for 30 min and irradiated at λ = 366 nm at 30 °C for 20 h. After irradiation, the volatile compounds were removed under reduced pressure and the crude reaction mixture was subjected to FCC (SiO_2, 50 → 80% EtOAc/hexanes) to yield (*R*)-chromane-2-carboxamide (**1a**) as a white solid (77.0 mg, 435 µmol, 87%, 94% *ee*).

Chiral HPLC: 94% *ee* (AD-H 250 × 4.6 mm, *n*-heptane/^{i}PrOH = 90/10, 1 ml/min, λ = 210 nm); t_R = 9.39 min (major, **1a**), 11.11 min (minor, *ent*-**1a**).

(*R*)-6-Fluorochromane-2-carboxamide (1b)

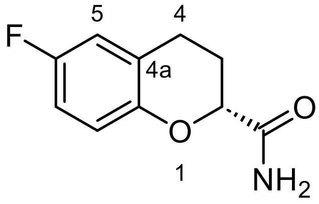

1b

$C_{10}H_{10}FNO_2$

MW = 195.19 $gmol^{-1}$

According to GP F, a dried phototube (Ø = 1 cm) was charged with 6-fluorochromane-2-carboxamide (*rac*-**1b**) (4.88 mg, 25.0 µmol, 1.00 equiv.) and enantiomerically pure (+)-benzophenone **2** (1.01 mg, 2.50 µmol, 10 mol%) under an argon atmosphere. In a separate vial, a stock solution of PhSH was prepared by dissolving PhSH (5.00 µL) in dry $PhCF_3$ (1.00 mL). From this stock solution, 103 µL (551 µg, 5.00 µmol, 20 mol%) was added followed by the addition of dry $PhCF_3$ (10 mL). The resulting solution was degassed by being sparged with argon under ultrasonication for 15 min and irradiated at λ = 366 nm at 30 °C for 18 h. After irradiation, the volatile compounds were removed under reduced pressure and the crude reaction mixture was subjected to FCC (SiO_2, 50 → 90% EtOAc/hexanes) to yield (*R*)-6-fluorochromane-2-carboxamide (**1b**) as a white solid (4.1 mg, 21.0 µmol, 84%, 98 % *ee*).

TLC (60% EtOAc/pentane): R_f = 0.27 [UV, $KMnO_4$].

Specific Rotation: $[\alpha]_D^{27}$: +70 (*c* = 0.5, $CHCl_3$) [98% *ee*].

Chiral HPLC: 99% *ee* (IA 250 × 4.6 mm, *n*-heptane/^{i}PrOH = 90/10, 1 ml/min, λ = 210 nm); t_R = 9.71 min (major, **1b**), 11.00 min (minor, *ent*-**1b**).

0.500 mmol Scale:

According to a modified GP F, a dried phototube was charged with 6-fluorochromane-2-carboxamide (*rac*-**1b**) (97.6 mg, 500 µmol, 1.00 equiv.), enantiomerically pure (+)-benzophenone **2** (20.1 mg, 50.0 µmol, 10 mol%) and PhSH (11.0 mg, 10.3 µL 100 µmol, 20 mol%) under an argon atmosphere followed by the addition of dry $PhCF_3$ (200 mL). The resulting solution was degassed by being sparged with argon under ultrasonication for 30 min and irradiated at λ = 366 nm at 30 °C for 24 h. After irradiation, the volatile compounds were removed under reduced pressure and the crude reaction mixture was subjected to FCC (SiO_2, 50 → 90% EtOAc/hexanes) followed by the precipitation from CH_2Cl_2 to yield (*R*)-6-fluorochromane-2-carboxamide (**1b**) as a white solid (81.0 mg, 415 µmol, 83%, 99% *ee*).

Chiral HPLC: 99% *ee* (IA 250 × 4.6 mm, *n*-heptane/^{i}PrOH = 90/10, 1 ml/min, λ = 210 nm); t_R = 10.04 min (major, **1b**), 11.91 min (minor, *ent*-**1b**).

Specific Rotation: $[\alpha]_D^{27}$: +72 (*c* = 0.5, $CHCl_3$) [99% *ee*].

(*S*)-6-Fluorochromane-2-carboxamide (*ent*-**1b**) **on a 0.5 mmol scale**

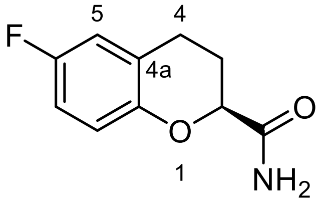

ent-**1b**
$C_{10}H_{10}FNO_2$
MW = 195.19 gmol^{-1}

According to GP F, a dried phototube (Ø = 1 cm) was charged with Following GP F, a dried phototube was charged with 6-fluorochromane-2-carboxamide (*rac*-**1b**) (97.6 mg, 500 µmol, 1.00 equiv.), enantiomerically pure (-)-benzophenone **2** (20.1 mg, 50.0 µmol, 10 mol%) and PhSH (11.0 mg, 10.3 µL 100 µmol, 20 mol%) under an argon atmosphere followed by the addition of dry $PhCF_3$ (200 mL). The resulting solution was degassed by being sparged with argon under ultrasonication for 30 min and irradiated at λ = 366 nm at 30 °C for 24 h. After irradiation, the volatile compounds were removed under reduced pressure and the crude reaction mixture was subjected to FCC (SiO_2, 50 → 90% EtOAc/hexanes) followed by the precipitation from CH_2Cl_2 to yield (*S*)-6-fluorochromane-2-carboxamide (**1b**) as a white solid (79.0 mg, 405 µmol, 81%, 99% *ee*).

Chiral HPLC: 99% *ee* (IA 250 × 4.6 mm, *n*-heptane/^{i}PrOH = 90/10, 1 ml/min, λ = 210 nm); t_R = 10.11 min (minor, **1b**), 11.21 min (major, *ent*-**1b**).

Specific Rotation: $[\alpha]_D^{27}$: −72 (*c* = 0.5, $CHCl_3$) [99% *ee*].

(*R*)-6-Chlorochromane-2-carboxamide (1c)

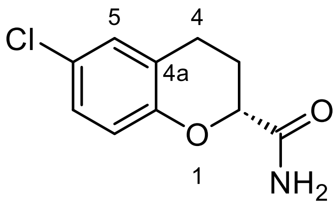

1c
$C_{10}H_{10}ClNO_2$
MW = 211.65 gmol^{-1}

According to GP F, a dried phototube (Ø = 1 cm) was charged with 6-chlorochromane-2-carboxamide (*rac*-**1c**) (5.29 mg, 25.0 µmol, 1.00 equiv.) and enantiomerically pure (+)-benzophenone **2** (1.01 mg, 2.50 µmol, 10 mol%) under an argon atmosphere. In a separate vial, a stock solution of PhSH was prepared by dissolving PhSH (5.00 µL) in dry $PhCF_3$ (1.00 mL). From this stock solution, 103 µL (551 µg, 5.00 µmol, 20 mol%) was added followed by the addition of dry

PhCF$_3$ (10 mL). The resulting solution was degassed by being sparged with argon under ultrasonication for 15 min and irradiated at λ = 366 nm at 30 °C for 18 h. After irradiation, the volatile compounds were removed under reduced pressure and the crude reaction mixture was subjected to FCC (SiO$_2$, 50 → 100% EtOAc/hexanes) to yield (*R*)-6-chlorochromane-2-carboxamide (**1c**) as a white solid (4.36 mg, 20.6 μmol, 82%, 99% *ee*).

TLC (80% EtOAc/pentane): R_f = 0.46 [UV, KMnO$_4$].

Specific Rotation: $[\alpha]_D^{27}$: −60 (*c* = 0.67, CHCl$_3$) [99% *ee*].

Chiral HPLC: 99% *ee* (AD-H 250 × 4.6 mm, *n*-heptane/^{i}PrOH = 90/10, 1 ml/min, λ = 210 nm); t_R = 10.90 min (minor, *ent*-**1c**), 13.15 min (major, **1c**).

(*R*)-6-Bromochromane-2-carboxamide (1d)

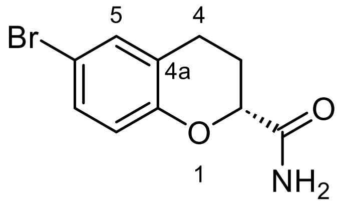

1d

$C_{10}H_{10}BrNO_2$

MW = 256.10 gmol^{-1}

According to GP F, a dried phototube (Ø = 1 cm) was charged with 6-bromochromane-2-carboxamide (*rac*-**1d**) (6.40 mg, 25.0 μmol, 1.00 equiv.) and enantiomerically pure (+)-benzophenone **2** (1.01 mg, 2.50 μmol, 10 mol%) under an argon atmosphere. In a separate vial, a stock solution of PhSH was prepared by dissolving PhSH (5.00 μL) in dry PhCF$_3$ (1.00 mL). From this stock solution, 103 μL (551 μg, 5.00 μmol, 20 mol%) was added followed by the addition of dry PhCF$_3$ (10 mL). The resulting solution was degassed by being sparged with argon under ultrasonication for 15 min and irradiated at λ = 366 nm at 30 °C for 18 h. After irradiation, the volatile compounds were removed under reduced pressure and the crude reaction mixture was subjected to FCC (SiO$_2$, 50 → 100% EtOAc/hexanes) to yield (*R*)-6-bromochromane-2-carboxamide (**1d**) as a white solid (5.2 mg, 20.3 μmol, 81%, 96% *ee*).

TLC (50% EtOAc/hexanes): R_f = 0.40 [UV, KMnO$_4$].

Specific Rotation: $[\alpha]_D^{27}$: +40 (*c* = 0.5, CHCl$_3$) [96% *ee*].

Chiral HPLC: 96% *ee* (AD-H 250 × 4.6 mm, *n*-heptane/^{i}PrOH = 90/10, 1 ml/min, λ = 210 nm); t_R = 11.39 min (major, **1d**), 13.89 min (minor, *ent*-**1d**).

(*R*)-6-Iodochromane-2-carboxamide (1e)

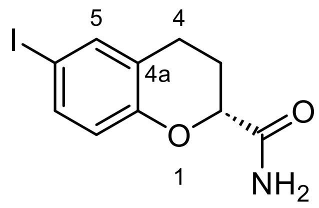

1e
$C_{10}H_{10}INO_2$
MW = 303.10 $gmol^{-1}$

According to GP F, a dried phototube (Ø = 1 cm) was charged with 6-iodochromane-2-carboxamide (*rac*-**1e**) (7.58 mg, 25.0 µmol, 1.00 equiv.) and enantiomerically pure (+)-benzophenone **2** (1.01 mg, 2.50 µmol, 10 mol%) under an argon atmosphere. In a separate vial, a stock solution of PhSH was prepared by dissolving PhSH (5.00 µL) in dry $PhCF_3$ (1.00 mL). From this stock solution, 103 µL (551 µg, 5.00 µmol, 20 mol%) was added followed by the addition of dry $PhCF_3$ (10 mL). The resulting solution was degassed by being sparged with argon under ultrasonication for 15 min and irradiated at λ = 366 nm at 30 °C for 18 h. After irradiation, the volatile compounds were removed under reduced pressure and the crude reaction mixture was subjected to FCC (SiO_2, 50 → 90% EtOAc/hexanes) to yield (*R*)-6-iodochromane-2-carboxamide (**1e**) as a white solid (6.07 mg, 20.0 µmol, 80%, 94% *ee*).

TLC (50% EtOAc/hexanes): R_f = 0.41 [UV, $KMnO_4$].

Specific Rotation: $[\alpha]_D^{25}$: +40 (*c* = 0.5, $CHCl_3$) [94% *ee*].

Chiral HPLC: 94% *ee* (AD-H 250 × 4.6 mm, *n*-heptane/^{i}PrOH = 80/20, 1 ml/min, λ = 210 nm); t_R = 7.52 min (major, **1e**), 8.83 min (minor, *ent*-**1e**).

(*R*)-6-Methylchromane-2-carboxamide (1f)

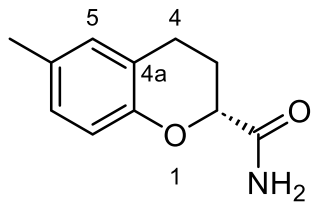

1f
$C_{11}H_{13}NO_2$
MW = 191.23 $gmol^{-1}$

According to GP F, a dried phototube (Ø = 1 cm) was charged with 6-methylchromane-2-carboxamide (*rac*-**1f**) (4.78 mg, 25.0 µmol, 1.00 equiv.) and enantiomerically pure (+)-benzophenone **2** (1.01 mg, 2.50 µmol, 10 mol%) under an argon atmosphere. In a separate vial, a stock solution of PhSH was prepared by dissolving PhSH (5.00 µL) in dry $PhCF_3$ (1.00 mL). From this stock solution, 103 µL (551 µg, 5.00 µmol, 20 mol%) was added followed by the addition of dry $PhCF_3$ (10 mL). The resulting solution was degassed by being sparged with argon under ultrasonication for 15 min and irradiated at λ = 366 nm at 30 °C for 18 h. After irradiation, the volatile compounds were removed under reduced pressure and the crude reaction mixture was subjected to FCC (SiO_2,

50 → 90% EtOAc/hexanes) to yield (*R*)-6-methylchromane-2-carboxamide (**1f**) as a white solid (3.9 mg, 20.4 μmol, 82%, 95% *ee*).

TLC (50% EtOAc/hexanes): R_f = 0.40 [UV, $KMnO_4$].

Specific Rotation: $[\alpha]_D^{27}$: +32 (*c* = 1.0, $CHCl_3$) [95% *ee*].

Chiral HPLC: 95% *ee* (AD-H 250 × 4.6 mm, *n*-heptane/^{i}PrOH = 90/10, 1 ml/min, λ = 210 nm); t_R = 8.97 min (major, **1f**), 11.46 min (minor, *ent*-**1f**).

(*R*)-6-Methoxychromane-2-carboxamide (1g)

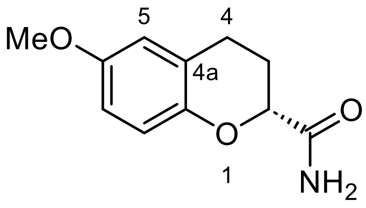

1g
$C_{11}H_{13}NO_3$
MW = 207.23 gmol^{-1}

According to GP F, a dried phototube (Ø = 1 cm) was charged with 6-methoxychromane-2-carboxamide (*rac*-**1g**) (5.18 mg, 25.0 μmol, 1.00 equiv.) and enantiomerically pure (+)-benzophenone **2** (1.01 mg, 2.50 μmol, 10 mol%) under an argon atmosphere. In a separate vial, a stock solution of PhSH was prepared by dissolving PhSH (5.00 μL) in dry $PhCF_3$ (1.00 mL). From this stock solution, 103 μL (551 μg, 5.00 μmol, 20 mol%) was added followed by the addition of dry $PhCF_3$ (10 mL). The resulting solution was degassed by being sparged with argon under ultrasonication for 15 min and irradiated at λ = 366 nm at 30 °C for 18 h. After irradiation, the volatile compounds were removed under reduced pressure and the crude reaction mixture was subjected to FCC (SiO_2, 50 → 100% EtOAc/hexanes) to yield (*R*)-6-methoxychromane-2-carboxamide (**1g**) as a white solid (4.3 mg, 20.8 μmol, 83%, 80% *ee*).

TLC (50% EtOAc/hexanes): R_f = 0.26 [UV, $KMnO_4$].

Specific Rotation: $[\alpha]_D^{27}$: +28 (*c* = 0.5, $CHCl_3$) [80% *ee*].

Chiral HPLC: 80% *ee* (IC 250 × 4.6 mm, *n*-heptane/^{i}PrOH = 70/30, 1 ml/min, λ = 210 nm); t_R = 12.49 min (minor, *ent*-**1g**), 21.52 min (major, **1g**).

(*R*)-6-Phenylchromane-2-carboxamide (1h)

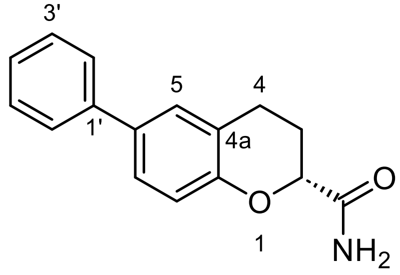

1h
$C_{16}H_{15}NO_2$
MW = 253.30 gmol^{-1}

According to GP F, a dried phototube (Ø = 1 cm) was charged with 6-phenylchromane-2-carboxamide (*rac*-**1h**) (6.33 mg, 25.0 µmol, 1.00 equiv.) and enantiomerically pure (+)-benzophenone **2** (1.01 mg, 2.50 µmol, 10 mol%) under an argon atmosphere. In a separate vial, a stock solution of PhSH was prepared by dissolving PhSH (5.00 µL) in dry $PhCF_3$ (1.00 mL). From this stock solution, 103 µL (551 µg, 5.00 µmol, 20 mol%) was added followed by the addition of dry $PhCF_3$ (10 mL). The resulting solution was degassed by being sparged with argon under ultrasonication for 15 min and irradiated at λ = 366 nm at 30 °C for 18 h. After irradiation, the volatile compounds were removed under reduced pressure and the crude reaction mixture was subjected to FCC (SiO_2, 50 → 100% EtOAc/hexanes) to yield (*R*)-6-phenylchromane-2-carboxamide (**1h**) as a white solid (5.64 mg, 22.3 µmol, 89%, 99% *ee*).

TLC (50% EtOAc/hexanes): R_f = 0.55 [UV, $KMnO_4$].

Specific Rotation: $[\alpha]_D^{27}$: +32 (*c* = 0.5, $CHCl_3$) [99% *ee*].

Chiral HPLC: 99% *ee* (IC 250 × 4.6 mm, *n*-heptane/^{i}PrOH = 90/10, 1 ml/min, λ = 210 nm); t_R = 11.54 min (major, **1h**), 13.36 min (minor, *ent*-**1h**).

(*R*)-6-Ethylchromane-2-carboxamide (1i)

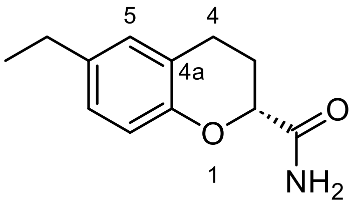

1i
$C_{12}H_{15}NO_2$
MW = 205.26 gmol^{-1}

According to GP F, a dried phototube (Ø = 1 cm) was charged with 6-ethylchromane-2-carboxamide (*rac*-**1i**) (5.13 mg, 25.0 µmol, 1.00 equiv.) and enantiomerically pure (+)-benzophenone **2** (1.01 mg, 2.50 µmol, 10 mol%) under an argon atmosphere. In a separate vial, a stock solution of PhSH was prepared by dissolving PhSH (5.00 µL) in dry $PhCF_3$ (1.00 mL). From this stock solution, 103 µL (551 µg, 5.00 µmol, 20 mol%) was added followed by the addition of dry $PhCF_3$ (10 mL). The resulting solution was degassed by being sparged with argon under ultrasonication for 15 min and irradiated at λ = 366 nm at 30 °C for 18 h. After irradiation, the volatile compounds were removed under reduced pressure and the crude reaction mixture was

subjected to FCC (SiO_2, 50 → 90% EtOAc/hexanes) to yield (*R*)-6-ethylchromane-2-carboxamide (**1i**) as a white solid (4.53 mg, 22.1 µmol, 88%, 93% *ee*).

TLC (50% EtOAc/hexanes): R_f = 0.23 [UV, $KMnO_4$].

Specific Rotation: $[\alpha]_D^{27}$: +36 (c = 0.5, $CHCl_3$) [93% *ee*].

Chiral HPLC: 93% *ee* (AD-H 250 × 4.6 mm, *n*-heptane/^{i}PrOH = 90/10, 1 ml/min, λ = 210 nm); t_R = 8.51 min (major, **1i**), 11.38 min (minor, *ent*-**1i**).

(*R*)-5-Methylchromane-2-carboxamide (1j)

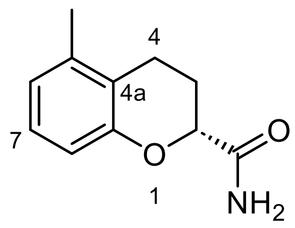

1j
$C_{11}H_{13}NO_2$
MW = 191.23 gmol^{-1}

According to GP F, a dried phototube (Ø = 1 cm) was charged with 5-methylchromane-2-carboxamide (*rac*-**1j**) (4.78 mg, 25.0 µmol, 1.00 equiv.) and enantiomerically pure (+)-benzophenone **2** (1.01 mg, 2.50 µmol, 10 mol%) under an argon atmosphere. In a separate vial, a stock solution of PhSH was prepared by dissolving PhSH (5.00 µL) in dry $PhCF_3$ (1.00 mL). From this stock solution, 103 µL (551 µg, 5.00 µmol, 20 mol%) was added followed by the addition of dry $PhCF_3$ (10 mL). The resulting solution was degassed by being sparged with argon under ultrasonication for 15 min and irradiated at λ = 366 nm at 30 °C for 18 h. After irradiation, the volatile compounds were removed under reduced pressure and the crude reaction mixture was subjected to FCC (SiO_2, 50 → 90% EtOAc/hexanes) to yield (*R*)-5-methylchromane-2-carboxamide (**1j**) as a white solid (4.1 mg, 21.4 µmol, 86%, 94% *ee*).

TLC (60% EtOAc/hexanes): R_f = 0.18 [UV, $KMnO_4$].

Specific Rotation: $[\alpha]_D^{26}$: +48 (c = 0.5, $CHCl_3$) [94% *ee*].

Chiral HPLC: 94% *ee* (AS-H 250 × 4.6 mm, *n*-heptane/^{i}PrOH = 50/50, 1 ml/min, λ = 210 nm); t_R = 9.51 min (major, **1j**), 18.63 min (minor, *ent*-**1j**).

(*R*)-7-Methylchromane-2-carboxamide (1k)

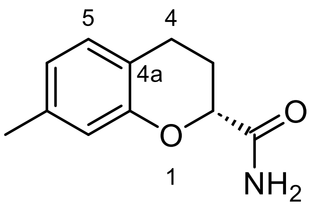

1k
$C_{11}H_{13}NO_2$
MW = 191.23 gmol^{-1}

According to GP F, a dried phototube (Ø = 1 cm) was charged with 7-methylchromane-2-carboxamide (*rac*-**1k**) (4.78 mg, 25.0 µmol, 1.00 equiv.) and enantiomerically pure (+)-benzophenone **2** (1.01 mg, 2.50 µmol, 10 mol%) under an argon atmosphere. In a separate vial, a stock solution of PhSH was prepared by dissolving PhSH (5.00 µL) in dry $PhCF_3$ (1.00 mL). From this stock solution, 103 µL (551 µg, 5.00 µmol, 20 mol%) was added followed by the addition of dry $PhCF_3$ (10 mL). The resulting solution was degassed by being sparged with argon under ultrasonication for 15 min and irradiated at λ = 366 nm at 30 °C for 18 h. After irradiation, the volatile compounds were removed under reduced pressure and the crude reaction mixture was subjected to FCC (SiO_2, 50 → 90% EtOAc/hexanes) to yield (*R*)-7-methylchromane-2-carboxamide (**1k**) as a white solid (4.1 mg, 21.4 µmol, 86%, 91% *ee*).

TLC (80% EtOAc/hexanes): R_f = 0.49 [UV, $KMnO_4$].

Specific Rotation: $[\alpha]_D^{26}$: +32 (*c* = 0.5, $CHCl_3$) [91% *ee*].

Chiral HPLC: 91% *ee* (AD-H 250 × 4.6 mm, *n*-heptane/^{i}PrOH = 90/10, 1 ml/min, λ = 210 nm); t_R = 7.77 min (major, **1k**), 9.57 min (minor, *ent*-**1k**).

(*R*)-8-Methylchromane-2-carboxamide (1l)

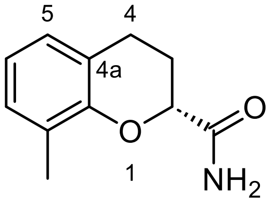

1l
$C_{11}H_{13}NO_2$
MW = 191.23 gmol^{-1}

According to GP F, a dried phototube (Ø = 1 cm) was charged with 8-methylchromane-2-carboxamide (*rac*-**1l**) (4.78 mg, 25.0 µmol, 1.00 equiv.) and enantiomerically pure (+)-benzophenone **2** (1.01 mg, 2.50 µmol, 10 mol%) under an argon atmosphere. In a separate vial, a stock solution of PhSH was prepared by dissolving PhSH (5.00 µL) in dry $PhCF_3$ (1.00 mL). From this stock solution, 103 µL (551 µg, 5.00 µmol, 20 mol%) was added followed by the addition of dry $PhCF_3$ (10 mL). The resulting solution was degassed by being sparged with argon under ultrasonication for 15 min and irradiated at λ = 366 nm at 30 °C for 18 h. After irradiation, the volatile compounds were removed under reduced pressure and the crude reaction mixture was subjected to FCC (SiO_2, 50 → 100%

EtOAc/hexanes) to (*R*)-8-methylchromane-2-carboxamide (**1l**) as a white solid (3.43 mg, 17.9 μmol, 72%, 97% *ee*).

TLC (60% EtOAc/hexanes): $R_f = 0.38$ [UV, $KMnO_4$].

Specific Rotation: $[\alpha]_D^{26}$: +52 ($c = 0.5$, $CHCl_3$) [97% *ee*].

Chiral HPLC: 97% *ee* (IC 250 × 4.6 mm, *n*-heptane/^{i}PrOH = 70/30, 1 ml/min, λ = 210 nm); t_R = 9.01 min (minor, *ent*-**1l**), 9.98 min (major, **1l**).

(*R*)-6,7,8,9-Tetrahydro-benzo[*g*]chromane-2-carboxamide (1m)

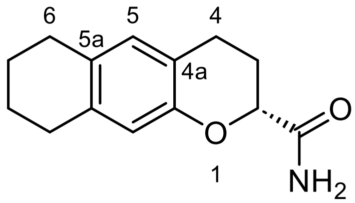

1m

$C_{14}H_{17}NO_2$

MW = 231.30 gmol^{-1}

According to GP F, a dried phototube (Ø = 1 cm) was charged with 6,7,8,9-tetrahydro-benzo[*g*]chromane-2-carboxamide (*rac*-**1m**) (5.78 mg, 25.0 μmol, 1.00 equiv.) and enantiomerically pure (+)-benzophenone **2** (1.01 mg, 2.50 μmol, 10 mol%) under an argon atmosphere. In a separate vial, a stock solution of PhSH was prepared by dissolving PhSH (5.00 μL) in dry $PhCF_3$ (1.00 mL). From this stock solution, 103 μL (551 μg, 5.00 μmol, 20 mol%) was added followed by the addition of dry $PhCF_3$ (10 mL). The resulting solution was degassed by being sparged with argon under ultrasonication for 15 min and irradiated at λ = 366 nm at 30 °C for 18 h. After irradiation, the volatile compounds were removed under reduced pressure and the crude reaction mixture was subjected to FCC (SiO_2, 50 → 100% EtOAc/hexanes) to yield (*R*)-6,7,8,9-tetrahydro-benzo[*g*]chromane-2-carboxamide (**1m**) as a white solid (4.27 mg, 18.5 μmol, 74%, 82% *ee*).

TLC (60% EtOAc/hexanes): $R_f = 0.34$ [UV, $KMnO_4$].

Specific Rotation: $[\alpha]_D^{27}$: +24 ($c = 0.5$, $CHCl_3$) [82% *ee*].

Chiral HPLC: 82% *ee* (AD-H 250 × 4.6 mm, *n*-heptane/^{i}PrOH = 90/10, 1 ml/min, λ = 210 nm); t_R = 8.12 min (major, **1m**), 9.93 min (minor, *ent*-**1m**).

(*R*)-6-(Pyridine-3-yl)chromane-2-carboxamide (1n)

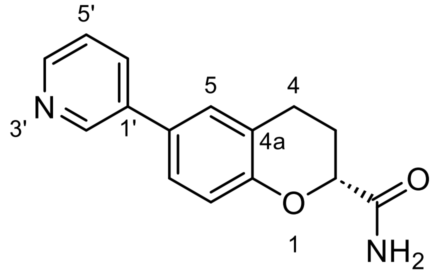

1n
$C_{15}H_{14}N_2O_2$
MW = 254.29 gmol^{-1}

According to GP F, a dried phototube (Ø = 1 cm) was charged with 6-(pyridine-3-yl)chromane-2-carboxamide (*rac*-**1n**) (6.36 mg, 25.0 μmol, 1.00 equiv.) and enantiomerically pure (+)-benzophenone **2** (1.01 mg, 2.50 μmol, 10 mol%) under an argon atmosphere. In a separate vial, a stock solution of PhSH was prepared by dissolving PhSH (5.00 μL) in dry $PhCF_3$ (1.00 mL). From this stock solution, 103 μL (551 μg, 5.00 μmol, 20 mol%) was added followed by the addition of dry $PhCF_3$ (10 mL). The resulting solution was degassed by being sparged with argon under ultrasonication for 15 min and irradiated at λ = 366 nm at 30 °C for 18 h. After irradiation, the volatile compounds were removed under reduced pressure and the crude reaction mixture was subjected to FCC (SiO_2, 60 → 100% EtOAc/hexanes) to yield (*R*)-6-(pyridine-3-yl)chromane-2-carboxamide (**1n**) as a white solid (5.53 mg, 21.8 μmol, 87%, 87% *ee*).

TLC (90% EtOAc/hexanes): R_f = 0.15 [UV, $KMnO_4$].

Specific Rotation: $[\alpha]_D^{25}$: +36 (*c* = 1.0, $CHCl_3$) [87% *ee*].

Chiral HPLC: 87% *ee* (AS-RH 150 × 4.6 mm, MeCN/water = 20/80 → 100/0 over 30 min, 1 ml/min, λ = 210 nm); t_R = 10.03 min (major, **1n**), 12.51 min (minor, *ent*-**1n**).

(*R*)-7-Methoxychromane-2-carboxamide (1o)

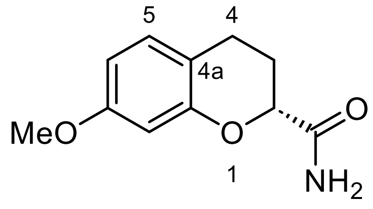

1o
$C_{11}H_{13}NO_3$
MW = 207.23 gmol^{-1}

According to GP F, a dried phototube (Ø = 1 cm) was charged with 7-methoxychromane-2-carboxamide (*rac*-**1o**) (5.18 mg, 25.0 μmol, 1.00 equiv.) and enantiomerically pure (+)-benzophenone **2** (1.01 mg, 2.50 μmol, 10 mol%) under an argon atmosphere. In a separate vial, a stock solution of PhSH was prepared by dissolving PhSH (5.00 μL) in dry $PhCF_3$ (1.00 mL). From this stock solution, 103 μL (551 μg, 5.00 μmol, 20 mol%) was added followed by the addition of dry $PhCF_3$ (10 mL). The resulting solution was degassed by being sparged with argon under ultrasonication for 15 min and irradiated at λ = 366 nm at 30 °C for 18 h. After irradiation, the volatile compounds were removed under reduced pressure and the

crude reaction mixture was subjected to FCC (SiO_2, 60 → 100% EtOAc/hexanes) to yield (*R*)-7-methoxychromane-2-carboxamide (**1o**) as a white solid (3.70 mg, 17.9 µmol, 71%, 80% *ee*).

TLC (50% EtOAc/hexanes): R_f = 0.22 [UV, $KMnO_4$].

Specific Rotation: $[\alpha]_D^{27}$: +32 (*c* = 1.0, $CHCl_3$) [80% *ee*].

Chiral HPLC: 80% *ee* (AD-H 250 × 4.6 mm, *n*-heptane/^{i}PrOH = 90/10, 1 ml/min, λ = 210 nm); t_R = 14.05 min (major, **1o**), 16.31 min (minor, *ent*-**1o**).

(*R*)-7-Fluorochromane-2-carboxamide (1p)

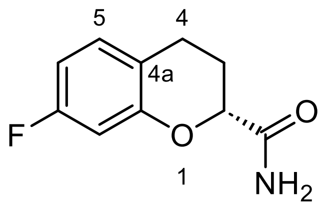

1p
$C_{10}H_{10}FNO_2$
MW = 195.19 gmol^{-1}

According to GP F, a dried phototube (Ø = 1 cm) was charged with 7-fluorochromane-2-carboxamide (*rac*-**1p**) (4.88 mg, 25.0 µmol, 1.00 equiv.) and enantiomerically pure (+)-benzophenone **2** (1.01 mg, 2.50 µmol, 10 mol%) under an argon atmosphere. In a separate vial, a stock solution of PhSH was prepared by dissolving PhSH (5.00 µL) in dry $PhCF_3$ (1.00 mL). From this stock solution, 103 µL (551 µg, 5.00 µmol, 20 mol%) was added followed by the addition of dry $PhCF_3$ (10 mL). The resulting solution was degassed by being sparged with argon under ultrasonication for 15 min and irradiated at λ = 366 nm at 30 °C for 18 h. After irradiation, the volatile compounds were removed under reduced pressure and the crude reaction mixture was subjected to FCC (SiO_2, 50 → 100% EtOAc/hexanes) to yield (*R*)-7-fluorochromane-2-carboxamide (**1p**) as a white solid (3.6 mg, 18.4 µmol, 74%, 90% *ee*).

TLC (60% EtOAc/hexanes): R_f = 0.27 [UV, $KMnO_4$].

Specific Rotation: $[\alpha]_D^{25}$: +38 (*c* = 1.0, $CHCl_3$) [90% *ee*].

Chiral HPLC: 90% *ee* (IA 250 × 4.6 mm, *n*-heptane/^{i}PrOH = 90/10, 1 ml/min, λ = 210 nm); t_R = 8.91 min (major, **1p**), 10.15 min (minor, *ent*-**1p**).

(*R*)-8-Fluorochromane-2-carboxamide (1q)

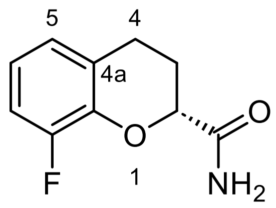

1q
$C_{10}H_{10}NO_2$
MW = 195.19 gmol^{-1}

According to GP F, a dried phototube (Ø = 1 cm) was charged with 8-fluorochromane-2-carboxamide (*rac*-**1q**) (4.88 mg, 25.0 µmol, 1.00 equiv.) and enantiomerically pure (+)-benzophenone **2** (1.01 mg, 2.50 µmol, 10 mol%) under an argon atmosphere. In a separate vial, a stock solution of PhSH was prepared by dissolving PhSH (5.00 µL) in dry $PhCF_3$ (1.00 mL). From this stock solution, 103 µL (551 µg, 5.00 µmol, 20 mol%) was added followed by the addition of dry $PhCF_3$ (10 mL). The resulting solution was degassed by being sparged with argon under ultrasonication for 15 min and irradiated at λ = 366 nm at 30 °C for 18 h. After irradiation, the volatile compounds were removed under reduced pressure and the crude reaction mixture was subjected to FCC (SiO_2, 50 → 100% EtOAc/hexanes) to yield (*R*)-8-fluorochromane-2-carboxamide (**1q**) as a white solid (4.1 mg, 21.0 µmol, 84%, 95% *ee*).

TLC (60% EtOAc/hexanes): R_f = 0.29 [UV, $KMnO_4$].

Specific Rotation: $[\alpha]_D^{26}$: +40 (*c* = 1.0, $CHCl_3$) [95% *ee*].

Chiral HPLC: 95% *ee* (AS-H 250 × 4.6 mm, *n*-heptane/^{i}PrOH = 50/50, 1 ml/min, λ = 210 nm); t_R = 17.86 min (major, **1q**), 27.31 min (minor, *ent*-**1q**).

(*R*)-1,4-Benzodioxane-2-carboxamide (1r)

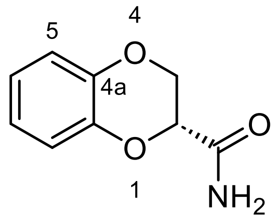

1r
$C_9H_9NO_3$
MW = 179.18 gmol^{-1}

According to GP F, a dried phototube (Ø = 1 cm) was charged with 2,3-dihydrobenzo[*b*][1,4]dioxine-2-carboxamide (*rac*-**1r**) (4.48 mg, 25.0 µmol, 1.00 equiv.) and enantiomerically pure (+)-benzophenone **2** (1.01 mg, 2.50 µmol, 10 mol%) under an argon atmosphere. In a separate vial, a stock solution of PhSH was prepared by dissolving PhSH (5.00 µL) in dry $PhCF_3$ (1.00 mL). From this stock solution, 206 µL (1.10 mg, 10.0 µmol, 40 mol%) was added followed by the addition of dry $PhCF_3$ (10 mL). The resulting solution was degassed by being sparged with argon under ultrasonication for 15 min and irradiated at λ = 366 nm at 30 °C for 18 h. After irradiation, the volatile compounds were removed under reduced pressure and the crude reaction mixture was subjected to FCC (SiO_2,

60 → 100% EtOAc/hexanes) to yield (*R*)-2,3-dihydrobenzo[*b*][1,4]dioxine-2-carboxamide (**1r**) as a white solid (3.7 mg, 20.7 µmol, 83%, 87% *ee*).

TLC (60% EtOAc/hexanes): R_f = 0.25 [UV, $KMnO_4$].

Chiral HPLC: 87% *ee* (IC 250 × 4.6 mm, *n*-heptane/^{i}PrOH = 70/30, 1 ml/min, λ = 210 nm); t_R = 7.56 min (minor, *ent*-**1r**), 8.90 min (major, **1r**).

0.500 mmol Scale:

According to a modified GP F, a dried phototube was charged with 2,3-dihydrobenzo[*b*][1,4]dioxine-2-carboxamide (*rac*-**1r**) (89.6 mg, 500 µmol, 1.00 equiv.), enantiomerically pure (+)-benzophenone **2** (20.1 mg, 50.0 µmol, 10 mol%) and PhSH (22.0 mg, 20.5 µL 100 µmol, 40 mol%) under an argon atmosphere followed by the addition of dry $PhCF_3$ (200 mL). The resulting solution was degassed by being sparged with argon under ultrasonication for 30 min and irradiated at λ = 366 nm at 30 °C for 20 h. After irradiation, the volatile compounds were removed under reduced pressure and the crude reaction mixture was subjected to FCC (SiO_2, 60 → 100% EtOAc/hexanes) to yield (*R*)-2,3-dihydrobenzo[*b*][1,4]dioxine-2-carboxamide (**1r**) as a white crystalline solid (75.5 mg, 421 µmol, 84%, 92% *ee*).

Specific Rotation: $[\alpha]_D^{27}$: +110 (*c* = 1.0, $CHCl_3$) [92% *ee*].

Chiral HPLC: 92% *ee* (IC 250 × 4.6 mm, *n*-heptane/^{i}PrOH = 70/30, 1 ml/min, λ = 210 nm); t_R = 7.56 min (minor, *ent*-**1r**), 8.90 min (major, **1r**).

(*R*)-3,3-Dimethyl-1,4-benzodioxane-2-carboxamide (1s)

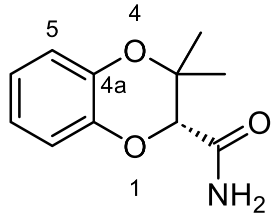

1s
$C_{11}H_{13}NO_3$
MW = 207.23 gmol^{-1}

According to GP F, a dried phototube (Ø = 1 cm) was charged with 3,3-dimethyl-2,3-dihydrobenzo[*b*][1,4]dioxine-2-carboxamide (*rac*-**1s**) (5.18 mg, 25.0 µmol, 1.00 equiv.) and enantiomerically pure (+)-benzophenone **2** (1.01 mg, 2.50 µmol, 10 mol%) under an argon atmosphere. In a separate vial, a stock solution of PhSH was prepared by dissolving PhSH (5.00 µL) in dry $PhCF_3$ (1.00 mL). From this stock solution, 103 µL (551 µg, 5.00 µmol, 20 mol%) was added followed by the addition of dry $PhCF_3$ (10 mL). The resulting solution was degassed by being sparged with argon under ultrasonication for 15 min and irradiated at λ = 366 nm at 30 °C for 18 h. After irradiation, the volatile compounds were removed under reduced pressure and the crude reaction mixture was

subjected to FCC (SiO_2, 50 → 100% EtOAc/hexanes) to yield (*R*)-3,3-dimethyl-2,3-dihydrobenzo[*b*][1,4]dioxine-2-carboxamide (**1s**) as a white solid (4.4 mg, 21.2 µmol, 85%, 88% *ee*).

TLC (40% EtOAc/hexanes): $R_f = 0.31$ [UV, $KMnO_4$].

Specific Rotation: $[\alpha]_D^{26}$: +56 (c = 0.5, $CHCl_3$) [88% *ee*].

Chiral HPLC: 88% *ee* (OD-RH 150 × 4.6 mm, MeCN/water = 20/80 → 100/0 over 30 min, 1 ml/min, λ = 210 nm); t_R = 8.65 min (major, **1s**), 9.61 min (minor, *ent*-**1s**).

(*R*)-2,3-Dihydrobenzo[*b*][1,4]oxazine-2-carboxamide (1t)

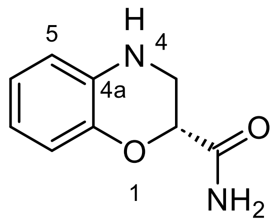

1t
$C_9H_{10}N_2O_2$
MW = 178.19 $gmol^{-1}$

According to GP F, a dried phototube (Ø = 1 cm) was charged with 2,3-dihydrobenzo[*b*][1,4]oxazine-2-carboxamide (*rac*-**1t**) (4.46 mg, 25.0 µmol, 1.00 equiv.) and enantiomerically pure (+)-benzophenone **2** (1.01 mg, 2.50 µmol, 10 mol%) under an argon atmosphere. In a separate vial, a stock solution of PhSH was prepared by dissolving PhSH (5.00 µL) in dry $PhCF_3$ (1.00 mL). From this stock solution, 103 µL (551 µg, 5.00 µmol, 20 mol%) was added followed by the addition of dry $PhCF_3$ (10 mL). The resulting solution was degassed by being sparged with argon under ultrasonication for 15 min and irradiated at λ = 366 nm at 30 °C for 18 h. After irradiation, the volatile compounds were removed under reduced pressure and the crude reaction mixture was subjected to FCC (SiO_2, 50 → 100% EtOAc/hexanes) to yield (*R*)-2,3-dihydrobenzo[*b*][1,4]oxazine-2-carboxamide (**1t**) as a white solid (3.20 mg, 18.0 µmol, 72%, 84% *ee*).

TLC (50% EtOAc/hexanes): $R_f = 0.20$ [UV, $KMnO_4$].

Specific Rotation: $[\alpha]_D^{26}$: +30 (c = 0.5, $CHCl_3$) [84% *ee*].

Chiral HPLC: 84% *ee* (AD-H 250 × 4.6 mm, *n*-heptane/^{i}PrOH = 70/30, 1 ml/min, λ = 210 nm); t_R = 6.71 min (major, **1t**), 9.39 min (minor, *ent*-**1t**).

(*R*)-4-Boc-2,3-dihydrobenzo[*b*][1,4]oxazine-2-carboxamide (1u)

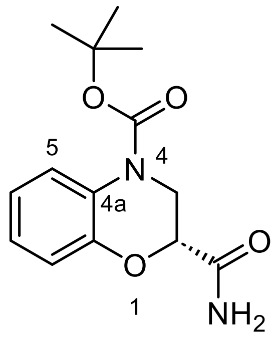

1u
$C_{14}H_{18}N_2O_4$
MW = 278.31 gmol^{-1}

According to GP F, a dried phototube (Ø = 1 cm) was charged with 4-Boc-2,3-dihydrobenzo[*b*][1,4]oxazine-2-carboxamide (*rac*-**1u**) (6.96 mg, 25.0 µmol, 1.00 equiv.) and enantiomerically pure (+)-benzophenone **2** (1.01 mg, 2.50 µmol, 10 mol%) under an argon atmosphere. In a separate vial, a stock solution of PhSH was prepared by dissolving PhSH (5.00 µL) in dry $PhCF_3$ (1.00 mL). From this stock solution, 103 µL (551 µg, 5.00 µmol, 20 mol%) was added followed by the addition of dry $PhCF_3$ (10 mL). The resulting solution was degassed by being sparged with argon under ultrasonication for 15 min and irradiated at λ = 366 nm at 30 °C for 18 h. After irradiation, the volatile compounds were removed under reduced pressure and the crude reaction mixture was subjected to FCC (SiO_2, 50 → 100% EtOAc/hexanes) to yield (*R*)-4-Boc-2,3-dihydrobenzo[*b*][1,4]oxazine-2-carboxamide (**1u**) as a white solid (6.02 mg, 21.6 µmol, 87%, 82% *ee*).

TLC (50% EtOAc/hexanes): R_f = 0.55 [UV, $KMnO_4$].

Specific Rotation: $[\alpha]_D^{25}$: +36 (*c* = 1.0, $CHCl_3$) [82% *ee*].

Chiral HPLC: 82% *ee* (AS-RH 150 × 4.6 mm, MeCN/water = 20/80 → 100/0 over 30 min, 1 ml/min, λ = 210 nm); t_R = 10.19 min (major, **1u**), 13.69 min (minor, *ent*-**1u**).

1-Boc-(*R*)-4-(1,4-benzodioxane-2-carbonyl)piperazine (7)

According to GP F, a dried phototube (Ø = 1 cm) was charged with 1-Boc-4-(1,4-benzodioxane-2-carbonyl)piperazine (*rac*-**7**) (8.71 mg, 25.0 µmol, 1.00 equiv.) and enantiomerically pure (+)-benzophenone **2** (1.01 mg, 2.50 µmol, 10 mol%) under an argon atmosphere. In a separate vial, a stock solution of PhSH was prepared by dissolving PhSH (5.00 µL) in dry $PhCF_3$ (1.00 mL). From this stock solution, 206 µL (1.10 mg, 10.0 µmol, 40 mol%) was added followed by the addition of dry $PhCF_3$ (10 mL). The resulting solution was degassed by being sparged with argon under ultrasonication for 15 min and irradiated at λ = 366 nm at 30 °C for 18 h. After irradiation, the volatile compounds were removed under reduced

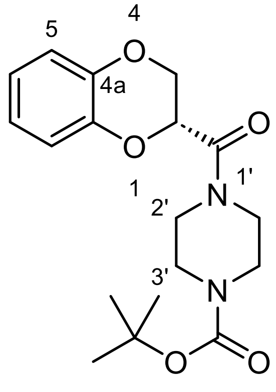

7
$C_{18}H_{24}N_2O_5$
MW = 348.40 gmol^{-1}

pressure and the crude reaction mixture was subjected to FCC (SiO_2, 50 → 80% EtOAc/hexanes) to yield 1-Boc-4-(1,4-benzodioxane-2-carbonyl)piperazine (**7**) as a colorless oil (8.28 mg, 23.8 µmol, 95%, <1% *ee*).

TLC (50% EtOAc/pentane): R_f = 0.4 [UV, $KMnO_4$].

Chiral HPLC: <1% *ee* (IA 250 × 4.6 mm, *n*-heptane/^{i}PrOH = 90/10, 1 ml/min, λ = 210 nm); t_R = 9.43 min, 11.31 min.

The results of this experiment established the importance of two-point hydrogen bonding for the designed deracemization reaction.

1,2,3,4-Tetrahydronaphthalene-2-carboxamide (12)

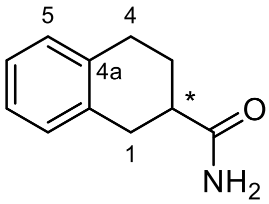

12
$C_{11}H_{13}NO$
MW = 175.23 gmol^{-1}

According to GP F, a dried phototube (Ø = 1 cm) was charged with 1,2,3,4-tetrahydronaphthalene-2-carboxamide (*rac*-**12**) (4.38 mg, 25.0 µmol, 1.00 equiv.) and enantiomerically pure (+)-benzophenone **2** (1.01 mg, 2.50 µmol, 10 mol%) under an argon atmosphere. In a separate vial, a stock solution of PhSH was prepared by dissolving PhSH (5.00 µL) in dry $PhCF_3$ (1.00 mL). From this stock solution, 103 µL (551 µg, 5.00 µmol, 20 mol%) was added followed by the addition of dry $PhCF_3$ (10 mL). The resulting solution was degassed by being sparged with argon under ultrasonication for 15 min and irradiated at λ = 366 nm at 30 °C for 18 h. After irradiation, the volatile compounds were removed under reduced pressure and the crude reaction mixture was subjected to FCC (SiO_2, 50 → 100% EtOAc/hexanes) to yield enantioenriched 1,2,3,4-tetrahydronaphthalene-2-carboxamide (**12**) as a white solid (3.45 mg, 19.7 µmol, 79%, 7% *ee*).

TLC (50% EtOAc/pentane): R_f = 0.2 [UV, $KMnO_4$].

Chiral HPLC: 7% *ee* (IC 250 × 4.6 mm, *n*-heptane/^{i}PrOH = 70/30, 1 ml/min, λ = 210 nm); t_R = 9.42 min (major, **12**), 10.50 min (minor, *ent*-**12**).

Thiochromane-2-carboxamide (13)

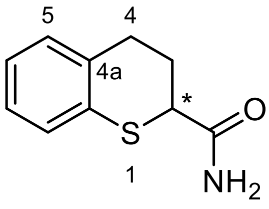

13
$C_{10}H_{11}NOS$
MW = 193.26 gmol^{-1}

According to GP F, a dried phototube (Ø = 1 cm) was charged with thiochramane-2-carboxamide (*rac*-**13**) (4.83 mg, 25.0 µmol, 1.00 equiv.) and enantiomerically pure (+)-benzophenone **2** (1.01 mg, 2.50 µmol, 10 mol%) under an argon atmosphere. In a separate vial, a stock solution of PhSH was prepared by dissolving PhSH (5.00 µL) in dry $PhCF_3$ (1.00 mL). From this stock solution, 103 µL (551 µg, 5.00 µmol, 20 mol%) was added followed by the addition of dry $PhCF_3$ (10 mL). The resulting solution was degassed by being sparged with argon under ultrasonication for 15 min and irradiated at λ = 366 nm at 30 °C for 18 h. After irradiation, the volatile compounds were removed under reduced pressure and the crude reaction mixture was subjected to FCC (SiO_2, 50 → 100% EtOAc/hexanes) to yield enantioenriched thiochromane-2-carboxamide (**13**) as a white solid (4.80 mg, 24.8 µmol, 99%, 14% *ee*).

TLC (50% EtOAc/hexanes): R_f = 0.40 [UV, $KMnO_4$].

Chiral HPLC: 14% *ee* (AD-H 250 × 4.6 mm, *n*-heptane/^{i}PrOH = 90/10, 1 ml/min, λ = 210 nm); t_R = 11.40 min (major, **13**), 12.31 min (minor, *ent*-**13**).

S11. Stereochemical Editing of *rac-cis*-1v and *rac-trans*-1v

The influence of an additional stereocenter in benzodioxane-2-carboxamide substrate **1r** on deracemization efficiency was investigated by subjecting both 3-methyl-substituted derivatives *rac-cis*-**1v** and *rac-trans*-**1v** to the substrate deracemization conditions (Scheme 4).

Matched case with *rac-cis*-1v

In case *rac-cis*-**1v** was subjected to the standard deracemization conditions, *cis*-**1v** prevailed as the main product with high *ee*. Binding of *ent-cis*-**1v** to catalyst **2** is ideal and therefore editing at both C2 (leading to *trans*-**1v**) and C3 (leading to *ent-trans*-**1v**, presumably via a radical intermediate and thiophenol-mediated bHAT) is possible. Both *trans*-**1v** and *ent-trans*-**1v** can again be edited, both cases resulting in the formation of *cis*-**1v**.

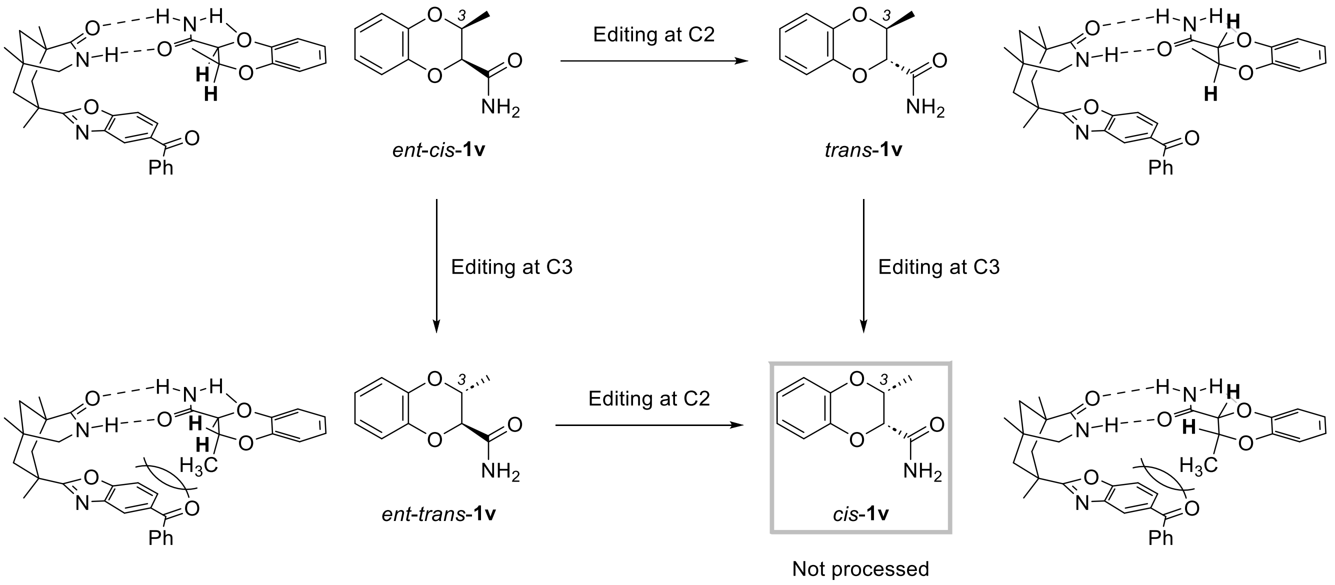

Fig. S20. Stereochemical editing of *rac-cis*-**1v** with chiral benzophenone catalyst **2** (for conditions see Scheme 4 in the main paper). Depicted are the possible editing steps of the substrate enantiomers, intermediate species as well as the structures of their association complexes with **2**.

Procedure for the stereochemical editing of *rac-cis*-1v:

According to GP F, a dried phototube (Ø = 1 cm) was charged with *rac-cis*-3-methyl-2,3-dihydrobenzo[*b*][1,4]dioxine-2-carboxamide (*rac-cis*-**1v**) (4.83 mg, 25.0 µmol, 1.00 equiv.) and enantiomerically pure (+)-benzophenone **2** (1.01 mg, 2.50 µmol, 10 mol%) under an argon atmosphere. In a separate vial, a stock solution of PhSH was prepared by dissolving PhSH (5.00 µL) in dry $PhCF_3$ (1.00 mL). From this stock solution, 206 µL (1.10 mg, 10.0 µmol,

40 mol%) were added followed by the addition of dry $PhCF_3$ (10 mL). The resulting solution was degassed by being sparged with argon under ultrasonication for 15 min and irradiated at λ = 366 nm at 30 °C for 18 h. After irradiation, the volatile compounds were removed under reduced pressure and the crude reaction mixture was subjected to FCC (SiO_2, 50 → 100% EtOAc/hexanes) to yield a mixture of *cis*-**1v** and *trans*-**1v** as a white solid (4.5 mg, 23.3 μmol, 93%, d.r. = 86/14 in favor of *cis*-**1v**). The diastereomeric ratio was determined by ^{1}H-NMR spectroscopy of the crude product. The two diastereoisomers were then separated by chiral preparative HPLC (AD-H 250 × 20 mm, *n*-heptane/^{i}PrOH = 90/10) for the *ee* determination of *cis*-**1v** and *trans*-**1v**.

TLC (50% EtOAc/hexanes): R_f = 0.30 [UV, $KMnO_4$].

Chiral HPLC for *cis*-**1v:** 87% *ee* (AS-RH 150 × 4.6 mm, MeCN/water = 20/80 → 100/0 over 30 min at 5 °C, 1 ml/min, λ = 210 nm); t_R = 9.00 min (major, *cis*-**1v**), 9.70 min (minor, *ent-cis*-**1v**).

Chiral HPLC for *trans*-**1v:** 78% *ee* (AD-H 250 × 4.6 mm, *n*-heptane/^{i}PrOH = 90/10, 1 ml/min, λ = 210 nm); t_R = 9.77 min (minor, *ent-trans*-**1v**), 10.32 min (major, *trans*-**1v**).

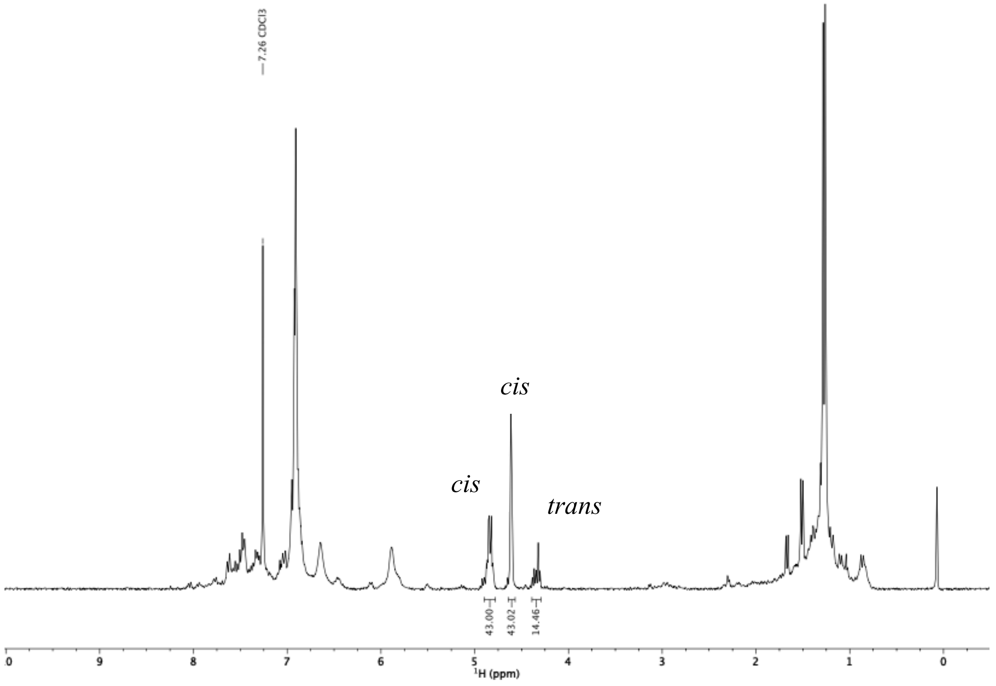

Fig. S21. Determination of the diastereomeric ratio after stereochemical editing of *rac-cis*-**1v** by ^{1}H-NMR. The NMR spectra were recorded in $CDCl_3$ on an AVHD300 Bruker NMR.

Mismatched case with *rac-trans*-1v

In case *rac*-*trans*-**1v** was subjected to the standard deracemization conditions, *cis*-**1v** was formed with very high *ee*, while a mixture of *ent*-*trans*-**1v** and *trans*-**1v** remained. Both enantiomers of *trans*-**1v** are converted to *cis*-**1v**, but presumably due to poor binding to the catalyst (in case of *ent*-*trans*-**1v**) or the lack of a bHAT motif (in case of *trans*-**1v**), stereochemical editing is not efficient for either case.

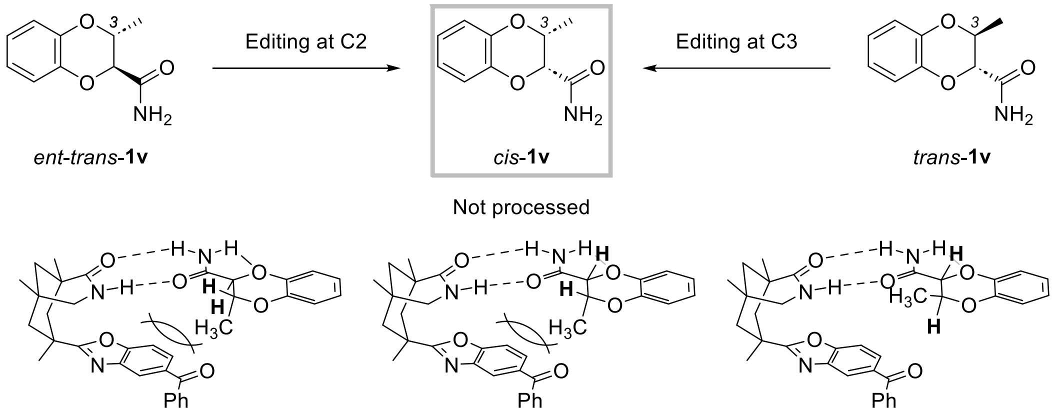

Fig. S22. Stereochemical editing of *rac*-*trans*-**1v** with chiral benzophenone catalyst **2** (for conditions see Scheme 4 in the main paper). Depicted are the possible editing steps of the substrate enantiomers as well as the structures of their association complexes with **2**.

Reaction with *rac-trans*-1v:

According to GP F, a dried phototube (Ø = 1 cm) was charged with *rac*-*trans*-3-methyl-2,3-dihydrobenzo[*b*][1,4]dioxine-2-carboxamide (*rac*-*trans*-**1v**) (4.83 mg, 25.0 μmol, 1.00 equiv.) and enantiomerically pure (+)-benzophenone **2** (1.01 mg, 2.50 μmol, 10 mol%) under an argon atmosphere. In a separate vial, a stock solution of PhSH was prepared by dissolving PhSH (5.00 μL) in dry $PhCF_3$ (1.00 mL). From this stock solution, 206 μL (1.10 mg, 10.0 μmol, 40 mol%) was added followed by the addition of dry $PhCF_3$ (10 mL). The resulting solution was degassed by being sparged with argon under ultrasonication for 15 min and irradiated at λ = 366 nm at 30 °C for 18 h. After irradiation, the volatile compounds were removed under reduced pressure and the crude reaction mixture was subjected to FCC (SiO_2, 50 → 100% EtOAc/hexanes) to yield mixture of *cis*-**1v** and *trans*-**1v** as a white solid (4.1 mg, 21.2 μmol, 85%, d.r. = 41/59 in favor of *trans*-**1v**). The diastereomeric ratio was determined by ^{1}H-NMR spectroscopy of the crude product. The two diastereoisomers were subsequently separated by

chiral preparative HPLC (AD-H 250 × 20 mm, *n*-heptane/^{i}PrOH = 90/10) for the *ee* determination of *cis*-**1v** and *trans*-**1v**.

TLC (50% EtOAc/hexanes): R_f = 0.30 [UV, $KMnO_4$].

Chiral HPLC for *cis*-**1v:** 96% *ee* (AS-RH 150 × 4.6 mm, MeCN/water = 20/80 → 100/0 over 30 min at 5 °C, 1 ml/min, λ = 210 nm); t_R = 8.99 min (major, *cis*-**1v**), 9.60 min (minor, *ent-cis*-**1v**).

Chiral HPLC for *trans*-**1v:** 23% *ee* (AD-H 250 × 4.6 mm, *n*-heptane/^{i}PrOH = 90/10, 1 ml/min, λ = 210 nm); t_R = 9.77 min (minor, *ent-trans*-**1v**), 10.31 min (major, *trans*-**1v**).

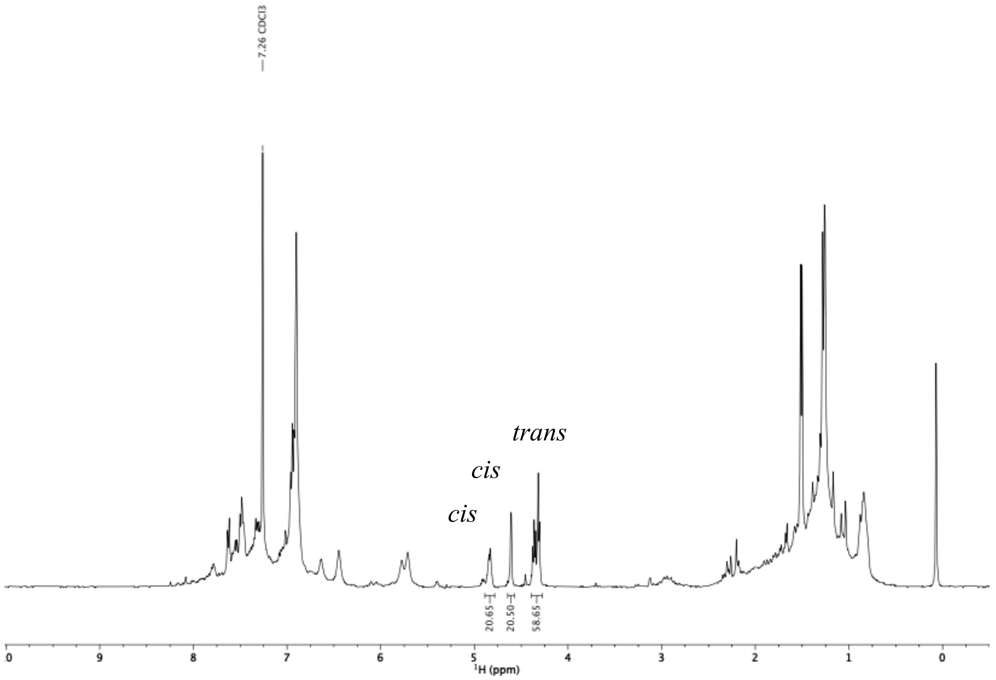

Fig. S23. Determination of the diastereomeric ratio after stereochemical editing of *rac-trans*-**1v** by ^{1}H-NMR. The NMR spectra were recorded in $CDCl_3$ on an AVHD300 Bruker NMR.

S12. Synthesis of APIs

Synthesis of Repinotan and Sarizotan

(*R*)-Chroman-2-ylmethanamine (3)

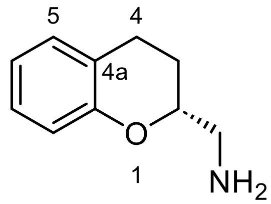

3

$C_{10}H_{13}NO$

MW = 163.22 gmol^{-1}

A solution of $LiAlH_4$ in THF (1.0 M, 53.5 mg, 1.41 mL, 1.41 mmol, 4.31 equiv.) was added dropwise to a solution of (*R*)-chroman-2-ylmethanamine (58.0 mg, 327 µmol, 1.00 equiv.) in THF (3.3 mL) under at 0 °C over a span of 5 min and the resulting solution was stirred at 0 °C for 15 h. EtOAc (10 mL) was slowly added at 0 °C followed by the addition of a saturated aqueous solution of Na_2SO_4 (10 mL). The resulting slurry was stirred for at 0 °C for 30 min and extracted thrice with EtOAC (3 × 10 mL). The combined organic layers were washed with brine and dried over Na_2SO_4 and the solvents were removed under reduced pressure. The crude product was subjected to FCC (SiO_2, 0 → 5% MeOH/CH_2Cl_2 + 1% Et_3N) to yield (*R*)-chroman-2-ylmethanamine (**3**) as a colorless oil (47.0 mg, 288 µmol, 88%). The compound was unstable under ambient conditions and was stored under argon at −20 °C.

TLC (10% MeOH/CH_2Cl_2): R_f = 0.3 [UV, $KMnO_4$].

^{1}H-NMR (400 MHz, $CDCl_3$, 300 K): δ [ppm] = 7.11 – 7.03 (m, 2H, H5, H7), 6.85 – 6.81 (m, 2H, H6, H8), 3.97 (*virt.* dtd, ^3J = 10.6 Hz, $^3J \approx {}^3J$ = 5.4 Hz, ^3J = 2.2 Hz, 1H, H2), 2.95 – 2.93 (m, 2H, CH_2NH$_2$), 2.92 – 2.84 (m, 2H, H4^a), 2.76 (ddd, ^2J = 16.4 Hz, ^3J = 5.7 Hz, ^3J = 3.0 Hz, 1H, H4^b), 1.96 (*virt.* ddt, ^2J = 13.4 Hz, ^3J = 6.1 Hz, $^3J \approx {}^3J$ = 2.8 Hz, 1H, H3^a), 1.78 (dddd, ^2J = 13.4 Hz, ^3J = 11.6 Hz, ^3J = 10.6 Hz, ^3J = 5.7 Hz, 1H, H3^b).

^{13}C-NMR (101 MHz, $CDCl_3$, 300 K): δ [ppm] = 154.9 (C8a), 129.6 (C5), 127.4 (C7), 122.1 (C4a), 120.3 (C6), 116.8 (C8), 77.7 (C2), 46.9 (CH_2NH_2), 25.2 (C3), 24.8 (C4).

HRMS (ESI) *m/z* $[M+H]^+$: calculated for $[C_{10}H_{14}NO]^+$: 164.1070; found: 164.1071.

IR (film) $\tilde{\nu}_{max}$/cm^{-1} = 3371 (w, NH), 3292 (w, NH), 3039 (w, CH_{arom}), 3022 (w, CH_{arom}) 2924 (w, CH_{aliph}), 2851 (w, CH_{aliph}), 1582 (m, C=C), 1488 (s, CH_{arom}), 1457 (s, CH_{aliph}), 1234 (s, C–O), 1112 (w, C–N).

Specific Rotation: $[\alpha]_D^{27}$: +110 (c = 1.0, THF).

2-(4-Bromobutyl)benzo[*d*]isothiazol-3(2*H*)-one 1,1-dioxide (4)

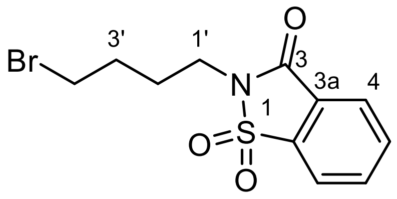

4

$C_{11}H_{12}BrNO_3S$

MW = 318.19 gmol^{-1}

1,4-Dibromobutane (2.65 g, 1.47 mL, 12.3 mmol, 3.00 equiv.) and anhydrous K_2CO_3 (2.26 g, 16.4 mmol, 4.00 equiv.) were added to a solution of saccharin (750 mg, 4.09 mmol, 1.00 equiv.) in dry acetonitrile (4.1 mL). The mixture was heated to reflux for 12 h and cooled to r.t., and poured into water in one portion. The resulting solution was extracted with CH_2Cl_2 (3 × 15 mL). The combined organic layers were washed with brine and dried over Na_2SO_4 before the solvents were removed under reduced pressure. The crude product was subjected to FCC (SiO_2, 0 → 20% EtOAc/hexanes) to yield 2-(4-bromobutyl)benzo[*d*]isothiazol-3(2*H*)-one 1,1-dioxide as a colorless thick oil (1.10 g, 3.46 mmol, 84%).

TLC (25% EtOAc/hexanes): R_f = 0.5 [UV].

^{1}H-NMR (400 MHz, $CDCl_3$, 300 K): δ [ppm] = 8.05 (dd, ^3J = 7.3 Hz, ^2J = 1.0 Hz, 1H, H4), 7.93 – 7.81 (m, 3H, H5, H6, H7), 3.83 – 3.80 (m, 2H, H1'), 3.47 – 3.44 (m, 2H, H4'), 2.06 – 1.94 (m, 4H, H2', H3').

^{13}C-NMR (101 MHz, $CDCl_3$, 300 K): δ [ppm] = 159.1 (C3), 137.8 (C7a), 134.9 (C6), 134.5 (C5), 127.4 (C3a), 125.3 (C4), 121.1 (C7), 38.5 (C1'), 32.7 (C4'), 29.9 (C2'), 27.2 (C3').

HRMS (ESI) *m/z* $[M+CH_3OH+H]^+$ calculated for $[C_{12}H_{17}{}^{79}BrNO_4S]^+$: 350.0056; found: 350.0049.

IR (film) $\tilde{\nu}_{max}/cm^{-1}$ = 3093 (w, CH_{arom}), 3022 (w, CH_{arom}) 2937 (w, CH_{aliph}), 2872 (w, CH_{aliph}), 1726 (s, C), 1595 (m, C=C), 1329 (s, S=O), 1254 (s, C–O), 1126 (s, C–N), 675 (s, C–Br).

(*R*)-2-(4-((Chroman-2-ylmethyl)amino)butyl)benzo[*d*]isothiazol-3(2*H*)-one 1,1-dioxide (Repinotan)

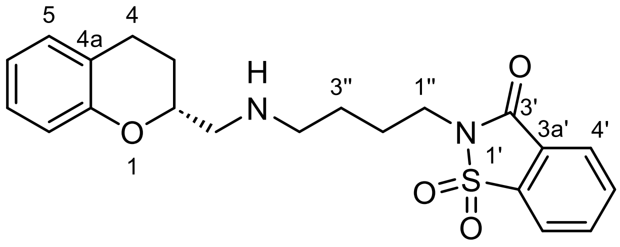

Repinotan
$C_{21}H_{24}N_2O_4S$
MW = 400.49 gmol^{-1}

Et_3N (6.51 mg, 8.97 µL, 64.3 µmol, 1.00 equiv.) was added dropwise to a solution of (*R*)-chroman-2-ylmethanamine (**3**) (11.6 mg, 70.8 µmol, 1.1 equiv.) in DMSO (0.50 mL) at r.t. and the resulting solution was heated to 50 °C. Then, a solution of 2-(4-bromobutyl)-1,1-dioxo-1,2-benzothiazol-3-one (4) (20.5 mg, 64.3 µmol, 1.00 equiv.) in DMSO (1.0 mL) was added dropwise over a span of 45 min and the resulting mixture was heated to 60 °C and stirred at this temperature for 2.5 h. The reaction mixture was then allowed to cool to r.t. Water (3 mL) was added and the mixture was extracted thrice with EtOAc (3 × 5 mL). The combined organic layers were washed with brine and dried over Na_2SO_4 before the solvents were removed under reduced pressure. The crude product was subjected to FCC (SiO_2, 0 → 5% MeOH/EtOAc) to yield (*R*)-2-(4-((chroman-2-ylmethyl)amino)butyl)benzo[*d*]isothiazol-3(2*H*)-one 1,1-dioxide (Repinotan) as a colorless oil (21.0 mg, 52.4 µmol, 82%).

TLC (15% MeOH/ EtOAc): R_f = 0.5 [UV, $KMnO_4$].

^{1}H-NMR (400 MHz, $CDCl_3$, 300 K): δ [ppm] = 8.06 – 8.05 (m, 1H, H4'), 7.92 – 7.90 (m, 1H, H7'), 7.87 – 7.79 (m, 2H, H5', H6'), 7.09 – 7.01 (m, 2H, H5, H7), 6.84 – 6.80 (m, 2H, H6, H8), 4.20 – 4.14 (m, 1H, H2), 3.81 (t, ^3J = 7.4 Hz, 2H, H1"), 2.94 – 2.70 (m, 6H, H4, C2CH$_2$, H4"), 2.01 – 1.89 (m, 3H, H3^a, H3"), 1.83 – 1.64 (m, 3H, H3^b, H2").

^{13}C-NMR (101 MHz, $CDCl_3$, 300 K): δ [ppm] = 159.1 (C3'), 154.7 (C8a), 137.9 (C7'a), 134.8 (C6'), 134.4 (C5'), 129.6 (C5), 127.6 (C3'a), 127.3 (C7), 125.3 (C4'), 122.1 (C4a), 121.0 (C7'), 120.3 (C6), 116.9 (C8), 74.9 (C2), 54.0 (C2*C*H$_2$), 49.2 (C4"), 39.3 (C1"), 27.0 (C2"), 26.3 (C3"), 25.7 (C3), 24.7 (C4).

HRMS (ESI) *m/z* [M+H]$^+$ calculated for $[C_{21}H_{25}N_2O_4S]^+$: 401.1530; found: 401.1513.

Specific Rotation: $[\alpha]_D^{23}$: −50 (*c* = 0.6, CH_2Cl_2).

The spectroscopic data matches the one reported in the literature.[27]

5-(4-Fluorophenyl)nicotinaldehyde (5)

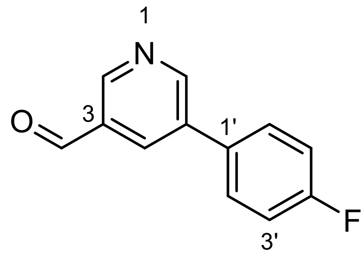

5
$C_{12}H_8FNO$
MW = 201.20 gmol^{-1}

Following a procedure by Yin *et al.*,[101] a mixture of 5-bromonicotinaldehyde (500 mg, 2.69 mmol, 1.00 equiv.), (4-fluorophenyl)boronic acid (564 mg, 4.03 mmol, 1.50 equiv.), K_2CO_3 (1.49 g, 10.8 mmol, 4.00 equiv.), bis(triphenylphosphine)palladium(II)-dichloride (189 mg, 269 µmol, 10 mol%) and tricyclohexylphosphane (151 mg, 538 µmol, 20 mol%) in 1,4-dioxane (5.4 mL) and water (1.8 mL) was degassed by being sparged with argon for 15 min. The yellow suspension was then stirred at 90 °C for 3 h before it was allowed to cool to r.t. again. Water (5 mL) was added, the layers were separated and the aqueous layer was extracted thrice with EtOAc (3 × 5 mL). The combined organic layers were washed with brine and dried over Na_2SO_4 before the remaining solvents were removed under reduced pressure. The obtained crude product was subjected to FCC (SiO_2, 20 → 50% EtOAc/hexanes) to yield 5-(4-fluorophenyl)nicotinaldehyde (**5**) as a slightly yellow solid (518 mg, 2.57 mmol, 96%).

TLC (30% EtOAc/hexanes): R_f = 0.26 [UV, $KMnO_4$].

^{1}H-NMR (400 MHz, $CDCl_3$, 300 K): δ [ppm] = 10.2 (s, 1H, CHO), 9.04 – 9.03 (m, 2H, H2, H6), 8.30 (*virt.* t, $^4J \approx {}^4J$ = 2.2 Hz, 1H, H4), 7.62 – 7.57 (m, 2H, H2'), 7.24 – 7.18 (m, 2H, H3').

^{13}C-NMR (101 MHz, $CDCl_3$, 300 K): δ [ppm] = 190.7 (CHO), 163.5 (d, $^1J_{CF}$ = 249 Hz, C4'), 153.2 (C6), 150.9 (C2), 136.2 (C5), 133.5 (C4), 132.6 (d, $^4J_{CF}$ = 3.4 Hz, C1'), 131.5 (C3), 129.2 ($^3J_{CF}$ = 8.5 Hz, C2'), 116.6 ($^2J_{CF}$ = 21.7 Hz, C3').

^{19}F-NMR (376 MHz, $CDCl_3$, 300 K): δ [ppm] = −112.7 – −112.8 (m).

The spectroscopic data matches the one reported in the literature.[102]

(*R*)-1-(Chroman-2-yl)-*N*-((5-(4-fluorophenyl)pyridin-3-yl)methyl)methanamine (Sarizotan)

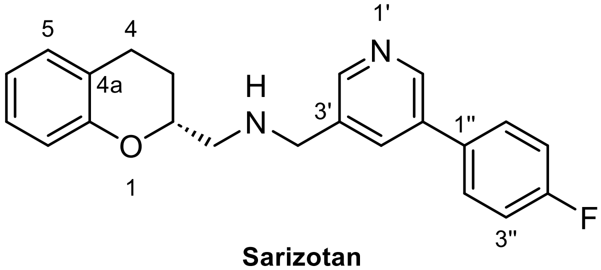

Sarizotan
$C_{22}H_{21}FN_2O$
MW = 348.42 gmol^{-1}

para-Toluenesulfonic acid monohydrate (2.68 mg, 14.1 µmol, 0.10 equiv.) was added to a solution of (*R*)-chroman-2-ylmethanamine (**3**) (23.0 mg, 141 µmol, 1.00 equiv.) and 5-(4-fluorophenyl)nicotinaldehyde (**5**) (31.2 mg, 155 µmol, 1.10 equiv.) in MeOH (700 µL) and

the resulting orange solution was stirred at r.t. for 14 h. Then, $NaBH_4$ (10.7 mg, 282 μmol, 2.00 equiv.) was added to the reaction mixture at 0 °C the resulting brown suspension was stirred at 0 °C for 2.5 h. Water (1 mL) was added and the mixture was extracted thrice with EtOAc (3 × 3 mL). The combined organic layers were washed with brine and dried over Na_2SO_4 and the remaining solvents were removed under reduced pressure. The obtained crude product was then subjected to FCC (SiO_2, 0 → 5% MeOH/EtOAc) to yield (*R*)-1-(chroman-2-yl)-*N*-((5-(4-fluorophenyl)pyridin-3-yl)methyl)methanamine (Sarizotan) (46.1 mg, 132 μmol, 94%, 99% *ee*) as a colorless oil.

TLC (5% MeOH/EtOAc): R_f = 0.15 [UV, $KMnO_4$].

^{1}H-NMR (500 MHz, $CDCl_3$, 300 K): δ [ppm] = 8.73 (d, ^4J = 2.1 Hz, 1H, H6'), 8.58 (d, ^4J = 2.1 Hz, 1H, H2'), 8.03 (*virt.* t, $^4J \approx {}^4J$ = 2.1 Hz, H4'), 7.59 – 7.56 (m, 2H, H2''), 7.18 – 7.13 (m, 2H, H3''), 7.08 – 7.04 (m, 1H, H7), 7.02 – 7.01 (m, 1H, H5), 6.85 – 6.82 (m, 2H, H6, H8), 4.28 – 4.24 (m, 1H, H2), 4.05 (*virt.* s, 2H, C3'CH_2), 2.95 – 2.94 (m, 2H, C2CH_2), 2.81 (ddd, ^2J = 17.0 Hz, ^3J = 11.5 Hz, ^3J = 5.9 Hz, 1H, $H4^a$), 2.72(ddd, ^2J = 17.0 Hz, ^3J = 5.8 Hz, ^3J = 2.9 Hz, 1H, $H4^b$), 2.06 (s, 1H, NH), 1.95 (*virt.* ddt, ^2J = 13.5 Hz, ^3J = 5.8 Hz, $^3J \approx {}^3J$ = 2.7 Hz, 1H, $H3^b$), 1.79 (*virt.* dtd, ^2J = 13.5 Hz, $^3J \approx {}^3J$ = 11.1 Hz, ^3J = 5.9 Hz, 1H, $H3^a$).

^{13}C-NMR (126 MHz, $CDCl_3$, 300 K): δ [ppm] = 163.1 (d, $^1J_{CF}$ = 248 Hz, C4''), 154.3 (C8a), 148.5 (C2'), 147.2 (C6'), 135.9 (C5'), 135.0 (C4'), 133.8 (C3'), 133.6 (d, $^4J_{CF}$ = 3.3 Hz, C1''), 129.7 (C5), 129.0 (d, $^3J_{CF}$ = 8.3 Hz, C2''), 127.4 (C7), 121.9 (C4a), 120.6 (C6), 116.9 (C8), 116.2 (d, $^2J_{CF}$ = 21.6 Hz, C3''), 74.3 (C2), 52.9 (C2*C*H_2), 50.5 (C3'*C*H_2), 25.6 (C3), 24.5 (C4).

^{19}F-NMR (471 MHz, $CDCl_3$, 300 K): δ [ppm] = −113.8 – −113.9 (m).

HRMS (ESI) *m/z* $[M+H]^+$ calculated for $[C_{22}H_{22}FN_2O]^+$: 349.1711; found: 349.1711.

IR (film) $\tilde{\nu}_{max}$/cm^{-1} = 3320 (w, NH), 3039 (w, CH_{arom}), 3024 (w, CH_{arom}), 2928 (w, CH_{aliph}), 2849 (w, CH_{aliph}), 1606 (m, C=C), 1582 (m, C=C), 1514 (s, CH_{arom}), 1488 (m, CH_{arom}), 1456 (m, CH_{aliph}), 1232 (s, C–O), 1160 (m, C–F), 1112 (m, C–N).

Specific Rotation: $[\alpha]_D^{25}$: −50 (*c* = 1.05, CH_2Cl_2) [99% *ee*].

Chiral HPLC: 99% *ee* (OD-H 250 × 4.6 mm, *n*-heptane/^{i}PrOH = 70/30, 1 ml/min, λ = 210 nm); t_R = 12.31 min (minor, *ent*-Sarizotan), 15.91 min (major, Sarizotan).

Synthesis of Doxazosin · *HCl*

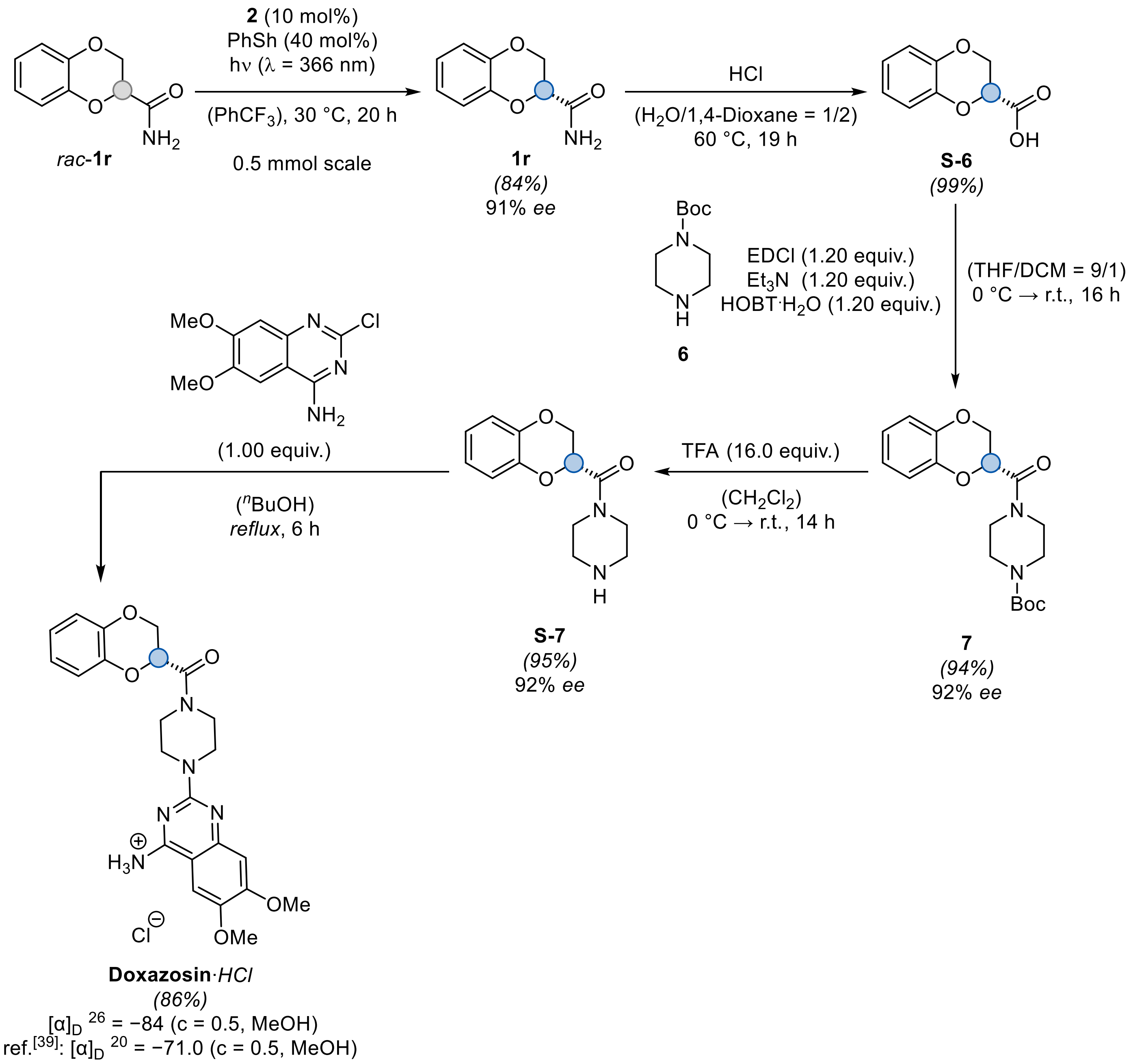

(*R*)-1,4-Benzodioxane-2-carboxylic acid (S-6)

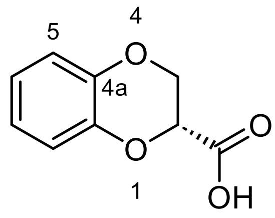

S-6
$C_9H_8O_4$
MW = 180.16 $gmol^{-1}$

A solution of (*R*)-1,4-benzodioxane-2-carboxamide (**1r**) (72.0 mg, 402 µmol, 1.00 equiv.) in aq. HCl (12 M, 440 mg, 1.00 mL, 12.1 mmol, 30.0 equiv.) and HCl in 1,4-dioxane (4.0 M, 293 mg, 2.01 mL, 8.04 mmol, 20.0 equiv.) was heated to 60 °C for 19 h. After cooling to r.t., the mixture was diluted with water (10 mL) and then extracted thrice with EtOAc (3 × 10 mL). The combined organic layers were washed with brine and dried over Na_2SO_4 to afford (*R*)-1,4-benzodioxane-2-carboxylic acid (**S-6**) as a white solid (72.0 mg, 400 µmol, 99%).

^{1}H-NMR (400 MHz, $CDCl_3$, 300 K): δ [ppm] = 7.01 – 6.98 (m, 1H, H5/H8), 6.93 – 6.88 (m, 3H, H5/H8, H6, H7), 4.90 (dd, ^3J = 4.5 Hz, ^3J = 3.0 Hz, 1H, H2), 4.46 – 4.38 (m, 2H, H3).

^{13}C-NMR (101 MHz, $CDCl_3$, 300 K): δ [ppm] = 173.3 (COOH), 143.0 (C4a), 142.1 (C8a), 122.5 (C6/C7), 122.3 (C6/C7), 117.6 (C5/C8), 117.5 (C5/C8), 71.7 (C2), 64.7 (C3).

Specific Rotation: $[a]_D^{25}$: +60 (*c* = 1.0, $CHCl_3$).

The spectroscopic data matches the one reported in the literature.[39]

1-Boc-(*R*)-4-(1,4-benzodioxane-2-carbonyl)piperazine (7)

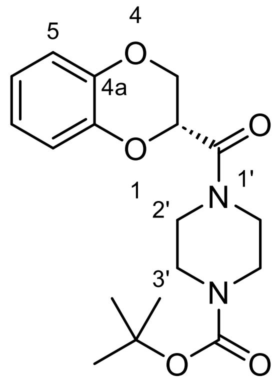

7
$C_{18}H_{24}N_2O_5$
MW = 348.40 $gmol^{-1}$

Following a modified procedure by Rouf *et al.*,[103] HOBt · H_2O (71.4 mg, 466 µmol, 1.20 equiv.) was added to a solution of (*R*)-1,4-benzodioxane-2-carboxylic acid (**S-6**) (70.0 mg, 389 µmol, 1.00 equiv.) in THF (2.5 mL) and CH_2Cl_2 (250 µL) at 0 °C. After 10 min, EDCI · HCl (89.4 mg, 466 µmol, 1.20 equiv.) and Et_3N (47.2 mg, 65.0 µL, 466 µmol, 1.20 equiv.) were added and the resulting mixture was stirred for another 5 min at 0 °C. Then, 1-Boc-piperazine (**6**) (181 mg, 971 µmol, 2.50 equiv.) was added and the reaction mixture was allowed to warm to r.t. and stirred for 16 h. The reaction was quenched by the addition of sat. aqueous NH_4Cl (10 mL). CH_2Cl_2 (10 mL) was added and the layers were separated. The aqueous layer was then extracted thrice with DCM (3 × 5 mL) and the combined org layers were washed with brine and dried over Na_2SO_4. The solvents were removed under reduced pressure and the crude product was subjected to FCC (SiO_2, 20 → 60% EtOAc/hexanes) to yield 1-Boc-(*R*)-4-

(1,4-benzodioxane-2-carbonyl)piperazine (**7**) as a colorless oil (127.0 mg, 365 µmol, 94%, 92% *ee*).

TLC (50% EtOAc/hexanes): R_f = 0.4 [UV, $KMnO_4$].

^{1}H-NMR (400 MHz, $CDCl_3$, 300 K): δ [ppm] = 6.93 – 6.83 (m, 4H, H5, H6, H7, H8), 4.82 (dd, ^3J = 8.0 Hz, ^3J = 2.6 Hz, 1H, H2), 4.49 (dd, ^2J = 12.0 Hz, ^3J = 2.6 Hz, 1H, H3^a), 4.34 (dd, ^2J = 12.0 Hz, ^3J = 8.0 Hz, 1H, H3^b), 3.81 – 3.78 (m, 2H, H2'^{a}, H3'^{a}), 3.65 – 3.32 (6H, H2'^{a}, H3'^{a}, H2'^{b}, H3'^{b}), 1.48 (s, 9H, $(CH_3)_3$).

^{13}C-NMR (101 MHz, $CDCl_3$, 300 K): δ [ppm] = 165.3 (C_2*C*ON), 154.7 (NCOO), 143.5 (C4a), 142.5 (C8a), 122.5 (C6/C7), 121.7 (C6/C7), 117.6 (C5/C8), 117.4 (C5/C8), 80.6 (*C*$(CH_3)_3$), 70.9 (C2), 65.3 (C3), 45.8 (C2', C3'), 42.2 (C2', C3'), 28.5 ($(CH_3)_3$).

Specific Rotation: $[a]_D^{26}$: −48 (*c* = 1.0, $CHCl_3$) [92% *ee*].

Chiral HPLC: 92% *ee* (IA 250 × 4.6 mm, *n*-heptane/^{i}PrOH = 90/10, 1 ml/min, λ = 210 nm); t_R = 9.43 min (minor, *ent*-**7**), 11.31 min (major, **7**).

The spectroscopic data matches the one reported in the literature.[39]

(*R*)-4-(1,4-Benzodioxane-2-carbonyl)piperazine (S-7)

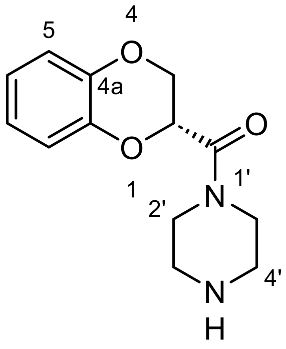

S-7
$C_{13}H_{16}N_2O_3$
MW = 248.28 gmol^{-1}

Following a modified procedure by Yin *et al.*,[39] 2,2,2-trifluoroacetic acid (417 mg, 317 µL, 4.13 mmol, 16.0 equiv.) was slowly added to a solution of 1-Boc-(*R*)-4-(1,4-benzodioxane-2-carbonyl)piperazine (**7**) (90.0 mg, 258 µmol, 1.00 equiv.) in CH_2Cl_2 (2.5 mL) at 0 °C. The resulting reaction mixture was allowed to warm to r.t. and stirred for 14 h before it was diluted with water (10 mL). The reaction was quenched by the addition of an aqueous $NaHCO_3$ solution (10%, 10 mL) before the layers were separated and the aqueous layer was extracted thrice with with CH_2Cl_2 (3 × 10 mL). The combined organic layers were washed with brine and dried over Na_2SO_4. The remaining solvents were removed under reduced pressure and the crude product was subjected to FCC (SiO_2, 0 → 10% MeOH/CH_2Cl_2 + 1% Et_3N) to yield (*R*)-4-(1,4-benzodioxane-2-carbonyl)piperazine (**S-7**) as a colorless oil (61.0 mg, 246 µmol, 95%, 92% *ee*).

TLC (10% MeOH/CH_2Cl_2): R_f = 0.50 [UV, $KMnO_4$].

^{1}H-NMR (400 MHz, $CDCl_3$, 300 K): δ [ppm] = 6.91 – 6.81 (m, 4H, H5, H6, H7, H8), 4.81 (dd, ^3J = 8.1 Hz, ^3J = 2.5 Hz, 1H, H2), 4.48 (dd, ^2J = 11.9 Hz, ^3J = 2.5 Hz, 1H, H3^a), 4.32 (dd,

^2J = 11.9 Hz, ^3J = 8.1 Hz, 1H, H3[b]), 3.77 – 3.70 (m, 2H, H2’[a], H5‘[a]), 3.57 – 3.49 (m, 2H, H2’[b], H5‘[b]), 3.00 – 2.88 (4H, H3‘, H4‘).

^{13}C-NMR (101 MHz, $CDCl_3$, 300 K): δ [ppm] = 165.0 (CON), 143.4 (C4a), 142.7 (C8a), 122.3 (C6/C7), 121.6 (C6/C7), 117.5 (C5/C8), 117.4 (C5/C8), 70.7 (C2), 65.4 (C3), 47.2 (C2’/C5’), 46.6 (C3’/C4’), 45.9 (C3’/C4’), 43.4 (C2’/C5’).

Specific Rotation: $[\alpha]_D^{26}$: −76 (*c* = 1.0, $CHCl_3$) [92% *ee*].

Chiral HPLC: 92% *ee* (IC 250 × 4.6 mm, *n*-heptane/^{i}PrOH = 50/50, 1 ml/min, λ = 210 nm); t_R = 13.40 min (minor, *ent*-**S-7**), 15.66 min (major, **S-7**).

The spectroscopic data matches the one reported in the literature.[39]

(*R*)-2-(4-(1,4-Benzodioxane-2-carbonyl)piperazin-1-yl)-6,7-dimethoxyquinazolin-4-ammonium chloride (Doxazosin · HCl)

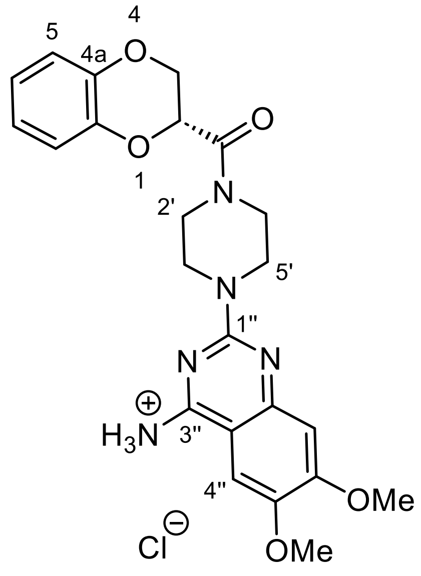

Doxazosin·*HCl*
$C_{23}H_{26}ClN_5O_5$
MW = 487.94 gmol^{-1}

Following a procedure by Yin *et al.,*[39] a solution of 2-chloro-6,7-dimethoxyquinazolin-4-amine (24.0 mg, 100 mmol, 1.00 equiv.) and (*R*)-4-(1,4-benzodioxane-2-carbonyl)piperazine (**S-8**) (29.8 mg, 120 µmol, 1.20 equiv.) in ^{n}BuOH (3.0 mL) was heated to reflux for 5 h. Then, the reaction mixture was allowed to cool to r.t. and the formed precipitate was collected, washed with ^{n}BuOH (12 mL) and dried under reduced pressure to obtain (*R*)-2-(4-(1,4-benzodioxane-2-carbonyl)piperazin-1-yl)-6,7-dimethoxyquinazolin-4-ammonium chloride (Doxazosin · HCl) as a white solid (42.0 mg, 86.1 µmol, 86%).

M.p.: > 230 °C

^{1}H-NMR (500 MHz, DMSO-d$_6$, 300 K): δ [ppm] = 12.38 (s, 1H, NH_3^+), 8.96 (bs, 1H, NH_3^+), 8.71 (bs, 1H, NH_3^+), 7.76 (s, 1H, H4”), 7.57 (s, 1H, H7”), 6.95 – 6.84 (m, 4H, H5, H6, H7, H8), 5.34 (dd, ^3J = 6.5 Hz, ^3J = 2.6 Hz, 1H, H2), 4.42 (dd, ^2J = 11.8 Hz, ^3J = 2.6 Hz, 1H, H3[a]), 4.22 (dd, ^2J = 11.8 Hz, ^3J = 6.5 Hz, 1H, H3[a]), 4.05 – 3.80 (m, 12H, OCH_3, H2’, H3’[a], H5’[a], H6’), 3.74 – 3.60 (m, 2H, H3’[b], H5’[b]).

^{13}C-NMR (126 MHz, DMSO-d$_6$, 300 K): δ [ppm] = 165.3 (CON), 161.3 (C3”), 155.3 (C6”), 151.4 (C1”), 146.9 (C5”), 143.1 (C4a), 142.9 (C8a), 136.2 (C7”[a]), 121.6 (C6/C7), 121.5 (C6/C7),

117.1 (C5/C8), 117.0 (C5/C8), 104.9 (C4''), 101.8 (C3''[a]), 99.2 (C7''), 69.4 (C2), 64.7 (C3), 56.3 (C5''O*C*H_3), 56.1 (C6''O*C*H_3), 44.8 (C3'/C5'), 44.2 (C2'/C6'), 44.1 (C2'/C6'), 40.8 (C3'/C5').
HRMS (ESI) *m/z* $[M-Cl]^+$: calculated for $[C_{23}H_{26}N_5O_5]^+$: 452.1928; found: 452.1922.
IR (film) $\tilde{\nu}_{max}/cm^{-1}$ = 3299 (w, NH), 3120 (m, NH), 3020 (w, CH_{arom}), 2942 (m, CH_{aliph}), 2839 (w, CH_{aliph}), 1675 (m, C=O), 1641 (m, C=N), 1630 (m, C=N), 1592 (s, C=C), 1496 (s, CH_{arom}), 1434 (s, CH_{aliph}), 1282 (s, C–O), 1250 (s, C–O), 1225 (s, C–O), 1109 (m, C–N), 1092 (m, C–N).
Specific Rotation: $[\alpha]_D^{26}$: −84 (*c* = 0.5, MeOH).

Synthesis of (*S*, *R*, *R*, *R*)-Nebivolol · *HCl*

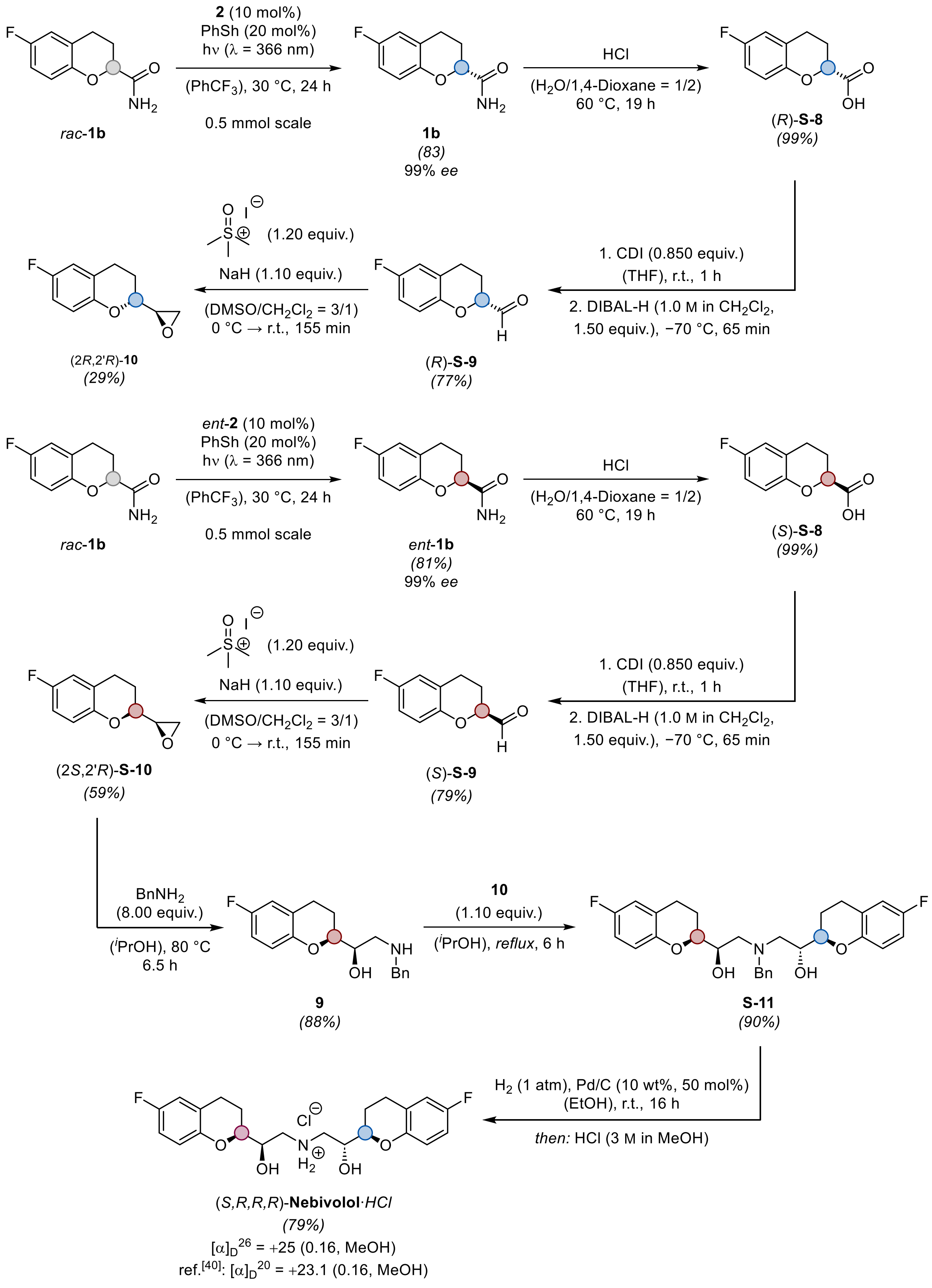

(*R*)-6-Fluorochromane-2-carboxylic acid ((*R*)-S-8)

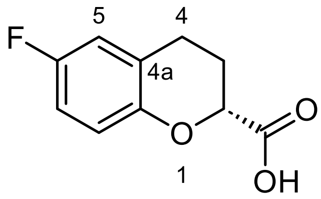

(*R*)-**S-8**
$C_{10}H_9O_3$
MW = 196.18 $gmol^{-1}$

A solution of (*R*)-6-fluorochromane-2-carboxamide (**1b**) (140 mg, 717 µmol, 1.00 equiv.) in aq. HCl (12 M, 784 mg, 1.79 mL, 21.5 mmol, 30.0 equiv.) and HCl in 1,4-dioxane (4.0 M, 523 mg, 3.59 mL, 14.3 mmol, 20.0 equiv.) was heated to 60 °C for 19 h. After cooling to r.t., the mixture was diluted with water (15 mL) and then extracted thrice with EtOAc (3 × 15 mL). The combined organic layers were washed with brine and dried over Na_2SO_4 to afford (*S*)-6-fluorochromane-2-carboxylic acid ((*R*)-**S-8**) as a white solid (140 mg, 714 µmol, 99%).

TLC (3% MeOH/CH_2Cl_2): R_f = 0.22 [UV, $KMnO_4$].

^{1}H-NMR (400 MHz, $CDCl_3$, 300 K): δ [ppm] = 9.56 (bs, 1H. COOH), 6.89 – 6.80 (m, 2H, H7, H8), 6.76 (dd, $^3J_{HF}$ = 9.0 Hz, $^4J_{HH}$ = 2.8 Hz, 1H, H5), 4.75 (dd, ^3J = 7.7 Hz, ^3J = 3.6 Hz, 1H, H2), 2.90 – 2.75 (m, 2H, H4), 2.33 (*virt.* dtd, ^2J = 13.8 Hz, $^3J \approx {}^3J$ = 6.3 Hz, ^3J = 3.6 Hz, 1H, $H3^a$), 2.19 (*virt.* dtd, ^2J = 13.8 Hz, $^3J \approx {}^3J$ = 7.9 Hz, ^3J = 5.8 Hz, 1H, $H3^b$).

^{13}C-NMR (101 MHz, $CDCl_3$, 300 K): δ [ppm] = 176.2 (COOH), 157.3 (d, $^1J_{CF}$ = 239 Hz, C6), 149.1 (d, $^4J_{CF}$ = 2.1 Hz, C8a), 122.4 (d, $^3J_{CF}$ = 7.5 Hz, C4a), 118.0 (d, $^3J_{CF}$ = 8.2 Hz, C8), 115.5 (d, $^2J_{CF}$ = 22.7 Hz, C5), 114.6 (d, $^2J_{CF}$ = 23.4 Hz, C7), 73.3 (C2), 24.3 (C3), 23.6 (d, $^4J_{CF}$ = 1.3 Hz, C4).

^{19}F-NMR (376 MHz, $CDCl_3$, 300 K): δ [ppm] = −123.1 – −123.2 (m).

Specific Rotation: $[\alpha]_D^{27}$: +26 (*c* = 1.0, $CHCl_3$).

The spectroscopic data matches the one in the literature.[29]

(*R*)-6-Fluorochromane-2-carbaldehyde ((*R*)-S-9)

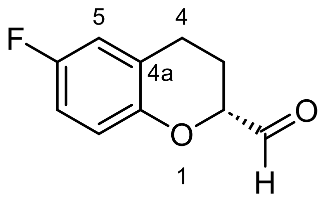

(*R*)-**S-9**
$C_{10}H_9O_2$
MW = 180.18 $gmol^{-1}$

1,1'-Carbonyldiimidazole (CDI) (94.8 mg, 585 µmol, 0.850 equiv.) was added to a solution of (*R*)-6-fluorochromane-2-carboxylic acid ((*R*)-**S-8**) (135 mg, 688 µmol, 1.00 equiv.) in THF (3.5 mL). The resulting solution was stirred at r.t. for 1 h. Then, a solution of diisobutyl aluminum hydride (DIBAL-H) in CH_2Cl_2 (1.0 M, 147 mg, 1.03 mL, 1.03 mmol, 1.50 equiv.) was added dropwise at −70 °C over a span of 45 min. After completion, the reaction mixture was stirred at −70 °C for another 20 min before MeOH (2.5 mL) was added dropwise over a span of 15 min

whereupon the mixture was poured into water (20 mL) at r.t.. The layers were separated and the aqueous layer was extracted thrice with Et_2O (3 × 15 mL). The combined organic layers were then washed with an aqueous HCl solution (10%, 5 mL), water (5 mL) and a saturated aqueous $NaHCO_3$ solution (5 mL) and dried over Na_2SO_4. The solvents were removed under reduced pressure and the obtained crude product was subjected to FCC (SiO_2, 0 → 10% EtOAc/hexanes) to yield (*R*)-6-fluorochromane-2-carbaldehyde ((*R*)-**S-9**) as a colorless oil (96.0 mg, 533 µmol, 77%).

TLC (20% EtOAc/hexanes): R_f = 0.63 [UV, DNP].

^{1}H-NMR (400 MHz, $CDCl_3$, 300 K): δ [ppm] = 9.80 (s, 1H, COH), 6.90 – 6.82 (m, 2H, H7, H8), 6.76 (dd, $^3J_{HF}$ = 8.8 Hz, $^4J_{HH}$ = 3.0 Hz, 1H, H5), 4.46 (dd, ^3J = 8.8 Hz, ^3J = 3.4 Hz, 1H, H2), 2.87 – 2.72 (m, 2H, H4), 2.21 (*virt.* dtd, ^2J = 13.8 Hz, $^3J \approx {}^3J$ = 5.8 Hz, ^3J = 3.4 Hz, 1H, H3^a), 2.03 (*virt.* dtd, ^2J = 13.8 Hz, $^3J \approx {}^3J$ = 8.9 Hz, ^3J = 5.8 Hz, 1H, H3^b).

^{13}C-NMR (101 MHz, $CDCl_3$, 300 K): δ [ppm] = 201.2 (COH), 157.3 (d, $^1J_{CF}$ = 239 Hz, C6), 149.5 (d, $^4J_{CF}$ = 2.1 Hz, C8a), 122.8 (d, $^3J_{CF}$ = 7.4 Hz, C4a), 118.0 (d, $^3J_{CF}$ = 8.2 Hz, C8), 115.6 (d, $^2J_{CF}$ = 22.6 Hz, C5), 114.7 (d, $^2J_{CF}$ = 23.4 Hz, C7), 79.5 (C2), 23.6 (d, $^4J_{CF}$ = 1.4 Hz, C4), 22.3 (C3).

^{19}F-NMR (376 MHz, $CDCl_3$, 300 K): δ [ppm] = −123.2 – −123.2 (m).

Specific Rotation: $[\alpha]_D^{27}$: −44 (c = 1.0, $CHCl_3$).

The spectroscopic data matches the one for (*S*)-**S-9**.

(*R*)-6-Fluoro-2-((*S*)-oxiran-2-yl)chromane ((2*R*,2'*S*)-10) and (*R*)-6-Fluoro-2-((*R*)-oxiran-2-yl)chromane ((2*R*,2'*R*)-10)

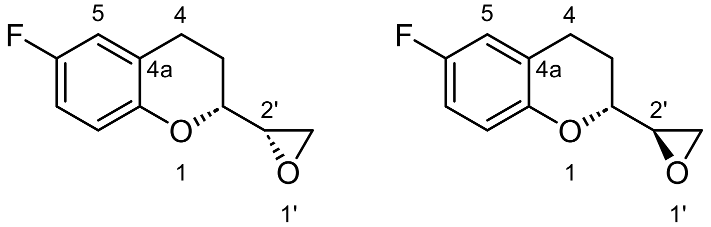

(2*R*,2'*S*)-**S-10** (2*R*,2'*R*)-**S-10**

$C_{11}H_{11}FO_2$
MW = 194.21 $gmol^{-1}$

DMSO (5.0 mL) was added dropwise to a mixture of NaH (60 wt% dispersion in mineral oil, 22.0 mg, 549 µmol, 1.10 equiv.) and trimethylsulfoxonium iodide (132 mg, 599 µmol, 1.20 equiv.) at 0 °C over 15 min. The obtained frozen reaction mixture was then allowed to warm to r.t. and stirred for 1 h. Then, a solution of (*R*)-6-fluorochromane-2-carbaldehyde ((*R*)-**S-9**) (90.0 mg, 500 µmol, 1.00 equiv.) in CH_2Cl_2 (1.7 mL) was added dropwise over at r.t. 20 min and the resulting reaction mixture was stirred for another 1 h. After confirmation of complete conversion of ((*R*)-**S-**

9) by TLC, the reaction was quenched by the addition of ice cooled water (10 mL). CH_2Cl_2 (10 mL) was added and the layers were separated. The aqueous layer was extracted with CH_2Cl_2 for another two times (2 × 15 mL) and the combined organic layers were washed with water twice (2 × 10 mL), aqueous $NaHCO_3$ solution (10%, 5 mL) and brine (10 mL) before being dried over Na_2SO_4. The solvents were removed under reduced pressure below 35 °C. The crude product was subjected to FCC (SiO_2, 0 → 4 → 7% Et_2O/pentane) to yield (*R*)-6-fluoro-2-((*S*)-oxiran-2-yl)chromane (2*R*,2'*S*)-**10** as a white solid (55.0 mg, 283 µmol, 57%) and (*R*)-6-Fluoro-2-((*R*)-oxiran-2-yl)chromane (2*R*,2'*R*)-**10** as a colorless oil (28.0 mg, 144 µmol, 29%). Due to their low stability, (2*R*,2'*S*)-**10** and (2*R*,2'*R*)-**10** were stored at −20 °C.

(2*R*,2'*S*)**-S-10**

TLC (15% EtOAc/hexanes): R_f = 0.65 [UV, KMnO4].

^{1}H-NMR (400 MHz, $CDCl_3$, 300 K): δ [ppm] = 6.82 – 6.74 (m, 3H, H5, H7, H8), 3.38 (ddd, ^3J = 10.0 Hz, ^3J = 5.4 Hz, ^3J = 2.6 Hz, 1H, H2), 3.12 (ddd, ^3J = 5.4 Hz, ^3J = 3.9 Hz, ^3J = 2.6 Hz, 1H, H2'), 2.89 (dd, ^3J = 5.0 Hz, ^3J = 3.9 Hz, 1H, H3'^{a}), 2.85 – 2.79 (m, 3H, H4, H3'^{b}), 2.14 (dddd, ^2J = 13.7 Hz, ^3J = 6.1 Hz, ^3J = 4.0 Hz, ^3J = 2.6 Hz, 1H, H3^a), 1.90 (*virt.* dtd, ^2J = 13.7 Hz, $^3J \approx {}^3J$ = 10.2 Hz, ^3J = 6.3 Hz, 1H, H3^b).

^{13}C-NMR (101 MHz, $CDCl_3$, 300 K): δ [ppm] = 157.0 (d, $^1J_{CF}$ = 238 Hz, C6), 150.2 (d, $^4J_{CF}$ = 2.1 Hz, C8a), 123.0 (d, $^3J_{CF}$ = 7.4 Hz, C4a), 117.7 (d, $^3J_{CF}$ = 8.2 Hz, C8), 115.5 (d, $^2J_{CF}$ = 22.6 Hz, C5), 114.3 (d, $^2J_{CF}$ = 23.3 Hz, C7), 75.8 (C2), 53.1 (C2'), 45.9 (C3'), 24.4 (C3), 24.3 (d, $^4J_{CF}$ = 1.4 Hz, C4).

^{19}F-NMR (376 MHz, $CDCl_3$, 300 K): δ [ppm] = −124.1 – −124.1 (m).

Specific Rotation: $[\alpha]_D^{25}$: −80 (c = 1.0, $CHCl_3$).

The spectroscopic data matches the one obtained for (2*S*, 2'*R*)-**S-10**.

(2*R*,2'*R*)-**10**:

TLC (15% EtOAc/hexanes): R_f = 0.50 [UV, KMnO4].

^{1}H-NMR (400 MHz, $CDCl_3$, 300 K): δ [ppm] = 6.80 – 6.78 (m, 2H, H7, H8), 6.75 – 6.72 (m, 1H, H5), 3.83 (ddd, ^3J = 10.5 Hz, ^3J = 5.6 Hz, ^3J = 2.5 Hz, 1H, H2), 3.19 (ddd, ^3J = 5.6 Hz, ^3J = 4.2 Hz, ^3J = 2.7 Hz, 1H, H2'), 2.89 – 2.76 (m, 4H, H4, H3'), 2.04 (*virt.* ddt ^2J = 13.5 Hz,

^3J = 5.9 Hz, $^3J \approx {}^3J$ = 3.0 Hz, 1H, H3[a]), 1.91 (*virt.* dtd, ^2J = 13.5 Hz, $^3J \approx {}^3J$ = 10.9 Hz, ^3J = 5.9 Hz, 1H, H3[b]).

^{13}C-NMR (101 MHz, $CDCl_3$, 300 K): δ [ppm] = 156.9 (d, $^1J_{CF}$ = 238 Hz, C6), 150.3 (d, $^4J_{CF}$ = 2.0 Hz, C8a), 122.7 (d, $^3J_{CF}$ = 7.5 Hz, C4a), 117.8 (d, $^3J_{CF}$ = 8.1 Hz, C8), 115.3 (d, $^2J_{CF}$ = 22.6 Hz, C5), 114.2 (d, $^2J_{CF}$ = 23.1 Hz, C7), 76.1 (C2), 53.8 (C2'), 43.9 (C3'), 24.5 (d, $^4J_{CF}$ = 1.0 Hz, C4), 24.1 (C3).

^{19}F-NMR (376 MHz, $CDCl_3$, 300 K): δ [ppm] = −124.2 – −124.3 (m).

Specific Rotation: $[\alpha]_D^{26}$: −84 (*c* = 0.5, $CHCl_3$).

The spectroscopic data matches the one reported in the literature.[40]

(*S*)-6-Fluorochromane-2-carboxylic acid ((*S*)-S-8)

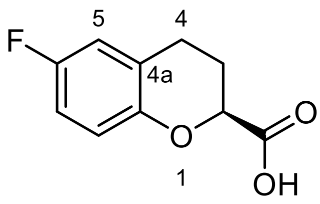

(*S*)-**S-8**
$C_{10}H_9O_3$
MW = 196.18 gmol^{-1}

A solution of (*S*)-6-fluorochromane-2-carboxamide (*ent*-**1b**) (140 mg, 717 µmol, 1.00 equiv.) in aq. HCl (12 M, 784 mg, 1.79 mL, 21.5 mmol, 30.0 equiv.) and HCl in 1,4-dioxane (4.0 M, 523 mg, 3.59 mL, 14.3 mmol, 20.0 equiv.) was heated to 60 °C for 19 h. After cooling to r.t., the mixture was diluted with water (15 mL) and then extracted thrice with EtOAc (3 × 15 mL). The combined organic layers were washed with brine and dried over Na_2SO_4 to afford (*S*)-6-fluorochromane-2-carboxylic acid ((*S*)-**S-8**) as a white solid (140 mg, 714 µmol, 99%).

TLC (3% MeOH/CH_2Cl_2): R_f = 0.22 [UV, $KMnO_4$].

TLC (3% MeOH/CH_2Cl_2): R_f = 0.22 [UV, $KMnO_4$].

^{1}H-NMR (400 MHz, $CDCl_3$, 300 K): δ [ppm] = 6.89 – 6.80 (m, 2H, H7, H8), 6.76 (dd, $^3J_{HF}$ = 8.8 Hz, $^4J_{HH}$ = 2.9 Hz, 1H, H5), 4.74 (dd, ^3J = 7.8 Hz, ^3J = 3.6 Hz, 1H, H2), 2.90 – 2.75 (m, 2H, H4), 2.34 (*virt.* dtd, ^2J = 13.8 Hz, $^3J \approx {}^3J$ = 6.2 Hz, ^3J = 3.6 Hz, 1H, H3[a]), 2.19 (*virt.* dtd, ^2J = 13.8 Hz, $^3J \approx {}^3J$ = 8.0 Hz, ^3J = 5.8 Hz, 1H, H3[b]).

^{13}C-NMR (101 MHz, $CDCl_3$, 300 K): δ [ppm] = 175.7 (COOH), 157.3 (d, $^1J_{CF}$ = 240 Hz, C6), 149.1 (d, $^4J_{CF}$ = 2.3 Hz, C8a), 122.5 (d, $^3J_{CF}$ = 7.5 Hz, C4a), 118.0 (d, $^3J_{CF}$ = 8.1 Hz, C8), 115.5 (d, $^2J_{CF}$ = 22.9 Hz, C5), 114.7 (d, $^2J_{CF}$ = 23.3 Hz, C7), 73.4 (C2), 24.3 (C3), 23.6 (d, $^4J_{CF}$ = 1.5 Hz, C4).

^{19}F-NMR (376 MHz, $CDCl_3$, 300 K): δ [ppm] = −123.0 – −123.1 (m).

Specific Rotation: $[\alpha]_D^{27}$: +16 (*c* = 1.0, DMF). $[\alpha]_D^{27}$: −22 (*c* = 1.0, $CHCl_3$).

The spectroscopic data matches the one of the enantiomer (*R*)-**S-8**.

(*S*)-6-Fluorochromane-2-carbaldehyde ((*S*)-S-9)

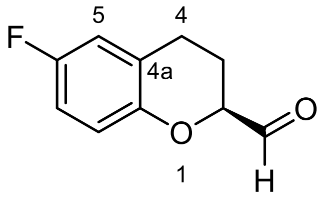

(*S*)-**S-9**
$C_{10}H_9O_2$
MW = 180.18 gmol^{-1}

1,1'-Carbonyldiimidazole (CDI) (94.8 mg, 585 μmol, 0.850 equiv.) was added to a solution of (*R*)-6-fluorochromane-2-carboxylic acid ((*S*)-**S-8**) (135 mg, 688 μmol, 1.00 equiv.) in THF (3.5 mL). The resulting solution was stirred at r.t. for 1 h. Then, a solution of diisobutyl aluminum hydride (DIBAL-H) in CH_2Cl_2 (1.0 M, 147 mg, 1.03 mL, 1.03 mmol, 1.50 equiv.) was added dropwise at −70 °C over a span of 45 min. After completion, the reaction mixture was stirred at −70 °C for another 20 min before MeOH (2.5 mL) was added dropwise over a span of 15 min whereupon the mixture was poured into water (20 mL) at r.t.. The layers were separated and the aqueous layer was extracted thrice with Et_2O (3 × 15 mL). The combined organic layers were then washed with an aqueous HCl solution (10%, 5 mL), water (5 mL) and a saturated aqueous $NaHCO_3$ solution (5 mL) and dried over Na_2SO_4. The solvents were removed under reduced pressure and the obtained crude product was subjected to FCC (SiO_2, 0 → 10% EtOAc/hexanes) to yield (*R*)-6-fluorochromane-2-carbaldehyde ((*S*)-**S-9**) as a colorless oil (98.0 mg, 544 μmol, 79%).

TLC (20% EtOAc/hexanes): R_f = 0.63 [UV, DNP].

^{1}H-NMR (400 MHz, $CDCl_3$, 300 K): δ [ppm] = 9.81 (s, 1H, COH), 6.90 – 6.82 (m, 2H, H7, H8), 6.76 (dd, $^3J_{HF}$ = 8.8 Hz, $^4J_{HH}$ = 3.0 Hz, 1H, H5), 4.46 (dd, ^3J = 8.9 Hz, ^3J = 3.5 Hz, 1H, H2), 2.88 – 2.72 (m, 2H, H4), 2.21 (*virt.* dtd, ^2J = 13.8 Hz, $^3J \approx {}^3J$ = 5.8 Hz, ^3J = 3.5 Hz, 1H, H3^a), 2.07 – 2.00 (m, 1H, H3^b).

Specific Rotation: $[\alpha]_D^{27}$: +43 (*c* = 1.0, $CHCl_3$).

The NMR data matches the one in the literature.[40]

(*S*)-6-Fluoro-2-((*R*)-oxiran-2-yl)chromane ((2*S*,2'*R)*-S-10) and (*S*)-6-Fluoro-2-((*S*)-oxiran-2-yl)chromane ((2*S*,2'*S)*-S-10)

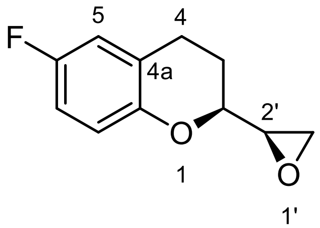

(2*S*,2'*R*)-**S-10**

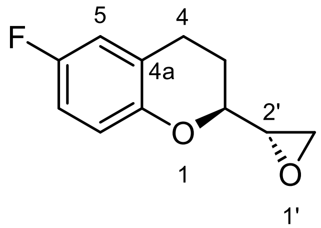

(2*S*,2'*S*)-**S-10**

$C_{11}H_{11}FO_2$

MW = 194.21 gmol^{-1}

DMSO (5.0 mL) was added dropwise to a mixture of NaH (60 wt% dispersion in mineral oil, 22.0 mg, 549 µmol, 1.10 equiv.) and trimethylsulfoxonium iodide (132 mg, 599 µmol, 1.20 equiv.) at 0 °C over 15 min. The obtained frozen reaction mixture was then allowed to warm to r.t. and stirred for 1 h. Then, a solution of (*S*)-6-fluorochromane-2-carbaldehyde ((*S*)-**S-9**) (90.0 mg, 500 µmol, 1.00 equiv.) in CH_2Cl_2 (1.7 mL) was added dropwise over at r.t. 20 min and the resulting reaction mixture was stirred for another 1 h. After confirmation of complete conversion of ((*S*)-**S-9**) by TLC, the reaction was quenched by the addition of ice cooled water (10 mL). CH_2Cl_2 (10 mL) was added and the layers were separated. The aqueous layer was extracted with CH_2Cl_2 for another two times (2 × 15 mL) and the combined organic layers were washed with water twice (2 × 10 mL), aqueous $NaHCO_3$ solution (10%, 5 mL) and brine (10 mL) before being dried over Na_2SO_4. The solvents were removed under reduced pressure at 35 °C. The crude product was subjected to FCC (SiO_2, 0 → 4 → 7% Et_2O/pentane) to yield (*S*)-6-fluoro-2-((*R*)-oxiran-2-yl)chromane (2*S*,2'*R*)-**S-10** as a white solid (57.0 mg, 294 µmol, 59%) and (*S*)-6-Fluoro-2-((*S*)-oxiran-2-yl)chromane (2*S*,2'*S)*-**S-10** as a colorless oil (28.0 mg, 144 µmol, 29%). Due to their low stability, (2*S*,2'*R)*-**S-10** and (2*S*,2'*S)*-**S-10** were stored at −20 °C.

(2*S*,2'*R*)-**S-10**

TLC (15% EtOAc/hexanes): R_f = 0.65 [UV, KMnO4].

^{1}H-NMR (400 MHz, $CDCl_3$, 300 K): δ [ppm] = 6.81 – 6.74 (m, 3H, H5, H7, H8), 3.38 (ddd, ^3J = 10.1 Hz, ^3J = 5.4 Hz, ^3J = 2.5 Hz, 1H, H2), 3.13 (ddd, ^3J = 5.4 Hz, ^3J = 3.9 Hz, ^3J = 2.6 Hz, 1H, H2'), 2.89 (dd, ^3J = 5.1 Hz, ^3J = 3.9 Hz, H3'^{a}), 2.87 – 2.76 (m, 3H, H4, H3'^{b}), 2.14 (dddd, ^2J = 13.7 Hz, ^3J = 6.1 Hz, ^3J = 3.8 Hz, ^3J = 2.5 Hz, 1H, H3^a), 1.89 (*virt.* dtd, ^2J = 13.7 Hz, $^3J \approx {}^3J$ = 10.4 Hz, ^3J = 6.0 Hz, 1H, H3^b).

^{13}C-NMR (101 MHz, $CDCl_3$, 300 K): δ [ppm] = 156.9 (d, $^1J_{CF}$ = 238 Hz, C6), 150.2 (d, $^4J_{CF}$ = 2.0 Hz, C8a), 123.0 (d, $^3J_{CF}$ = 7.5 Hz, C4a), 117.6 (d, $^3J_{CF}$ = 8.2 Hz, C8), 115.5 (d,

$^2J_{CF}$ = 22.6 Hz, C5), 114.2 (d, $^2J_{CF}$ = 23.3 Hz, C7), 75.7 (C2), 53.1 (C2'), 45.9 (C3'), 24.3 (C3), 24.3 (d, $^4J_{CF}$ = 1.2 Hz, C4).

^{19}F-NMR (376 MHz, $CDCl_3$, 300 K): δ [ppm] = −124.1 – −124.2 (m).

Specific Rotation: $[\alpha]_D^{26}$: +78 (*c* = 1.0, $CHCl_3$).

The spectroscopic data matches the one reported in the literature.[104]

(2*S*,2'*S*)-**S-10**:

TLC (15% EtOAc/hexanes): R_f = 0.50 [UV, KMnO4].

^{1}H-NMR (400 MHz, $CDCl_3$, 300 K): *δ* [ppm] = 6.80 – 6.78 (m, 2H, H7, H8), 6.76 – 6.72 (m, 1H, H5), 3.84 (ddd, ^3J = 10.4 Hz, ^3J = 5.5 Hz, ^3J = 2.5 Hz, 1H, H2), 3.19 (ddd, ^3J = 5.5 Hz, ^3J = 4.1 Hz, ^3J = 2.7 Hz, 1H, H2'), 2.91 – 2.76 (m, 4H, H4, H3'), 2.04 (*virt.* ddt ^2J = 13.6 Hz, ^3J = 6.1 Hz, $^3J \approx {}^3J$ = 3.1 Hz, 1H, H3^a), 1.92 (*virt.* dtd, ^2J = 13.6 Hz, $^3J \approx {}^3J$ = 10.7 Hz, ^3J = 6.0 Hz, 1H, H3^b).

^{13}C-NMR (101 MHz, $CDCl_3$, 300 K): *δ* [ppm] = 157.0 (d, $^1J_{CF}$ = 238 Hz, C6), 150.4 (d, $^4J_{CF}$ = 2.0 Hz, C8a), 122.7 (d, $^3J_{CF}$ = 7.5 Hz, C4a), 117.9 (d, $^3J_{CF}$ = 8.2 Hz, C8), 115.3 (d, $^2J_{CF}$ = 22.6 Hz, C5), 114.2 (d, $^2J_{CF}$ = 23.3 Hz, C7), 76.1 (C2), 53.8 (C2'), 43.9 (C3'), 24.6 (d, $^4J_{CF}$ = 1.3 Hz, C4), 24.2 (C3).

^{19}F-NMR (376 MHz, $CDCl_3$, 300 K): δ [ppm] = −124.2 – −124.3 (m).

Specific Rotation: $[\alpha]_D^{26}$: +78 (*c* = 1.0, $CHCl_3$).

The spectroscopic data matches the one for (2*R*,2'*R*)-**10**.

(*R*)-2-(Benzylamino)-2-((*S*)-6-fluorochroman-2-yl)ethan-1-ol (9)

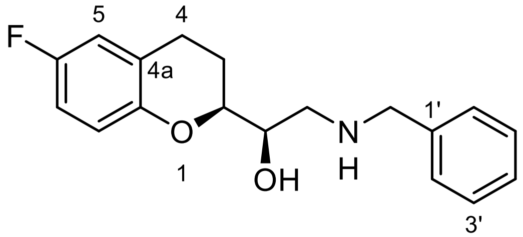

9
$C_{18}H_{20}FNO_2$
MW = 301.36 gmol^{-1}

A solution of (*S*)-6-Fluoro-2-((*R*)-oxiran-2-yl)chromane ((2*S*,2'*R*)-**S-10**) (50.0 mg, 257 µmol, 1.00 equiv) in ^{i}PrOH (750 µL) was added dropwise over a span of 60 min to a solution of benzylamine (221 mg, 2.06 mmol, 8.00 equiv.) in ^{i}PrOH (750 µL) at 80 °C. The resulting solution was then stirred at this temperature for another 5.5 h. Upon confirmation of full conversion of the starting material by TLC, the mixture was allowed to cool to r.t. and the volatile compounds were removed under reduced pressure to obtain a light yellow oil. Hexanes

(2.5 mL) were added dropwise which resulted in the precipitation of a white solid. After being a allowed to sit for 1 h, the mother liquor was decanted and the white solids were washed thoroughly with hexanes thrice (3 × 5 mL) and then dried under reduced pressure at 50 °C. (*R*)-2-(benzylamino)-1-((*S*)-6-fluorochroman-2-yl)ethan-1-ol (**9**) (68.0 mg, 257 µmol, 88%) was obtained as a white solid.

TLC (10% MeOH/CH_2Cl_2): $R_f = 0.2$ [UV, KMnO4].
^{1}H-NMR (400 MHz, $CDCl_3$, 300 K): δ [ppm] = 7.36 – 7.25 (m, 5H, H2', H3', H4'), 6.80 – 6.69 (m, 3H, H5, H7, H8), 3.90 – 3.84 (m, 3H, H2, CH_2C1'), 3.76 (ddd, $^3J = 8.0$ Hz, $^3J = 6.2$ Hz, $^3J = 3.6$ Hz, 1H, C*H*OH), 2.89 (dd, $^2J = 12.3$ Hz, $^3J = 3.6$ Hz, COHCH_2^a), 2.86 – 2.72 (m, 3H, H4, COHCH_2^b), 2.27 (bs, 1H, NH/OH), 2.14 (*virt.* ddt, $^2J = 13.6$ Hz, $^3J = 5.8$ Hz, $^3J \approx {}^3J = 2.9$ Hz, 1H, H3^a),1.82 (*virt.* dtd, $^2J = 13.6$ Hz, $^3J \approx {}^3J = 10.8$ Hz, $^3J = 6.0$ Hz, 1H, H3^b).
^{13}C-NMR (101 MHz, $CDCl_3$, 300 K): δ [ppm] = 156.9 (d, $^1J_{CF} = 238$ Hz, C6), 150.6 (d, $^4J_{CF} = 2.1$ Hz, C8a), 140.1 (C1'), 128.7 (C2'/C3'), 128.3 (C2'/C3'), 127.3 (C4'), 123.5 (d, $^3J_{CF} = 7.4$ Hz, C4a), 117.6 (d, $^3J_{CF} = 8.1$ Hz, C8), 115.5 (d, $^2J_{CF} = 22.4$ Hz, C5), 114.0 (d, $^2J_{CF} = 23.1$ Hz, C7), 77.8 (C2), 70.9 (COH), 54.0 (CH$_2$C1'), 50.7 (COHCH$_2$), 24.7 (d, $^4J_{CF} = 1.3$ Hz, C4), 23.3 (C3).
^{19}F-NMR (376 MHz, $CDCl_3$, 300 K): δ [ppm] = −124.4 – −124.4 (m).
Specific Rotation: $[\alpha]_D^{27}$: +82 (*c* = 1.0, $CHCl_3$).
Chiral HPLC: 99% *ee* (AD-H 250 × 4.6 mm, *n*-heptane/^{i}PrOH = 90/10, 1 ml/min, λ = 210 nm); t_R = 12.54 min (minor, *ent*-**9**), 16.11 min (major, **9**).
The spectroscopic data matches the one reported in the literature.[105]

(*R*)-2-(Benzyl((*R*)-2-((*R*)-6-fluorochroman-2-yl)-2-hydroxyethyl)amino)-1-((*S*)-6-fluorochroman-2-yl)ethan-1-ol (S-11)

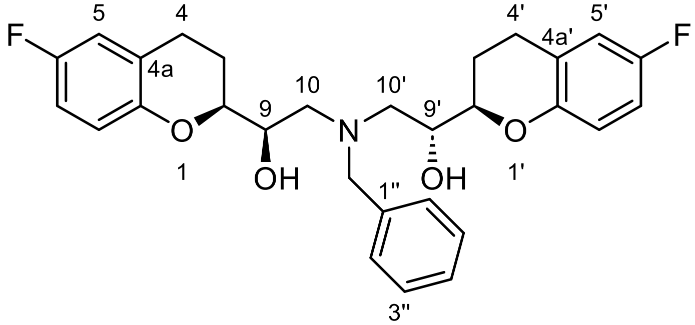

S-11

$C_{29}H_{31}F_2NO_4$

MW = 495.57 gmol^{-1}

A solution of (*R*)-2-(benzylamino)-1-((*S*)-6-fluorochroman-2-yl)ethan-1-ol (**9**) (30.5 mg, 101 µmol, 1.00 equiv.) and (*R*)-6-fluoro-2-((*R*)-oxiran-2-yl)chromane (**10**) (21.6 mg, 111 µmol, 1.10 equiv.) in ^{i}PrOH (500 µL) was heated to reflux for 6 h. After being allowed to cool to r.t., the volatile compounds were removed under reduced pressure and the obtained crude product was subjected to FCC (SiO_2, 0 → 2% MeOH/CH_2Cl_2) to yield (*R*)-2-(benzyl((*R*)-2-((*R*)-6-fluorochroman-2-yl)-2-hydroxyethyl)amino)-1-((*S*)-6-fluorochroman-2-yl)ethan-1-ol (**S-11**) as a colorless oil (45.0 mg, 90.8 µmol, 90%).

TLC (2% MeOH/CH_2Cl_2): R_f = 0.75 [UV, KMnO4].

^{1}H-NMR (400 MHz, $CDCl_3$, 300 K): δ [ppm] = 7.36 – 7.25 (m, 5H, H2', H3', H4'), 6.79 – 6.68 (m, 6H, H5, H7, H8, H5', H7', H8'), 3.93 (d, ^2J = 13.6 Hz, 1H, C1"CH_2^a), 3.89 – 3.80 (m, 4H, H2, H2', H9, H9'), 3.68 (d, ^2J = 13.6 Hz, 1H, C1"CH_2^b), 3.15 (bs, 2H, OH), 2.97 (dd, ^2J = 13.2 Hz, ^3J = 2.3 Hz, H10^a), 2.90 (dd, ^2J = 13.5 Hz, ^3J = 9.0 Hz, H10'^{a}), 2.84 – 2.66 (m, 6H, H4, H4', H10^b, H10'^{b}), 2.12 (*virt.* dtd, ^2J = 13.3 Hz, $^3J \approx {}^3J$ = 4.5 Hz, ^3J = 2.1 Hz, 1H, H3^a), 1.91 – 1.76 (m, 3H, H3^b, H3'^{a}, H3'^{b}).

^{13}C-NMR (101 MHz, $CDCl_3$, 300 K): δ [ppm] = 157.3 (d, $^1J_{CF}$ = 238 Hz, C6/C6'), 156.9 (d, $^1J_{CF}$ = 238 Hz, C6/C6'), 150.6 (d, $^4J_{CF}$ = 2.1 Hz, C8a/C8a'), 150.5 (d, $^4J_{CF}$ = 2.1 Hz, C8a/C8a'), 138.4 (C1"), 129.3 (C2"/C3"), 128.7 (C2"/C3"), 127.6 (C4"), 123.5 (d, $^3J_{CF}$ = 7.3 Hz, C4a/C4a'), 123.2 (d, $^3J_{CF}$ = 7.4 Hz, C4a/C4a'), 117.7 (d, $^3J_{CF}$ = 8.0 Hz, C8/C8'), 117.5 (d, $^3J_{CF}$ = 8.0 Hz, C8/C8'), 115.5 (d, $^2J_{CF}$ = 22.7 Hz, C5/C5'), 115.4 (d, $^2J_{CF}$ = 22.5 Hz, C5/C5'), 114.0 (d, $^2J_{CF}$ = 23.4 Hz, C7/C7'), 113.9 (d, $^2J_{CF}$ = 23.1 Hz, C7/C7'), 77.9 (C2), 77.1 (C2'), 70.9 (C9'), 69.6 (C9), 60.2 (C1"CH_2), 57.4 (C10), 56.6 (C10'), 24.9 (d, $^4J_{CF}$ = 1.2 Hz, C4'), 24.6 (d, $^4J_{CF}$ = 0.8 Hz, C4), 23.6 (C3'), 23.3 (C3).

^{19}F-NMR (376 MHz, $CDCl_3$, 300 K): δ [ppm] = −124.0 – −124.1 (m), −124.4 – −124.4 (m).

Specific Rotation: $[a]_D^{25}$: +50 (*c* = 1.0, $CHCl_3$).

The spectroscopic data matches the one reported in the literature.[40]

(*R*)-2-((*R*)-6-Fluorochroman-2-yl)-*N*-((*R*)-2-((*S*)-6-fluorochroman-2-yl)-2-hydroxyethyl)-2-hydroxyethan-1-ammonium chloride ((*S*, *R*, *R*, *R*)Nebivolol · *HCl*)

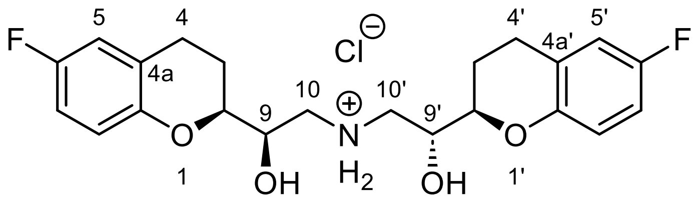

(*S*,*R*,*R*,*R*)-**Nebivolol**·*HCl*
$C_{22}H_{26}ClF_2NO_4$
MW = 441.90 gmol^{-1}

Following a modified procedure by Carreño *et al.,*[40] a solution of (*R*)-2-(benzyl((*R*)-2-((*R*)-6-fluorochroman-2-yl)-2-hydroxyethyl)amino)-1-((*S*)-6-fluorochroman-2-yl)ethan-1-ol (**S-11**) (30.0 mg, 60.5 µmol, 1.00 equiv.) and Pd/C (10 wt%, 32.2 mg, 30.3 µmol, 50 mol%) in EtOH (1.0 mL) was degassed by being sparged with argon for 15 min and then sparged continuously sparged with H_2 (1 atm) and stirred at r.t. for 16 h. The mixture was filtered through celite and the celite was rinsed with EtOH (10 mL). The filtrate was dried under reduced pressure and the obtained oiled was dissolved in dry MeOH (200 µL). A solution of HCl in MeOH (3 M, 3 mL) was added and the resulting solution was stirred at r.t. for 30 min and dried under reduced pressure. The obtained yellow solids were thoroughly washed with CH_2Cl_2 (3 × 2 mL) to yield (*R*)-2-((*R*)-6-fluorochroman-2-yl)-*N*-((*R*)-2-((*S*)-6-fluorochroman-2-yl)-2-hydroxyethyl)-2-hydroxyethan-1-ammonium chloride ((*S*, *R*, *R*, *R*)Nebivolol · HCl) as a white solid (21.0 mg, 30.3 µmol, 79%).

^{1}H-NMR (500 MHz, CD_3OD, 300 K): δ [ppm] = 6.84 – 6.76 (m, 6H, H5, H7, H8, H5', H7', H8'), 4.11 (*virt.* dt, ^3J = 9.3 Hz, $^3J \approx {}^3J$ = 3.3 Hz, 1H, H9'), 4.04 – 3.99 (m, 2H, H2', H9), 3.92(ddd, ^3J = 10.4 Hz, ^3J = 6.9 Hz, ^3J =2.1 Hz, 1H, H2), 3.53 (dd, ^2J = 12.8 Hz, ^3J =3.3 Hz, 1H, H10^a), 3.41 (dd, ^2J = 12.7 Hz, ^3J =9.3 Hz, 1H, H10'^{a}), 3.37 – 3.35 (m, 1H, H10'^{b}), 3.25 (dd, ^2J = 12.8 Hz, ^3J =9.3 Hz, 1H, H10^b), 2.96 – 2.79 (m, 4H, H4, H4'), 2.25 (*virt.* ddt, ^2J = 13.5 Hz, ^3J = 5.7 Hz, $^3J \approx {}^3J$ = 2.7 Hz, 1H, H3^a), 2.02 (*virt.* ddt, ^2J = 13.7 Hz, $^3J \approx {}^3J$ = 6.2 Hz, ^3J = 2.5 Hz, 1H, H3'^{a}), 1.93 (dddd, ^2J = 13.7 Hz, ^3J = 12.2 Hz, ^3J = 11.0 Hz, ^3J = 5.5 Hz, 1H, 3'^{b}), 1.79 (*virt.* dtd, ^2J = 13.5 Hz, $^3J \approx {}^3J$ = 10.9 Hz, ^3J = 5.9 Hz, 1H, H3^b).

^{13}C-NMR (126 MHz, CD_3OD, 300 K): δ [ppm] = 158.4 (d, $^1J_{CF}$ = 237 Hz, C6/C6'), 158.4 (d, $^1J_{CF}$ = 237 Hz, C6/C6'), 151.8 (d, $^4J_{CF}$ = 2.1 Hz, C8a/C8a'), 151.4 (d, $^4J_{CF}$ = 2.2 Hz, C8a/C8a'), 124.8 (d, $^3J_{CF}$ = 7.6 Hz, C4a/C4a'), 124.7 (d, $^3J_{CF}$ = 7.4 Hz, C4a/C4a'), 118.7 (d, $^3J_{CF}$ = 7.5 Hz,

C8/C8'), 118.7 (d, $^{3}J_{CF}$ = 7.5 Hz, C8/C8'), 116.3 (d, $^{2}J_{CF}$ = 22.9 Hz, C5/C5'), 116.2 (d, $^{2}J_{CF}$ = 22.6 Hz, C5/C5'), 114.8 (d, $^{2}J_{CF}$ = 23.6 Hz, C7/C7'), 114.8 (d, $^{2}J_{CF}$ = 23.5 Hz, C7/C7'), 79.0 (C2), 78.7 (C2'), 69.4 (C9), 69.2 (C9'), 51.4 (C10), 51.2 (C10'), 25.8 (d, $^{4}J_{CF}$ = 1.2 Hz, C4), 25.2 (d, $^{4}J_{CF}$ = 1.0 Hz, C4'), 24.5 (C3), 24.1 (C3').

^{19}F-NMR (376 MHz, CD_3OD, 300 K): δ [ppm] = −125.9 – −126.0 (m), −126.1 – −126.1 (m).

HRMS (ESI) *m/z* $[M{-}Cl]^+$: calculated for $[C_{22}H_{26}F_2NO_4]^+$: 406.1824; found: 406.1805.

Specific Rotation: $[\alpha]_D^{27}$: +25 (*c* = 0.16, MeOH).

The spectroscopic data matches the one reported in the literature.[40]

Synthesis of Fidarestat

(*S*)-6-Fluoro-4-oxochromane-2-carboxamide (11)

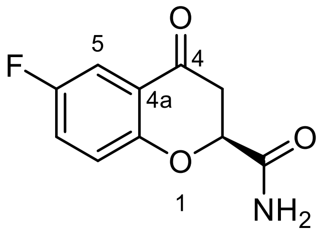

11

$C_{10}H_8FNO_3$

MW = 209.18 gmol^{-1}

Following a modified procedure by Shukla *et al.,* [106] $KMnO_4$ (267 mg, 1.69 mmol, 5.50 equiv.) was added portionwise of the course of 1 h to a solution of (*S*)-6-fluorochromane-2-carboxylic acid (*ent*-**1b**) (60.0 mg, 307 µmol, 1.00 equiv.) and $MgSO_4$ (88.8 mg, 738 µmol, 2.40 equiv.) in water (1.5 mL) and acetone (3.1 mL). The resulting purple mixture was allowed to warm to r.t. and stirred at r.t. for 14 h. Then, an aq. $Na_2S_2O_3$ solution (10%, 10 mL) was added and the brown suspension was filtered over celite and washed with copious amounts of EtOAc. The layers of the filtrate were separated and the aqueous layer was extracted thrice with EtOAc (3 × 10 mL). The combined organic layers were washed with brine (20 mL) and dried over Na_2SO_4. The solvents were removed under reduced pressure and the crude product was subjected to FCC (SiO_2, 50 → 100% EtOAc/hexanes) to yield (*S*)-6-fluoro-4-oxochromane-2-carboxamide (**11**) as a white solid (45.0 mg, 215 µmol, 70%, 99% *ee*).

TLC (60% EtOAc/pentane): $R_f = 0.14$ [UV, $KMnO_4$].

M.p.: 215 °C

^{1}H-NMR (400 MHz, DMSO-d$_6$, 300 K): δ [ppm] = 7.69 (bs, 1H, NH_2^a), 7.51 – 7.46 (m, 2H, H7, NH_2^b), 7.42 (dd, $^2J_{HF}$ = 8.4 Hz, $^4J_{HH}$ = 3.2 Hz, 1H, H5), 7.17 (dd, $^3J_{HH}$ = 9.1 Hz, $^4J_{HF}$ = 4.3 Hz, 1H, H8), 5.09 (dd, ^3J = 7.5 Hz, ^3J = 6.3 Hz, 1H, H2), 2.98 – 2.96 (m, 2H, H3).

^{13}C-NMR (101 MHz, DMSO-d$_6$, 300 K): δ [ppm] = 189.7 (d, $^4J_{CF}$ = 1.9 Hz, C4), 169.9 ($CONH_2$), 156.6 (d, $^1J_{CF}$ = 240 Hz, C6), 156.2 (d, $^4J_{CF}$ = 1.7 Hz, C8a), 123.5 (d, $^2J_{CF}$ = 24.5 Hz, C7), 121.2 (d, $^3J_{CF}$ = 6.4 Hz, C4a), 120.2 (d, $^3J_{CF}$ = 7.5 Hz, C8), 110.8 (d, $^2J_{CF}$ = 23.3 Hz, C5), 75.9 (C2) 38.6 (C3).

Due to an overlap with the signal of the solvent DMSO-d$_6$, the ^{13}C-NMR signal for C3 could not be fully analyzed.

^{19}F-NMR (376 MHz, DMSO-d$_6$, 300 K): δ [ppm] = −121.4 – −121.4 (m).

HRMS (ESI) *m/z* $[M+H]^+$ calculated for $[C_{10}H_9FNO_3]^+$: 210.0561; found: 210.0562.

IR (film) $\tilde{\nu}_{max}$/cm^{-1} = 3397 (m, NH), 3203 (m, NH), 3074 (w, CH_{arom}), 2917 (w, CH_{aliph}), 1694 (s, $C{=}O_{amide}$), 1668 (s, $C{=}O_{ketone}$),1619 (m, C=C), 1481 (s, CH_{arom}), 1431 (s, CH_{aliph}), 1236 (s, C–O), 1162 (m, C–N), 1115 (m, C–O), 1008 (m, C–O).

Specific Rotation: $[\alpha]_D^{26}$: +16 (c = 0.5, MeOH) [99% *ee*].

Chiral HPLC: 99% *ee* (IC 250 × 4.6 mm, *n*-Hep/*iso*-PrOH = 50/50, 1 ml/min, λ = 210 nm); t_R = 9.99 min (major, **11**), 12.89 min (minor, *ent*-**11**).

(2*S*,4*S*)-6-Fluoro-2',5'-dioxospiro[chromane-4,4'-imidazolidine]-2-carboxamide (Fidarestat)

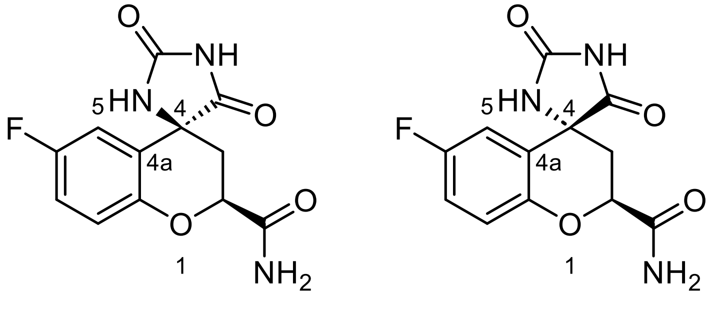

$C_{12}H_{10}FN_3O_4$
MW = 279.07 gmol^{-1}
d.r. = 63/37

Following a modified procedure by Yamaguchi *et al.*,[16] a mixture of (*S*)-6-fluoro-4-oxochromane-2-carboxamide (**11**) (25.0 mg, 120 µmol, 1.00 equiv.), KCN (15.6 mg, 239 µmol, 2.00 equiv.), and $(NH_4)_2CO_3$ (68.9 mg, 717 µmol, 6.00 equiv.) in H_2O (1.2 mL) was stirred in a 20 mL pressure tube (max. 150 psig at 120 °C) at 70 °C for 23 h and then at 90 °C for 1 h. The brown mixture was then acidified with aqueous HCl (6 N) until pH = 1 was reached and then extracted thrice with EtOAc (3 × 5 mL). The combined organic layers were washed with brine (5 mL) and dried over Na_2SO_4 and the solvents were removed under reduced pressure. The crude product was taken up in a minimal amount of EtOAc before pentane was added to precipitate the crude product as a brown solid which was subjected to (SiO_2, 0 → 1% MeOH/EtOAc) to yield (2*S*,4*S*)-6-fluoro-2',5'-dioxospiro[chromane-4,4'-imidazolidine]-2-carboxamide (2*S*,4*S*-**Fidarestat**) as the major diastereomer and (2*S*,4*R*)-6-fluoro-2',5'-dioxospiro[chromane-4,4'-imidazolidine]-2-carboxamide (2*S*,4*R*-**Fidarestat**) as the minor diastereomer in a diastereomeric ratio of 63/37 as a white solid (23.3 mg, 83.4 µmol, 70%).

TLC (1% MeOH/EtOAc): R_f = 0.29 [UV, $KMnO_4$].

M.p.: > 230 °C

^{1}H-NMR (500 MHz, DMSO-d$_6$, 300 K): δ [ppm] = 11.00 (bs, 2H, $CONHCO_{major}$, $CONHCO_{minor}$), 8.89 (s, 1H, $C4NH_{minor}$), 8.40 (s, 1H, $C4NH_{major}$), 7.75 – 7.72 (m, 2H, $NH_2^{a}{}_{major}$, $NH_2^{a}{}_{minor}$), 7.58 (bs, 1H, $NH_2^{b}{}_{minor}$), 7.51 (bs, 1H, $NH_2^{b}{}_{major}$), 7.21 – 7.14 (m, 2H, $H7_{major}$, $H7_{minor}$), 7.03 – 6.97 (m, 3H, $H5_{major}$, $H8_{major}$, $H8_{minor}$), 6.89 (dd, $^3J_{HF}$ = 8.9 Hz, $^4J_{HH}$ = 3.1 Hz, 1H, $H5_{minor}$), 5.03 (dd, ^3J = 12.4 Hz, ^3J = 2.4 Hz, 1H, $H2_{major}$), 4.46 (dd, ^3J = 12.8 Hz, ^3J = 2.1 Hz, 1H, $H2_{minor}$), 2.45 (dd,

^{2}J = 13.9 Hz, ^{3}J = 2.4 Hz, 1H, H3$^{a}_{major}$), 2.37 (dd, ^{2}J = 13.9 Hz, ^{3}J = 2.1 Hz, 1H, H3$^{a}_{minor}$), 2.17 (dd, ^{2}J = 13.9 Hz, ^{3}J = 12.8 Hz, 1H, H3$^{b}_{minor}$), 2.03 (dd, ^{2}J = 13.9 Hz, ^{3}J = 12.4 Hz, 1H, H3$^{b}_{major}$).

^{13}C-NMR (126 MHz, DMSO-d$_6$, 300 K): δ [ppm] = 176.3 (C4*C*O$_{major}$), 176.0 (C4*C*O$_{minor}$), 170.6 (CONH$_{2,minor}$), 170.4 (CONH$_{2,major}$), 156.7 (d, $^{1}J_{CF}$ = 238 Hz, C6$_{minor}$), 156.5 (d, $^{1}J_{CF}$ = 238 Hz, C6$_{major}$), 156.5 (NHCONH$_{major}$), 155.8 (NHCONH$_{minor}$), 150.3 (d, $^{5}J_{CF}$ = 1.6 Hz, C8a$_{major}$), 149.9 (d, $^{5}J_{CF}$ = 1.4 Hz, C8a$_{minor}$), 121.4 – 121.3 (m, C4a$_{major}$, C4a$_{minor}$), 119.1 (d, $^{3}J_{CF}$ = 8.0 Hz, C8$_{minor}$), 119.0 (d, $^{3}J_{CF}$ = 7.9 Hz, C8$_{major}$), 117.7 (d, $^{2}J_{CF}$ = 23.3 Hz, C7$_{minor}$), 117.2 (d, $^{2}J_{CF}$ = 23.4 Hz, C7$_{major}$), 113.4 (d, $^{2}J_{CF}$ = 23.4 Hz, C5$_{minor}$), 112.1 (d, $^{2}J_{CF}$ = 24.1 Hz, C5$_{major}$), 72.2 (C2$_{major}$), 71.8 (C2$_{minor}$), 60.3 (d, $^{5}J_{CF}$ = 1.0 Hz, C4$_{minor}$), 59.4 (d, $^{5}J_{CF}$ = 0.7 Hz, C4$_{minor}$), 35.7 (C3$_{minor}$), 32.7 (C3$_{major}$).

^{19}F-NMR (471 MHz, CDCl$_3$, 300 K): δ [ppm] = −121.5 – −121.6 (m, *minor*), −122.0 – −122.1 (m, *major*).

HRMS (ESI) *m/z* [M+H]$^{+}$: calculated for [$C_{12}H_{11}FN_3O_4$]$^{+}$: 280.0728; found: 280.0722.

IR (film) $\tilde{\nu}_{max}$/cm^{-1} = 3468 (w, NH), 3197 (m, NH), 3075 (w, CH$_{arom}$), 2956 (w, CH$_{aliph}$), 2924 (w, CH$_{aliph}$), 2854 (w, CH$_{aliph}$), 1717 (s, C=O), 1674 (s, C=O), 1594 (w, C=C), 1490 (s, CH$_{arom}$), 1429 (m, CH$_{aliph}$), 1397 (m, C–O)1357 (m, C–N), 1112 (s, C–O).

The recorded spectroscopic data match the reported values.[16]

S13. NMR Spectra

Chromane-2-carboxylic acid ethyl ester (*rac*-S-2a)

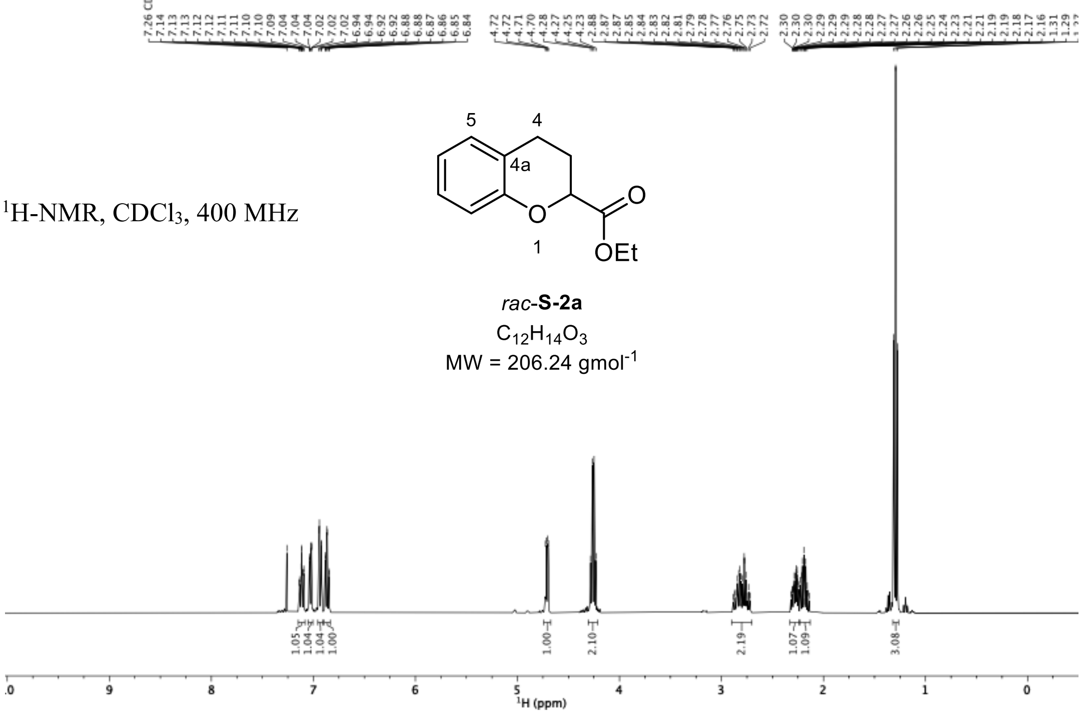

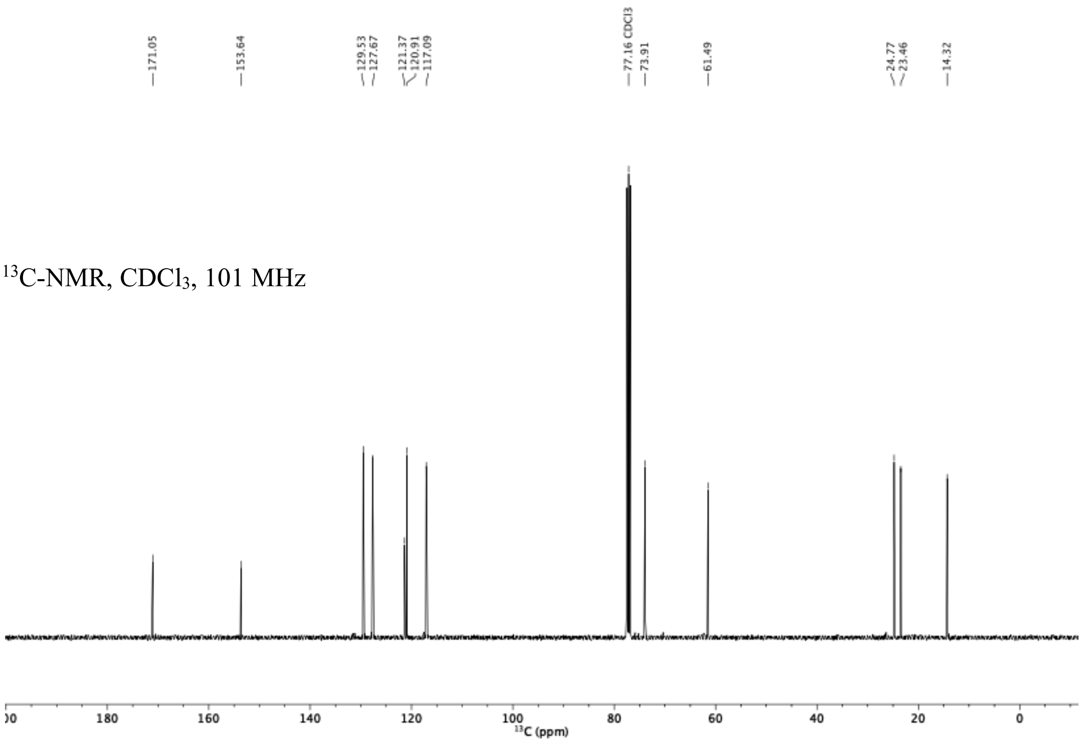

Chromane-2-carboxamide (*rac*-**1a-*d*₁**)

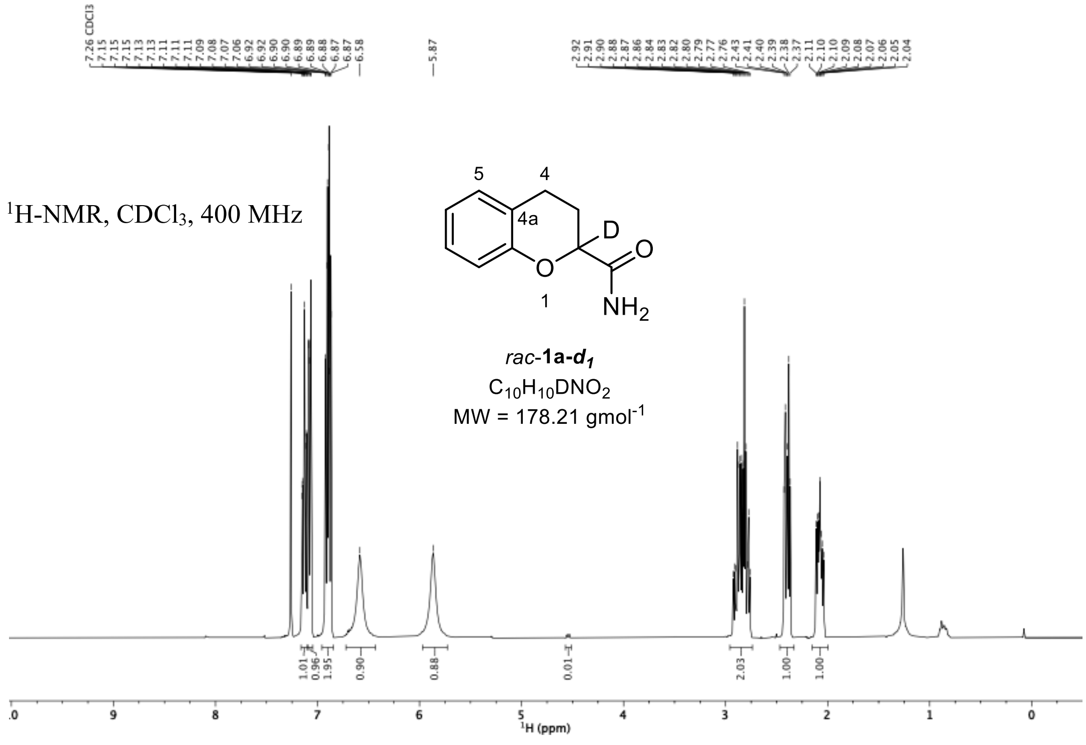

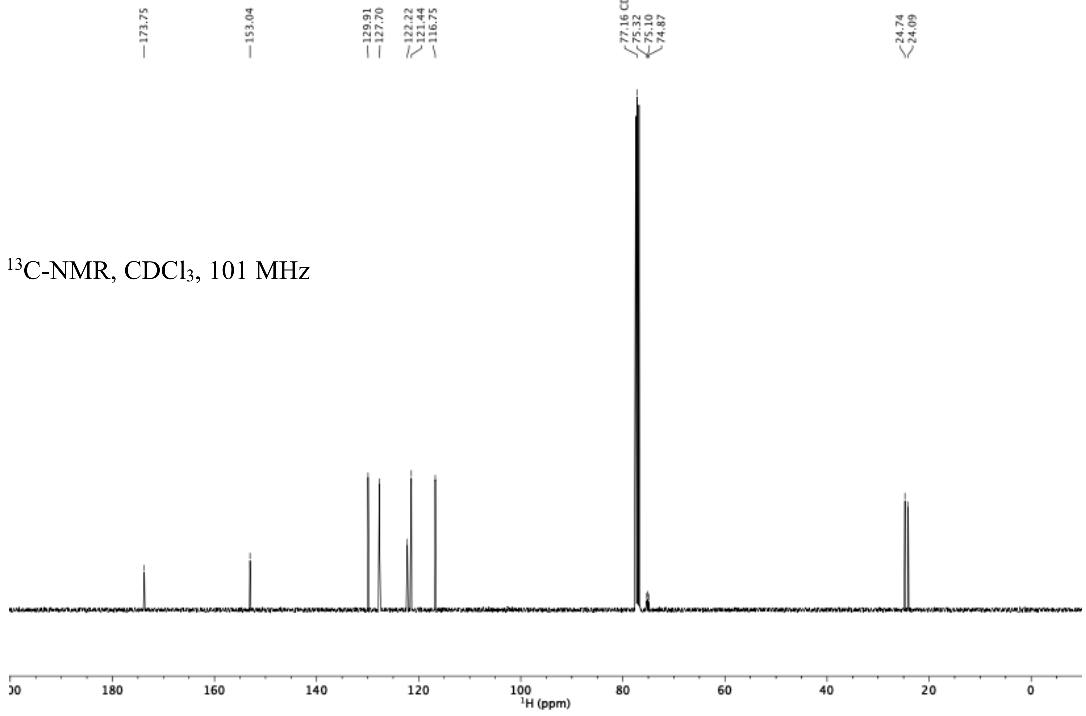

Thiophenol-*d*$_1$ (PhSD)

^{1}H-NMR, $CDCl_3$, 500 MHz

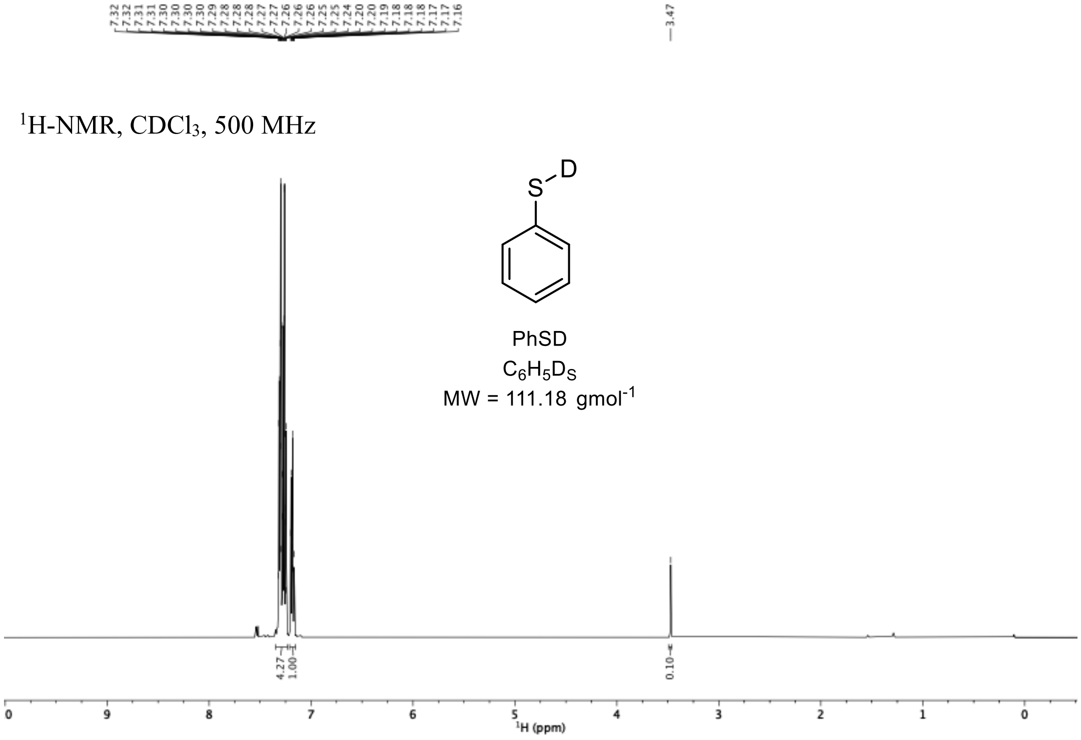

6-Ethyl-4-oxo-4*H*-chromene-2-carboxylic acid ethyl ester (S-1i)

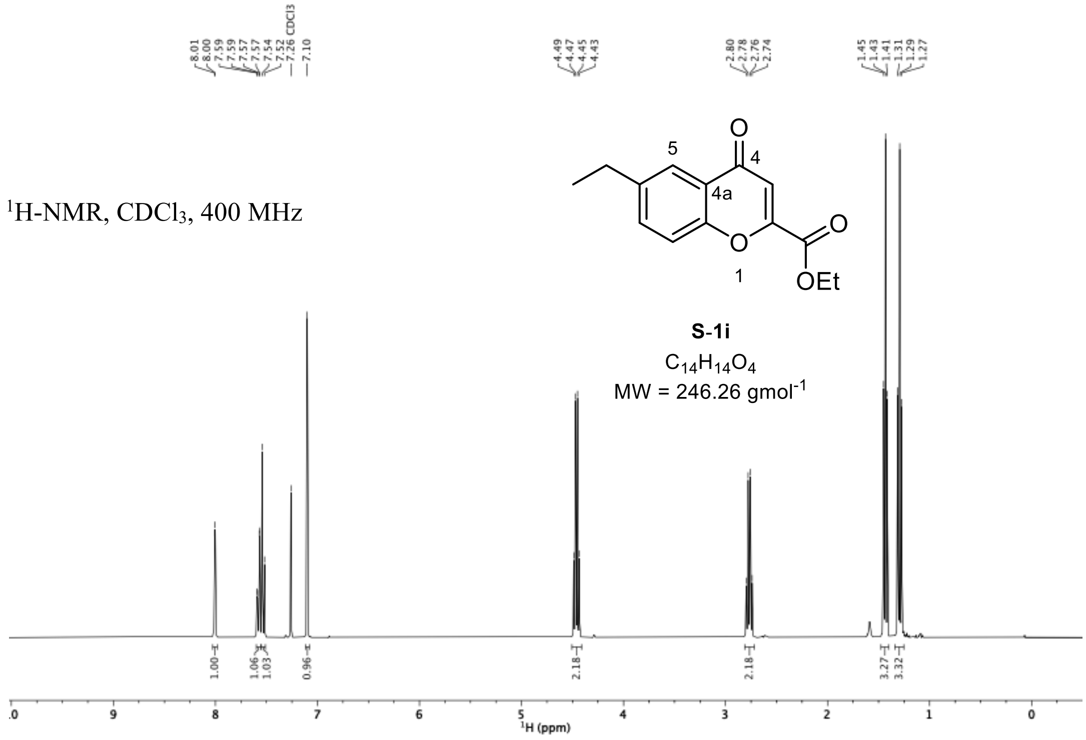

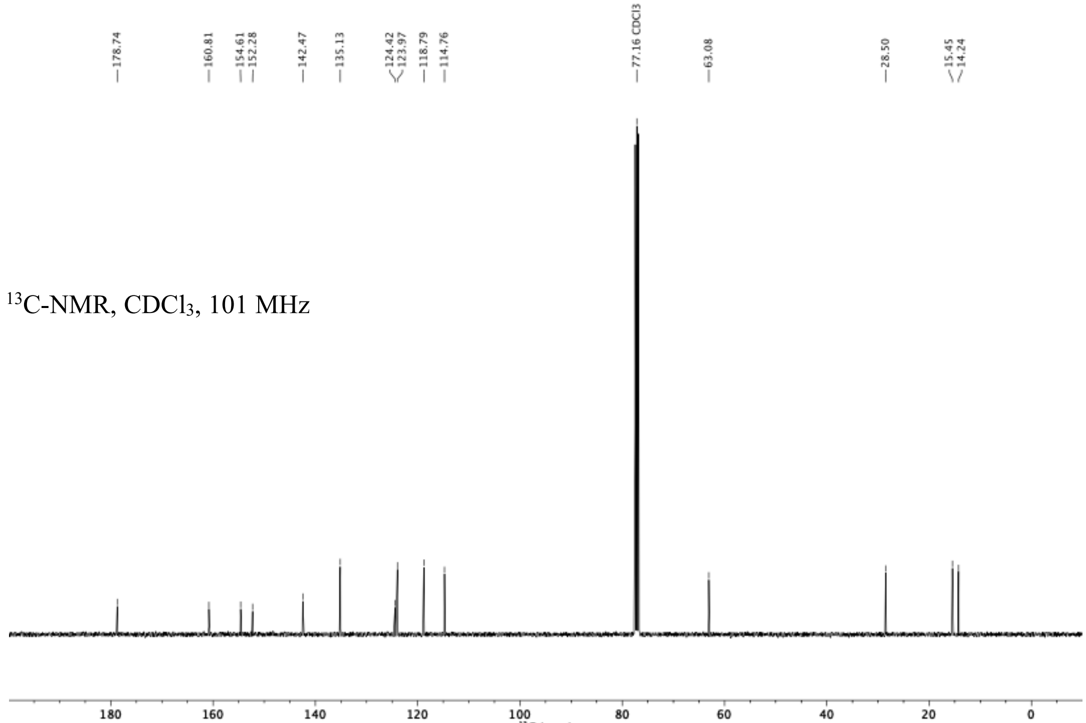

5-Methyl-4-oxo-4*H*-chromene-2-carboxylic acid ethyl ester (S-1j)

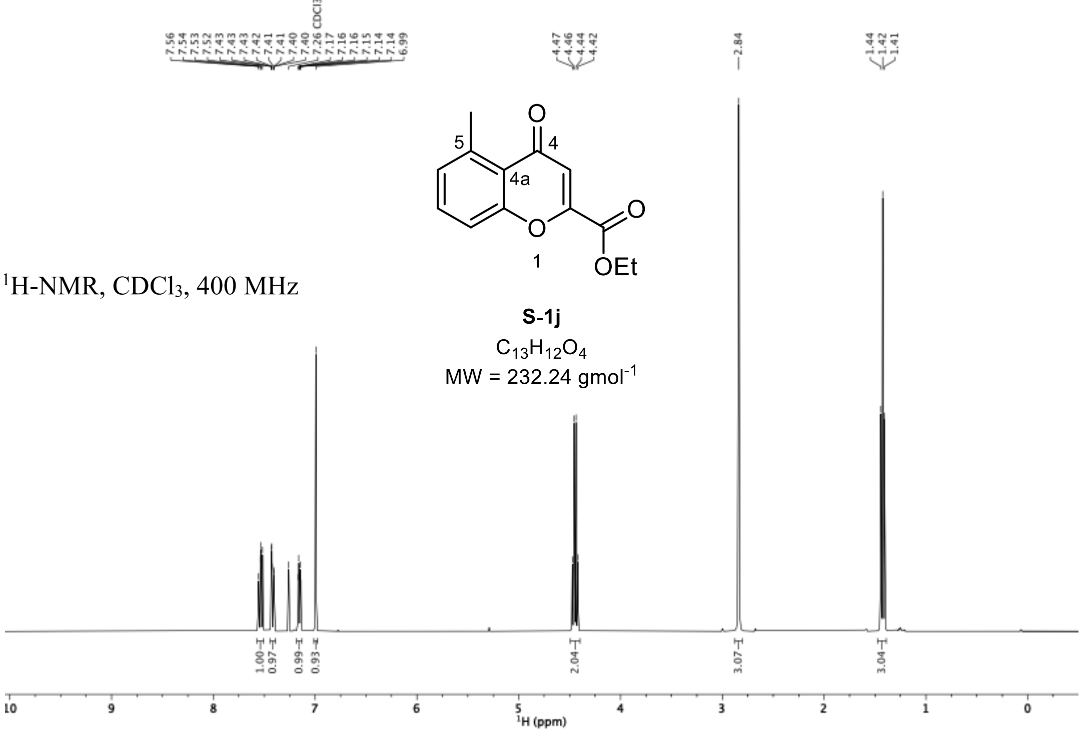

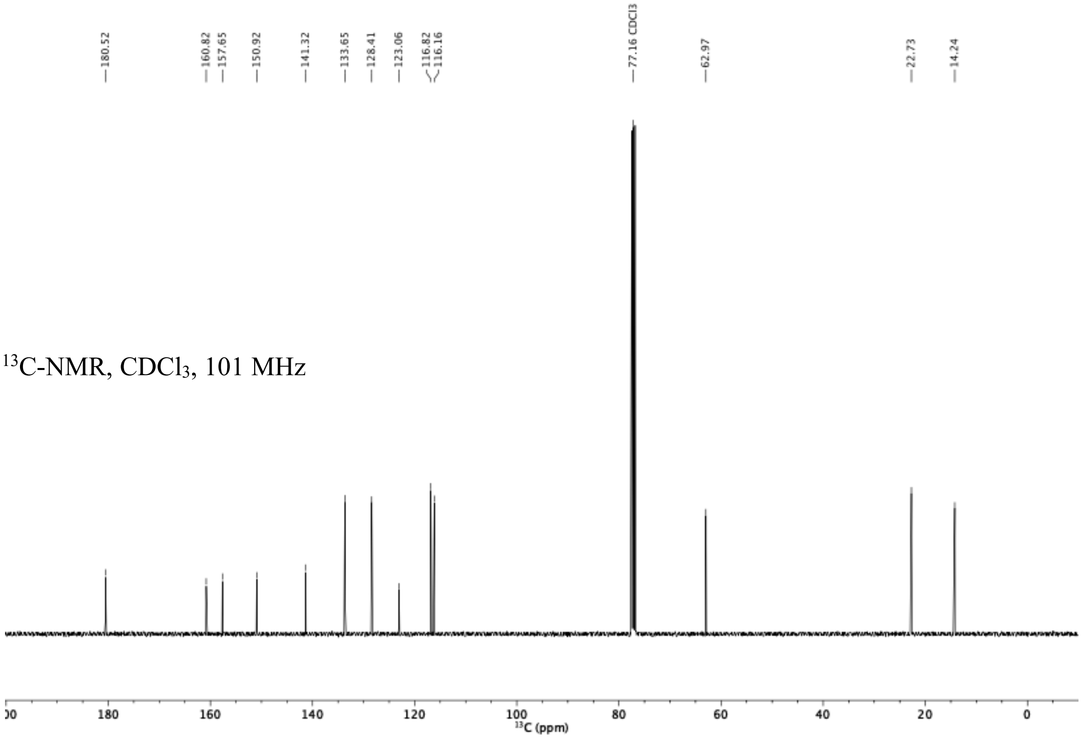

7-Methyl-4-oxo-4*H*-chromene-2-carboxylic acid ethyl ester (S-1k)

^{1}H-NMR, $CDCl_3$, 400 MHz

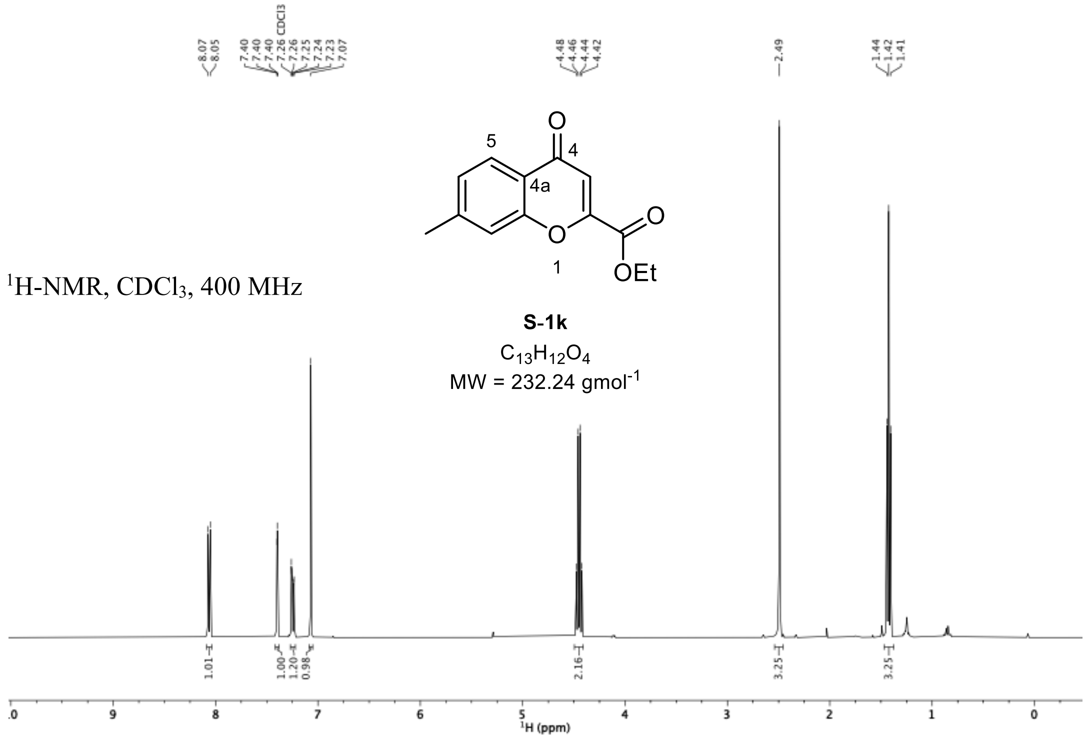

^{13}C-NMR, $CDCl_3$, 101 MHz

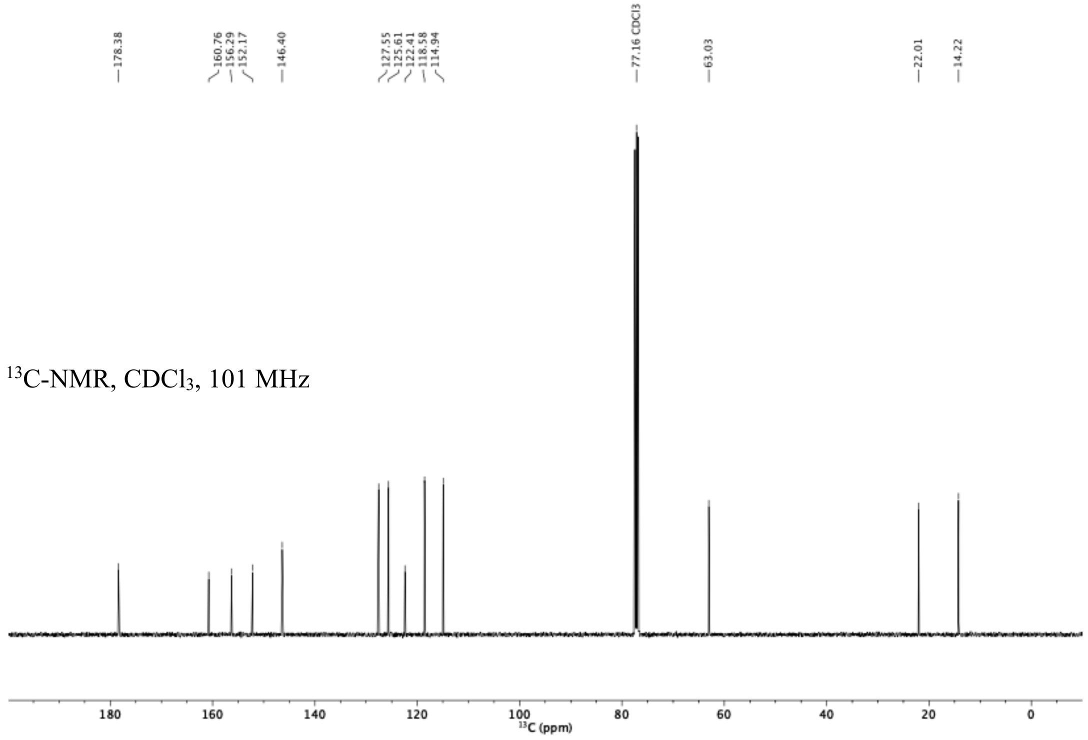

8-Methyl-4-oxo-4*H*-chromene-2-carboxylic acid ethyl ester (S-1l)

^{1}H-NMR, $CDCl_3$, 400 MHz

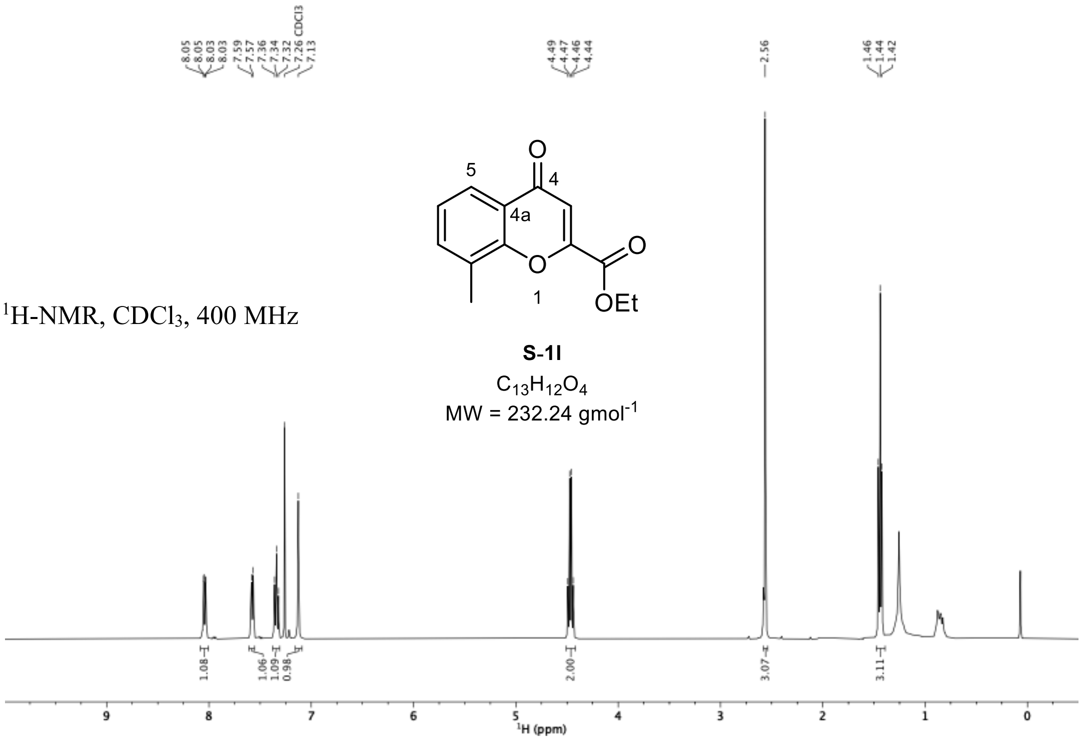

^{13}C-NMR, $CDCl_3$, 101 MHz

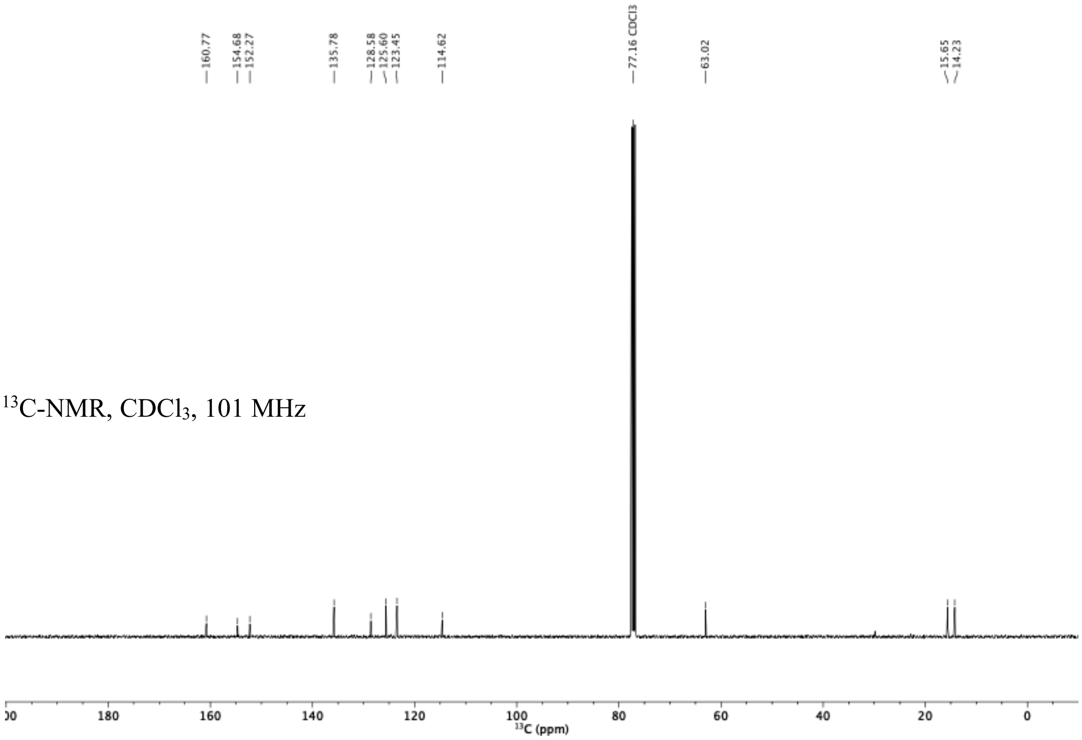

6,7,8,9-Tetrahydro-4-oxo-4*H*-benzo[*g*]chromene-2-carboxylic acid ethyl ester (S-1m)

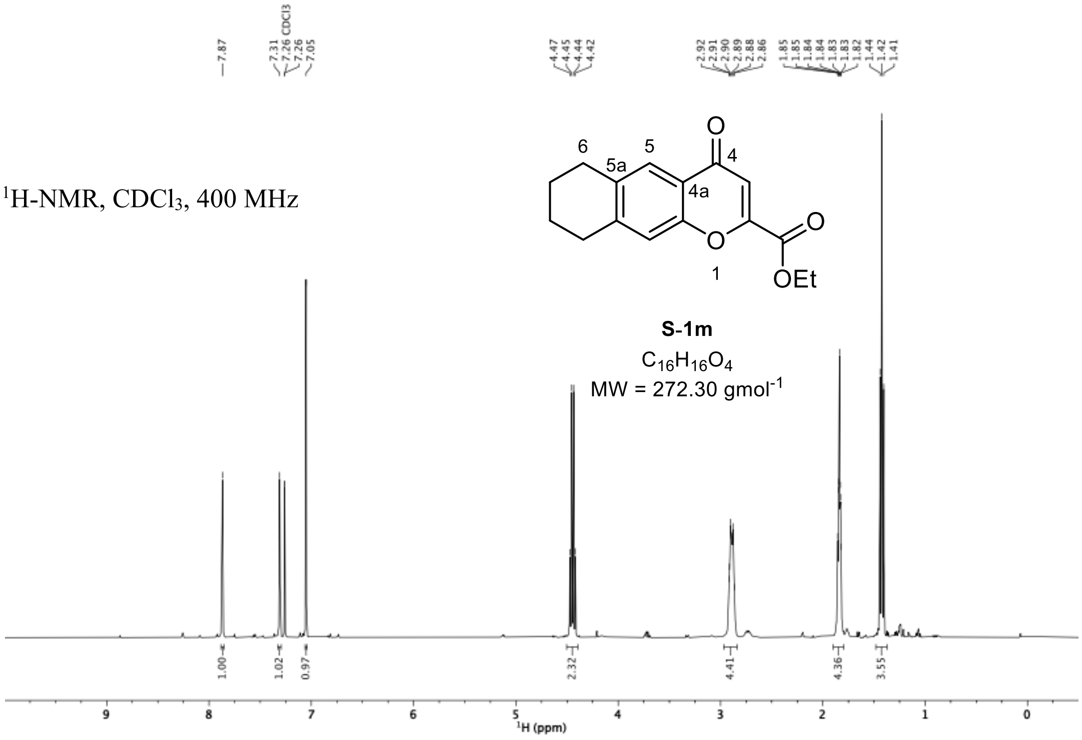

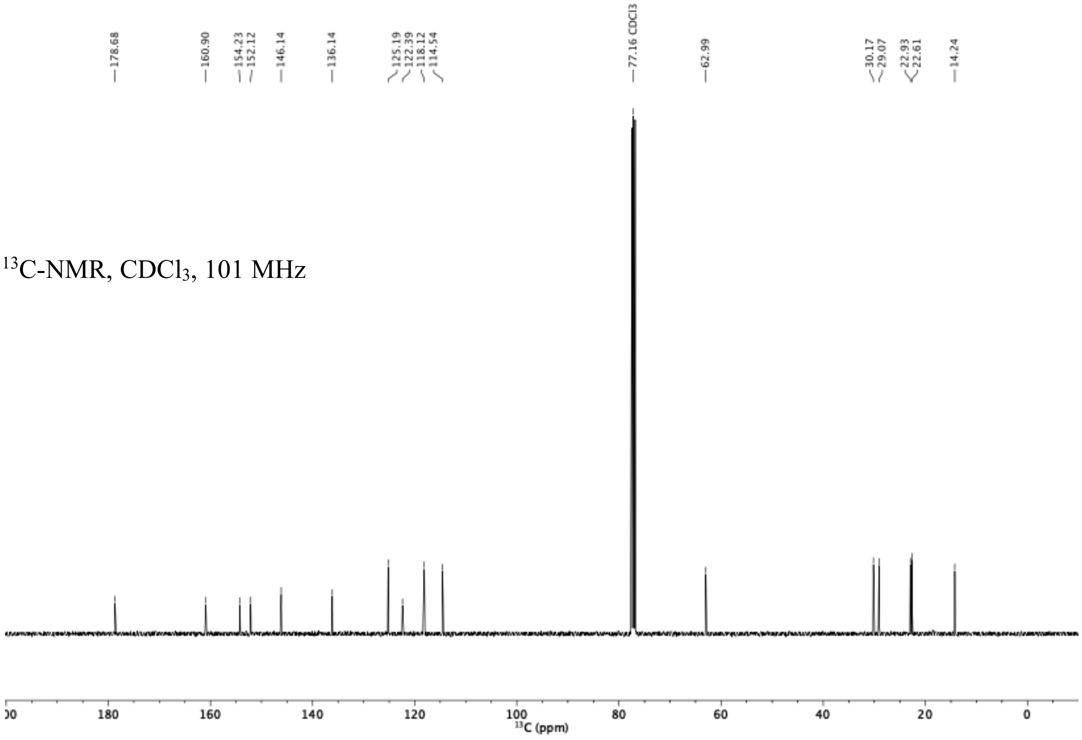

7-Methoxy-4-oxo-4*H*-benzo[*g*]chromene-2-carboxylic acid ethyl ester (S-1o)

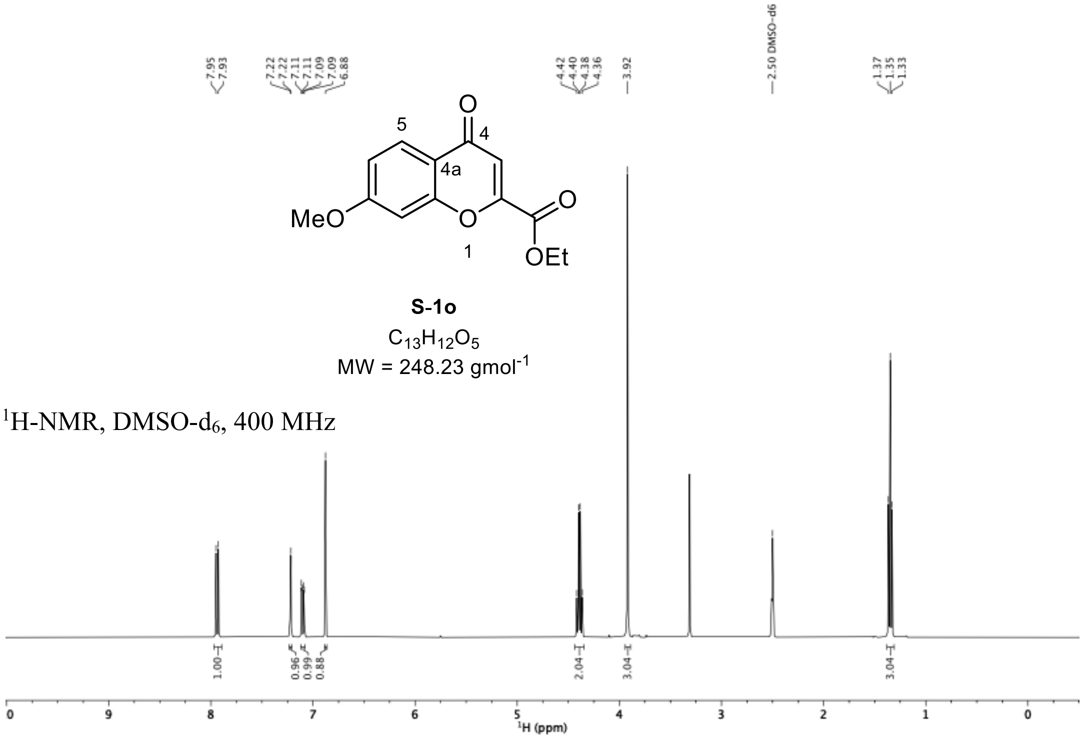

S-1o

$C_{13}H_{12}O_5$

MW = 248.23 gmol[-1]

^{1}H-NMR, DMSO-d$_6$, 400 MHz

^{13}C-NMR, DMSO-d$_6$, 101 MHz

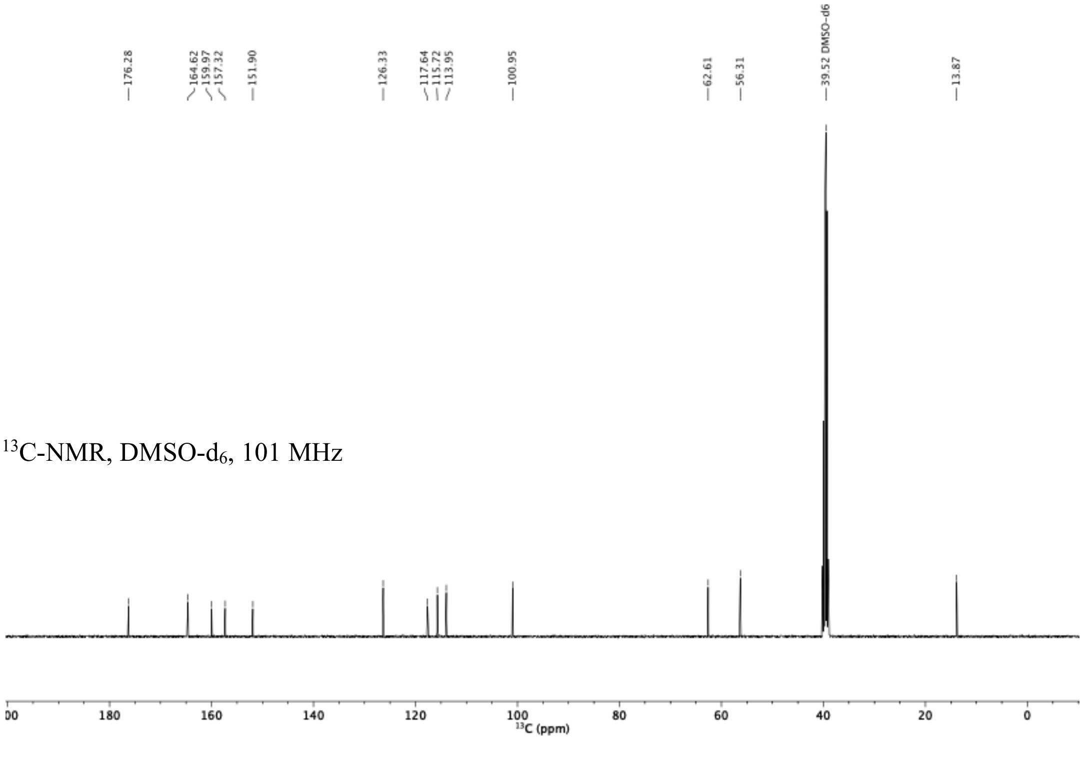

7-Fluoro-4-oxo-4*H*-benzo[*g*]chromene-2-carboxylic acid ethyl ester (S-1p)

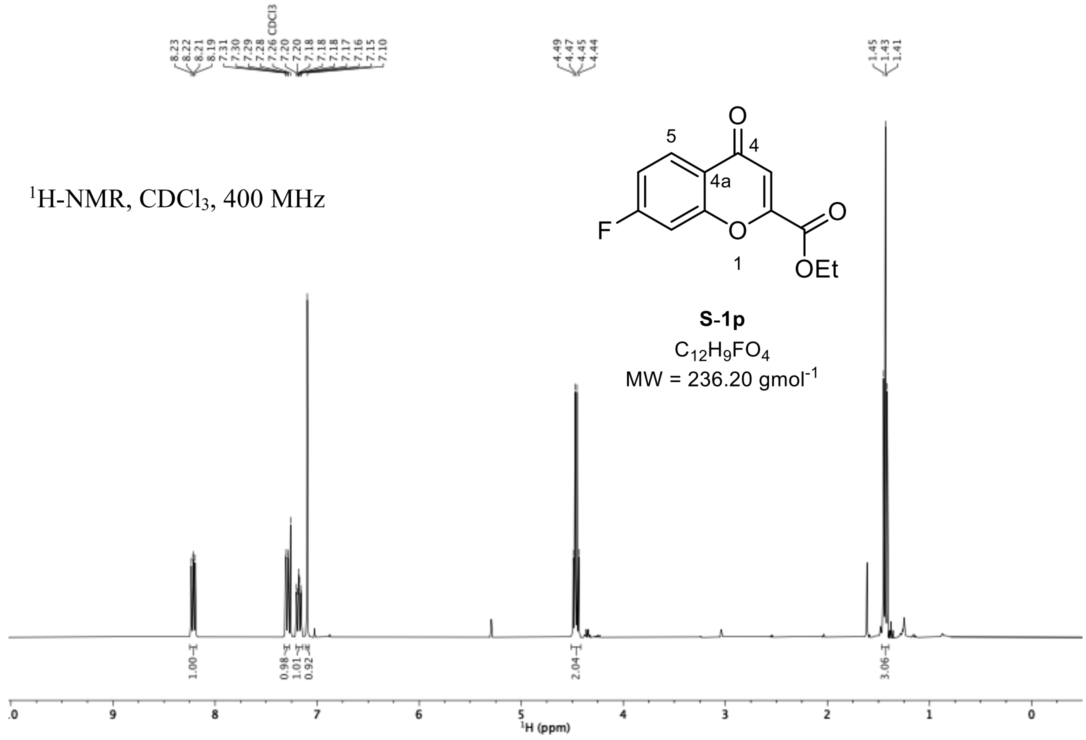

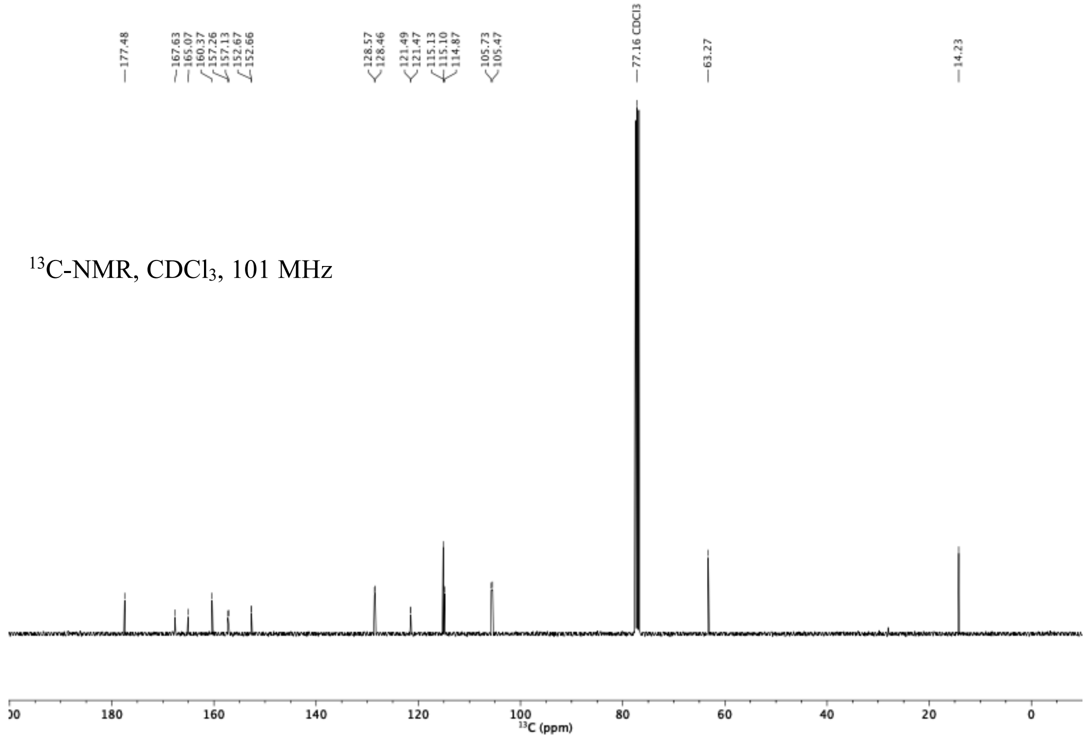

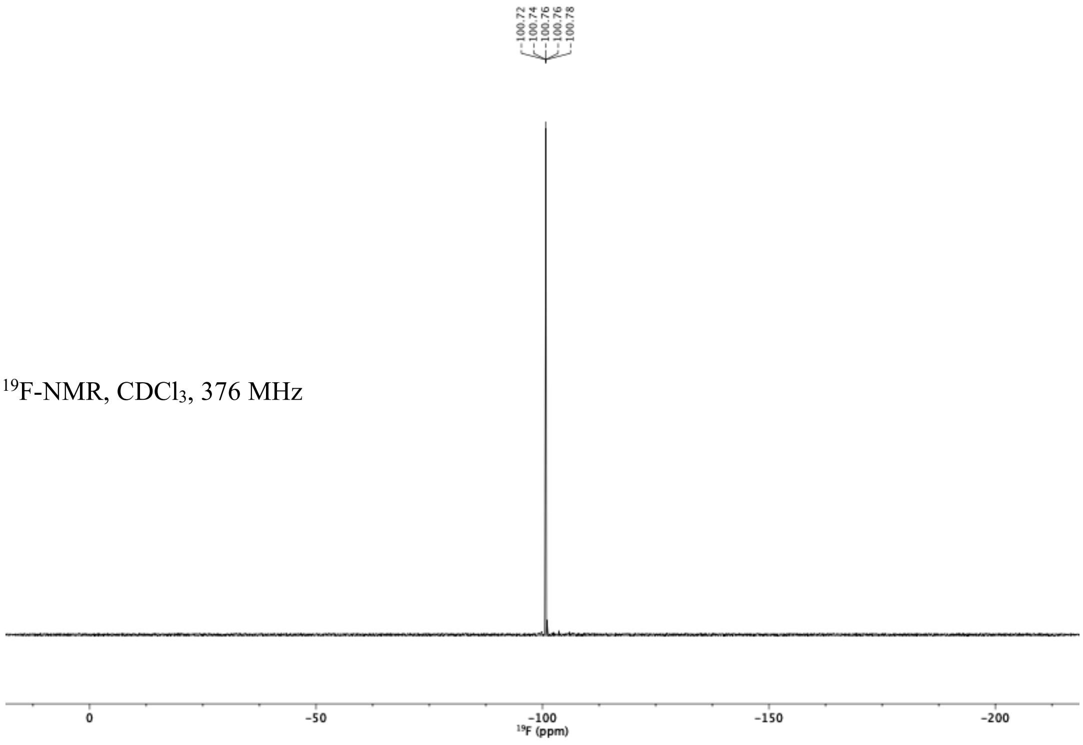

^{19}F-NMR, $CDCl_3$, 376 MHz

8-Fluoro-4-oxo-4*H*-chromene-2-carboxylic acid ethyl ester (S-1q)

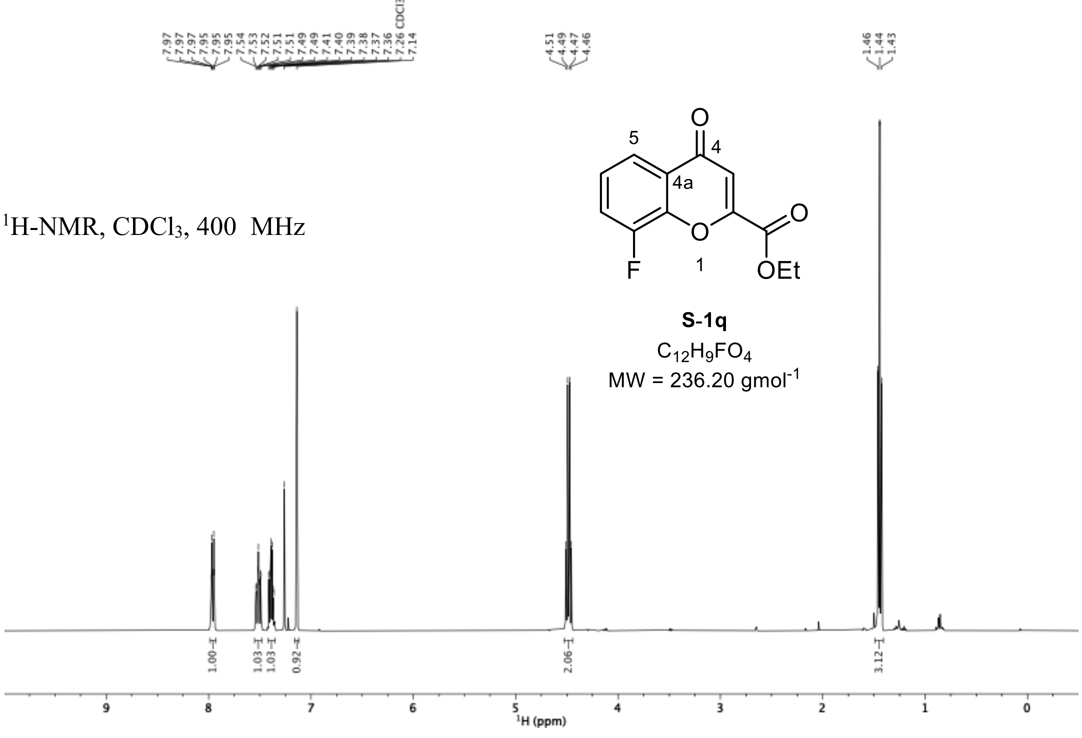

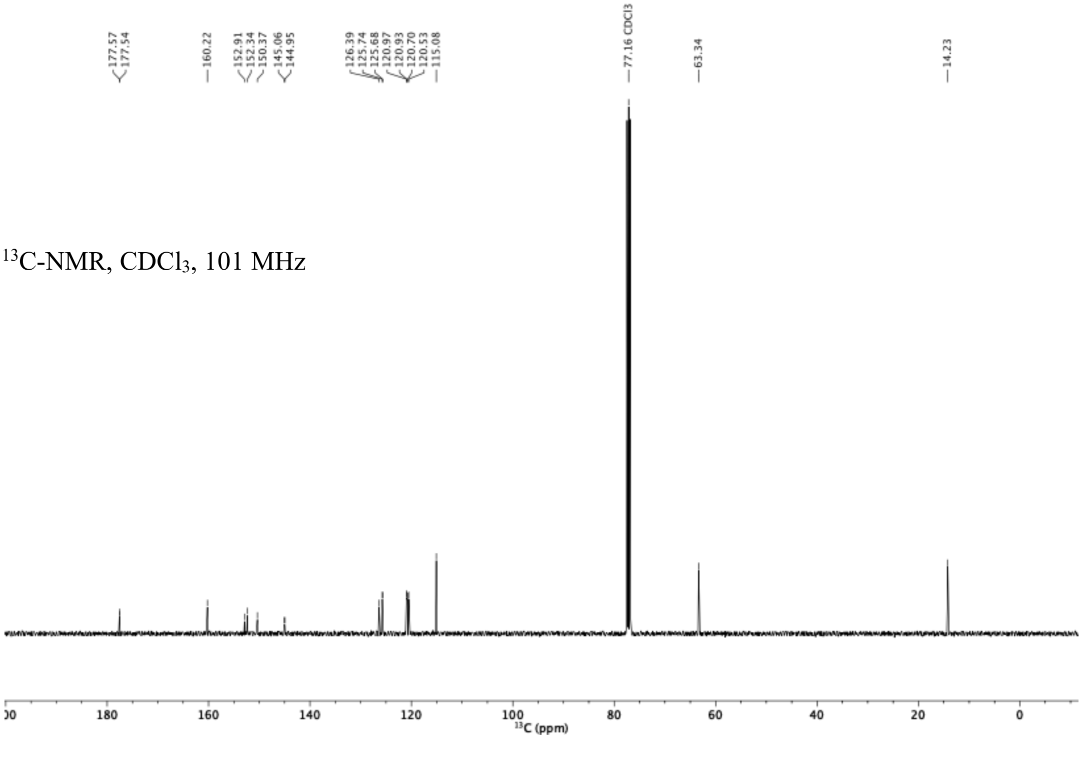

^{19}F-NMR, $CDCl_3$, 376 MHz

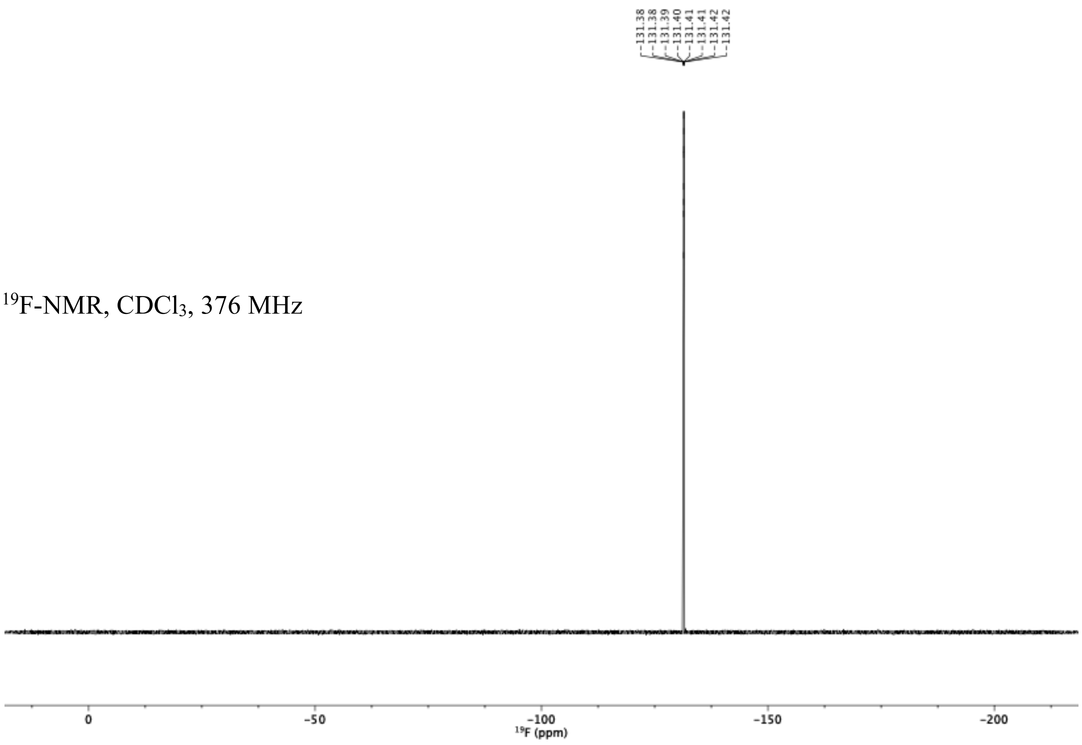

6-Ethylchromane-2-carboxylic acid ethyl ester (*rac*-**S-2i**)

^{1}H-NMR, CDCl$_3$, 400 MHz

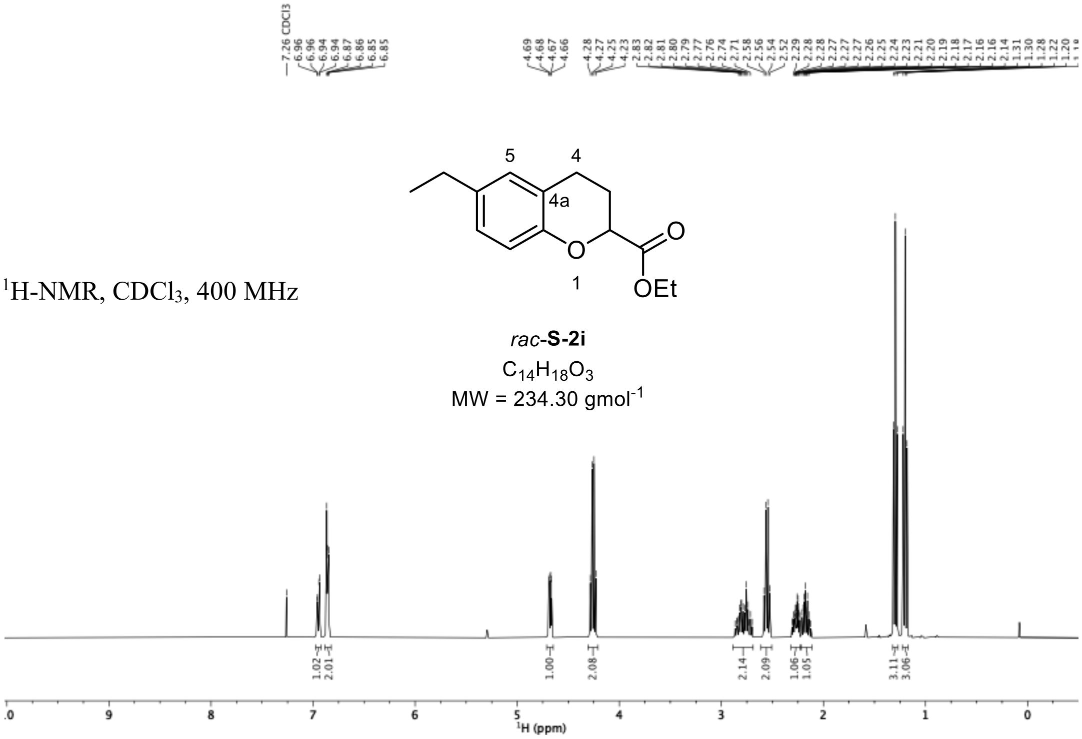

^{13}C-NMR, CDCl$_3$, 101 MHz

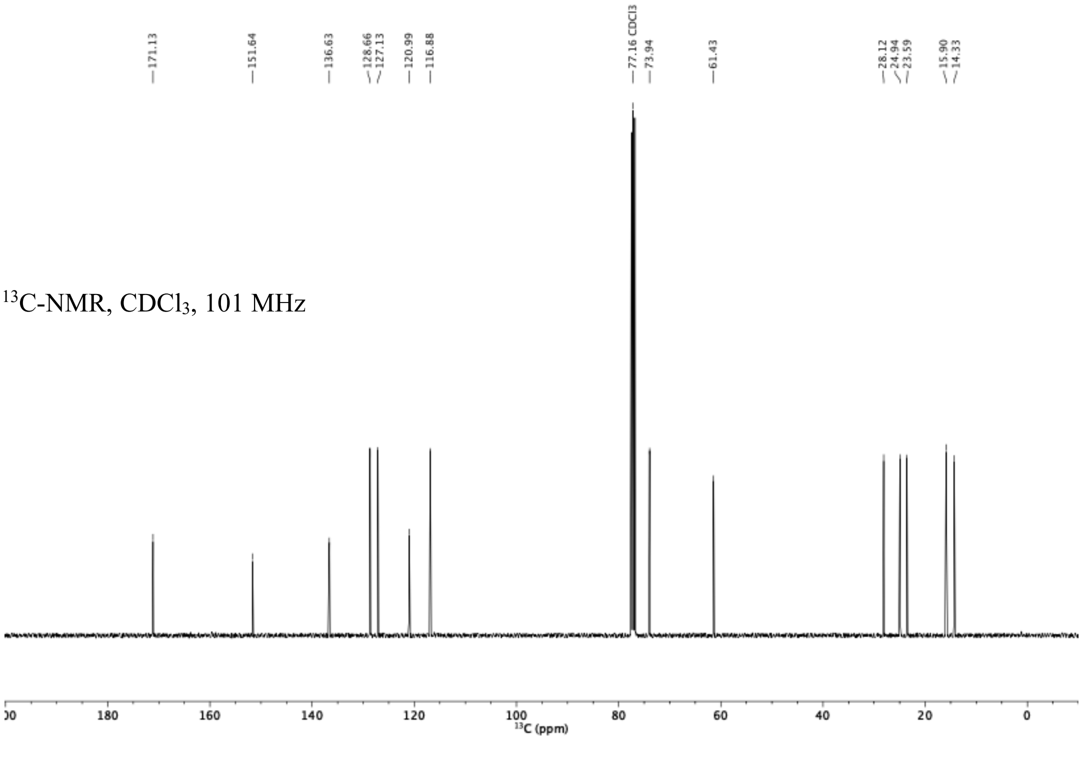

5-Methylchromane-2-carboxylic acid ethyl ester (*rac*-**S-2j**)

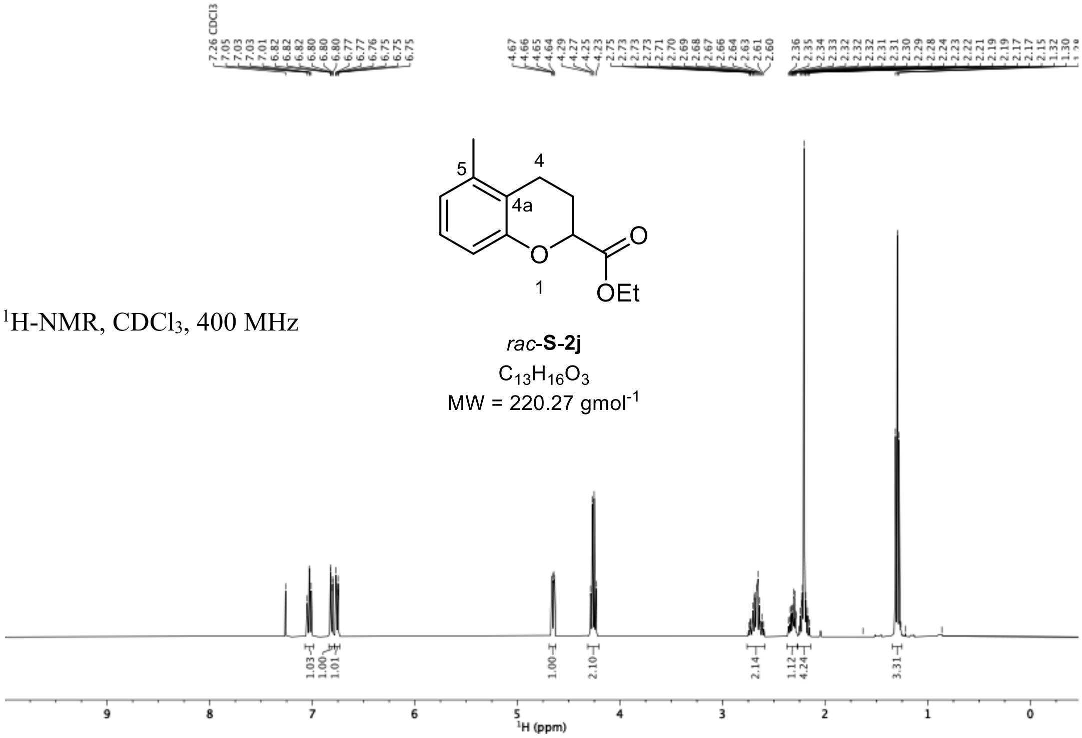

^{1}H-NMR, $CDCl_3$, 400 MHz

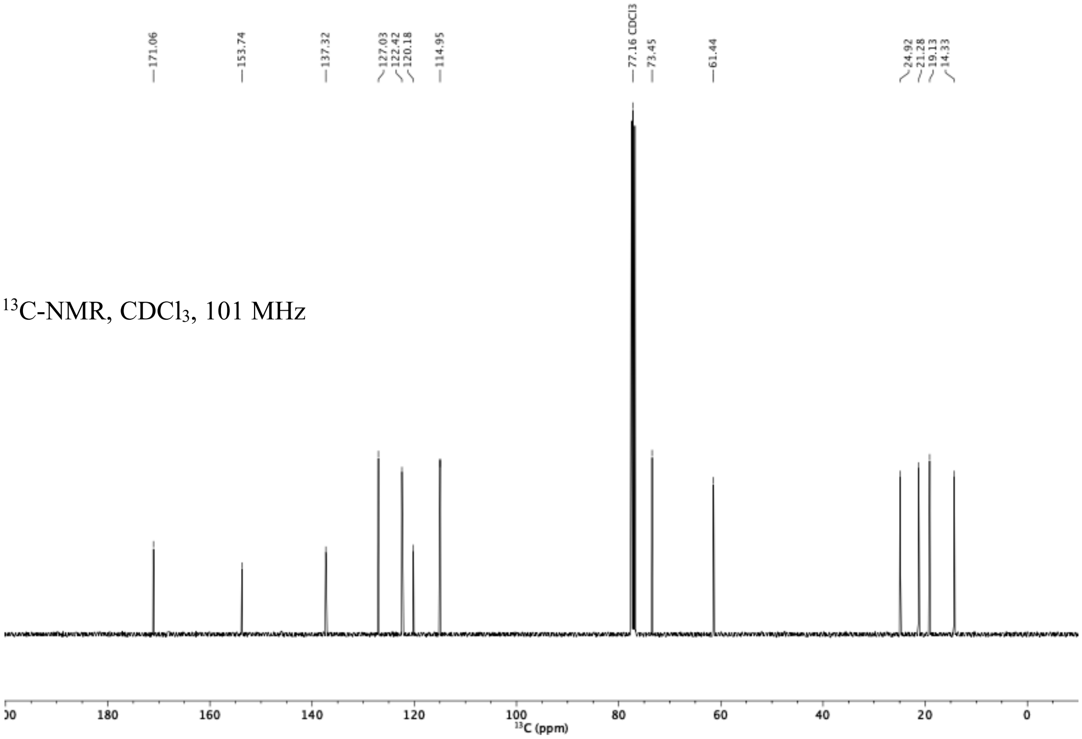

^{13}C-NMR, $CDCl_3$, 101 MHz

7-Methylchromane-2-carboxylic acid ethyl ester (*rac*-**S-2k**)

^{1}H-NMR, $CDCl_3$, 400 MHz

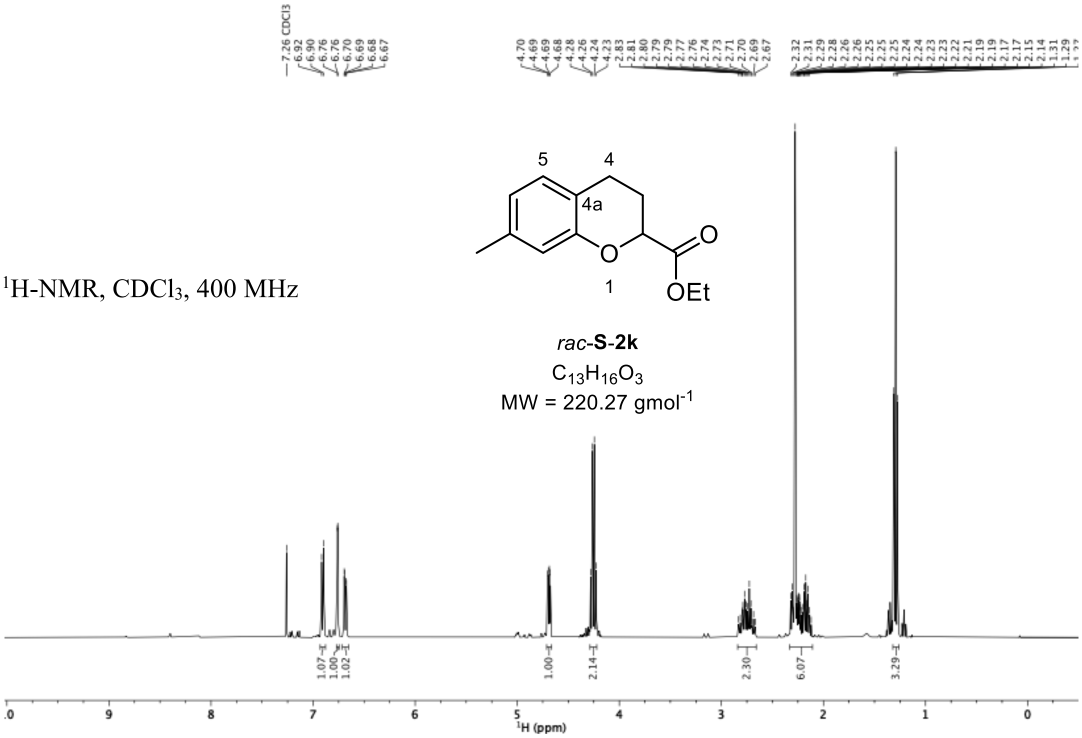

^{13}C-NMR, $CDCl_3$, 101 MHz

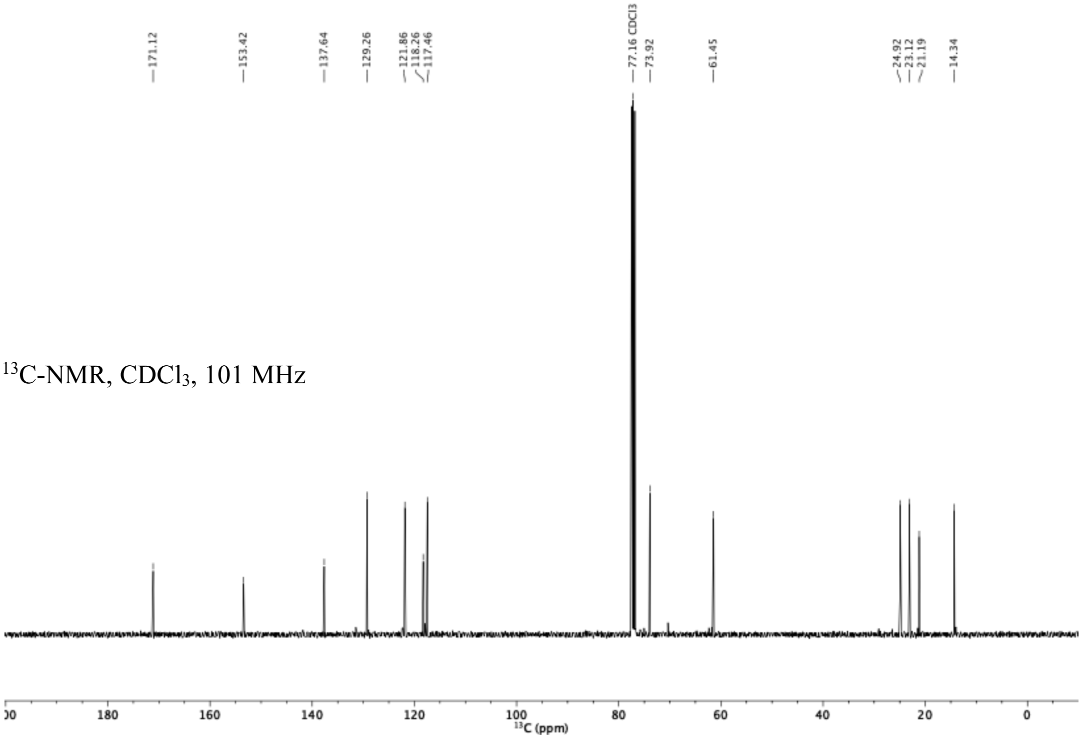

8-Methylchromane-2-carboxylic acid ethyl ester (*rac*-**S-2l**)

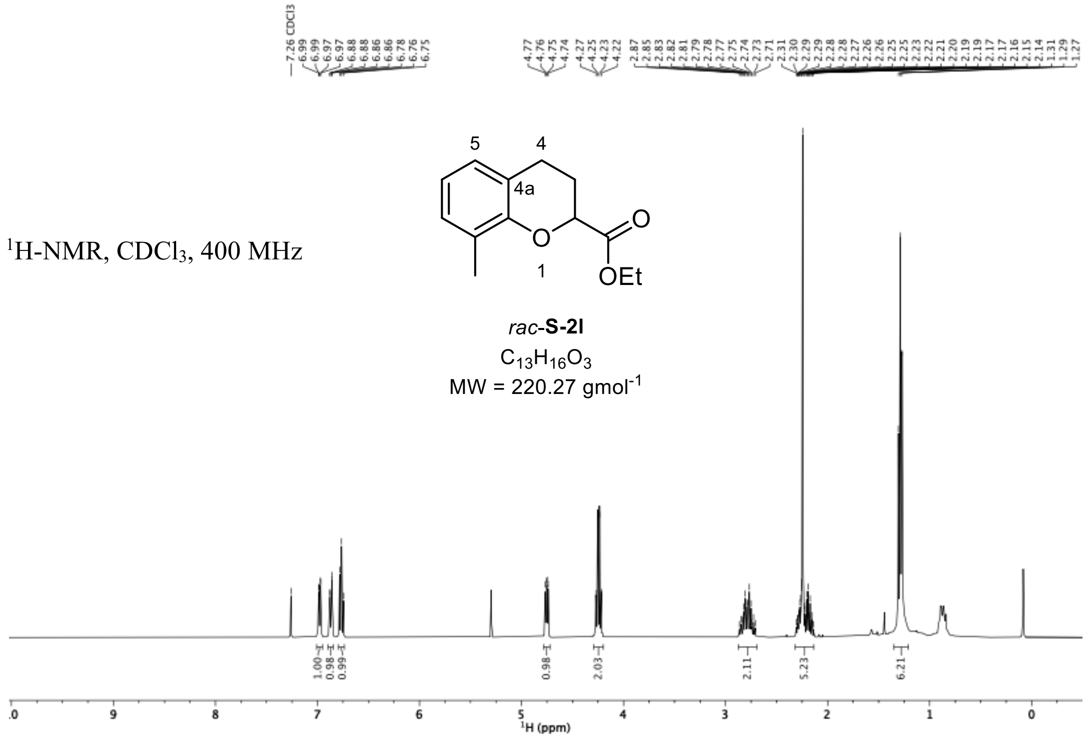

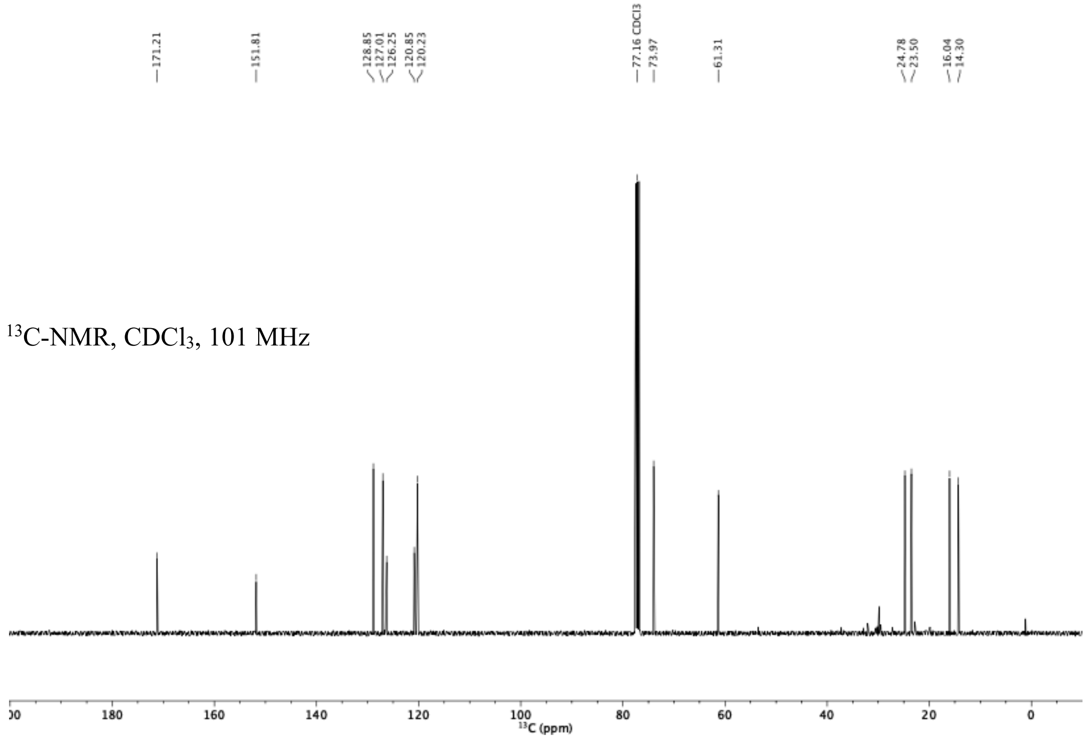

6,7,8,9-Tetrahydro-benzo[*g*]chromane-2-carboxylic acid ethyl ester (*rac*-S-2m)

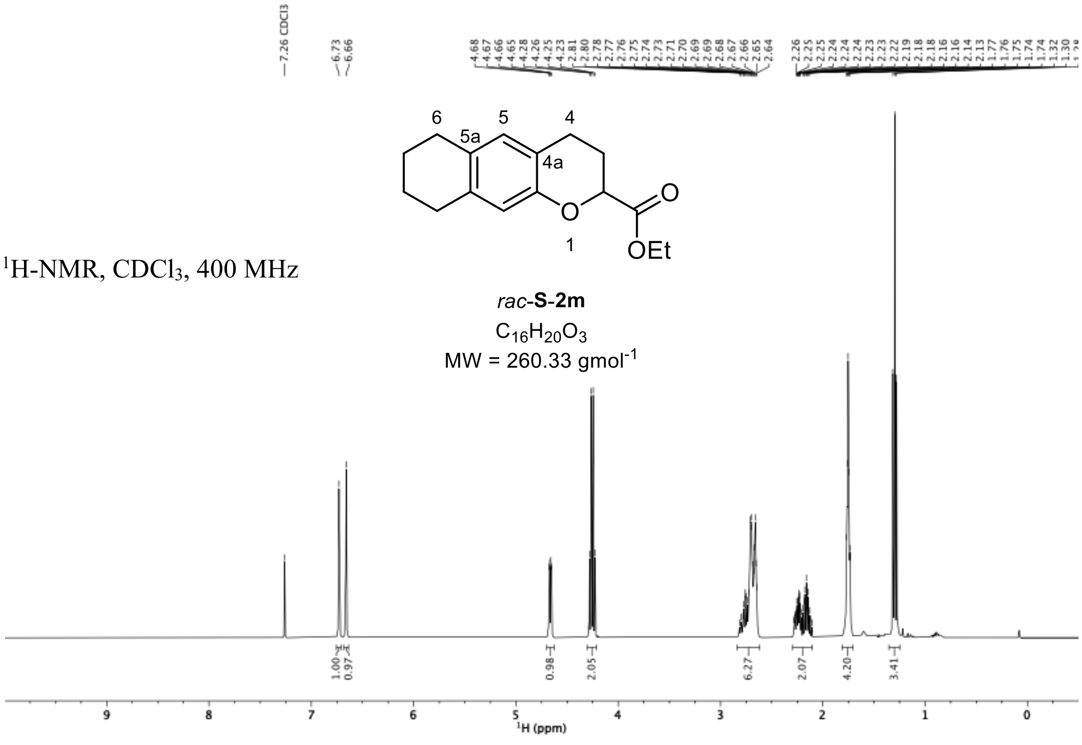

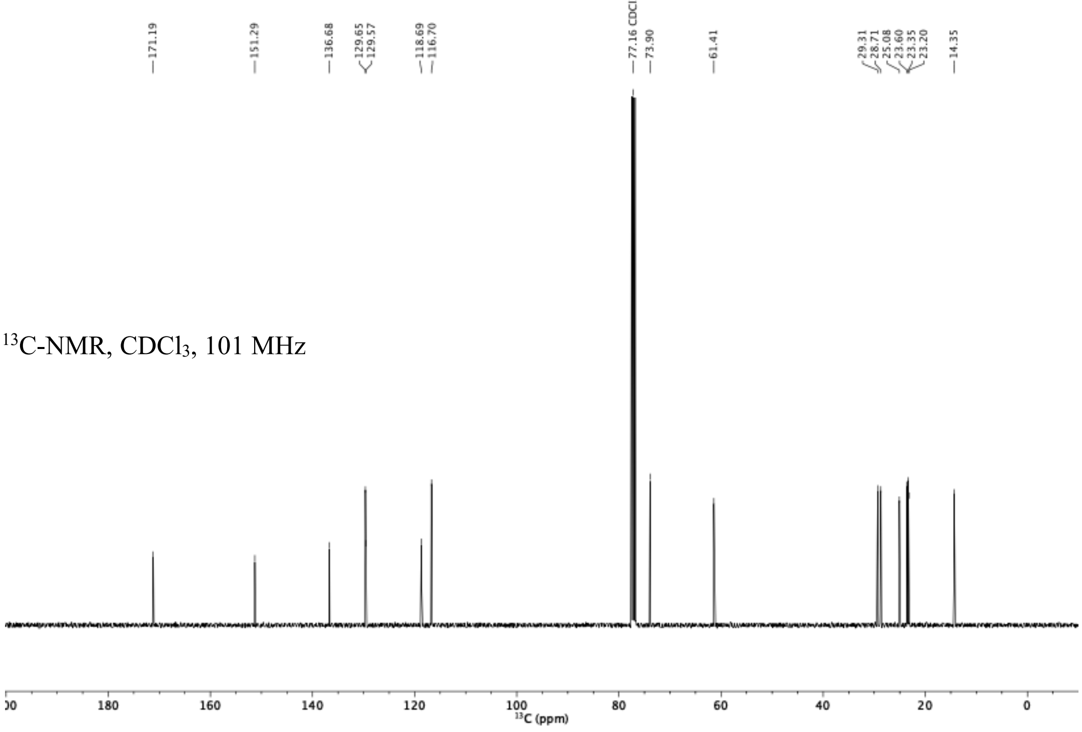

7-Methoxychromane-2-carboxylic acid ethyl ester (*rac*-**S-2o**)

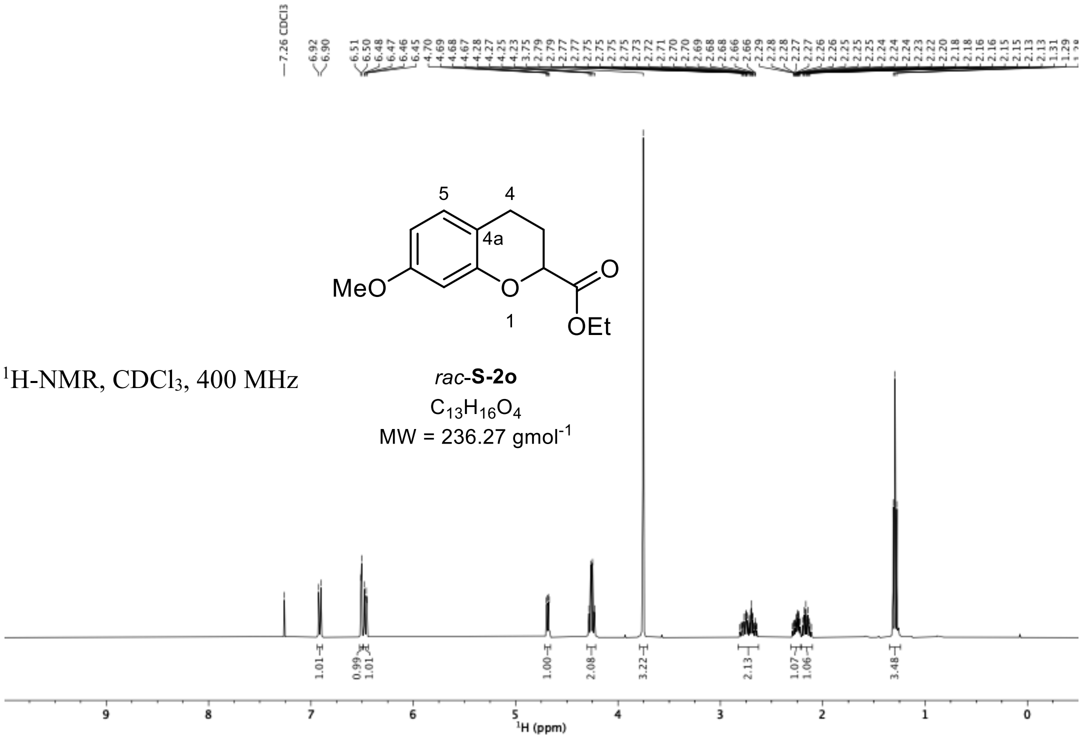

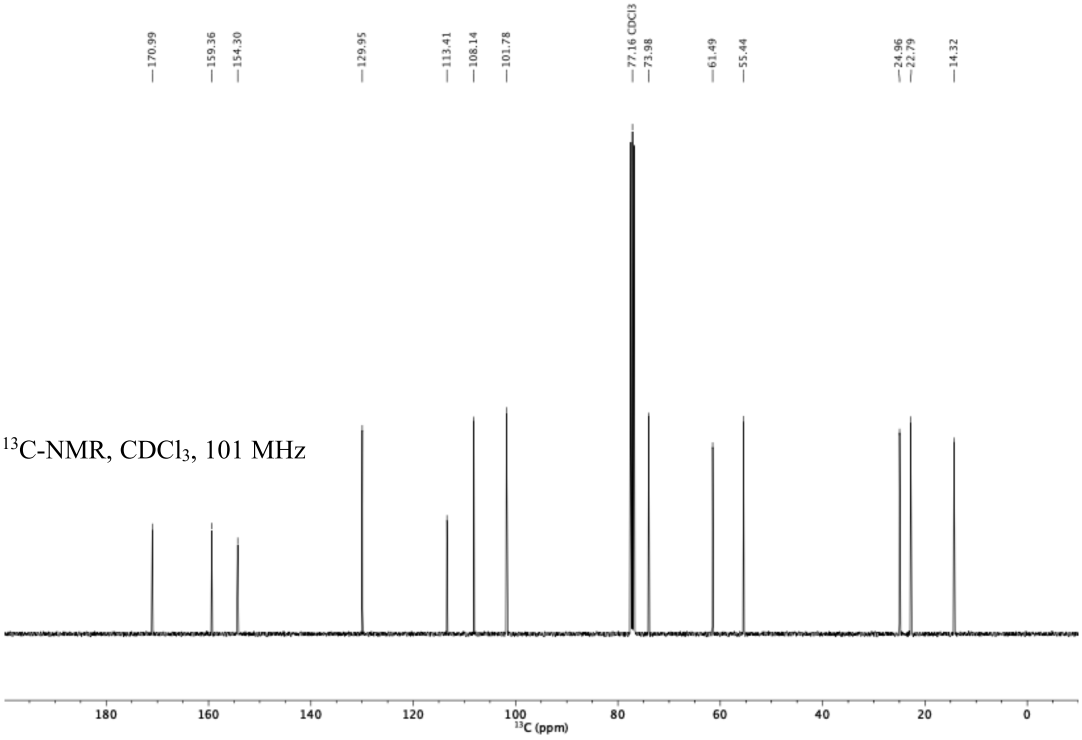

7-Fluorochromane-2-carboxylic acid ethyl ester (*rac*-S-2p)

^{1}H-NMR, $CDCl_3$, 400 MHz

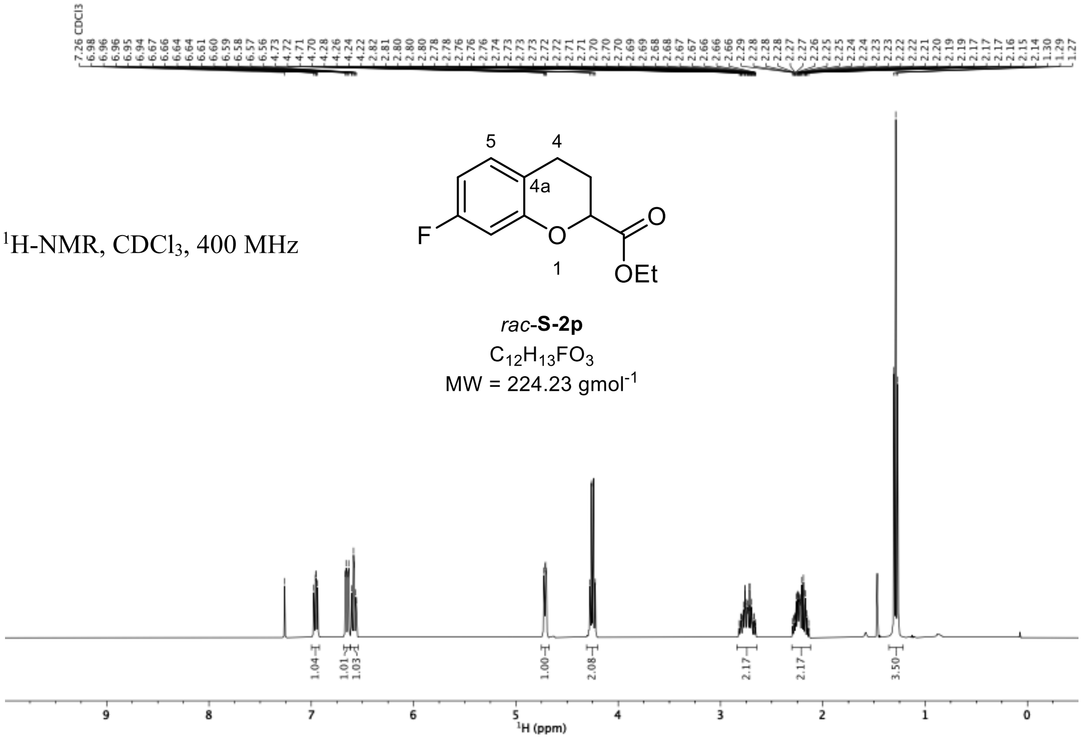

^{13}C-NMR, $CDCl_3$, 101 MHz

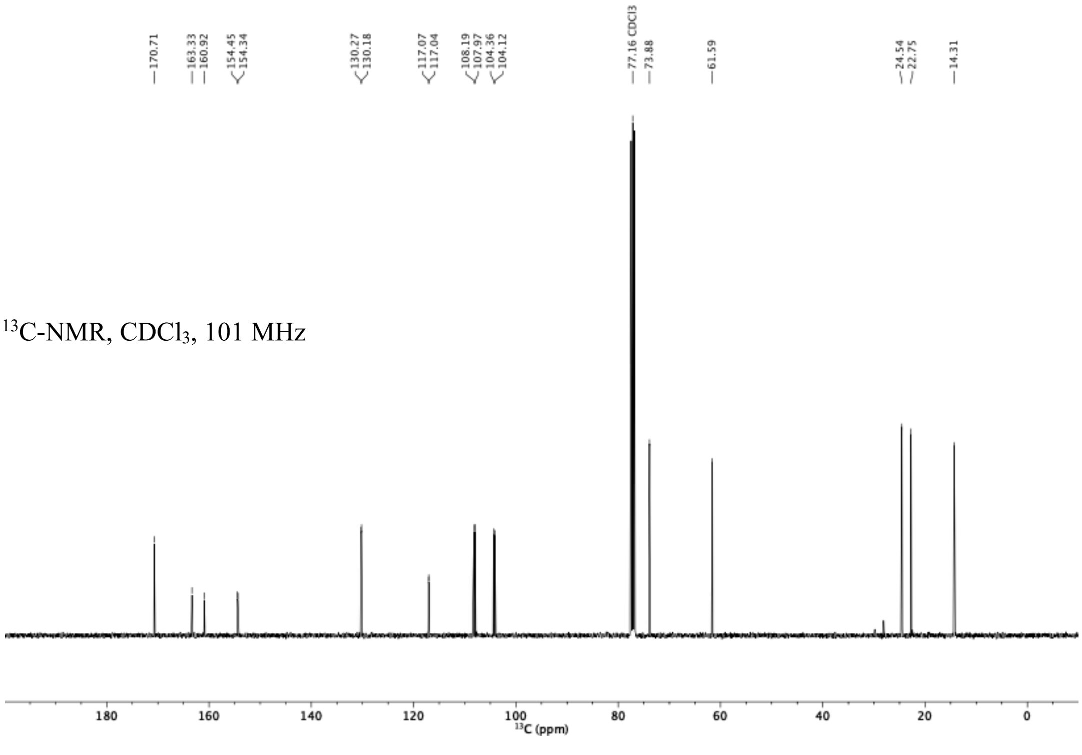

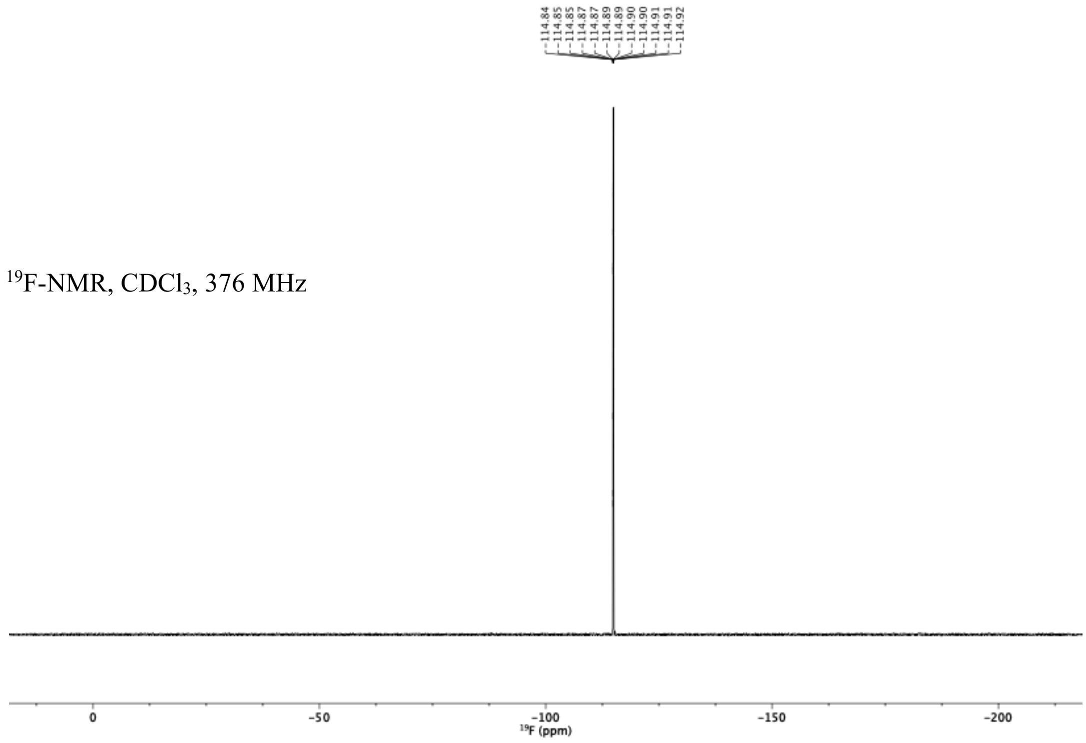
-114.84
-114.85
-114.85
-114.87
-114.87
-114.89
-114.89
-114.90
-114.90
-114.91
-114.91
-114.92
19F-NMR, CDCl3, 376 MHz
0
-50
-100
-150
-200
19F (ppm)

8-Fluorochromane-2-carboxylic acid ethyl ester (*rac*-**S-2q**)

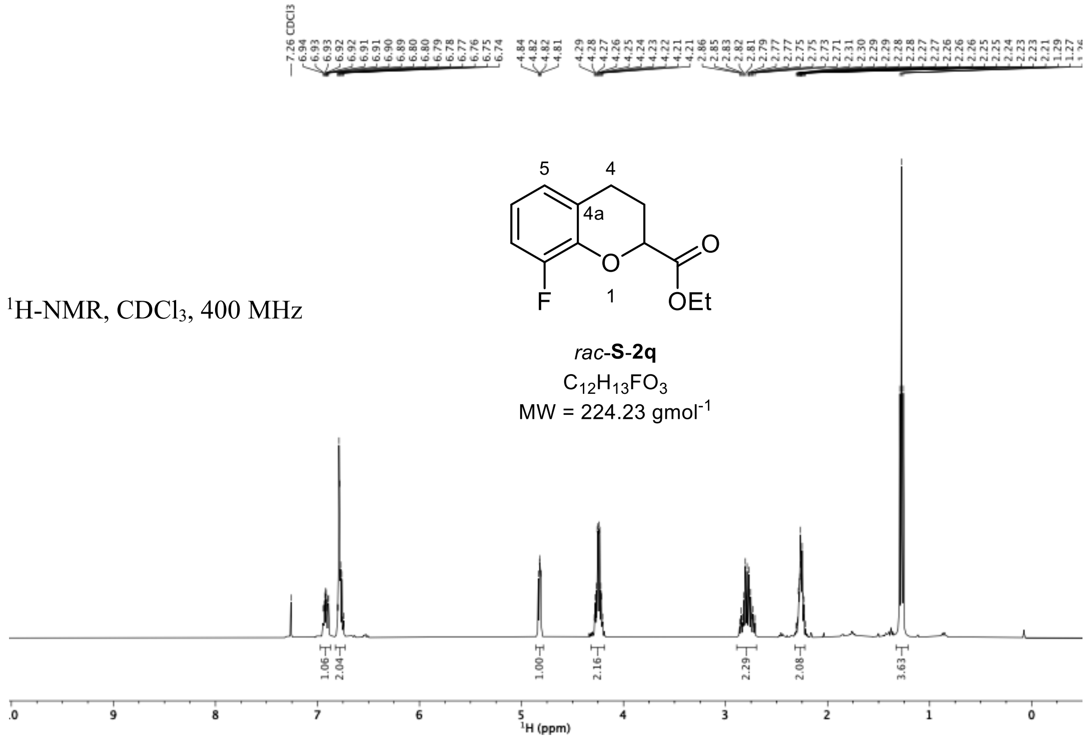

^{1}H-NMR, $CDCl_3$, 400 MHz

rac-**S-2q**
$C_{12}H_{13}FO_3$
MW = 224.23 $gmol^{-1}$

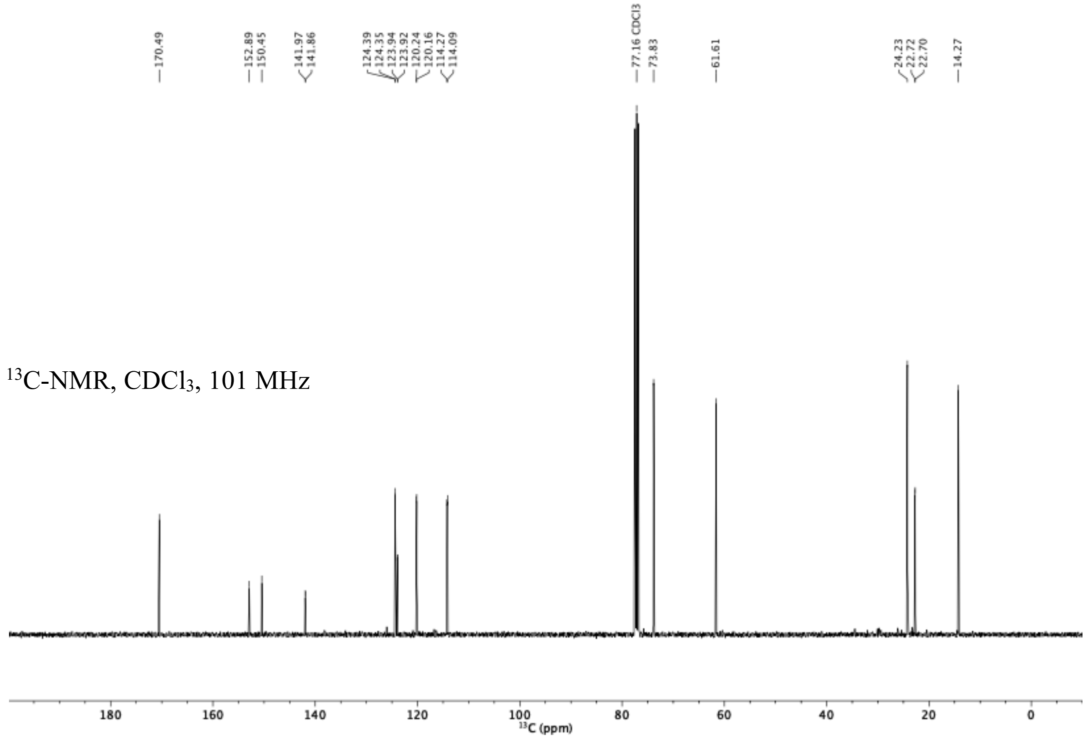

^{13}C-NMR, $CDCl_3$, 101 MHz

^{19}F-NMR, $CDCl_3$, 376 MHz

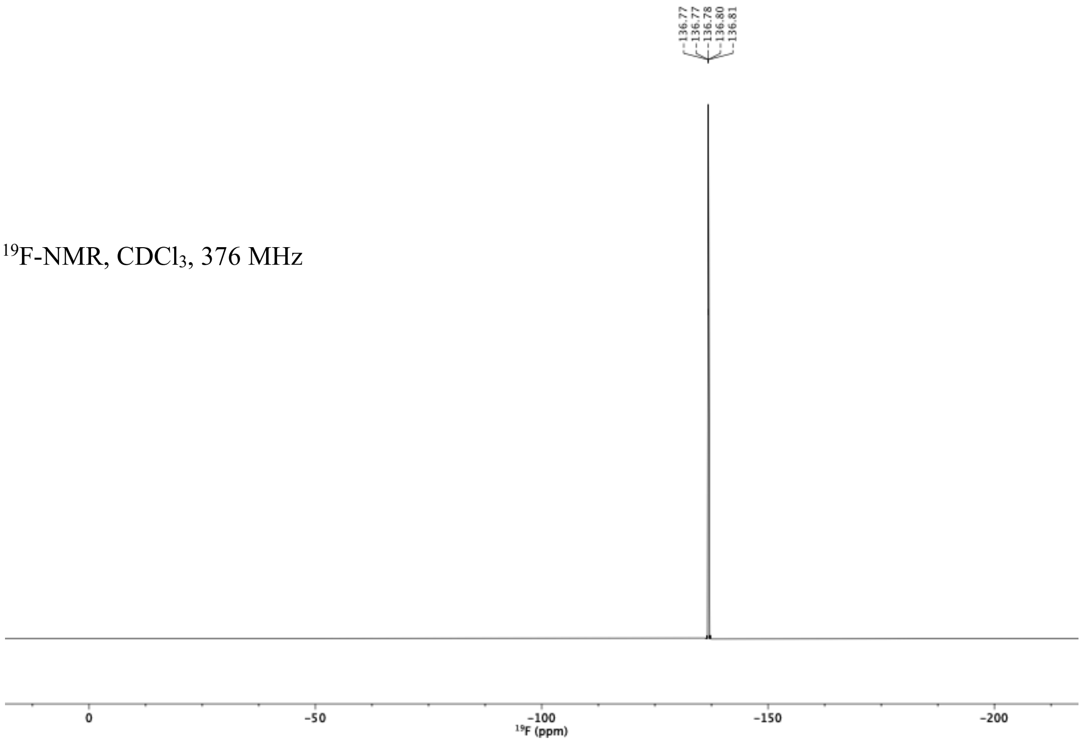

2,3-Dibromo-3-methylbutanoic acid methyl ester (*rac*-S-4)

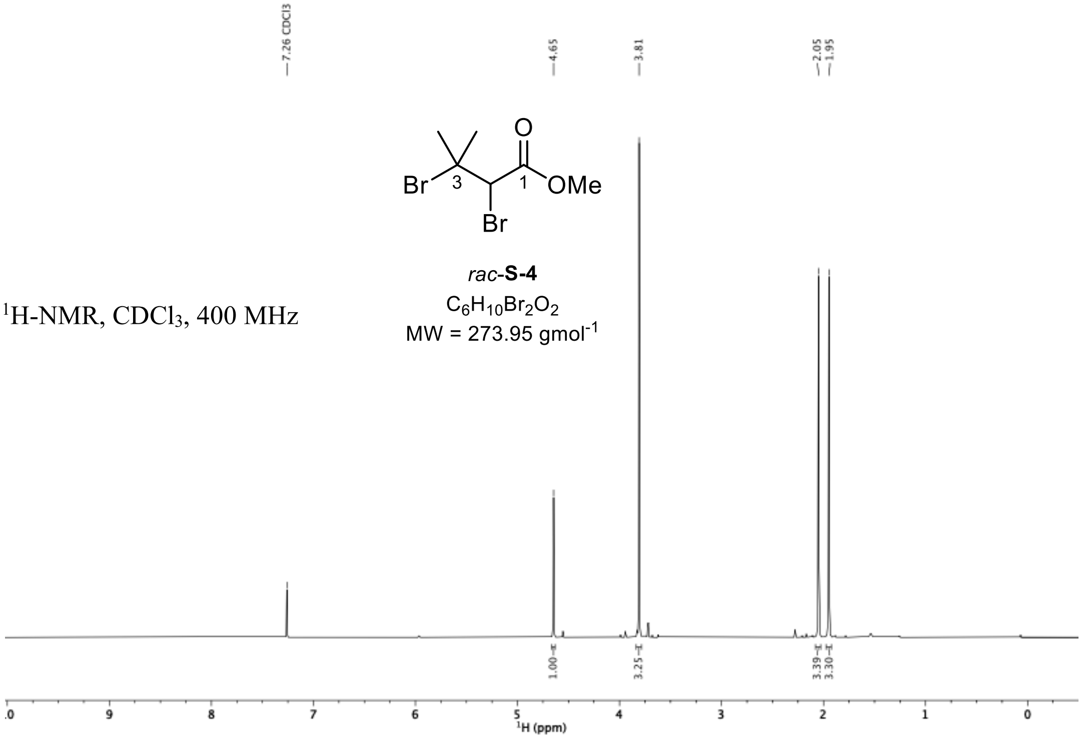

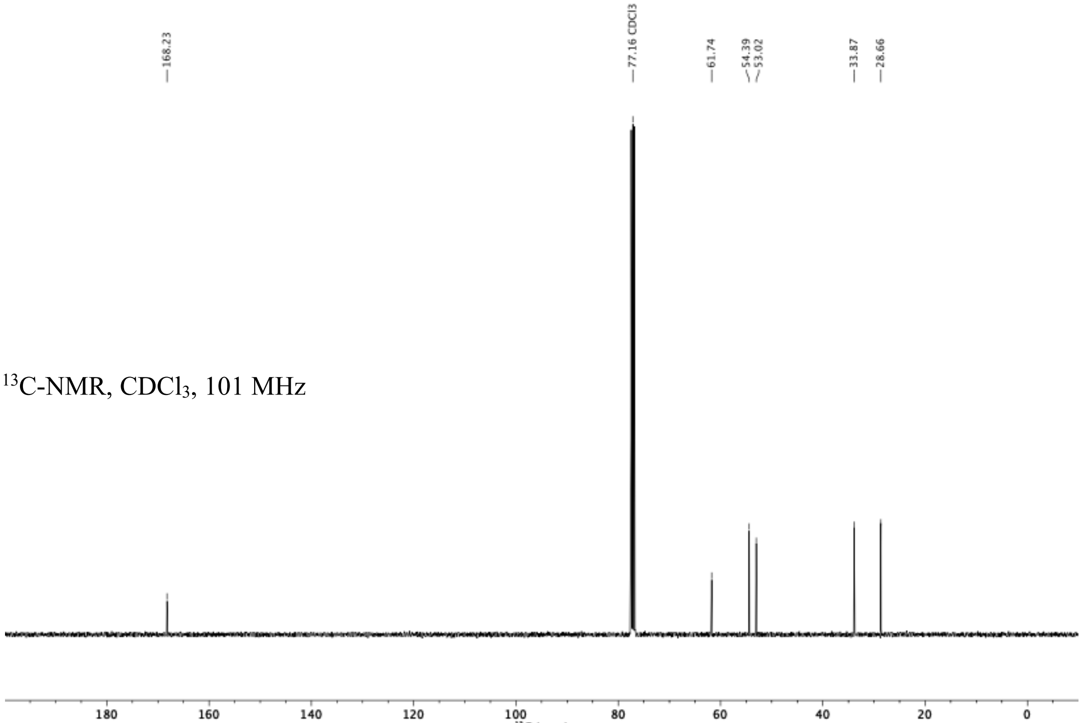

3,3-Dimethyl-1,4-benzodioxane-2-carboxylic acid methyl ester (*rac*-S-2s)

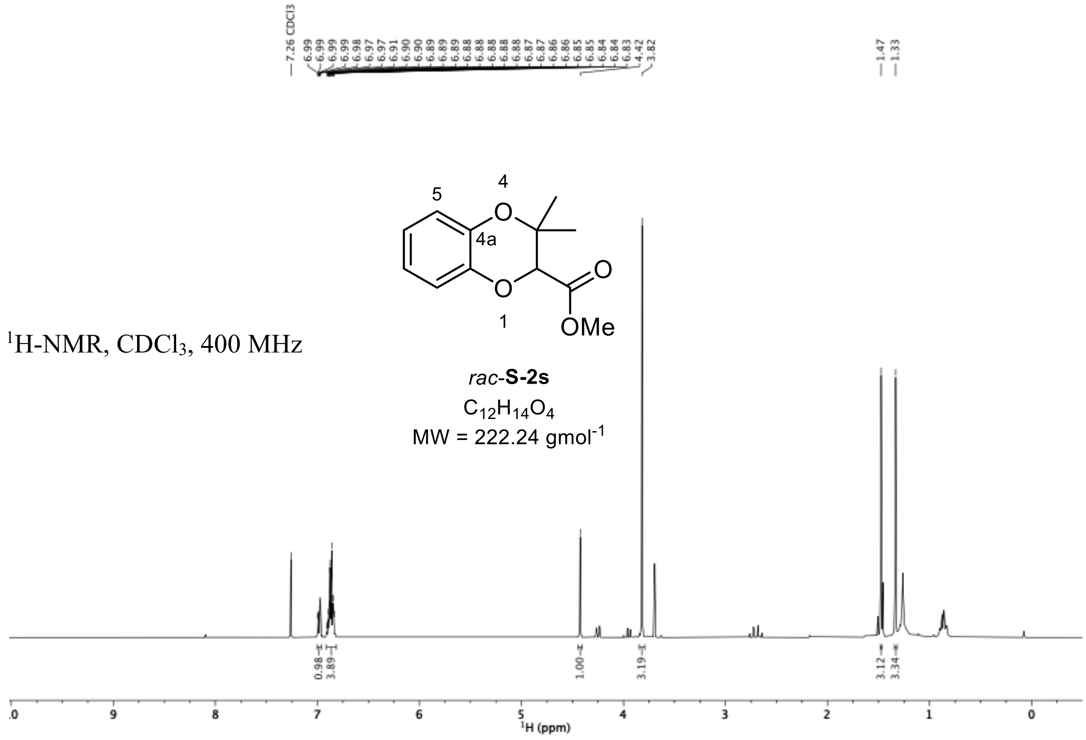

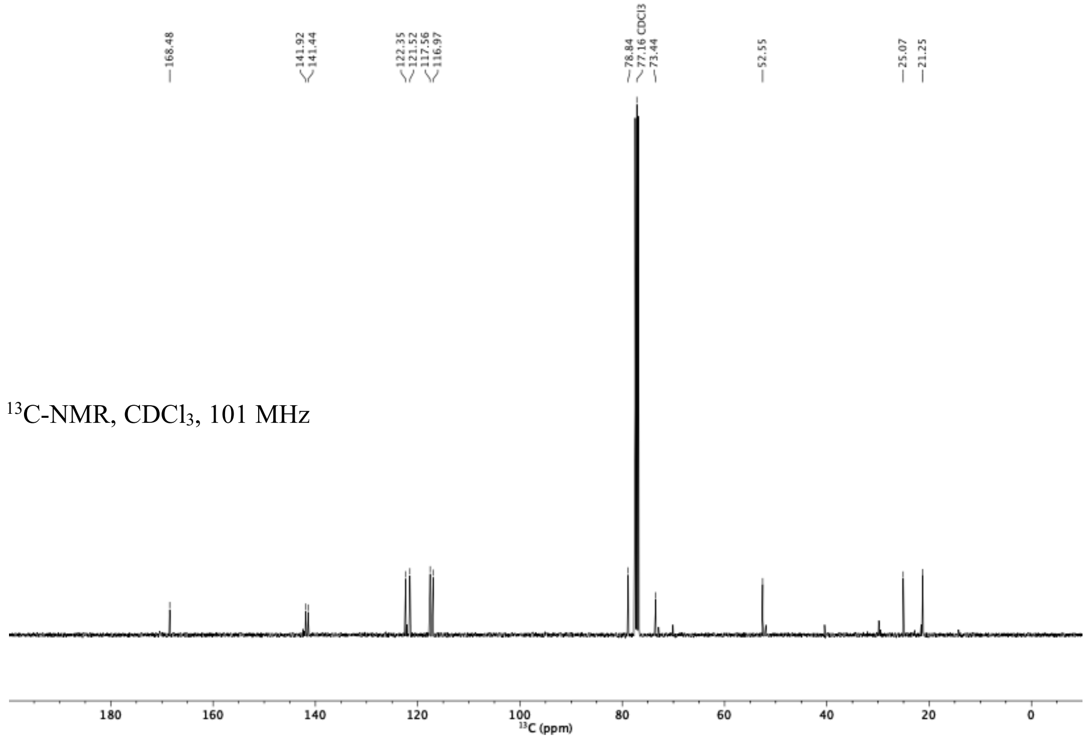

6-Ethylchromane-2-carboxylic acid (*rac*-**S-3i**)

^{1}H-NMR, $CDCl_3$, 400 MHz

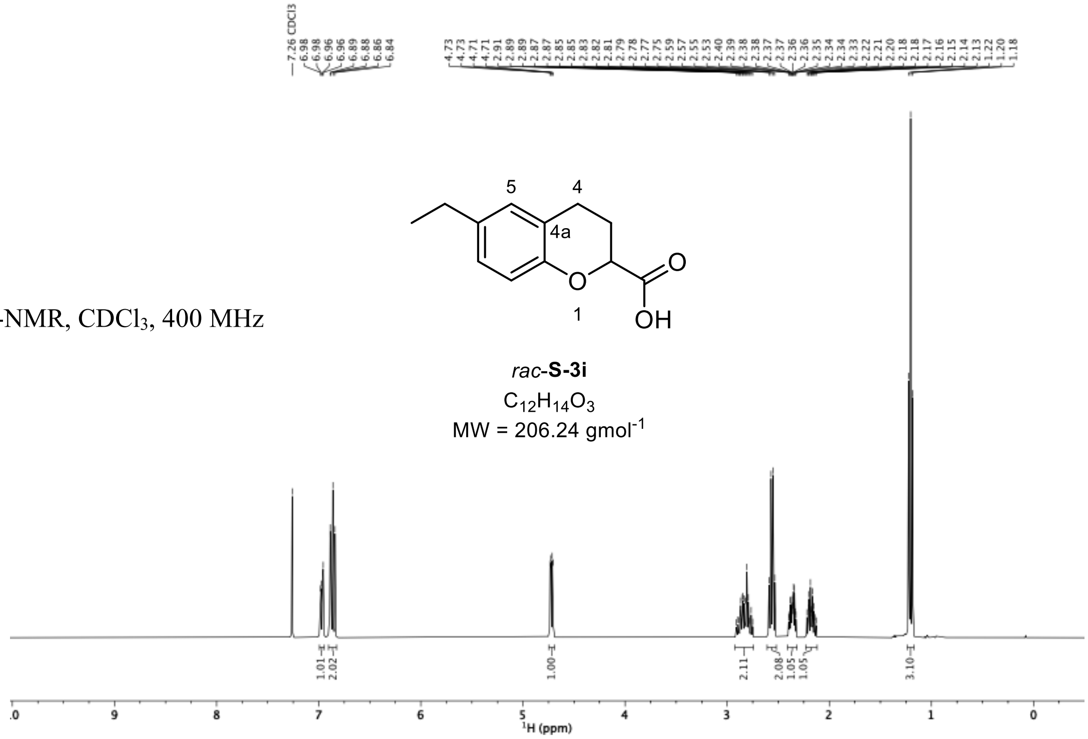

^{13}C-NMR, $CDCl_3$, 101 MHz

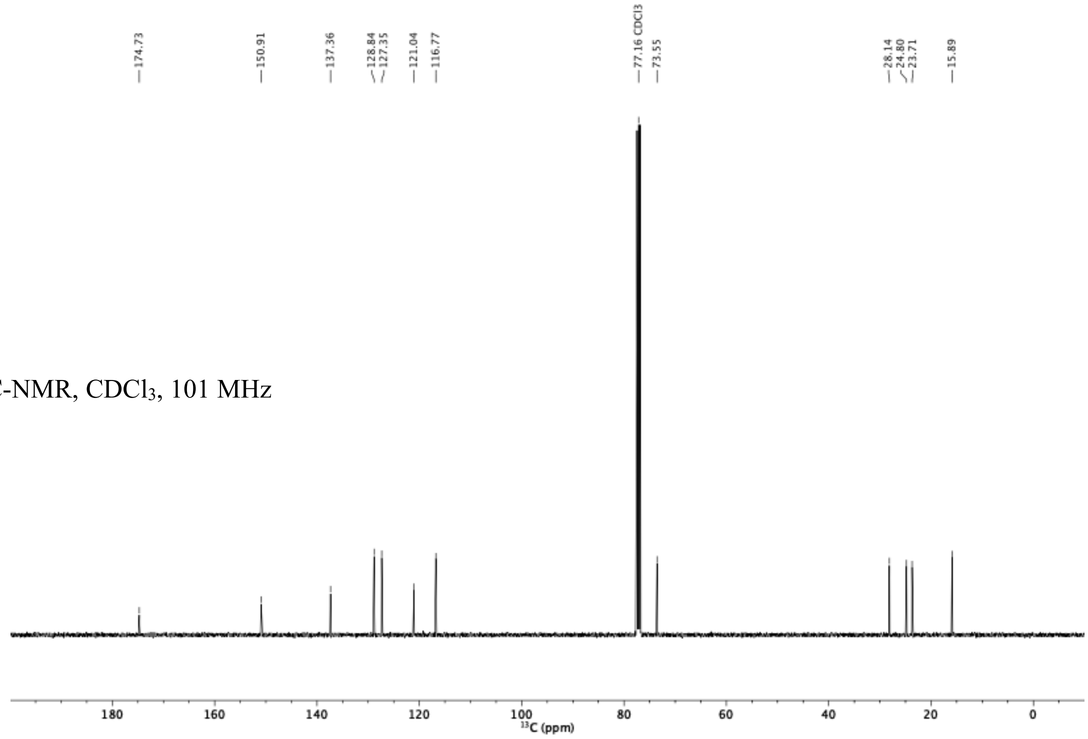

5-Methylchromane-2-carboxylic acid (*rac*-**S-3j**)

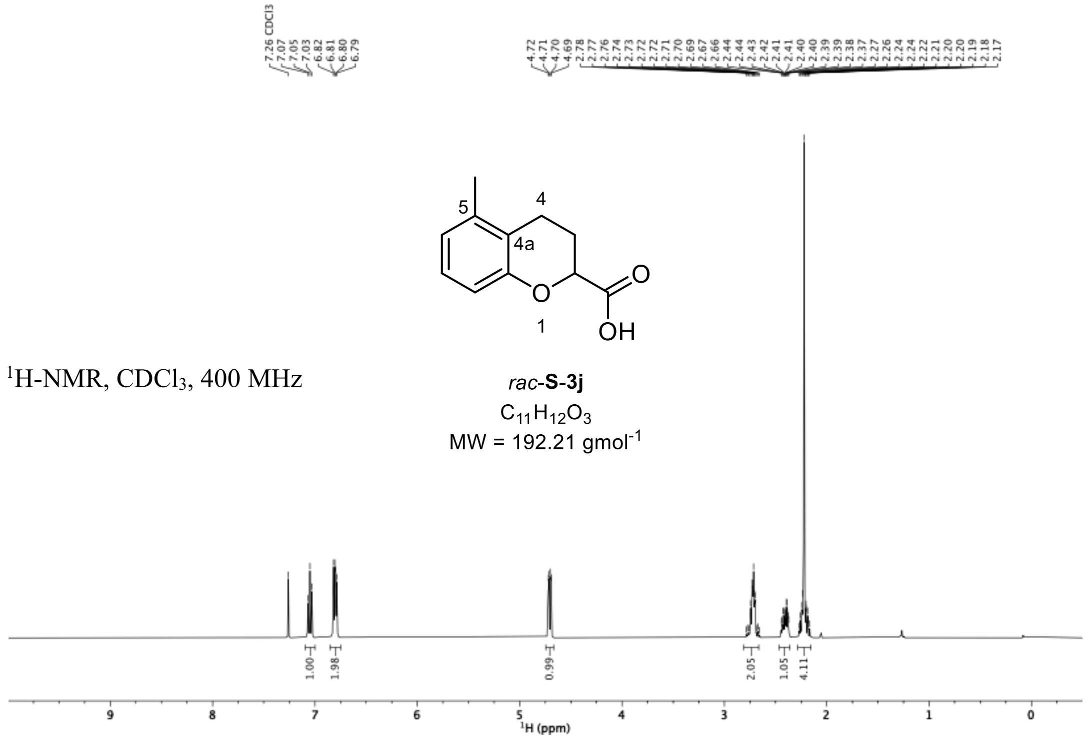

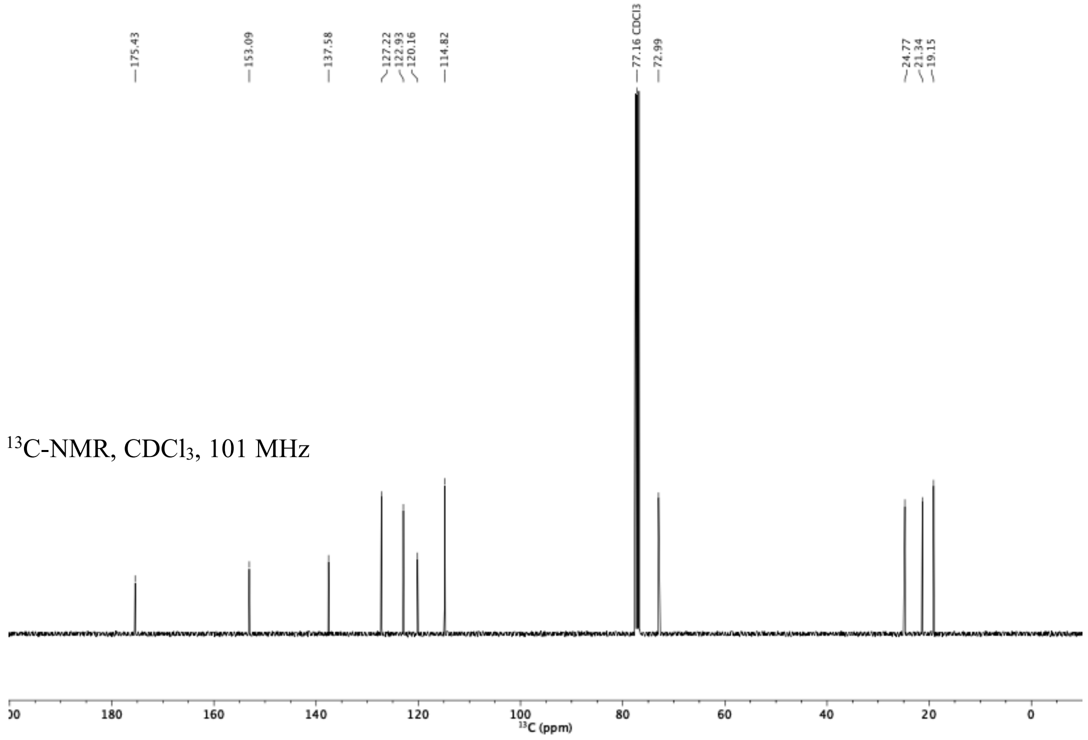

7-Methylchromane-2-carboxylic acid (*rac*-**S-3k**)

^{1}H-NMR, $CDCl_3$, 400 MHz

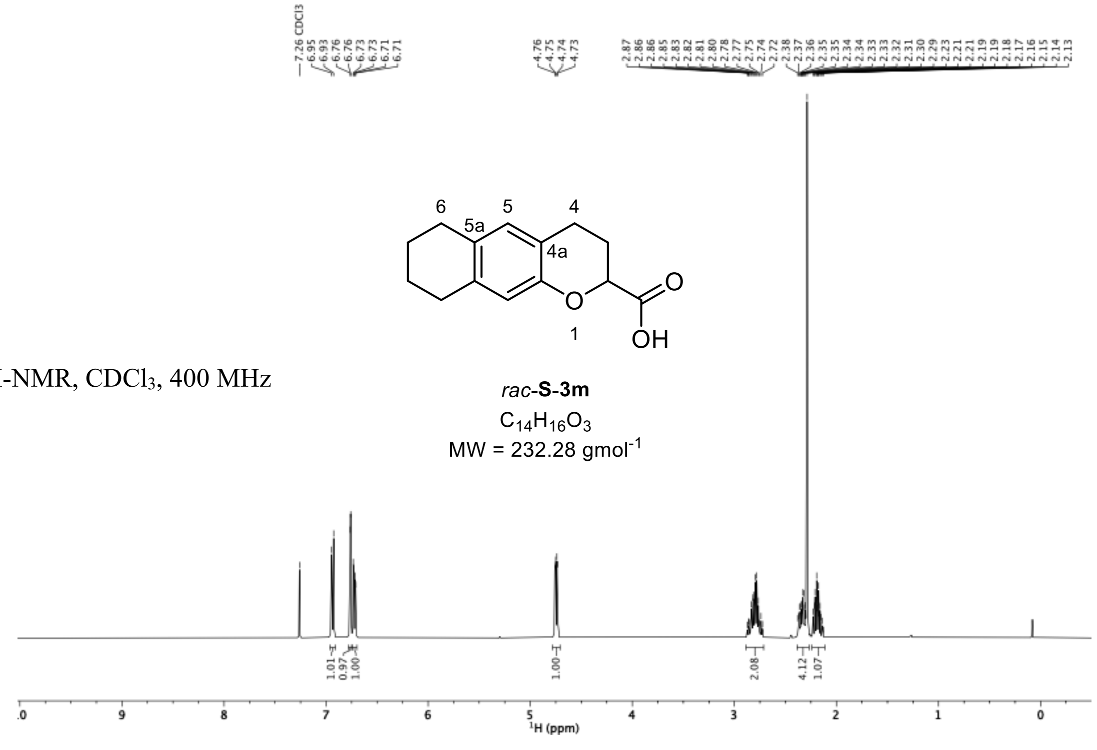

^{13}C-NMR, $CDCl_3$, 101 MHz

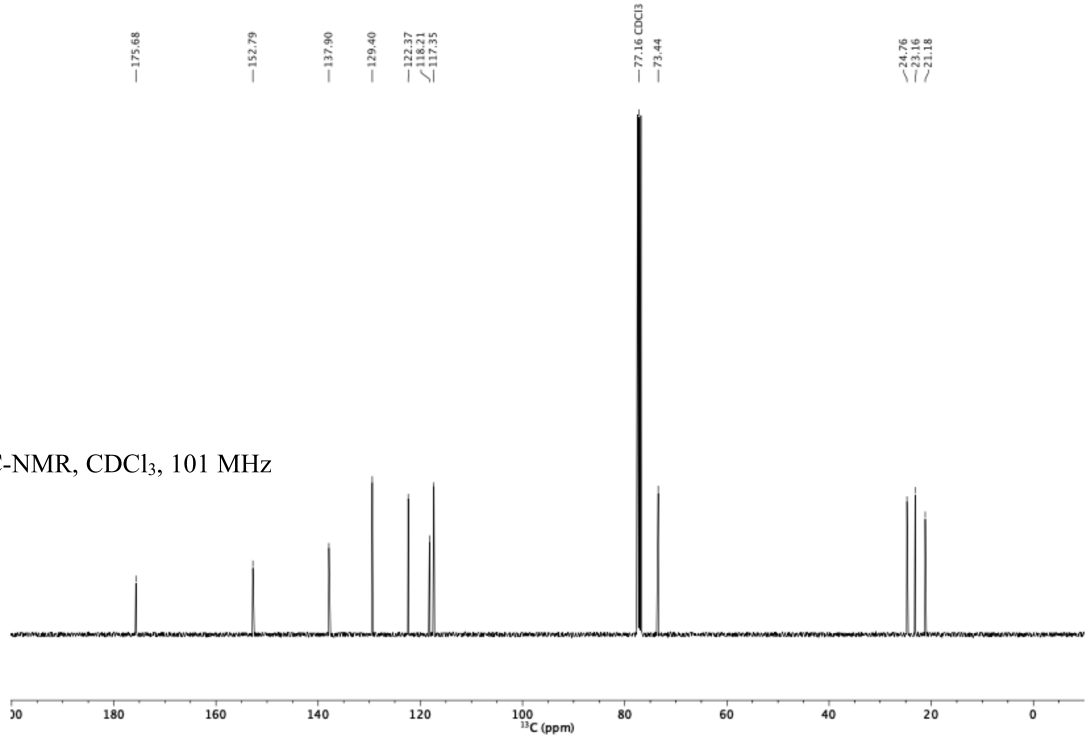

6,7,8,9-Tetrahydro-benzo[*g*]chromane-2-carboxylic acid (*rac*-**S-3m**)

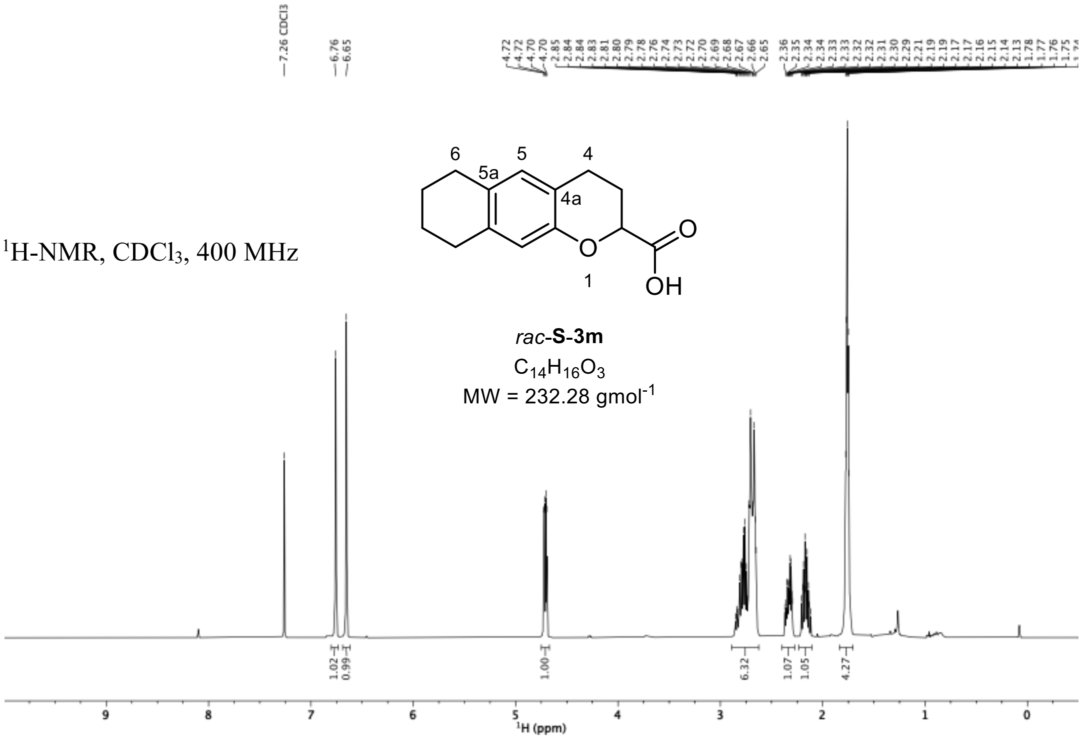

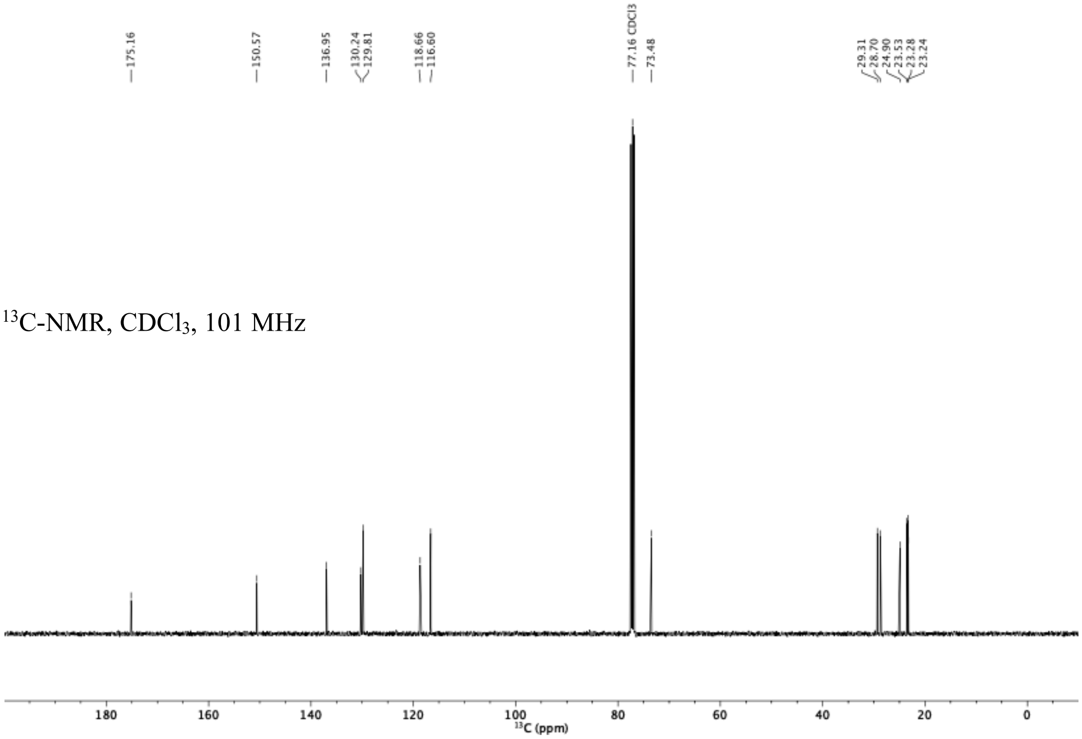

8-Fluorochromane-2-carboxylic acid (*rac*-S-3q)

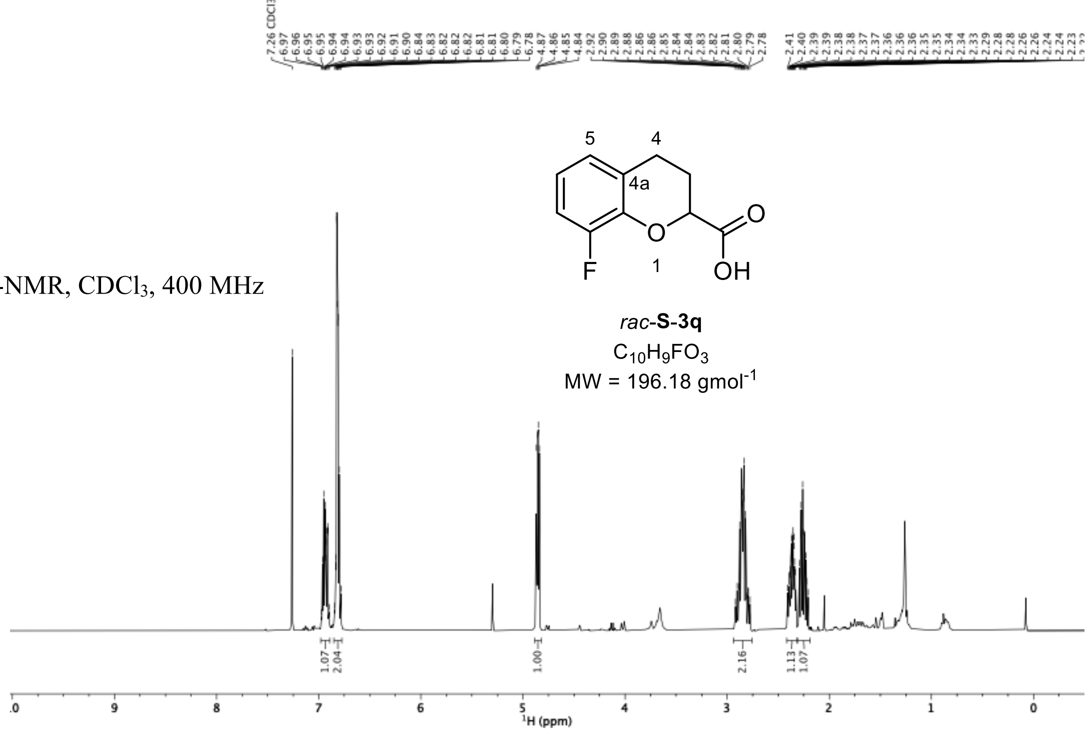

rac-**S**-**3q**

$C_{10}H_9FO_3$

MW = 196.18 $gmol^{-1}$

^{1}H-NMR, $CDCl_3$, 400 MHz

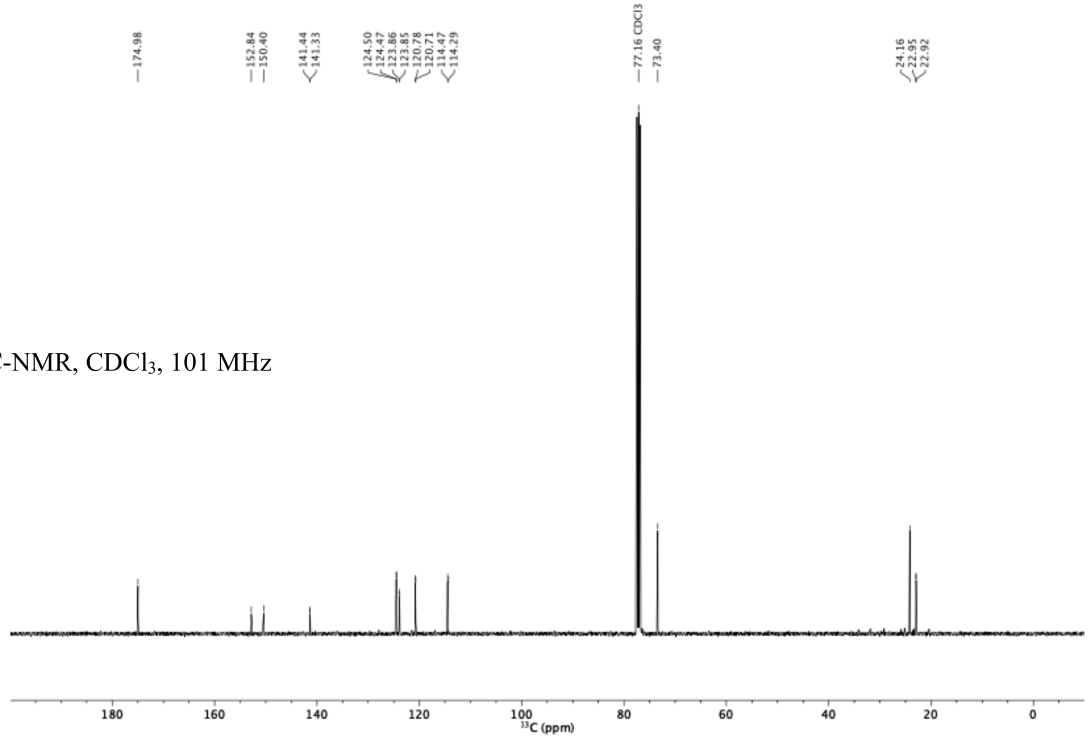

^{13}C-NMR, $CDCl_3$, 101 MHz

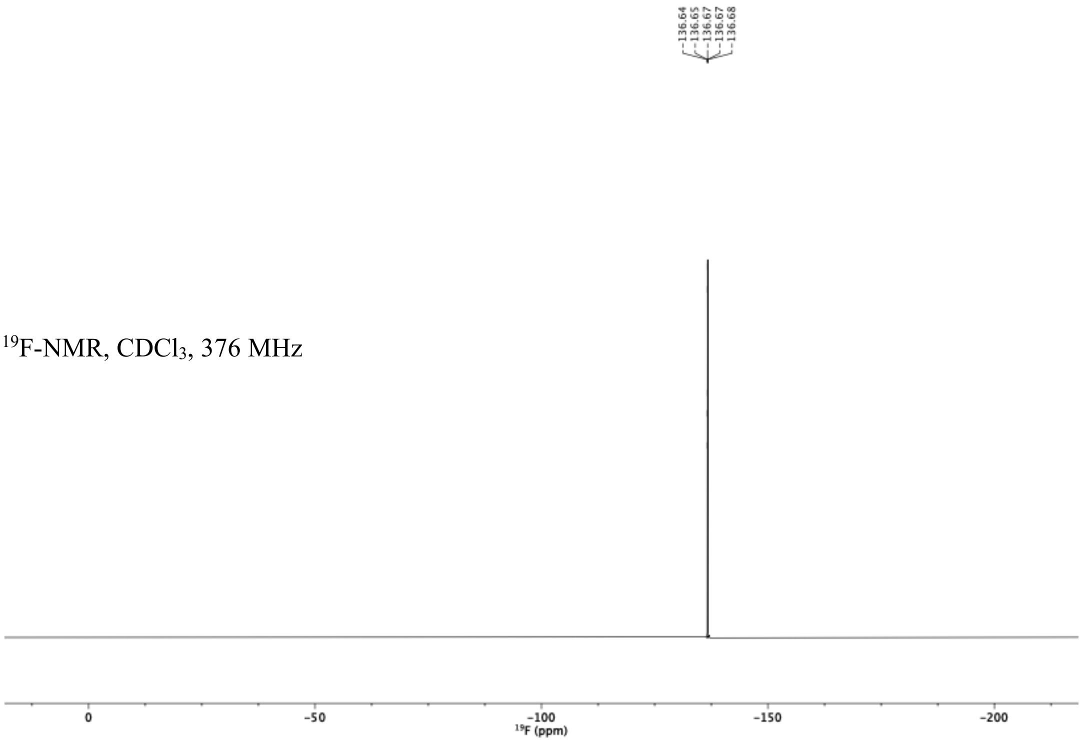

^{19}F-NMR, $CDCl_3$, 376 MHz

3,3-Dimethyl-1,4-benzodioxane-2-carboxylic acid (*rac*-S-3s)

^{1}H-NMR, $CDCl_3$, 400 MHz

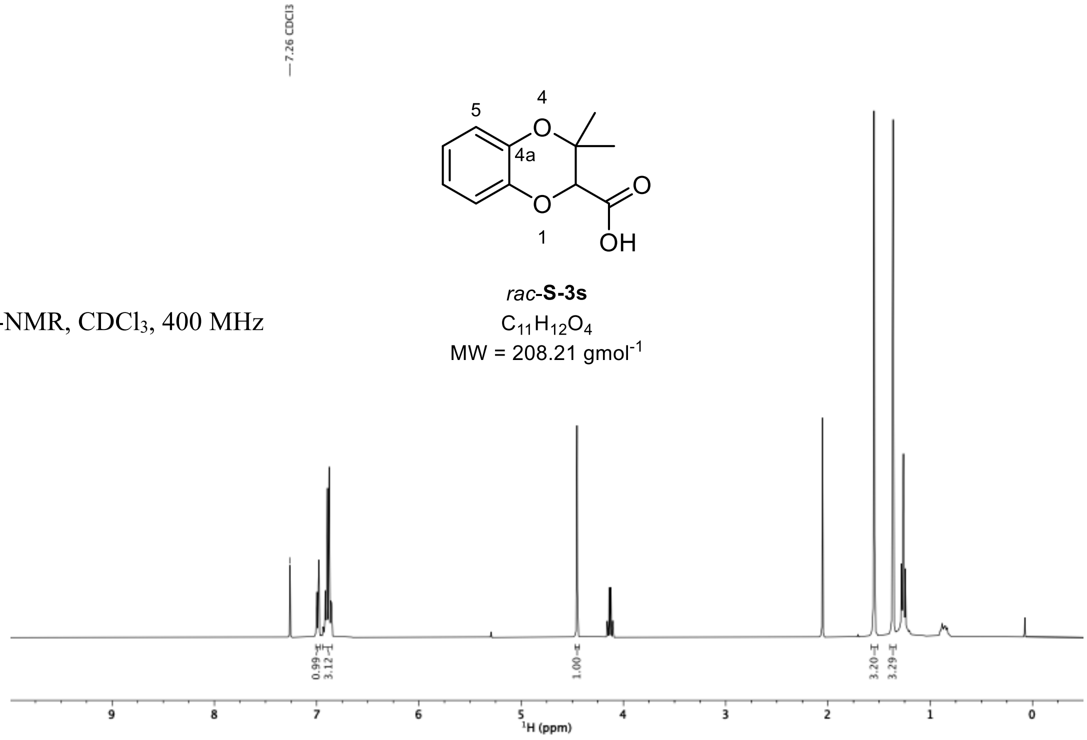

^{13}C-NMR, $CDCl_3$, 101 MHz

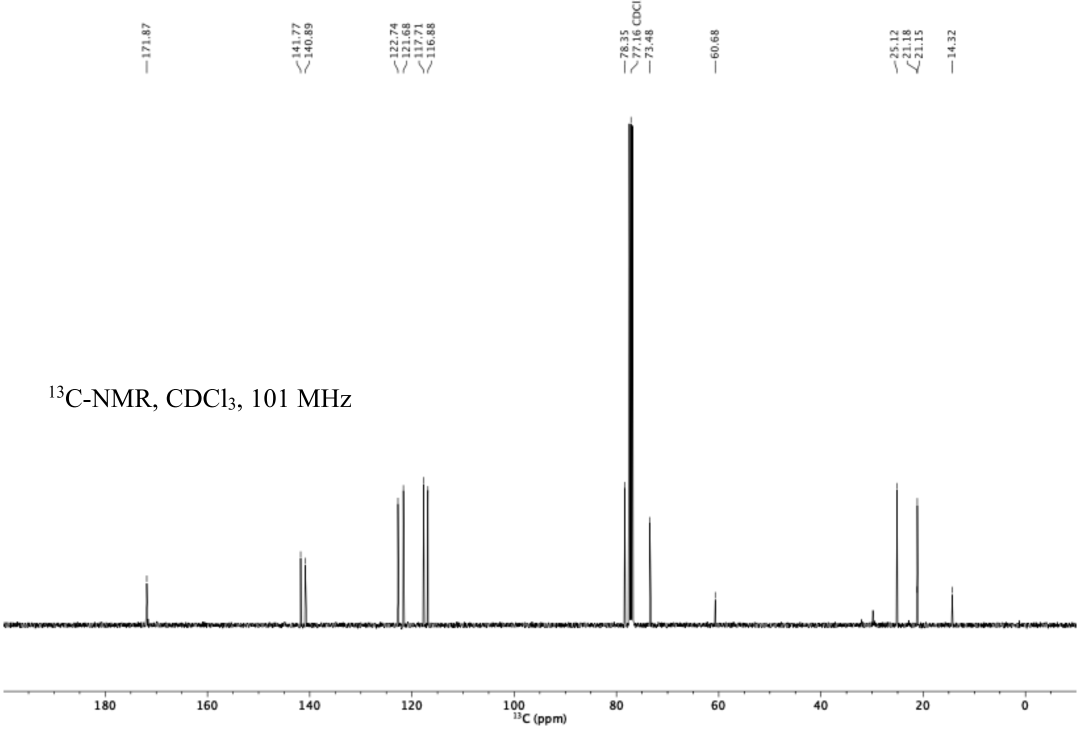

3-Methyl-1,4-benzodioxane-2-carboxylic acid (*rac*-S-3v)

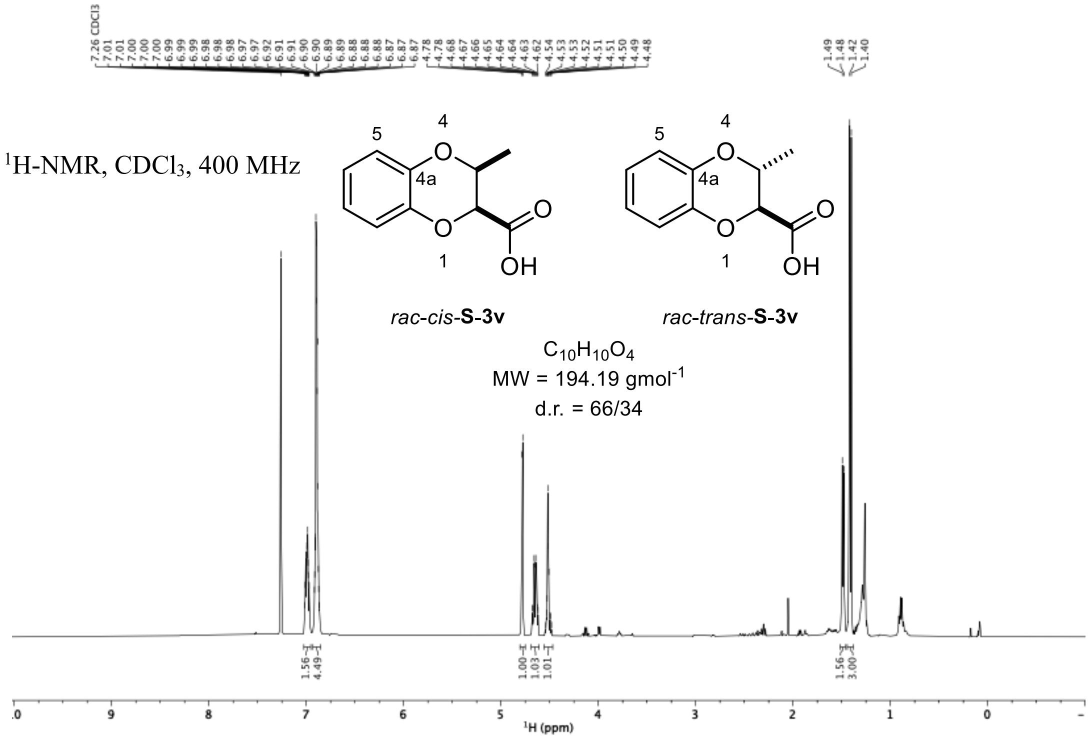

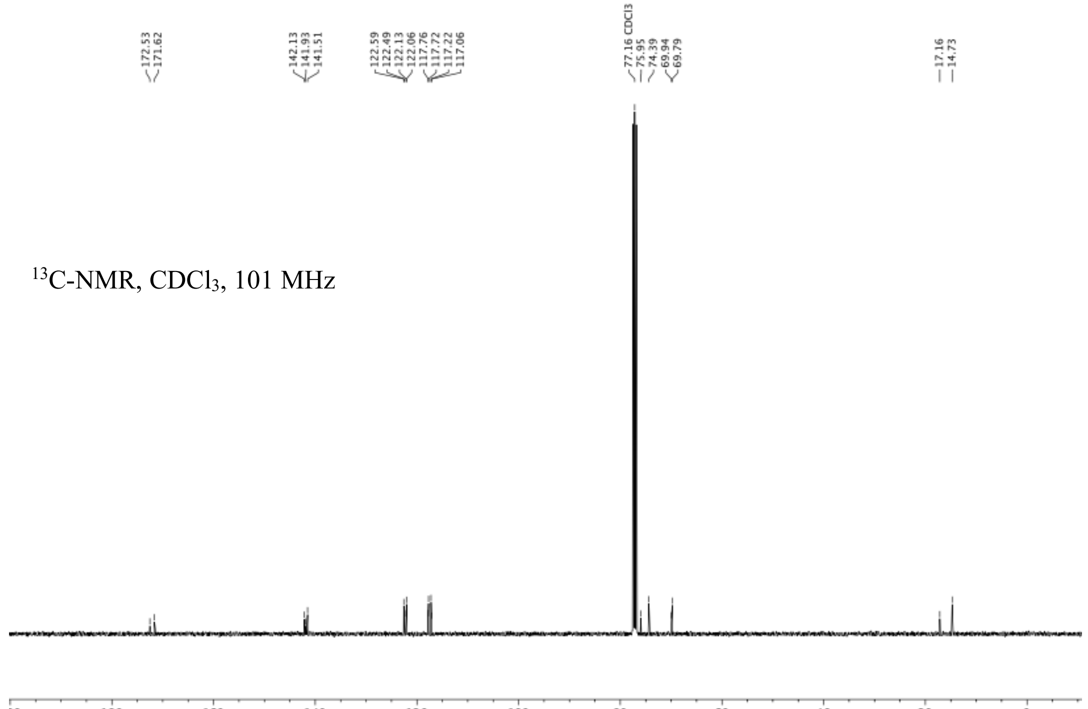

4-Oxothiochromane-2-carboxylic acid (*rac*-**S-5**)

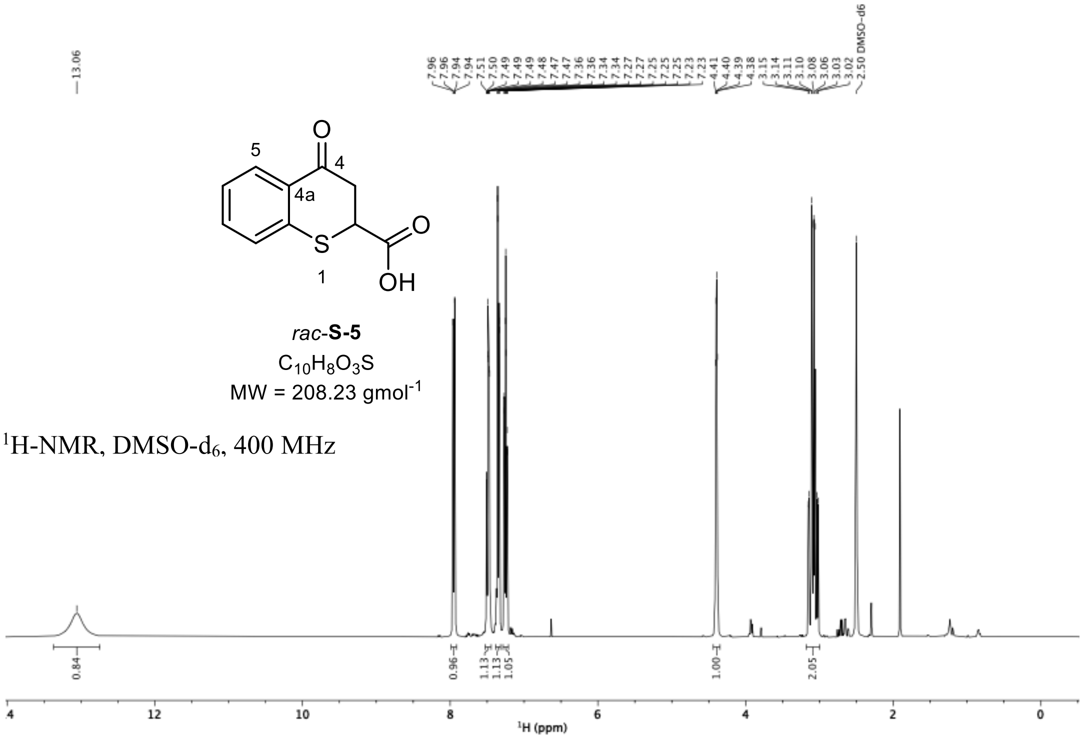

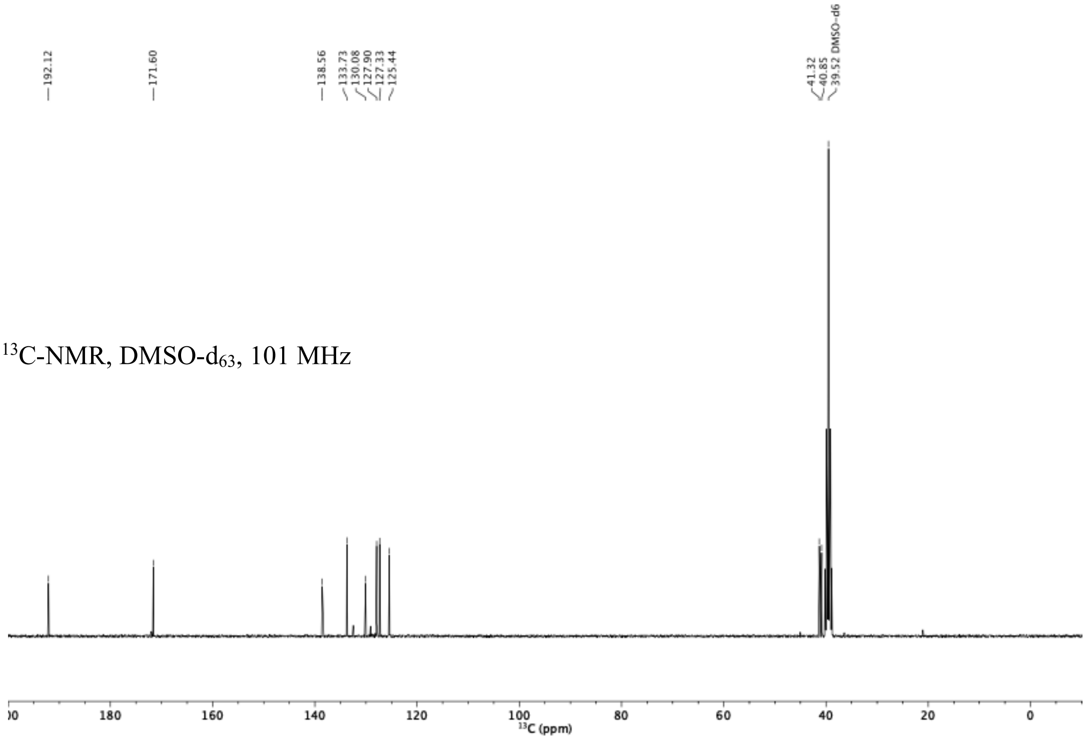

Chromane-2-carboxamide (*rac*-**1a**)

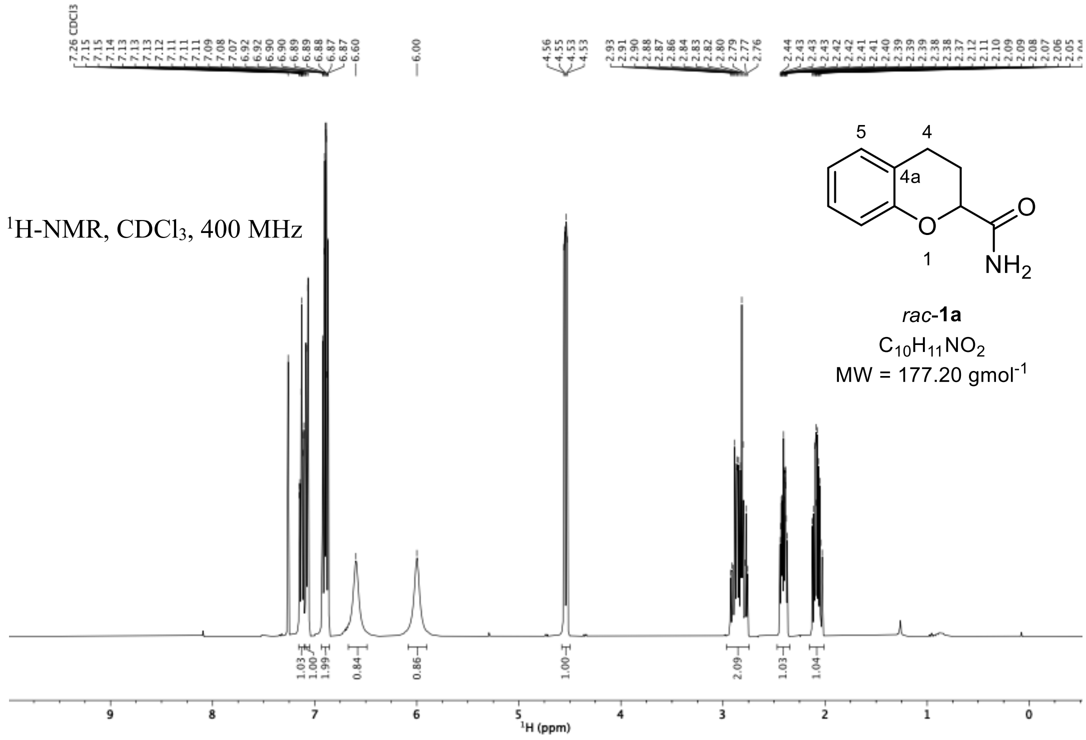

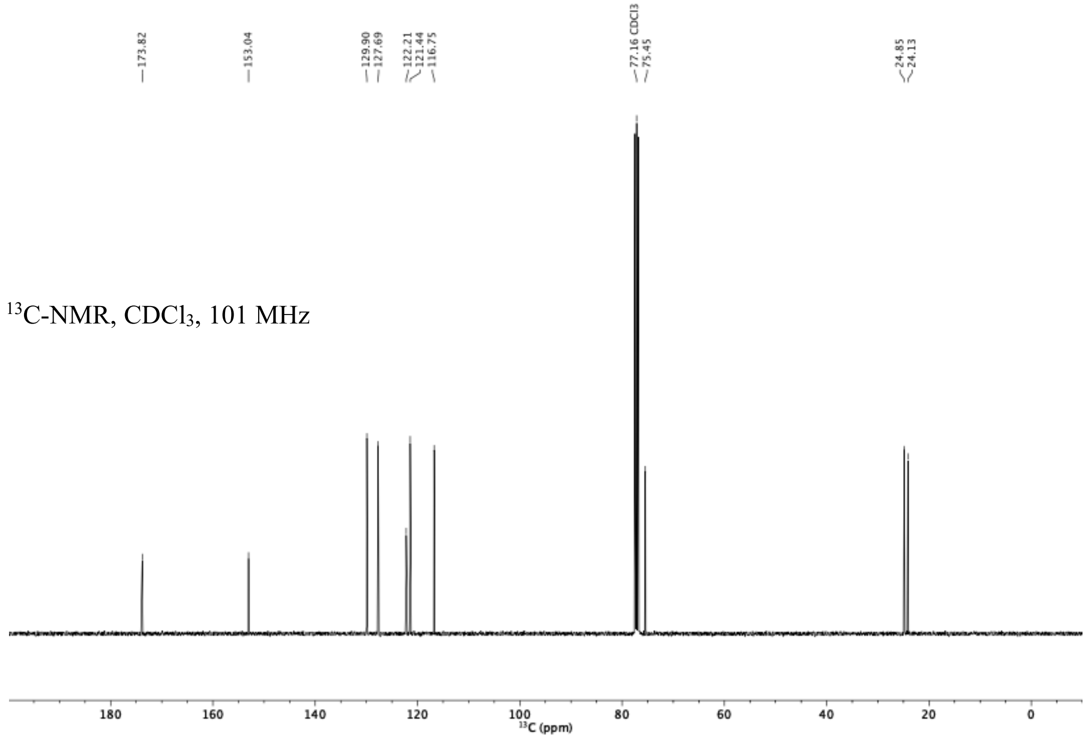

6-Fluorochromane-2-carboxamide (*rac*-**1b**)

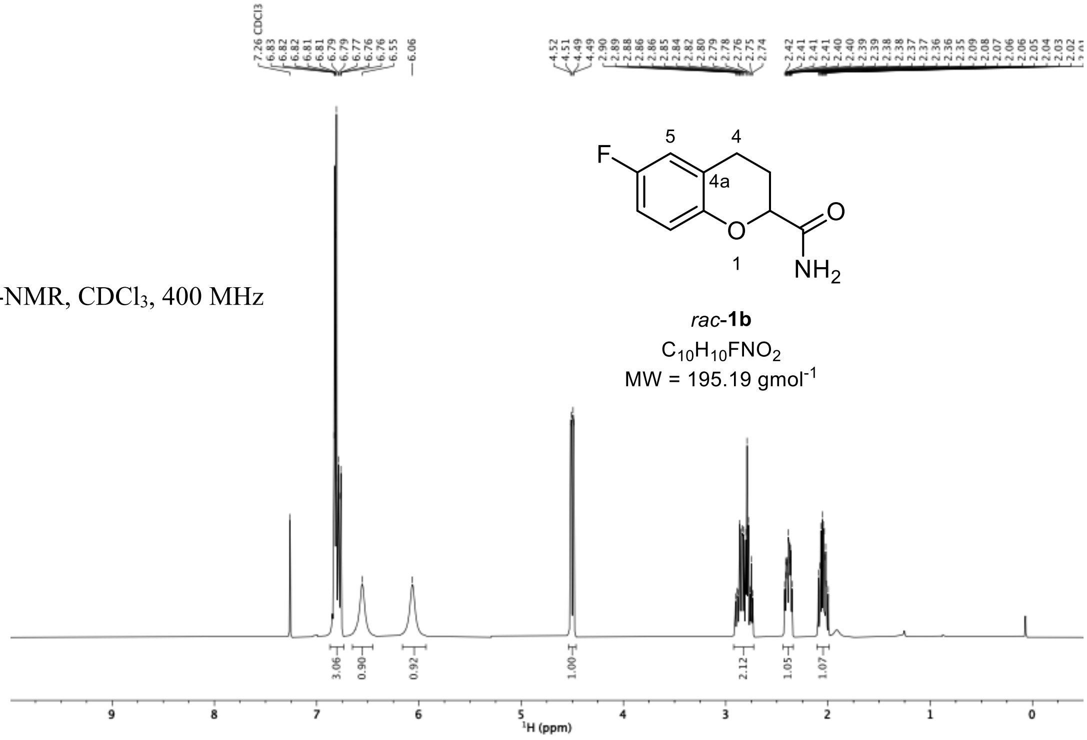

^{1}H-NMR, $CDCl_3$, 400 MHz

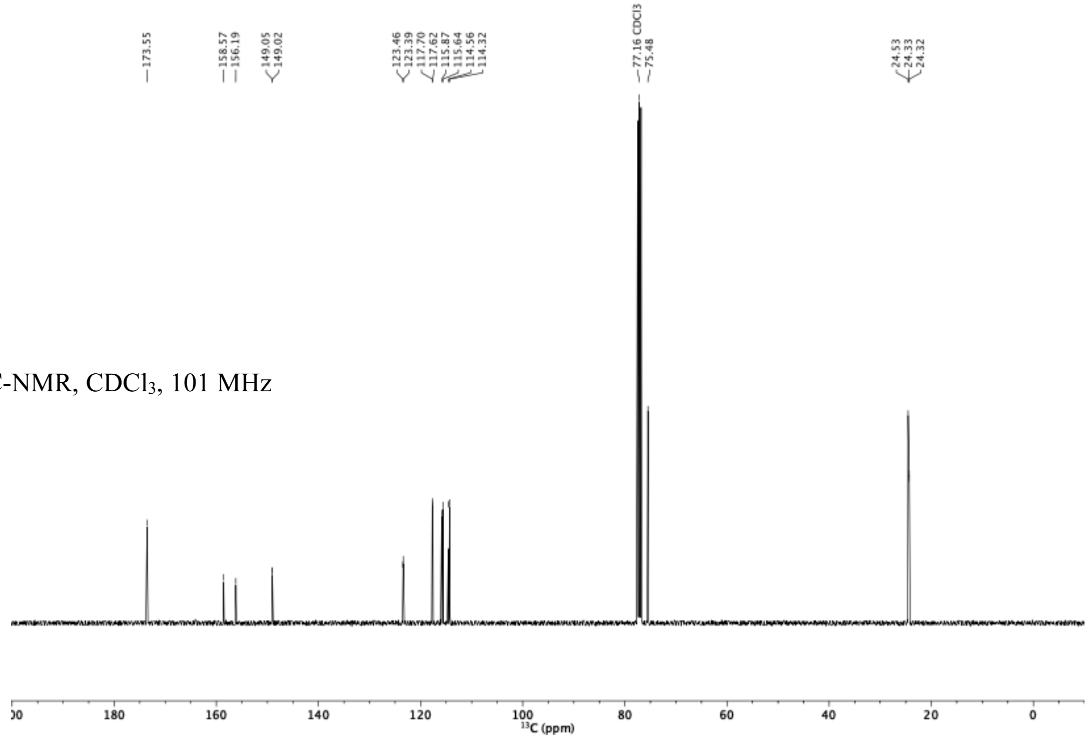

^{13}C-NMR, $CDCl_3$, 101 MHz

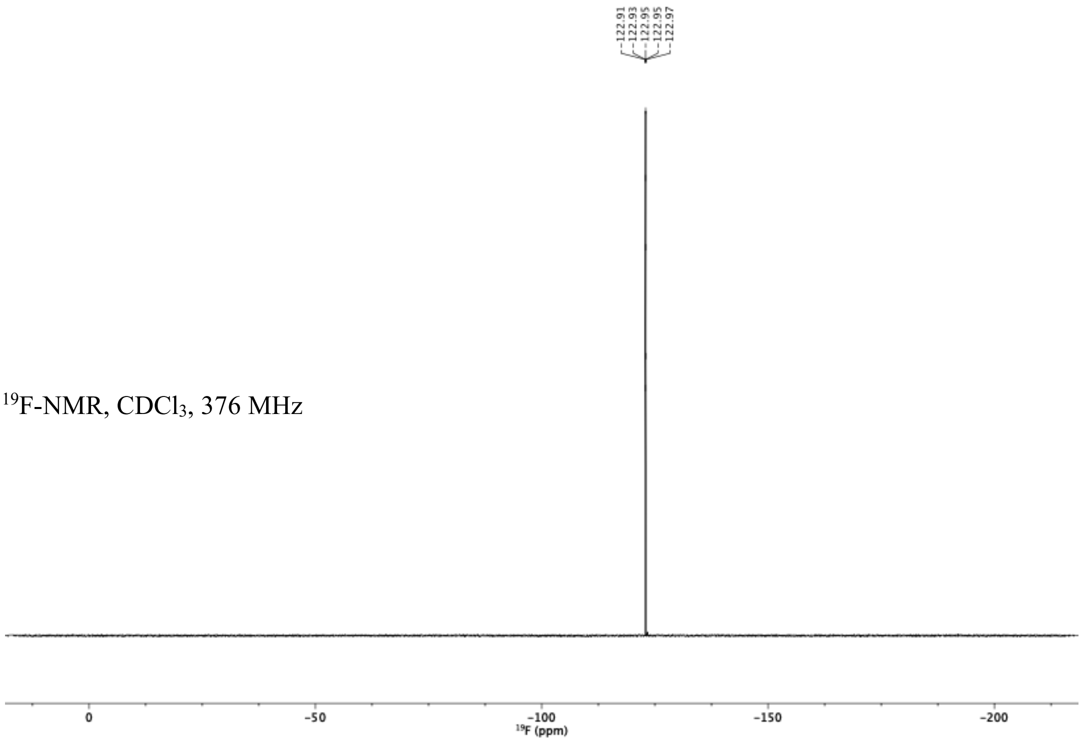

^{19}F-NMR, $CDCl_3$, 376 MHz

6-Chlorochromane-2-carboxamide (*rac*-1c)

^{1}H-NMR, $CDCl_3$, 400 MHz

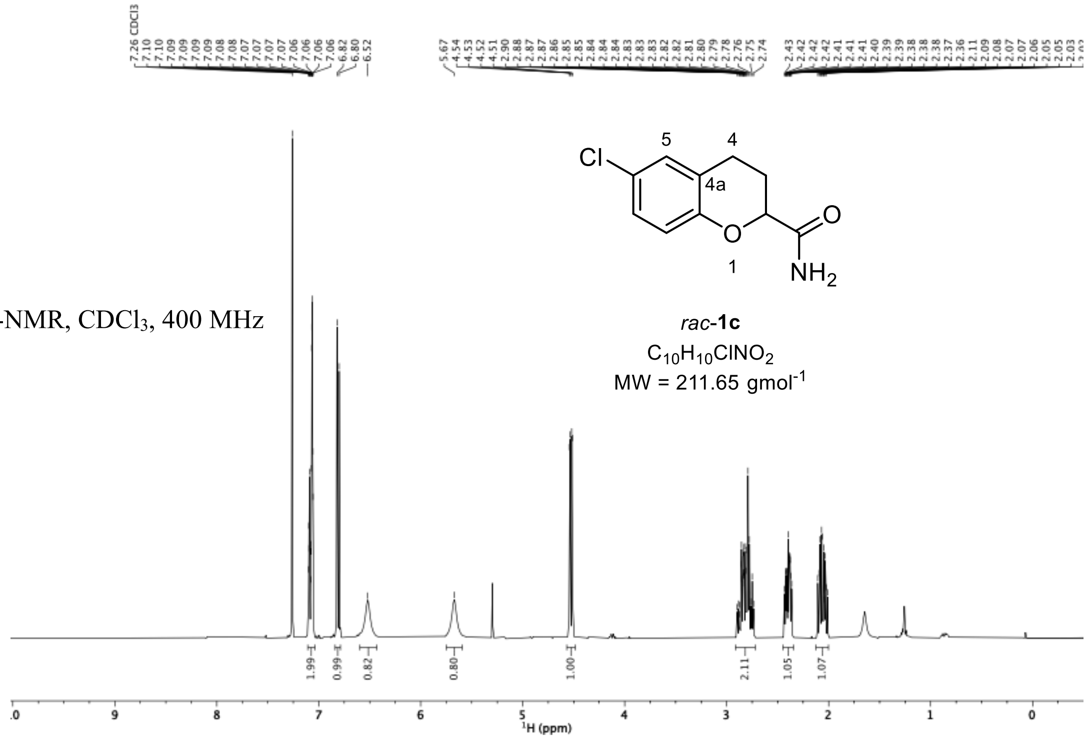

^{13}C-NMR, $CDCl_3$, 101 MHz

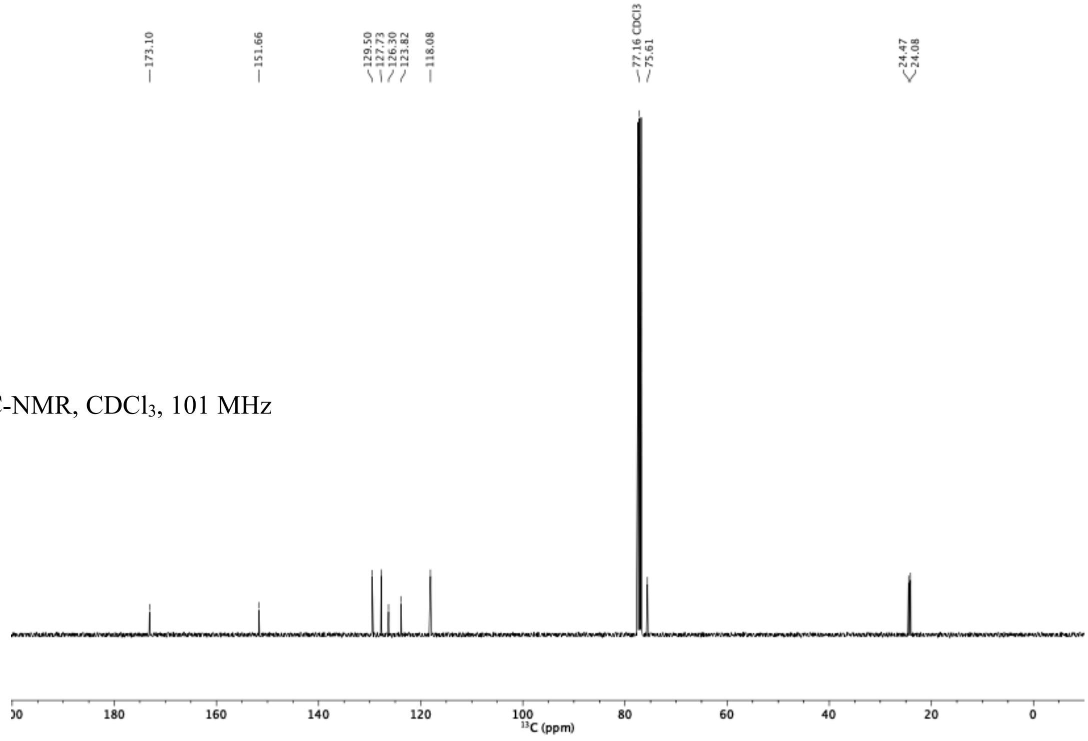

6-Bromochromane-2-carboxamide (*rac*-1d)

^{1}H-NMR, $CDCl_3$, 400 MHz

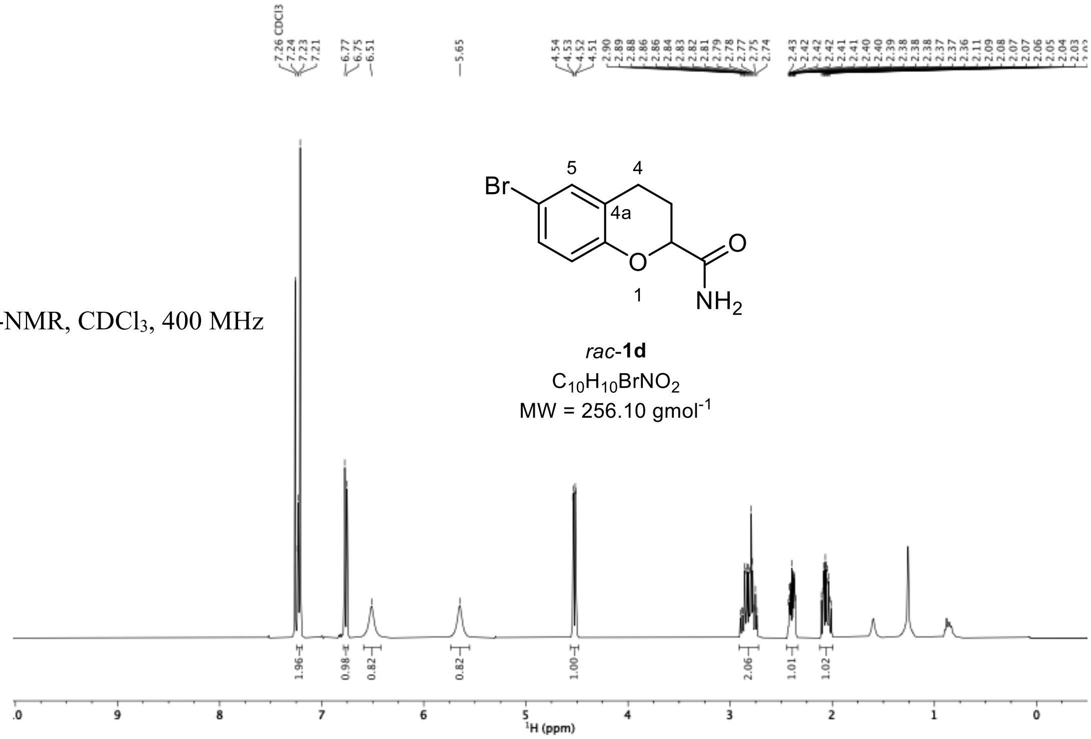

^{13}C-NMR, $CDCl_3$, 101 MHz

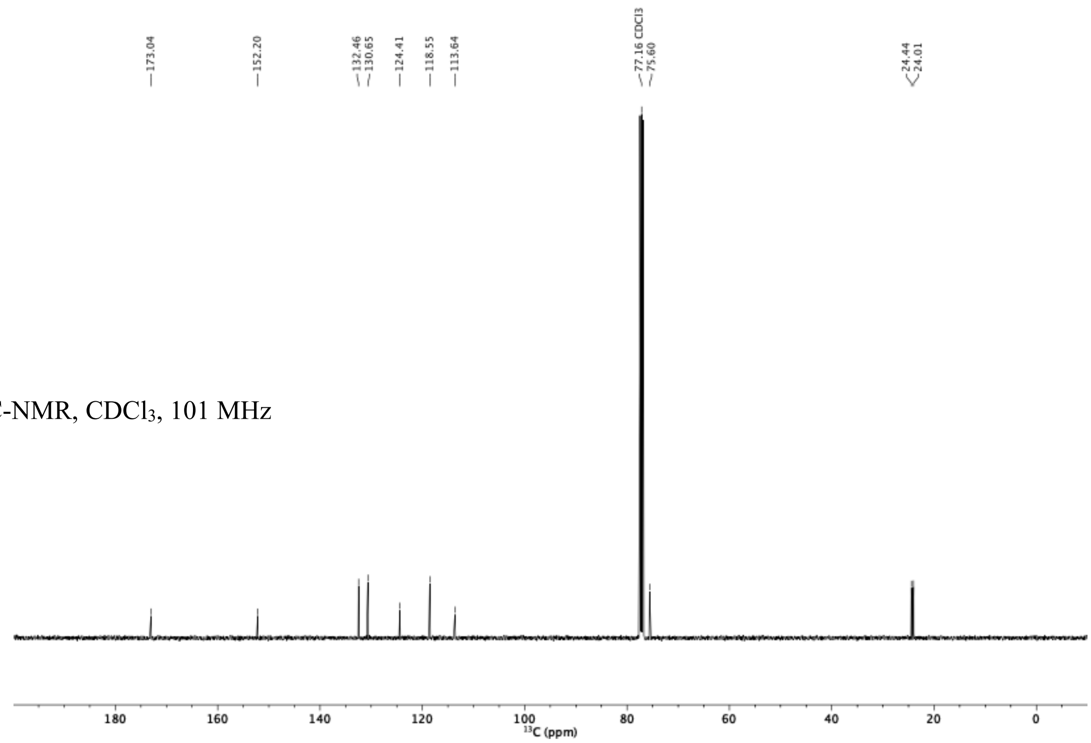

6-Iodochromane-2-carboxamide (*rac*-**1e**)

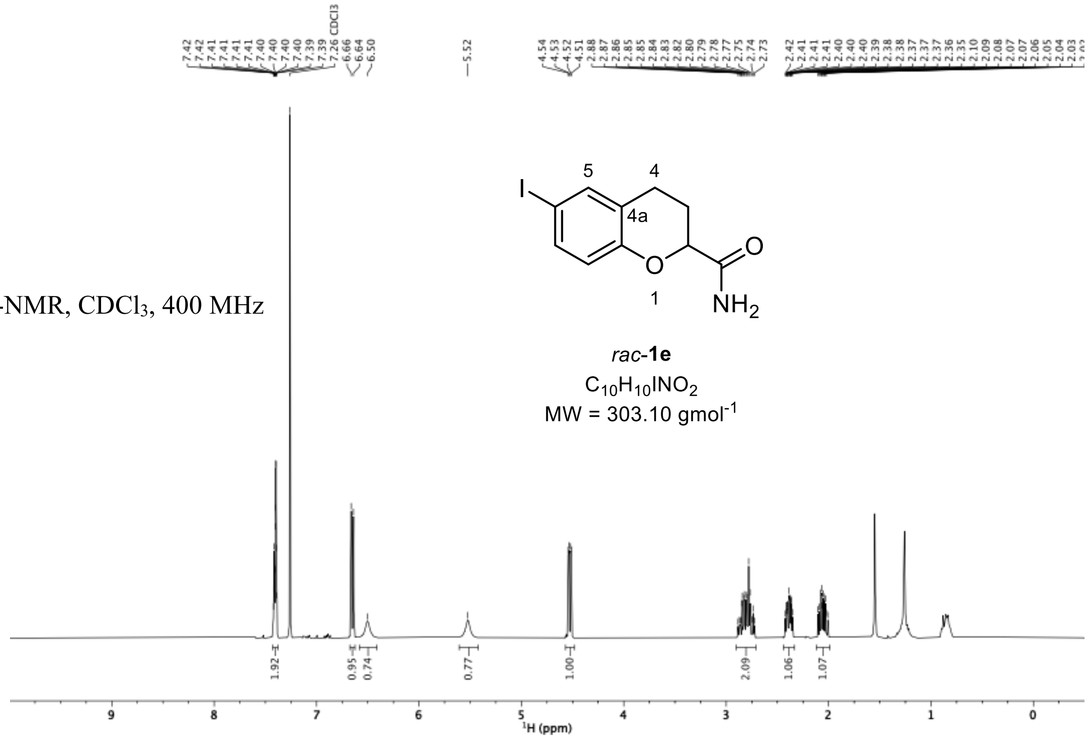

^{1}H-NMR, $CDCl_3$, 400 MHz

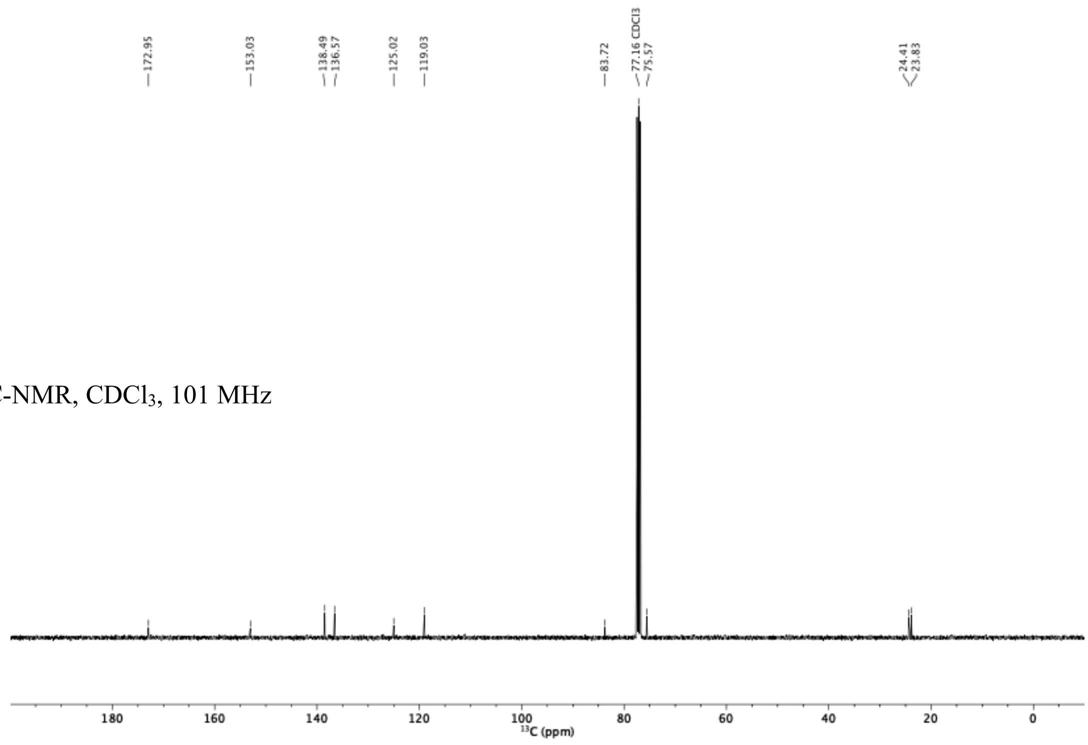

^{13}C-NMR, $CDCl_3$, 101 MHz

6-Methylchromane-2-carboxamide (*rac*-1f)

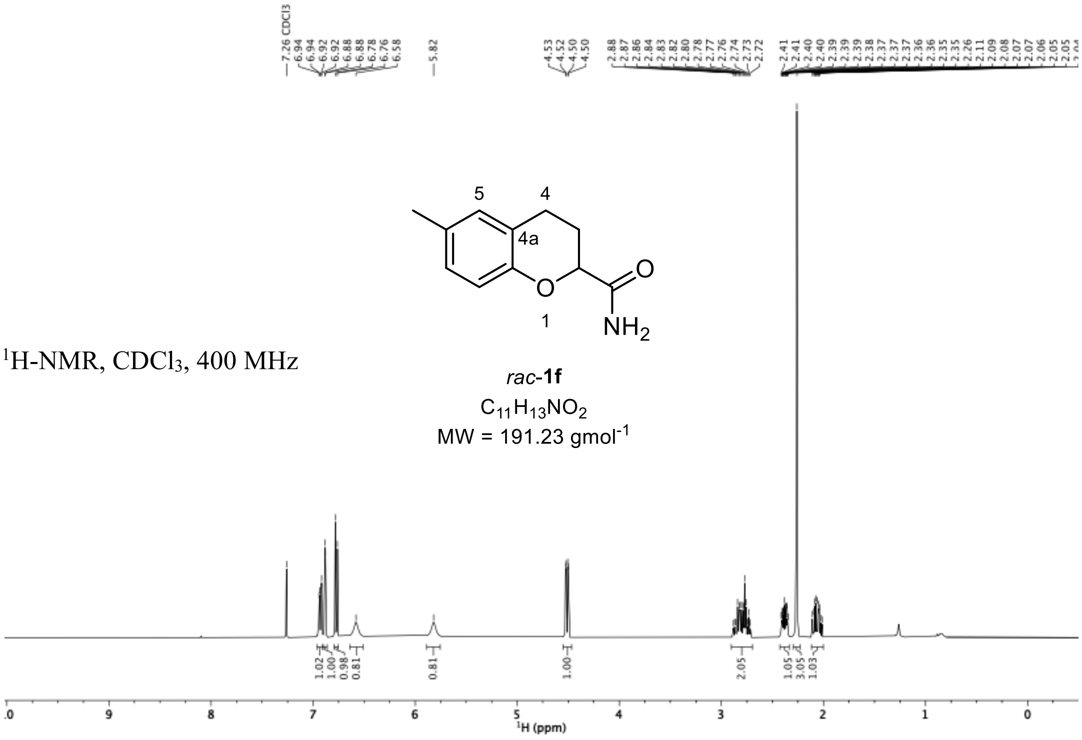

^{1}H-NMR, $CDCl_3$, 400 MHz

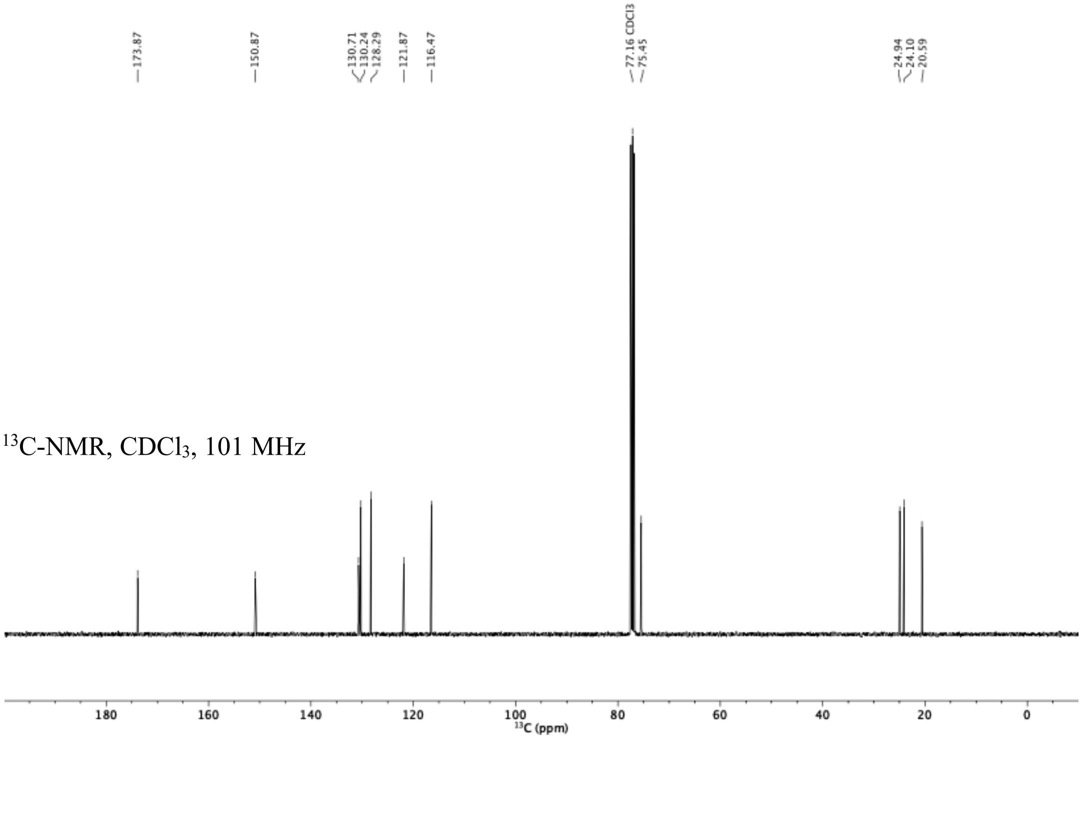

^{13}C-NMR, $CDCl_3$, 101 MHz

6-Methoxychromane-2-carboxamide (*rac*-**1g**)

^{1}H-NMR, $CDCl_3$, 400 MHz

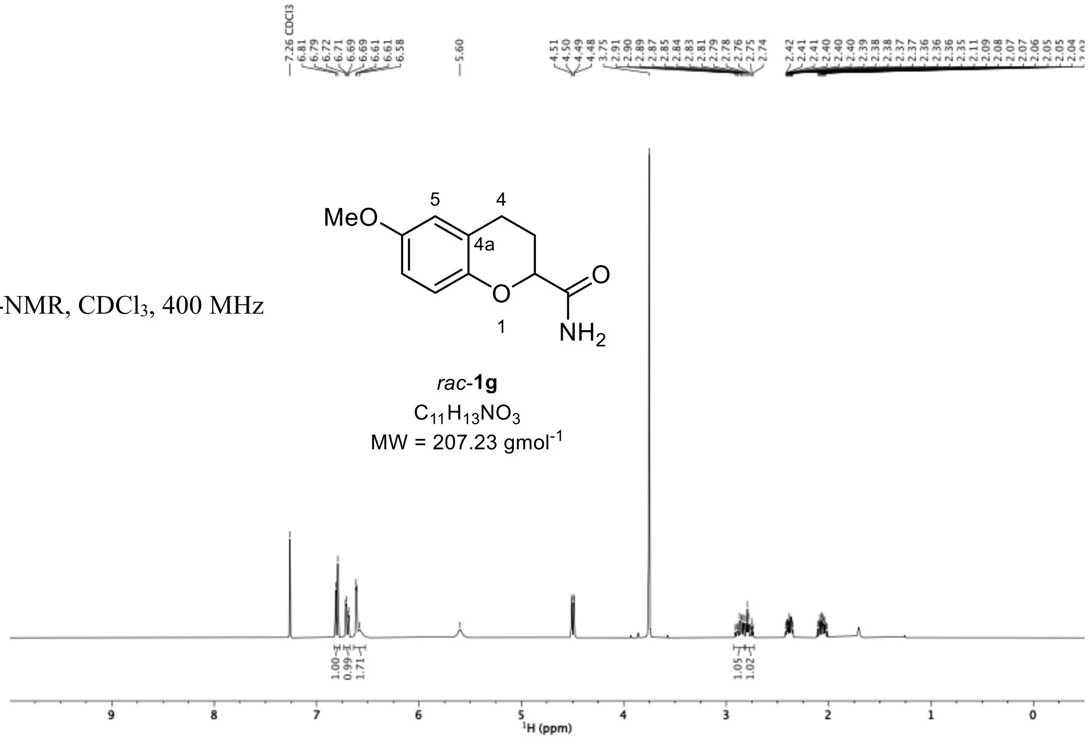

^{13}C-NMR, $CDCl_3$, 101 MHz

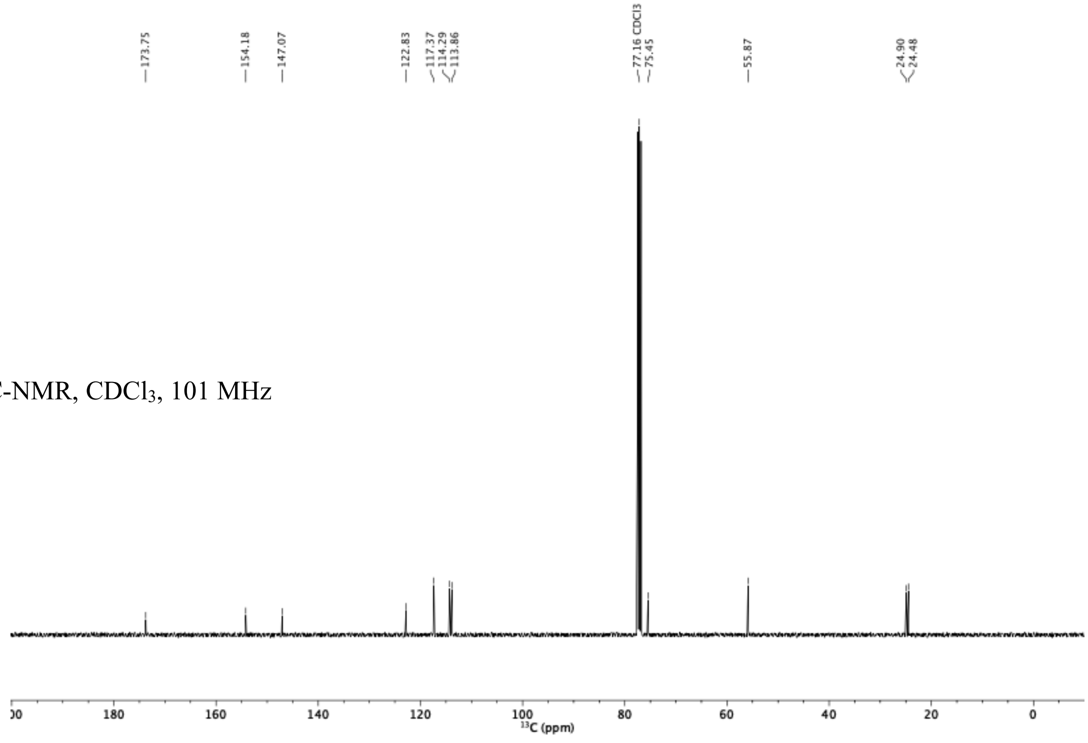

6-Phenylchromane-2-carboxamide (*rac*-**1h**)

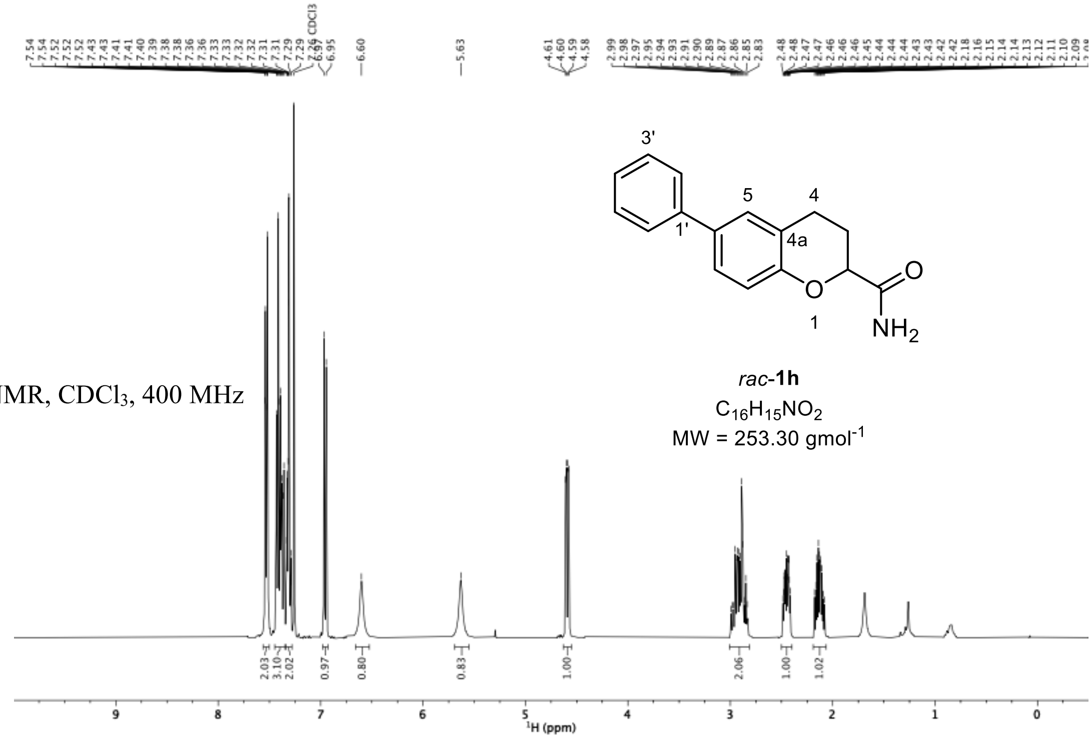

^{1}H-NMR, $CDCl_3$, 400 MHz

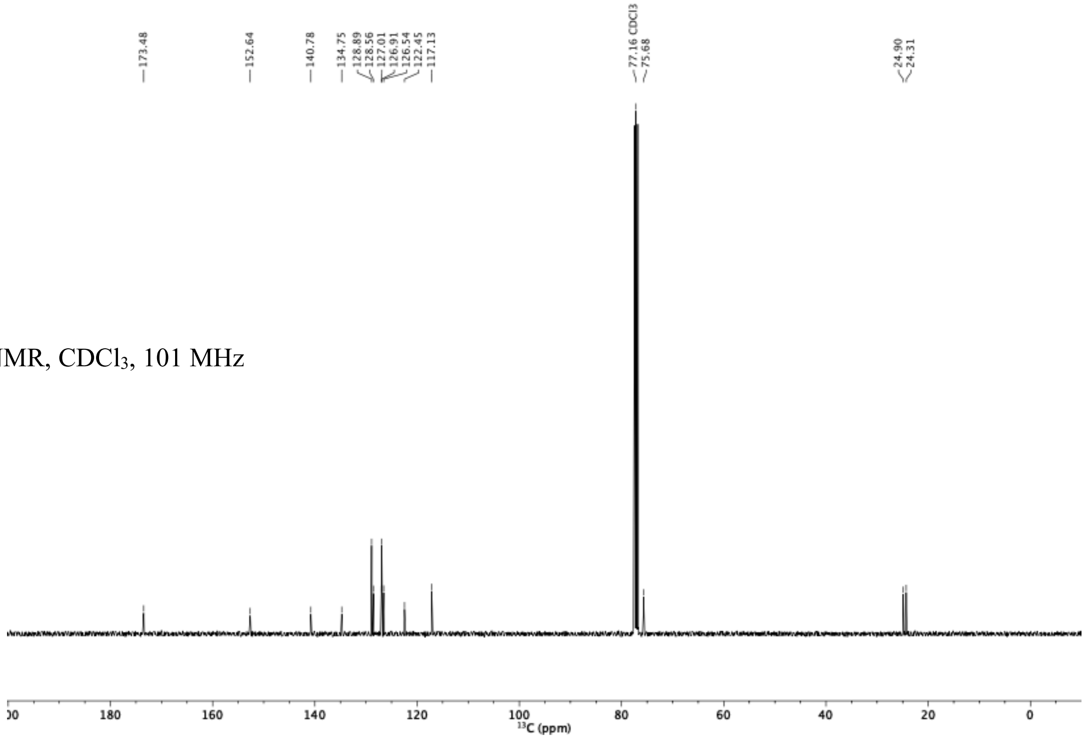

^{13}C-NMR, $CDCl_3$, 101 MHz

6-Ethylchromane-2-carboxamide (*rac*-1i)

^{1}H-NMR, $CDCl_3$, 400 MHz

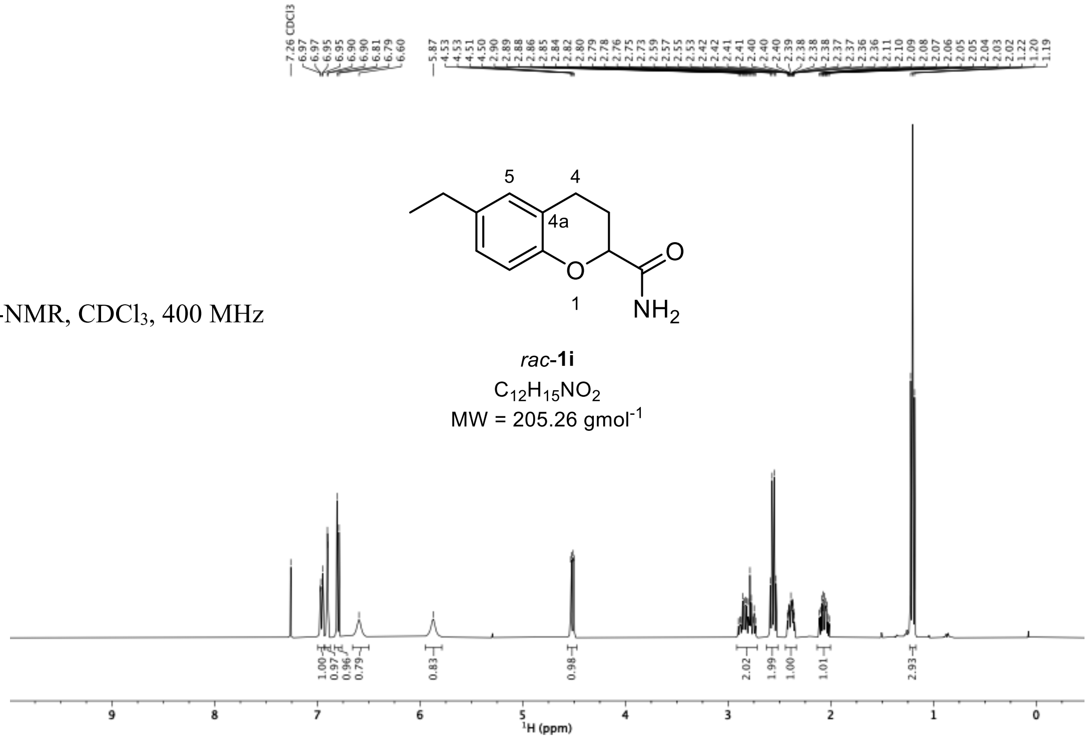

^{13}C-NMR, $CDCl_3$, 101 MHz

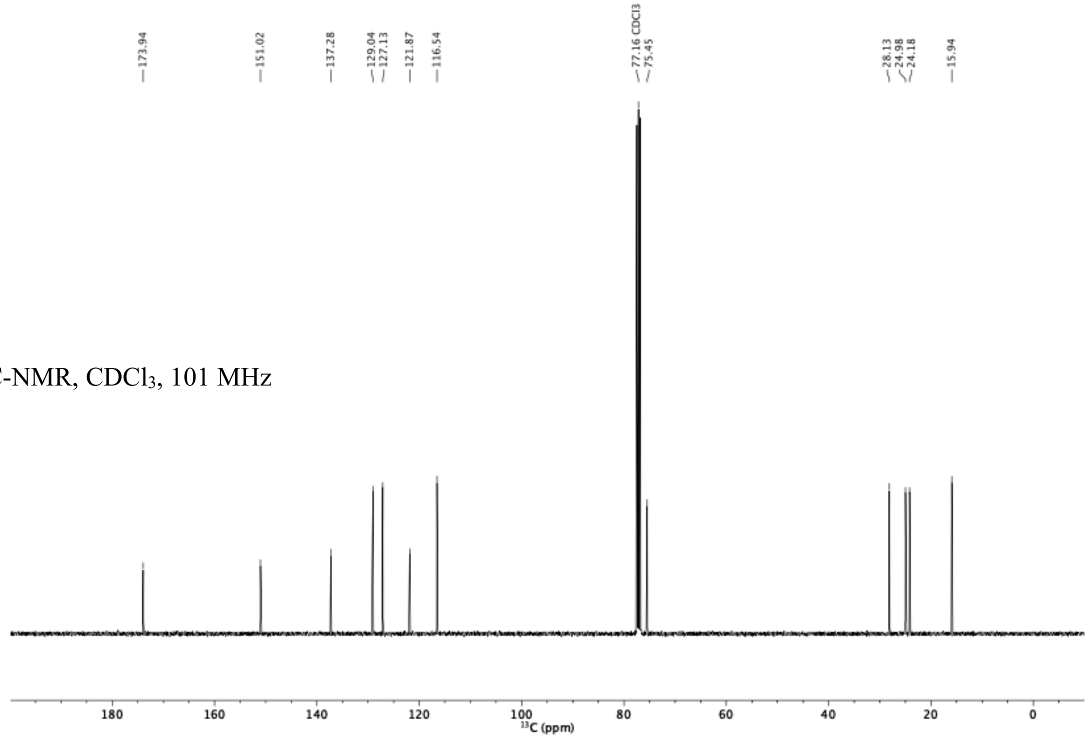

5-Methylchromane-2-carboxamide (*rac*-1j)

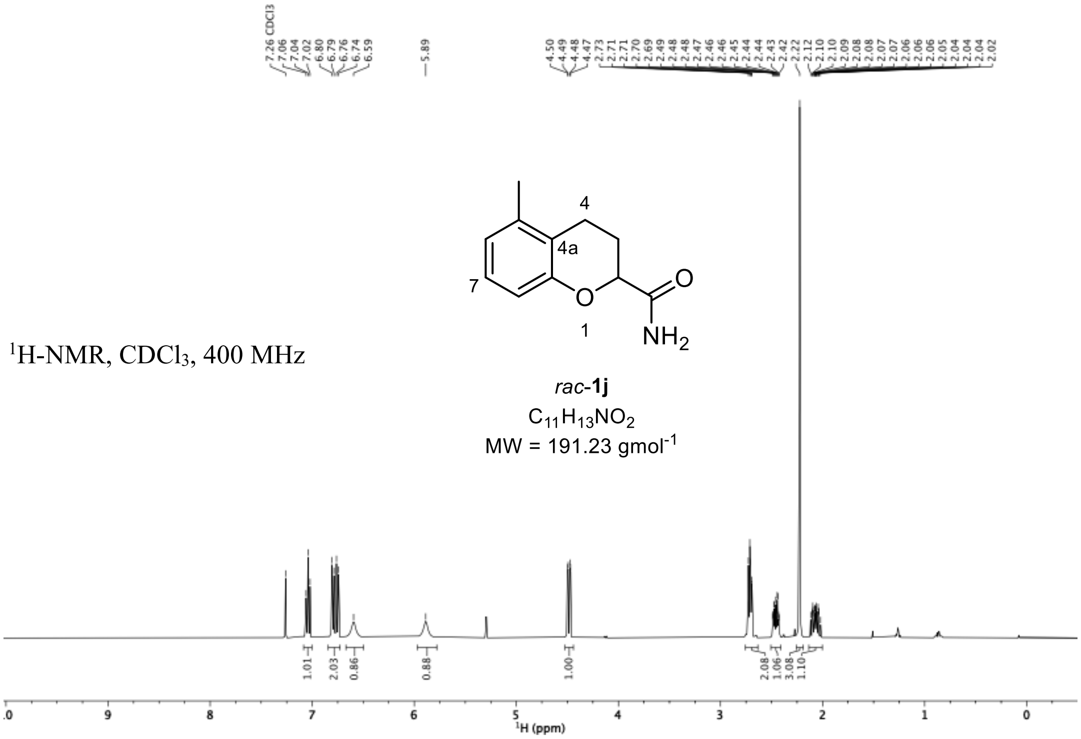

^{1}H-NMR, $CDCl_3$, 400 MHz

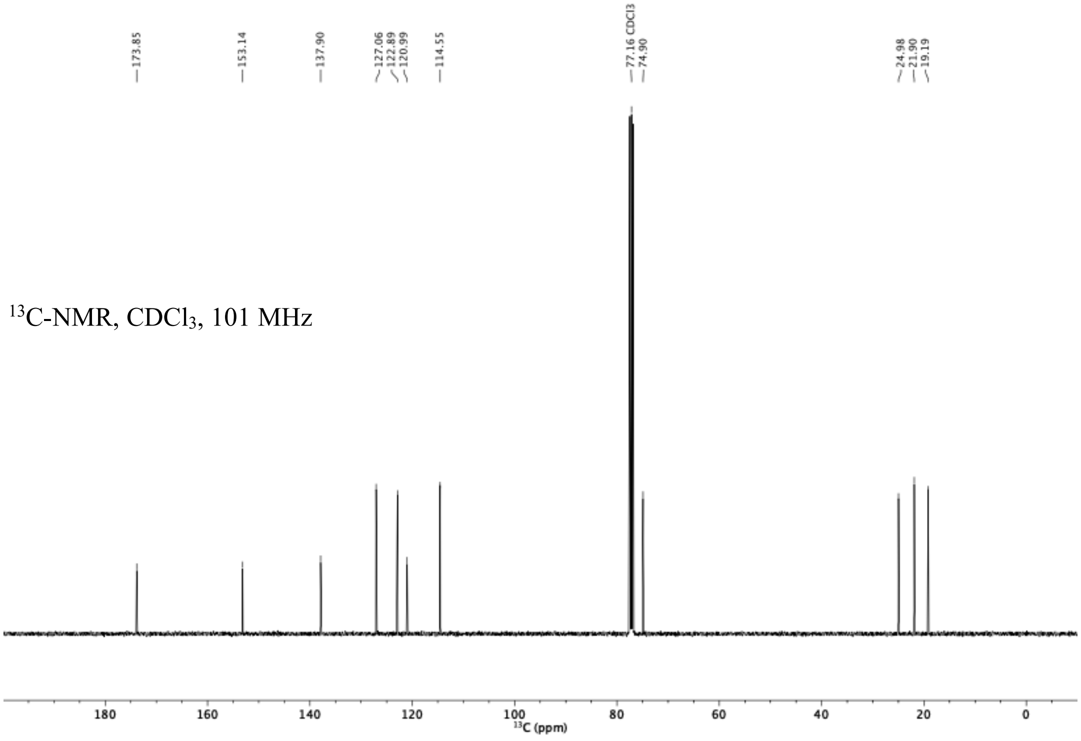

^{13}C-NMR, $CDCl_3$, 101 MHz

7-Methylchromane-2-carboxamide (*rac*-1k)

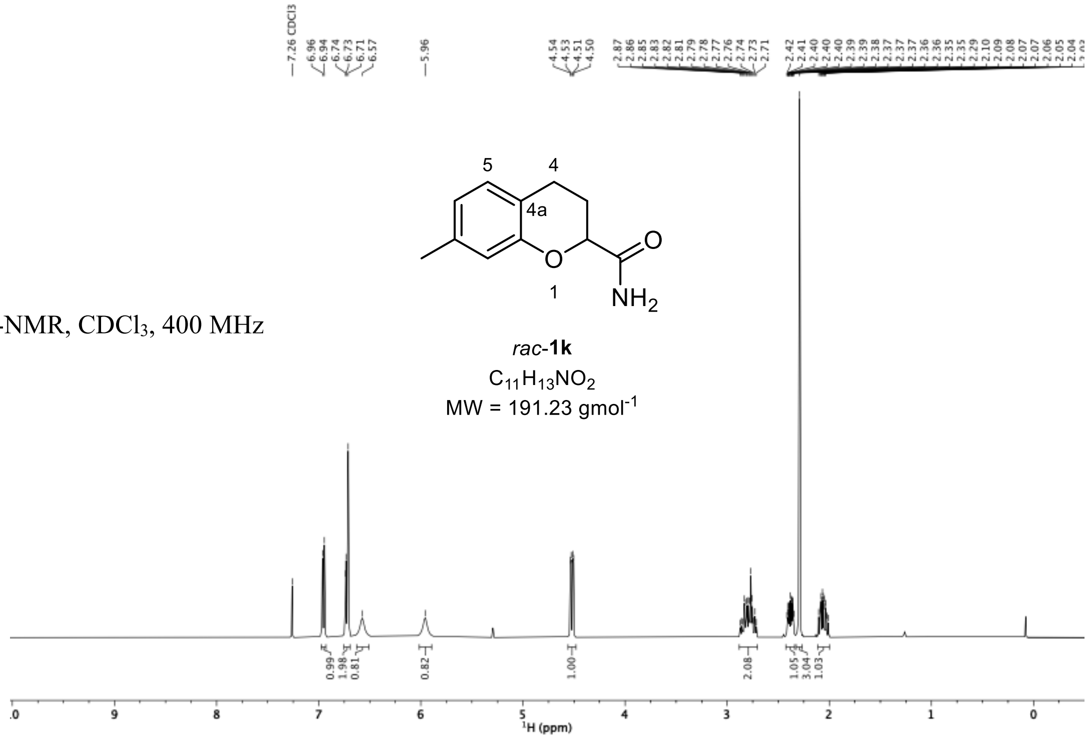

^{1}H-NMR, $CDCl_3$, 400 MHz

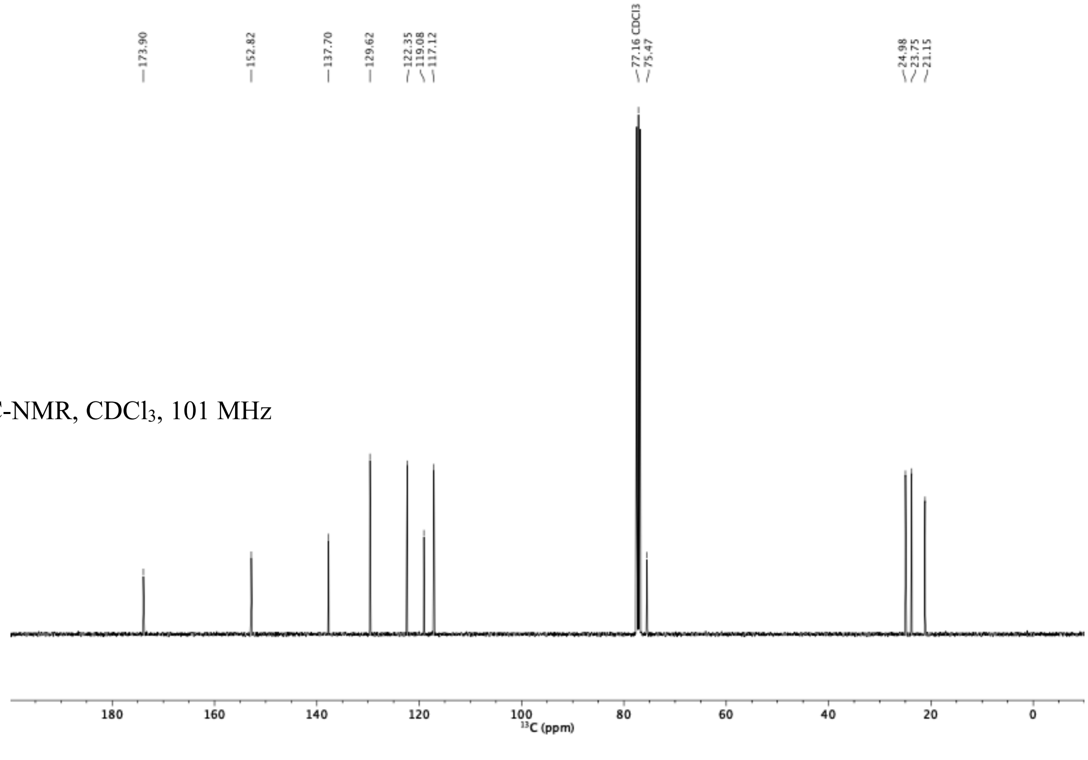

^{13}C-NMR, $CDCl_3$, 101 MHz

8-Methylchromane-2-carboxamide (*rac*-1l)

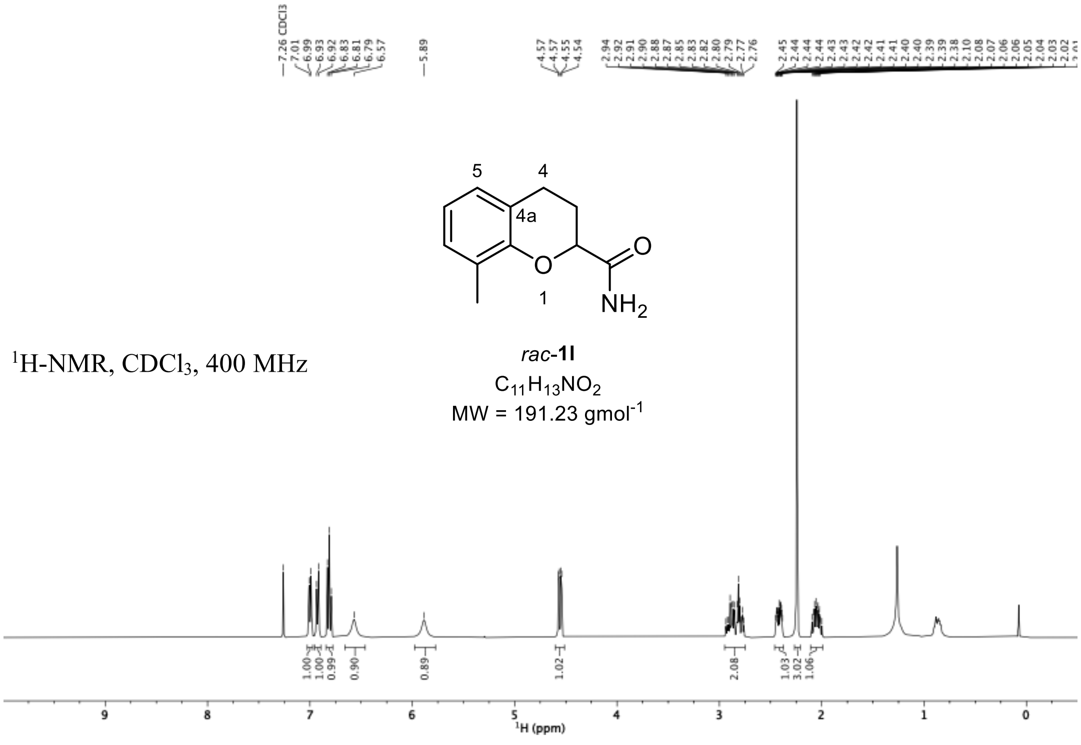

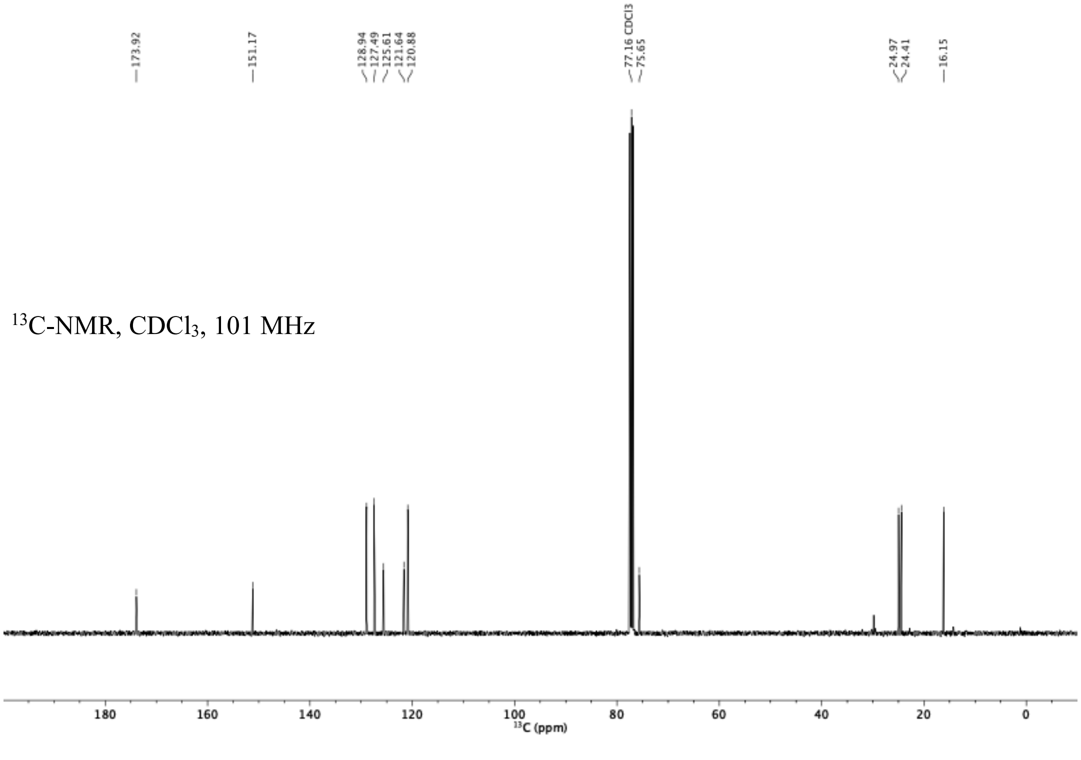

6,7,8,9-Tetrahydro-benzo[*g*]chromane-2-carboxamid (*rac*-1m)

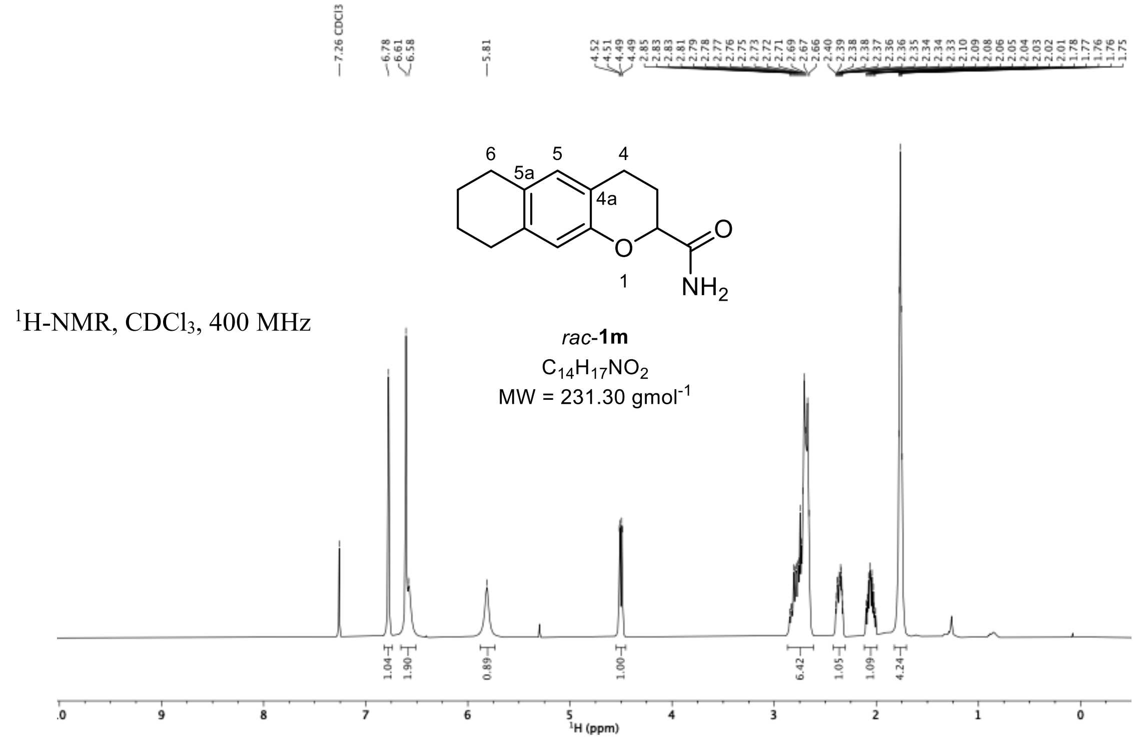

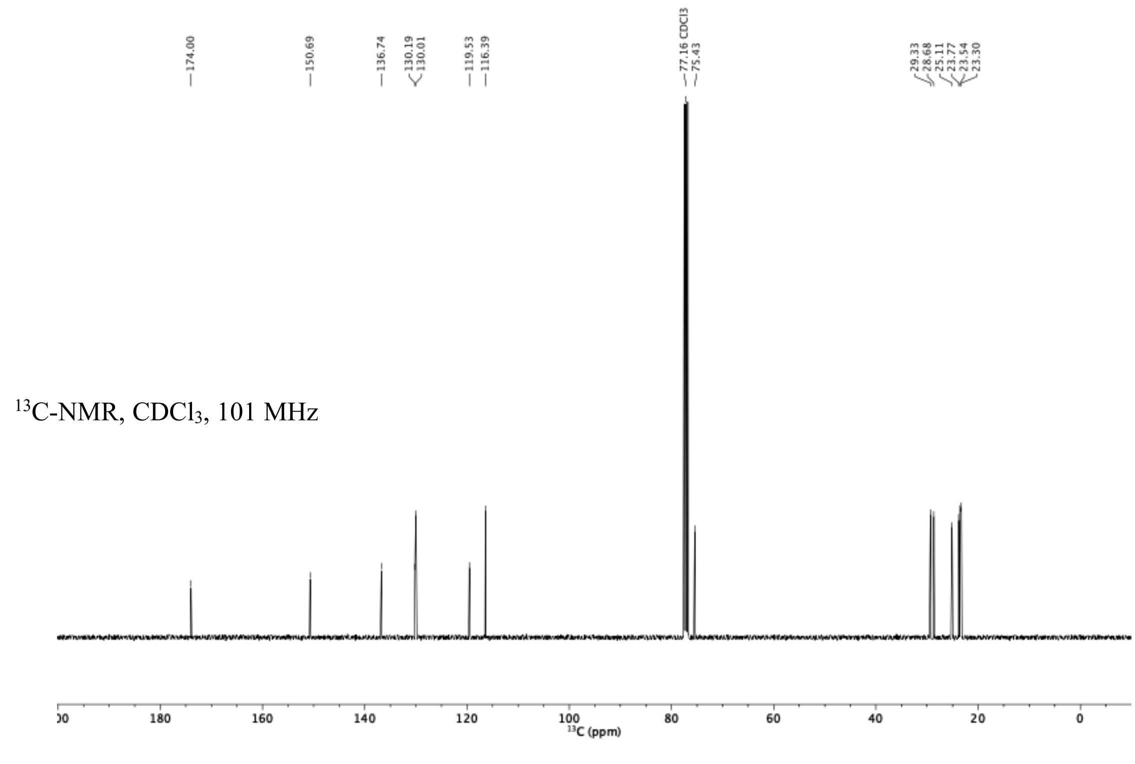

6-(Pyridine-3-yl)chromane-2-carboxamide (*rac*-1n)

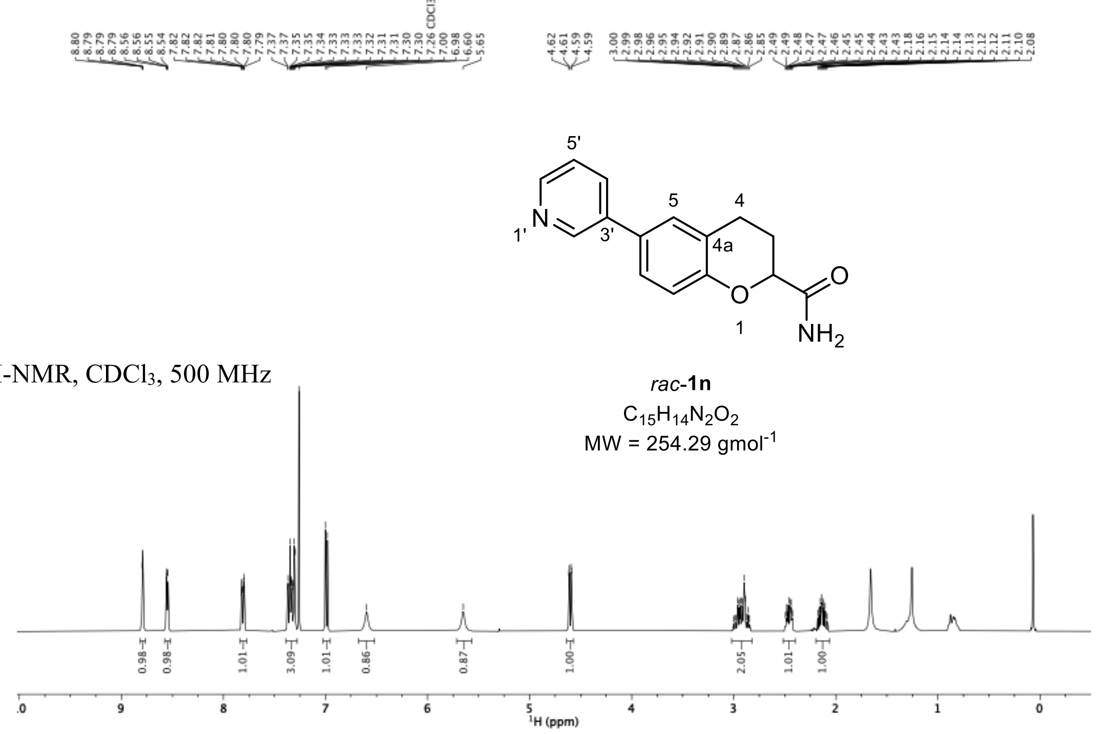

^{1}H-NMR, $CDCl_3$, 500 MHz

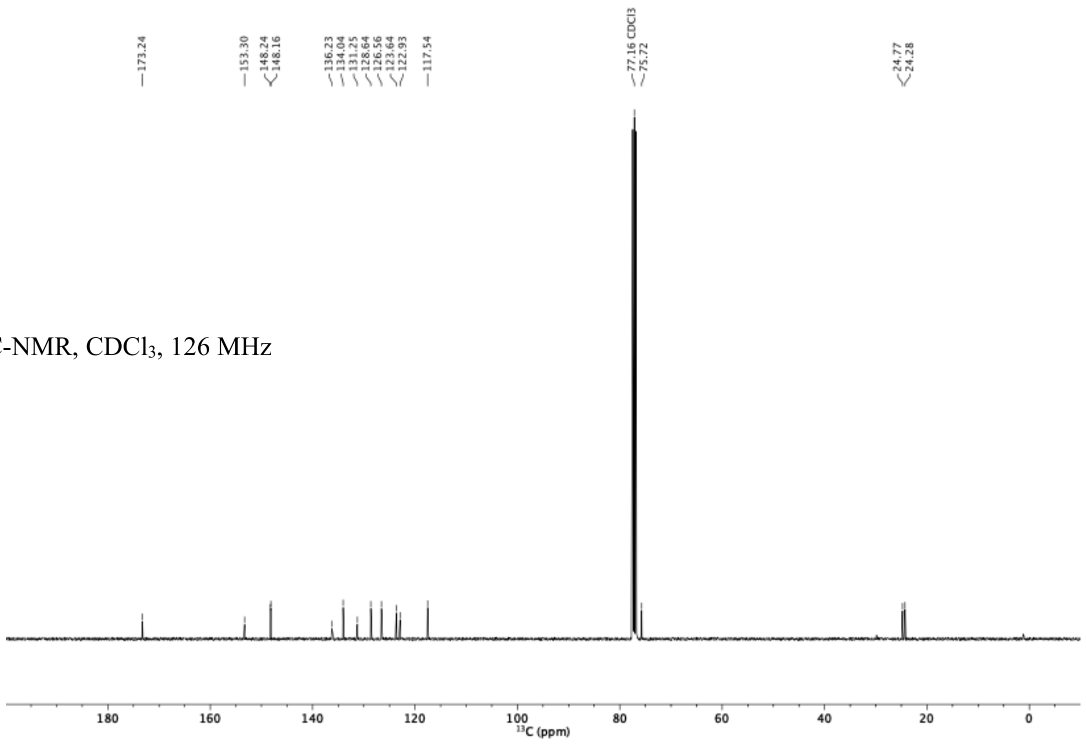

^{13}C-NMR, $CDCl_3$, 126 MHz

7-Methoxychromane-2-carboxamide (*rac*-**1o**)

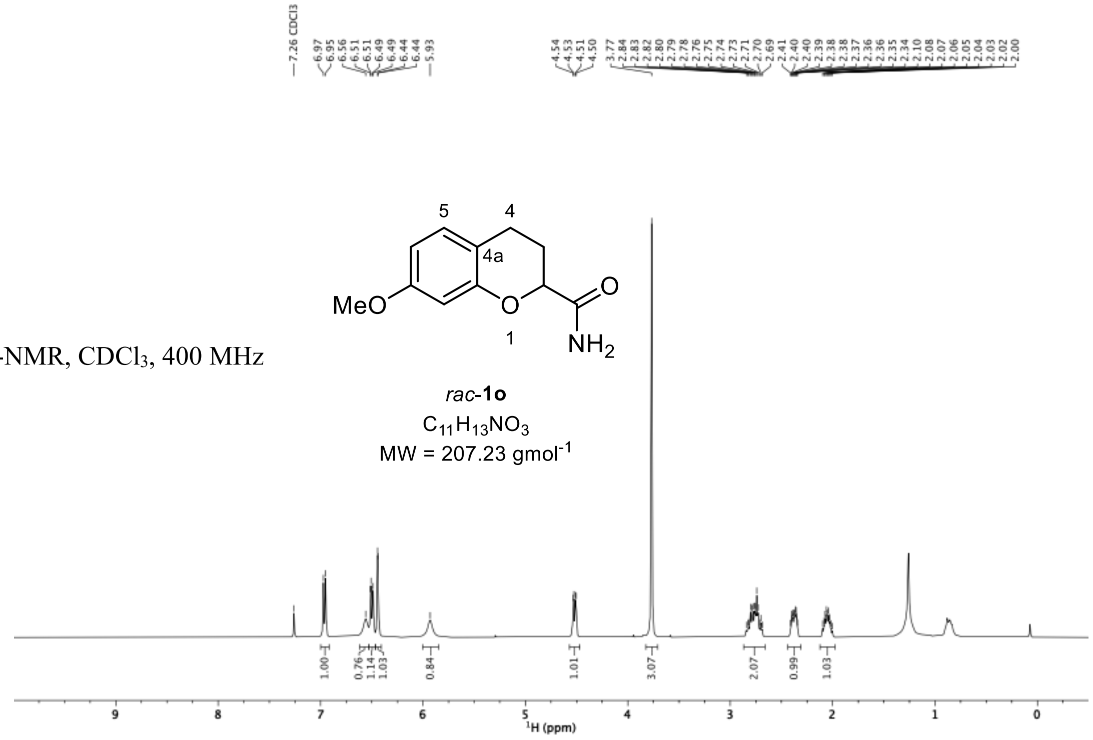

^{1}H-NMR, $CDCl_3$, 400 MHz

^{13}C-NMR, $CDCl_3$, 101 MHz

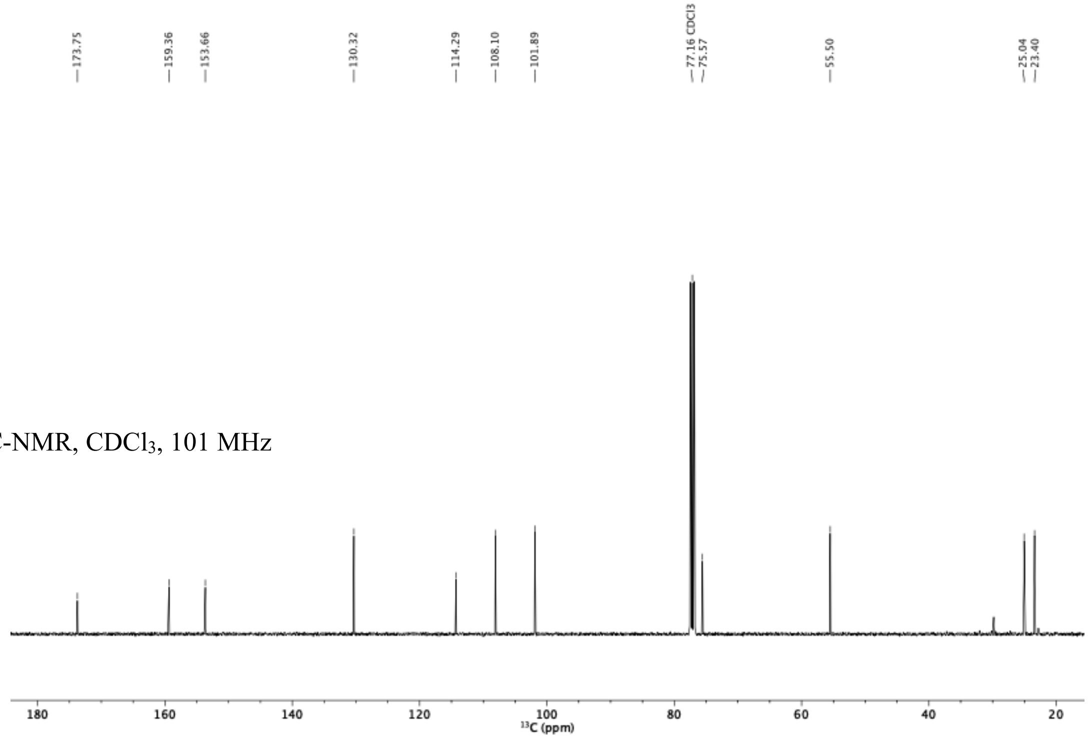

7-Fluoro-chromane-2-carboxamide (*rac*-1p)

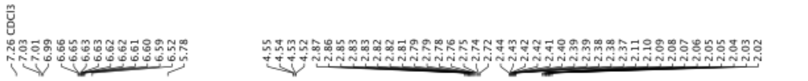

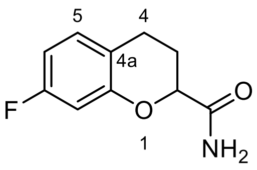

^{1}H-NMR, $CDCl_3$, 400 MHz

rac-**1p**

$C_{10}H_{10}FNO_2$

MW = 195.19 gmol^{-1}

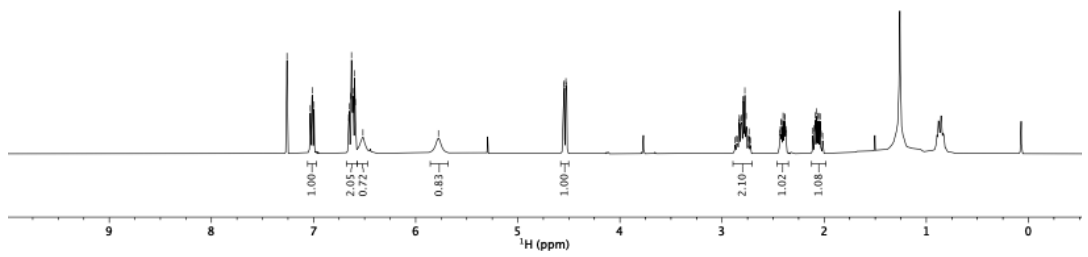

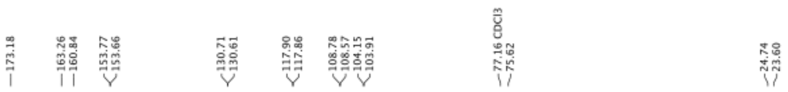

^{13}C-NMR, $CDCl_3$, 101 MHz

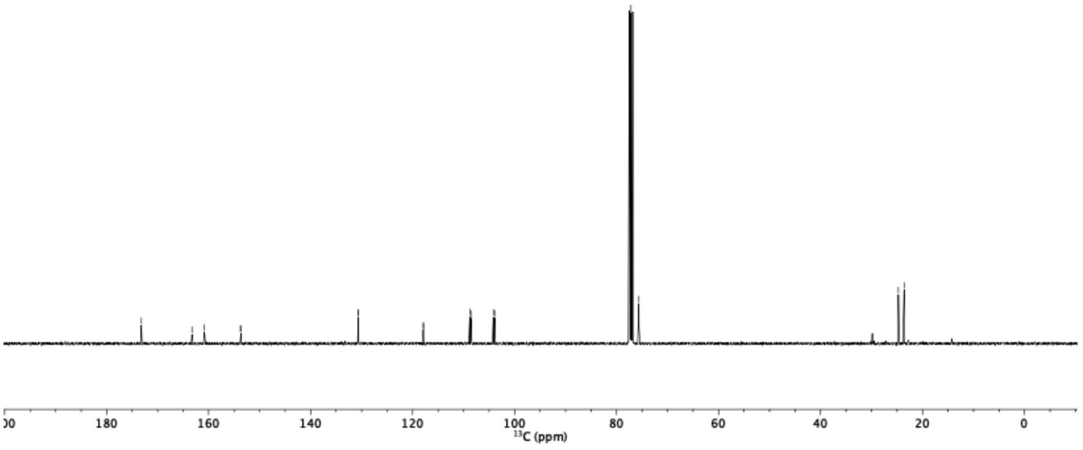

^{19}F-NMR, $CDCl_3$, 376 MHz

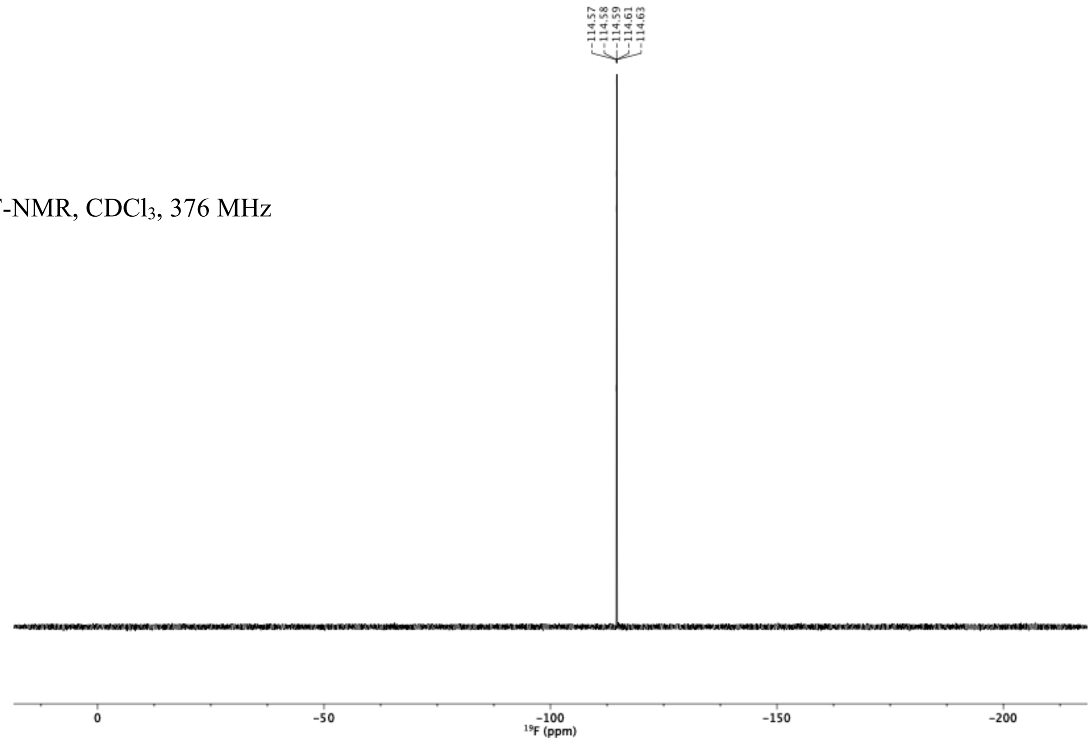

8-Fluorochromane-2-carboxamide (*rac*-**1q**)

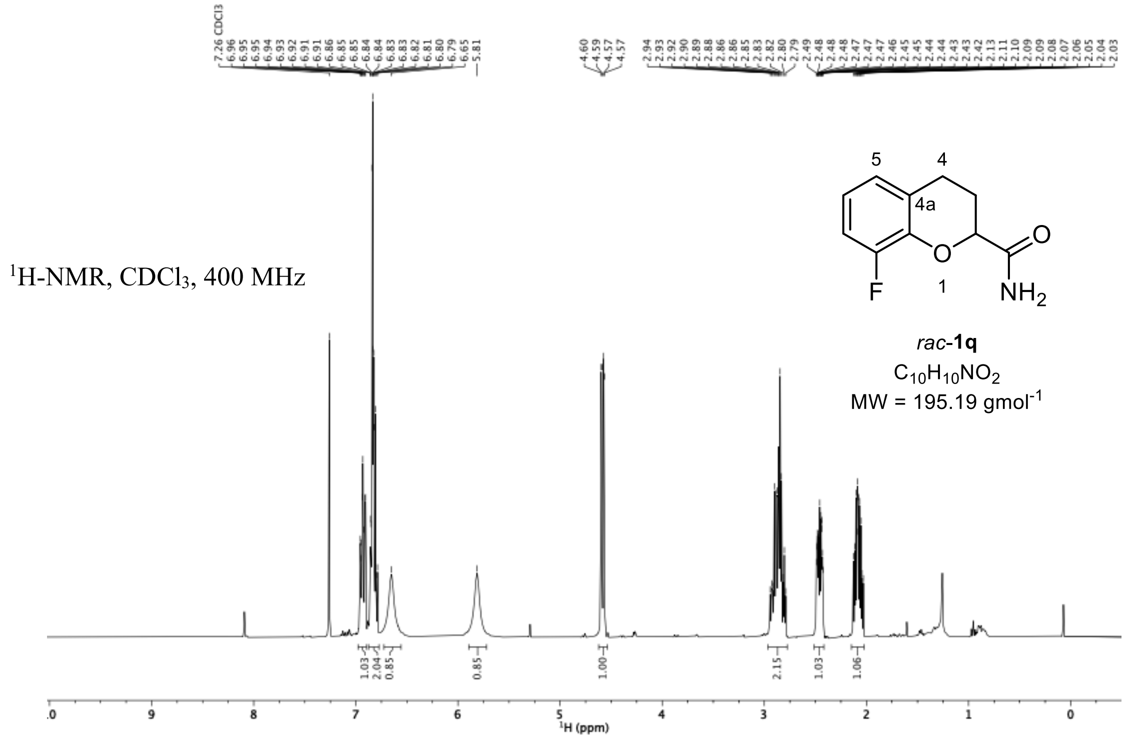

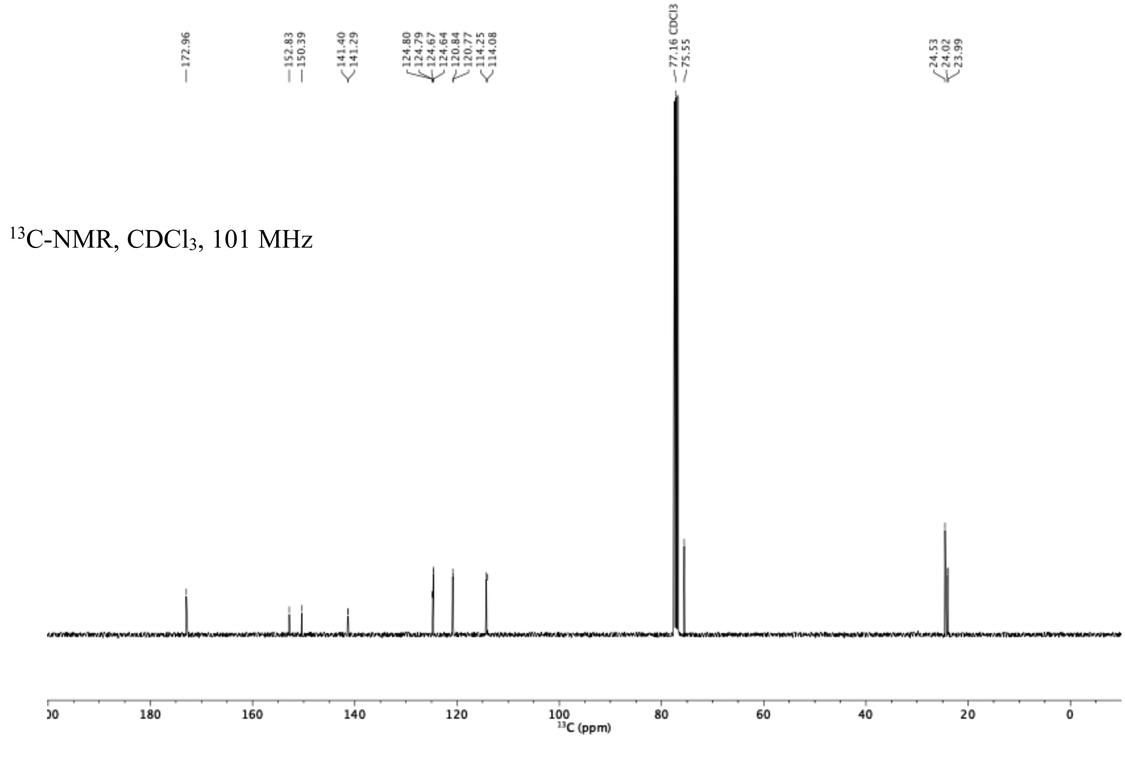

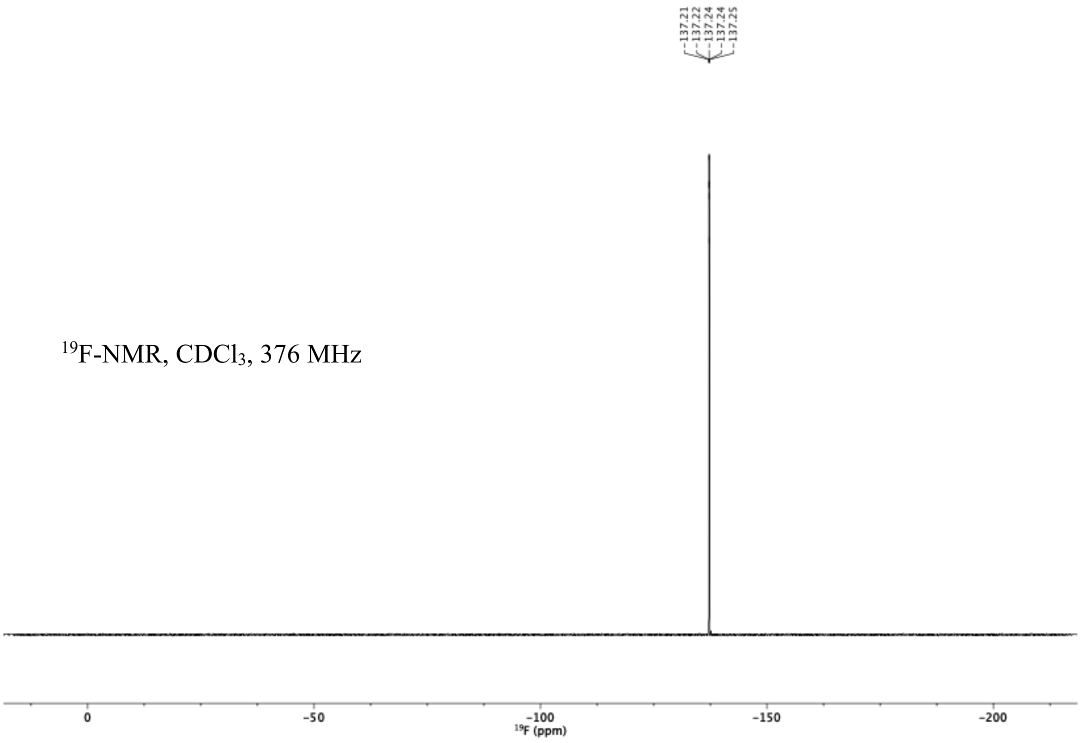

-137.21
-137.22
-137.24
-137.24
-137.25
^{19}F-NMR, $CDCl_3$, 376 MHz
0
-50
-100
^{19}F (ppm)
-150
-200

1,4-Benzodioxane-2-carboxamide (*rac*-1r)

^{1}H-NMR, $CDCl_3$, 400 MHz

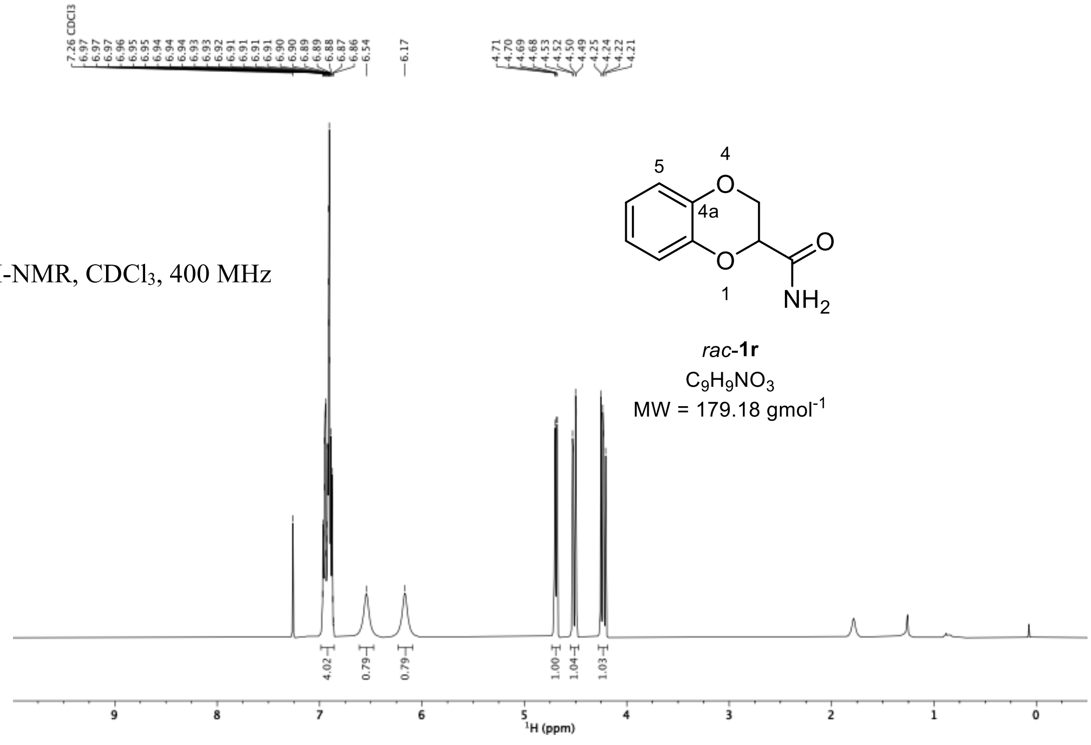

^{13}C-NMR, $CDCl_3$, 101 MHz

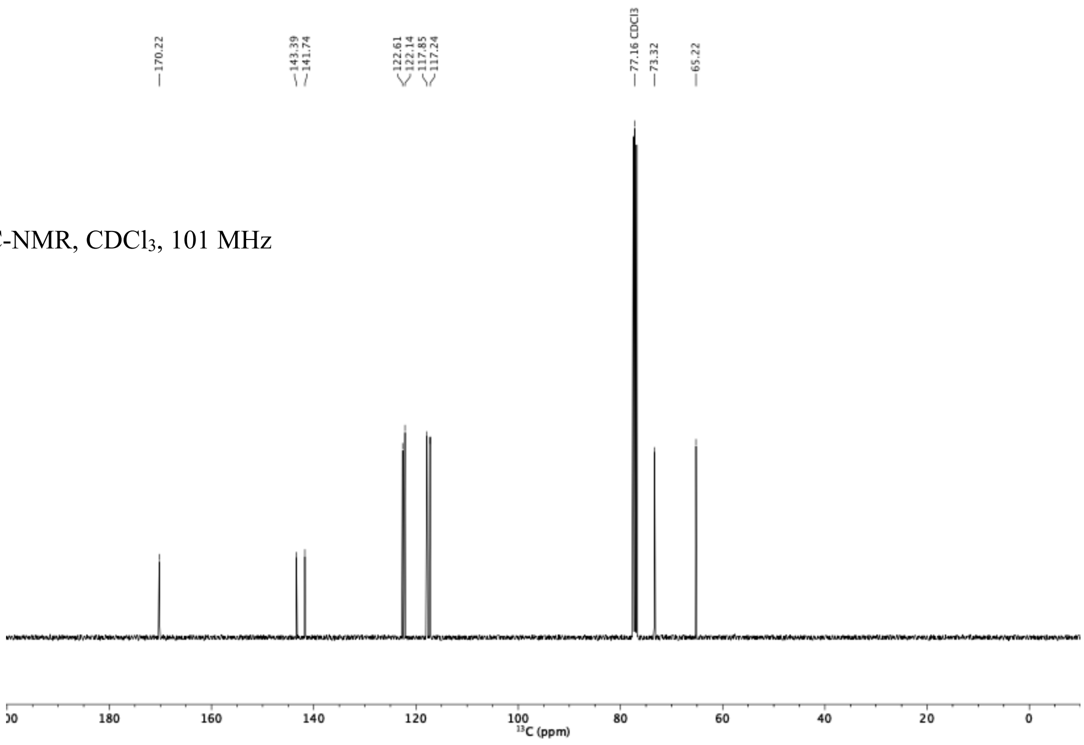

3,3-Dimethyl-1,4-benzodioxane-2-carboxamide (*rac*-1s)

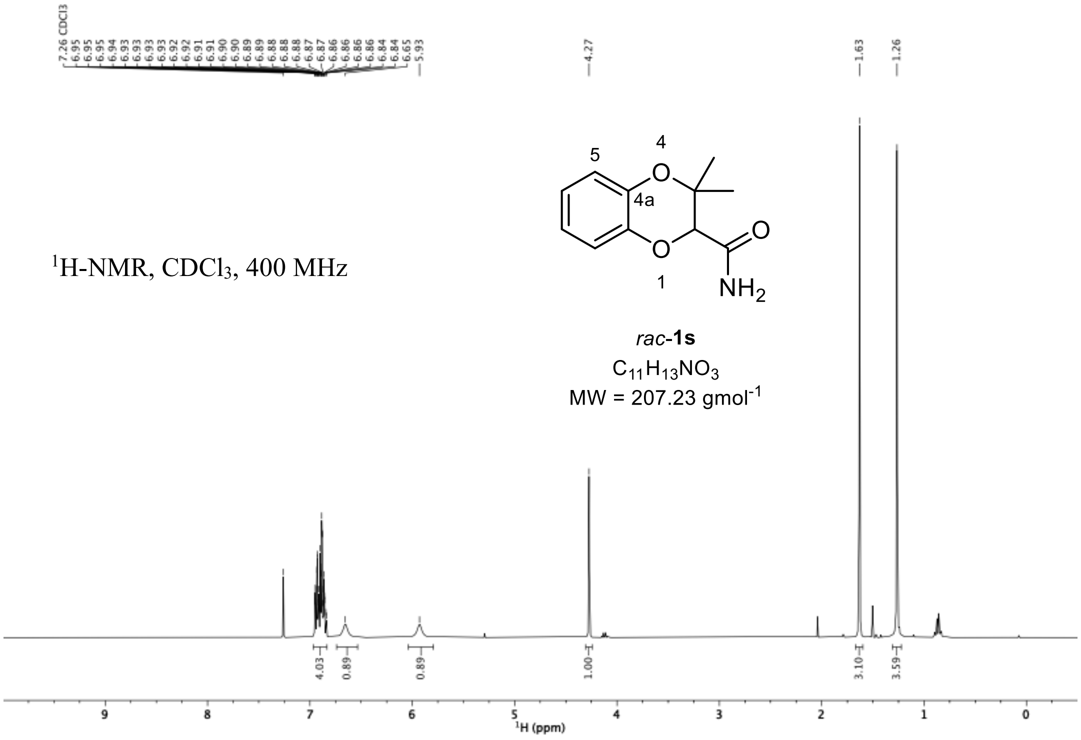

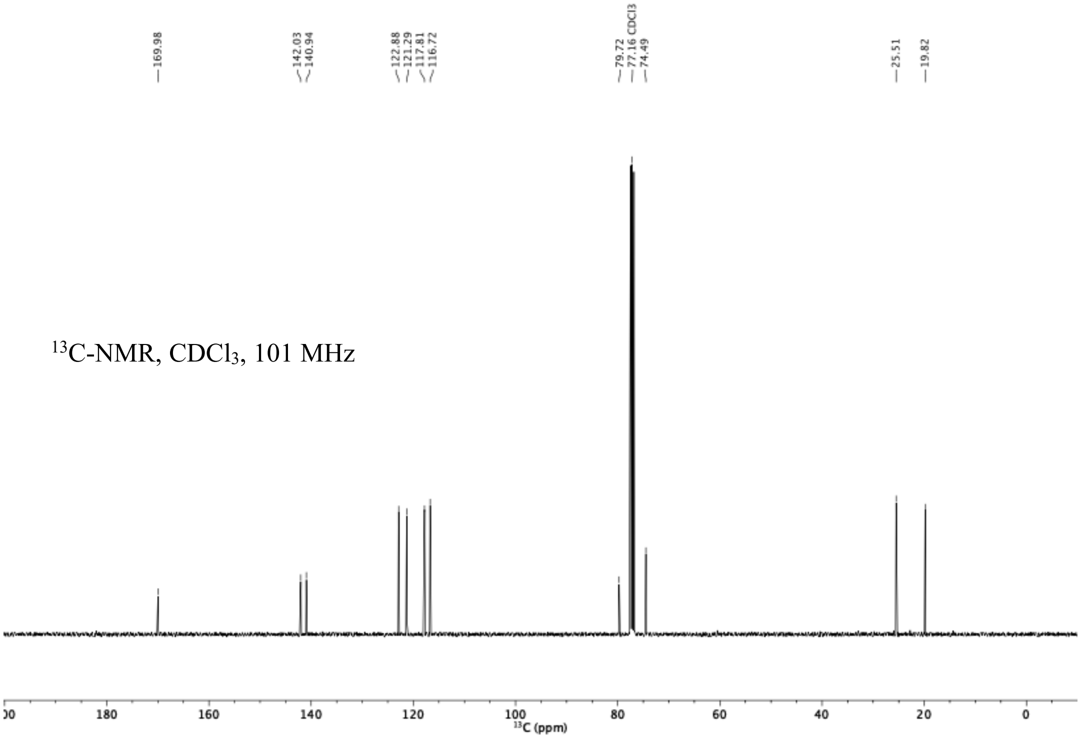

2,3-Dihydrobenzo[*b*][1,4]oxazine-2-carboxamide (*rac*-**1t**)

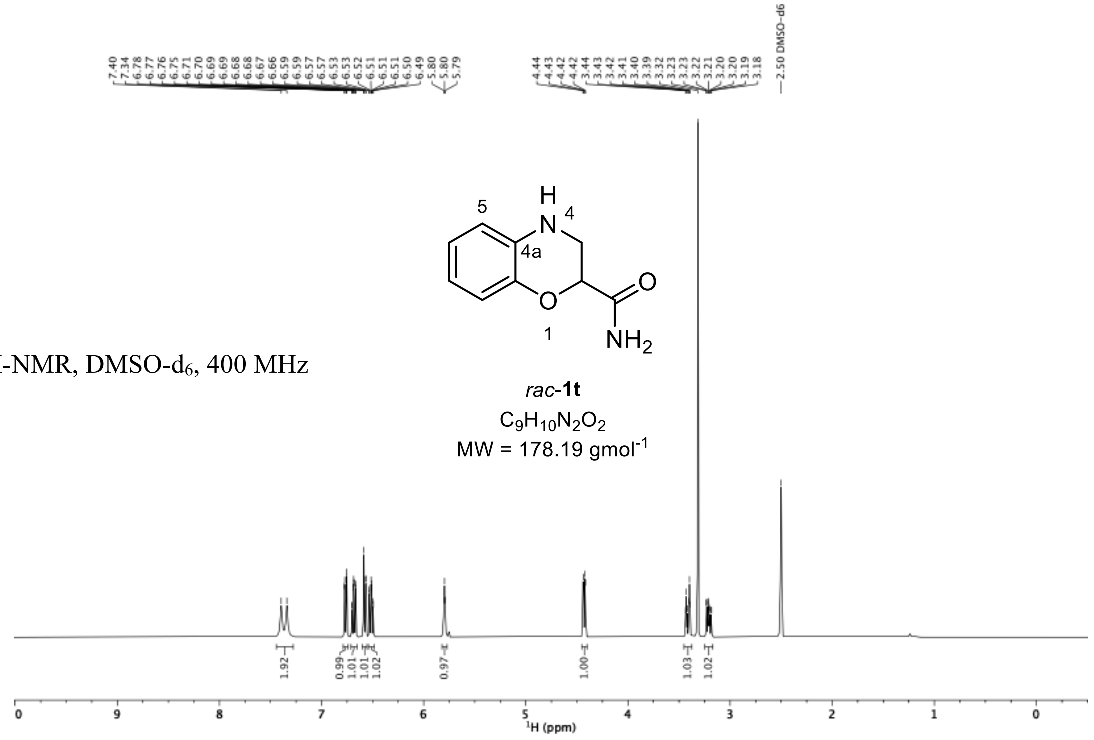

^{1}H-NMR, DMSO-d$_6$, 400 MHz

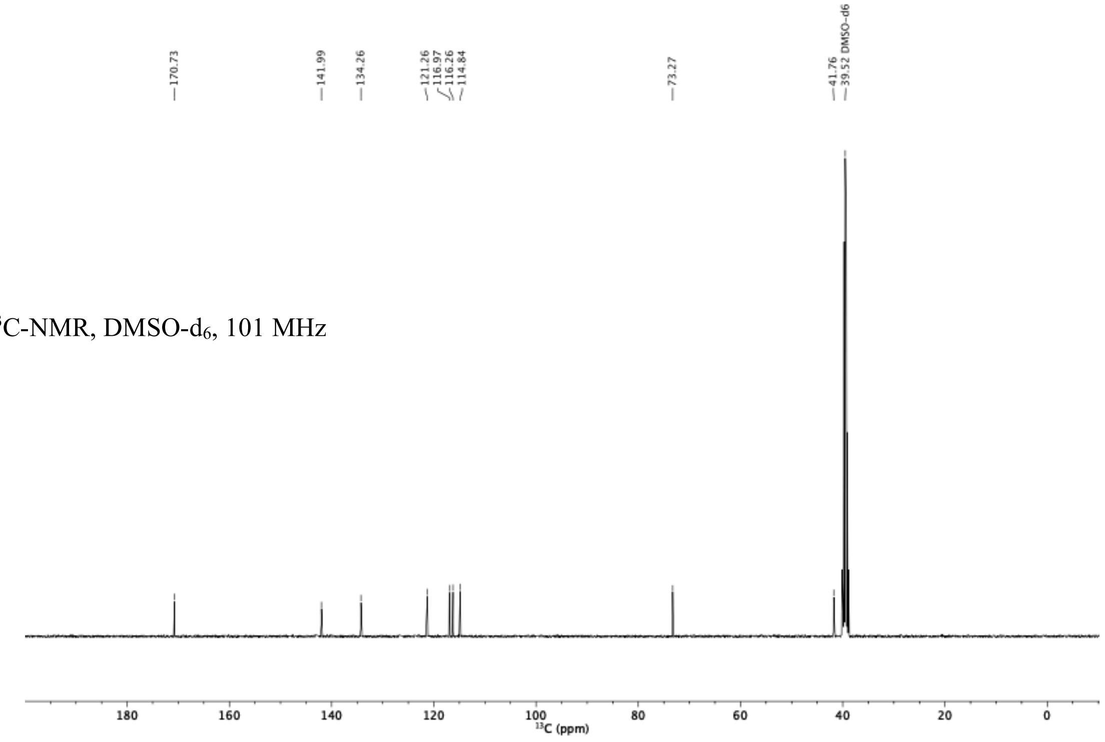

^{13}C-NMR, DMSO-d$_6$, 101 MHz

4-Boc-2,3-Dihydrobenzo[*b*][1,4]oxazine-2-carboxamide (*rac*-**1u**)

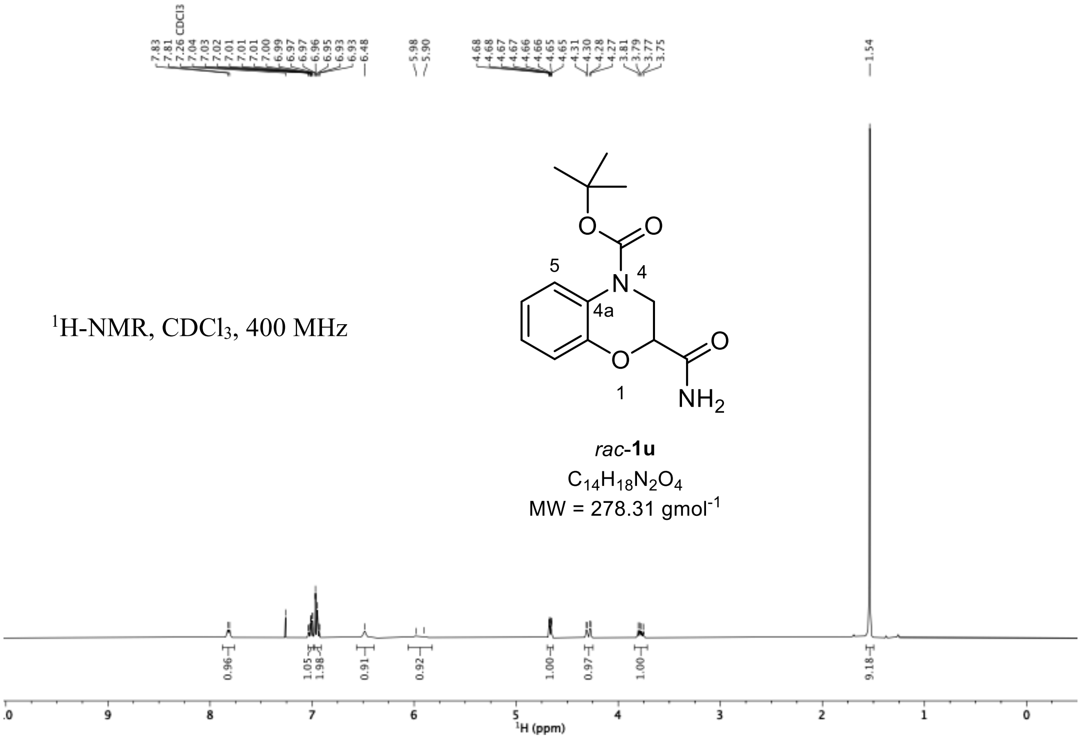

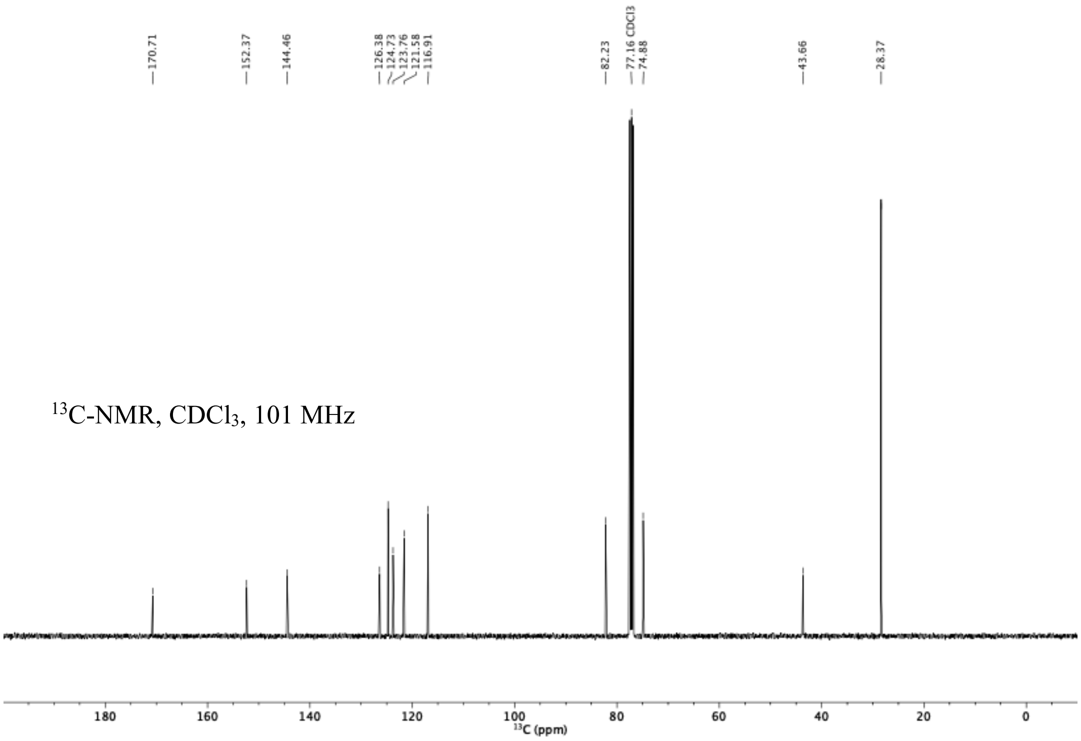

cis-3-Methyl-1,4-benzodioxane-2-carboxamide (_rac_-_cis_-1v)

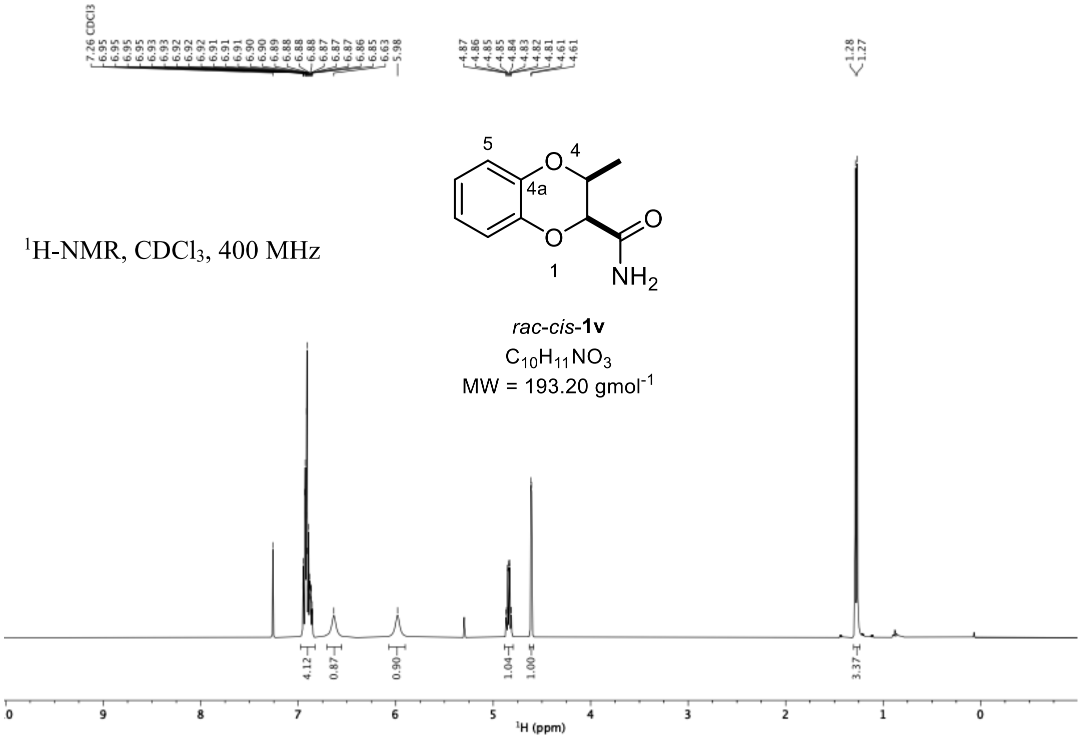

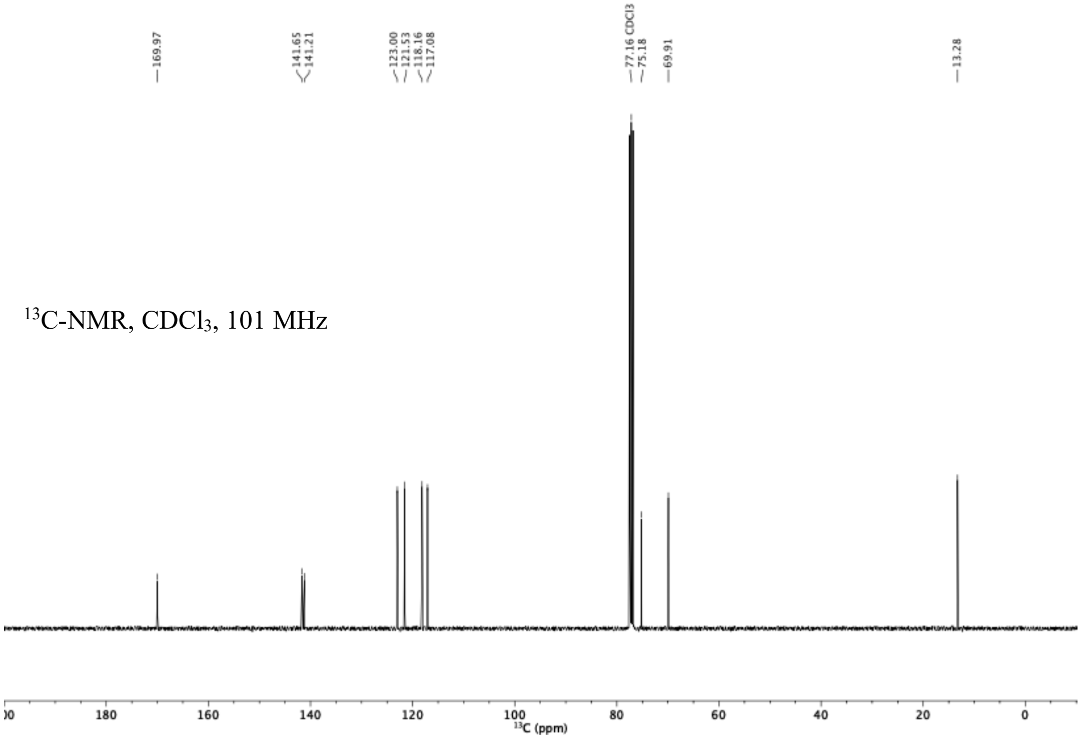

***trans*-3-Methyl-1,4-benzodioxane-2-carboxamide (*rac-trans*-1v)**

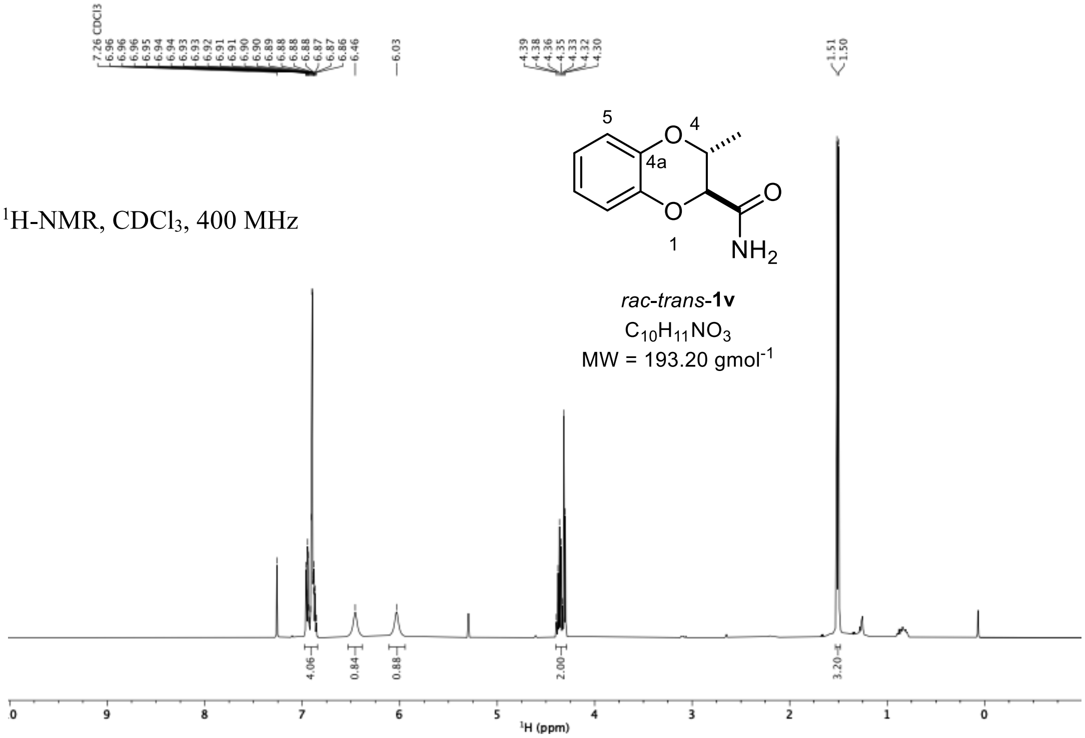

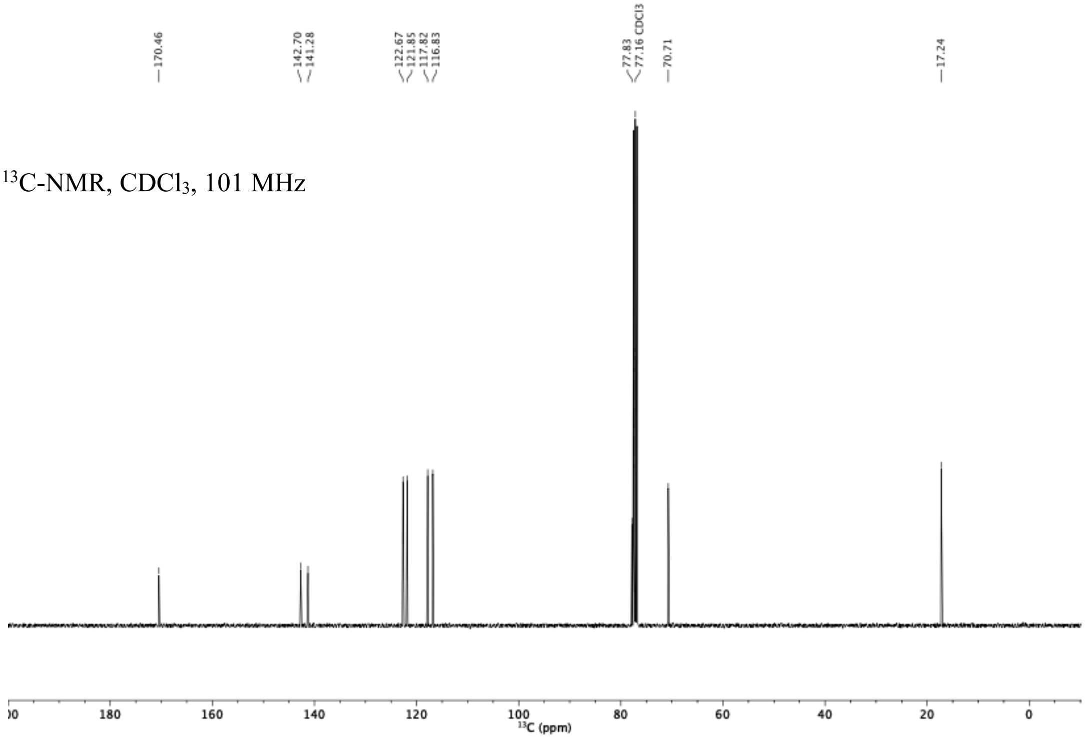

1,2,3,4-Tetrahydronaphthalene-2-carboxamide (*rac*-**12**)

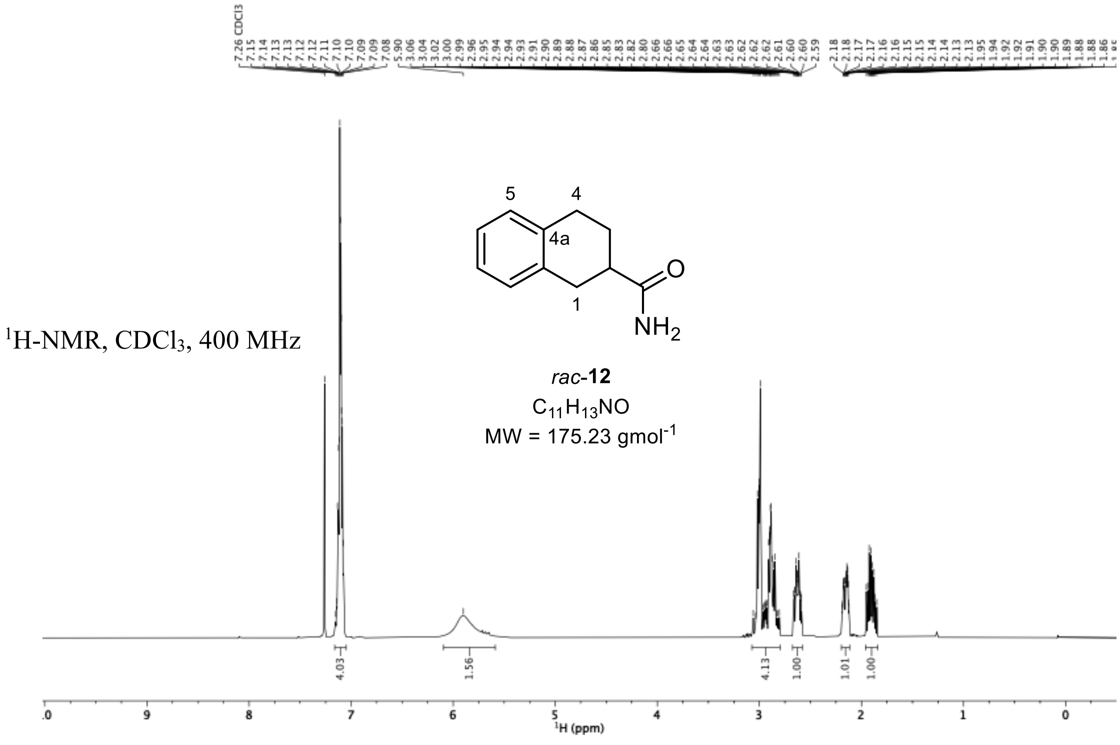

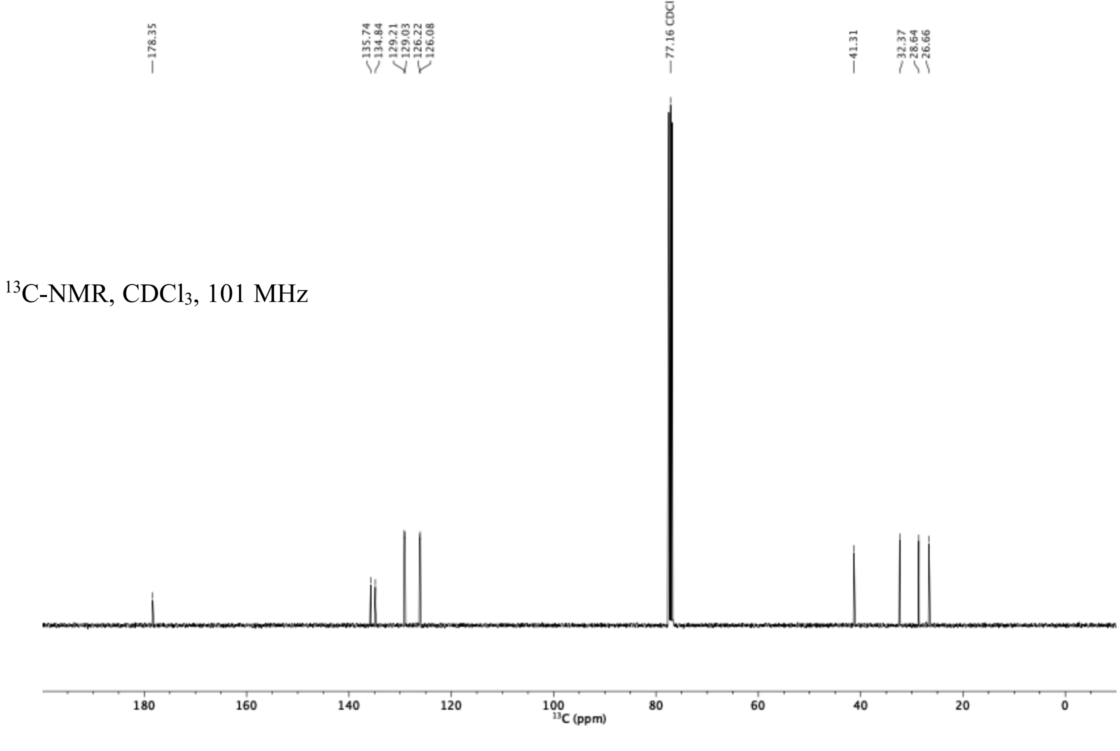

Thiochromane-2-carboxamide (*rac*-13)

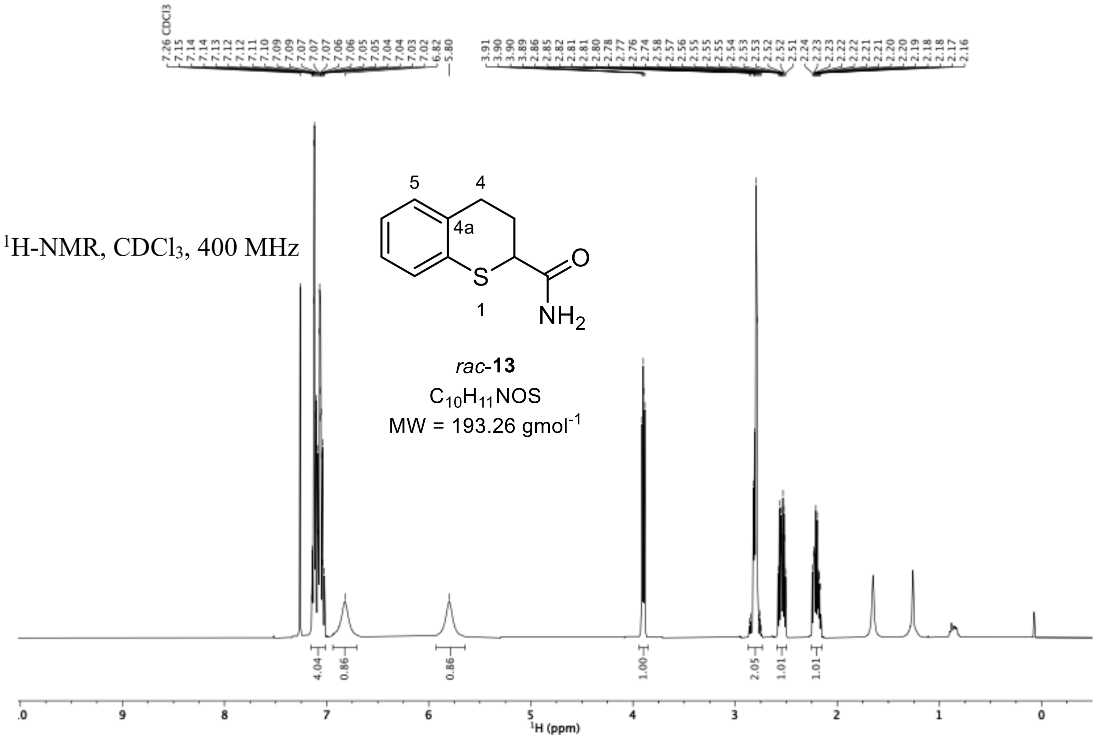

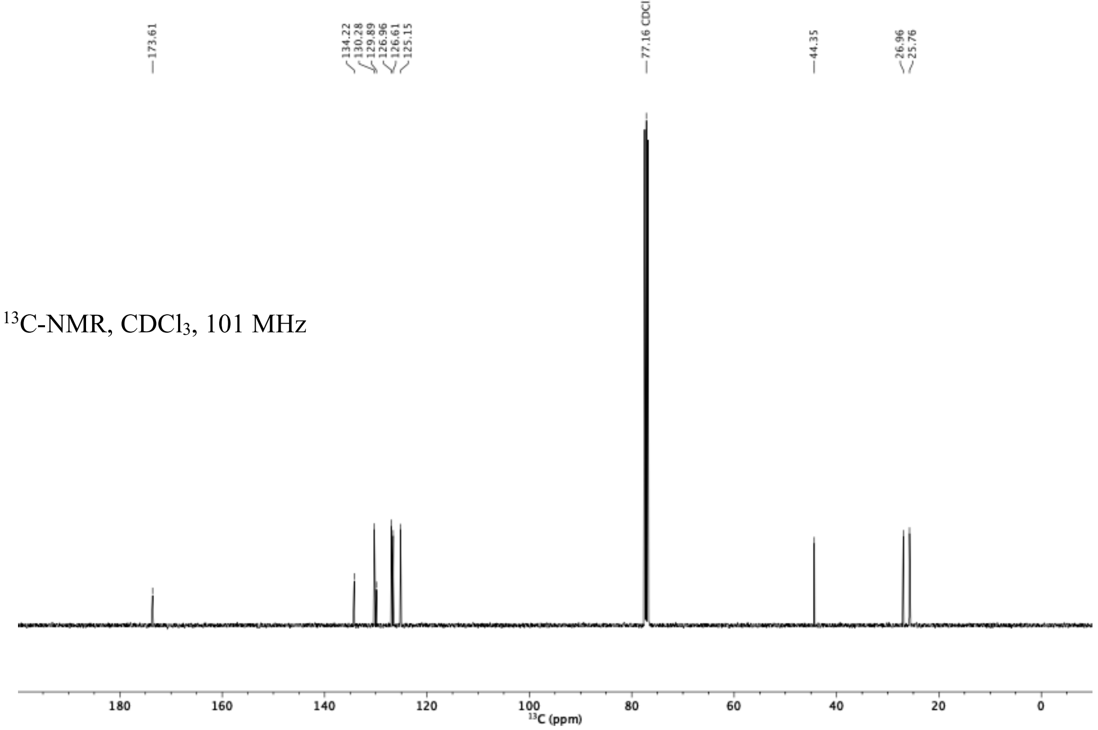

(*R*)-Chroman-2-ylmethanamine (3)

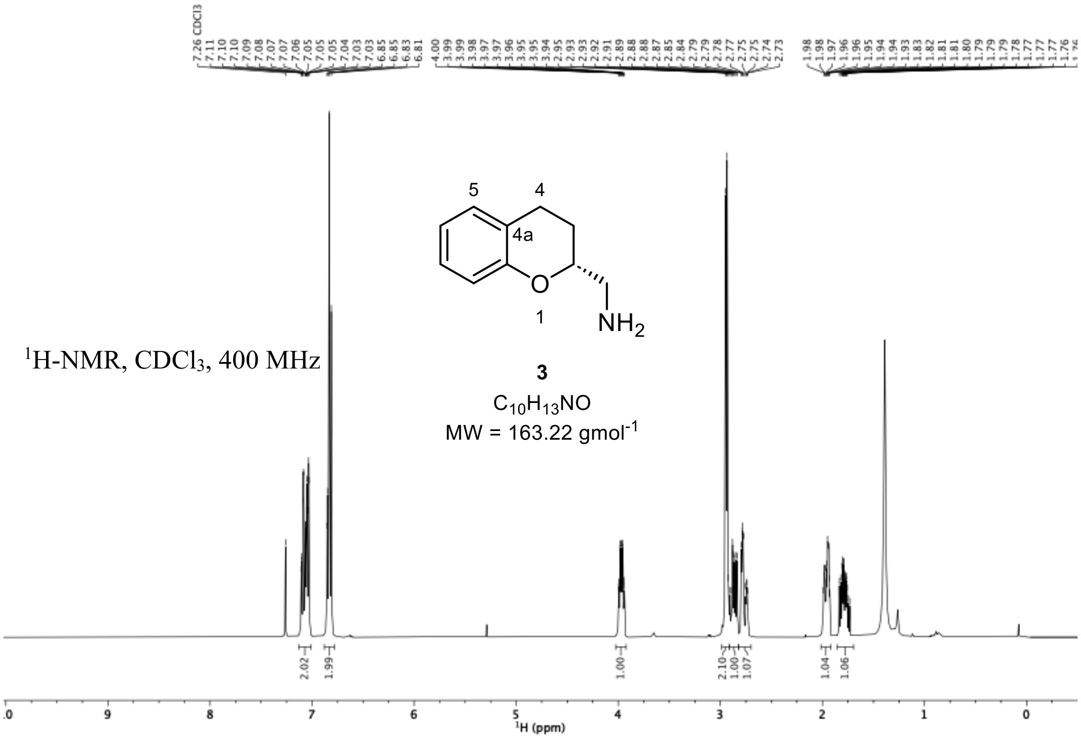

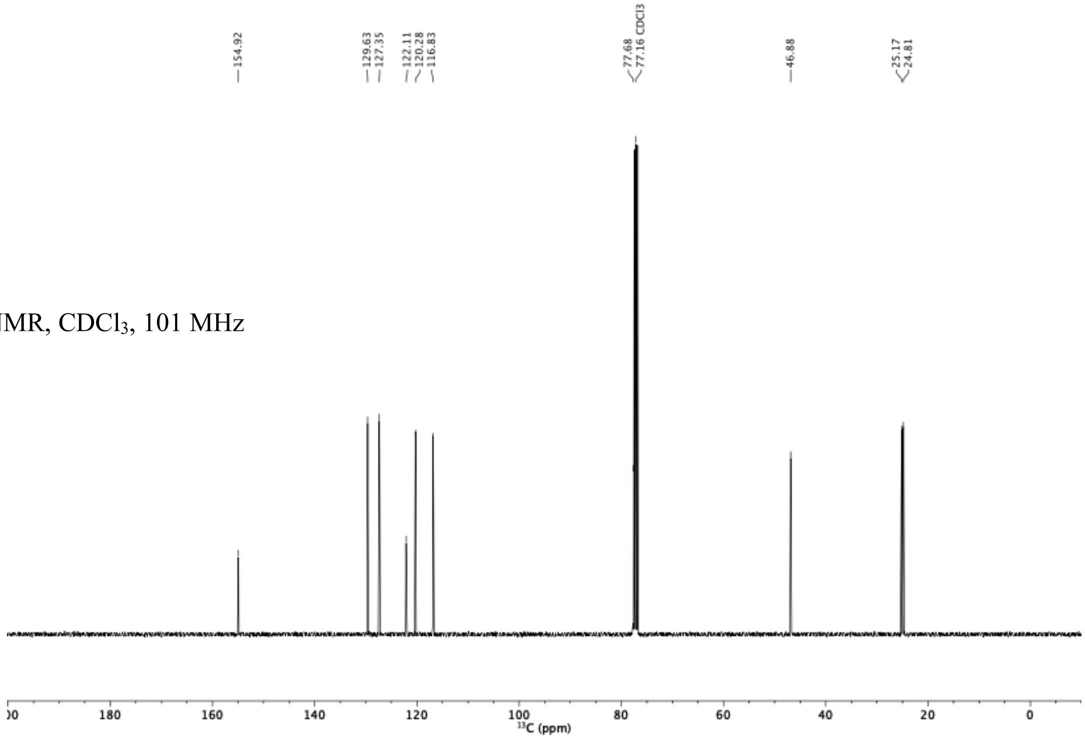

2-(4-Bromobutyl)benzo[*d*]isothiazol-3(2*H*)-one 1,1-dioxide (4)

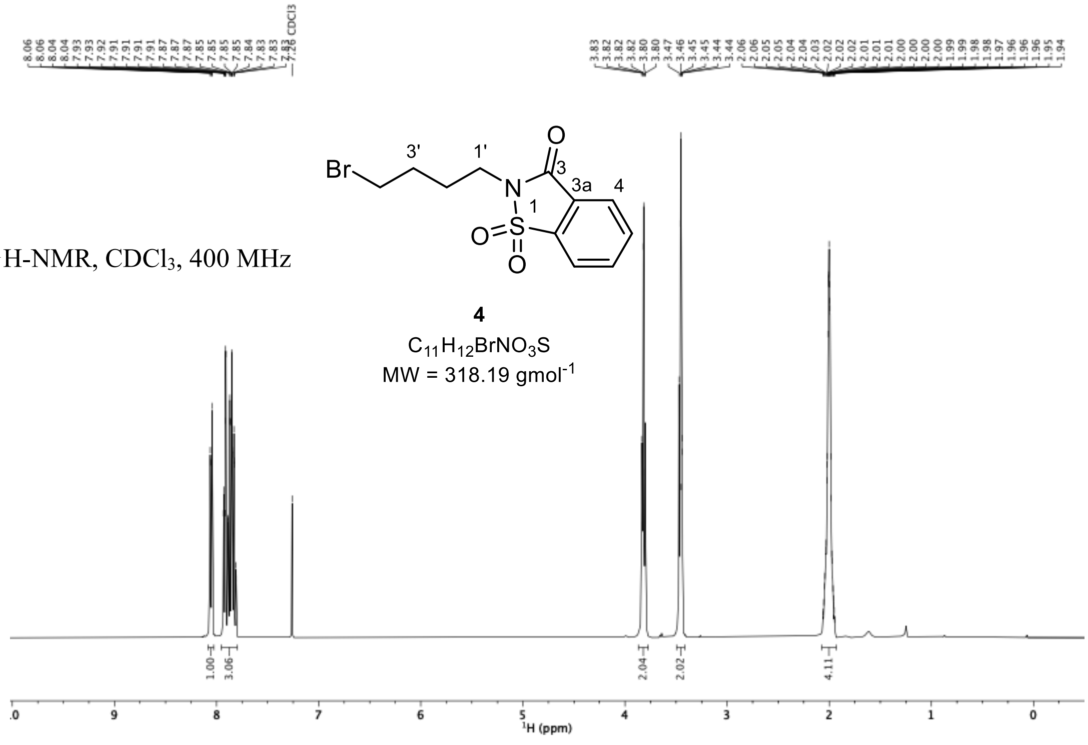

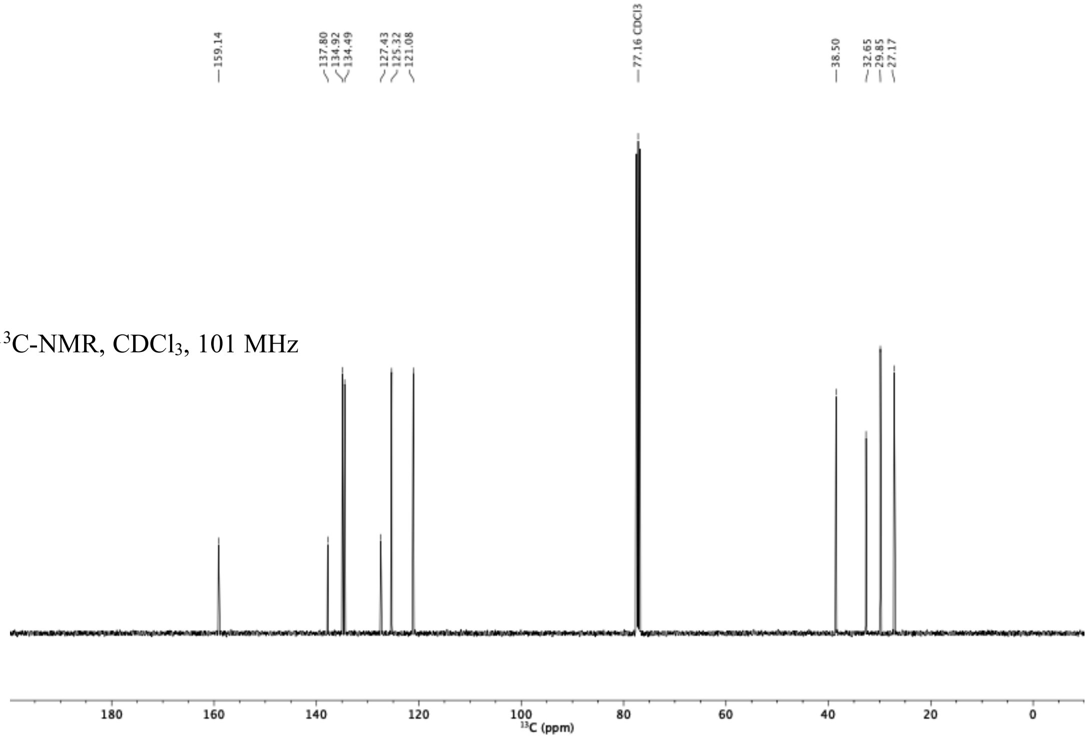

(*R*)-2-(4-((Chroman-2-ylmethyl)amino)butyl)benzo[*d*]isothiazol-3(2*H*)-one 1,1-dioxide (Repinotan)

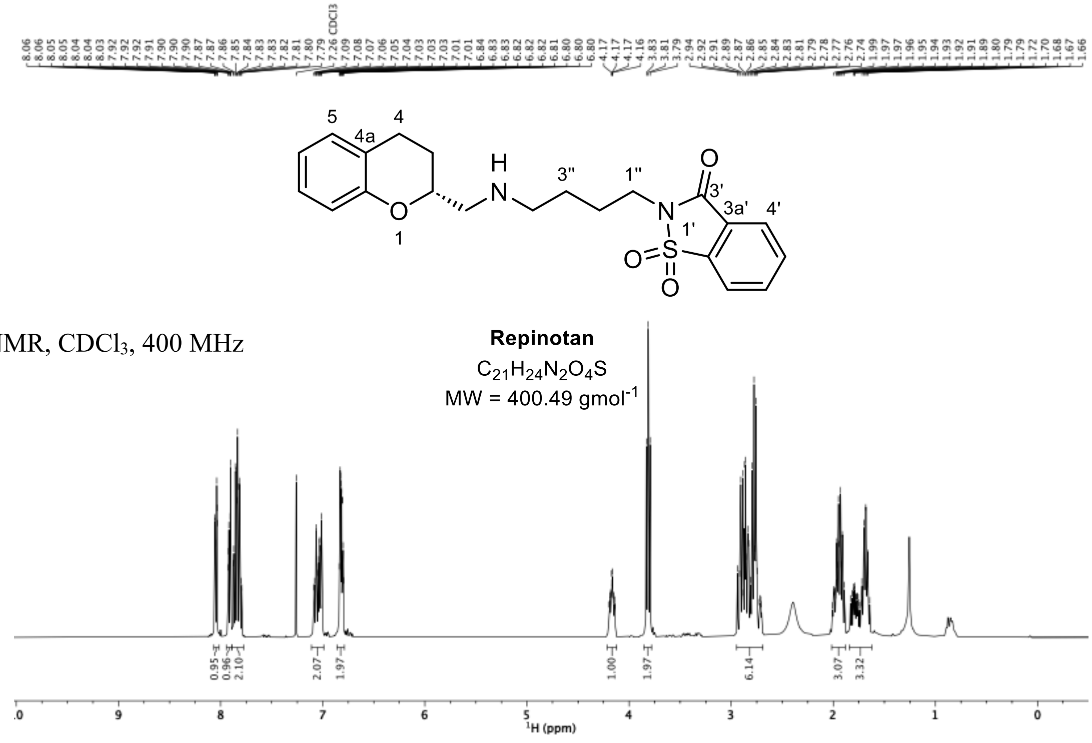

^{1}H-NMR, $CDCl_3$, 400 MHz

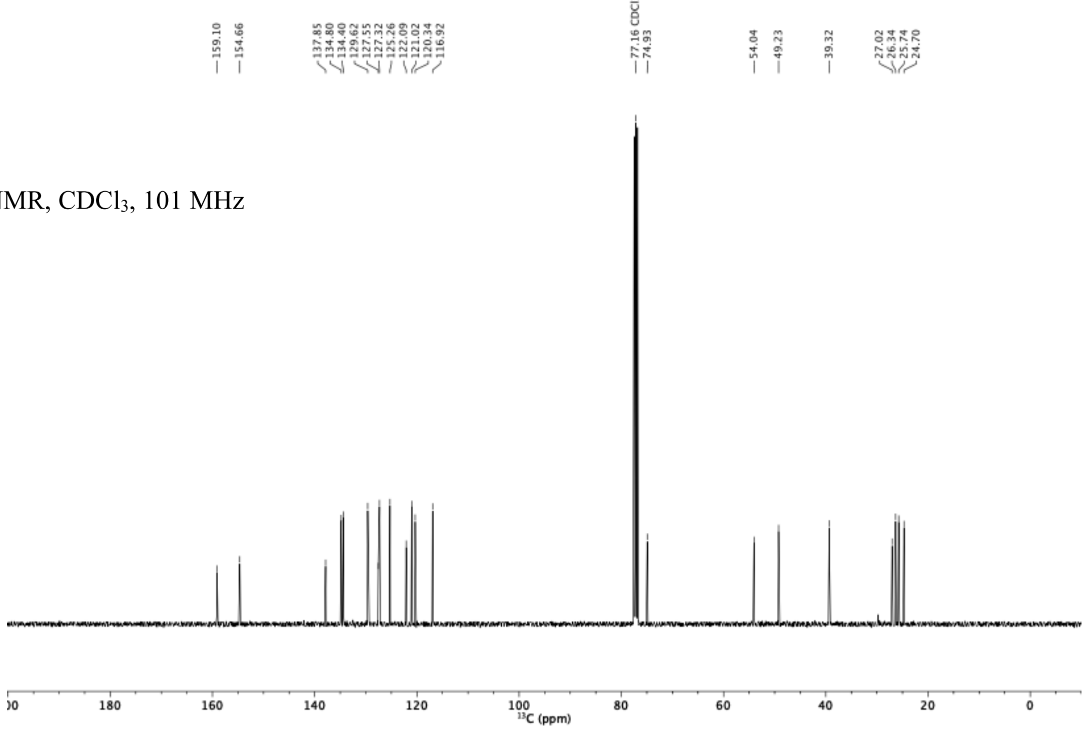

^{13}C-NMR, $CDCl_3$, 101 MHz

5-(4-Fluorophenyl)nicotinaldehyde (5)

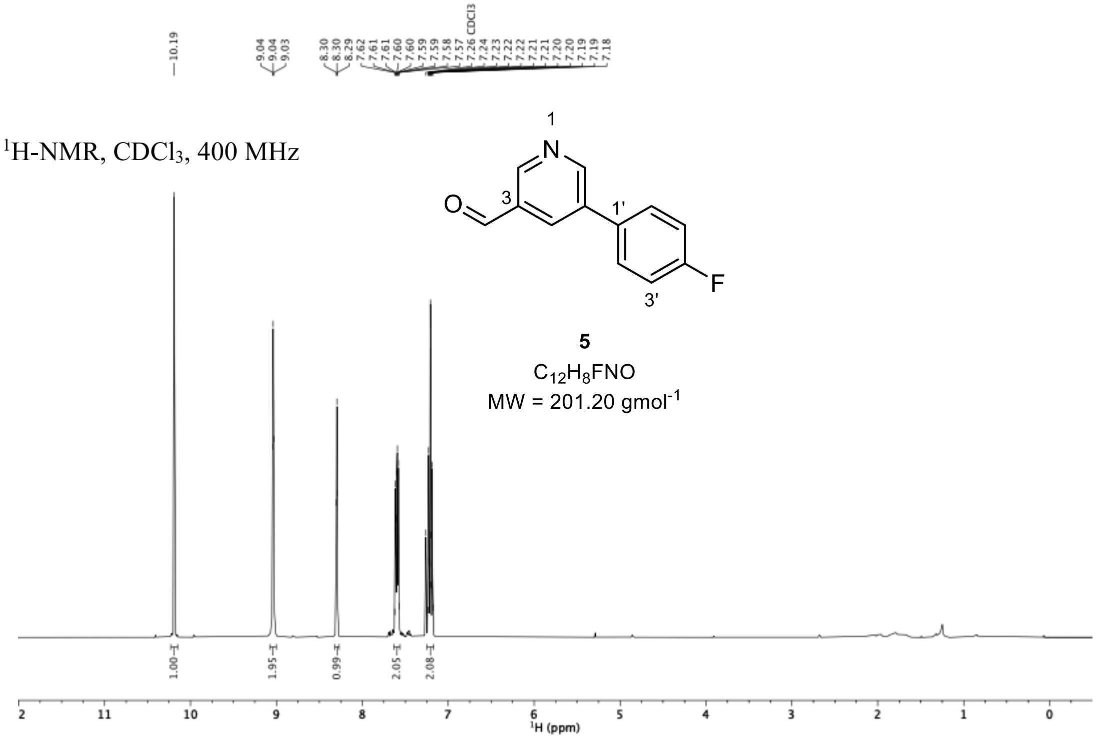

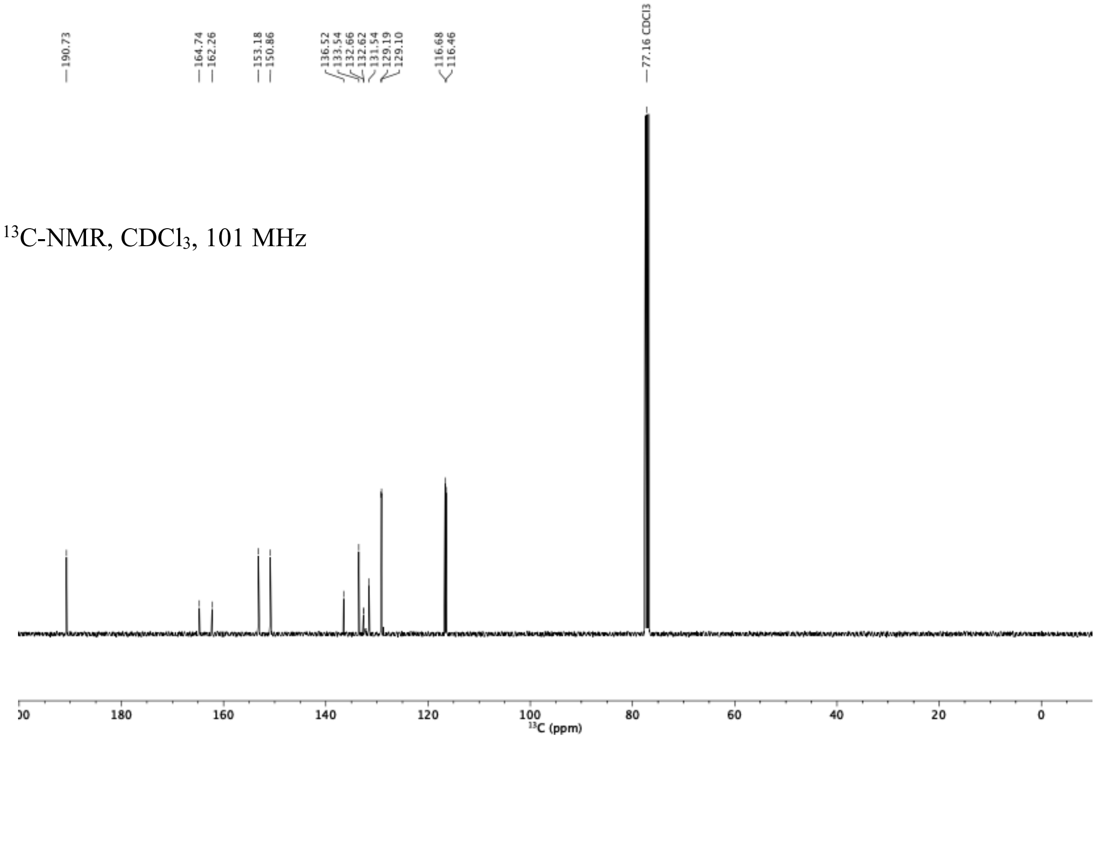

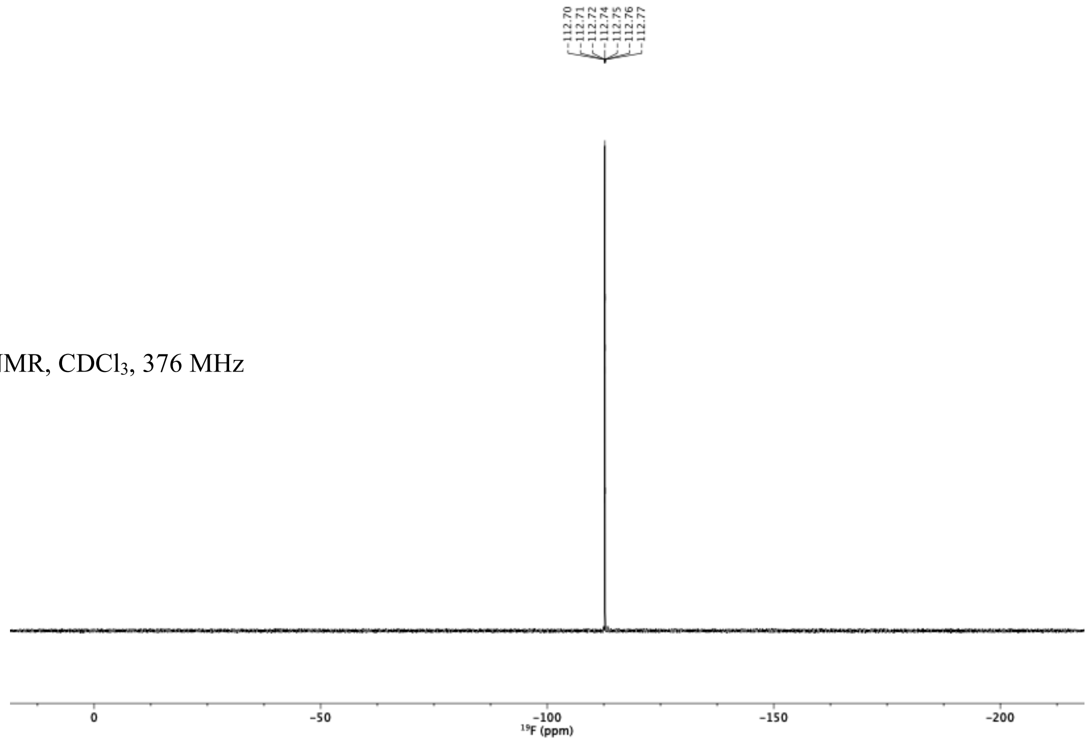

^{19}F-NMR, $CDCl_3$, 376 MHz

R)-1-(chroman-2-yl)-N-((5-(4-fluorophenyl)pyridin-3-yl)methyl)methanamine (Sarizotan)

^{1}H-NMR, $CDCl_3$, 500 MHz

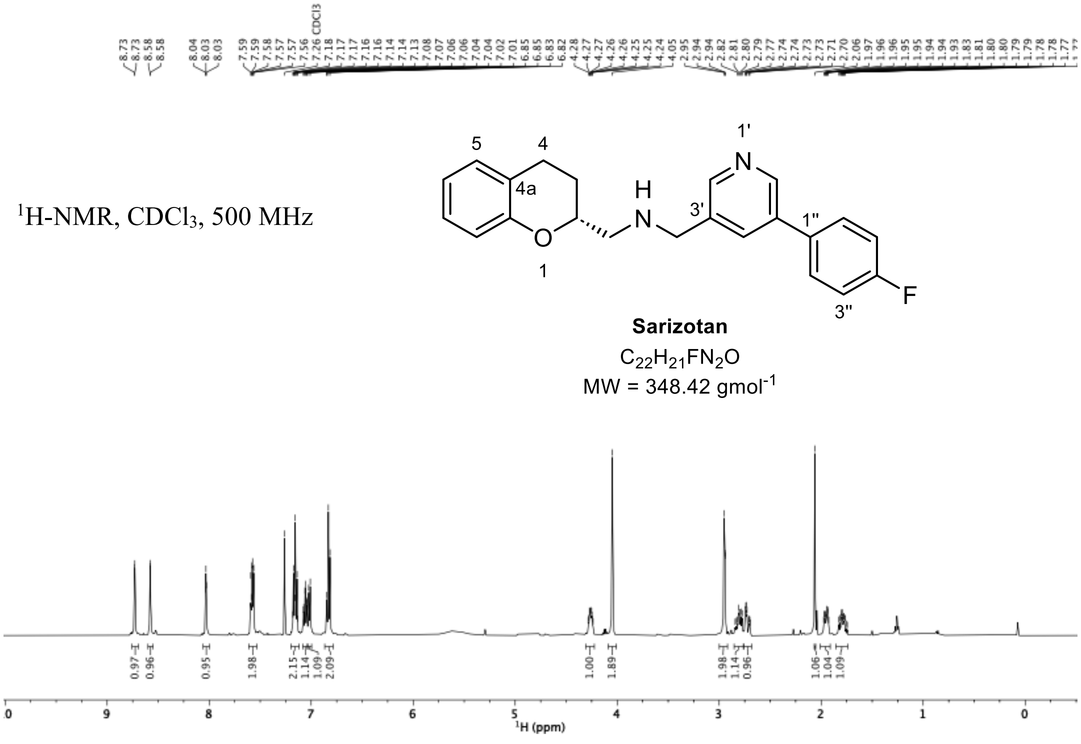

^{13}C-NMR, $CDCl_3$, 126 MHz

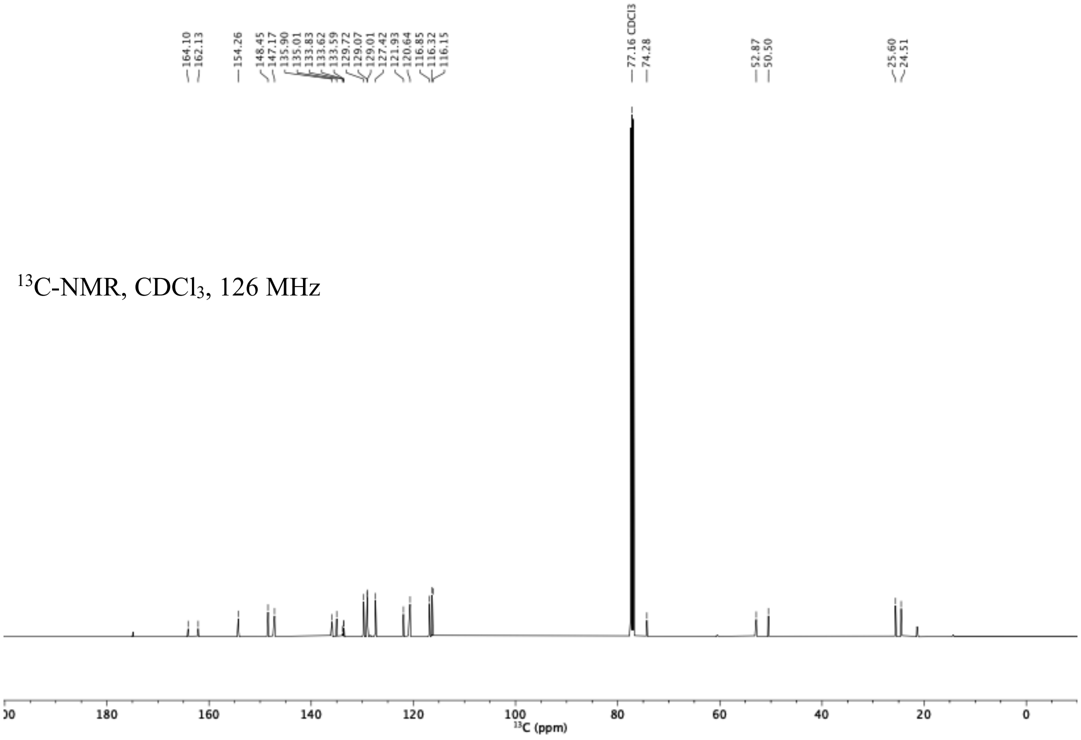

^{19}F-NMR, $CDCl_3$, 471 MHz

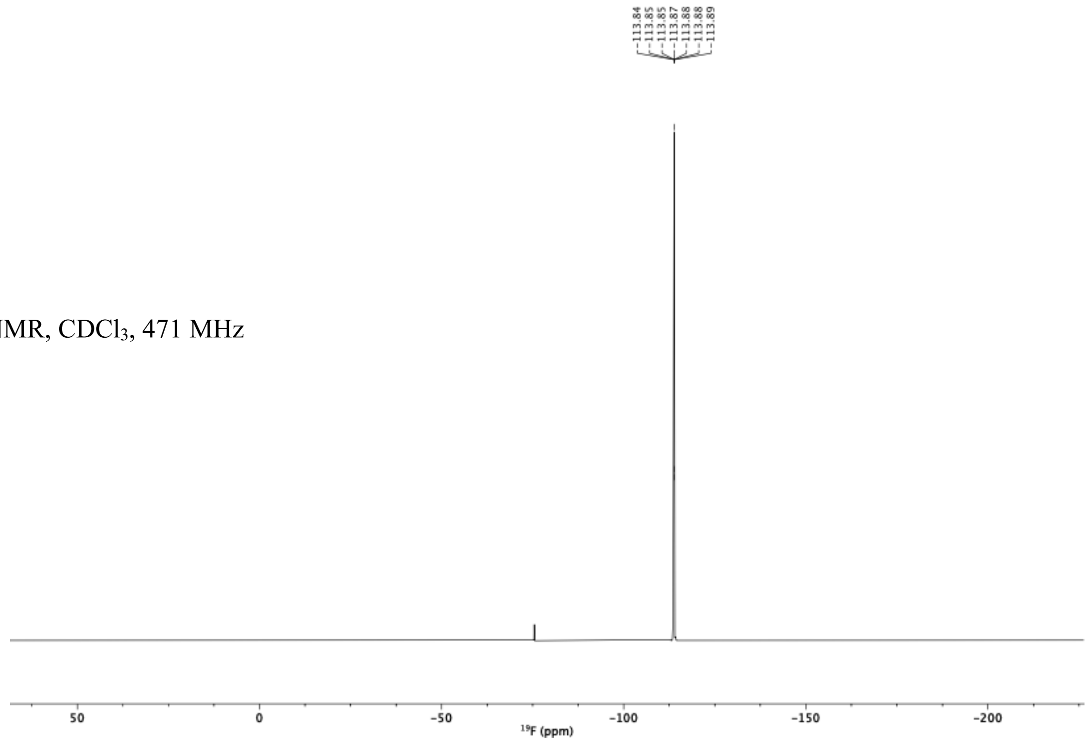

(*R*)-1,4-Benzodioxane-2-carboxylic acid (S-6)

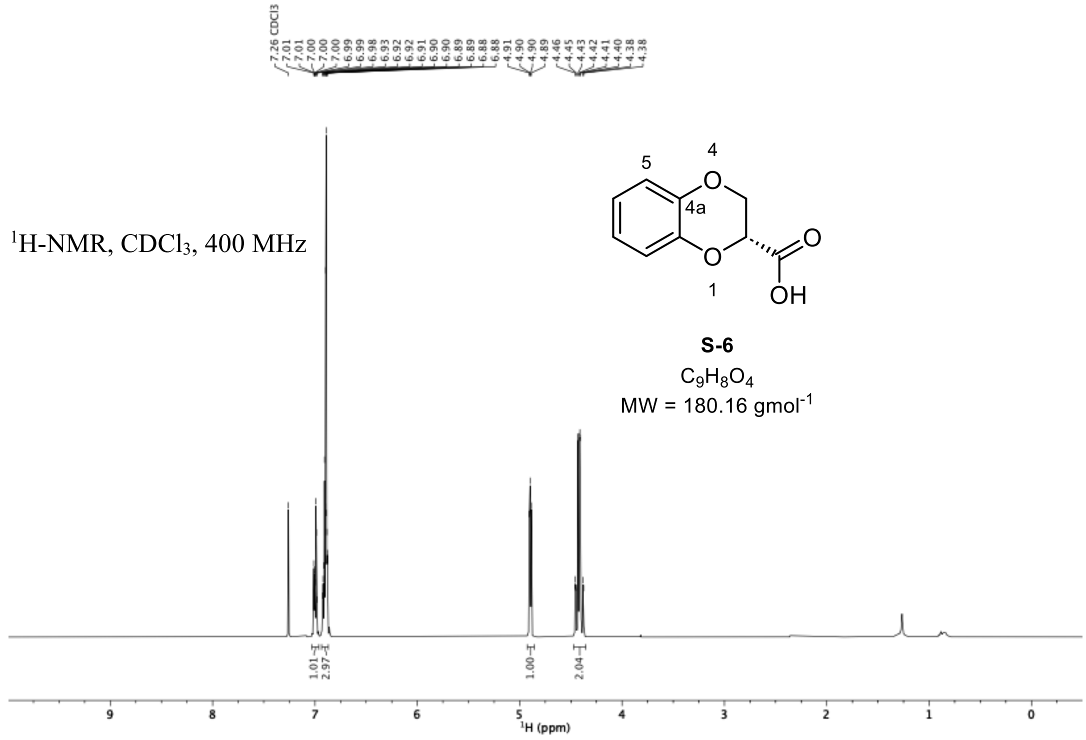

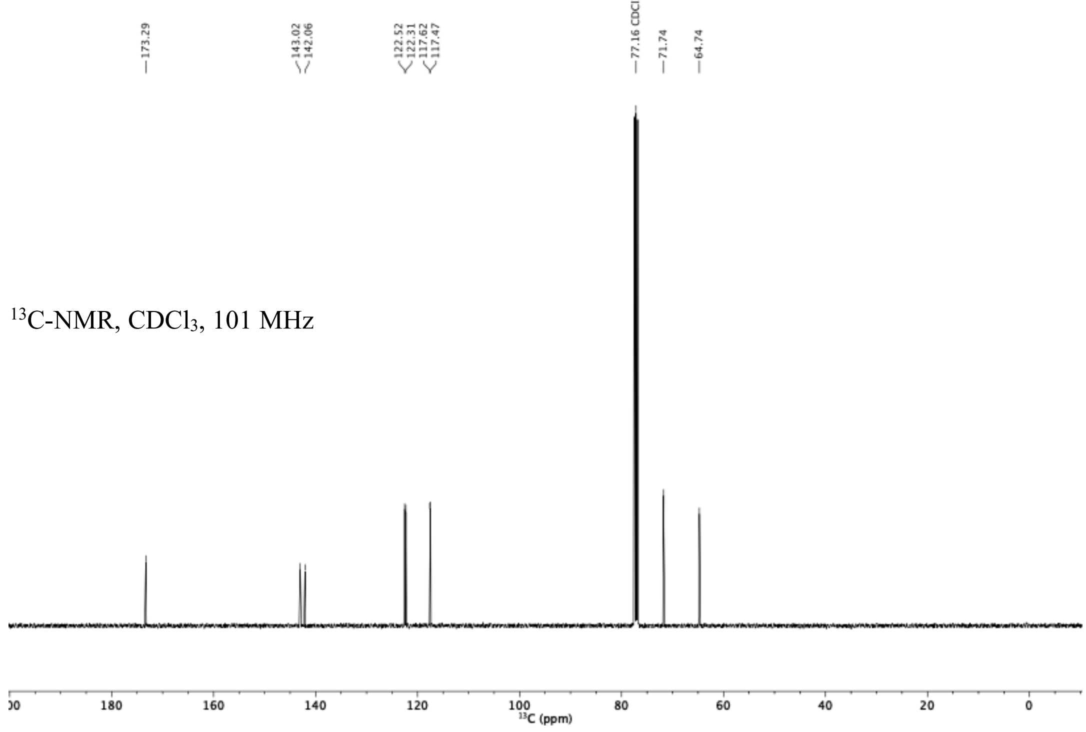

1-Boc-(*R*)-4-(1,4-benzodioxane-2-carbonyl)piperazine (7)

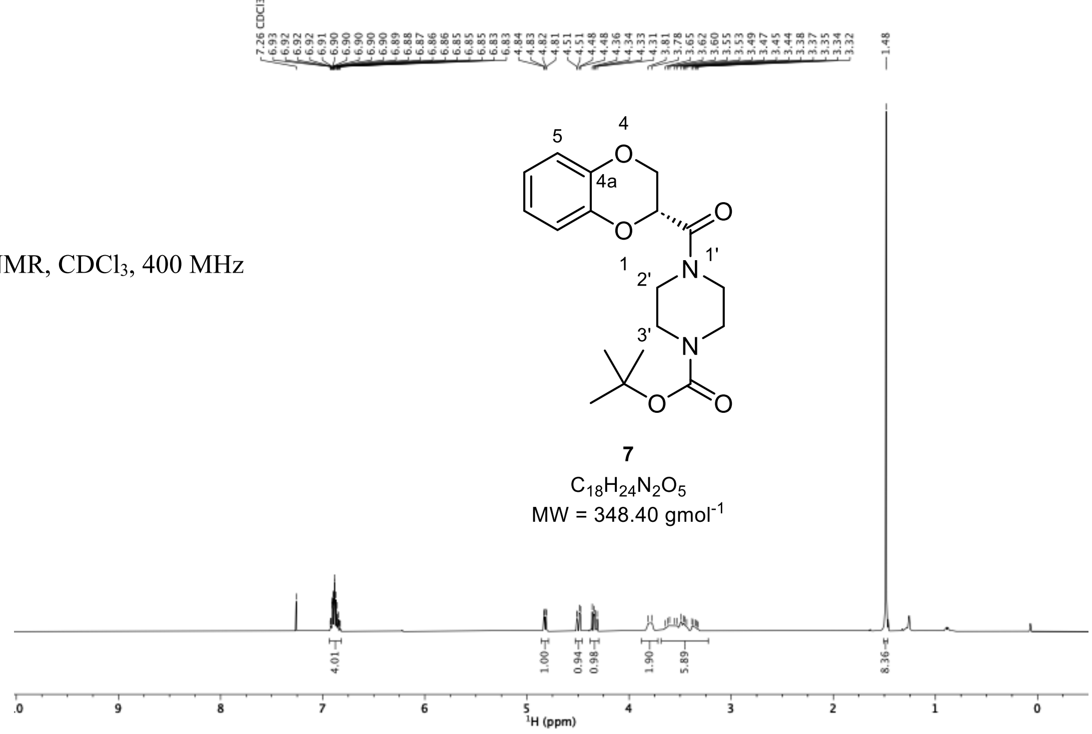

^{1}H-NMR, $CDCl_3$, 400 MHz

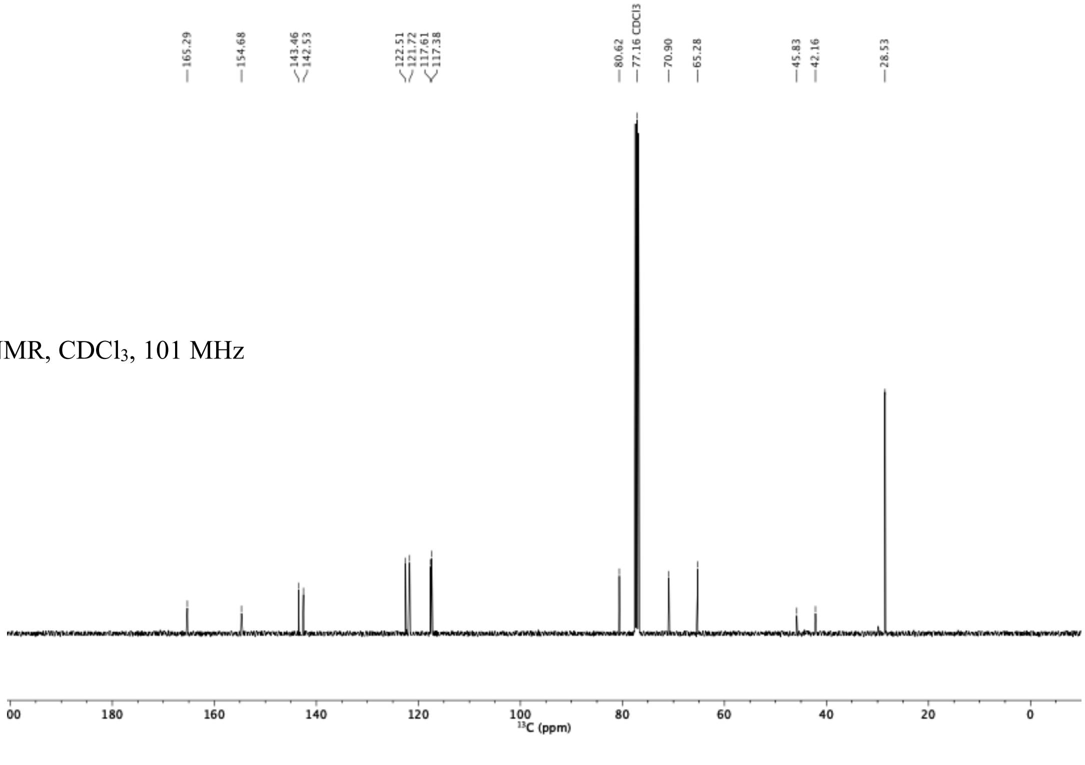

^{13}C-NMR, $CDCl_3$, 101 MHz

(*R*)-4-(1,4-Benzodioxane-2-carbonyl)piperazine (S-7)

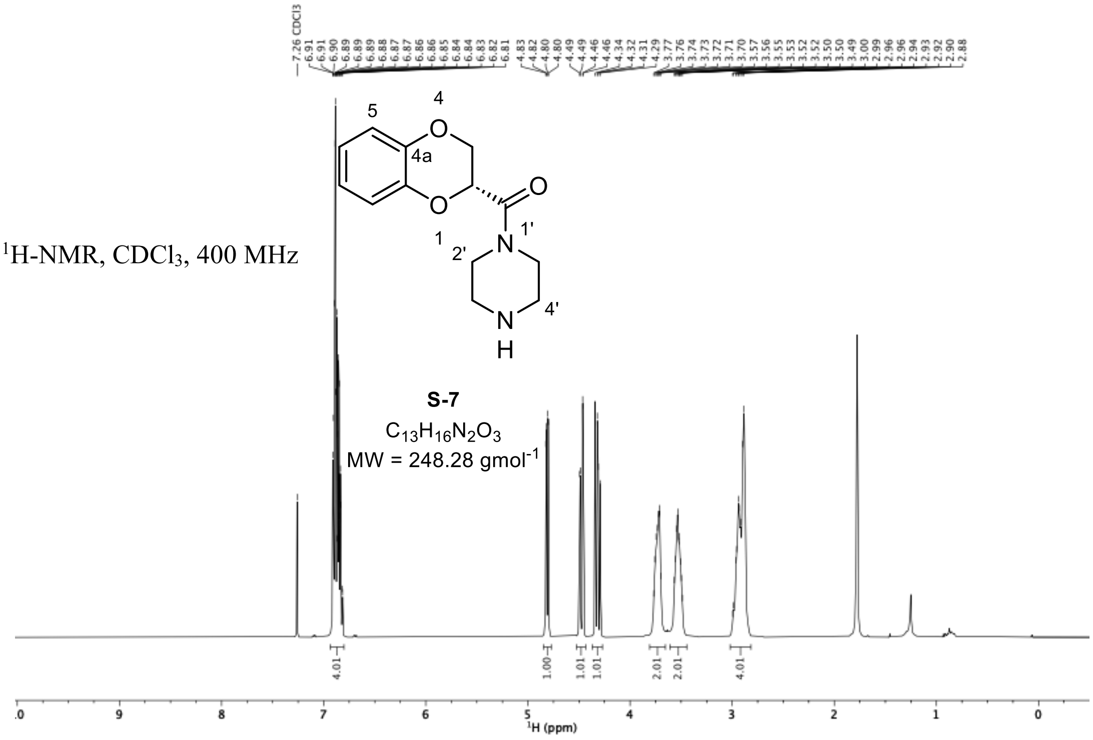

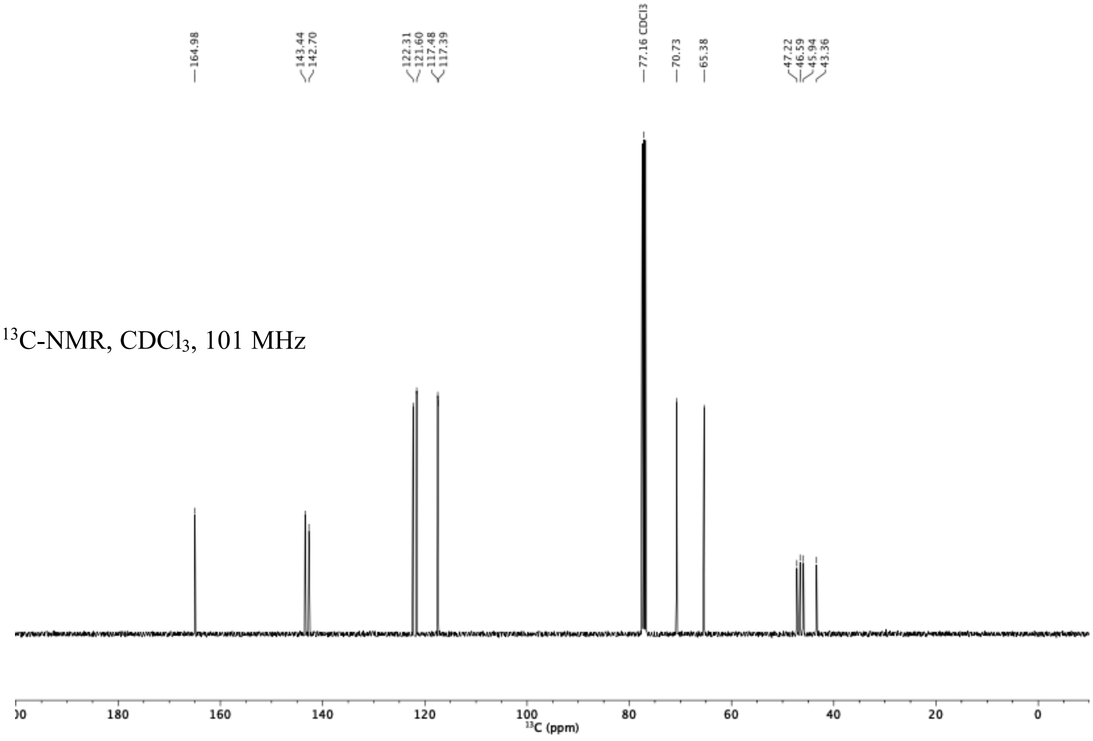

(*R*)-2-(4-(1,4-Benzodioxane-2-carbonyl)piperazin-1-yl)-6,7-dimethoxyquinazolin-4-ammonium chloride (Doxazosin · HCl)

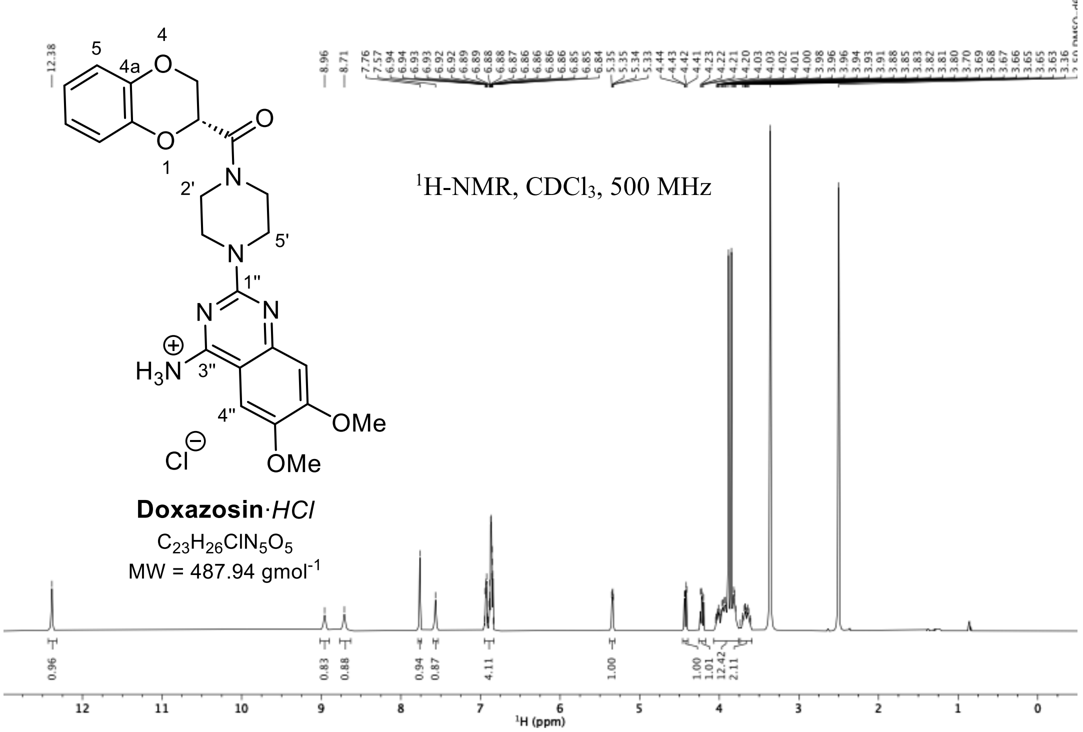

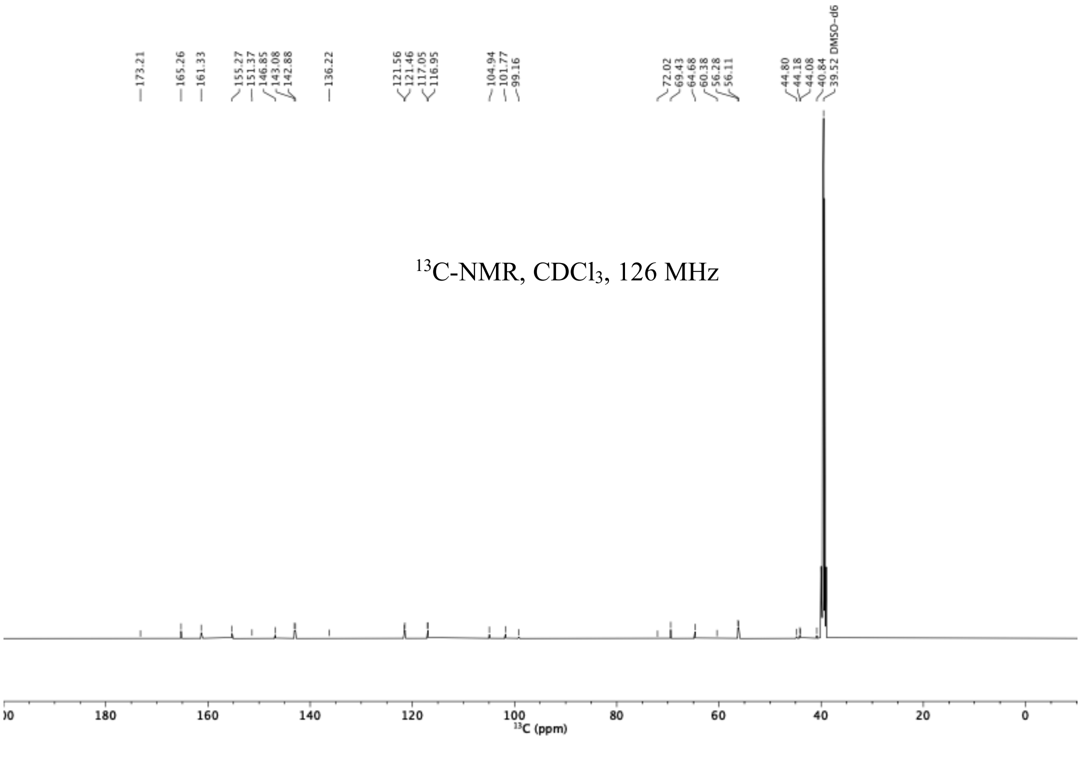

(*R*)-6-Fluorochromane-2-carboxylic acid ((*R*)-S-8)

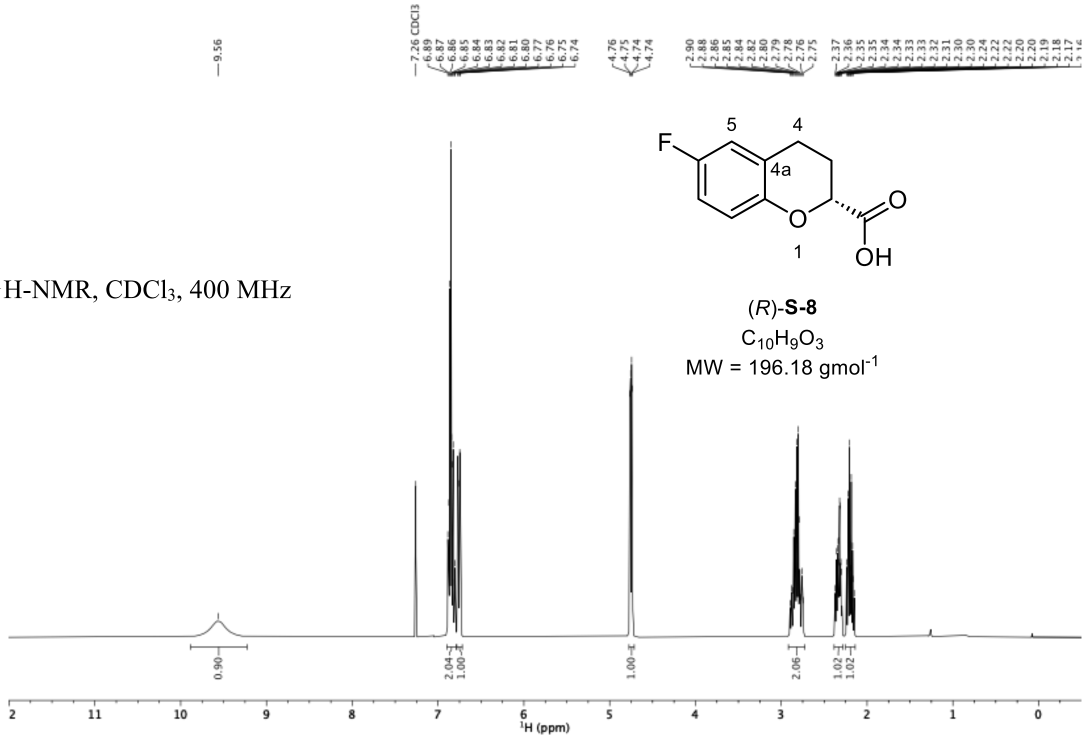

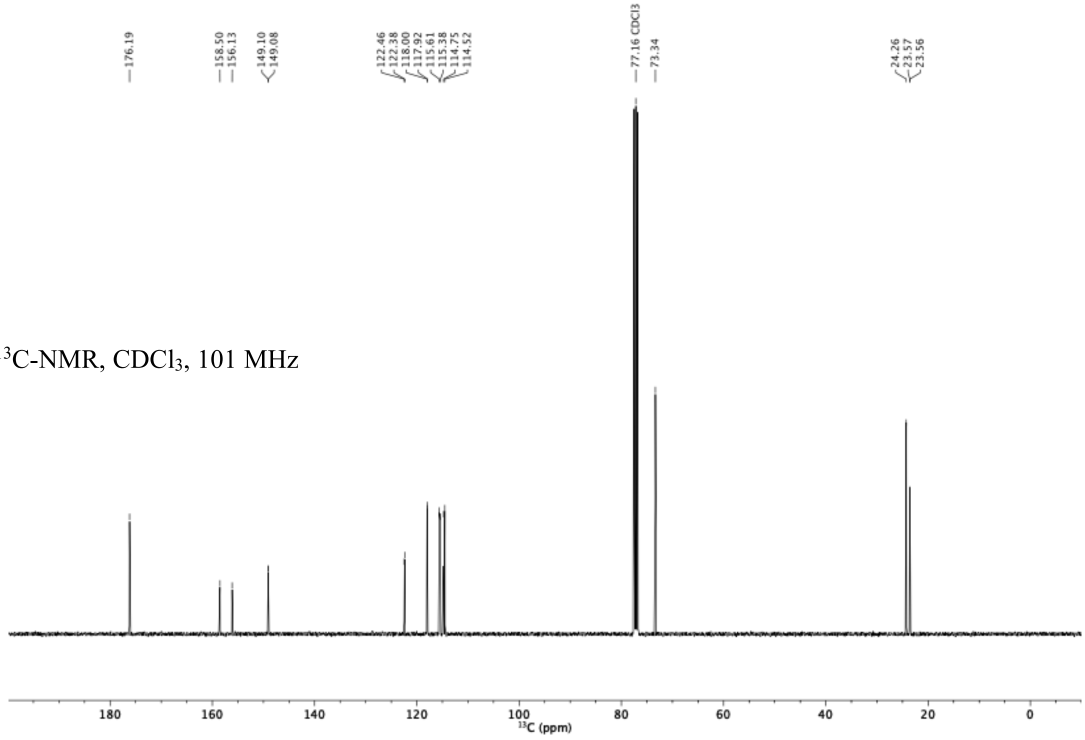

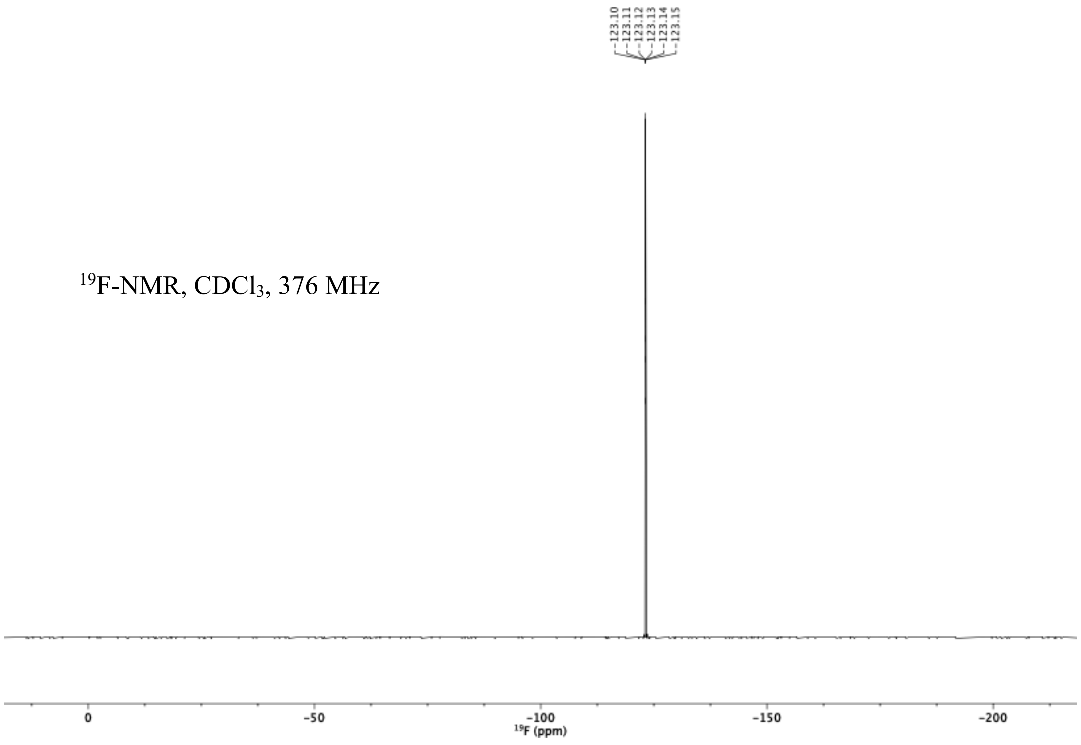
-123.10
-123.11
-123.12
-123.13
-123.14
-123.15
19F-NMR, CDCl3, 376 MHz
0
-50
-100
-150
-200
19F (ppm)

(*R*)-6-Fluorochromane-2-carbaldehyde ((*R*)-S-9)

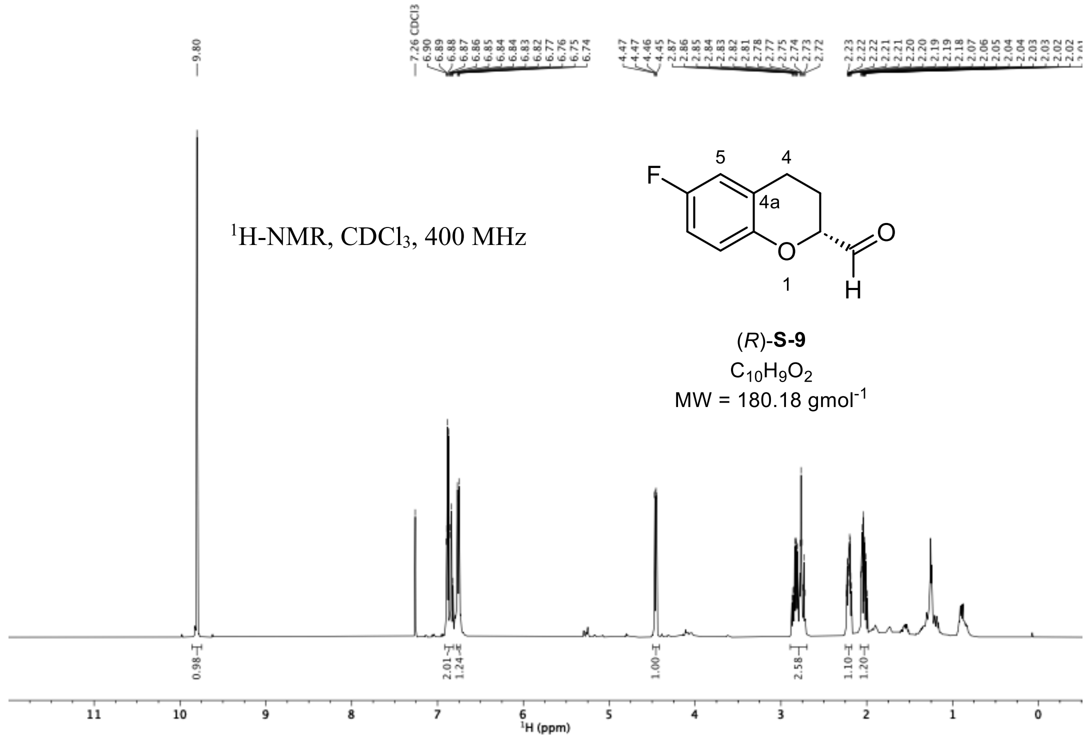

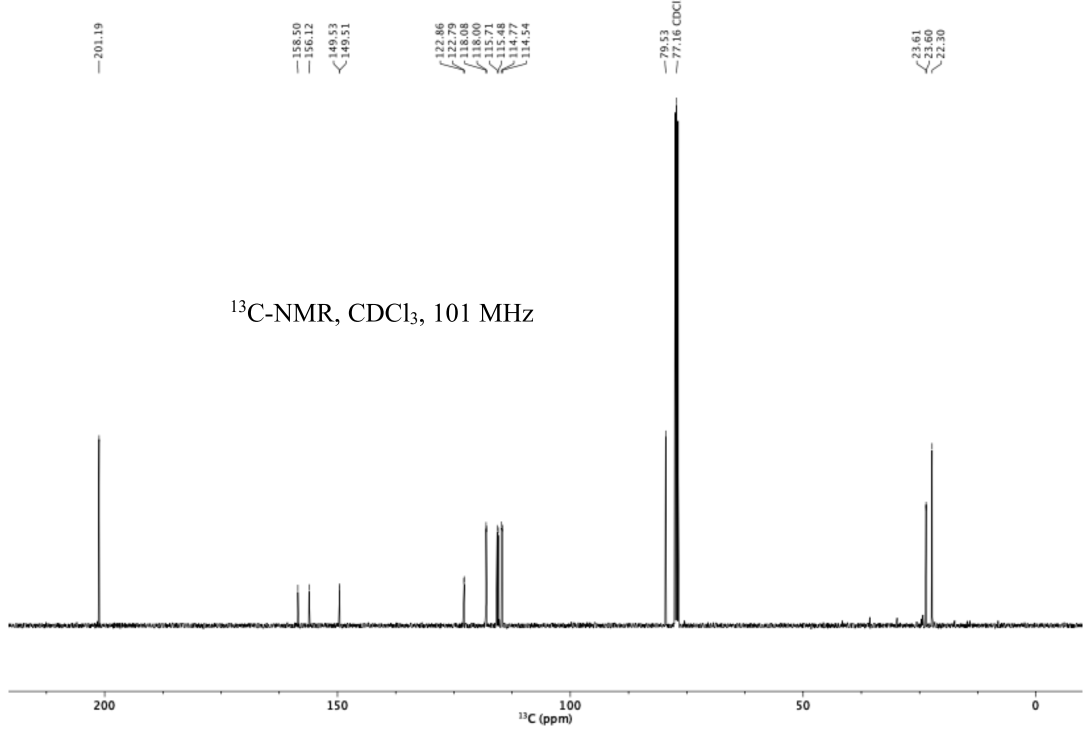

^{19}F-NMR, $CDCl_3$, 376 MHz

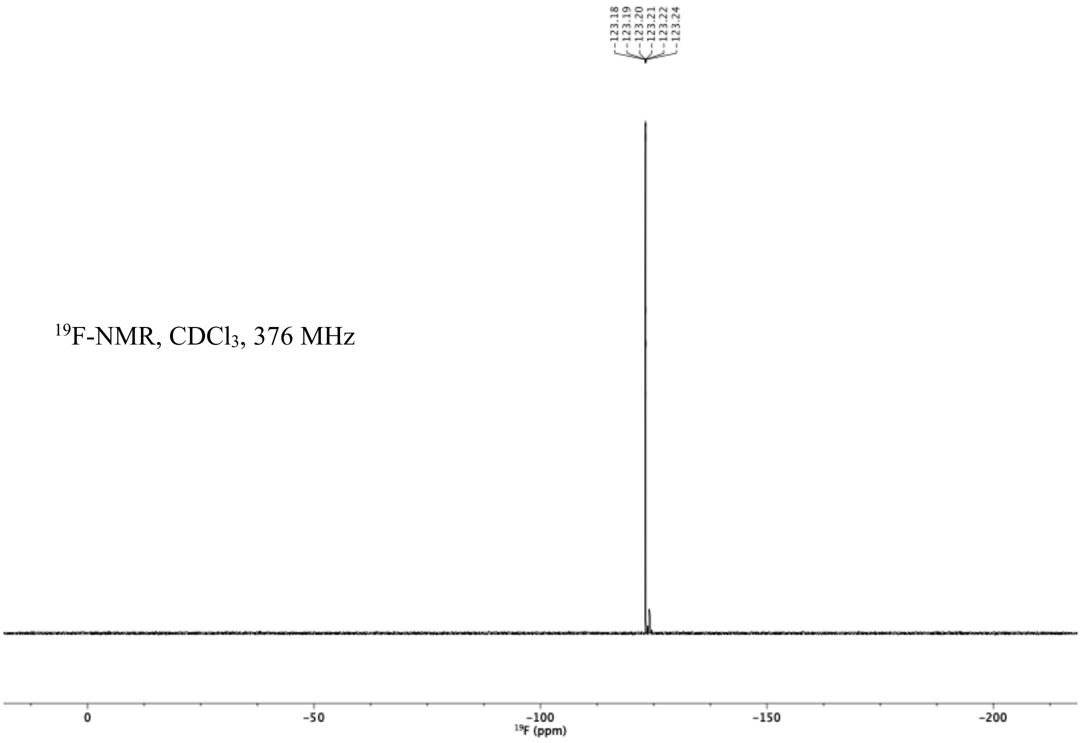

(*R*)-6-Fluoro-2-((*S*)-oxiran-2-yl)chromane ((2*R*,2'*S*)-10)

^{1}H-NMR, $CDCl_3$, 400 MHz

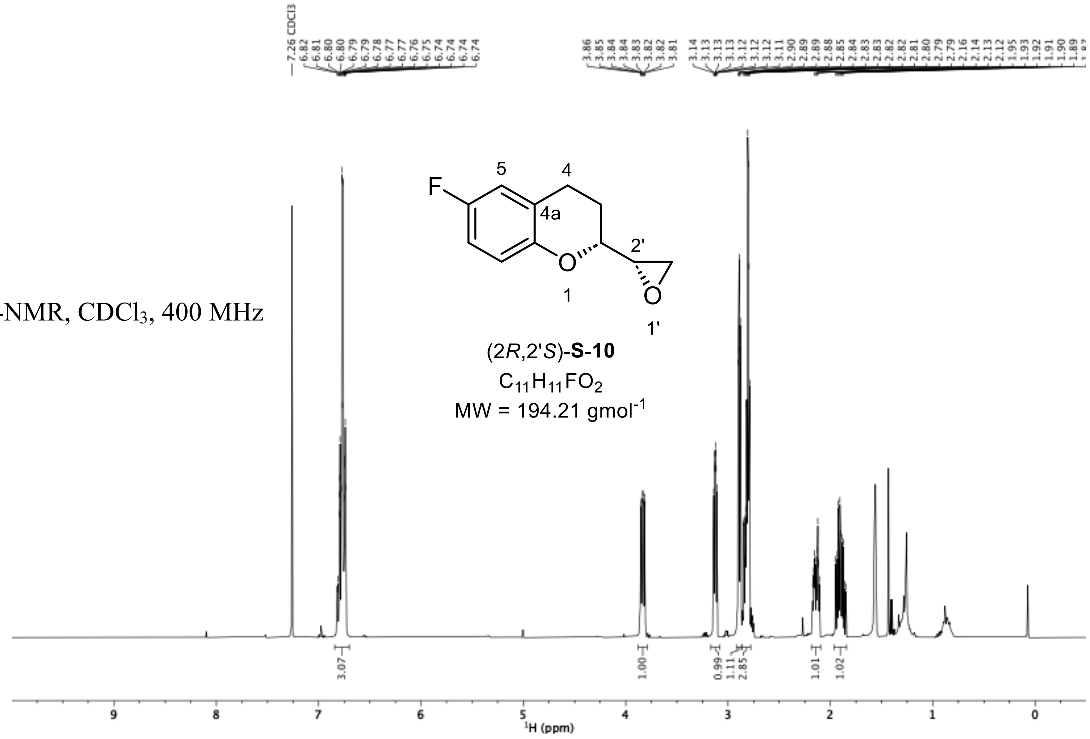

^{13}C-NMR, $CDCl_3$, 101 MHz

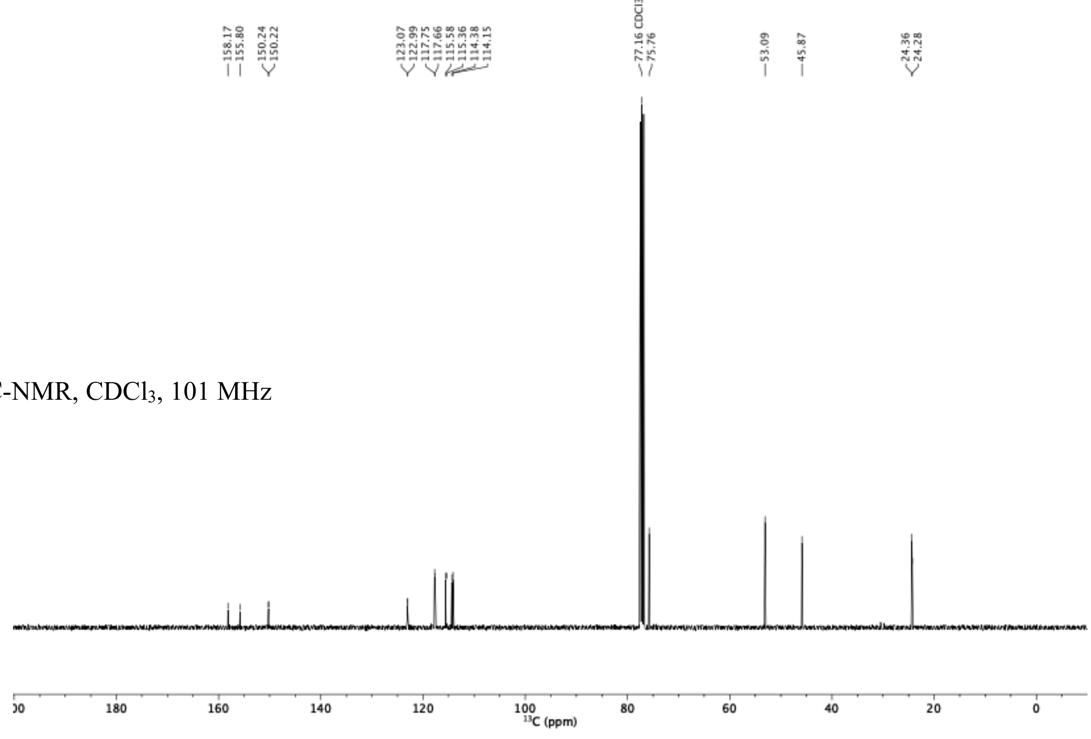

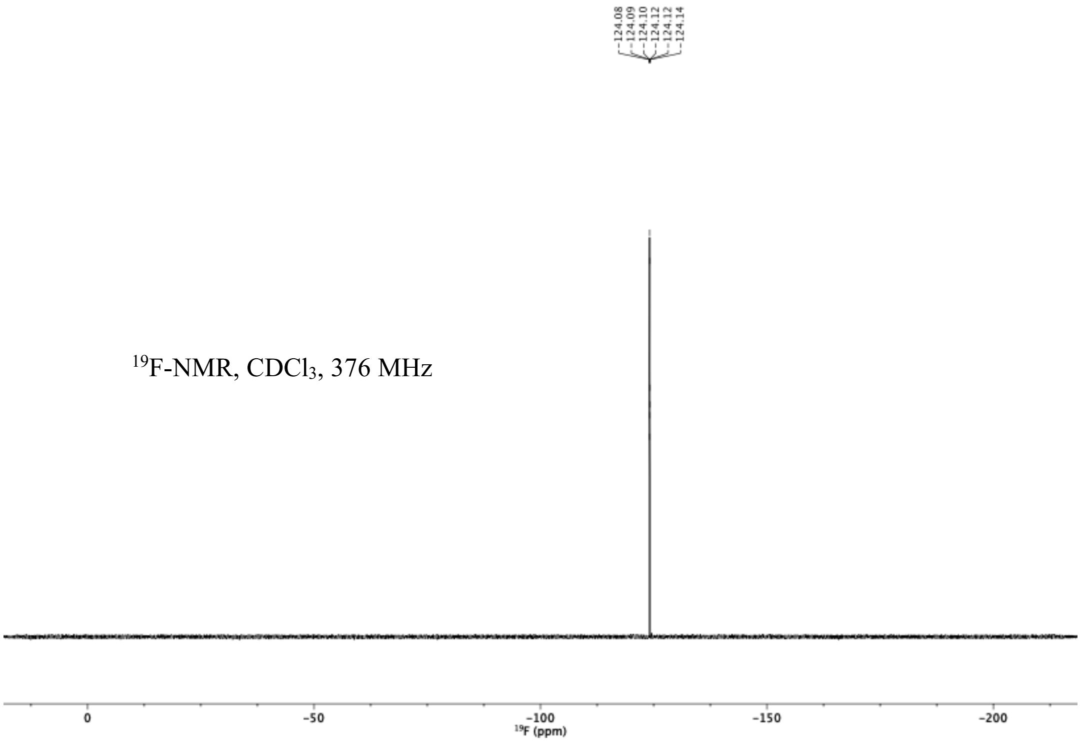
-124.08
-124.09
-124.10
-124.12
-124.12
-124.14
19F-NMR, CDCl3, 376 MHz
0
-50
-100
-150
-200
19F (ppm)

(*R*)-6-Fluoro-2-((*R*)-oxiran-2-yl)chromane ((2*R*,2'*R*)-10)

^{1}H-NMR, $CDCl_3$, 400 MHz

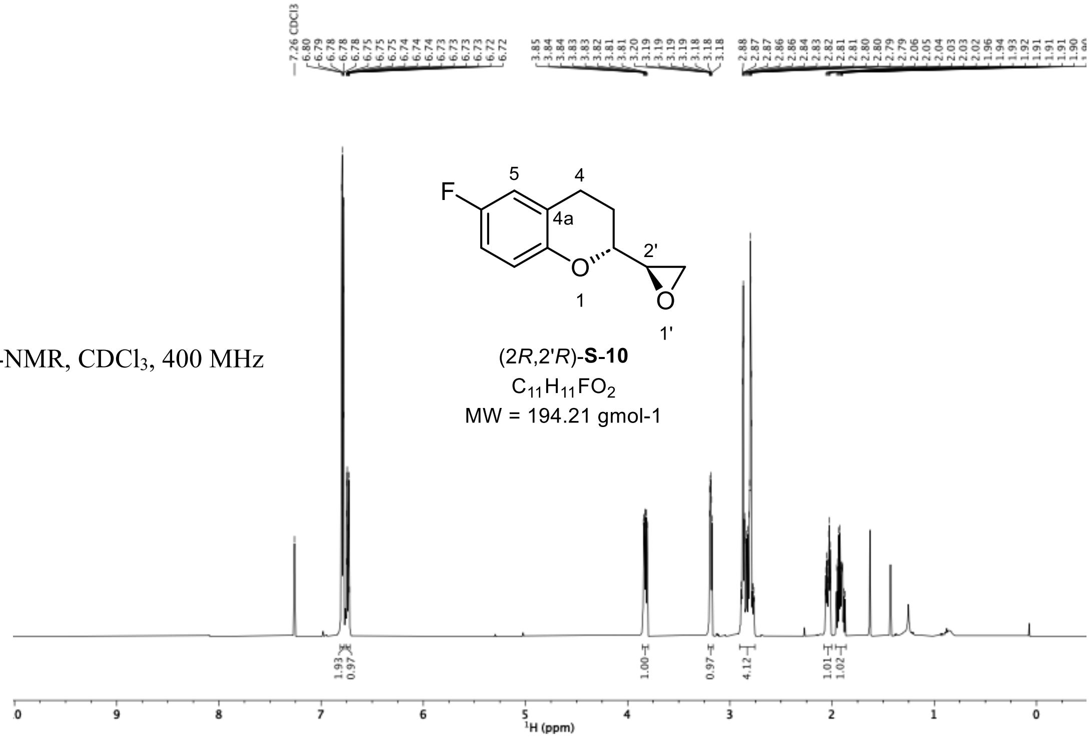

^{13}C-NMR, $CDCl_3$, 101 MHz

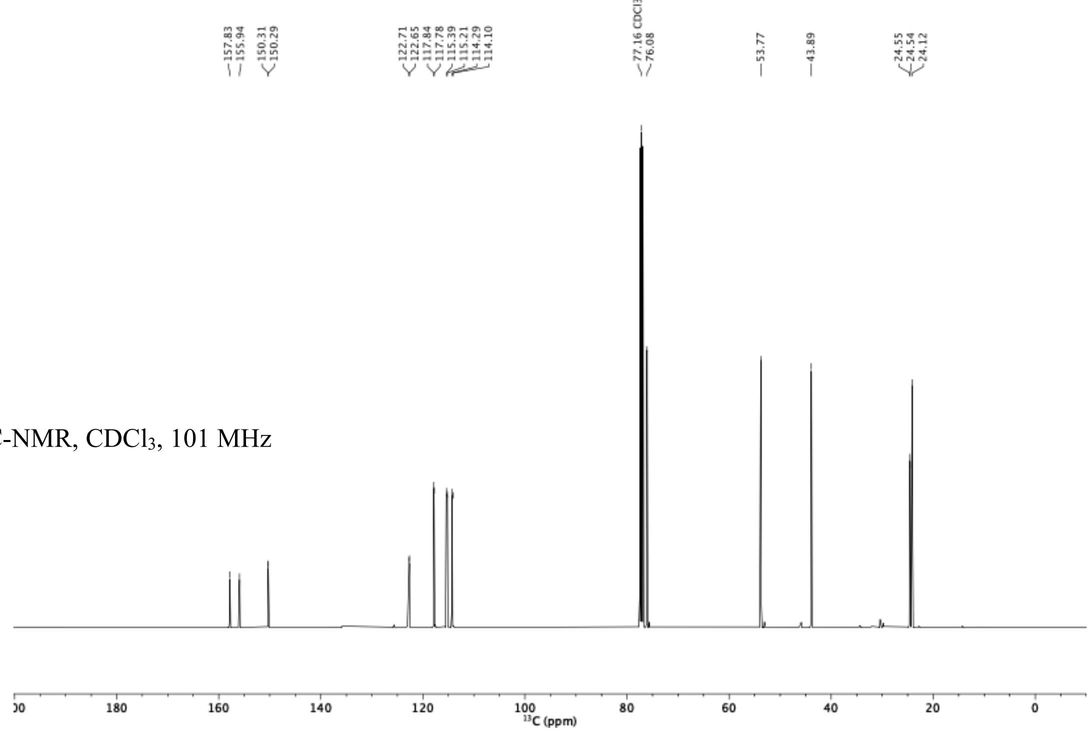

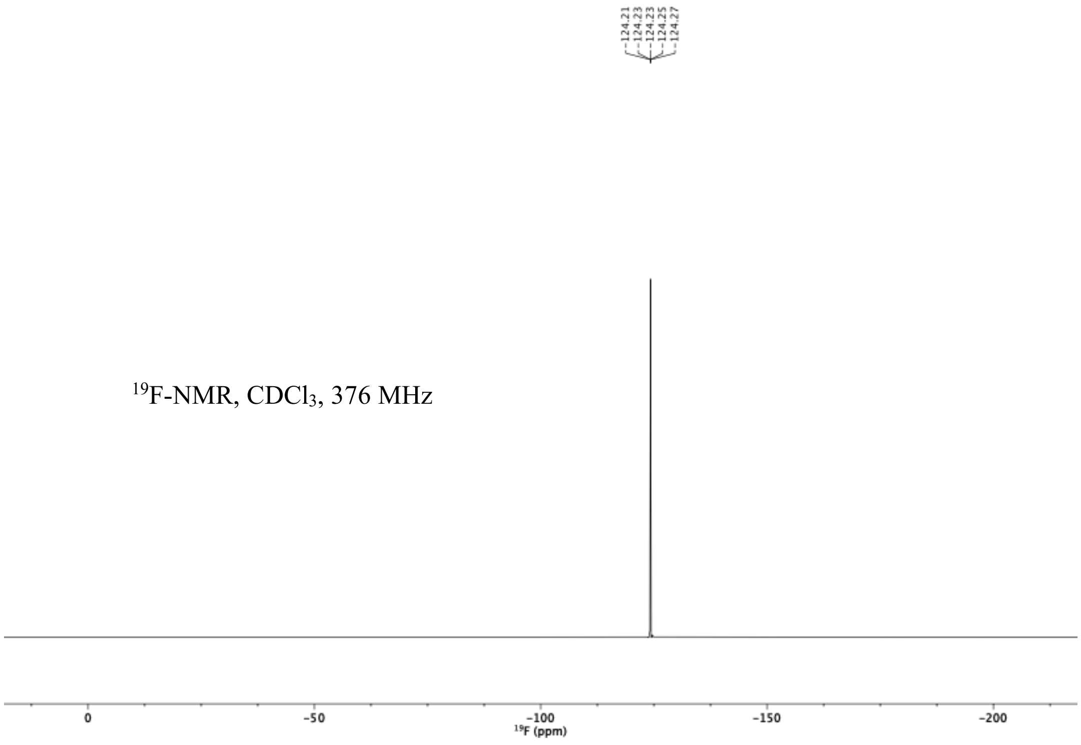
-124.21
-124.23
-124.23
-124.25
-124.27
19F-NMR, CDCl3, 376 MHz
0
-50
-100
-150
-200
19F (ppm)

(*S*)-6-Fluorochromane-2-carboxylic acid ((*S*)-S-8)

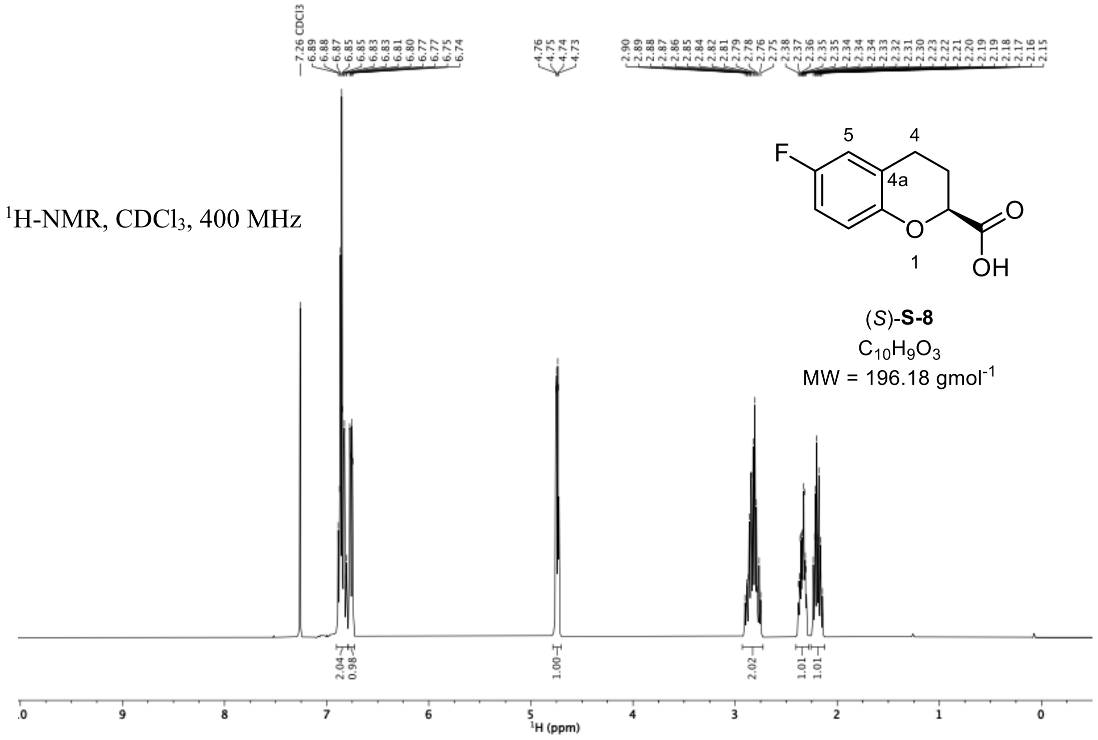

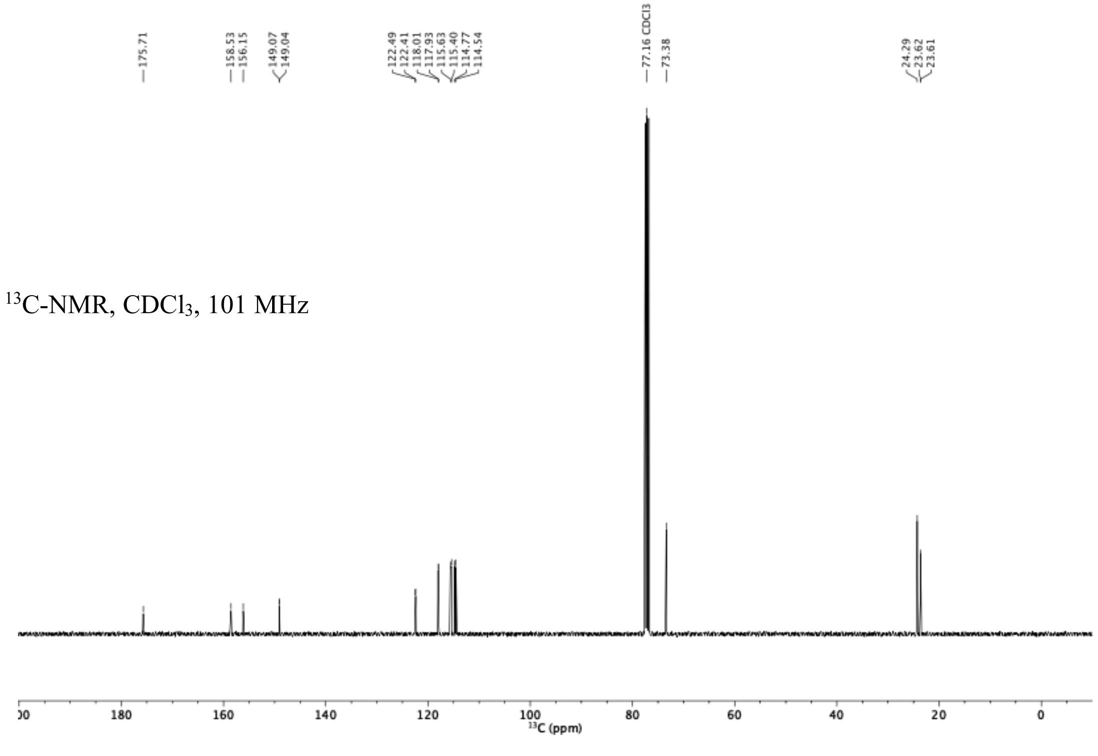

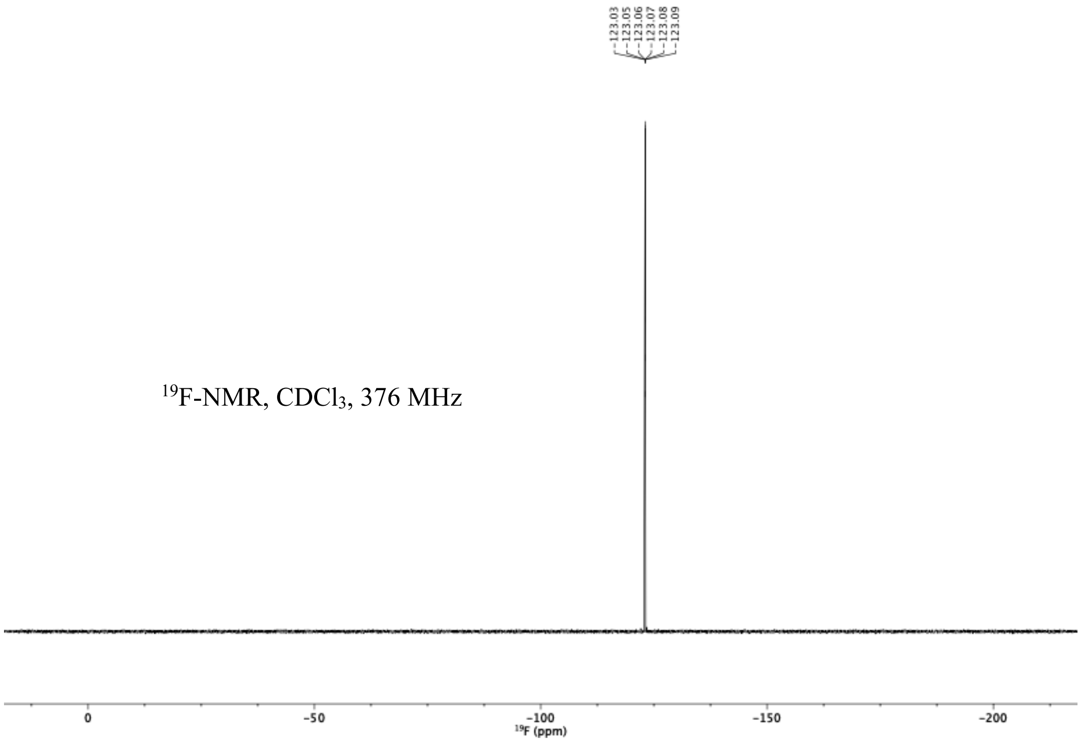
-123.03
-123.05
-123.06
-123.07
-123.08
-123.09
19F-NMR, CDCl3, 376 MHz
0
-50
-100
-150
-200
19F (ppm)

(*S*)-6-Fluorochromane-2-carbaldehyde ((*S*)-S-9)

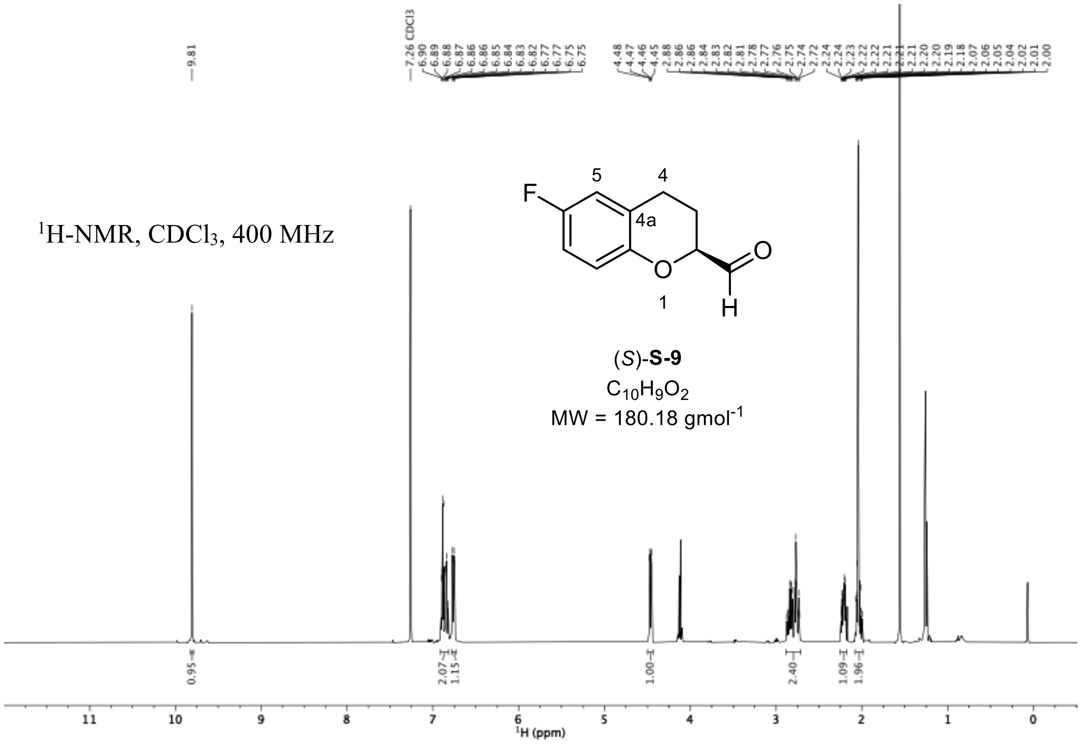

(*S*)-6-Fluoro-2-((*R*)-oxiran-2-yl)chromane ((2*S*,2'*R*)-S-10)

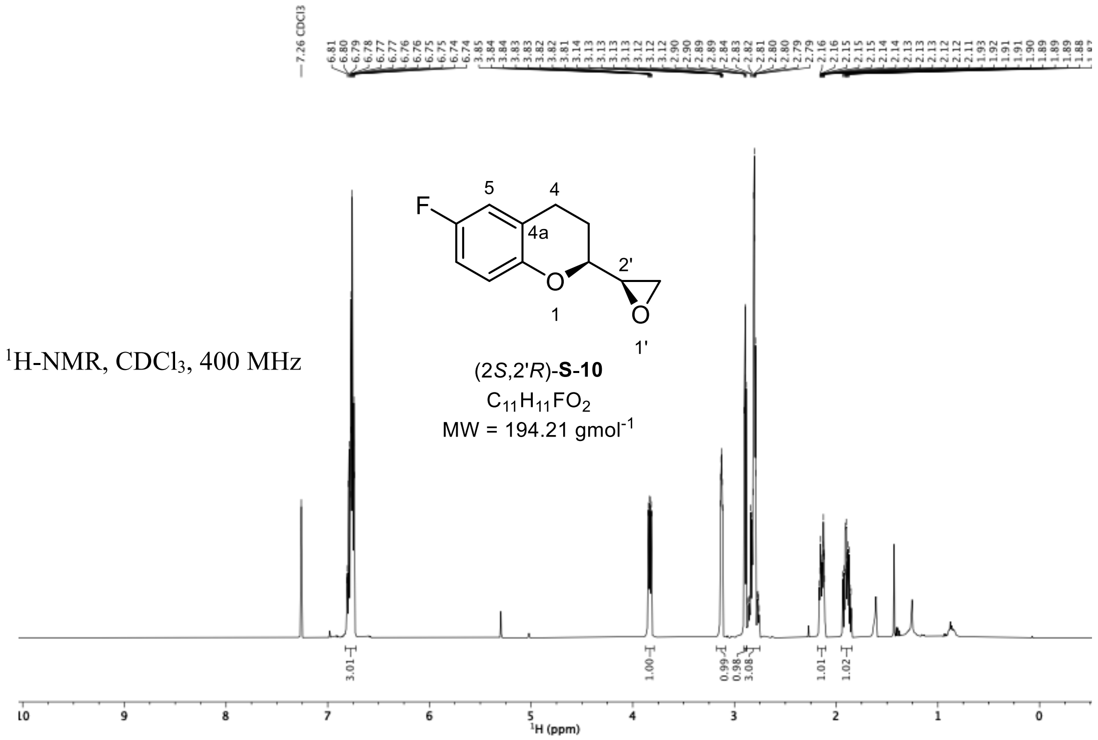

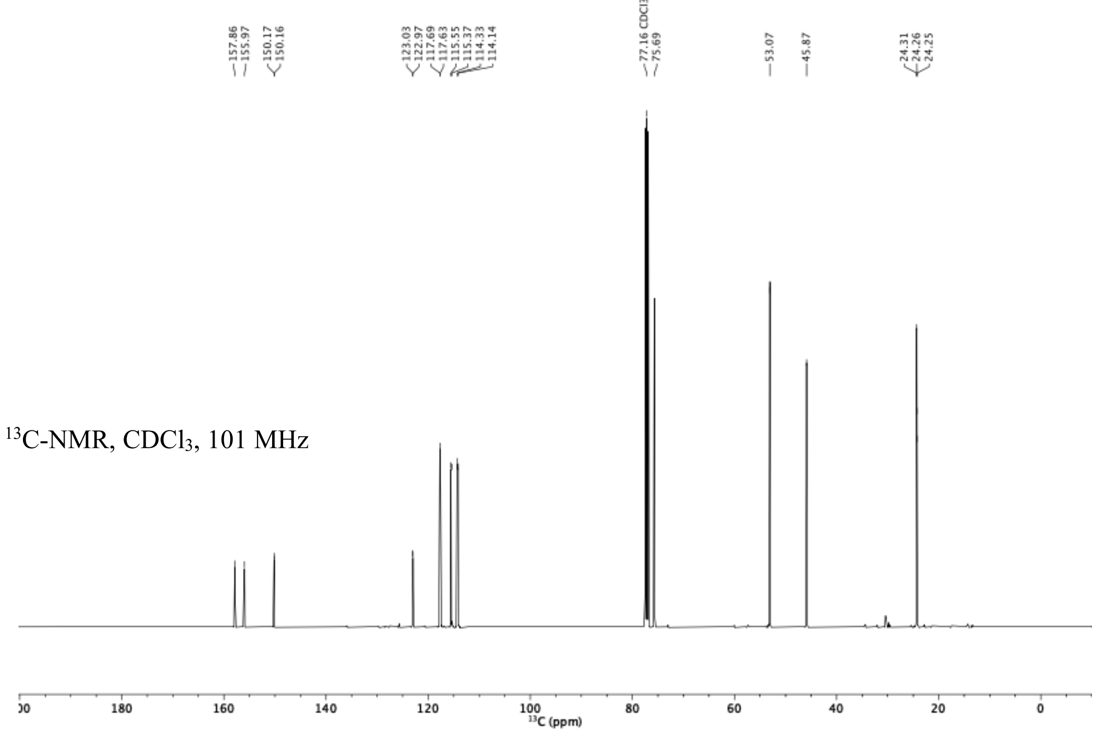

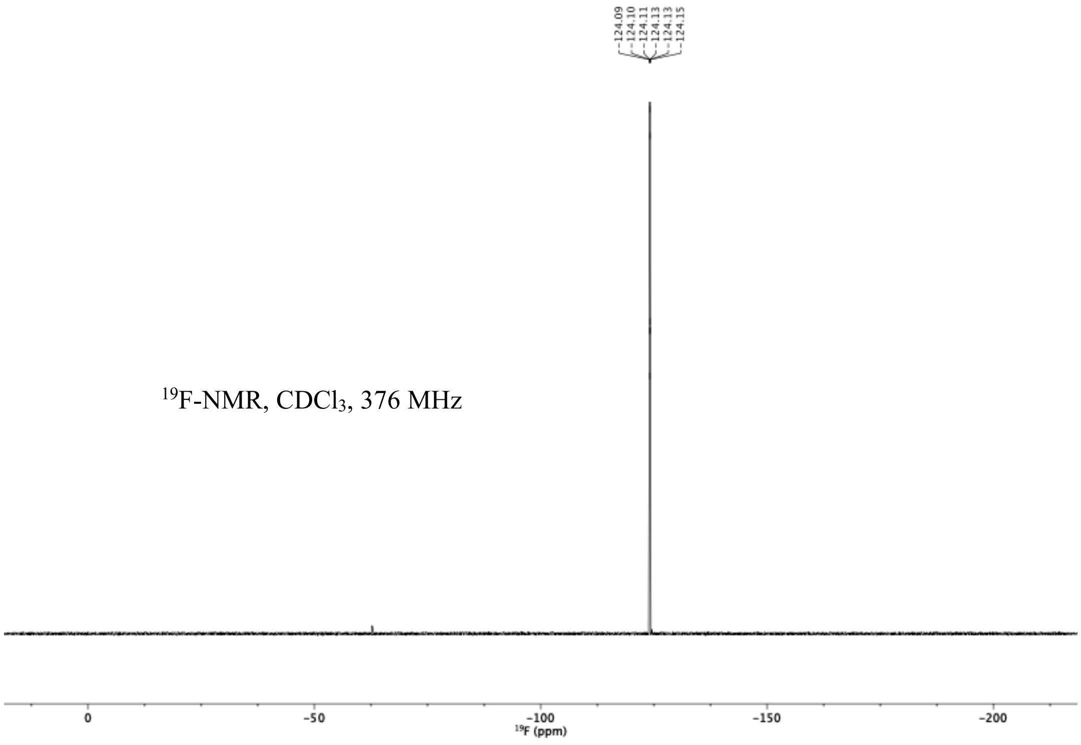
-124.09
-124.10
-124.11
-124.13
-124.13
-124.15
19F-NMR, CDCl3, 376 MHz
0
-50
-100
-150
-200
19F (ppm)

(*S*)-6-Fluoro-2-((*S*)-oxiran-2-yl)chromane ((2*S*,2'*S*)-S-10)

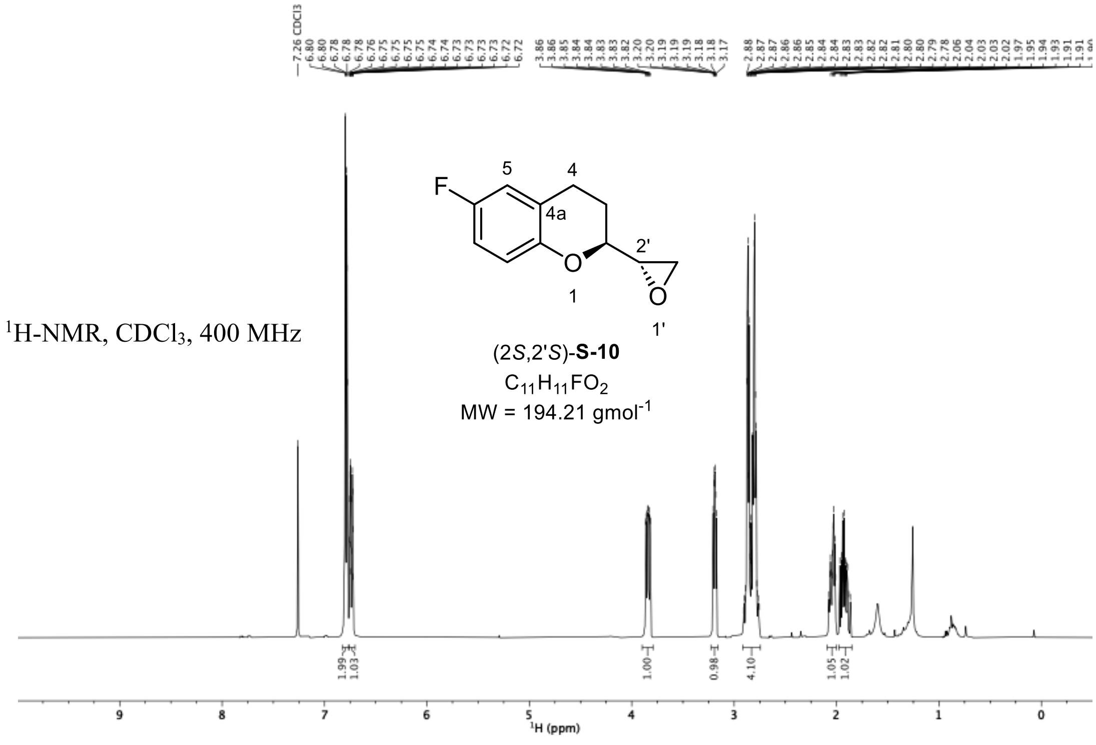

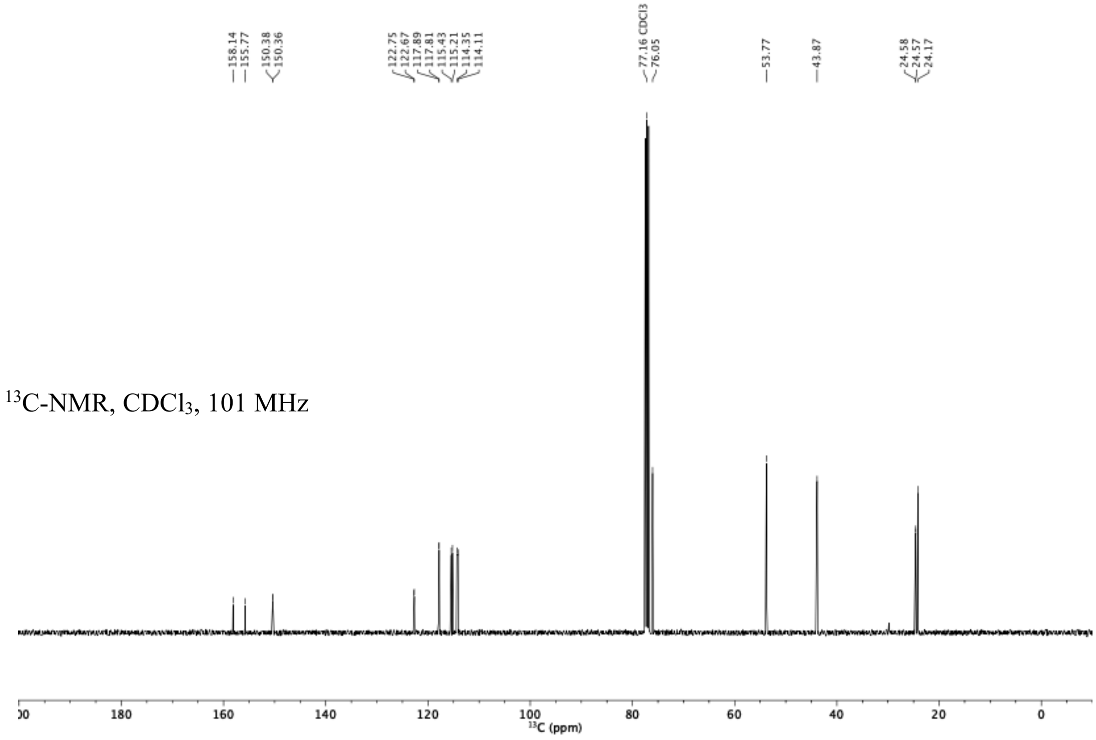

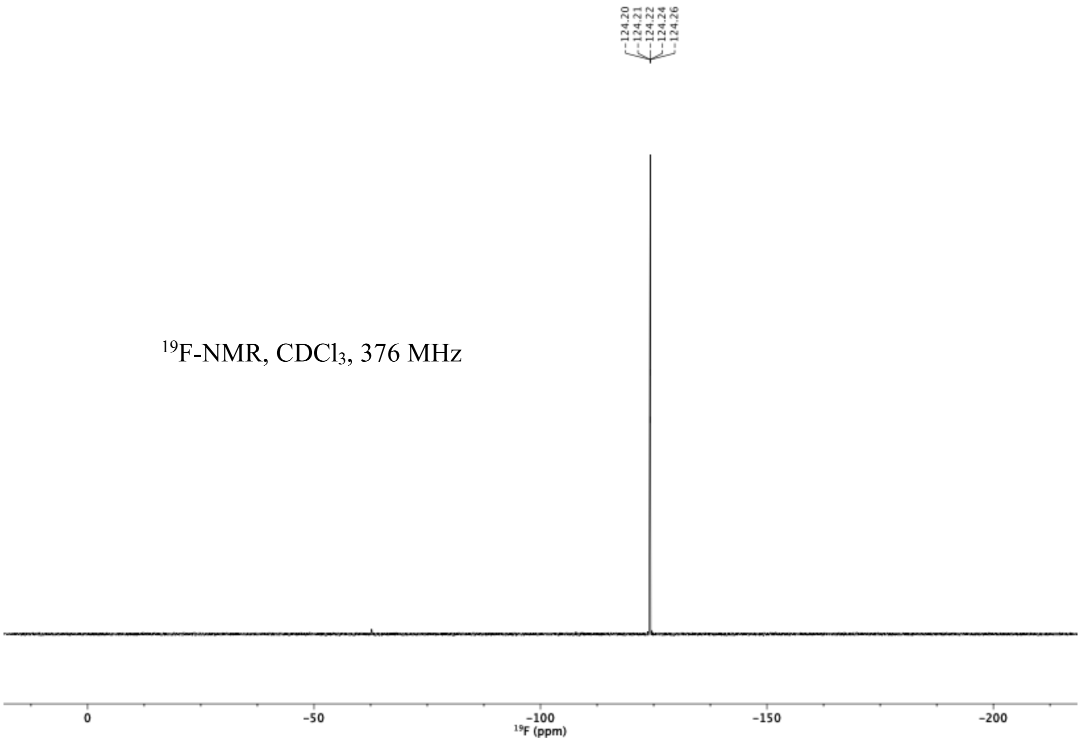
-124.20
-124.21
-124.22
-124.24
-124.26
19F-NMR, CDCl3, 376 MHz
0
-50
-100
-150
-200
19F (ppm)

(*R*)-2-(Benzylamino)-2-((*S*)-6-fluorochroman-2-yl)ethan-1-ol (9

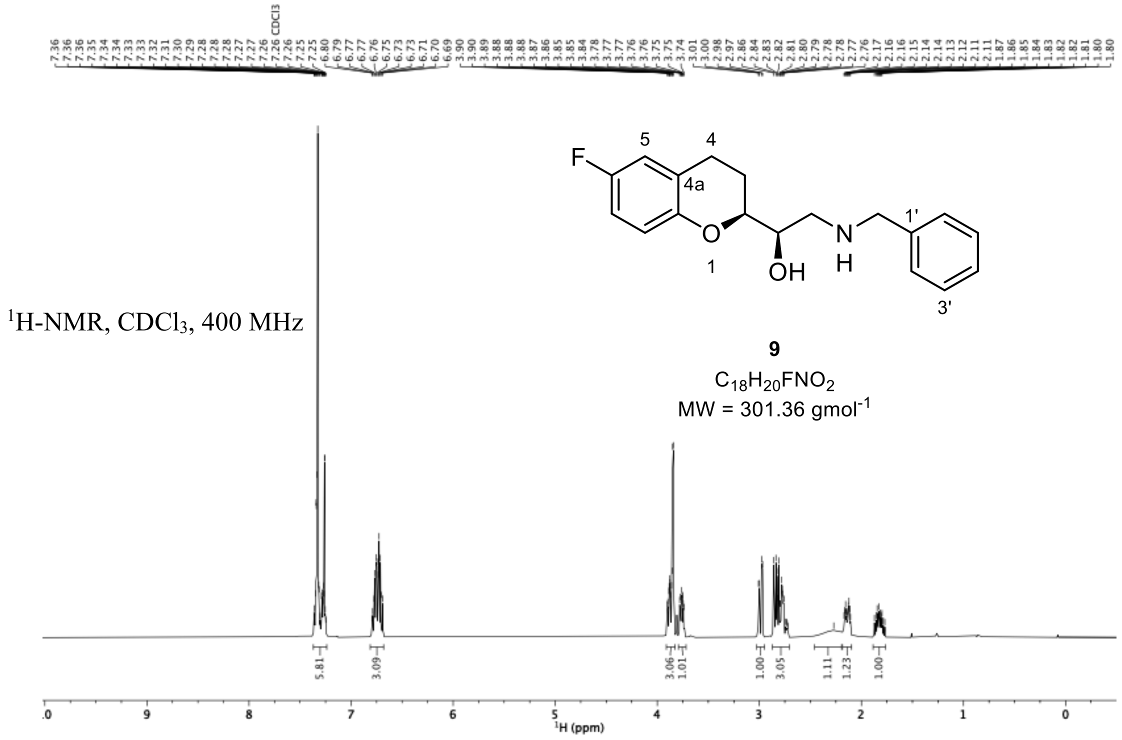

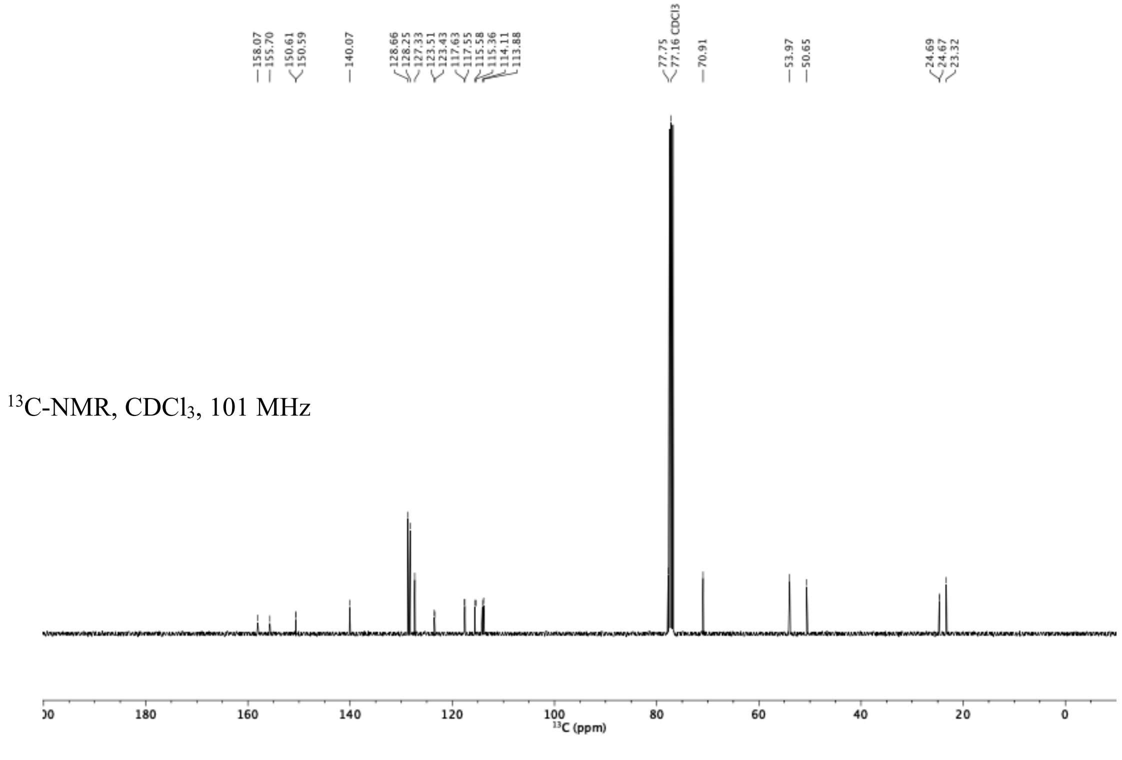

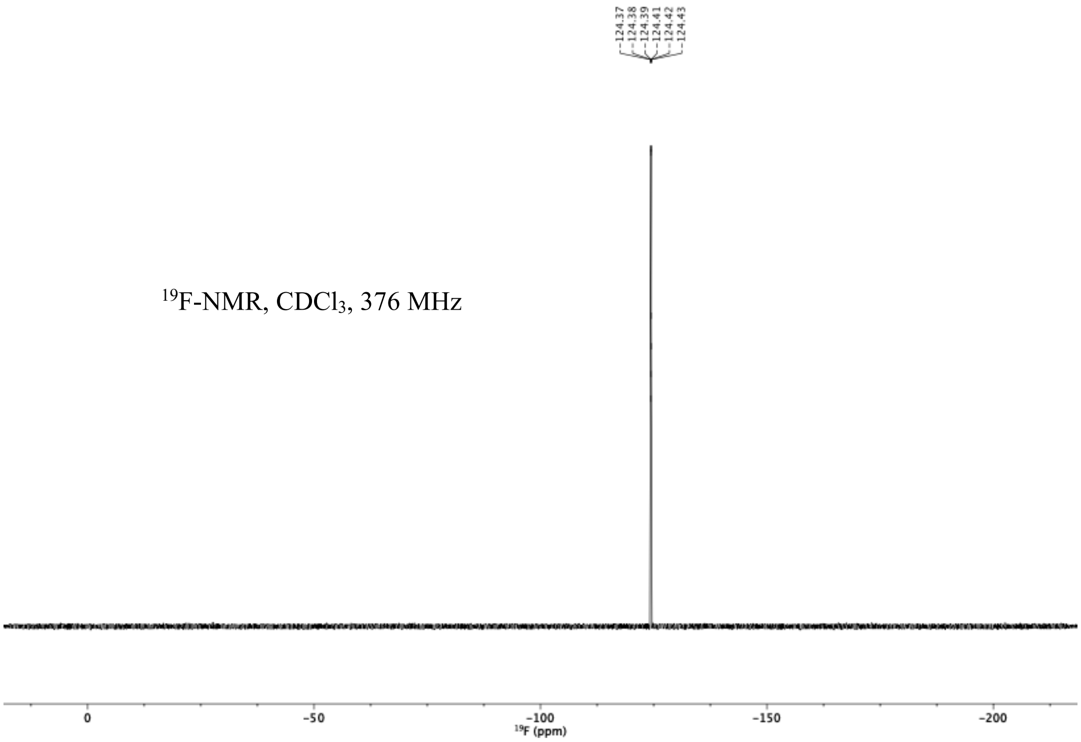
-124.37
-124.38
-124.39
-124.41
-124.42
-124.43
^{19}F-NMR, $CDCl_3$, 376 MHz
0
-50
-100
-150
-200
^{19}F (ppm)

(*R*)-2-(Benzyl((*R*)-2-((*R*)-6-fluorochroman-2-yl)-2-hydroxyethyl)amino)-1-((*S*)-6-fluorochroman-2-yl)ethan-1-ol (S-11)

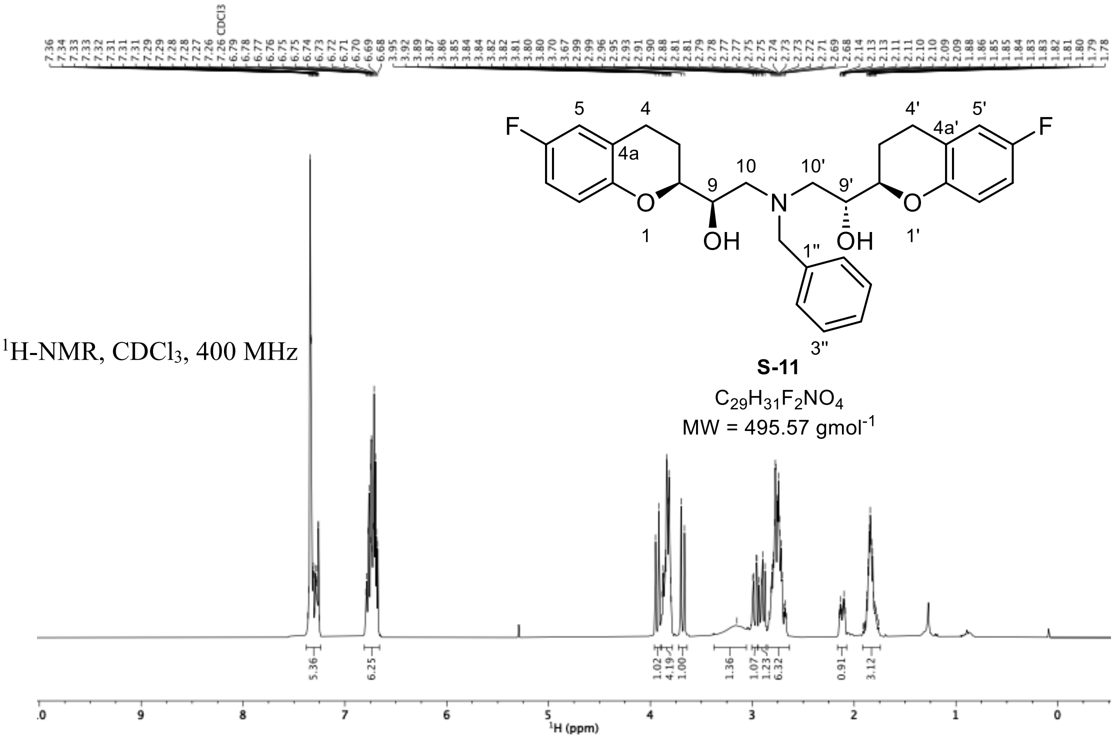

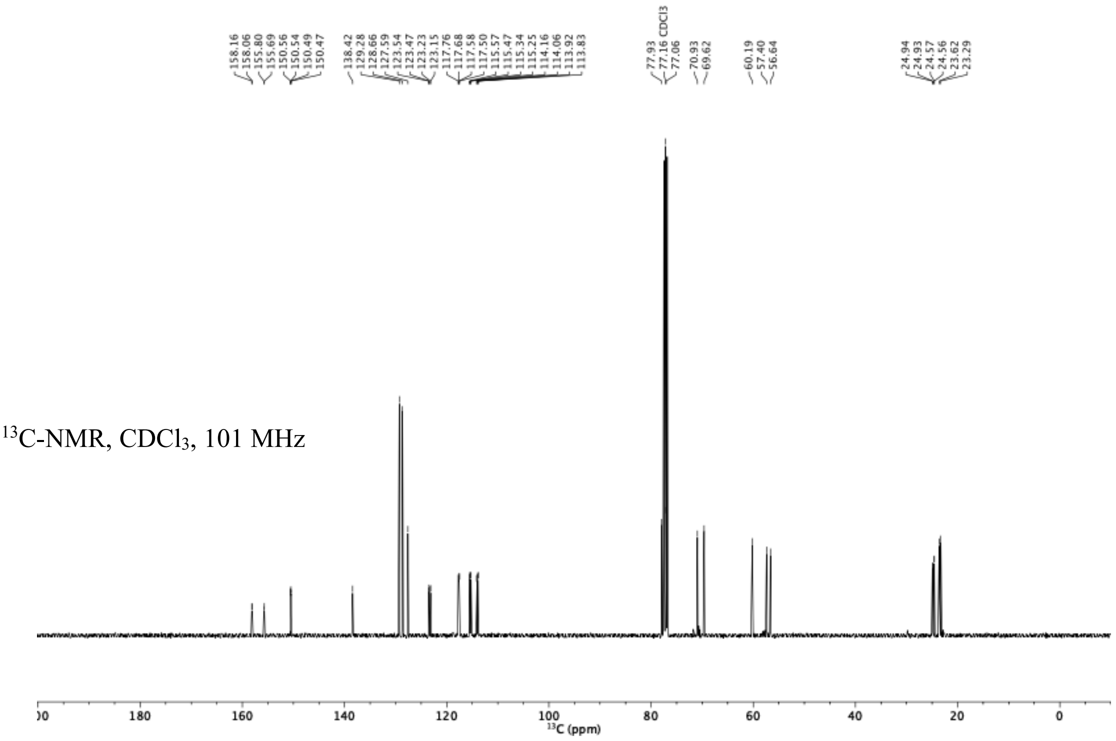

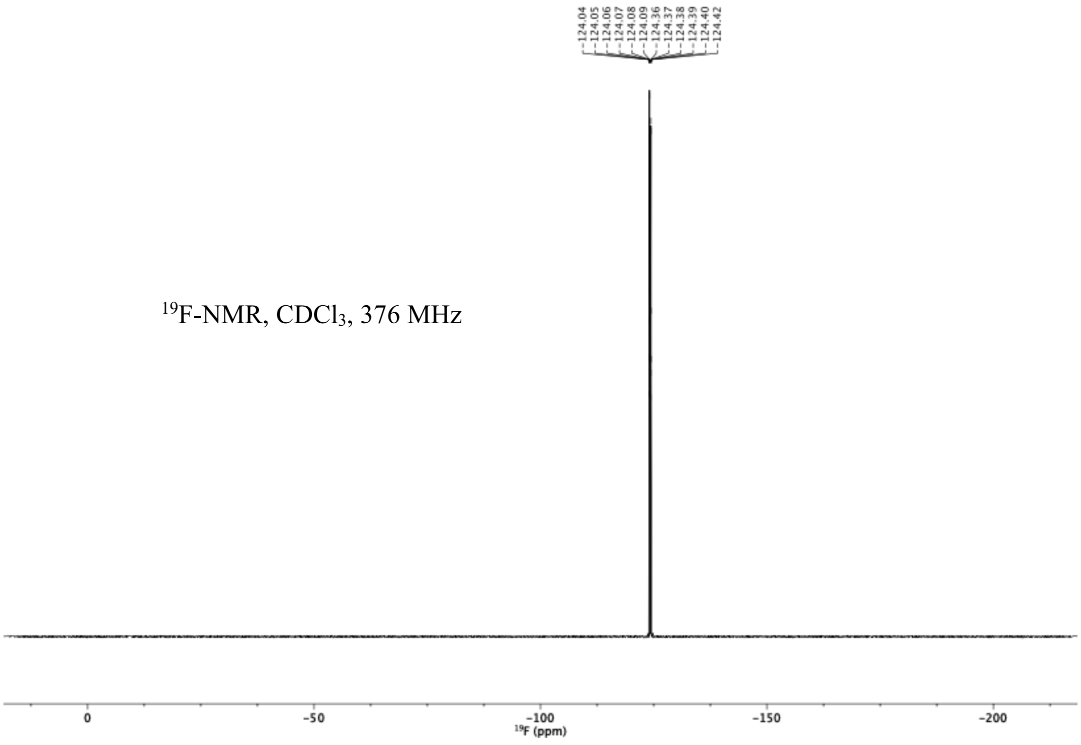
-124.04
-124.05
-124.06
-124.07
-124.08
-124.09
-124.36
-124.37
-124.38
-124.39
-124.40
-124.42
^{19}F-NMR, $CDCl_3$, 376 MHz
0
-50
-100
-150
-200
^{19}F (ppm)

(*R*)-2-((*R*)-6-Fluorochroman-2-yl)-*N*-((*R*)-2-((*S*)-6-fluorochroman-2-yl)-2-hydroxyethyl)-2-hydroxyethan-1-ammonium chloride ((*S*, *R*, *R*, *R*)Nebivolol · *HCl*)

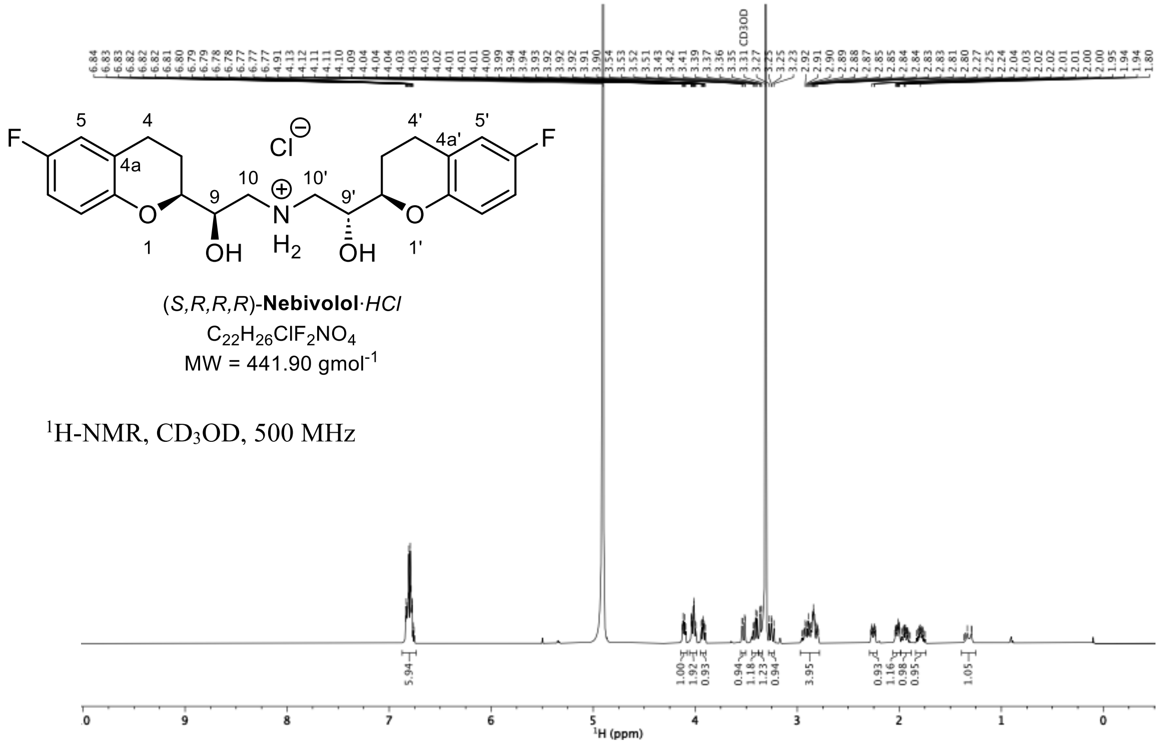

(*S,R,R,R*)-**Nebivolol**·*HCl*

$C_{22}H_{26}ClF_2NO_4$

MW = 441.90 $gmol^{-1}$

^{1}H-NMR, CD_3OD, 500 MHz

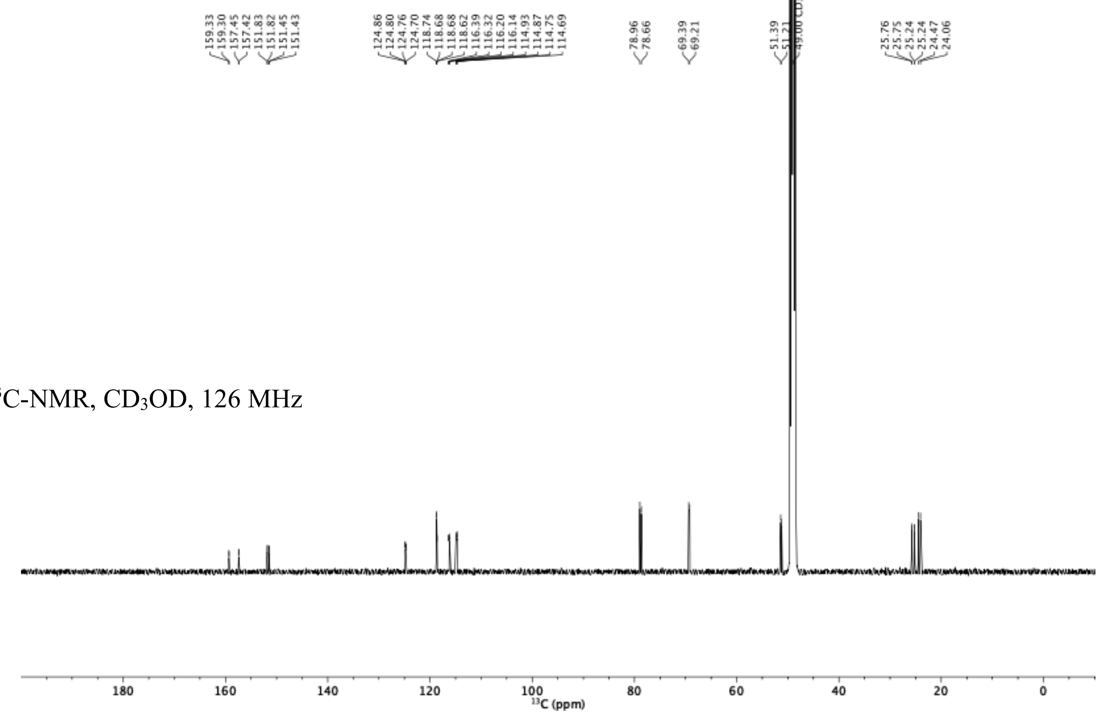

^{13}C-NMR, CD_3OD, 126 MHz

^{19}F-NMR, CD_3OD, 376 MHz

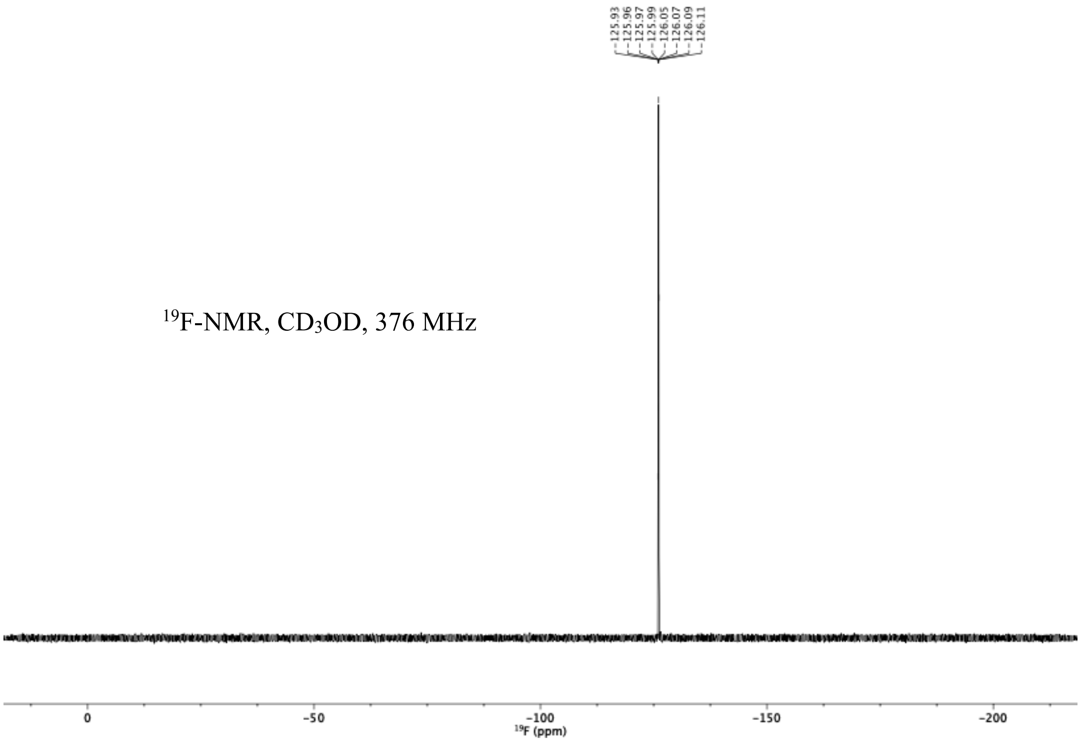

(*S*)-6-Fluoro-4-oxochromane-2-carboxamide (11)

^{1}H-NMR, DMSO-d$_6$, 400 MHz

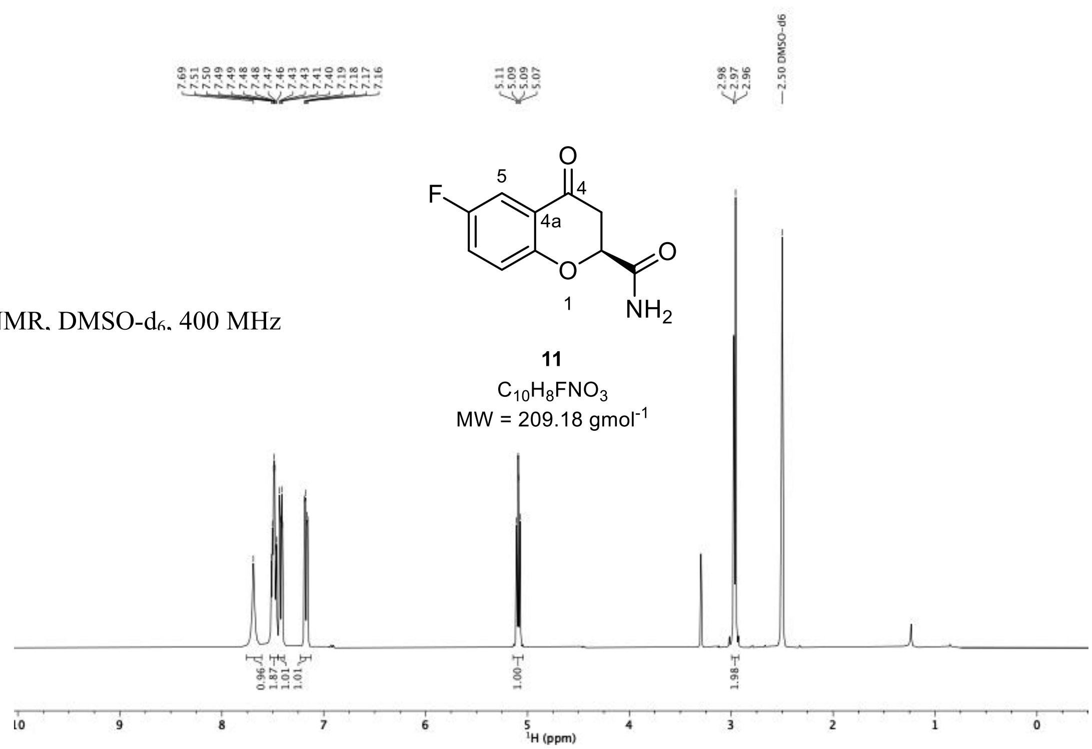

11

$C_{10}H_8FNO_3$

MW = 209.18 gmol^{-1}

^{13}C-NMR, DMSO-d$_6$, 101 MHz

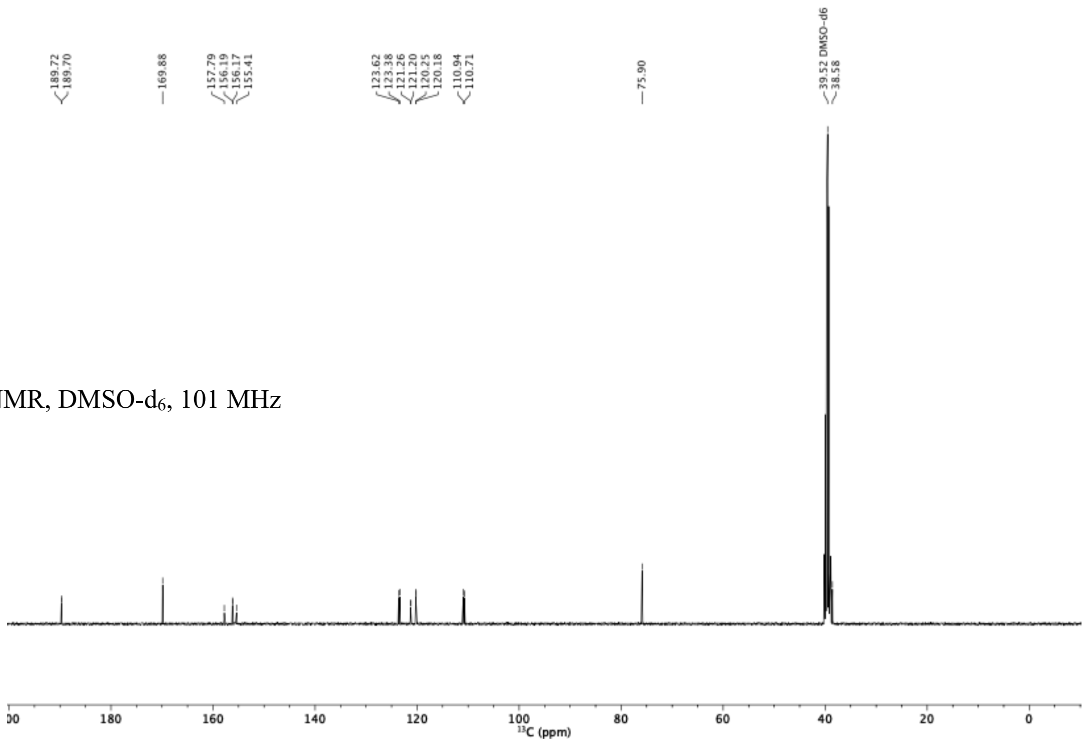

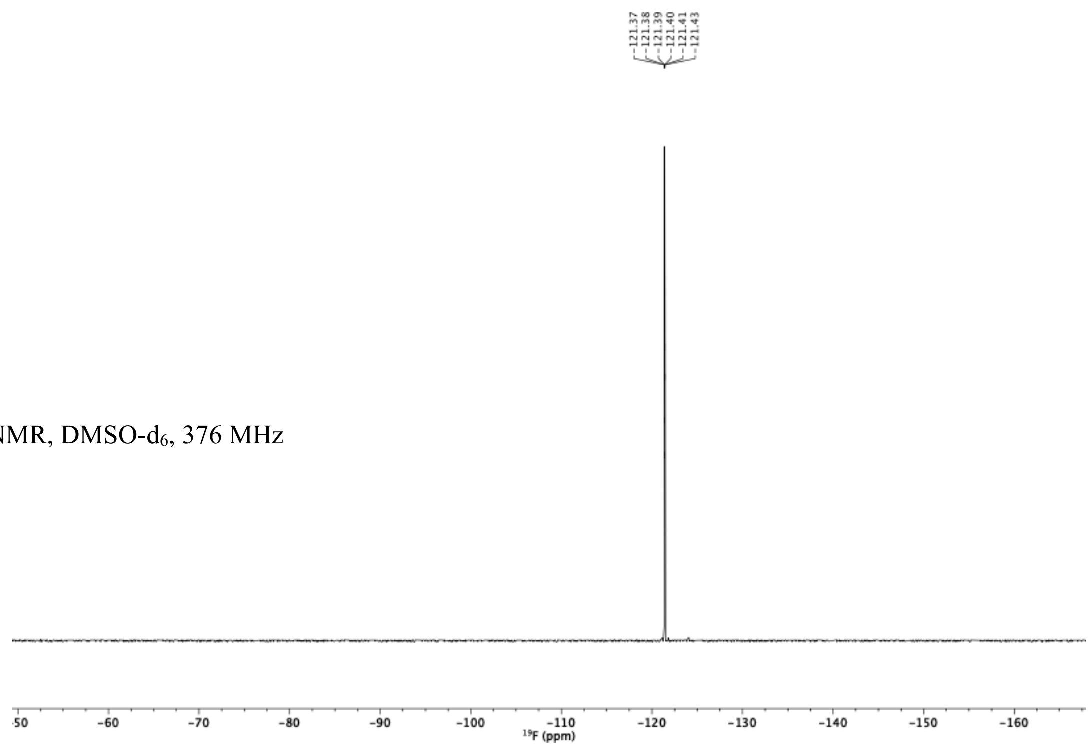

^{19}F-NMR, DMSO-d_6, 376 MHz

(2*S*,4*S*)-6-Fluoro-2',5'-dioxospiro[chromane-4,4'-imidazolidine]-2-carboxamide (Fidarestat)

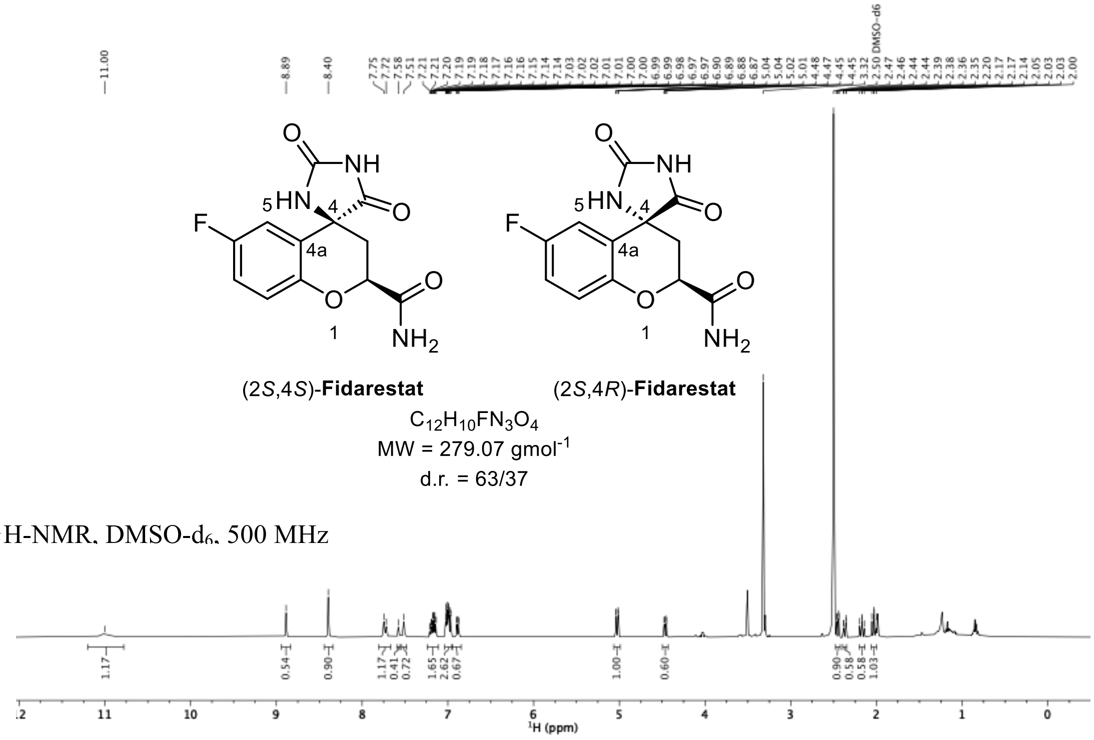

^{1}H-NMR, DMSO-d$_6$, 500 MHz

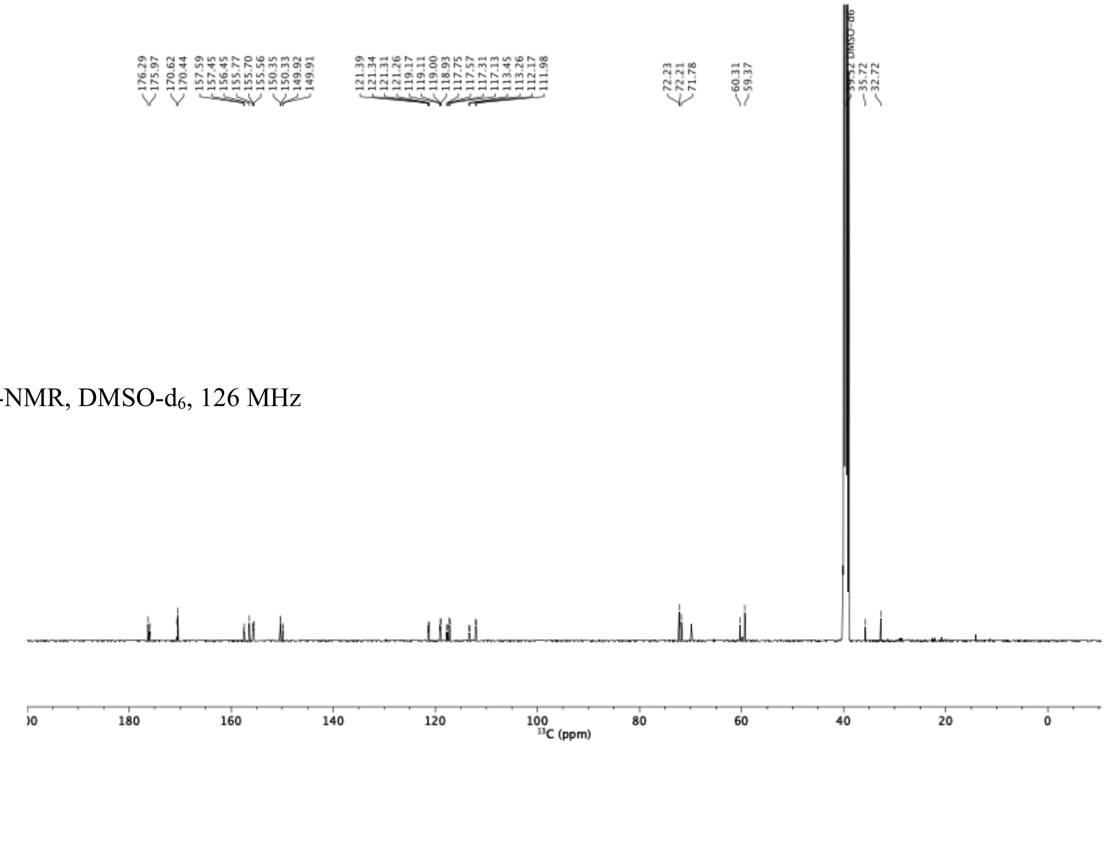

^{13}C-NMR, DMSO-d$_6$, 126 MHz

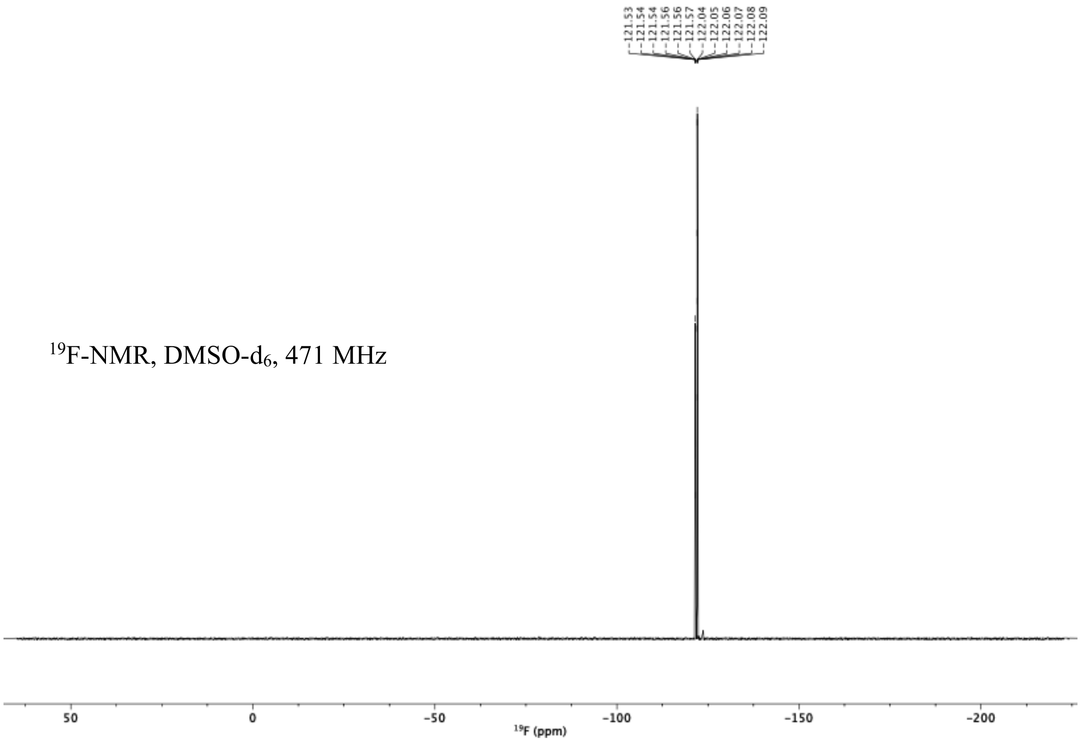

-121.53
-121.54
-121.54
-121.56
-121.56
-121.57
-122.04
-122.05
-122.06
-122.07
-122.08
-122.09
50
0
-50
-100
-150
-200
19F (ppm)

S14. HPLC Traces

(*R*)-Chromane-2-carboxamide (1a)

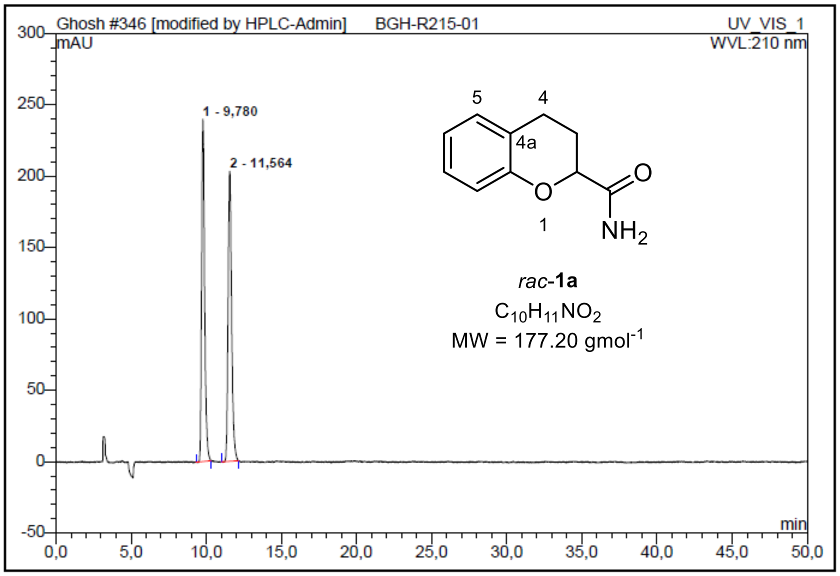

No.	Ret.Time min	Peak Name	Height mAU	Area mAU*min	Rel.Area %	Amount	Type
1	9,78	n.a.	239,871	55,231	50,06	n.a.	BMB*
2	11,56	n.a.	203,014	55,099	49,94	n.a.	BMB*
Total:			442,886	110,330	100,00	0,000	

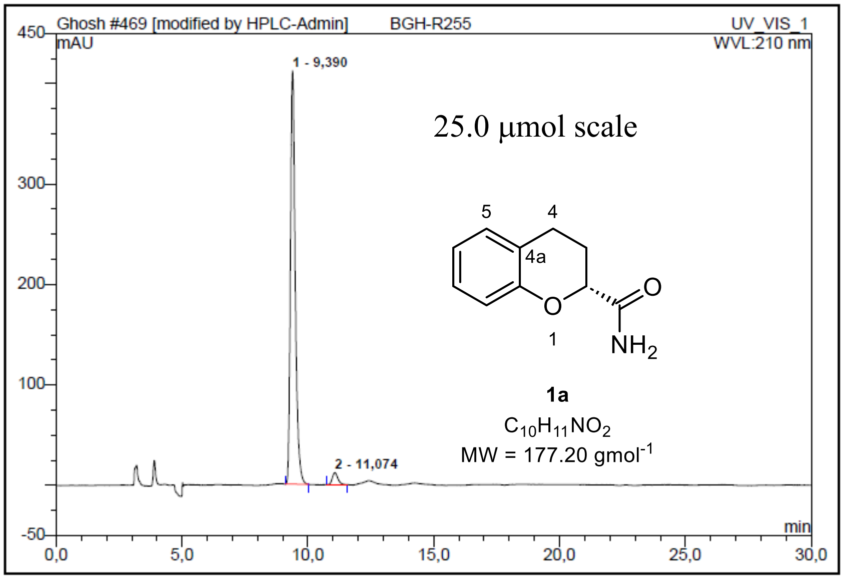

No.	Ret.Time min	Peak Name	Height mAU	Area mAU*min	Rel.Area %	Amount	Type
1	9,39	n.a.	411,787	91,188	96,68	n.a.	BMB
2	11,07	n.a.	12,083	3,128	3,32	n.a.	BMB*
Total:			423,870	94,317	100,00	0,000	

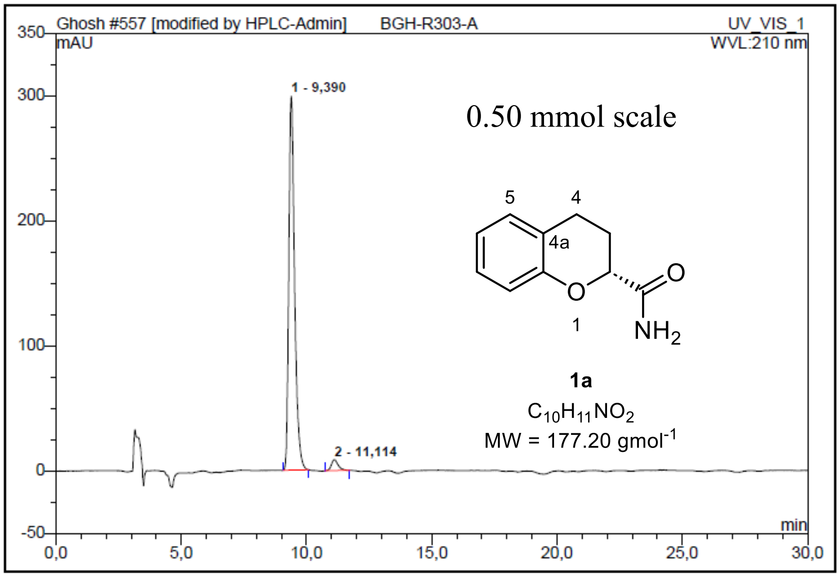

No.	Ret.Time min	Peak Name	Height mAU	Area mAU*min	Rel.Area %	Amount	Type
1	9,39	n.a.	298,816	80,216	96,83	n.a.	BMB
2	11,11	n.a.	8,666	2,624	3,17	n.a.	BMB*
Total:			307,482	82,841	100,00	0,000	

(*R*)-6-Fluorochromane-2-carboxamide (1b)

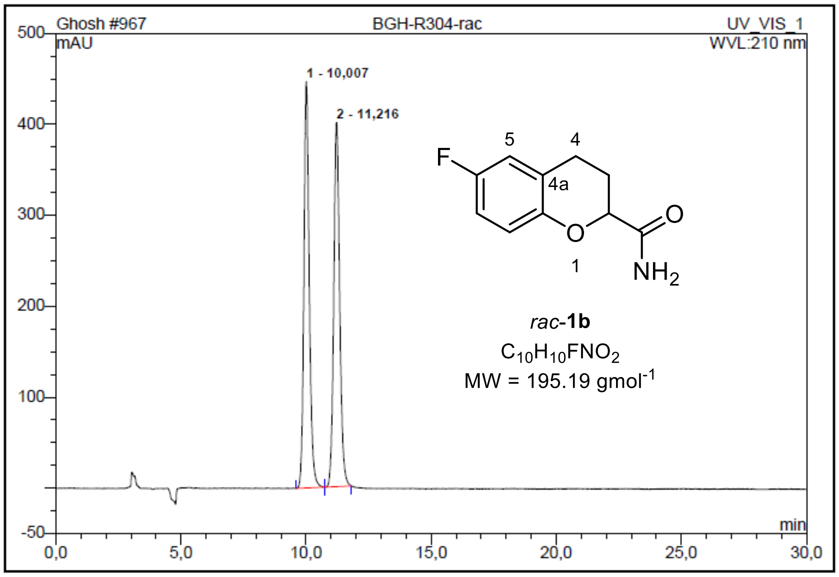

No.	Ret.Time min	Peak Name	Height mAU	Area mAU*min	Rel.Area %	Amount	Type
1	10,01	n.a.	446,763	110,740	50,29	n.a.	BM
2	11,22	n.a.	400,237	109,449	49,71	n.a.	MB
Total:			847,000	220,189	100,00	0,000	

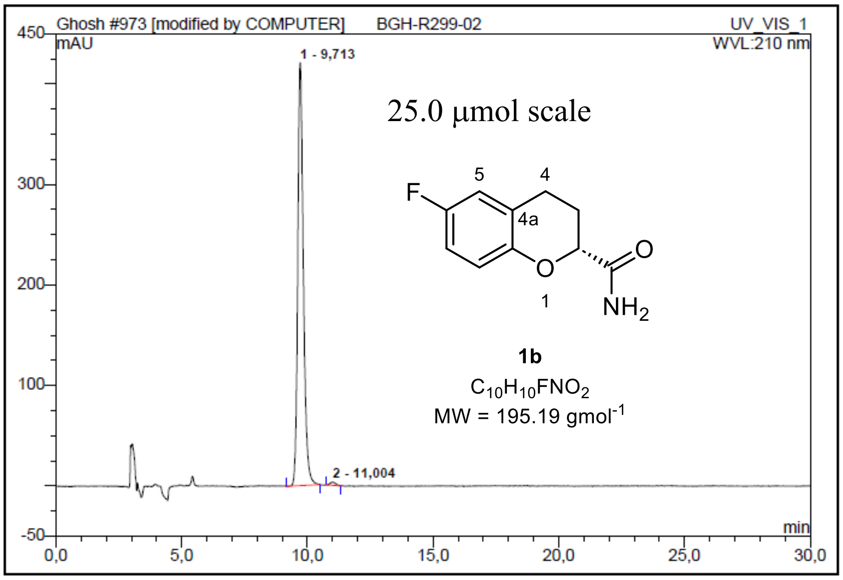

No.	Ret.Time min	Peak Name	Height mAU	Area mAU*min	Rel.Area %	Amount	Type
1	9,71	n.a.	420,938	108,579	99,27	n.a.	BMB*
2	11,00	n.a.	3,339	0,796	0,73	n.a.	BMB*
Total:			424,277	109,375	100,00	0,000	

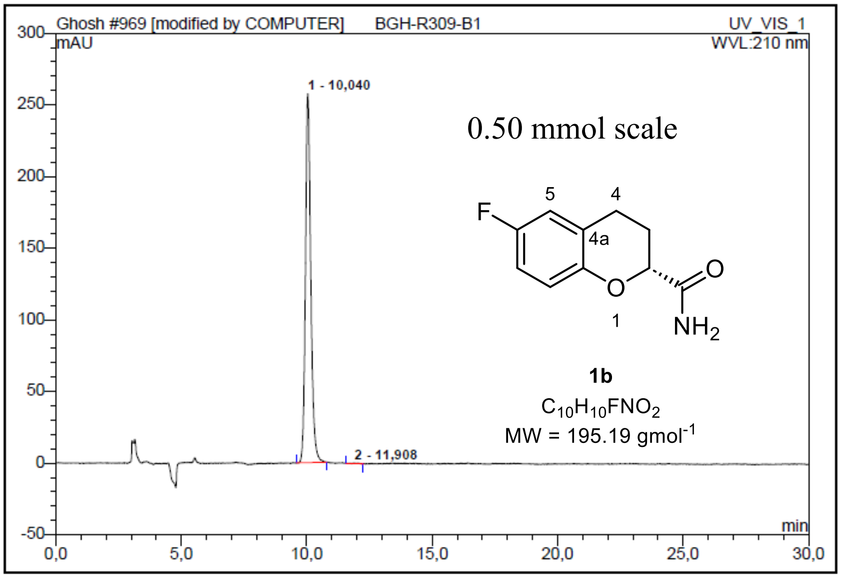

No.	Ret.Time min	Peak Name	Height mAU	Area mAU*min	Rel.Area %	Amount	Type
1	10,04	n.a.	257,591	63,859	99,86	n.a.	BMB*
2	11,91	n.a.	0,376	0,093	0,14	n.a.	BMB*
Total:			257,967	63,951	100,00	0,000	

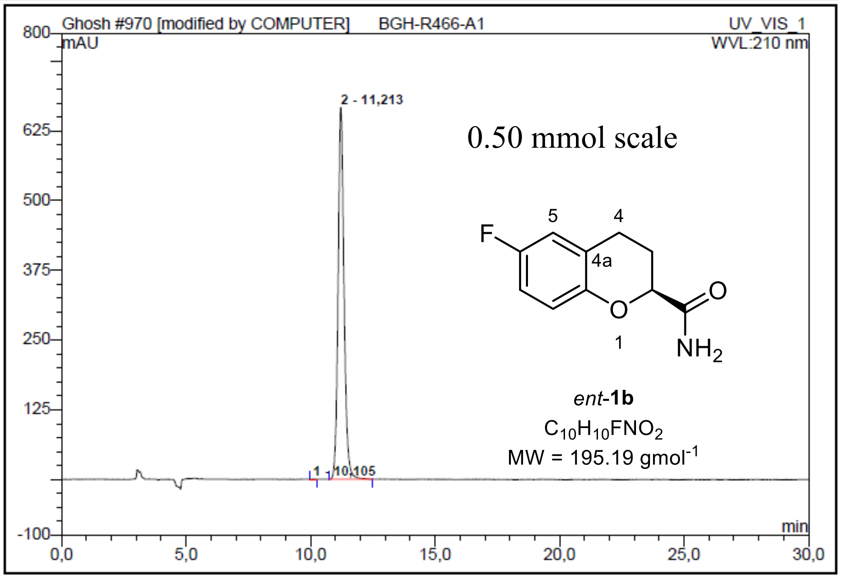

No.	Ret.Time min	Peak Name	Height mAU	Area mAU*min	Rel.Area %	Amount	Type
1	10,10	n.a.	0,599	0,069	0,04	n.a.	BMB*
2	11,21	n.a.	666,562	185,318	99,96	n.a.	BMB*
Total:			667,161	185,387	100,00	0,000	

(*R*)-6-Chlorochromane-2-carboxamide (1c)

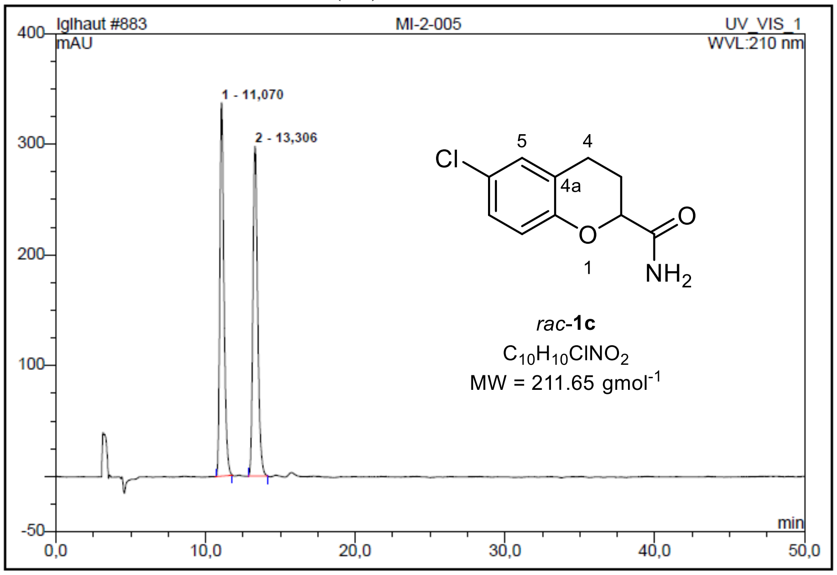

No.	Ret.Time min	Peak Name	Height mAU	Area mAU*min	Rel.Area %	Amount	Type
1	11,07	n.a.	337,500	108,999	49,83	n.a.	BMB
2	13,31	n.a.	297,839	109,756	50,17	n.a.	BMB
Total:			635,340	218,755	100,00	0,000	

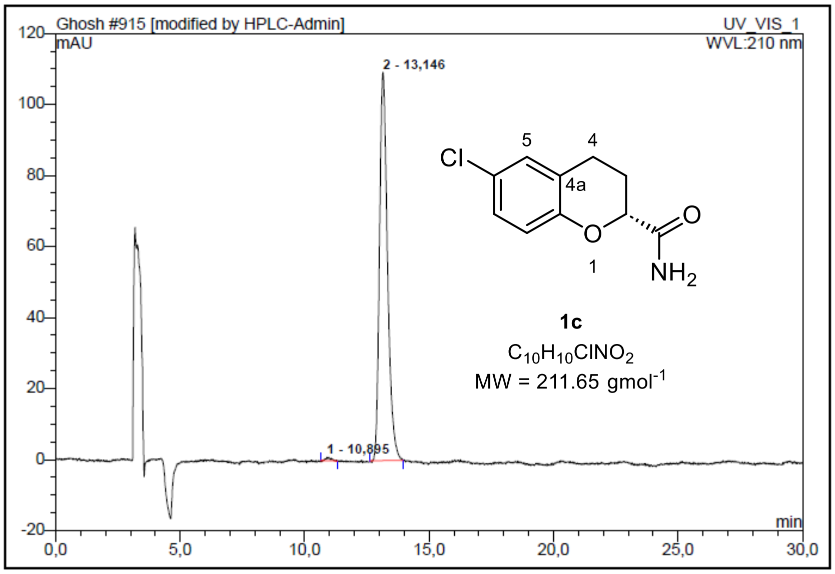

No.	Ret.Time min	Peak Name	Height mAU	Area mAU*min	Rel.Area %	Amount	Type
1	10,90	n.a.	0,960	0,213	0,52	n.a.	BMB*
2	13,15	n.a.	109,302	40,745	99,48	n.a.	BMB*
Total:			110,262	40,958	100,00	0,000	

(*R*)-6-Bromochromane-2-carboxamide (1d)

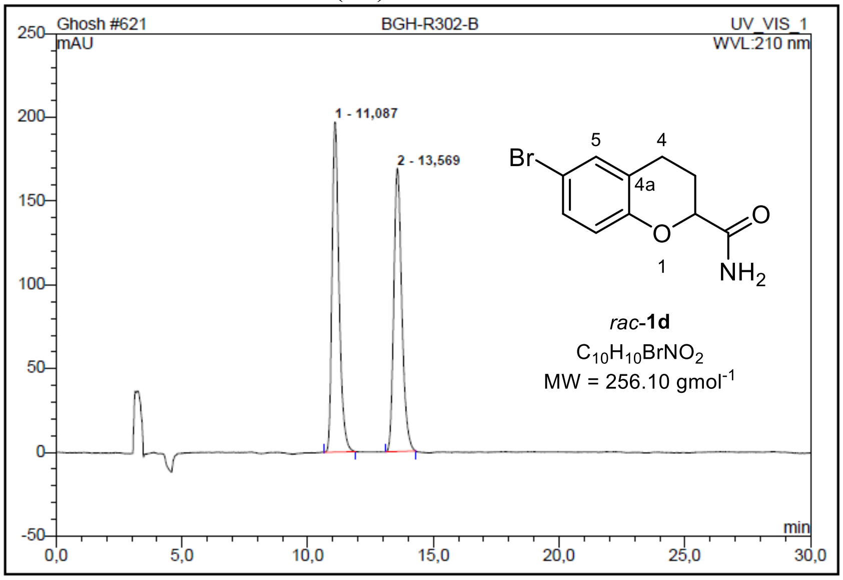

No.	Ret.Time min	Peak Name	Height mAU	Area mAU*min	Rel.Area %	Amount	Type
1	11,09	n.a.	196,954	63,193	50,23	n.a.	BMB
2	13,57	n.a.	169,080	62,622	49,77	n.a.	BMB
Total:			366,034	125,815	100,00	0,000	

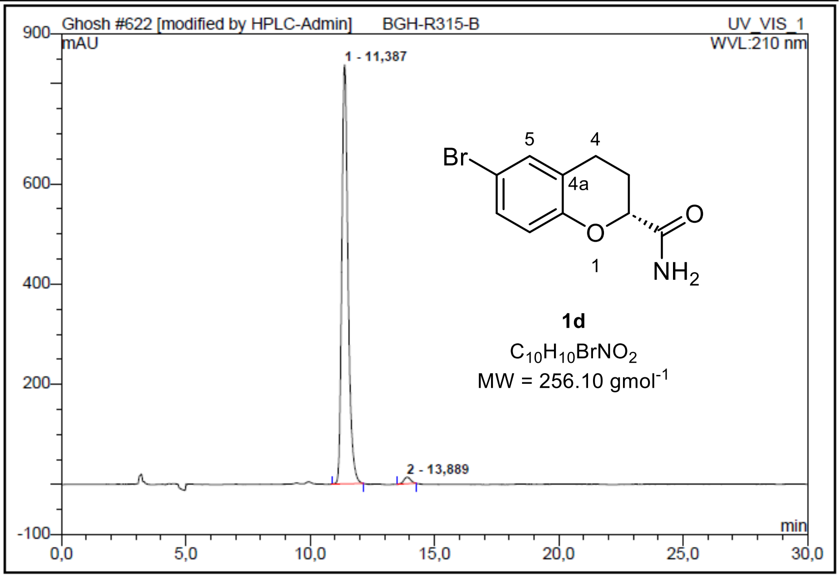

No.	Ret.Time min	Peak Name	Height mAU	Area mAU*min	Rel.Area %	Amount	Type
1	11,39	n.a.	836,297	244,008	98,32	n.a.	BMB
2	13,89	n.a.	13,309	4,166	1,68	n.a.	BMB*
Total:			849,606	248,174	100,00	0,000	

(*R*)-6-Iodochromane-2-carboxamide (1e)

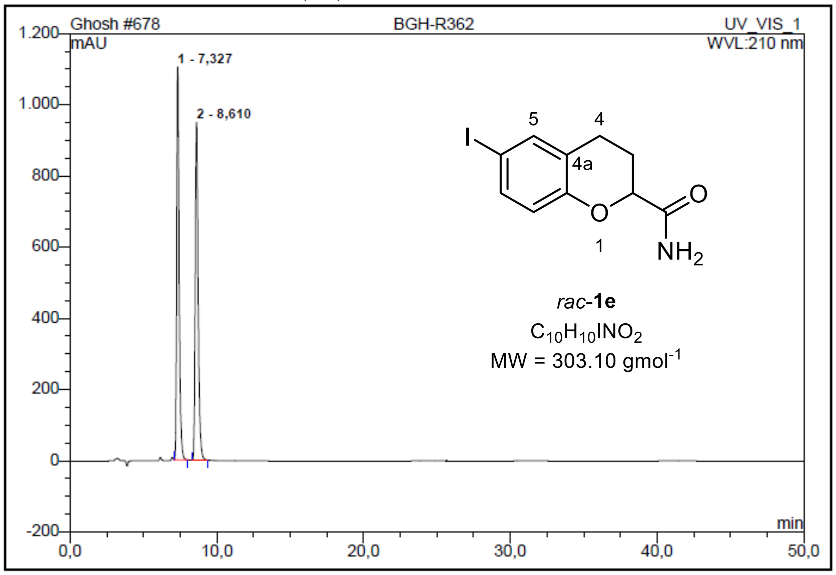

No.	Ret.Time min	Peak Name	Height mAU	Area mAU*min	Rel.Area %	Amount	Type
1	7,33	n.a.	1103,710	215,784	49,59	n.a.	BMB
2	8,61	n.a.	948,848	219,342	50,41	n.a.	BMB
Total:			2052,558	435,126	100,00	0,000	

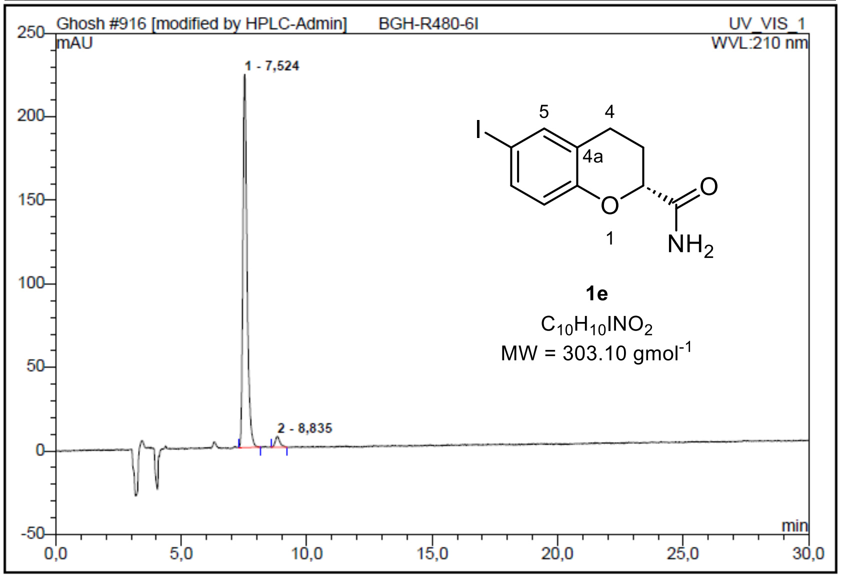

No.	Ret.Time min	Peak Name	Height mAU	Area mAU*min	Rel.Area %	Amount	Type
1	7,52	n.a.	223,326	42,825	96,84	n.a.	BMB*
2	8,83	n.a.	6,541	1,399	3,16	n.a.	BMB*
Total:			229,866	44,224	100,00	0,000	

(*R*)-6-Methylchromane-2-carboxamide (1f)

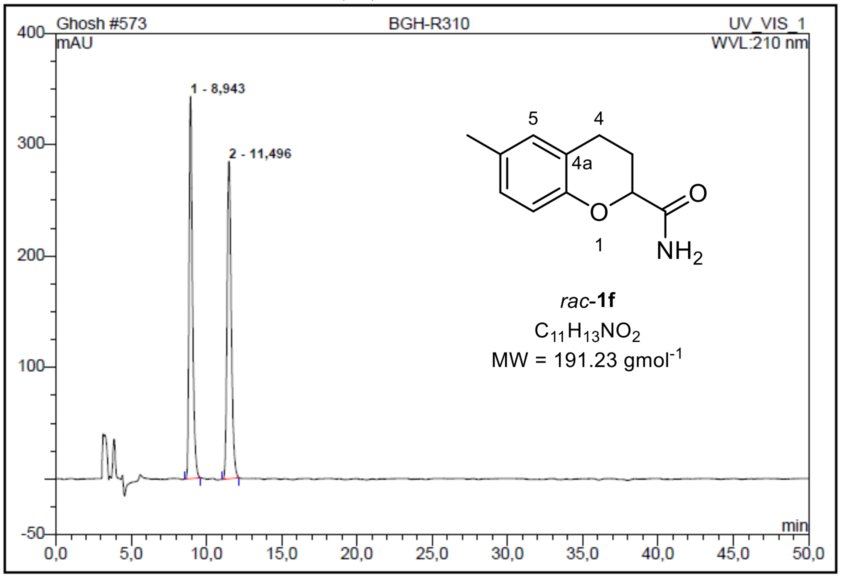

No.	Ret.Time min	Peak Name	Height mAU	Area mAU*min	Rel.Area %	Amount	Type
1	8,94	n.a.	343,174	91,360	49,93	n.a.	BMB
2	11,50	n.a.	284,960	91,612	50,07	n.a.	BMB
Total:			628,135	182,972	100,00	0,000	

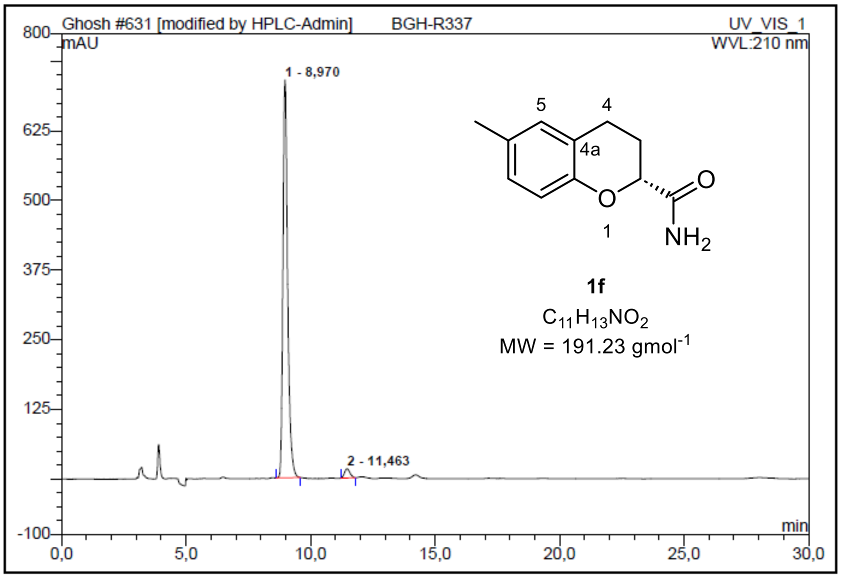

No.	Ret.Time min	Peak Name	Height mAU	Area mAU*min	Rel.Area %	Amount	Type
1	8,97	n.a.	714,342	156,767	97,43	n.a.	BMB
2	11,46	n.a.	16,435	4,129	2,57	n.a.	BMB*
Total:			730,777	160,896	100,00	0,000	

(*R*)-6-Methoxychromane-2-carboxamide (1g)

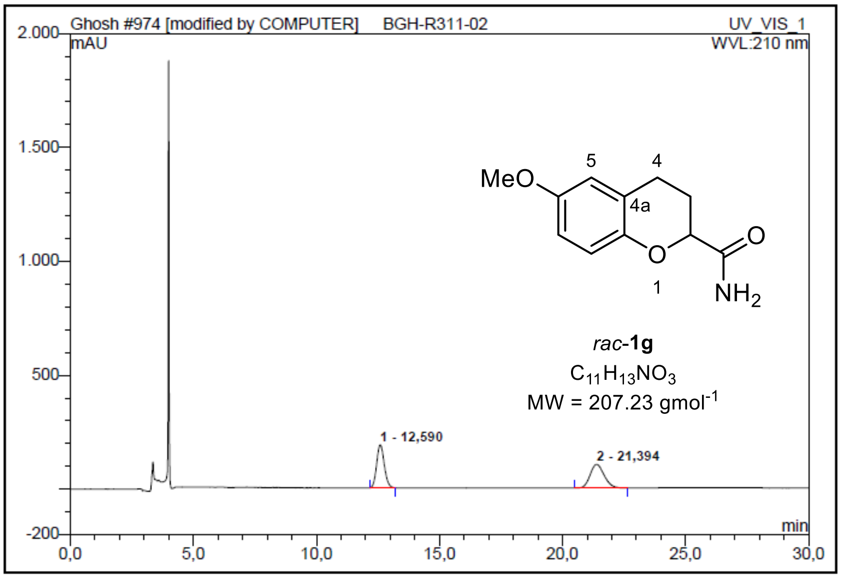

No.	Ret.Time min	Peak Name	Height mAU	Area mAU*min	Rel.Area %	Amount	Type
1	12,59	n.a.	188,481	67,376	49,60	n.a.	BMB*
2	21,39	n.a.	103,908	68,454	50,40	n.a.	BMB*
Total:			292,388	135,830	100,00	0,000	

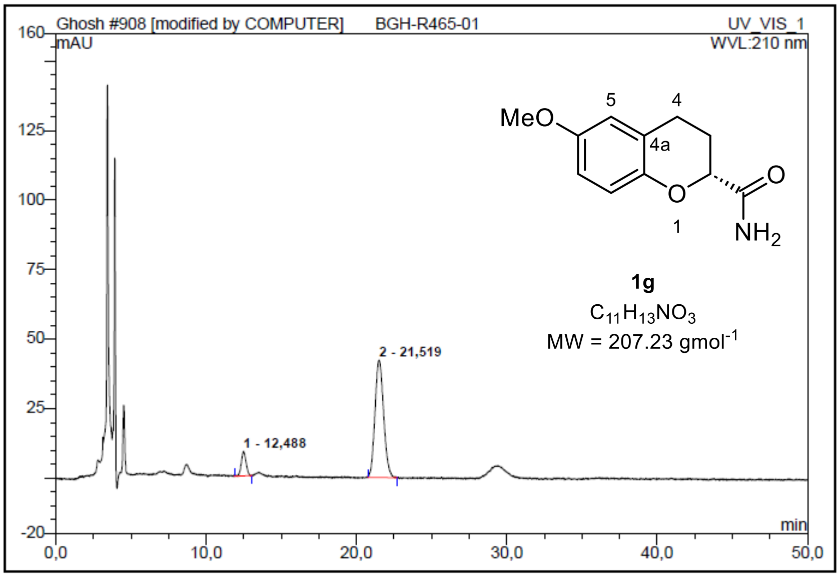

No.	Ret.Time min	Peak Name	Height mAU	Area mAU*min	Rel.Area %	Amount	Type
1	12,49	n.a.	8,880	3,215	10,24	n.a.	BMB*
2	21,52	n.a.	42,282	28,177	89,76	n.a.	BMB*
Total:			51,162	31,392	100,00	0,000	

(*R*)-6-Phenylchromane-2-carboxamide (1h)

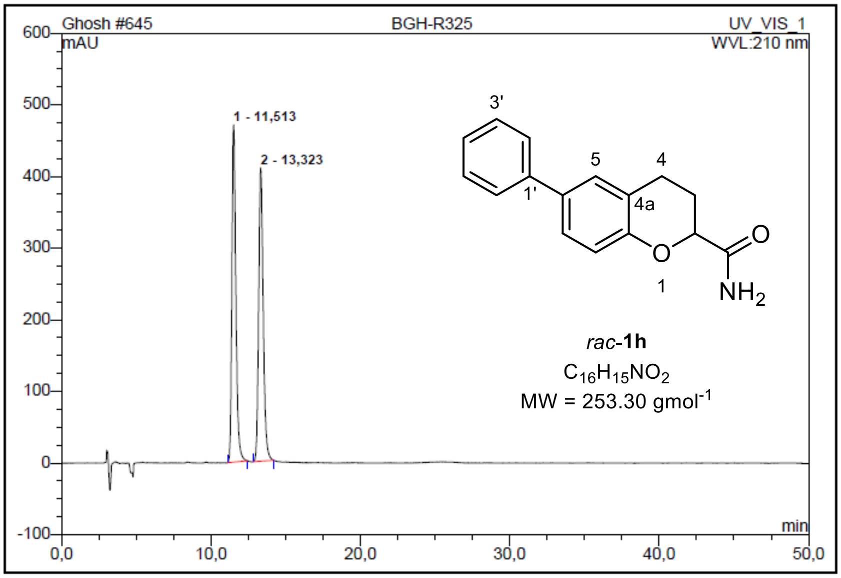

No.	Ret.Time min	Peak Name	Height mAU	Area mAU*min	Rel.Area %	Amount	Type
1	11,51	n.a.	470,809	145,200	50,20	n.a.	BMB
2	13,32	n.a.	409,522	144,063	49,80	n.a.	BMB
Total:			880,330	289,264	100,00	0,000	

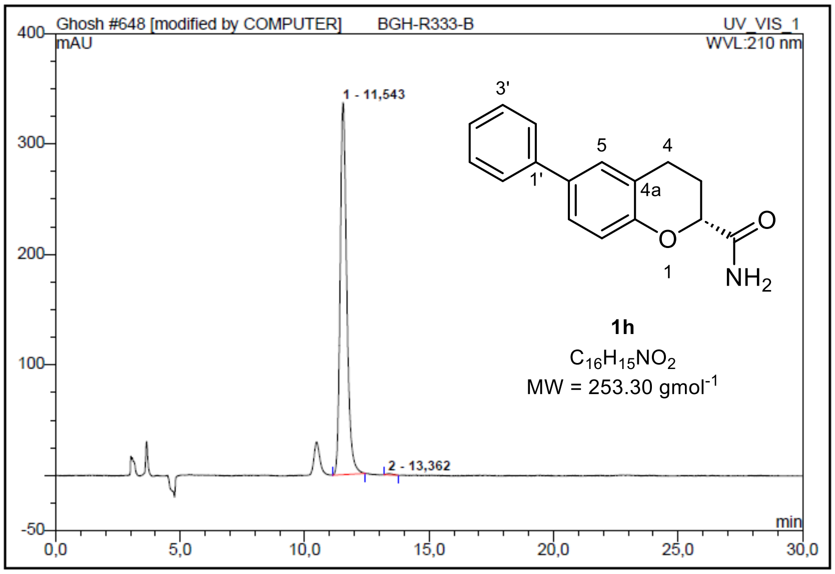

No.	Ret.Time min	Peak Name	Height mAU	Area mAU*min	Rel.Area %	Amount	Type
1	11,54	n.a.	336,741	103,421	99,71	n.a.	BMB
2	13,36	n.a.	1,142	0,304	0,29	n.a.	BMB*
Total:			337,882	103,725	100,00	0,000	

(*R*)-6-Ethylchromane-2-carboxamide (1i)

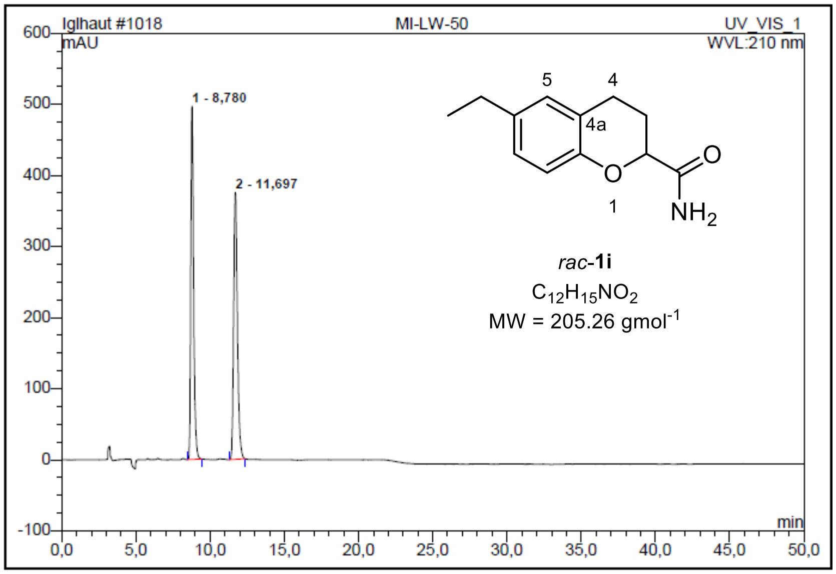

No.	Ret.Time min	Peak Name	Height mAU	Area mAU*min	Rel.Area %	Amount	Type
1	8,78	n.a.	496,738	108,612	49,79	n.a.	BMB
2	11,70	n.a.	375,439	109,542	50,21	n.a.	BMB
Total:			872,177	218,154	100,00	0,000	

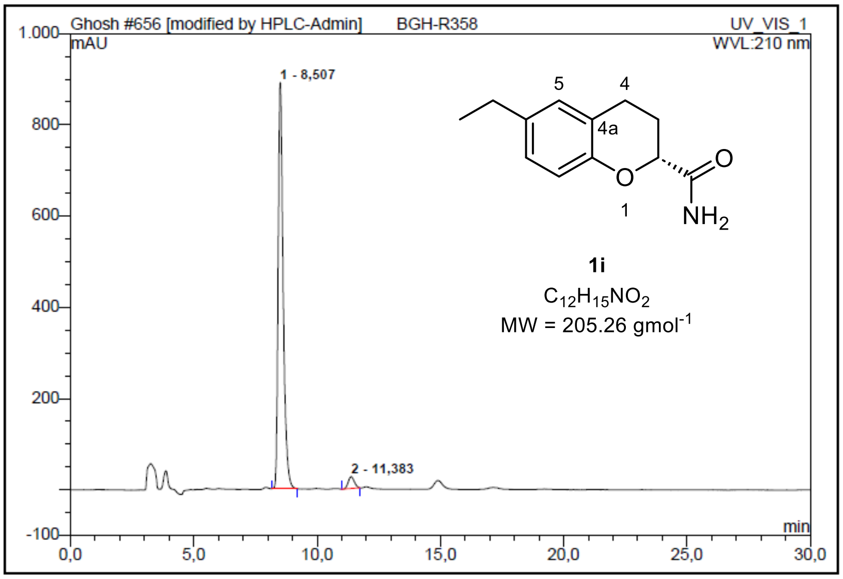

No.	Ret.Time min	Peak Name	Height mAU	Area mAU*min	Rel.Area %	Amount	Type
1	8,51	n.a.	890,171	208,994	96,58	n.a.	BMB
2	11,38	n.a.	26,001	7,395	3,42	n.a.	BMB*
Total:			916,172	216,390	100,00	0,000	

(*R*)-5-Methylchromane-2-carboxamide (1j)

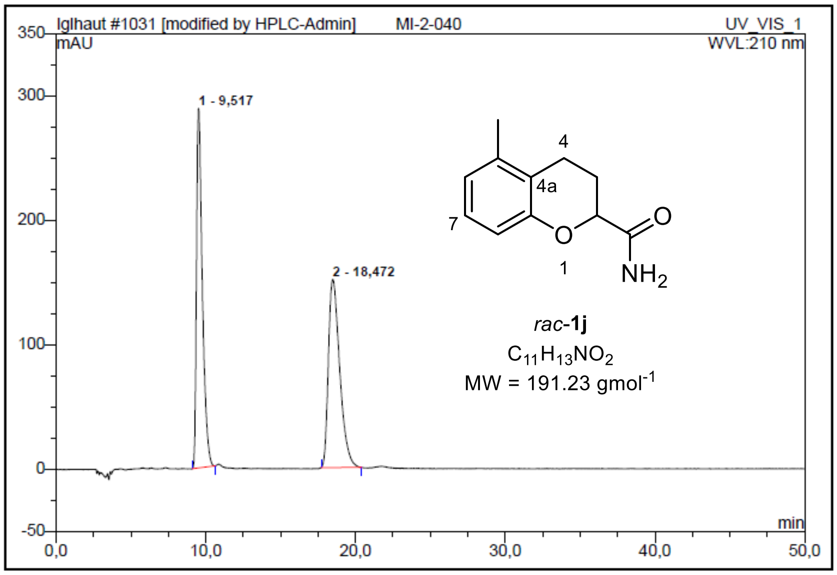

No.	Ret.Time min	Peak Name	Height mAU	Area mAU*min	Rel.Area %	Amount	Type
1	9,52	n.a.	288,683	131,203	49,81	n.a.	BMB*
2	18,47	n.a.	151,035	132,212	50,19	n.a.	BMB*
Total:			439,718	263,415	100,00	0,000	

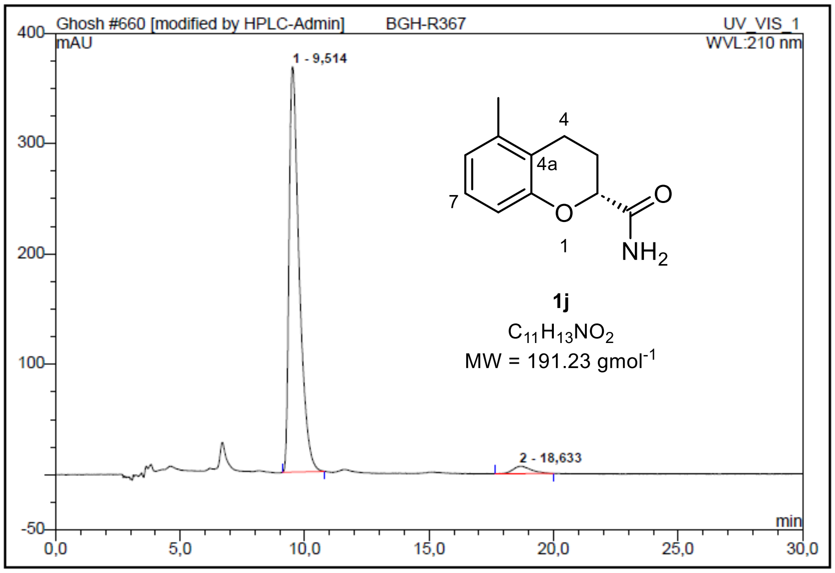

No.	Ret.Time min	Peak Name	Height mAU	Area mAU*min	Rel.Area %	Amount	Type
1	9,51	n.a.	367,474	173,400	96,83	n.a.	BMB*
2	18,63	n.a.	6,777	5,677	3,17	n.a.	BMB*
Total:			374,251	179,077	100,00	0,000	

(*R*)-7-Methylchromane-2-carboxamide (1k)

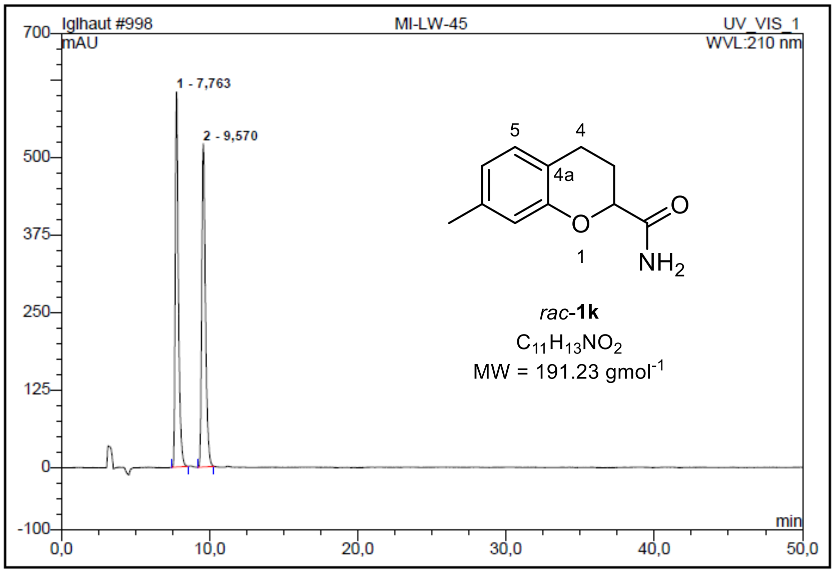

No.	Ret.Time min	Peak Name	Height mAU	Area mAU*min	Rel.Area %	Amount	Type
1	7,76	n.a.	604,188	149,294	49,84	n.a.	BMB
2	9,57	n.a.	520,964	150,252	50,16	n.a.	BMB
Total:			1125,152	299,546	100,00	0,000	

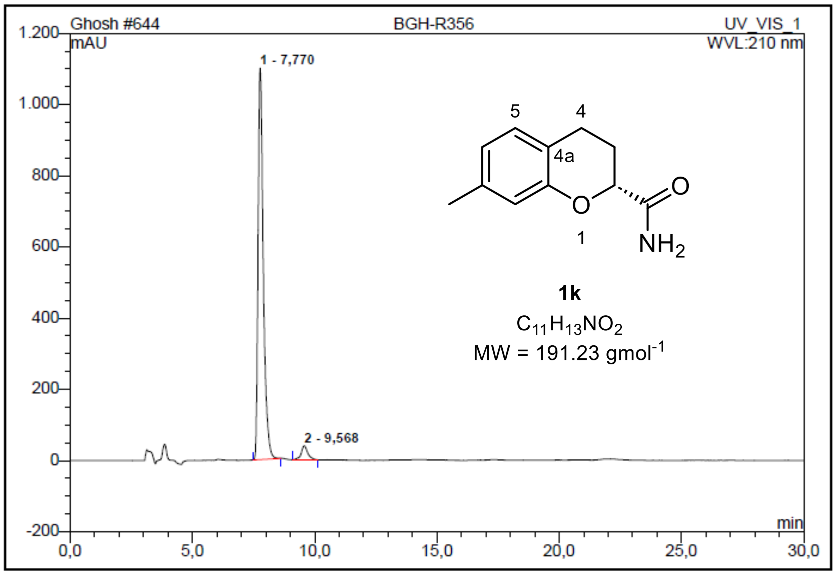

No.	Ret.Time min	Peak Name	Height mAU	Area mAU*min	Rel.Area %	Amount	Type
1	7,77	n.a.	1099,123	272,214	95,62	n.a.	BMB
2	9,57	n.a.	39,651	12,470	4,38	n.a.	BMB
Total:			1138,774	284,684	100,00	0,000	

(*R*)-8-Methylchromane-2-carboxamide (1l)

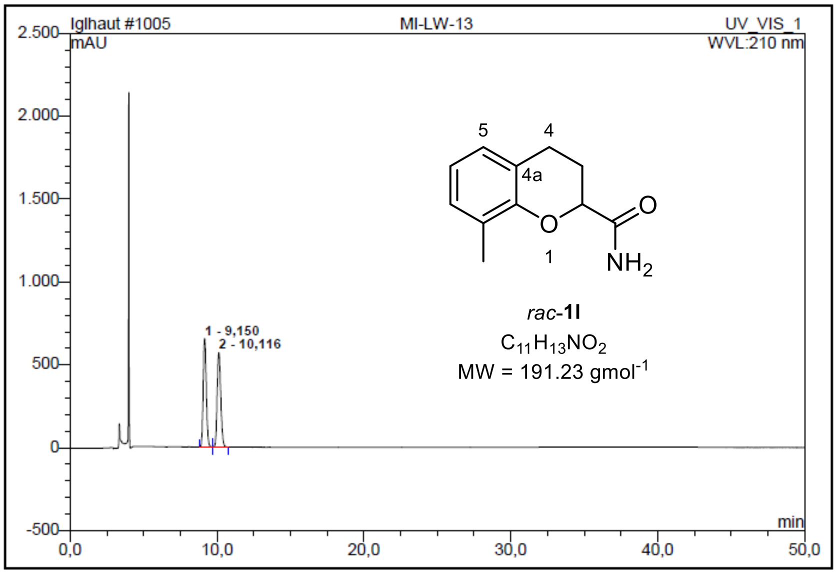

No.	Ret.Time min	Peak Name	Height mAU	Area mAU*min	Rel.Area %	Amount	Type
1	9,15	n.a.	653,700	160,903	49,87	n.a.	BM
2	10,12	n.a.	569,942	161,750	50,13	n.a.	MB
Total:			1223,641	322,654	100,00	0,000	

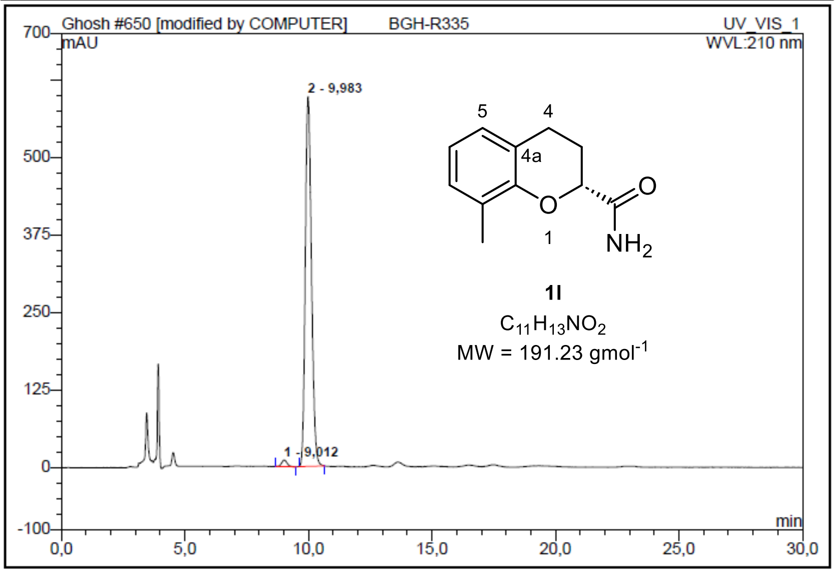

No.	Ret.Time min	Peak Name	Height mAU	Area mAU*min	Rel.Area %	Amount	Type
1	9,01	n.a.	10,480	2,724	1,55	n.a.	BMB*
2	9,98	n.a.	595,570	172,664	98,45	n.a.	BMB
Total:			606,051	175,388	100,00	0,000	

(*R*)-6,7,8,9-Tetrahydro-benzo[*g*]chromane-2-carboxamide (1m)

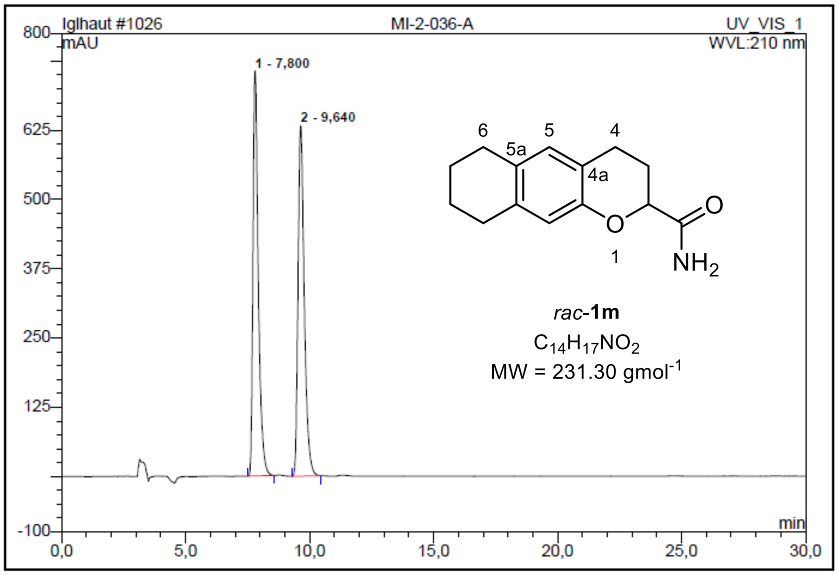

No.	Ret.Time min	Peak Name	Height mAU	Area mAU*min	Rel.Area %	Amount	Type
1	7,80	n.a.	731,250	182,241	49,76	n.a.	BMB
2	9,64	n.a.	632,231	183,971	50,24	n.a.	BMB
Total:			1363,481	366,212	100,00	0,000	

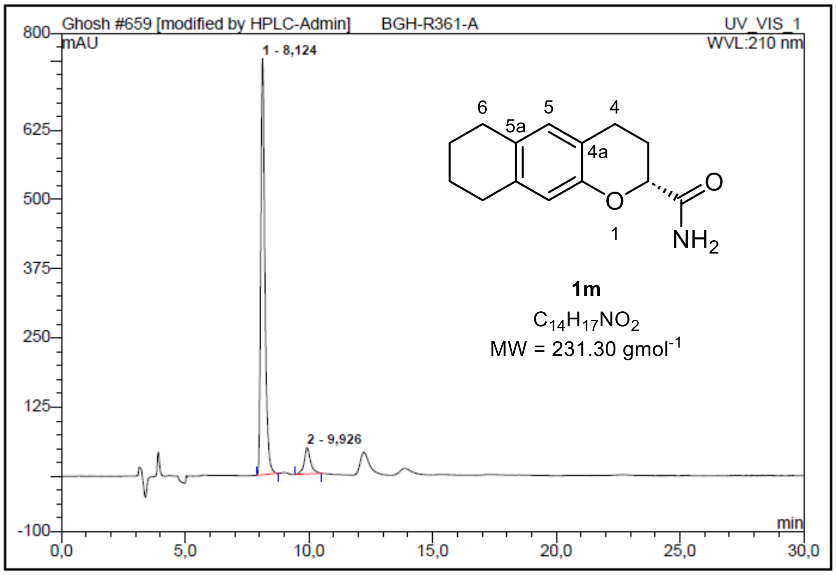

No.	Ret.Time min	Peak Name	Height mAU	Area mAU*min	Rel.Area %	Amount	Type
1	8,12	n.a.	751,563	145,907	91,15	n.a.	BMB
2	9,93	n.a.	47,208	14,172	8,85	n.a.	BMB
Total:			798,772	160,080	100,00	0,000	

(*R*)-6-(Pyridine-3-yl)chromane-2-carboxamide (1n)

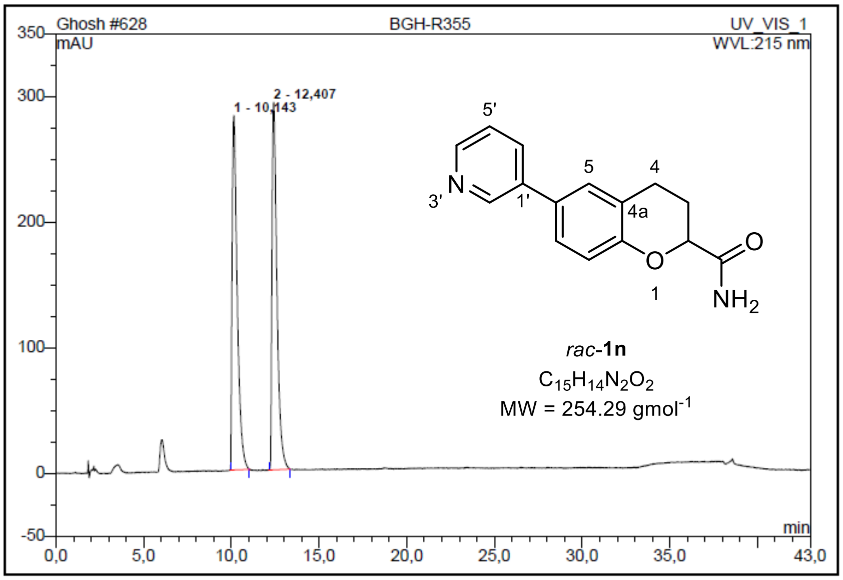

No.	Ret.Time min	Peak Name	Height mAU	Area mAU*min	Rel.Area %	Amount	Type
1	10,14	n.a.	281,996	95,436	49,88	n.a.	BMB
2	12,41	n.a.	292,472	95,885	50,12	n.a.	BMB
Total:			574,468	191,321	100,00	0,000	

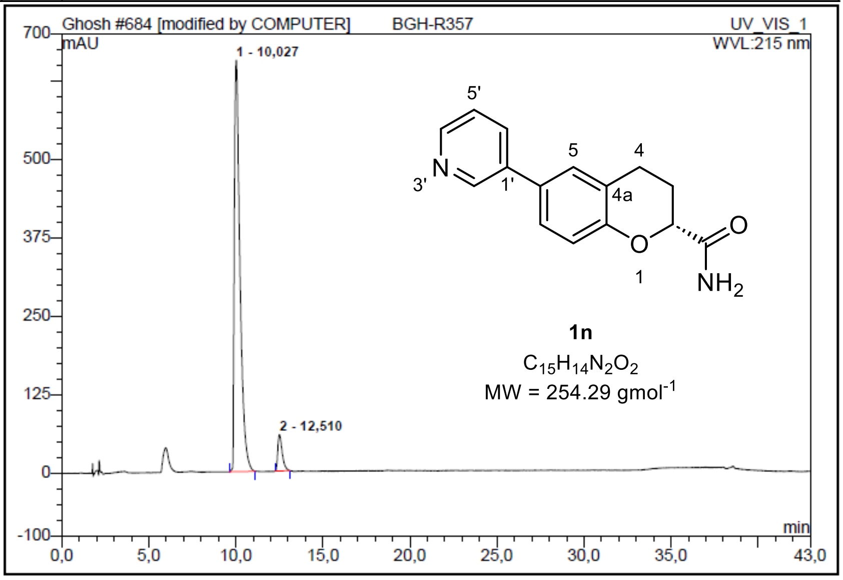

No.	Ret.Time min	Peak Name	Height mAU	Area mAU*min	Rel.Area %	Amount	Type
1	10,03	n.a.	655,063	244,056	93,44	n.a.	BMB
2	12,51	n.a.	57,971	17,138	6,56	n.a.	BMB
Total:			713,034	261,195	100,00	0,000	

(*R*)-7-Methoxychromane-2-carboxamide (1o)

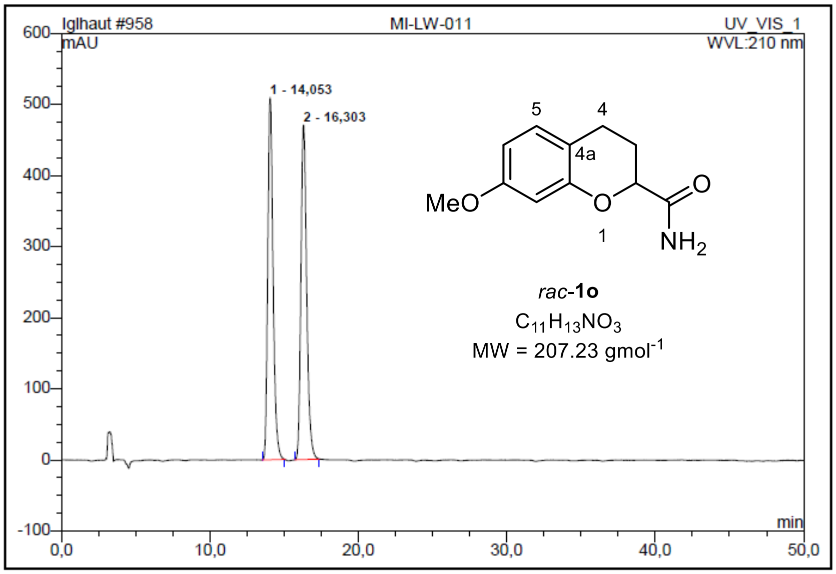

No.	Ret.Time min	Peak Name	Height mAU	Area mAU*min	Rel.Area %	Amount	Type
1	14,05	n.a.	509,112	211,004	49,87	n.a.	BMB
2	16,30	n.a.	470,346	212,097	50,13	n.a.	BMB
Total:			979,458	423,101	100,00	0,000	

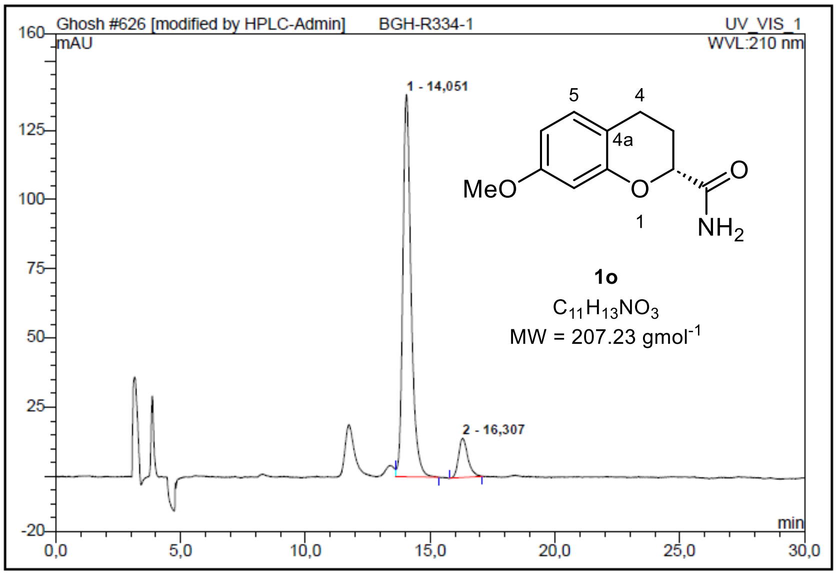

No.	Ret.Time min	Peak Name	Height mAU	Area mAU*min	Rel.Area %	Amount	Type
1	14,05	n.a.	138,079	52,920	89,65	n.a.	MB*
2	16,31	n.a.	14,140	6,110	10,35	n.a.	BMB*
Total:			152,219	59,031	100,00	0,000	

(*R*)-7-Fluorochromane-2-carboxamide (1p)

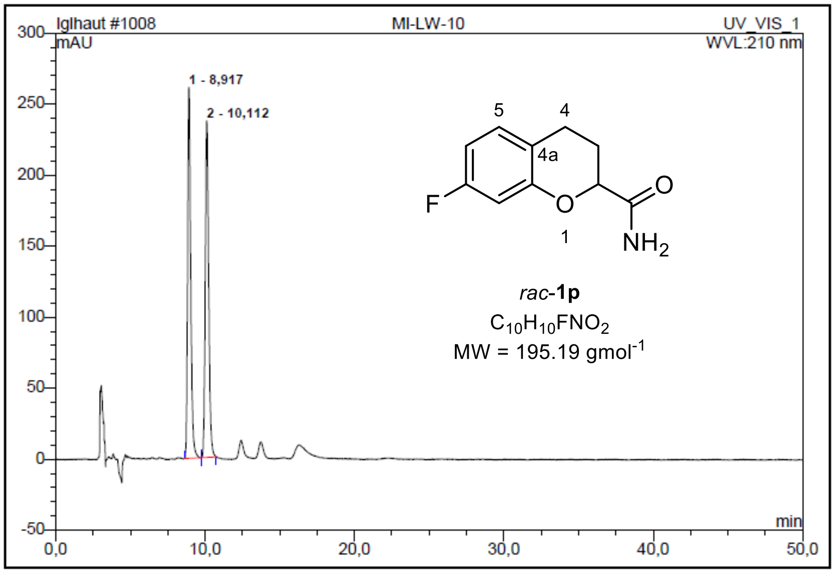

No.	Ret.Time min	Peak Name	Height mAU	Area mAU*min	Rel.Area %	Amount	Type
1	8,92	n.a.	261,356	63,350	50,20	n.a.	BM
2	10,11	n.a.	237,032	62,852	49,80	n.a.	MB
Total:			498,388	126,203	100,00	0,000	

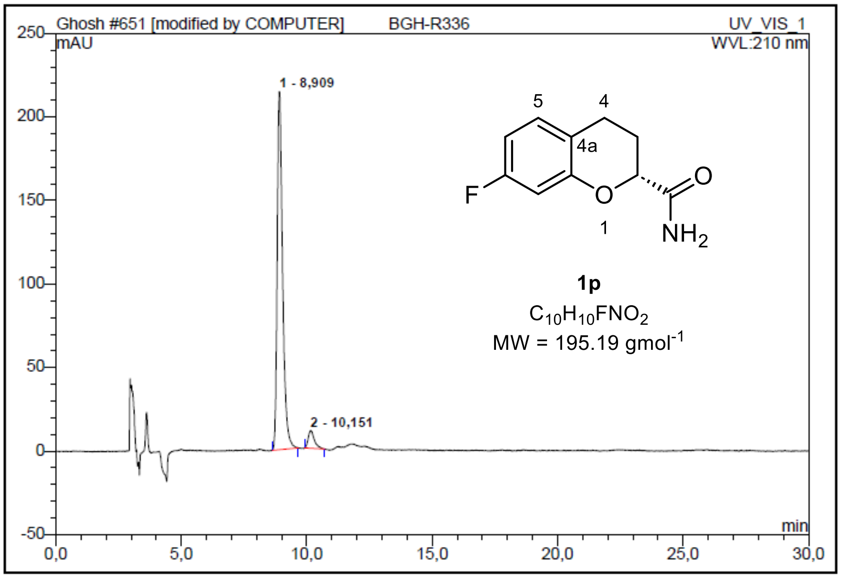

No.	Ret.Time min	Peak Name	Height mAU	Area mAU*min	Rel.Area %	Amount	Type
1	8,91	n.a.	214,349	53,193	94,68	n.a.	BMB*
2	10,15	n.a.	10,585	2,991	5,32	n.a.	BMB*
Total:			224,934	56,184	100,00	0,000	

(*R*)-8-Fluorochromane-2-carboxamide (1q)

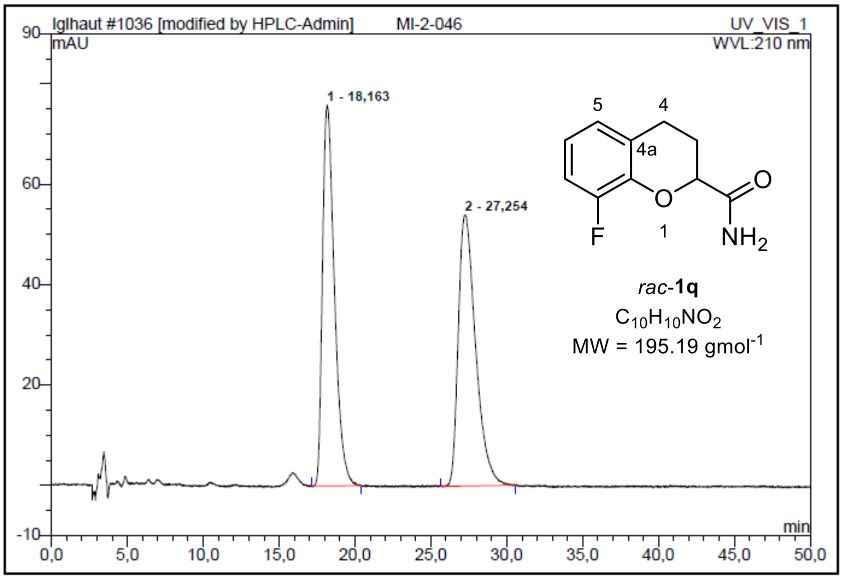

No.	Ret.Time min	Peak Name	Height mAU	Area mAU*min	Rel.Area %	Amount	Type
1	18,16	n.a.	75,813	69,083	49,22	n.a.	BMB*
2	27,25	n.a.	54,043	71,272	50,78	n.a.	BMB*
Total:			129,856	140,355	100,00	0,000	

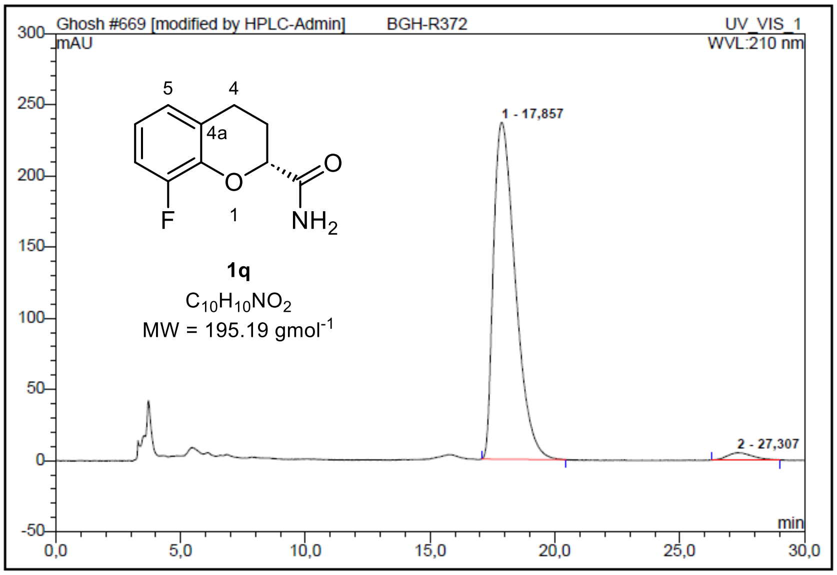

No.	Ret.Time min	Peak Name	Height mAU	Area mAU*min	Rel.Area %	Amount	Type
1	17,86	n.a.	236,617	239,994	97,58	n.a.	BMB*
2	27,31	n.a.	5,222	5,943	2,42	n.a.	BMB*
Total:			241,838	245,937	100,00	0,000	

(*R*)-1,4-Benzodioxane-2-carboxamide (1r)

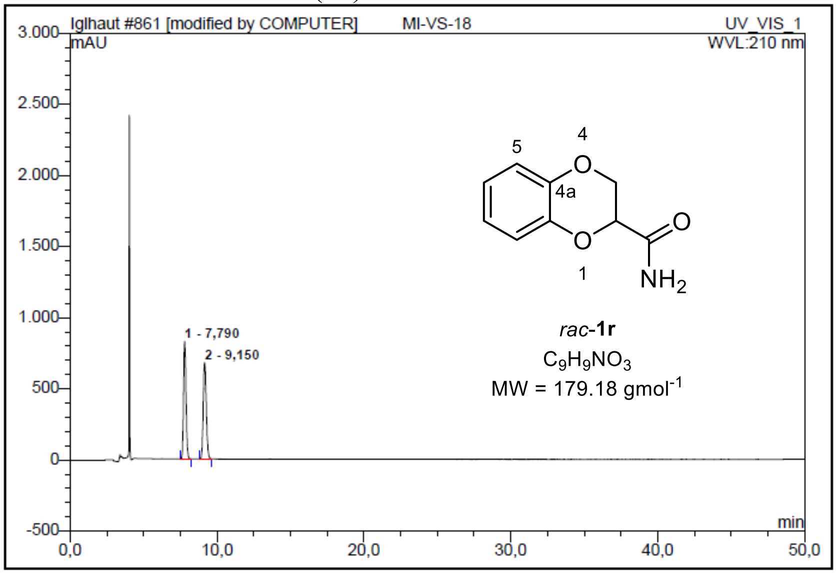

No.	Ret.Time min	Peak Name	Height mAU	Area mAU*min	Rel.Area %	Amount	Type
1	7,79	n.a.	825,638	162,224	49,79	n.a.	BMB
2	9,15	n.a.	677,786	163,607	50,21	n.a.	BMB
Total:			1503,424	325,831	100,00	0,000	

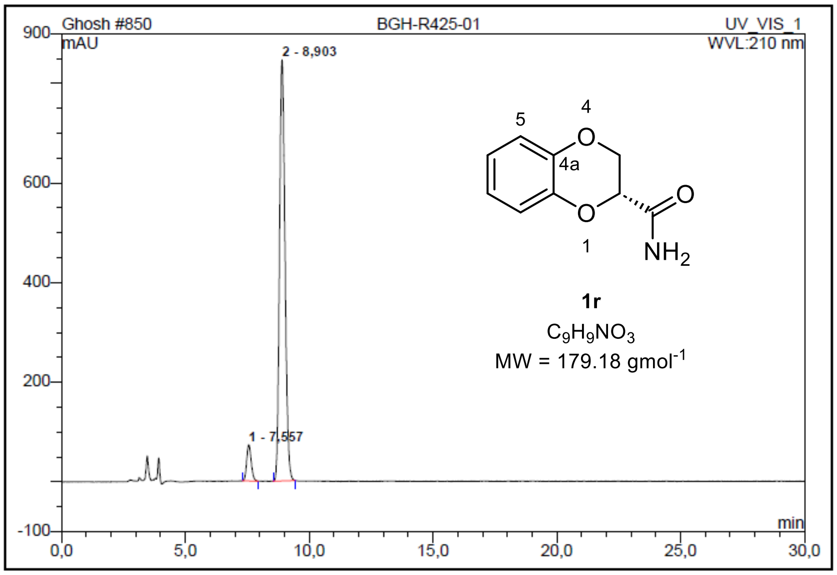

No.	Ret.Time min	Peak Name	Height mAU	Area mAU*min	Rel.Area %	Amount	Type
1	7,56	n.a.	72,535	15,327	6,56	n.a.	BMB
2	8,90	n.a.	845,380	218,423	93,44	n.a.	BMB
Total:			917,914	233,750	100,00	0,000	

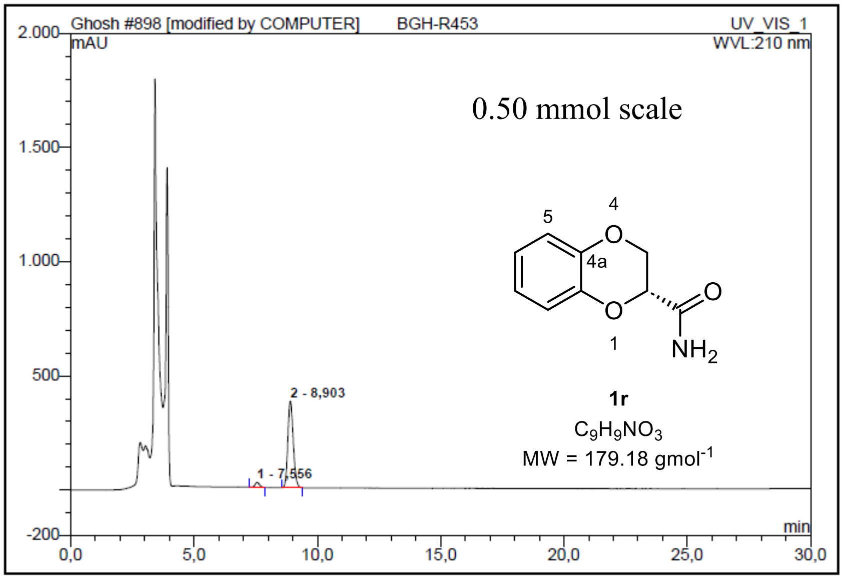

No.	Ret.Time min	Peak Name	Height mAU	Area mAU*min	Rel.Area %	Amount	Type
1	7,56	n.a.	21,657	4,464	4,43	n.a.	BMB*
2	8,90	n.a.	379,269	96,261	95,57	n.a.	BMB
Total:			400,927	100,724	100,00	0,000	

(*R*)-3,3-Dimethyl-1,4-benzodioxane-2-carboxamide (1s)

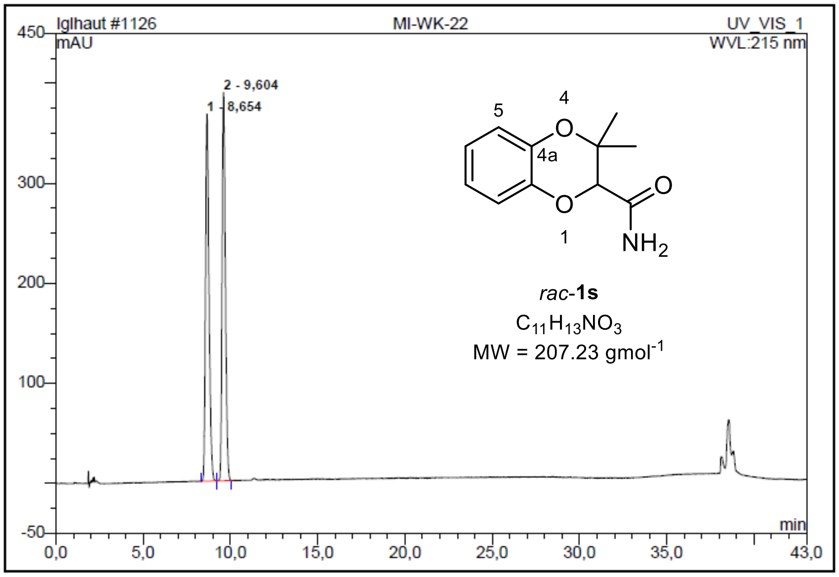

No.	Ret.Time min	Peak Name	Height mAU	Area mAU*min	Rel.Area %	Amount	Type
1	8,65	n.a.	366,848	85,077	50,04	n.a.	BM
2	9,60	n.a.	388,079	84,925	49,96	n.a.	MB
Total:			754,927	170,002	100,00	0,000	

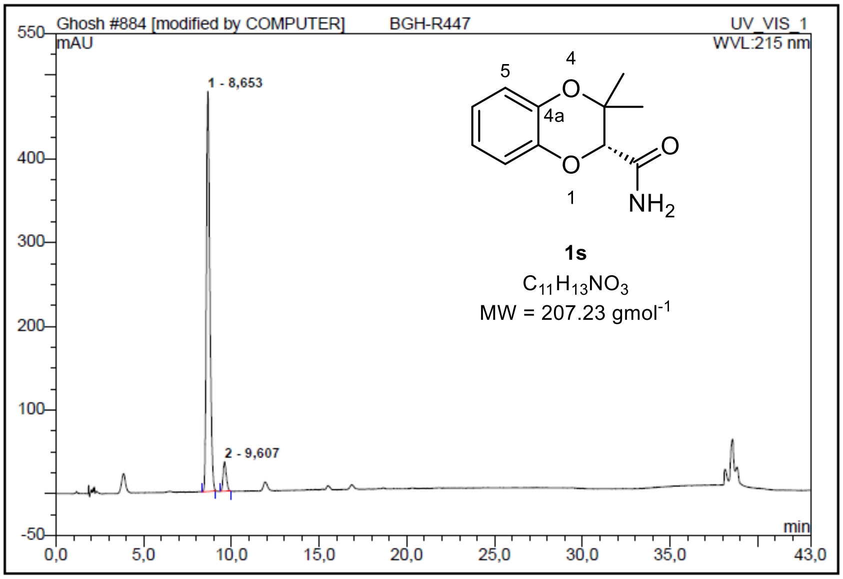

No.	Ret.Time min	Peak Name	Height mAU	Area mAU*min	Rel.Area %	Amount	Type
1	8,65	n.a.	478,052	108,951	93,68	n.a.	BMB
2	9,61	n.a.	35,108	7,349	6,32	n.a.	BMB*
Total:			513,160	116,300	100,00	0,000	

(*R*)-2,3-Dihydrobenzo[*b*][1,4]oxazine-2-carboxamide (1t)

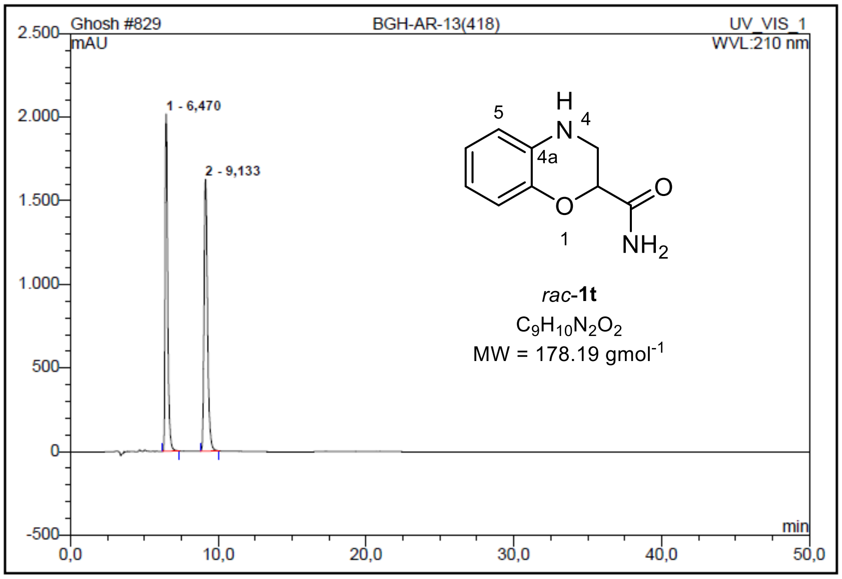

No.	Ret.Time min	Peak Name	Height mAU	Area mAU*min	Rel.Area %	Amount	Type
1	6,47	n.a.	2016,801	409,534	48,36	n.a.	BMB
2	9,13	n.a.	1624,810	437,317	51,64	n.a.	BMB
Total:			3641,610	846,851	100,00	0,000	

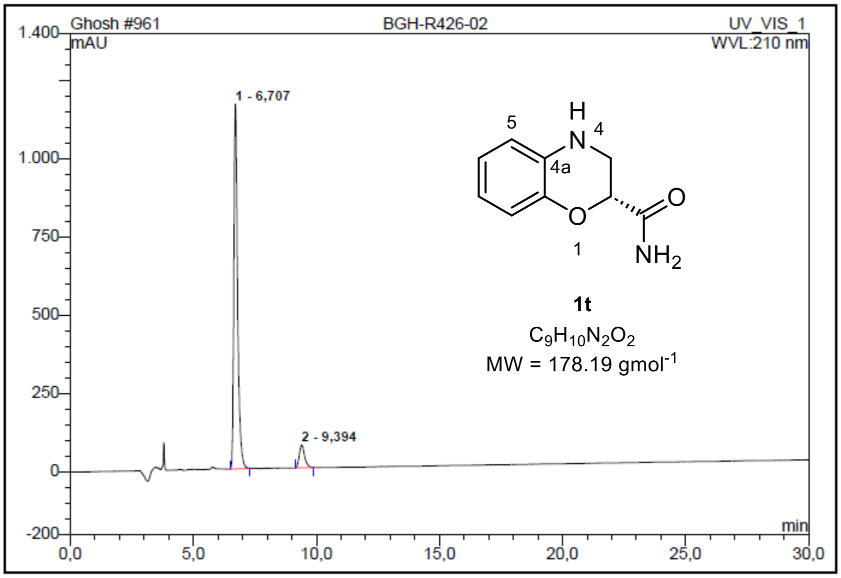

No.	Ret.Time min	Peak Name	Height mAU	Area mAU*min	Rel.Area %	Amount	Type
1	6,71	n.a.	1163,873	204,397	91,73	n.a.	BMB
2	9,39	n.a.	73,548	18,435	8,27	n.a.	BMB
Total:			1237,421	222,832	100,00	0,000	

(*R*)-4-Boc-2,3-dihydrobenzo[*b*][1,4]oxazine-2-carboxamide (1u)

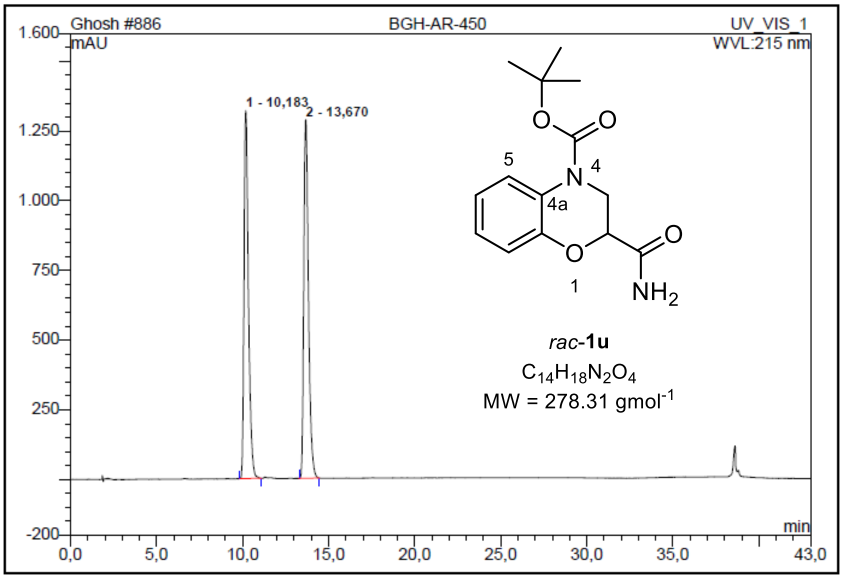

No.	Ret.Time min	Peak Name	Height mAU	Area mAU*min	Rel.Area %	Amount	Type
1	10,18	n.a.	1320,033	393,884	49,31	n.a.	BMB
2	13,67	n.a.	1286,229	404,851	50,69	n.a.	BMB
Total:			2606,262	798,735	100,00	0,000	

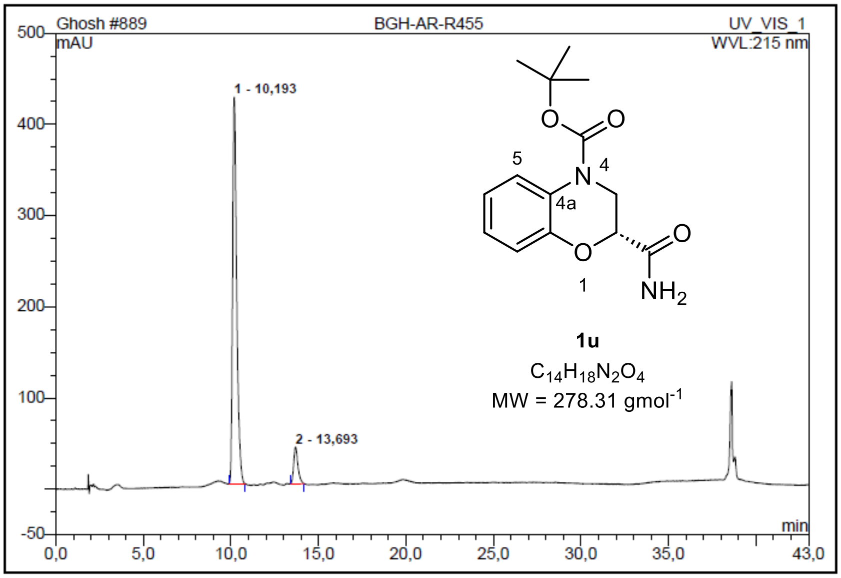

No.	Ret.Time min	Peak Name	Height mAU	Area mAU*min	Rel.Area %	Amount	Type
1	10,19	n.a.	424,426	115,306	90,99	n.a.	BMB
2	13,69	n.a.	40,445	11,419	9,01	n.a.	BMB
Total:			464,871	126,724	100,00	0,000	

cis-3-Methyl-1,4-benzodioxane-2-carboxamide (*cis*-1v)

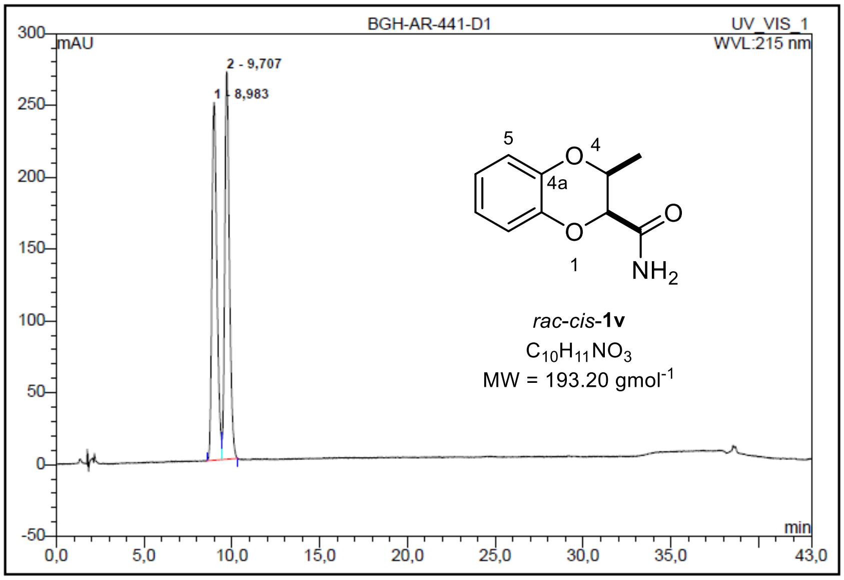

No.	Ret.Time min	Peak Name	Height mAU	Area mAU*min	Rel.Area %	Amount	Type
1	8,98	n.a.	249,121	81,969	49,85	n.a.	BM
2	9,71	n.a.	269,815	82,462	50,15	n.a.	MB
Total:			518,936	164,432	100,00	0,000	

After stereochemical editing of *rac-cis*-1v:

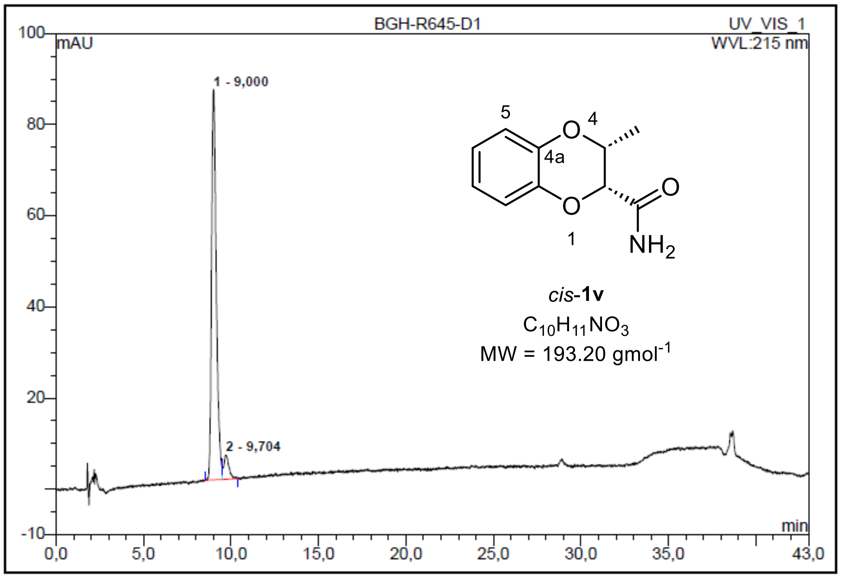

No.	Ret.Time min	Peak Name	Height mAU	Area mAU*min	Rel.Area %	Amount	Type
1	9,00	n.a.	85,671	26,724	93,57	n.a.	BM *
2	9,70	n.a.	5,277	1,836	6,43	n.a.	MB*
Total:			90,948	28,560	100,00	0,000	

After Stereochemical Editing of *rac-trans*-1v

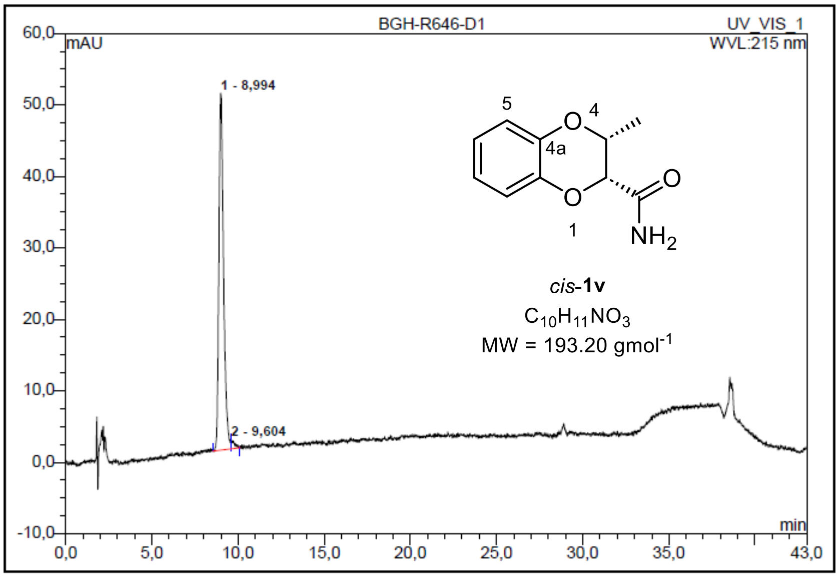

No.	Ret.Time min	Peak Name	Height mAU	Area mAU*min	Rel.Area %	Amount	Type
1	8,99	n.a.	49,927	15,828	98,06	n.a.	BM *
2	9,60	n.a.	1,256	0,313	1,94	n.a.	MB*
Total:			51,184	16,141	100,00	0,000	

***trans*-3-Methyl-1,4-benzodioxane-2-carboxamide (*trans*-1v)**

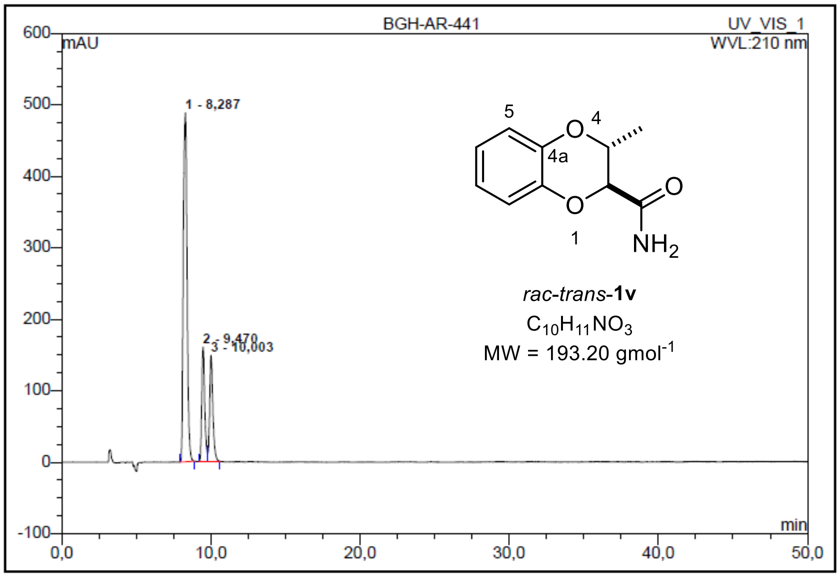

No.	Ret.Time min	Peak Name	Height mAU	Area mAU*min	Rel.Area %	Amount	Type
1	8,29	n.a.	488,090	142,604	65,76	n.a.	BMB
2	9,47	n.a.	160,331	36,514	16,84	n.a.	BM
3	10,00	n.a.	148,582	37,748	17,41	n.a.	MB
Total:			797,003	216,866	100,00	0,000	

After stereochemical editing of *rac-cis*-1v:

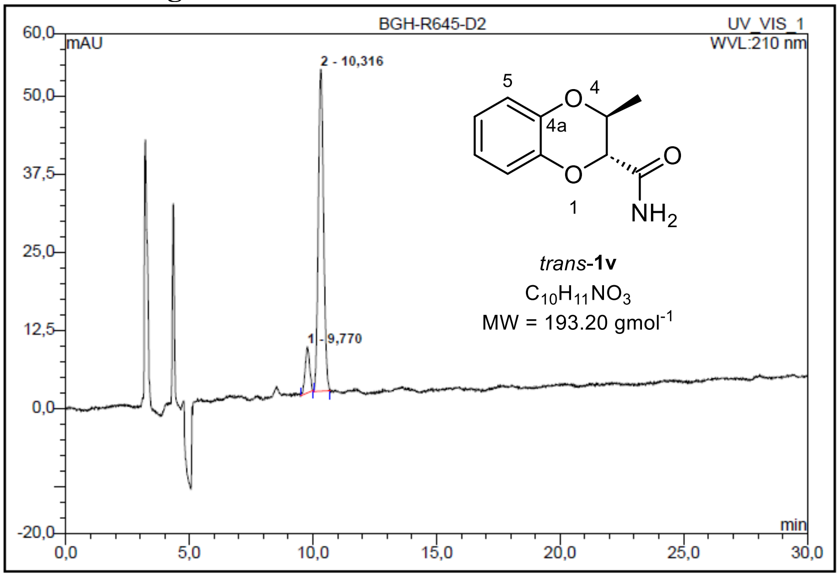

No.	Ret.Time min	Peak Name	Height mAU	Area mAU*min	Rel.Area %	Amount	Type
1	9,77	n.a.	7,395	1,555	10,92	n.a.	BMB*
2	10,32	n.a.	51,482	12,687	89,08	n.a.	BMB
Total:			58,877	14,243	100,00	0,000	

After stereochemical editing of *rac-trans*-1v:

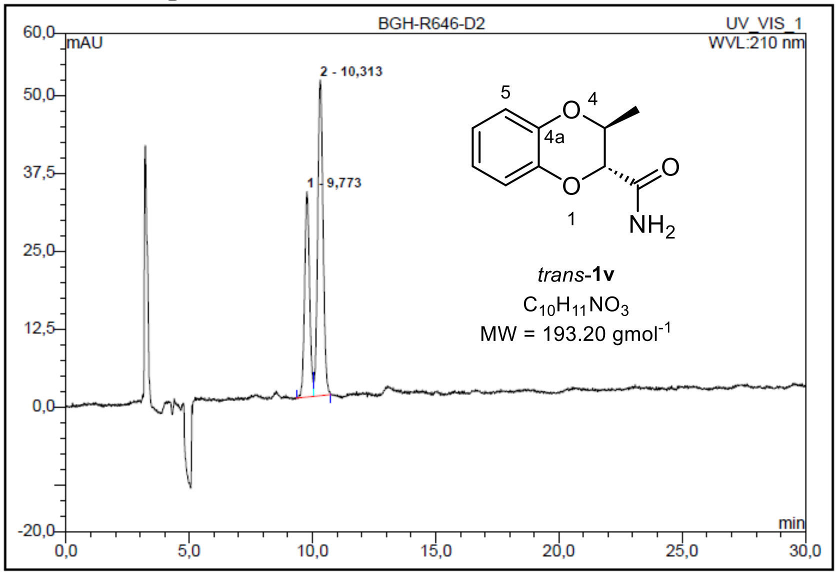

No.	Ret.Time min	Peak Name	Height mAU	Area mAU*min	Rel.Area %	Amount	Type
1	9,77	n.a.	32,946	7,732	37,84	n.a.	BM *
2	10,31	n.a.	50,674	12,699	62,16	n.a.	MB*
Total:			83,620	20,431	100,00	0,000	

(*R*)-1-(Chroman-2-yl)-*N*-((5-(4-fluorophenyl)pyridin-3-yl)methyl)methanamine (Sarizotan)

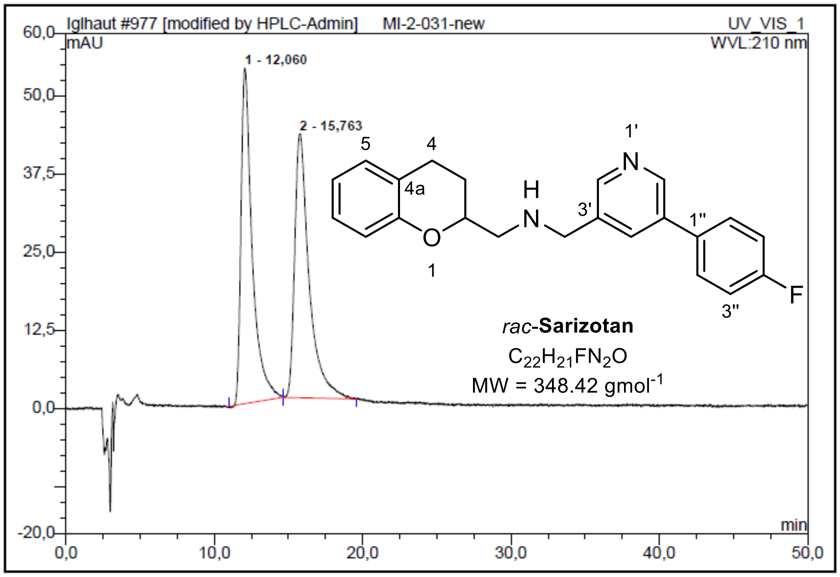

No.	Ret.Time min	Peak Name	Height mAU	Area mAU*min	Rel.Area %	Amount	Type
1	12,06	n.a.	53,595	46,837	49,64	n.a.	BMb*
2	15,76	n.a.	42,212	47,511	50,36	n.a.	bMB*
Total:			95,807	94,347	100,00	0,000	

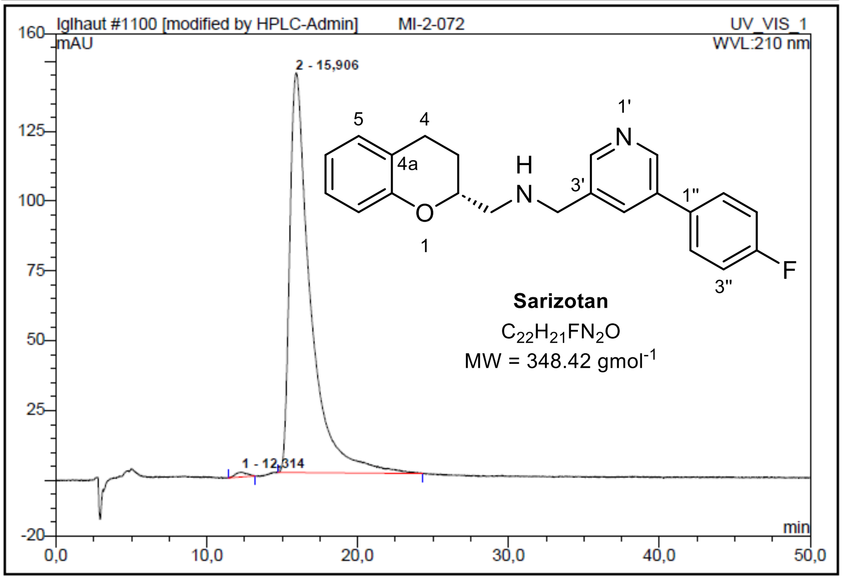

No.	Ret.Time min	Peak Name	Height mAU	Area mAU*min	Rel.Area %	Amount	Type
1	12,31	n.a.	1,733	1,530	0,67	n.a.	BMB*
2	15,91	n.a.	143,116	228,287	99,33	n.a.	BMB*
Total:			144,849	229,817	100,00	0,000	

1-Boc-(*R*)-4-(1,4-benzodioxane-2-carbonyl)piperazine (7)

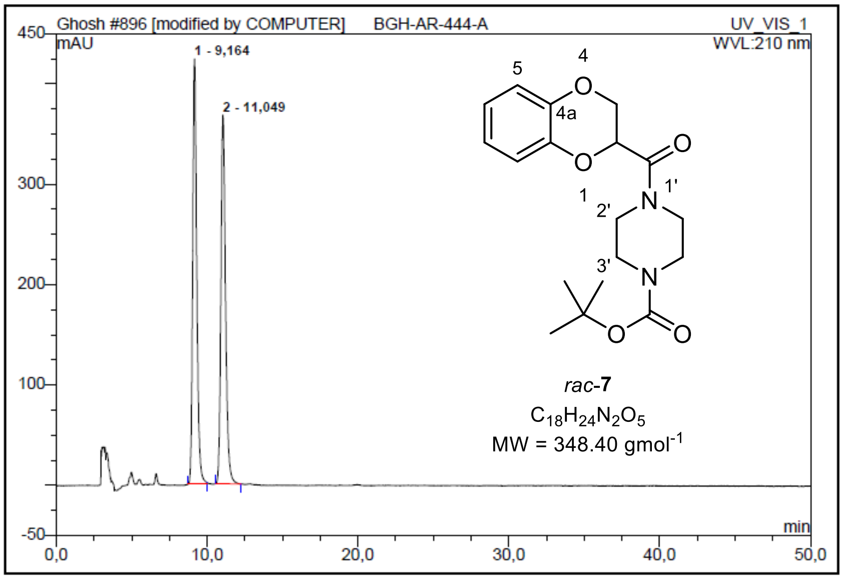

No.	Ret.Time min	Peak Name	Height mAU	Area mAU*min	Rel.Area %	Amount	Type
1	9,16	n.a.	423,485	131,565	50,08	n.a.	BM *
2	11,05	n.a.	367,160	131,133	49,92	n.a.	BMB*
Total:			790,645	262,698	100,00	0,000	

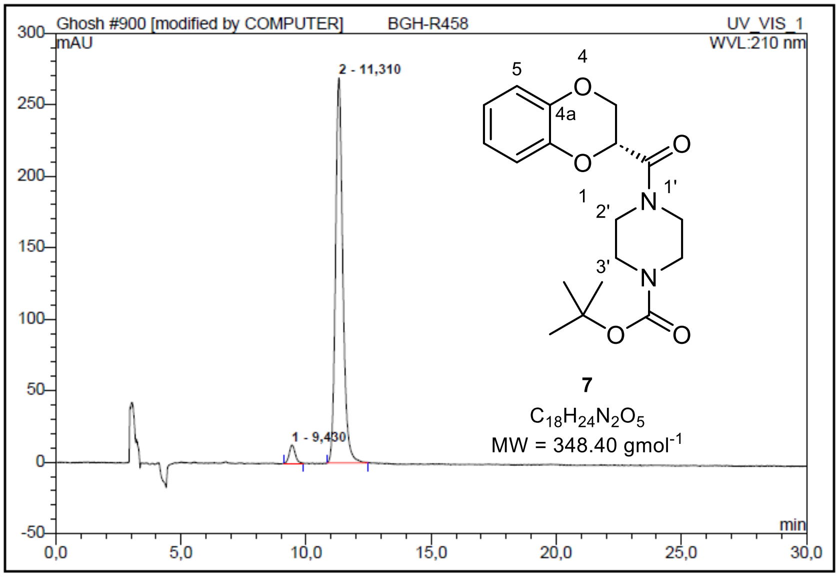

No.	Ret.Time min	Peak Name	Height mAU	Area mAU*min	Rel.Area %	Amount	Type
1	9,43	n.a.	13,052	3,592	3,79	n.a.	BMB*
2	11,31	n.a.	269,342	91,233	96,21	n.a.	BMB*
Total:			282,394	94,825	100,00	0,000	

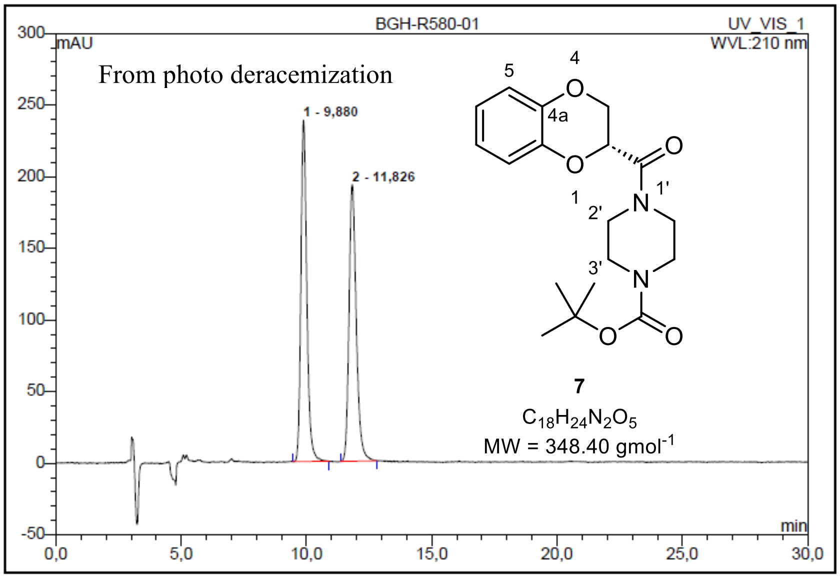

7
$C_{18}H_{24}N_2O_5$
MW = 348.40 gmol^{-1}

No.	Ret.Time min	Peak Name	Height mAU	Area mAU*min	Rel.Area %	Amount	Type
1	9,88	n.a.	238,384	65,167	50,16	n.a.	BMB*
2	11,83	n.a.	193,072	64,760	49,84	n.a.	BMB*
Total:			431,456	129,927	100,00	0,000	

(*R*)-4-(1,4-Benzodioxane-2-carbonyl)piperazine (S-7)

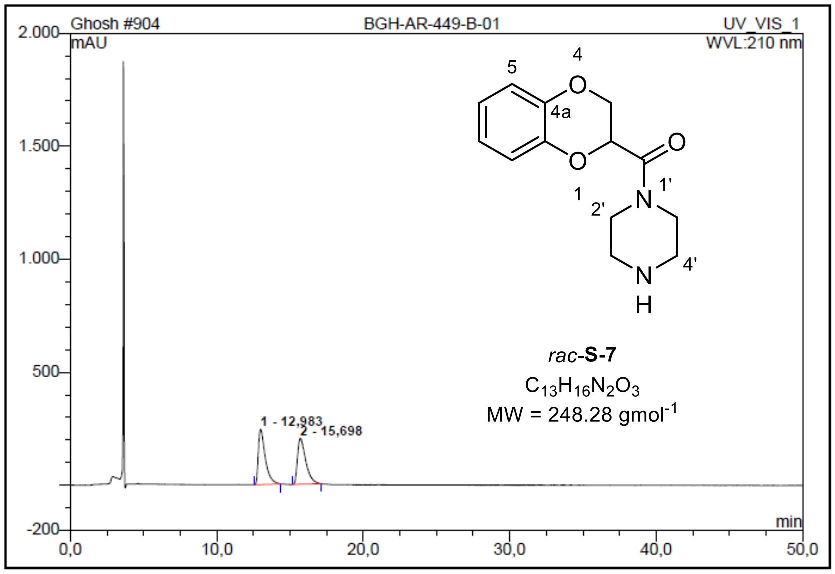

No.	Ret.Time min	Peak Name	Height mAU	Area mAU*min	Rel.Area %	Amount	Type
1	12,98	n.a.	244,394	134,677	50,37	n.a.	BMB
2	15,70	n.a.	202,404	132,723	49,63	n.a.	BMB
Total:			446,797	267,400	100,00	0,000	

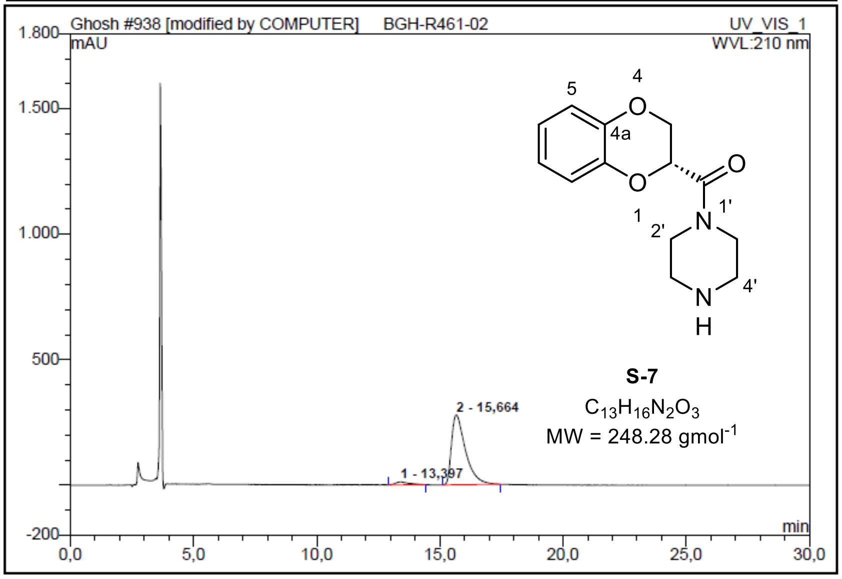

No.	Ret.Time min	Peak Name	Height mAU	Area mAU*min	Rel.Area %	Amount	Type
1	13,40	n.a.	11,030	6,947	3,65	n.a.	BMB*
2	15,66	n.a.	278,013	183,244	96,35	n.a.	BMB
Total:			289,042	190,191	100,00	0,000	

(*R*)-2-(Benzylamino)-2-((*S*)-6-fluorochroman-2-yl)ethan-1-ol (9)

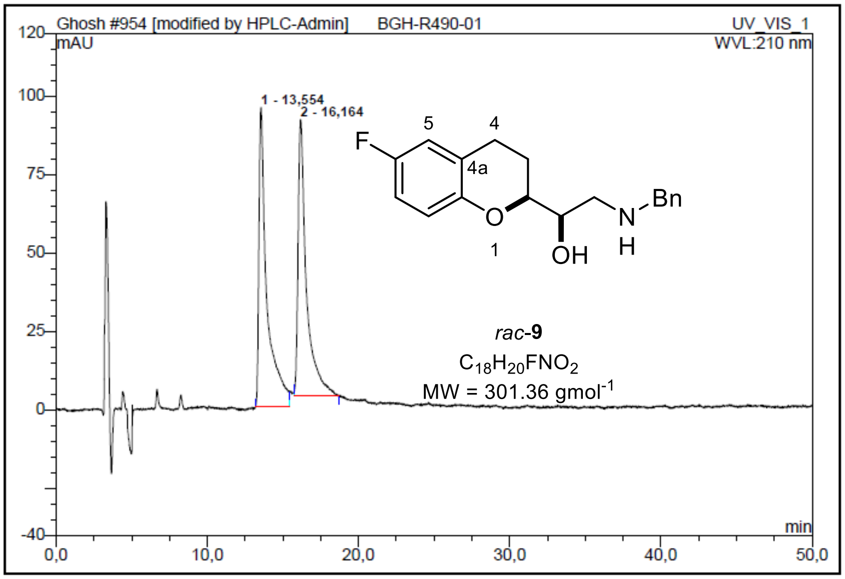

No.	Ret.Time min	Peak Name	Height mAU	Area mAU*min	Rel.Area %	Amount	Type
1	13,55	n.a.	95,376	58,434	50,94	n.a.	BM *
2	16,16	n.a.	87,982	56,279	49,06	n.a.	MB*
Total:			183,358	114,713	100,00	0,000	

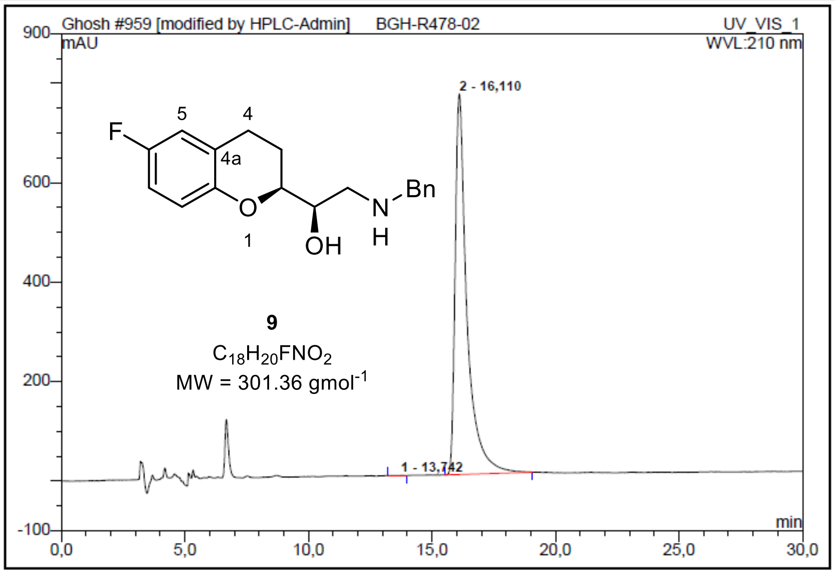

No.	Ret.Time min	Peak Name	Height mAU	Area mAU*min	Rel.Area %	Amount	Type
1	13,74	n.a.	0,722	0,271	0,06	n.a.	BMB*
2	16,11	n.a.	765,235	425,101	99,94	n.a.	BMB*
Total:			765,957	425,373	100,00	0,000	

(*S*)-6-Fluoro-4-oxochromane-2-carboxamide (11)

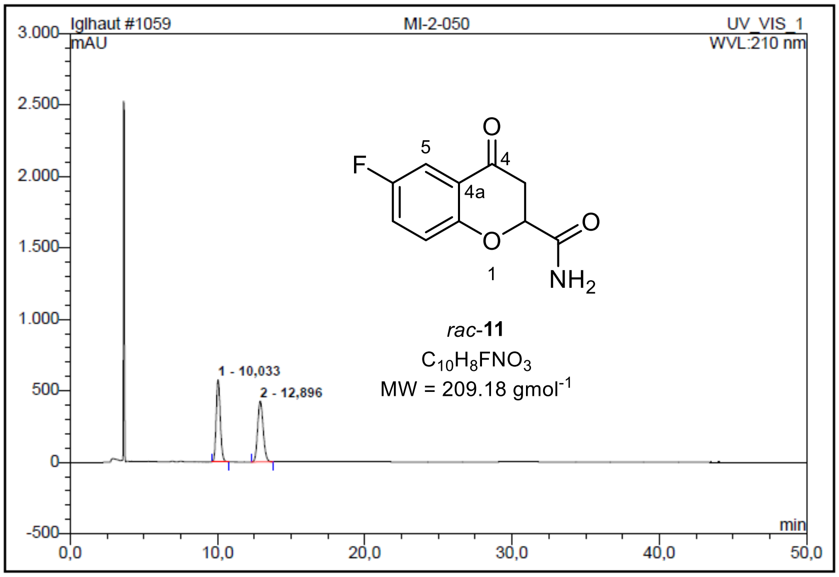

No.	Ret.Time min	Peak Name	Height mAU	Area mAU*min	Rel.Area %	Amount	Type
1	10,03	n.a.	573,791	184,679	49,90	n.a.	BMB
2	12,90	n.a.	425,067	185,408	50,10	n.a.	BMB
Total:			998,858	370,087	100,00	0,000	

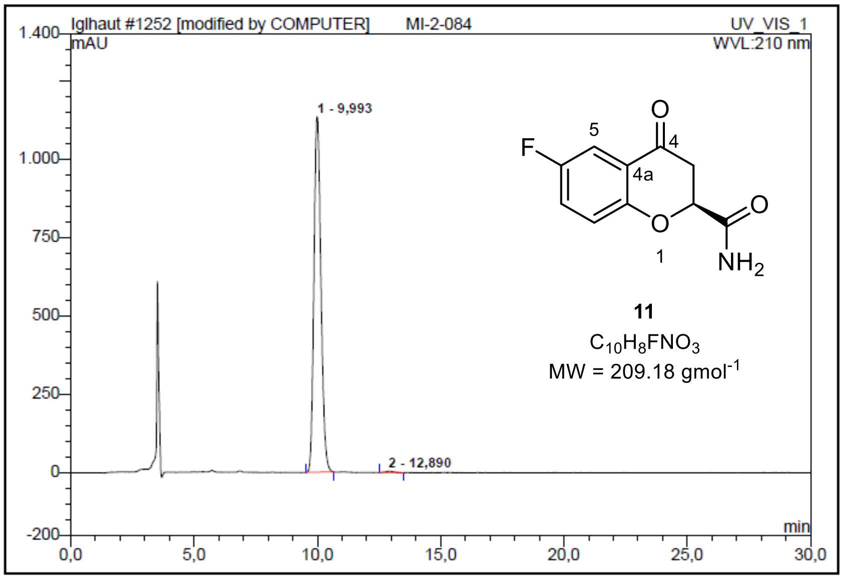

No.	Ret.Time min	Peak Name	Height mAU	Area mAU*min	Rel.Area %	Amount	Type
1	9,99	n.a.	1133,104	372,804	99,55	n.a.	BMB
2	12,89	n.a.	3,777	1,671	0,45	n.a.	BMB*
Total:			1136,880	374,475	100,00	0,000	

1,2,3,4-Tetrahydronaphthalene-2-carboxamide (12)

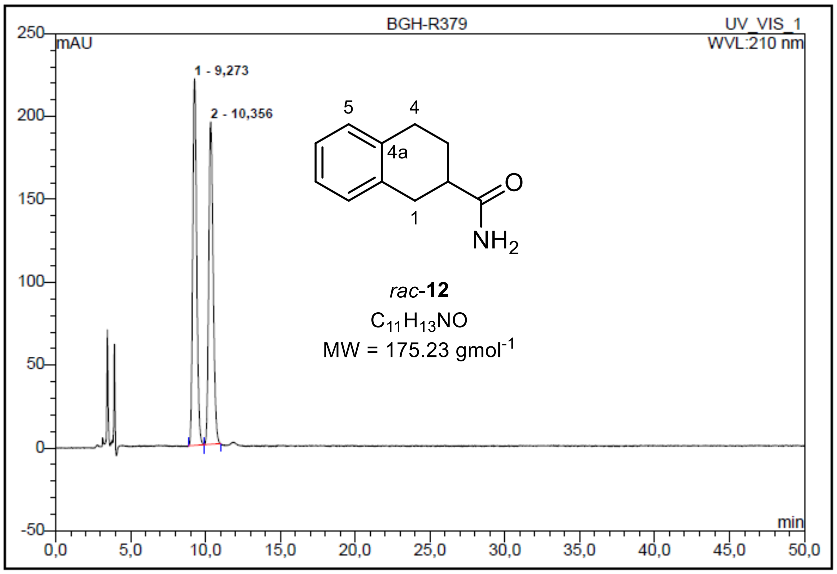

No.	Ret.Time min	Peak Name	Height mAU	Area mAU*min	Rel.Area %	Amount	Type
1	9,27	n.a.	221,021	67,514	50,17	n.a.	BM
2	10,36	n.a.	194,401	67,059	49,83	n.a.	MB
Total:			415,422	134,573	100,00	0,000	

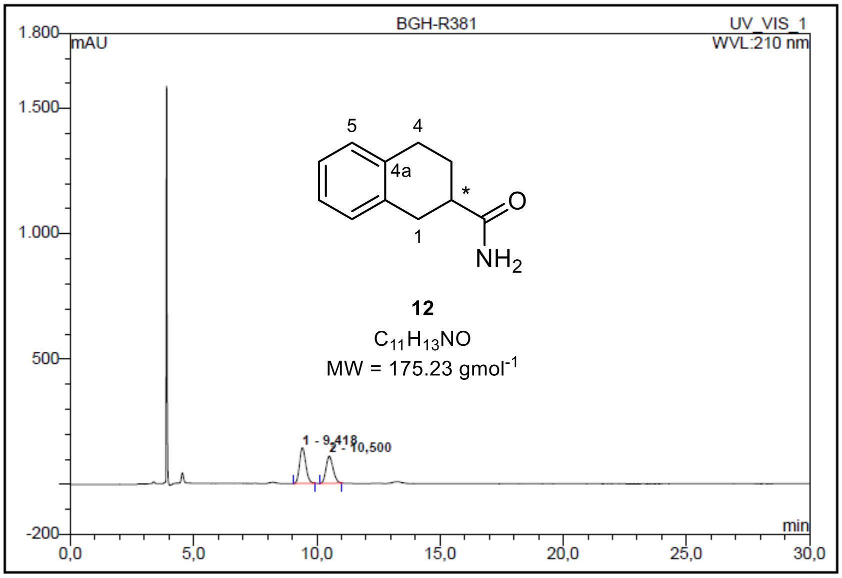

No.	Ret.Time min	Peak Name	Height mAU	Area mAU*min	Rel.Area %	Amount	Type
1	9,42	n.a.	141,684	41,576	53,66	n.a.	BMB
2	10,50	n.a.	107,951	35,910	46,34	n.a.	BMB
Total:			249,635	77,486	100,00	0,000	

Thiochromane-2-carboxamide (13)

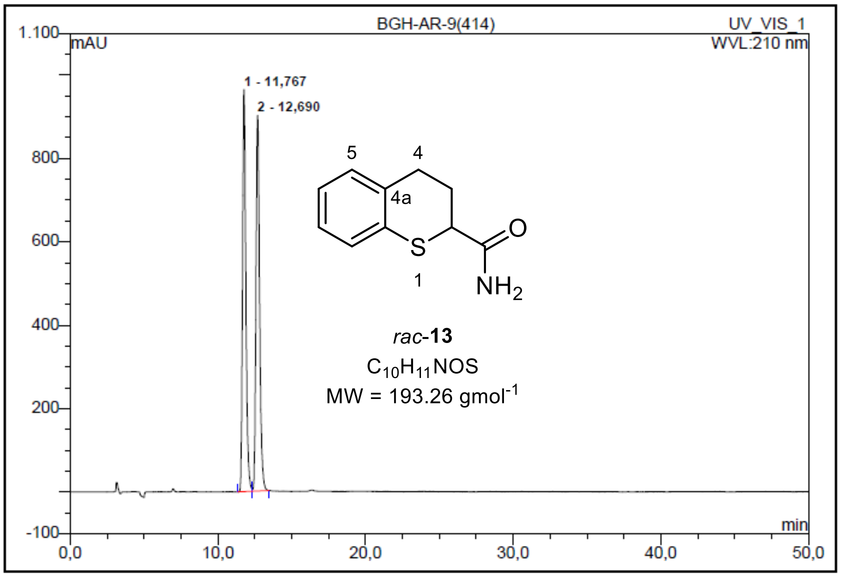

No.	Ret.Time min	Peak Name	Height mAU	Area mAU*min	Rel.Area %	Amount	Type
1	11,77	n.a.	963,917	253,971	49,94	n.a.	BM
2	12,69	n.a.	900,478	254,557	50,06	n.a.	MB
Total:			1864,396	508,528	100,00	0,000	

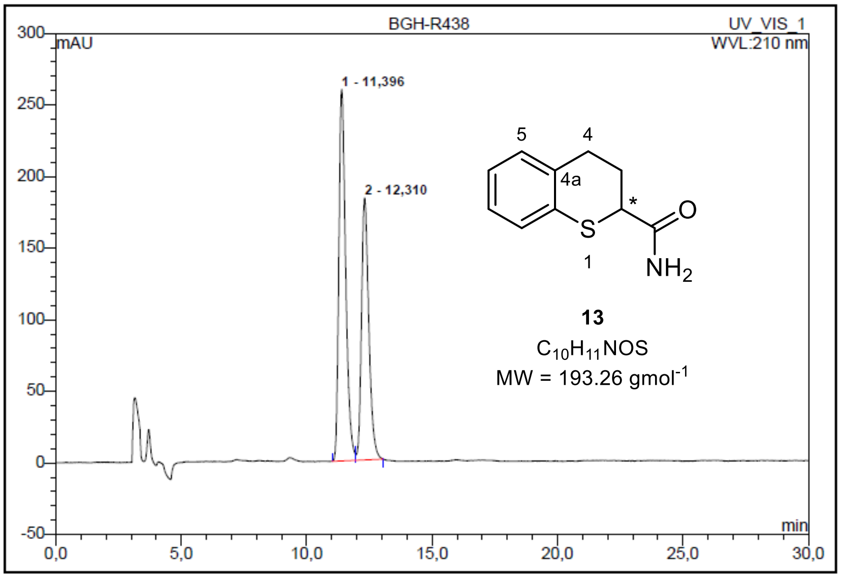

No.	Ret.Time min	Peak Name	Height mAU	Area mAU*min	Rel.Area %	Amount	Type
1	11,40	n.a.	259,522	82,267	57,06	n.a.	BM
2	12,31	n.a.	182,945	61,922	42,94	n.a.	MB
Total:			442,468	144,189	100,00	0,000	

S15. List of References

[56] S. K. Sabui, P. Mondal, R. V. Venkateswaran, *J. Chem. Res.* **2002**, *2002*, 428-429.

[57] F. Wakita, Y. Ando, K. Ohmori, K. Suzuki, *Org. Lett.* **2018**, *20*, 3928-3932.

[58] I. Marco, M. Valhondo, M. Martín-Fontecha, H. Vazquez-Villa, J. n. Del Río, A. Planas, O. Sagredo, J. A. Ramos, I. R. Torrecillas, L. Pardo, *J. Med. Chem.* **2011**, *54*, 7986-7999.

[59] M. Plaza, J. Großkopf, S. Breitenlechner, C. Bannwarth, T. Bach, *J. Am. Chem. Soc.* **2021**, *143*, 11209-11217.

[60] Grimme lab, "Semiempirical Extended Tight-Binding Program Package (xtb)" can be found under https://github.com/grimme-lab/xtb, **2024**.

[61] Grimme lab, "Conformer-Rotamer Ensemble Sampling Tool (CREST)" can be found under https://github.com/grimme-lab/crest, **2025**.

[62] S. Grimme, C. Bannwarth, E. Caldeweyher, J. Pisarek, A. Hansen, *J. Chem. Phys.* **2017**, *147*, 161708–161721.

[63] S. Grimme, C. Bannwarth, P. Shushkov, *J. Chem. Theory Comput.* **2017**, *13*, 1989-2009.

[64] P. Pracht, F. Bohle, S. Grimme, *Phys. Chem. Chem. Phys.* **2020**, *22*, 7169-7192.

[65] Grimme lab, "Ensemble Sorting" can be found under, https://crest-lab.github.io/crest-docs/page/examples/example_2.html, **2024**.

[66] F. Neese, *WIREs Comput. Mol. Sci.* **2022**, *12*, e1606.

[67] J. P. Perdew, K. Burke, M. Ernzerhof, *Phys. Rev. Lett.* **1996**, *77*, 3865-3868.

[68] S. Grimme, J. Antony, S. Ehrlich, H. Krieg, *J. Chem. Phys.* **2010**, *132*, 154104–154123.

[69] S. Grimme, S. Ehrlich, L. Goerigk, *J. Comput. Chem.* **2011**, *32*, 1456-1465.

[70] H. Kruse, S. Grimme, *J. Chem. Phys.* **2012**, *136*, 154101–154117.

[71] M. Garcia-Ratés, F. Neese, *J. Comput. Chem.* **2020**, *41*, 922-939.

[72] O. Vahtras, J. Almlöf, M. Feyereisen, *Chem. Phys. Lett.* **1993**, *213*, 514-518.

[73] F. Neese, *J. Comput. Chem.* **2003**, *24*, 1740-1747.

[74] F. Weigend, *Phys. Chem. Chem. Phys.* **2006**, *8*, 1057-1065.

[75] F. Neese, F. Wennmohs, A. Hansen, U. Becker, *Chem. Phys.* **2009**, *356*, 98-109.

[76] B. Helmich-Paris, B. de Souza, F. Neese, R. Izsák, *J. Chem. Phys.* **2021**, *155*, 104109-104123.

[77] S. Grimme, *Chem. Eur. J.* **2012**, *18*, 9955-9964.

[78] S. Spicher, S. Grimme, *J. Phys. Chem. Lett.* **2020**, *11*, 6606-6611.

[79] R. Siewert, K. V. Zherikova, S. P. Verevkin, *Chem. Eur. J.* **2022**, *28*, e202200080.

[80] S. M. Hussain, R. Kumar, M. M. N. Ali, D. Sankar, V. Kannappan, *J. Mol. Liq.* **2022**, *345*, 117806-117818.

[81] C. M. Marian, A. Heil, M. Kleinschmidt, *WIREs Comput. Mol. Sci.* **2019**, *9*, e1394.

[82] S. Grimme, M. Waletzke, *J. Chem. Phys.* **1999**, *111*, 5645-5655.

[83] A. Heil, M. Kleinschmidt, C. M. Marian, *J. Chem. Phys.* **2018**, *149*, 164106-164122.

[84] M. Kleinschmidt, C. M. Marian, M. Waletzke, S. Grimme, *J. Chem. Phys.* **2009**, *130*, 044708-044719.

[85] A. D. Becke, *J. Chem. Phys.* **1993**, *98*, 1372-1377.

[86] A. Hellweg, C. Hättig, S. Höfener, W. Klopper, *Theor. Chem. Acc.* **2007**, *117*, 587-597.

[87] J. Baker, *J. Comput. Chem.* **1986**, *7*, 385-395.

[88] K. Ishida, K. Morokuma, A. Komornicki, *J. Chem. Phys.* **1977**, *66*, 2153-2156.

[89] P. Pracht, C. Bannwarth, *J. Phys. Chem. Lett.* **2023**, *14*, 4440-4448.

[90] N. van Staalduinen, C. Bannwarth, *Digital Discovery* **2024**, *3*, 2298-2319.

[91] BannwarthLab, "MolBar: A Molecular Identifier for Inorganic and Organic Molecules with Full Support of Stereoisomerism" can be found under, https://git.rwth-aachen.de/bannwarthlab/molbar, **2024**.

[92] S. Seritan, C. Bannwarth, B. S. Fales, E. G. Hohenstein, C. M. Isborn, S. I. L. Kokkila-Schumacher, X. Li, F. Liu, N. Luehr, J. W. Snyder Jr., C. Song, A. V. Titov, I. S. Ufimtsev, L.-P. Wang, T. J. Martínez, *WIREs Comput. Mol. Sci.* **2021**, *11*, e1494.

[93] B. Bandyopadhyay, P. Pandey, P. Banerjee, A. K. Samanta, T. Chakraborty, *J. Phys. Chem. A* **2012**, *116*, 3836-3845.

[94] Y. Hussain, C. Empel, R. M. Koenigs, P. Chauhan, *Angew. Chem. Int. Ed.* **2023**, *62*, e202309184.

[95] C. Huang, J.-H. Guo, H.-M. Fu, *Heterocycles* **2015**, *91*, 1204-1211.

[96] S. Tummanapalli, S. K. Punna, K. C. Gulipalli, S. Endoori, S. Bodige, A. K. Pommidi, S. Medaboina, S. Choppadandi, R. Boya, V. K. Ganapathi, D. Y. Mamindla, R. Konakalla, G. K. Bodala, M. R. Bakangari, S. D. Kottam, D. Jarikote, T. Potewar, M. Valluri, *J. Org. Chem.* **2023**, *88*, 8387-8399.

[97] D. Sarkar, R. V. Venkateswaran, *Tetrahedron* **2011**, *67*, 4559-4568.

[98] C. Ortiz, F. Echeverri, S. Robledo, D. Lanari, M. Curini, W. Quiñones, E. Vargas, *Molecules* **2020**, *25*, 800-822.

[99] M. Spinck, M. Bischoff, P. Lampe, F.-J. Meyer-Almes, S. Sievers, H. Neumann, *J. Med. Chem.* **2021**, *64*, 5838-5849.

[100] W. Quaglia, M. Pigini, S. K. Tayebati, A. Piergentili, M. Giannella, G. Marucci, C. Melchiorre, *J. Med. Chem.* **1993**, *36*, 1520-1528.

[101] L. Yin, Y. Pan, Y. Xue, X. Chen, T. You, J. Huang, Q. Xu, Q. Hu, *J. Med. Chem.* **2022**, *65*, 11876-11888.

[102] N. Ullah, *Z. Naturforsch. B.* **2012**, *67*, 75-84.

[103] A. Rouf, P. Gupta, M. A. Aga, B. Kumar, A. Chaubey, R. Parshad, S. C. Taneja, *Tetrahedron: Asymm.* **2012**, *23*, 1615-1623.

[104] R. Devi, S. K. Das, *Beilstein J. Org. Chem.* **2017**, *13*, 571-578.

[105] M. R. Mannam, S. S, P. Kumar, N. R. Chamarthi, P. K. R. S, *Phosphorus Sulfur Silicon Relat. Elem.* **2020**, *195*, 65-74.

[106] M. R. Shukla, G. Sadasivam, A. Sarde, M. Sayyed, V. Pachpute, R. Phadtare, N. Walke, V. D. Chaudhari, R. Loriya, T. Khan, G. Gote, C. Pawar, M. Tryambake, N. Mahajan, A. Gandhe, S. Sabde, S. Pawar, V. Patil, D. Modi, M. Mehta, P. Nigade, V. Modak, R. Ghodke, L. Narasimham, M. Bhonde, J. Gundu, R. Goel, C. Shah, S. Kulkarni, S. Sharma, D. Bakhle, R. K. Kamboj, V. P. Palle, *J. Med. Chem.* **2023**, *66*, 9418-9444.
